# Supplementary material for: Exposure to formaldehyde and asthma outcomes: A systematic review, meta-analysis, and economic assessment
Source: PLoS One. 2021 Mar 31;16(3):e0248258. doi: 10.1371/journal.pone.0248258 (PMC8011796; doi:10.1371/journal.pone.0248258)
Supplement: S1 Fig — (DOCX) [file pone.0248258.s002.docx]

Supplemental Figure 1. Risk of bias ratings for prospective cohort studies, by year
