# Supplementary material for: Exposure to formaldehyde and asthma outcomes: A systematic review, meta-analysis, and economic assessment
Source: PLoS One. 2021 Mar 31;16(3):e0248258. doi: 10.1371/journal.pone.0248258 (PMC8011796; doi:10.1371/journal.pone.0248258)
Supplement: S2 Fig — (DOCX) [file pone.0248258.s003.docx]

Supplemental Figure 2. Risk of Bias Heat Map, By Study Population
