# Supplementary material for: Exposure to formaldehyde and asthma outcomes: A systematic review, meta-analysis, and economic assessment
Source: PLoS One. 2021 Mar 31;16(3):e0248258. doi: 10.1371/journal.pone.0248258 (PMC8011796; doi:10.1371/journal.pone.0248258)

Supplemental Figure 5. Scatterplot of Categorical Odds/Risk Ratios Not Included in Child Asthma Diagnosis Meta-Analysis


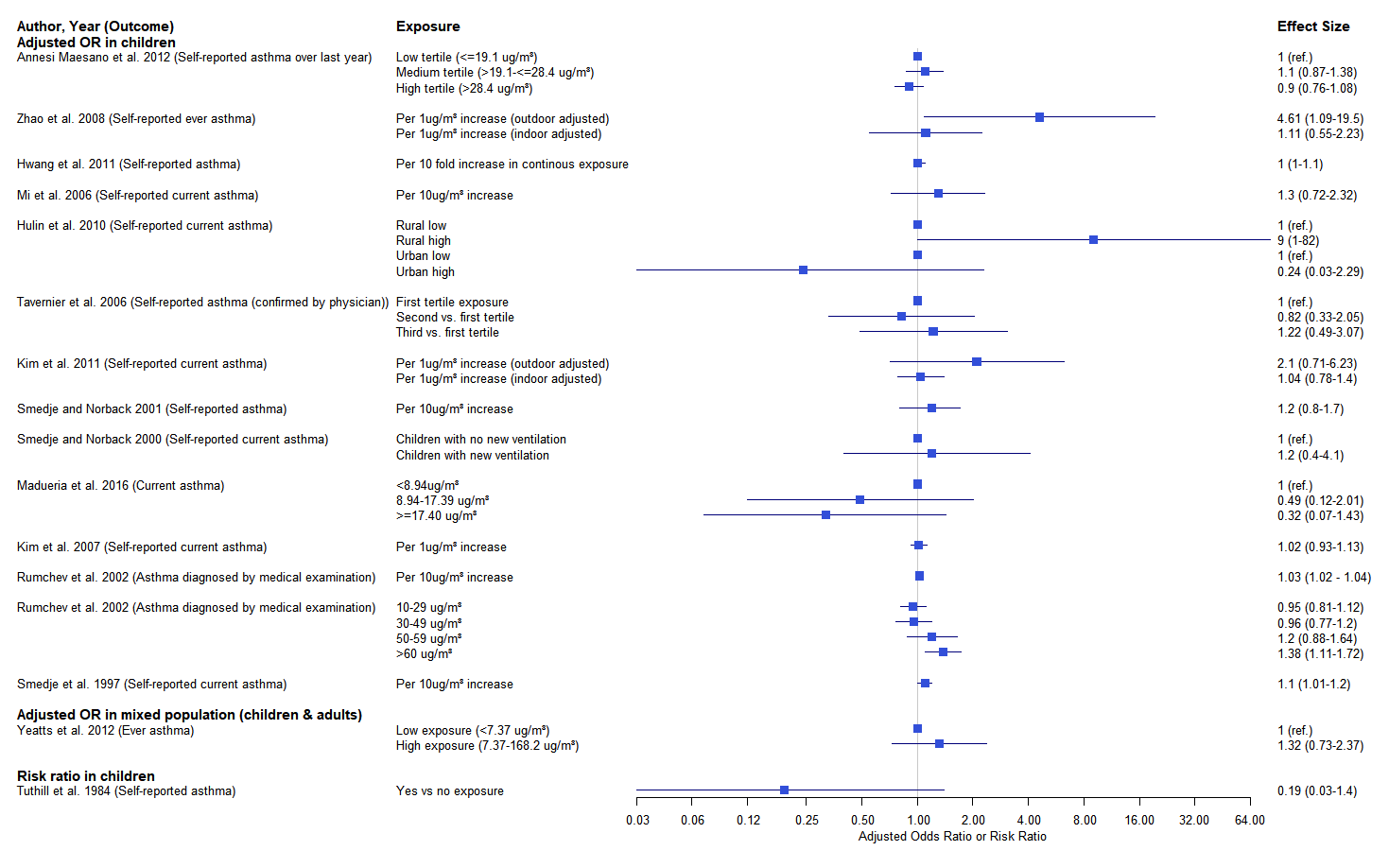

Supplement: S5 Fig — (DOCX) [file pone.0248258.s006.docx]
