# Supplementary figures and images for: Exposure to formaldehyde and asthma outcomes: A systematic review, meta-analysis, and economic assessment
Source: PLoS One. 2021 Mar 31;16(3):e0248258. doi: 10.1371/journal.pone.0248258 (PMC8011796; doi:10.1371/journal.pone.0248258)

Supplemental Figure 7. Scatterplot of child and adult formaldehyde exposures by asthma status


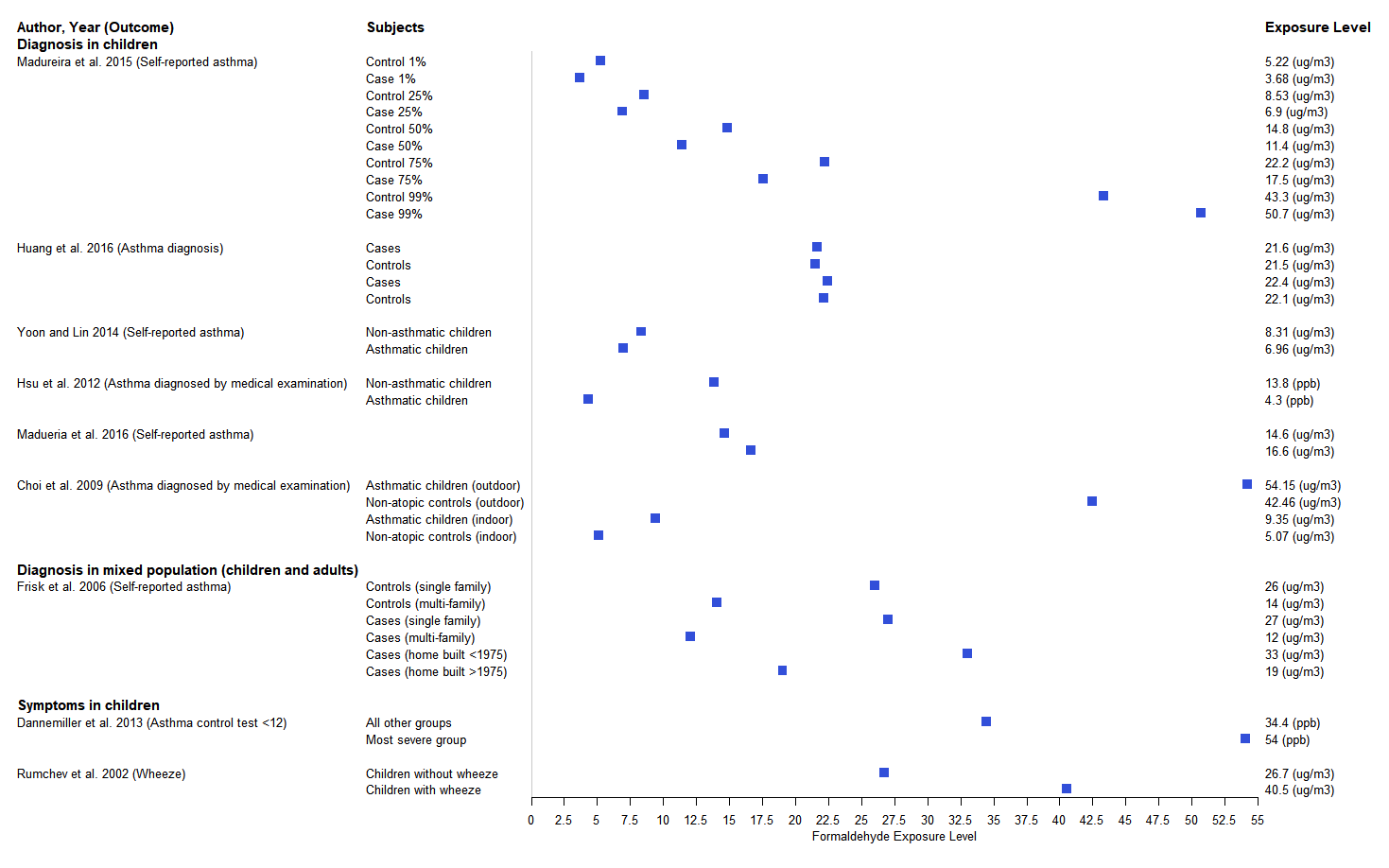

Supplement: S7 Fig — (DOCX) [file pone.0248258.s008.docx]

Supplemental Figure 10. Scatterplot of adult categorical odds ratios


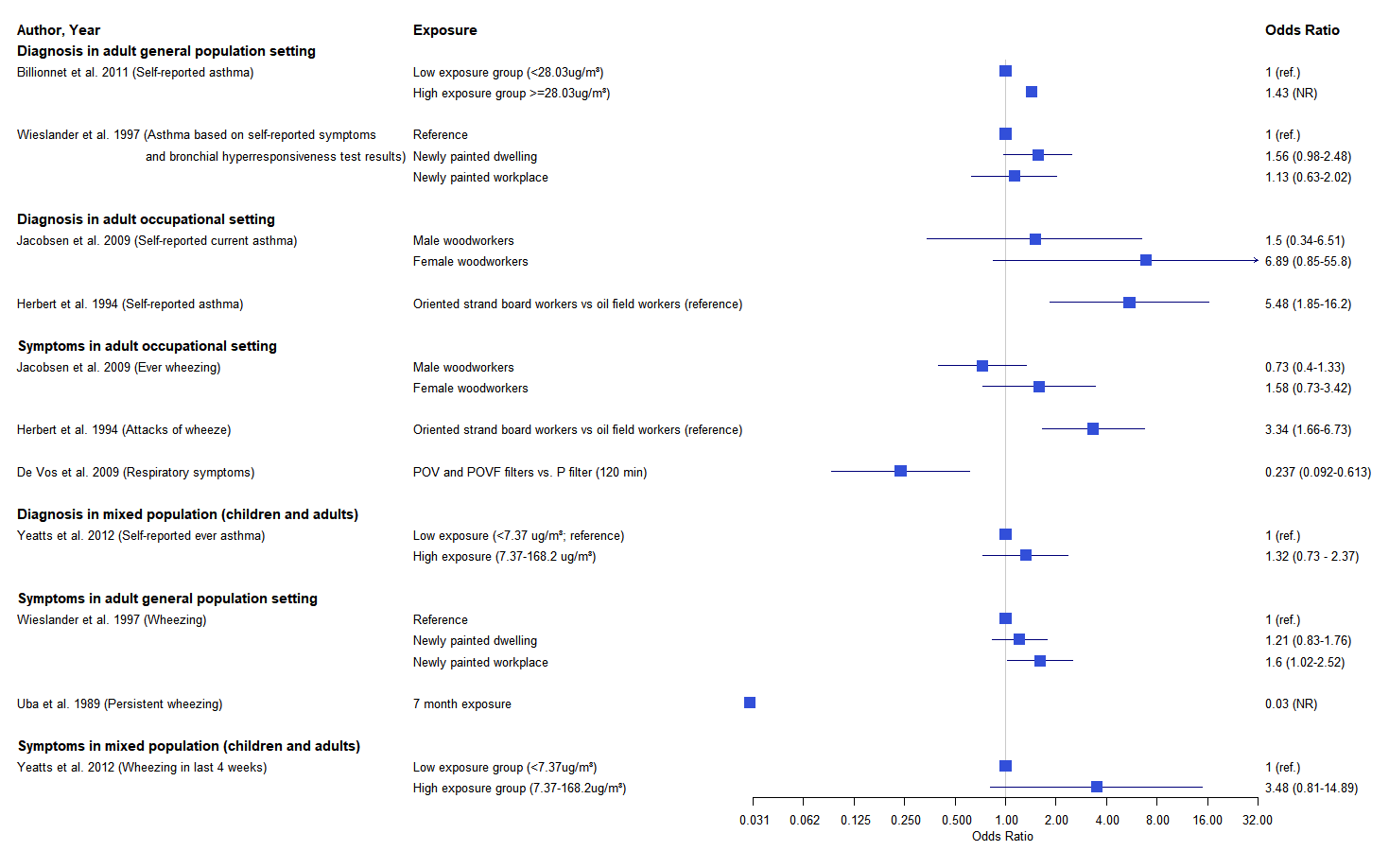

Supplement: S10 Fig — (DOCX) [file pone.0248258.s011.docx]

Supplemental Figure 11. Scatterplot of adult asthma prevalence


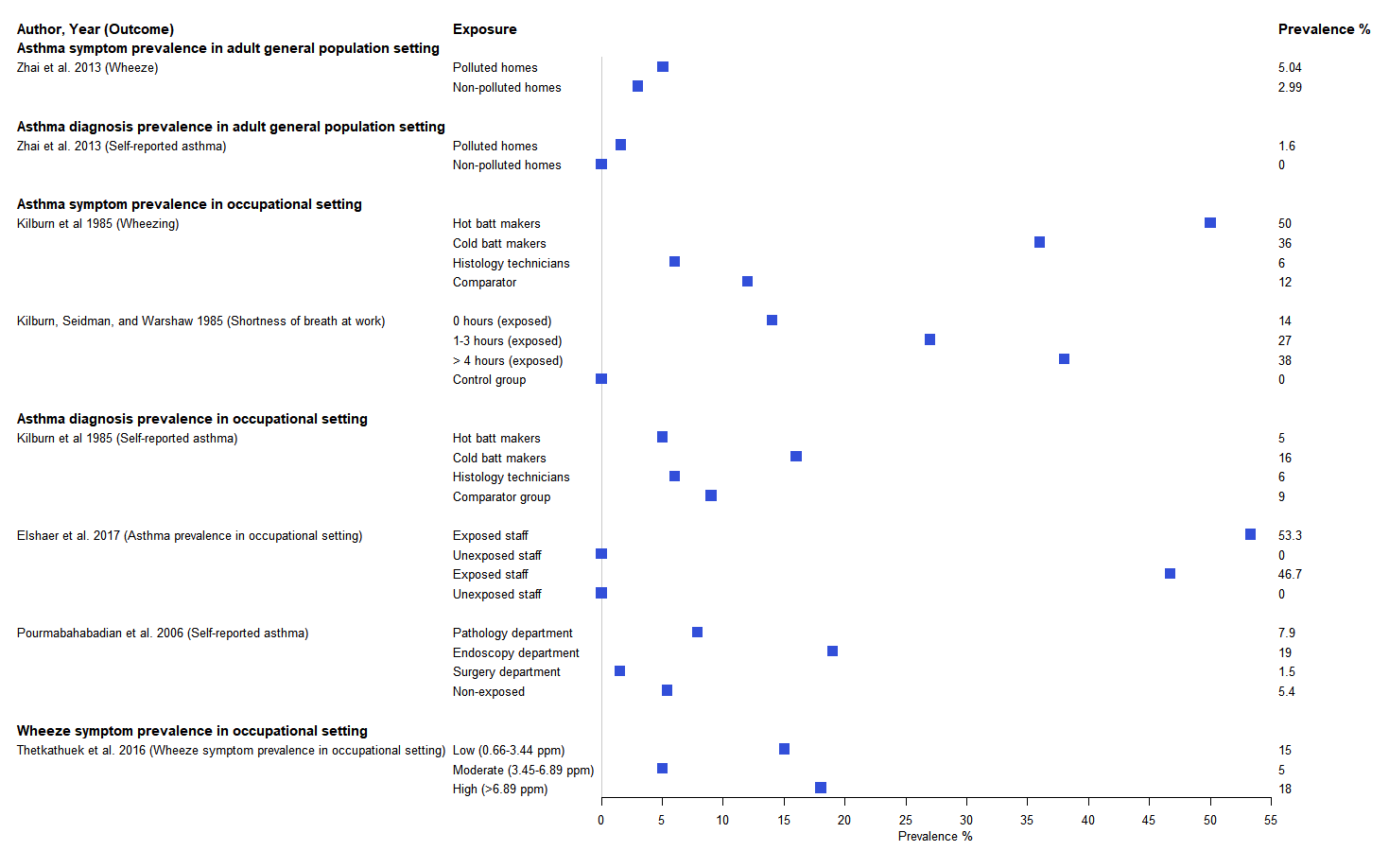

Supplement: S11 Fig — (DOCX) [file pone.0248258.s012.docx]

Supplemental Figure 12. Scatterplot of adult FEV measures


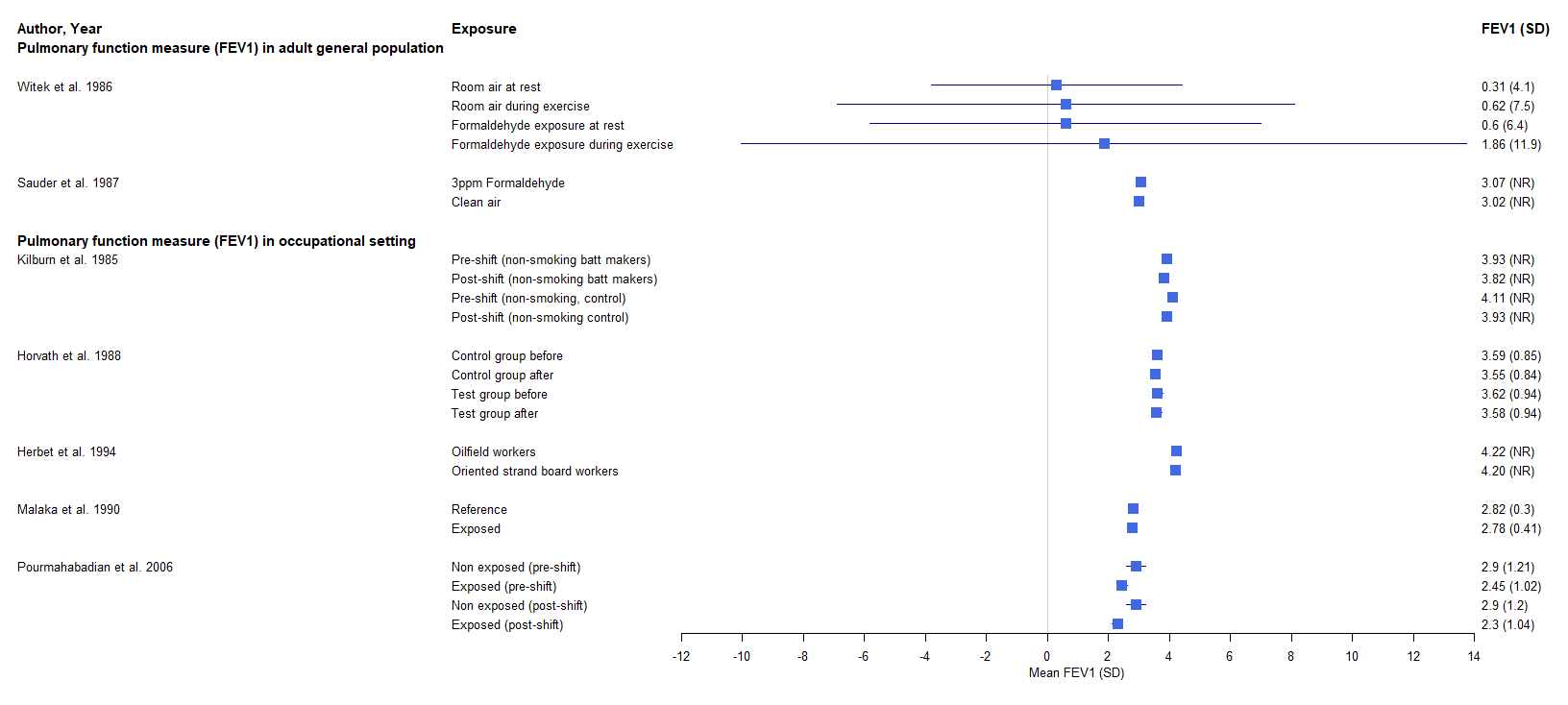

Supplement: S12 Fig — (DOCX) [file pone.0248258.s013.docx]
