# Supplementary material for: Exposure to formaldehyde and asthma outcomes: A systematic review, meta-analysis, and economic assessment
Source: PLoS One. 2021 Mar 31;16(3):e0248258. doi: 10.1371/journal.pone.0248258 (PMC8011796; doi:10.1371/journal.pone.0248258)
Supplement: S1 Table — (DOCX) [file pone.0248258.s014.docx]

Supplemental Table 1. Search Terms in PubMed

| **Search** | **PubMed** |
| --- | --- |
| #1 | ((("Asthma"[Mesh] OR asthma*[tiab] OR reactive airway[tiab] OR reactive airways[tiab] OR airway inflammation[tiab] OR wheeze[tiab] OR wheezes[tiab] OR wheezing[tiab] OR dyspnea[tiab] OR "Respiratory Function Tests"[MeSH Terms] OR "Respiratory Sounds"[Mesh:noexp] OR spirometry[tiab] OR lung function[tiab] OR lung functions[tiab] OR respiratory function[tiab] OR respiratory functions[tiab] OR pulmonary function[tiab] OR pulmonary functions[tiab] OR "Forced Expiratory Volume"[Mesh] OR "Peak Expiratory Flow Rate"[Mesh] OR "Forced Expiratory Volume"[Mesh] OR FEV1[tiab] OR PEFR[tiab] OR PEF[tiab] OR "peak expiratory"[tiab] OR "forced expiratory volume"[tiab] OR "forced expiratory flow"[tiab] OR "Bronchial Hyperreactivity"[MH] OR bronchial hyperreactivity[tiab] OR bronchial hyper-reactivity[tiab] OR bronchial hypersensitivity[tiab] OR bronchial hyper-sensitivity[tiab] OR "Bronchial Spasm"[Mesh] OR bronchial spasm[tiab] OR bronchial spasms[tiab] OR "Airway Resistance"[MH] OR "Airway Obstruction"[mh] OR airway obstruction[tiab] OR airway resistance[tiab] OR "Bronchoconstriction"[Mesh] OR bronchial constriction[tiab] OR bronchial constrictions[tiab] OR respiratory health[tiab] OR reactive airway disease[tiab]))) AND ((("Formaldehyde"[Mesh] OR 50-00-0[RN] OR Formaldehyde[tiab] OR Oxomethane[tiab] OR Methanal[tiab] OR Formol[tiab] OR Formalin[tiab] OR paraformaldehyde[tiab] OR Medium-density fibreboard[tiab] OR medium-density fiberboard[tiab] OR particleboard[tiab] OR particle-board[tiab] OR plywood[tiab] OR wood-based[tiab] OR wood-product*[tiab] OR composite-wood[tiab] OR pressed-wood[tiab] OR varnish* OR laminate[tiab] OR "Floors and Floorcoverings"[Mesh] OR flooring[tiab] OR "polyurethane foam"[Supplementary Concept] OR "polyurethane foam"[tiab] OR "urea formaldehyde foam"[Supplementary Concept] OR "urea formaldehyde foam"[tiab] OR "Adhesives"[Mesh] OR Adhesives[tiab] OR salon[tiab] OR hair-straighten*[tiab] OR hair-smooth*[tiab] OR (trailer*[tiab] AND (housing[tiab] OR "housing"[Mesh])) OR Travel-trailer*[tiab] OR manufactured-home*[tiab] OR mobile-home*[tiab] OR manufactured-hous*[tiab] OR modular-home*[tiab]) OR "temporary housing unit"[tiab] OR "temporary housing units"[tiab] OR (("Construction Materials"[Mesh] OR "Housing"[Mesh:noexp]) AND (air pollution[tiab] OR air quality[tiab] OR "Air pollution"[Mesh]) AND "asthma"[Mesh])) OR cadaver[tiab] OR embalm*[tiab] OR garment-workers[tiab] OR textile-workers[tiab] OR wood-workers[tiab] OR woodworkers[tiab]) |
