# Supplementary material for: Exposure to formaldehyde and asthma outcomes: A systematic review, meta-analysis, and economic assessment
Source: PLoS One. 2021 Mar 31;16(3):e0248258. doi: 10.1371/journal.pone.0248258 (PMC8011796; doi:10.1371/journal.pone.0248258)
Supplement: S3 Table — (DOCX) [file pone.0248258.s016.docx]

Supplemental Table 3. Search Terms in Biosis Previews

| **Search** | **Biosis Previews** |
| --- | --- |
| #1 | (asthma OR “reactive airway” OR “reactive airways” OR “airway inflammation” OR wheeze OR wheezes OR wheezing OR dyspnea OR “lung function test” OR spirometry OR “lung function” OR “lung functions” OR “respiratory function” OR respiratory functions OR “pulmonary function” OR “pulmonary functions” OR “bronchus hyperreactivity” OR “bronchial hyperreactivity” OR “bronchial hyper-reactivity” OR “bronchial hypersensitivity” OR “bronchial hyper-sensitivity” OR bronchospasm OR bronchospasm OR “bronchial spasm” OR “bronchial spasms” OR airway resistance OR airway obstruction OR “airway obstruction” OR “airway resistance” OR bronchoconstriction OR “bronchial constriction” OR “bronchial constrictions” OR “respiratory health” OR “reactive airway disease”) AND (50-00-0 OR formaldehyde OR oxomethane OR methanal OR formol OR formalin OR paraformaldehyde OR “medium-density fibreboard” OR “medium-density fiberboard” OR particleboard OR particle-board OR plywood OR wood-based OR composite-wood OR pressed-wood OR “polyurethane foam” OR “polyurethane foam” OR “urea formaldehyde” OR adhesive OR adhesives OR polyurethane foam OR polyurethane* foam OR urea formaldehyde foam OR adhesives OR ((trailer* AND hous*) OR (travel trailer* OR manufactured home* OR mobile home* OR manufactured hous* OR modular home* OR “temporary housing unit” OR “temporary housing units”) OR (“building material” OR housing) AND (“air pollution” OR “air quality”) AND (asthma))) |
