# Supplementary material for: Exposure to formaldehyde and asthma outcomes: A systematic review, meta-analysis, and economic assessment
Source: PLoS One. 2021 Mar 31;16(3):e0248258. doi: 10.1371/journal.pone.0248258 (PMC8011796; doi:10.1371/journal.pone.0248258)
Supplement: S4 Table — (DOCX) [file pone.0248258.s017.docx]

Supplemental Table 4. Search Terms in Embase

| **Search** | **Embase** |
| --- | --- |
| #1 | (‘Asthma’/exp OR  asthma:ab,ti OR  ‘reactive airway*’:ab,ti OR  ‘airway inflammation’:ab,ti OR  'wheezing'/exp OR  wheez*:ab,ti OR  dyspnea:ab,ti OR  'lung function test'/exp OR  spirometry:ab,ti OR  ‘lung function’:ab,ti OR  ‘lung functions’:ab,ti OR  ‘respiratory function’:ab,ti OR  ‘respiratory functions’:ab,ti OR  ‘pulmonary function’:ab,ti OR  ‘pulmonary functions’:ab,ti OR  ‘Forced Expiratory Volume’/exp OR  ‘Peak Expiratory Flow’/exp OR  FEV1:ab,ti OR  PEFR:ab,ti OR  PEF:ab,ti OR  ‘peak expiratory’:ab,ti OR  ‘forced expiratory volume’:ab,ti OR  ‘forced expiratory flow’:ab,ti OR  'bronchus hyperreactivity'/exp OR  ‘bronchial hyperreactivity’:ab,ti OR  ‘bronchial hyper-reactivity’:ab,ti OR  ‘bronchial hypersensitivity’:ab,ti OR  ‘bronchial hyper-sensitivity’:ab,ti OR  'bronchospasm'/exp OR  bronchospasm:ab,ti OR  ‘bronchial spasm’:ab,ti OR  ‘bronchial spasms’:ab,ti OR  'airway resistance'/exp OR  'airway obstruction'/exp OR  ‘airway obstruction’:ab,ti OR  ‘airway resistance’:ab,ti OR  'bronchoconstriction'/exp OR  ‘bronchial constriction’:ab,ti OR  ‘bronchial constrictions’:ab,ti OR  ‘respiratory health’:ab,ti OR  ‘reactive airway disease’:ab,ti) |
| #2 | 'formaldehyde'/exp OR  'formaldehyde' OR  '50-00-0':rn OR  formaldehyde:ab,ti OR  oxomethane:ab,ti OR  methanal:ab,ti OR  formol:ab,ti OR  formalin:ab,ti OR  paraformaldehyde:ab,ti OR  'medium-density fibreboard':ab,ti OR  'medium-density fiberboard':ab,ti OR  particleboard:ab,ti OR  'particle-board':ab,ti OR  plywood:ab,ti OR  'wood-based':ab,ti OR  'composite-wood':ab,ti OR  'pressed-wood':ab,ti OR  ‘varnish*’:ab,ti OR ‘laminate’:ab,ti OR  ‘Floorcoverings’:ab,ti OR flooring:ab,ti OR  'polyurethane foam'/exp OR  'adhesive agent'/exp OR  'polyurethane foam':ab,ti OR  'polyurethan foam'/exp OR  'urea formaldehyde foam':ab,ti OR  adhesives:ab,ti OR  salon:ab,ti OR hair-straighten*:ab,ti OR hair-smooth*:ab,ti OR  (trailer*:ab,ti AND (housing:ab,ti OR 'housing'/exp OR housing)) OR  'travel trailer*':ab,ti OR  'manufactured home*':ab,ti OR  'mobile home*':ab,ti OR  'manufactured hous*':ab,ti OR  'modular home*':ab,ti OR  'temporary housing unit*':ab,ti OR  ('building material/exp' OR 'housing'/exp OR 'housing' AND ('air pollution':ab,ti OR 'air quality':ab,ti OR 'air pollution'/exp OR 'air pollution') AND ('asthma'/exp OR asthma OR asthma:ab,ti)) OR  cadaver:ab,ti OR embalm*:ab,ti OR garment-workers:ab,ti OR textile-workers:ab,ti OR wood-workers:ab,ti OR woodworkers:ab,ti |
| #3 | #1 AND #2 |
