# Supplementary material for: Exposure to formaldehyde and asthma outcomes: A systematic review, meta-analysis, and economic assessment
Source: PLoS One. 2021 Mar 31;16(3):e0248258. doi: 10.1371/journal.pone.0248258 (PMC8011796; doi:10.1371/journal.pone.0248258)
Supplement: S5 Table — (DOCX) [file pone.0248258.s018.docx]

Supplemental Table 5. Search Terms in Toxline and DART

| **Search** | **Toxline & DART** |
| --- | --- |
| #1 | Formaldehyde and asthma |
