# Supplementary material for: Exposure to formaldehyde and asthma outcomes: A systematic review, meta-analysis, and economic assessment
Source: PLoS One. 2021 Mar 31;16(3):e0248258. doi: 10.1371/journal.pone.0248258 (PMC8011796; doi:10.1371/journal.pone.0248258)
Supplement: S6 Table — (DOCX) [file pone.0248258.s019.docx]

Supplemental Table 6. Toxicological Websites/Databases

| ATSDR Interaction Profiles <http://www.atsdr.cdc.gov/interactionprofiles/index.asp> | EPA Substance Registry System <http://ofmpub.epa.gov/sor_internet/registry/substreg/searchandretrieve/substancesearch/search.do> |
| --- | --- |
| ATSDR Toxicological Profiles <http://www.atsdr.cdc.gov/toxprofiles/index.asp> | Health Canada First Priority List Assessments [http://www.hcsc.gc.ca/hecs sesc/exsd/psl1.htm](http://www.hcsc.gc.ca/hecs%20sesc/exsd/psl1.htm) |
| ACTOR | Health Canada Second Priority List Assessments [http://www.hcsc.gc.ca/hecs sesc/exsd/psl2.htm](http://www.hcsc.gc.ca/hecs%20sesc/exsd/psl2.htm) |
| CalEPA Office of Environmental Health Hazard Assessment <http://www.oehha.ca.gov/risk.html>, <http://oehha.ca.gov/air.html> | Hazardous Substances Data Bank <http://toxnet.nlm.nih.gov/cgi-bin/sis/htmlgen?HSDB> |
| Chem ID <http://chem.sis.nlm.nih.gov/chemidplus/> | IPCS INCHEM <http://www.inchem.org/> |
| EPA Acute Exposure Guideline Levels <http://www.epa.gov/oppt/aegl/chemlist.htm> | NIOSHTIC 2 [http://www2.cdc.gov/nioshtic 2/Nioshtic2.htm](http://www2.cdc.gov/nioshtic%202/Nioshtic2.htm) |
| EPA IRIS internet [www.epa.gov/iris](http://www.epa.gov/iris) | Toxicology Data Network <http://toxnet.nlm.nih.gov/> |
| EPA NEPIS and NSCEP <http://www.epa.gov/nscep/> | RTECS Toxcenter <http://www.cdc.gov/niosh/rtecs/default.html> |
| FIFRA docket: <http://www.regulations.gov> | WHO assessments – CICADS, EHC <http://www.who.int/ipcs/assessment/en/> |
| EPA Science Inventory <http://cfpub.epa.gov/si/> | USEPA Health and Environmental Studies Online <http://hero.epa.gov/> |
