# Supplementary material for: Exposure to formaldehyde and asthma outcomes: A systematic review, meta-analysis, and economic assessment
Source: PLoS One. 2021 Mar 31;16(3):e0248258. doi: 10.1371/journal.pone.0248258 (PMC8011796; doi:10.1371/journal.pone.0248258)
Supplement: S7 Table — (DOCX) [file pone.0248258.s020.docx]

Supplemental Table 7. Grey Literature Websites/Databases

| Google: <http://www.google.com> | Open Grey: <http://www.opengrey.eu/> |
| --- | --- |
| Google Scholar: <http://scholar.google.com/> | Proceedings First (Covers every published congress, symposium, conference, exposition, workshop and meeting received by The British Library Document Supply Centre) |
| Database of federally-funded scientific research: Science.gov | Papers First (Access to individual papers presented at conferences worldwide) |
| ScienceResearch.com (Science federated search engine by Deep Web Technologies): [http://scienceresearch.com/](http://scienceresearch.com/scienceresearch/search.html) | ProQuest Dissertations and Theses (International repository of graduate dissertations and theses) |
| Oaister database (an open-source repository of difficult-to-access, academically-oriented digital resources): <http://www.oclc.org/oaister> |  |
