# Supplementary material for: Exposure to formaldehyde and asthma outcomes: A systematic review, meta-analysis, and economic assessment
Source: PLoS One. 2021 Mar 31;16(3):e0248258. doi: 10.1371/journal.pone.0248258 (PMC8011796; doi:10.1371/journal.pone.0248258)
Supplement: S8 Table — (DOCX) [file pone.0248258.s021.docx]

Supplemental Materials, Table 8. Characteristics of Akbar Khanzadeh et al. 1994

| Bias domain | Authors’ judgment | Support for judgment |
| --- | --- | --- |
| Source population representation | Probably high | Subjects worked with human cadavers preserved in formaldehyde in the gross anatomy laboratory at the medical college. Control subjects were students and instructors recruited from the same institution. Although it appears that all formaldehyde exposed subjects were approached, there are few details on how the controls were recruited. There were a total of 54 subjects who volunteered (36 exposed and 18 unexposed). Smokers were excluded from the study (2 study and 1 control subject). Five of 18 controls dropped out for unspecified reasons. There is no comparison between characteristics for drop-outs and controls that remained in the study. The authors note that males and females in the study group were younger than males and females in the control group, but were otherwise comparable. |
| Blinding | Probably high | There is no evidence of blinding. In fact, subjects were introduced to the study protocol and objectives of the study before the start of the study. Pulmonary tests were performed by exposed subjects who were aware of their exposure status. |
| Outcome assessment | Low | Subjects used a spirometer to measure lung function before and after their shift. The specifications for the measurements were within criteria set forth by the American Thoracic Society. Accuracy of the spirometry was checked with a calibrator syringe with a volume of 3L before and after the daily measurement. One researcher repeated pre- and post-shift pulmonary function tests every day to ensure consistency of the results. A minimum of 4 technically acceptable forced expiratory maneuvers were obtained. Presence of acute symptoms during or after the laboratory were self-reported. |
| Confounding | Low | All subjects were non-smokers. Height and weight was similar between the two groups. The control subjects were older than the exposed subjects, both among females and males, There were slight differences in the ethnicity of the subjects, although the majority of them were white in both groups. However, subjects were their own control with pre and post shift measurements being taken. |
| Incomplete outcome data | Low | There is no apparent missing outcome data. |
| Exposure assessment | Probably low | Personal air sampling devices were attached to study subjects during the gross anatomy laboratory class and for one control subject on 6 test days. One subject refused to wear the sampler and another accidentally turned off the sampler. Two time-weighted average area samples were also taken on days 4 and 9. Air samples were collected and analyzed using OSHA Method 52, with an estimated detection limit of 1 ug formaldehyde per sample. |
| Selective outcome reporting | Low | All of the published manuscript's outcomes outlined in the methods, abstract, and/or introduction section that are of interest in the review have been reported in the specified way. |
| Conflict of interest | Probably low | Funding is not reported. Authors are affiliated with an academic institution and there is no reason to believe that a conflict of interest exists. |
| Other sources of bias | Probably low | The authors do not specify whether the paired t-test used or tested the homogeneity of variances assumption. |
