# Supplementary material for: Exposure to formaldehyde and asthma outcomes: A systematic review, meta-analysis, and economic assessment
Source: PLoS One. 2021 Mar 31;16(3):e0248258. doi: 10.1371/journal.pone.0248258 (PMC8011796; doi:10.1371/journal.pone.0248258)
Supplement: S9 Table — (DOCX) [file pone.0248258.s022.docx]

Supplemental Materials, Table 9. Characteristics of Akbar Khanzadeh et al. 1997

| Bias domain | Authors’ judgment | Support for judgment |
| --- | --- | --- |
| Source population representation | Probably low | Group consisted of 50 first year medical students (25 men and 25 women) recruited from a medical school and a control group of 36 second year physiotherapy students (8 men and 28 women) attending the same university. It appears that subjects were matched because it was noted that there were fewer males in the control because there were a limited number of matched control males. However, the methods for recruitment and matching were not reported. A table of characteristics was provided for exposed and non-exposed individuals. |
| Blinding | Probably high | There is no evidence of blinding. However, the subjects would have had knowledge of the exposure status and they all used the spirometer. |
| Outcome assessment | Low | Spirometric tests were used. Three separate sets of tests were performed. One investigator administered the test according to the guidelines prescribed by the American Thoracic Society. The portable spirometer was calibrated on each study day. Biological calibrations were also performed weekly by one researcher to ensure consistent results. Four efforts were attempted and the best test results recorded. This was done before class and at 1 and 3 hours. The barometric pressure and ambient temperature were recorded and test variables were corrected accordingly. |
| Confounding | Low | All participants were non-smoking individuals (Tier I), and were similar in age, height, weight, gender ratios in the study groups, and baseline respiratory function (Tier II). Subjects were tested before and after so were acting as their own control. |
| Incomplete outcome data | Low | There is no apparent missing data. |
| Exposure assessment | Probably low | Personal and area formaldehyde samples were collected using a constant flow pump and analyzed with analytical grade chemicals by spectrophotometer according to NIOSH method 3500. LODs and QA/QC details are not provided. Controls were in a class in a room in a separate building. |
| Selective outcome reporting | Low | All of the study’s pre-specified outcomes outlined in the published manuscript’s methods, abstract, and/or introduction section that are of interest in the review have been reported in the pre-specified way. |
| Conflict of interest | Probably low | Funding source was not reported, but authors were affiliated with the Medical College of Ohio, Department of Occupational Health and are unlikely to have a conflict of interest. |
| Other sources of bias | Low | The study appears to be free of other sources of bias. |
