# Supplementary material for: Exposure to formaldehyde and asthma outcomes: A systematic review, meta-analysis, and economic assessment
Source: PLoS One. 2021 Mar 31;16(3):e0248258. doi: 10.1371/journal.pone.0248258 (PMC8011796; doi:10.1371/journal.pone.0248258)
Supplement: S10 Table — (DOCX) [file pone.0248258.s023.docx]

Supplemental Materials, Table 10. Characteristics of Annesi Maesano et al. 2012

| Bias domain | Authors’ judgment | Support for judgment |
| --- | --- | --- |
| Source population representation | Probably low | The study surveyed 401 randomly chosen classrooms in 108 primary schools attended by 6590 children in the French 5 City study. Study participants were children aged 9-10 years who were invited to take part in a health survey according to a standardized protocol. The study report at 81% response rate and a 69% participation rate. Characteristics of subjects who did not complete the entire survey protocol are not provided. |
| Blinding | Probably low | There is no evidence of blinding, but times of school visits for air quality assessment and medical examination of the children were randomly chosen. |
| Outcome assessment | Probably low | EIA and allergic sensitization appear to be assessed and defined consistently across all participants (medical exam) using valid and reliable methods, and some QA/QC is described. Health outcomes other than EIA and allergic sensitization were reported by parents using the ISAAC questionnaire. There is no evidence of objective validation of parents' reports and this could lead to misclassification of asthma and rhinitis. Rating is probably low because allergic sensitization is not a standard way to confirm diagnosis or symptoms, although using parental report is adequate for probably low risk of bias. |
| Confounding | Low | All Tier 1 and some Tier 2 potential confounders were accounted for and included age, gender, passive smoking, paternal or maternal history of asthma and allergic diseases, dampness, gas appliance, ethnicity and socioeconomic status (SES). |
| Incomplete outcome data | Low | The study was restricted to only children with fully reported data. There is no comparison of the characteristics of subjects who did not complete the entire survey protocol. However, the authors performed a sensitivity analysis on a restricted sample of children with concurrent/ same week medical examination and air quality assessments. Children of the restricted sample did not differ significantly from the others in terms of age, sex, weight, height and baseline peak flow, but they had more educated fathers. |
| Exposure assessment | Low | Concentrations of formaldehyde (and other pollutants) were measured with passive diffusion samplers. Measurements were made during the week when most of the children using the classroom underwent medical examinations using standardized methodology. Methods, reliability and reproducibility of air pollution assessment have been presented elsewhere, and they appear to be robust. |
| Selective outcome reporting | Low | All of the published manuscript's outcomes outlined in the methods, abstract, and/or introduction section that are of interest in the review have been reported in the specified way. |
| Conflict of interest | Low | Funding source is limited to government offices and authors made a claim of no competing interests. |
| Other sources of bias | Low | There is no evidence other potential sources of bias. |
