# Supplementary material for: Exposure to formaldehyde and asthma outcomes: A systematic review, meta-analysis, and economic assessment
Source: PLoS One. 2021 Mar 31;16(3):e0248258. doi: 10.1371/journal.pone.0248258 (PMC8011796; doi:10.1371/journal.pone.0248258)
Supplement: S11 Table — (DOCX) [file pone.0248258.s024.docx]

Supplemental Materials, Table 11. Characteristics of Billionnet et al. 2011

| Bias domain | Authors’ judgment | Support for judgment |
| --- | --- | --- |
| Source population representation | Probably low | The study is a cross-sectional survey with a three-stage random selection procedure to obtain a population-based representative sample. Details are provided on the selection procedure and rate of participation. Authors note that the acceptance rate to participate was low (19.5%) and consider it a study limitation. |
| Blinding | Probably low | There is insufficient information on blinding. Participants self-reported outcomes and potentially had knowledge of possible exposure within their own home. However, it is unlikely they were aware of the exact levels of formaldehyde, or that formaldehyde was the VOC of concern in this cross-sectional survey. |
| Outcome assessment | Probably low | A standardized self-administered questionnaire was completed by individuals aged 15 years or older living in the dwelling. The questionnaire was derived from the European Community Respiratory Health Survey(ECRHS)(http://www.ecrhs.org/) and the International Study of Asthma and Allergies in Childhood (ISAAC). Asthma in the past year was defined as suggested in the ECRHS (Ellison-Loschmann et al., 2007), on the basis of one of the following criteria: (i) having an asthma attack in the last 12 months;(ii) having been woken by an attack of shortness of breath in the last 12 months; and (iii) currently using asthma medicine. However diagnoses were not confirmed by a physician. |
| Confounding | Low | The study accounted for all Tier I confounders (smoking status, age, and education). Several other tier II confounders, such as sex, humidity, time of survey, presence of pets, presence of mold, proportion of time spent at home and other outdoor sources of pollution were also included in the models. |
| Incomplete outcome data | Low | Approximately 10% missing data for asthma (905/1012) and rhinitis (916/1012). The authors noted that the characteristics of the individuals not included in the model due to missing values did not differ from the characteristics of included individuals. They also note that dwellings not included in the model due to missing values were not different from included dwellings in pollutant and comfort parameter concentrations. |
| Exposure assessment | Probably low | Exposures were measured for one week in the bedroom of the reference person of the household using area. The measurement was conducted for a single week and may be a poor surrogate for past year exposure, and concentrations in the dwellings did not take into account the exposure to outdoor air. Exposure of the bedroom of one person in the household was assumed representative the household exposure of all inhabitants. The detection limit (0.6 ug/m3) and quantification limit (1.1 ug/m3) for formaldehyde were presented, and no samples were LOD or LOQ. The maximum values of 8 hour moving averages were used for analysis. In addition, weekly diaries were used to record activities and time spent at home. |
| Selective outcome reporting | Low | Results were presented for all outcomes discussed in the abstract and methods. |
| Conflict of interest | Low | Funding source is limited to government agencies and authors state that there is not conflict of interest. |
| Other sources of bias | Low | The authors note that atopy was not considered as a risk factor, which could lead to an overestimation of the effects of VOCs. However, they expect this potential risk of bias to be low because the sample was randomly selected, so an equal distribution of atopic individuals is expected. |
