# Supplementary material for: Exposure to formaldehyde and asthma outcomes: A systematic review, meta-analysis, and economic assessment
Source: PLoS One. 2021 Mar 31;16(3):e0248258. doi: 10.1371/journal.pone.0248258 (PMC8011796; doi:10.1371/journal.pone.0248258)
Supplement: S12 Table — (DOCX) [file pone.0248258.s025.docx]

Supplemental Materials, Table 12. Characteristics of Burge et al. 1984

| Bias domain | Authors’ judgment | Support for judgment |
| --- | --- | --- |
| Source population representation | Probably high | Subjects were referred after presenting symptoms, and there were only 5 with asthma or asthma symptoms thought to be due to exposure to formaldehyde; there could be additional individuals with symptoms that did not report them so may not be representative of population. No additional information is provided about recruitment or inclusion/exclusion criteria and small sample size (15 subjects). |
| Blinding | High | There is no discussion of blinding. Bronchial provocation testing with formaldehyde was not blinded and investigators (and possibly subjects) likely knew which participants were being exposed to formaldehyde when performing the FEV tests after exposure. |
| Outcome assessment | Low | Outcomes were measured by bronchial reactivity and histamine reactivity. Bronchial provocation tests were carried out in a 6 m3 chamber without air extraction during the test using solutions of 0 1%, 1%, 10%, 20%, and 25% of saturated formaldehyde painted onto a paper surface. Non-specific bronchial reactivity to histamine was measured on the day before the formaldehyde bronchial provocation test in 14/15 patients. Doubling concentrations of histamine from 0.25 to 32 mg/ml were inhaled for 30 seconds with a Wright's nebuliser and rebreathing bag, following a previously described method. Low ROB because objective measures were used (bronchial provocation tests) to determine outcomes. |
| Confounding | Probably low | Authors measured smoking (Tier I), but did not mention SES. Some Tier II confounders were also measured including age, sex, and previous history of asthma or rhinitis. |
| Incomplete outcome data | Probably low | There was 10% missing data (1/15) |
| Exposure assessment | Probably low | Bronchial provocation tests were carried out in a 6 m3 chamber. Formaldehyde solutions of different dilutions (0.1, 1, 10, 20, and 25%) were painted on to a paper surface continuously during exposure. Atmospheric concentrations of formaldehyde were measured 11 times during formaldehyde exposure. The samples were taken in the breathing zone of the exposed worker. Formaldehyde was assayed with nitroblue tetrazolium. No QA/QC methods were described. |
| Selective outcome reporting | Low | Results are reported for all outcomes specified in the abstract and methods. |
| Conflict of interest | Probably low | Authors are medical researchers. No information is provided on source of funding for the study. There is no reason to believe that a conflict of interest exists. |
| Other sources of bias | Probably low | Workers from various industries were referred to study after presenting with symptoms of occupational asthma. It seems that this study design could capture highly affected individuals, as all cases were referred for having symptoms and study is not focused on a particular plant or group of workers, etc. |
