# Supplementary material for: Exposure to formaldehyde and asthma outcomes: A systematic review, meta-analysis, and economic assessment
Source: PLoS One. 2021 Mar 31;16(3):e0248258. doi: 10.1371/journal.pone.0248258 (PMC8011796; doi:10.1371/journal.pone.0248258)
Supplement: S13 Table — (DOCX) [file pone.0248258.s026.docx]

Supplemental Materials, Table 13. Characteristics of Chatzidiakou et al. 2014

| Bias domain | Authors’ judgment | Support for judgment |
| --- | --- | --- |
| Source population representation | Probably low | Two schools (one urban, one rural) in Greater London area were recruited from a number of consenting school authorities. The schools were contacted initially by phone and were offered a small financial recompense for their involvement. In each school three representative classrooms in terms of geometrical characteristics and occupancy schedule and one outdoor site in the school premises were selected. Classrooms accommodating older children were preferred, as their responses to questionnaires were considered likely to be more accurate. Out of the 176 children attending the classrooms, 151 (86%) returned a completed questionnaire. |
| Blinding | Probably low | There is no information on blinding. Data collection incorporated an on-site building survey completed by school personnel, energy performance data, measurements of air pollutants, and a standardized questionnaire survey on health symptoms distributed to school children attending selected classrooms. Since the schools received financial compensation for participating in the study, it is likely that school personnel was aware of study aim. Study was rated probably low because questionnaire was completed by student participants who likely did not know their exposure status. |
| Outcome assessment | Probably low | A standardized questionnaire survey was used for collecting information from the school children on asthma and asthmatic symptoms. Versions of the questionnaire have been used in epidemiological studies among students in schools, and the authors state that their validity is similar to medical interviews. However, there is no information that asthma was doctor diagnosed. |
| Confounding | Probably low | Potential confounders considered included personal (gender, age, exposure to tobacco smoke, satisfaction with the school environment, and stress levels; collected via standardized questionnaire, with validity similar to medical interviews) and psychosocial factors. There is no information on which psychosocial factors were considered. Parental SES was not considered. The study adjusted for environmental tobacco smoke (Tier I), gender, and indoor climate variables (Tier II). |
| Incomplete outcome data | Low | Data was complete for the 151 subjects included in the study. |
| Exposure assessment | Low | Formaldehyde was sampled (indoor and outdoors) with cartridges impregnated with 2,4-dinitrophenylhydrazine and analyzed with high performance liquid chromatography. Cartridges were carried onsite in cooling bags and were exposed immediately before sampling. After the 5-day exposure cartridges were sealed in air-tight glass vials and sent for analysis to an external laboratory. Prior to fieldwork, all monitoring equipment was calibrated by the manufacturers. One duplicate and one field blank were used per school. Duplicates showed a very high reproducibility of the method and blanks were below instrumental noise. |
| Selective outcome reporting | Low | Results are reported for all outcomes specified in the abstract and methods. |
| Conflict of interest | Low | The authors have academic affiliations, and the authors declared no conflict of interest. Funding source was declared as " no specific grant from any funding agency in the public, commercial, or not-for-profit sectors." |
| Other sources of bias | Low | No other potential sources of bias have been identified. |
