# Supplementary material for: Exposure to formaldehyde and asthma outcomes: A systematic review, meta-analysis, and economic assessment
Source: PLoS One. 2021 Mar 31;16(3):e0248258. doi: 10.1371/journal.pone.0248258 (PMC8011796; doi:10.1371/journal.pone.0248258)
Supplement: S14 Table — (DOCX) [file pone.0248258.s027.docx]

Supplemental Materials, Table 14. Characteristics of Choi et al. 2009

| Bias domain | Authors’ judgment | Support for judgment |
| --- | --- | --- |
| Source population representation | Probably low | The study consists of patients with atopy and controls in the same age group identified through the same hospital. Characteristics of houses provided in the study are presented. Details of inclusion/exclusion criteria are not presented. |
| Blinding | Probably low | There is no evidence of blinding. However, outcomes were assessed prior to the exposure measurement and likely did not influence the measurements. Exposure was performed using active sampling pumps and likely not to be biased by investigator's knowledge of outcome for the participants. |
| Outcome assessment | Probably low | Specific details of the outcome assessment are not provided. Outcome is based on whether an individual is an atopy patient (either atopic dermatitis or allergic asthma) or a non-patient from the recruiting hospital. Diagnosis of atopy was confirmed by medical history, medical skin prick tests and IgE assays. |
| Confounding | High | Multivariate analyses adjusting for potential confounders (house characteristics) were only for investigations of factors affecting the indoor air concentrations of formaldehyde in atopy patients' households. No Tier I or II confounders considered. Subject specific characteristics were not considered. |
| Incomplete outcome data | Low | There are no missing data. |
| Exposure assessment | Probably high | The exposure assessment used active sampling pumps (EPA method TO-11A). Samples were conducted in the living room of subjects (reported as well away from known sources). Sampling times were not reported, and authors noted only VOCs with detection probabilities greater than 50% and a concentration greater than 1ug/m3 were selected for comparisons. |
| Selective outcome reporting | Low | Results were presented for all outcomes mentioned in the abstract and methods. |
| Conflict of interest | Low | The study was funded by the government and all authors were academics. |
| Other sources of bias | Probably low | The authors noted a relatively small sample size as a limitation. |
