# Supplementary material for: Exposure to formaldehyde and asthma outcomes: A systematic review, meta-analysis, and economic assessment
Source: PLoS One. 2021 Mar 31;16(3):e0248258. doi: 10.1371/journal.pone.0248258 (PMC8011796; doi:10.1371/journal.pone.0248258)
Supplement: S15 Table — (DOCX) [file pone.0248258.s028.docx]

Supplemental Materials, Table 15. Characteristics of Dannemiller et al. 2013

| Bias domain | Authors’ judgment | Support for judgment |
| --- | --- | --- |
| Source population representation | Probably high | Participants were asthmatic children and adults from 70 primarily low-income homes, part of the Boston Allergen Sampling Study, for which the authors noted the manuscript was in preparation. There was no information provided on the selection procedure and the study population. Brief demographic characteristics of the children included in the analysis (n=67) were presented. |
| Blinding | Probably low | There is no evidence of blinding. The outcome (asthma status) was assessed with the validated Asthma Control Test (ACT) for ages 12 and up was administered to the asthmatic resident. If the child was under 12, the parent or caregiver was asked to answer for the child. It is unlikely that participants were aware of their exposure level, or that either exposure measurements or outcome assessment were influenced. |
| Outcome assessment | Probably low | Outcomes were reported using the validated Asthma Control Test (ACT); parents or caregivers provided answers for children under the age of 12. |
| Confounding | Low | Participants were primarily low income. Data on type of housing, age of the building home ownership was also considered. Resident smoking habits were recorded. Authors addressed a number of other covariates, including age, gender, and race. Additional demographic information for children is provided in the supplemental material. |
| Incomplete outcome data | Low | Formaldehyde exposure assessments were not completed in 3 homes which were removed from the analysis (final n = 67). Analyses considering the health effects of formaldehyde exposure were restricted to children 18 years (n= 37). No other missing data are reported. |
| Exposure assessment | Probably low | Exposure was assessed using Kitagawa 710 formaldehyde detector tubes. Formaldehyde readings and pump performance were validated to ensure that the alternate pump did not affect the results. Formaldehyde measurement was checked in a laboratory setting by heating a permeation tube. Pump performance was also validated by measuring the flow rate through the permeation tubes from four different pumps and from the same pump over time. The pump flow rate remained constant over the time period required for sampling. However, samples were taken for only a total of 30 minutes. The exposure assessment method has a reported standard deviation of 10% and a detection limit of 5 ppb. |
| Selective outcome reporting | Low | Results are presented for all outcomes outlined in the abstract and methods. The analysis of health effects was limited to children under the age of 18 for whom ACT data were available because they represented the major age group of the study, and no health analyses were conducted for adults. |
| Conflict of interest | Low | Funding was provided by government bodies and an academic grant. All authors were affiliated with academic institutions and there is no reason to believe there is a conflict of interest. |
| Other sources of bias | Low | No other threats to internal validity were identified. |
