# Supplementary material for: Exposure to formaldehyde and asthma outcomes: A systematic review, meta-analysis, and economic assessment
Source: PLoS One. 2021 Mar 31;16(3):e0248258. doi: 10.1371/journal.pone.0248258 (PMC8011796; doi:10.1371/journal.pone.0248258)
Supplement: S16 Table — (DOCX) [file pone.0248258.s029.docx]

Supplemental Materials, Table 16. Characteristics of De Vos et al. 2009

| Bias domain | Authors’ judgment | Support for judgment |
| --- | --- | --- |
| Source population representation | Probably low | 67 study participants were selected from 1000 currently active career firefighters in Western Australia. Individuals with unstable asthma, current acute or chronic respiratory illness, or any other chronic or severe illnesses were excluded from the study. Participant characteristics were described. The researchers determined the random allocation of the intervention after completion of the participant register, to ensured that the decision to participate in the study was made regardless of which intervention group the participant would be allocated. The authors note that there may have been selection bias towards healthier individuals or individuals concerned with health effects, as they were more likely to choose to participate in the study. Adjustment techniques to account for potential selection bias were not described. |
| Blinding | Low | This was a double-blind randomized study. Study authors tried to minimize "unblinding" by ensuring the different filters looked as similar as possible. |
| Outcome assessment | Low | Outcomes were assessed by respiratory symptom questionnaire, spirometry, and oximetry. Spirometry was performed by a single trained individual according to the recommendations for maneuver performance provided by the American Thoracic Society [American Thoracic Society, 1995]. The arterial oxygen saturation of hemoglobin was measured in percentages (%) using a Datex-Ohmeda TuffSatTM pulse oximeter. |
| Confounding | Low | Participants with uncontrolled asthma and respiratory conditions were excluded. Authors measured some Tier I variables such as smoking, but did not measure SES. SES may have less influence in this study, as all participants have the same occupation. They measured some Tier II variables including sex, age, respiratory symptoms, and number of years working as a firefighter. Participants were randomized to different interventions. |
| Incomplete outcome data | Low | Results are complete for the 67 study subjects. |
| Exposure assessment | Low | Authors used a validated method to conduct personal air sampling for formaldehyde inside respirators (Johnston et al. 1992). The sampling lasted for 120 min. Formaldehyde analysis was conducted by HPLC by NIOSH method 2016. |
| Selective outcome reporting | Low | Results are reported for all outcomes specified in the methods. |
| Conflict of interest | Low | Authors were from academic and medical institutions. Funding was provided through the Bushfire Cooperative Research Centre (which was funded by a grant from the Australian government). |
| Other sources of bias | High | The study randomly allocates firefighters to various groups to test the effectiveness of various protective filters. The authors specifically exclude individuals with "unstable asthma, current acute or chronic respiratory illness, or any other chronic or severe illnesses" and note that this could lead to selection bias, as it favors healthier individuals. Also, the authors did not specify whether the paired t-test assumed equal or unequal variance. |
