# Supplementary material for: Exposure to formaldehyde and asthma outcomes: A systematic review, meta-analysis, and economic assessment
Source: PLoS One. 2021 Mar 31;16(3):e0248258. doi: 10.1371/journal.pone.0248258 (PMC8011796; doi:10.1371/journal.pone.0248258)
Supplement: S17 Table — (DOCX) [file pone.0248258.s030.docx]

Supplemental Materials, Table 17. Characteristics of Delfino et al. 2003

| Bias domain | Authors’ judgment | Support for judgment |
| --- | --- | --- |
| Source population representation | Probably low | The authors received referrals from local schools in Los Angeles to identify study participants. While the authors stated lengthy inclusion criteria, they admit to relaxing some requirements (mostly age), as to increase recruitment for several participants, however, this is not likely to pose bias. This panel study consisted of 24 Hispanic children, primarily male, ages 10-16. |
| Blinding | Probably low | Subjects and their parents were blinded to the substances being monitored (this is stated in the Delfino 2002 report). Trained research assistants recorded baseline and end-of-study spirometry. Authors did not report whether the research assistants, data abstractors, or those conducting the statistical analyses were blinded to exposure status. |
| Outcome assessment | Low | Participants recorded the severity of their asthma symptoms daily using a six-level ordinal scale. They also recorded the daily number of beta-agonist inhaler puffs and use of preventitive asthma medications. And three PEF maneuvers in the morning and in the evening; the highest was retained in analyses. Authors excluded 17.8% of PEF that did not meet the reproducibility criterion of ≤ 10% difference between the highest and second highest PEF. Trained research assistants recorded baseline and end-of-study spirometry. Sensitivity analyses were not reported. Rating of low risk of bias was given because objective measures (pulmonary function tests) were used to determine outcomes. |
| Confounding | Low | The authors used study exclusion criteria to limit confounders such as active and passive smoking, and selected non-working Hispanic children. Confounding by weekend versus weekday, maximum temperature, and respiratory infections was also accounted for. In the 2002 report, it is stated that SES was considered, and all families in the study had low SES. |
| Incomplete outcome data | Low | There is very little missing data. Investigators recruited 26 children. Two subjects were excluded from the study because they did not complete their diaries. Evidence of falsified PEF data led to exclusion of two additional subjects. Respiratory infection reports for another two subjects were invalid. This left 22 subjects for univariate regression models and 20 subjects for models including the respiratory infection variable. |
| Exposure assessment | Low | Outdoor 24-hr air samples for noncarbonyl VOCs were collected in canisters with the XonTech 910A (XonTech Inc., Van Nuys, CA) and analyzed using U.S. EPA TO-14 methodology (SCAQMD 2000). The Outdoor 24-hr air samples for carbonyls were collected with the XonTech 920 (XonTech Inc.) and analyzed using U.S. EPA method TO-11 (SCAQMD 2000). QA/QC data are provided in the 2002 report. |
| Selective outcome reporting | Low | All of the study’s pre-specified (primary and secondary) outcomes outlined in the published manuscript’s methods, abstract, and/or introduction section that are of interest in the review have been reported in the pre-specified way. |
| Conflict of interest | Low | All authors are affiliated with academic institutions and the work was funded by government with additional contracts from non-profit organizations. |
| Other sources of bias | Low | The study appears to be free of other sources of bias. |
