# Supplementary material for: Exposure to formaldehyde and asthma outcomes: A systematic review, meta-analysis, and economic assessment
Source: PLoS One. 2021 Mar 31;16(3):e0248258. doi: 10.1371/journal.pone.0248258 (PMC8011796; doi:10.1371/journal.pone.0248258)
Supplement: S18 Table — (DOCX) [file pone.0248258.s031.docx]

Supplemental Materials, Table 18. Characteristics of Dumas et al. 2017

| Bias domain | Authors’ judgment | Support for judgment |
| --- | --- | --- |
| Source population representation | Low | Detailed information is provided in Figure 1 flow chart. The source population is an ongoing population-based prospective study—all nurses with asthma in 2011 were asked to participated and they had an 80% response rate. Nonrespondents were significantly younger and were more often current smokers, obese and non-White than respondents. |
| Blinding | Low | Assessment of exposure and outcome measures were self-reported so there was no blinding of the study investigator; outcomes were assessed using the same measurement or estimation metrics across the exposure groups and exposure was assessed using the same measurement or estimation metrics across the diagnostic or outcome groups. |
| Outcome assessment | Low | Asthma was well-characterized in this population of nurses—a prior validation study confirmed the nurses’ self-reports of doctor-diagnosed asthma. Asthma control was defined using a standardized and validated definition, integrating the main domains of asthma control symptoms, use of rescue therapy, sleep interference and activity limitations. |
| Confounding | Low | Authors adjusted for all Tier I confounders except for SES, but all the participants were currently working nurses, which could be a surrogate for SES. Analyses were adjusted for age, smoking, status, body mass index, race (White versus Other), and ethnicity (Hispanic versus Other). |
| Incomplete outcome data | Low | Participants with missing data for ACT score questions did not differ from included participants for age, smoking status, body mass index, race, ethnicity or self-reported use of disinfectants. |
| Exposure assessment | Low | Job task exposure was used, which had a greater capacity to classify exposures than nursing job titles alone, since nurses with the same job title could potentially perform different tasks. Authors conducted sensitivity analysis, which supported the exposure assessment results. |
| Selective outcome reporting | Low | Results reported for all outcomes specified in the abstract and methods. |
| Conflict of interest | Low | The authors have academic affiliations and declare that there is no conflict of interest. |
| Other sources of bias | Low | No other threats to internal validity were identified. |
