# Supplementary material for: Exposure to formaldehyde and asthma outcomes: A systematic review, meta-analysis, and economic assessment
Source: PLoS One. 2021 Mar 31;16(3):e0248258. doi: 10.1371/journal.pone.0248258 (PMC8011796; doi:10.1371/journal.pone.0248258)
Supplement: S19 Table — (DOCX) [file pone.0248258.s032.docx]

Supplemental Materials, Table 19. Characteristics of Elshaer et al. 2017

| Bias domain | Authors’ judgment | Support for judgment |
| --- | --- | --- |
| Source population representation | Probably high | No numbers of the source population of staff and unexposed were given. The paper states that tat the time of the surveys a few number of staff were available; seems to imply the same may not have been representative of all the staff. There is a high concern about possible health impacts of exposure among the anatomy department staff, so there were likely differential selection bias exposed into the study compared to unexposed. |
| Blinding | Probably low | The researchers knew who was “exposed” based on their job, but the participants were the ones to conduct the outcome assessment. |
| Outcome assessment | Probably high | All outcomes were self-reported with no mention of whether equally valid among exposed and unexposed |
| Confounding | High | No adjustment for any confounders |
| Incomplete outcome data | Low | No incomplete outcome data |
| Exposure assessment | Probably low | Exposure determination based on the department of job site; appears to be a valid assumption that formaldehyde is not used in one department and that there is exposure in the anatomy department even if it was not measured. Working in the anatomy department appears to be a reliable measure, as formalin is used extensively. |
| Selective outcome reporting | Low | All asthma related outcomes reported among exposed and unexposed |
| Conflict of interest | Low | The authors have academic affiliations and declare that there is no conflict of interest. |
| Other sources of bias | Low | No other threats to internal validity were identified. |
