# Supplementary material for: Exposure to formaldehyde and asthma outcomes: A systematic review, meta-analysis, and economic assessment
Source: PLoS One. 2021 Mar 31;16(3):e0248258. doi: 10.1371/journal.pone.0248258 (PMC8011796; doi:10.1371/journal.pone.0248258)
Supplement: S20 Table — (DOCX) [file pone.0248258.s033.docx]

Supplemental Materials, Table 20. Characteristics of Ezratty et al. 2007

| Bias domain | Authors’ judgment | Support for judgment |
| --- | --- | --- |
| Source population representation | Probably high | Twelve subjects ages 18-44 who were previously diagnosed with intermittent asthma and allergy to pollen were included. These inclusion criteria were confirmed by skin standardized skin prick, blood IgE, and pulmonary function tests prior to inclusion. Limited details on the recruitment protocol were included. No information was provided on how the subjects were recruited and demographic information was limited. |
| Blinding | Low | This was a double-blind crossover study. |
| Outcome assessment | Low | Outcomes of lung function and peak expiratory flow were measured with standard spirometry methods in a controlled laboratory setting. Specifically, lung function was measured with a spirometer according to the European Community Respiratory Health Survey specifications immediately before, during, and 8 hours after the end of an allergen challenge. Forced expiratory volume in 1 sec (FEV1) and PEF were measured with a portable combined spirometer every 15 min during the exposure to formaldehyde or air-only in the chamber and every hour until the methacholine test. Respiratory symptoms were self-reported after 0, 15, 30, 45, and 60 minutes of exposure to formaldehyde or air-only. FEV1 and PEF were self-measured using the portable combined spirometer two times per day for two weeks following exposure. |
| Confounding | Low | All subjects were nonsmokers (Tier I), and the age, sex, and asthma duration (Tier II) of all the subjects were presented. SES was not addressed. Authors noted none of the twelve subjects were receiving anti-inflammatory therapy or other current treatments, and the study was performed outside of grass pollen season. Participants were randomized to different interventions. |
| Incomplete outcome data | Low | No missing outcome data reported. |
| Exposure assessment | Probably low | Formaldehyde concentrations were kept constant using standard methods in an experimental laboratory setting. A continuous 1 hour injection of formaldehyde solution was injected into the test chamber and the concentration was monitored continuously with semiconductor gas sensor technology. No QA/QC methods were described. |
| Selective outcome reporting | Low | Results were reported for all outcomes specified in the abstract and methods. |
| Conflict of interest | Low | This study was supported by Electricite de France (EDF, French electric utility company, largely owned by the French government) as part of a research programme on Indoor Air Quality; authors declared no competing financial interests. |
| Other sources of bias | Low | No other threats to internal validity were identified. |
