# Supplementary material for: Exposure to formaldehyde and asthma outcomes: A systematic review, meta-analysis, and economic assessment
Source: PLoS One. 2021 Mar 31;16(3):e0248258. doi: 10.1371/journal.pone.0248258 (PMC8011796; doi:10.1371/journal.pone.0248258)
Supplement: S21 Table — (DOCX) [file pone.0248258.s034.docx]

Supplemental Materials, Table 21. Characteristics of Fornander et al. 2014

| Bias domain | Authors’ judgment | Support for judgment |
| --- | --- | --- |
| Source population representation | Low | A descriptive cross-sectional study of metal factory workers was conducted in Sweden to determine the respiratory effects from exposure to metal working fluids (MWF). All workers received a questionnaire and 78% participated (n=238). These subjects were divided into groups based on exposure to MWF based on work area and job duties. A reference group of (n=4780) school and office personnel were used as controls. Within each group 15 were selected to represent those with and without symptoms to evaluate biomarkers of exposure. There were no comparison of the exposure group to the reference group however in subjects with airway symptoms, demographics between exposed directly and indirectly and those in the no exposure were compared based on sex and smoking status indicating no significant differences between these groups. |
| Blinding | Probably high | No mention of blinding of participants or research personnel; Passive measurement of area and personal exposure monitoring not likely influenced by knowledge of exposure/outcome. Authors note that participants answered baseline questionnaire on exposure environment variables prior to recruitment for study and health status/symptoms assessed by self-report on a questionnaire. Occupational exposures may bias self-reported symptoms as workers may have some idea of their own exposures already. |
| Outcome assessment | Probably high | Respiratory symptoms related to asthma noted by self-report with no mention of validation of questionnaire instrument. |
| Confounding | Probably high | Analyses of within-subject changes controlled for potential time-invariant confounding factors. However between-subject analyses of exposed and non-exposed reported as conducted for participants of similar age, all non-smokers and "all men"; however results analyzed by simple Mann-Whitney U or Chi-squared tests with no mention of multivariate adjustment for other confounders. |
| Incomplete outcome data | Low | No missing outcome data reported. |
| Exposure assessment | Probably high | The authors also used work area and job tasks to assign direct, indirect, and no exposure groups. QA/QC methods were not reported. There is no evidence that control group was confirmed as unexposed. Although measured, FA levels were not used in the analysts. The authors used stationary and personal monitors to assess exposure to formaldehyde by UMEx 100 passive samplers from SKS for 6 hours. On follow-up only stationary monitors were used. Equipment was calibrated prior to use and all were measured by standard protocols by accredited labs. Detection limits were not presented. The FA exposure levels were not described for the direct and indirect exposure groups. |
| Selective outcome reporting | Low | Results were presented for all the relevant outcomes specified. |
| Conflict of interest | Low | The authors were either academic or government employees and state they had no competing interests. Funding was from the Research council of South East Sweden and the Cancer and Allergy Foundation. They indicate that the funding agencies had no role in the study design, execution or publication. |
| Other sources of bias | Probably high | Study recruited workers stationed at various places in the factory according to level of exposure to metal working fluids. Asthmatics were included but some of the most affected workers could have left the job prior to the study taking place, thus introducing a healthy worker bias, which would likely bias the results towards the null. |
