# Supplementary material for: Exposure to formaldehyde and asthma outcomes: A systematic review, meta-analysis, and economic assessment
Source: PLoS One. 2021 Mar 31;16(3):e0248258. doi: 10.1371/journal.pone.0248258 (PMC8011796; doi:10.1371/journal.pone.0248258)
Supplement: S22 Table — (DOCX) [file pone.0248258.s035.docx]

Supplemental Materials, Table 22. Characteristics of Fransman et al. 2003

| Bias domain | Authors’ judgment | Support for judgment |
| --- | --- | --- |
| Source population representation | Probably low | All subjects were workers at a plywood mill. Authors note recruitment of volunteers was through plywood mill site managers or team leaders, the exact number of workers approached for participation was unknown. Based on records, the authors estimate that 170 individuals were approached, and 112 (66%) participated in the study. Characteristics of the study participants are provided. |
| Blinding | Probably high | No mention of blinding of study personnel. Outcomes assessed through face-to-face questionnaire interviews of participants with questions which included knowledge of work characteristics and possible knowledge of assignment of high versus low exposure groups. |
| Outcome assessment | Probably low | Outcomes of respiratory symptoms and asthma prevalence assessed through face-to-face interviews of participants, however authors noted asthma prevalence assessed through ECRHS criteria questions. No information was provided on the validity of the questionnaires, or interviewer agreement. Asthma diagnosis confirmed by medical history, not objective testing. Based on description, can assume both groups were asked the same questions. |
| Confounding | Low | The authors adjusted for some Tier I and some Tier II confounders (age, gender, ethnicity, smoking). SES was not accounted for, however, all subjects were workers in similar positions at the same mill so it would not be unreasonable to assume they would all be similar SES. |
| Incomplete outcome data | Low | No missing outcome data reported. |
| Exposure assessment | Probably high | Personal formaldehyde samples were taken using NIOSH method 2016, for 22 individuals, each with a sampling time of 15 minutes. A detection limit of 0.03 ppm was reported. No duplicate measures were reported. The authors note potential for misclassification of exposure since workers were classified as to high or low exposure based on a small number of measurements (22 measurements for 112 workers). |
| Selective outcome reporting | Low | Results are presented for all outcomes described in the abstract and methods. |
| Conflict of interest | Probably high | No information was provided on sources of funding for the study. All authors are affiliated with academic institutions, and there is no reason to believe a conflict of interest exists. However, the authors note that basic costs of the study were covered by the plywood mill in which the study was conducted. |
| Other sources of bias | Probably high | Subjects were workers employed in a plywood mill and while asthmatics were included, some of the most affected could have left the job prior to the study taking place, thus introducing a healthy worker bias, which would likely bias the results towards the null. |
