# Supplementary material for: Exposure to formaldehyde and asthma outcomes: A systematic review, meta-analysis, and economic assessment
Source: PLoS One. 2021 Mar 31;16(3):e0248258. doi: 10.1371/journal.pone.0248258 (PMC8011796; doi:10.1371/journal.pone.0248258)
Supplement: S23 Table — (DOCX) [file pone.0248258.s036.docx]

Supplemental Materials, Table 23. Characteristics of Frey et al. 2014

| Bias domain | Authors’ judgment | Support for judgment |
| --- | --- | --- |
| Source population representation | Probably low | No information was provided on recruitment or enrollment. The authors note 77 residents (72 apartments out of 116 possible in the building) of a single, low-income senior housing building in Pheonix. Arizona participated. |
| Blinding | Probably low | Blinding was not addressed. Exposure was measured through air sampling. Respiratory outcomes were self-reported; however, authors note the exposure assessment and questionnaire administration was simultaneous and thus each are unlikely to influence the other. |
| Outcome assessment | Probably low | Respiratory health outcomes self-reported, however survey questions developed from applicable portions of the National Health Interview Survey and the Behavioral Risk Factor Surveillance System instruments. |
| Confounding | Probably low | Questionnaires included questions to assess emphysema, asthma, pets, smoking and consumer product use. Given the cross-sectional nature of this study many of the confounders were described in a table 1 in a comparison between smokers and non-smokers. |
| Incomplete outcome data | Low | 5 participants did not respond to the questionnaire. No other missing outcome data are reported. |
| Exposure assessment | Low | Sampling occurred over a one hour period during the survey administration. Researchers sampled for aldehydes using an active pump with a DNPH-coated gel sampler, analyzed by a referenced EPA method TO-11A which presumably would indicate detection limits and data quality objectives for the analytical results. They indicate equipment calibration and verification and repeat measures in 7% of apartments to ensure the one hour period of the day was not biasing the sampling. Sampling occurred both in the home and outdoors. |
| Selective outcome reporting | Low | Results were presented for all the relevant outcomes specified. |
| Conflict of interest | Low | The authors were academic or government and the funding was from the US Dept of Housing and Urban Development. |
| Other sources of bias | Low | No other threats to internal validity were identified. |
