# Supplementary material for: Exposure to formaldehyde and asthma outcomes: A systematic review, meta-analysis, and economic assessment
Source: PLoS One. 2021 Mar 31;16(3):e0248258. doi: 10.1371/journal.pone.0248258 (PMC8011796; doi:10.1371/journal.pone.0248258)
Supplement: S24 Table — (DOCX) [file pone.0248258.s037.docx]

Supplemental Materials, Table 24. Characteristics of Frigas et al. 1984

| Bias domain | Authors’ judgment | Support for judgment |
| --- | --- | --- |
| Source population representation | Probably low | The study included 13 patients between the ages of 15 and 70 years who requested that local health agencies test indoor concentrations of formaldehyde. Characteristics of the participants are provided. |
| Blinding | Probably high | Authors noted the bronchial challenge sequence was double-blinded and randomized in 3 of 13 patients and single-blinded in the rest. Rated probably high risk of bias because not all of the formaldehyde challenges were double-blinded and there was no justification as to why most were only single-blinded. With single blinding, if the investigator is aware of the exposure level there is an opportunity for biased measurement of the outcome. |
| Outcome assessment | Low | General physical examination, chest roentgenography, pulmonary function tests, and standard laboratory tests were performed on the first day. Spirometry was performed according to standard techniques before the challenge, at 0, 15, and 30 minutes, and 1, 3, 6, and 24 hours after the challenge. Efforts were made in triplicate and the best efforts were used. |
| Confounding | Probably low | The study examined smoking (Tier I), sex, age, and occupation (Tier II). SES was not addressed. |
| Incomplete outcome data | Low | There is no missing data. |
| Exposure assessment | Probably low | Known concentrations of formaldehyde were delivered via a Dynacalibrator. Accurate delivery was confirmed by measuring the concentration of formaldehyde gas at the end of the delivery system. Additional details on the QA/QC process were not provided. |
| Selective outcome reporting | Low | Results were reported for all outcomes specified in the abstract and methods. |
| Conflict of interest | Low | Authors noted this investigation was supported in part by a grant from the NIH, and authors were affiliated with a graduate school of medicine. |
| Other sources of bias | Low | No other threats to internal validity were identified. |
