# Supplementary material for: Exposure to formaldehyde and asthma outcomes: A systematic review, meta-analysis, and economic assessment
Source: PLoS One. 2021 Mar 31;16(3):e0248258. doi: 10.1371/journal.pone.0248258 (PMC8011796; doi:10.1371/journal.pone.0248258)
Supplement: S25 Table — (DOCX) [file pone.0248258.s038.docx]

Supplemental Materials, Table 25. Characteristics of Frisk et al. 2002

| Bias domain | Authors’ judgment | Support for judgment |
| --- | --- | --- |
| Source population representation | Low | A consecutive series of adult asthmatics, recruited from the outpatient clinic of the Department of Respiratory Medicine, Örebro University Hospital, were asked to participate in the study. Inclusion criteria were increasing asthma symptoms in the home environment, that subjects should plan to live in their homes during the following 18 months, and that the proposed intervention should be implemented during one year from time point O1. Exclusion criteria were described in detail. 45 asthmatics were eligible to participate but six of these were excluded. An additional 18 participants were excluded from follow up due to either the inability to perform the planned adaptation or presence of other diseases or conditions. |
| Blinding | Probably high | There was no discussion of blinding of participants or study staff. The indoor environment was monitored by occupational therapists using environmental monitoring methods. Interventions included removing wall-to-wall carpets or improving air exchange (i.e., improved ventilation). Outcome measures were by self-report (diary), lung function tests, and histamine tests. Participants were most likely aware of interventions, which could potentially bias the reporting of outcomes. |
| Outcome assessment | Low | At the medical investigations, established clinical methods were sued. Lung function was tested by spirometry. Bronchial hyper-responsiveness was measured by histamine provocation. PEF was measured by a peak flow meter and the values were self-recorded. Symptoms and medicine use were self-reported. The authors note that for some participants, there were fewer diary values or missing data from periods with stable asthma at both the pre- and post-tests. |
| Confounding | Probably low | Authors excluded participants with tobacco smoke exposure, but did not consider SES. They considered some Tier II confounders including age, sex, and other environmental co-exposures. |
| Incomplete outcome data | Probably low | The reasons for excluding participants from follow-up were described in detail and not likely to be related to true outcome. Authors do not discuss potential differences between those included or excluded from the study. |
| Exposure assessment | Probably low | Formaldehyde was measured by passive diffusion on 2,4-DNF filters for 2 weeks and the method paper is referenced (High-performance liquid-chromatography). No QA/QC methods were presented. |
| Selective outcome reporting | Low | Results are reported for all outcomes specified in the abstract and methods. |
| Conflict of interest | Low | The study was funded by government and a non-governmental foundation. All authors were affiliated with hospitals or academic institutions, and there is no reason to believe that a conflict of interest exists. |
| Other sources of bias | Low | No other threats to internal validity were identified. |
