# Supplementary material for: Exposure to formaldehyde and asthma outcomes: A systematic review, meta-analysis, and economic assessment
Source: PLoS One. 2021 Mar 31;16(3):e0248258. doi: 10.1371/journal.pone.0248258 (PMC8011796; doi:10.1371/journal.pone.0248258)
Supplement: S26 Table — (DOCX) [file pone.0248258.s039.docx]

Supplemental Materials, Table 26. Characteristics of Frisk et al. 2006

| Bias domain | Authors’ judgment | Support for judgment |
| --- | --- | --- |
| Source population representation | Low | In this case control study the authors adequately describe inclusion and exclusion criteria of participants from the Orebro section of the 1996 FinEsS study. Cases and controls were subject to the same inclusion criteria. Cases were identified as persons who had replied ‘‘Yes’’ to all four questions of a 1996 survey. Controls were randomly selected and matched for age group, gender, and type of accommodation, from those who had responded ‘‘No/Don’t know’’ to all four questions described above. Authors note the amount and reasons for loss to follow up from the 1996 survey, but rates are comparable between study groups. The selection process is robust and well-described. |
| Blinding | Low | An experienced construction engineer without knowledge of case status performed home inspection for building characteristics, a biomedical scientist or occupational therapist performed physical, chemical and biological measurements in each participants bedroom. Cases were matched to controls and there were no significant differences in the case and control groups. |
| Outcome assessment | Probably low | The cases were self-reported as doctor diagnosed asthmatics and had to answer yes to 4 additional questions to confirm case status. Controls answered No/Don't Know to all five questions. Although the reliability of the questionnaire was not reported (but all questions had to have a yes answer). |
| Confounding | Probably high | This was a matched case control study. The researchers measured most Tier I and some Tier II confounders and evaluated differences between cases and controls. This was a matched study, but they did not control for ETS, or evaluate ETS differences between cases and controls (higher in cases). Also did not adjust for SES. |
| Incomplete outcome data | Low | The proportion of cohort study members not interested in participating in this study were similar among cases and controls. The authors lead us to believe that complete cases and controls were those used in the study. |
| Exposure assessment | Probably low | Formaldehyde was measured for a 24-hr period by commercial diffusion measurements (GMD, Systems Inc.) using a 2.4 DNF filter and an uptake of 25.2 ml/min. Formaldehyde concentrations were quantified using HPLC, and the estimated uncertainty of the analysis was reported to be 15%. QC methods were not reported. No repeated measurements. Housing environment factors were also accounted for. |
| Selective outcome reporting | Low | All outcomes outlined in abstract and method section have been reported. |
| Conflict of interest | Low | The authors were academic and this study was supported by the Swedish Council of Building Research, Asthma and Allergy Scientific Foundation, and by departmental support from the authors’ university hospital. There is no reason to expect potential conflict of interest. |
| Other sources of bias | Probably low | The primary aim of this study was to evaluate housing characteristics in asthmatics and non-asthmatics, not the impact of exposure to formaldehyde on the risk of asthma. The secondary aim evaluated physical, chemical and biological differences between these groups. |
