# Supplementary material for: Exposure to formaldehyde and asthma outcomes: A systematic review, meta-analysis, and economic assessment
Source: PLoS One. 2021 Mar 31;16(3):e0248258. doi: 10.1371/journal.pone.0248258 (PMC8011796; doi:10.1371/journal.pone.0248258)
Supplement: S27 Table — (DOCX) [file pone.0248258.s040.docx]

Supplemental Materials, Table 27. Characteristics of Frisk et al. 2009

| Bias domain | Authors’ judgment | Support for judgment |
| --- | --- | --- |
| Source population representation | Probably low | Participants were selected from the Orebro section of the 1996 FinEsS study. Details of the FinEsS study are provided elsewhere. 49 asthma cases between ages 15-54 were recruited for an investigation of their housing and a clinical examination. |
| Blinding | Probably low | No discussion on investigator blinding. All participants had asthma so that outcome was already known by investigators. Home inspections and clinical investigations who knew exposure risks may not have been blinded in measuring lung function outcomes, so there is potential for bias since blinding is not explicitly discussed. |
| Outcome assessment | Low | Subjects were persons with asthma (identified as having answered positively to four questions about physician-diagnosed asthma, current use of asthma medication, attacks of breathlessness, and episodes of wheezing). An expanded lung function test following American Thoracic Society Standards was performed. Three expiratory measurements were performed by trained nurses using a calibrated spirometer. The best of the three measurements was used for FEV1 and PEF. Bronchial responsiveness was tested through a methacholine challenge test and performed using a Vitalograph1 spirometer, calibrated daily. Bronchial provocation tests were performed in 39 of the 49 subjects. |
| Confounding | Probably high | The researchers performed measures of Tier I and some Tier II confounders. However, the analyses only accounted for water content, moisture content, CO2 and dust mites. The authors indicate that they did not control for asthma medication use prior to the clinical exams. |
| Incomplete outcome data | Low | The authors lead us to believe that complete data were used in the study. |
| Exposure assessment | Probably low | Formaldehyde was measured over a 24 hour period by diffusion and analyzed by HPLC. No details were provided on QA/QC. |
| Selective outcome reporting | Low | All outcomes outlined in abstract and method section have been reported. |
| Conflict of interest | Low | The authors had academic affiliations and this study was supported by the Swedish Council of Building Research and by Orebro County Council Research Committee. |
| Other sources of bias | Probably low | Formaldehyde was not detected above WHO cut-off levels and therefore could not be included in the analysis when comparing occurrence vs. non-occurrence. If lower cut-off levels described for sensitive persons was used, then formaldehyde would have been included. |
