# Supplementary material for: Exposure to formaldehyde and asthma outcomes: A systematic review, meta-analysis, and economic assessment
Source: PLoS One. 2021 Mar 31;16(3):e0248258. doi: 10.1371/journal.pone.0248258 (PMC8011796; doi:10.1371/journal.pone.0248258)
Supplement: S28 Table — (DOCX) [file pone.0248258.s041.docx]

Supplemental Materials, Table 28. Characteristics of Gannon et al. 1995

| Bias domain | Authors’ judgment | Support for judgment |
| --- | --- | --- |
| Source population representation | Low | Eight subjects were assessed for occupational asthma due to glutaraldehyde. They were a consecutive series of workers with direct or indirect exposure to glutaraldehyde referred to a specialist occupational lung disease clinic. |
| Blinding | Probably high | There is no discussion on blinding. Workers were likely aware of workplace exposure to glutaraldehyde. No mention as to whether investigator measuring pulmonary lung functions were blinded to each participant's exposure status. |
| Outcome assessment | Low | Bronchial provocation tests were performed in a challenge chamber for 10 minutes. Methacholine or histamine challenges were performed on the day before and the day after the provocation tests following a previously described method. FEV1 was measured at regular intervals prior to and after the provocation tests. Occupational asthma was diagnosed based on criteria well described. Objective measures were used (bronchial provocation tests) to determine outcomes. |
| Confounding | Low | Workers' characteristics are presented (age, sex, smoking, occupation, years of exposure, agents of exposure). No regression analyses were performed (since this is a case series), so no need to formally account for confounders. |
| Incomplete outcome data | Probably low | Some tests were not done in certain subjects and the reasons are not specified. However, this is not likely to truly bias the results since this is a case series report. |
| Exposure assessment | Probably low | Exposure of concern in the study is glutaraldehyde, and it was measured with well reported, valid methods; however, formaldehyde exposure was controlled in a chamber (1% FA challenge). |
| Selective outcome reporting | Low | Results were reported for all outcomes specified in the abstract and methods. |
| Conflict of interest | Probably low | Authors were affiliated with a hospital or academic institution. There is no reason to believe that a conflict of interest exists. |
| Other sources of bias | Probably low | Subjects were recruited who were exposed to glutaraldehyde and had a history of asthmatic symptoms that improved when away from work. Since the only subjects included were those with reasonably severe symptoms linked specifically to their job, this study may have a more limited risk of healthy worker bias, which would likely bias the results towards the null. |
