# Supplementary material for: Exposure to formaldehyde and asthma outcomes: A systematic review, meta-analysis, and economic assessment
Source: PLoS One. 2021 Mar 31;16(3):e0248258. doi: 10.1371/journal.pone.0248258 (PMC8011796; doi:10.1371/journal.pone.0248258)
Supplement: S29 Table — (DOCX) [file pone.0248258.s042.docx]

Supplemental Materials, Table 29. Characteristics of Garrett et al. 1999

| Bias domain | Authors’ judgment | Support for judgment |
| --- | --- | --- |
| Source population representation | Probably high | Households with children aged 7-14 were recruited from two Australian towns in the same region. Authors report that information sheets were initially distributed at schools and medical centers, but later they also utilized press advertisements due to difficulty recruiting non-asthmatics. More families with allergy problems volunteered to participate. |
| Blinding | Probably low | Blinding was not addressed. The main outcomes of asthma, atopy, and respiratory symptoms were self-reported using a questionnaire that participants completed on the last day of exposure monitoring; therefore, formaldehyde exposure would have been quantified after the fact, and it is unlikely that participants were aware of the formaldehyde levels in their homes. However, authors do not report whether the analytical chemists were blinded to participants' outcome status. |
| Outcome assessment | Probably low | Parents reported respiratory symptoms in a validated questionnaire modified for this study. The authors note that major recall bias was unlikely. Asthma was diagnosed by a physician, but diagnoses were not confirmed against medical records. |
| Confounding | Probably low | Smoking status but not SES (Tier I) and some of Tier II (gender, parental asthma/allergy), as well as additional environmental exposures (indoor NO2, airborn fungal, house dust mite) considered for final models. |
| Incomplete outcome data | Probably high | There are some issues with the numbers provided in the paper. For example, authors state in the methods that there are 53 asthmatic children, but then list n=57 asthmatics in table 2. Also, they mislabeled table 4 to read "nonatopic" and "atopic" when I believe they meant "nonasthmatic" and "asthmatic". But this table is also confusing because here they list the numbers 53 and 95, which don't match the numbers in the previous tables. While it is possible that these mistakes do not bias the results, it introduces so many questions it makes it difficult to judge, thus justifying the probably high ROB rating. |
| Exposure assessment | Probably low | Formaldehyde was measured each season using passive samplers for four days in participants' bedrooms, living rooms, kitchens, and outdoors. No QA/QC methods were provided. |
| Selective outcome reporting | Low | Results were reported for all relevant outcomes outlined in the abstract and methods. |
| Conflict of interest | Low | Funding for the study was provided by the Australian Research Council and university funds. All authors were affiliated with academic institutions. |
| Other sources of bias | Low | No other threats to validity were identified. |
