# Supplementary material for: Exposure to formaldehyde and asthma outcomes: A systematic review, meta-analysis, and economic assessment
Source: PLoS One. 2021 Mar 31;16(3):e0248258. doi: 10.1371/journal.pone.0248258 (PMC8011796; doi:10.1371/journal.pone.0248258)
Supplement: S30 Table — (DOCX) [file pone.0248258.s043.docx]

Supplemental Materials, Table 30. Characteristics of Gorski et al. 1991

| Bias domain | Authors’ judgment | Support for judgment |
| --- | --- | --- |
| Source population representation | Probably high | Authors reported limited information on selection/recruitment. The study included 367 workers occupationally exposed to formaldehyde for at least one year. All subjects suffered from respiratory symptoms. No additional information on inclusion/exclusion criteria, recruitment, participation rates, or demographics are provided. |
| Blinding | Probably high | Authors noted single-blind crossover results for peak expiratory flow in subjects suffering from respiratory symptoms. Investigator knowledge of exposure status for participants could have influenced the measurement of lung function measures. |
| Outcome assessment | Low | Main spirometry outcomes of FEV1, vital capacity measured through spirometry, skin prick testing and allergic reactions to formaldehyde-specific IgE antibodies with serologic assessment by ELISA (objective measures). Chronic respiratory disease was diagnosed according to the American Thoracic Society. |
| Confounding | Low | Authors report information on smoking, age, and possible co-exposures. However, the crossover design used the subjects as their own controls, hence lack of adjustment for certain confounders is not likely to induce bias. |
| Incomplete outcome data | Low | No missing outcome data reported. |
| Exposure assessment | Probably high | A true exposure assessment was not performed, and work location was used as a proxy measure. |
| Selective outcome reporting | Low | Results were presented for all the relevant outcomes specified. |
| Conflict of interest | Probably low | Authors were medical researchers. Source of funding was not reported. |
| Other sources of bias | Probably high | The study recruited workers who were occupationally exposed to formaldehyde for at least a year. Asthmatics were included but some of the most affected workers could have left the job prior to the study taking place, thus introducing a healthy worker bias, which would likely bias the results towards the null. |
