# Supplementary material for: Exposure to formaldehyde and asthma outcomes: A systematic review, meta-analysis, and economic assessment
Source: PLoS One. 2021 Mar 31;16(3):e0248258. doi: 10.1371/journal.pone.0248258 (PMC8011796; doi:10.1371/journal.pone.0248258)
Supplement: S31 Table — (DOCX) [file pone.0248258.s044.docx]

Supplemental Materials, Table 31. Characteristics of Green et al. 1987

| Bias domain | Authors’ judgment | Support for judgment |
| --- | --- | --- |
| Source population representation | Probably high | The researchers recruited subjects through a newspaper advertisement that was looking for research volunteers. The advertisement did not mention formaldehyde so the subjects were self-selected and underwent clinical examination to identify normal subjects and those with a clinical history of asthma 6 weeks prior to the trial. No demographic information provided. |
| Blinding | Low | The authors used a randomized block experimental design with a random presentation of the stimulus. Subjects and pulmonary technicians were not told which exposure was being performed. |
| Outcome assessment | Low | Researchers performed an extensive clinical assessment of each subject prior to group assignment and conducting trials. The main outcomes of FEV1 and FVC were measured in experimental setting using standard methods. Asthmatics had a confirmed clinical history of asthma and normal subjects had no reported history of allergies or hayfever. The researchers assessed respiratory symptoms and severity through questionnaire and spirometric measures using 10-L Stead-Wells spirometer interfaced with an Eagle II microprocessor at 0, 17, 25, 47, and 55 min. Lung function measures of FVC, FEV1, FEF25-75, met the criteria set forth by the American Thoracic Society. Airway resistance and FRC were determined with a modified whole-body pressure plethysmographs technique of Dubois and associates. Paired T-test were used to compare subjects outcomes at each time point between exposed and unexposed trials. |
| Confounding | Low | All subjects were nonsmokers, but SES was not addressed. Several tier 2 confounders were measured. Asthmatics were taken off medications 48 prior to exposures. No subjects were allowed to take anti-histamines within 12 hours of the trials. Given the randomized exposure and each subject being its own control one would expect low risk of bias given the amount of protocols in place. |
| Incomplete outcome data | Low | No missing outcome data reported. |
| Exposure assessment | Low | Formaldehyde concentration within the experimental exposure chamber was monitored continuously by colorimetric monitors specific for formaldehyde and calibrated weekly, daily exposure samples also conducted and analyzed for formaldehyde using NIOSH methods. |
| Selective outcome reporting | Low | Results were presented for all the relevant outcomes specified. |
| Conflict of interest | Low | This study was funded by the Department of Energy and authors were university affiliated. |
| Other sources of bias | Low | No other threats to internal validity were identified. |
