# Supplementary material for: Exposure to formaldehyde and asthma outcomes: A systematic review, meta-analysis, and economic assessment
Source: PLoS One. 2021 Mar 31;16(3):e0248258. doi: 10.1371/journal.pone.0248258 (PMC8011796; doi:10.1371/journal.pone.0248258)
Supplement: S32 Table — (DOCX) [file pone.0248258.s045.docx]

Supplemental Materials, Table 32. Characteristics of Hanson et al. 1993

| Bias domain | Authors’ judgment | Support for judgment |
| --- | --- | --- |
| Source population representation | Probably high | The authors noted the self-administration of a voluntary questionnaire provided for employees of the 4th floor and other areas of one hospital. However no other information on recruitment strategies was provided. The authors noted higher participation rates on the 4th floor and an awareness of participants of the complaints from 4th floor employees. |
| Blinding | Probably high | There is no evidence of blinding, and it is possible that workers were aware of their exposure. Authors noted awareness of 4th floor exposures in study subjects as workers had complained about air quality since its opening. Main outcomes of physician-diagnosed asthma, etc. as well as respiratory symptoms were self-reported and could have been potentially biased by knowledge of exposure. |
| Outcome assessment | Probably low | Respiratory symptoms related to asthma noted by self-report; authors noted only some questions were adapted from the American Thoracic Society Respiratory Diseases questionnaire. Asthma diagnosis confirmed by medical history, not objective testing so rated probably low. |
| Confounding | Probably low | Analyses were stratified by smoking status, ES (by job description) (Tier I), age, gender, and Registered Nurse status (Tier II). |
| Incomplete outcome data | Low | There is no missing data for subjects included in the analyses. |
| Exposure assessment | Probably high | Chemical analyses were performed using site and individual monitoring. The authors note that there may have been a laboratory error in the formaldehyde measurements in the operating room area. Exposure to common hospital chemicals were self-reported. |
| Selective outcome reporting | Low | Results were reported for all outcomes specified in the abstract and methods. |
| Conflict of interest | Probably low | All authors were affiliated with an academic institution. Information on study funding was not provided, but there is no reason to believe that a conflict of interest exists. |
| Other sources of bias | Probably high | Asthmatics were included but some of the most affected workers could have left the job prior to the study taking place, thus introducing a healthy worker bias, which would likely bias the results towards the null. Although this is not designed as an occupational study per se, as workers were not knowingly exposed to formaldehyde because of working conditions, some air samples did indicate a high level of formaldehyde (over limits). |
