# Supplementary material for: Exposure to formaldehyde and asthma outcomes: A systematic review, meta-analysis, and economic assessment
Source: PLoS One. 2021 Mar 31;16(3):e0248258. doi: 10.1371/journal.pone.0248258 (PMC8011796; doi:10.1371/journal.pone.0248258)
Supplement: S33 Table — (DOCX) [file pone.0248258.s046.docx]

Supplemental Materials, Table 33. Characteristics of Harving et al. 1990

| Bias domain | Authors’ judgment | Support for judgment |
| --- | --- | --- |
| Source population representation | Probably low | Fifteen nonsmoking (8 female, 7 male) volunteers were selected on the basis of substantial bronchial hyperreactivity to histamine, with no patients using methylxanthines or corticosteroids. The authors recruited 15 nonsmoking volunteers that were known to have substantial bronchial hyper reactivity to histamine. No inclusion or exclusion criteria were reported. |
| Blinding | Low | This was a double-blinded study. |
| Outcome assessment | Low | The main outcomes in this study were direct measures of FEV1 and bronchial reactivity measured by monitoring lung function following exposure to increasing doses of histamine. Measures of lung function were taken ever 30 min during the exposure through to follow up bronchial reactivity test, FEV1 was taken by participant every 2 hours following exposure. While the authors describe all the equipment used in these measures they do not provide information regarding calibration checks or other QC commonly performed. Participants were questioned every 15 mins during exposure for their severity of asthma symptoms on a visual analog scale not visible to other participants. Study rated low risk of bias because objective measures (pulmonary function test) used to determine outcomes. |
| Confounding | Low | This was a controlled exposure where the researchers controlled the use of bronchial dilators and oral medications use on the day of exposure. Other tier I confounders such as passive smoking exposure at home were not addressed by the authors. However, each participant served as their own control. |
| Incomplete outcome data | Low | No missing data reported. |
| Exposure assessment | Low | The authors performed 3 controlled exposures in a chamber with groups of 5 participants. The authors described the parameters of the exposure chamber and indicate that chamber concentrations were confirmed using the acetylacetone method. |
| Selective outcome reporting | Low | Results were presented for all the relevant outcomes specified. |
| Conflict of interest | Low | The authors were academic and this research was support by a grant from the National Union Against Lung Diseases in Denmark. |
| Other sources of bias | Low | No other threats to internal validity were identified. |
