# Supplementary material for: Exposure to formaldehyde and asthma outcomes: A systematic review, meta-analysis, and economic assessment
Source: PLoS One. 2021 Mar 31;16(3):e0248258. doi: 10.1371/journal.pone.0248258 (PMC8011796; doi:10.1371/journal.pone.0248258)
Supplement: S34 Table — (DOCX) [file pone.0248258.s047.docx]

Supplemental Materials, Table 34. Characteristics of Hendrick et al. 1977

| Bias domain | Authors’ judgment | Support for judgment |
| --- | --- | --- |
| Source population representation | Low | Study subjects are all the 28 members of the nursing staff of a haemodialysis unit. Subject characteristics such as smoking prevalence, age distribution are described. |
| Blinding | High | Blinding was not possible as all subjects in the occupational settling were had been known to have history of exposure. During the inhalation provocation tests it would not be possible to be blind to the exposure for either participant and likely not the investigator, which could potentially lead to biased outcome measurements. |
| Outcome assessment | Low | Lung function outcomes were evaluated with simple tests of airways function (peak expiratory flow (PEF) using a Wright's meter, and one second forced expiratory volume (FEV1), and forced vital capacity (FVC) using a Vitalograph dry spirometer. However, QA/QC information was not addressed. Study rated low risk of bias because used objective measures (pulmonary function tests) to test for outcomes. |
| Confounding | High | Only age and smoking were described. The results are descriptive, with no analyses accounting for confounders. |
| Incomplete outcome data | Low | Case reports were all described and details of the 28 workers were presented. |
| Exposure assessment | Probably high | There are no measured formaldehyde exposure levels in this study. Exposure status defined by being at the workplace. There is no doubt that everyone was exposed, and thus differential exposure assessment is unlikely. |
| Selective outcome reporting | Low | All of the published manuscript's outcomes outlined in the methods, abstract, and/or introduction section that are of interest in the review have been reported in the specified way. |
| Conflict of interest | Probably high | The authors' affiliation is the hospital in the study. The study appeared to have been funded by the employer who may have a financial stake in the outcome - if there are very high exposures or health outcomes identified there will need to be action taken which can be costly to address the exposures. |
| Other sources of bias | Probably high | Subjects were staff members in a haemodialysis unit exposed to high levels of formaldehyde used to sterilize artificial kidneys. While individuals were included with asthma symptoms, some of the most affected could have left the job prior to the study taking place, thus introducing a healthy worker bias, which would likely bias the results towards the null. |
