# Supplementary material for: Exposure to formaldehyde and asthma outcomes: A systematic review, meta-analysis, and economic assessment
Source: PLoS One. 2021 Mar 31;16(3):e0248258. doi: 10.1371/journal.pone.0248258 (PMC8011796; doi:10.1371/journal.pone.0248258)
Supplement: S35 Table — (DOCX) [file pone.0248258.s048.docx]

Supplemental Materials, Table 35. Characteristics of Herbert et al. 1988

| Bias domain | Authors’ judgment | Support for judgment |
| --- | --- | --- |
| Source population representation | Probably low | Workers at a plant involved in the manufacturing of oriented strand board were included in this study; of the 165 workers employed at the plant, only the 99 who were actively involved in operations or maintenance were included. A group of 165 oil field and gas plant workers from the same geographic area were included as referents; workers who reported having had an exposure to hydrogen sulfide that caused them to lose consciousness (n=14) were excluded from the group because they were found to have significant excess of certain respiratory symptoms. The study group had more current smokers, and the referent group had more non-smokers, but the number of pack-years were comparable. There is no information provided on inclusion/exclusion criteria. |
| Blinding | Probably high | There is no evidence of blinding. Participants may not have been aware of exposure status since they were both from occupational sector; but personnel were likely aware of the exposure groups and measured outcomes (lung function) could have potentially been biased with investigator knowledge. |
| Outcome assessment | Low | Respiratory symptoms were reported in a questionnaire conducted by interviewers trained in the proper interpretation of the questions and unbiased methods of questionnaire administration. Spirometric tests were performed with a minimum of five technically satisfactory maneuvers, and were assessed using the Snowbird criteria. The testing protocol was identical for both groups with the exception of the post-shift spirometry measurement, which the reference group did not perform. Study rated as low risk of bias because used objective measures (pulmonary function tests) to determine outcomes. |
| Confounding | Low | The analysis was controlled for cigarette pack-years and adjusted for age and height. Authors also considered years of employment. While authors did not specify controlling for SES, the authors compared groups of employees; one group was involved in production of materials with formaldehyde, and the other was oil and gas plant workers. Given they were both similar occupational type jobs, it would not be unreasonable to assume that that they might be of similar SES. |
| Incomplete outcome data | Low | No reason to believe there were incomplete outcome data. |
| Exposure assessment | Probably low | The sampling strategies and selection of sampling instruments were based on OSHA's technical manual for organic vapors and gases. Formaldehyde samples were collected at five work sites for 21 hours of continuous sampling on 2 days. The sampler included a filter cassette, midget impinger, secondary impinger, and a desiccant trap connected to an air sampling pump. No additional QA/QC methods were described. |
| Selective outcome reporting | Low | Results were reported for all outcomes specified in the abstract and methods. |
| Conflict of interest | Low | Authors were medical and government researchers. Funding was provided by the Alberta Occupational Health and Safety Heritage Grant Program and the National Health Research and Development Program. |
| Other sources of bias | Probably high | Subjects were oriented strand board workers and controls were individuals employed in the log yard or as office staff. While individuals were included with asthma, some of the most affected could have left the job prior to the study taking place, thus introducing a healthy worker bias, which would likely bias the results towards the null. |
