# Supplementary material for: Exposure to formaldehyde and asthma outcomes: A systematic review, meta-analysis, and economic assessment
Source: PLoS One. 2021 Mar 31;16(3):e0248258. doi: 10.1371/journal.pone.0248258 (PMC8011796; doi:10.1371/journal.pone.0248258)
Supplement: S36 Table — (DOCX) [file pone.0248258.s049.docx]

Supplemental Materials, Table 36. Characteristics of Horvath et al. 1988

| Bias domain | Authors’ judgment | Support for judgment |
| --- | --- | --- |
| Source population representation | Low | Participants were workers from a wood processing/manufacturing facility (test group) and two nearby food-processing facilities (control group) in Wisconsin. 71% of formaldehyde-exposed individuals (112/159) agreed to participate, and 300 representative control individuals agreed to participate. Four test subjects (4%) and 25 controls (8%) were excluded because of unsatisfactory spirometry results, and an additional 21 controls were excluded because of previous formaldehyde exposure (12), absence from post-shift testing (7), and to match the test group's race/ethnicity makeup (2). The groups did not differ significantly in age or sex distribution. |
| Blinding | Probably high | There is no evidence of blinding. Participants were likely aware of their exposure risk. Blinding of key study personnel was not mentioned. |
| Outcome assessment | Low | Spirometry was conducted before and after the shift by trained technicians according to the American Thoracic Society procedures. The pre-shift evaluation was always performed on a Monday or the first day after a two-day break. Symptoms were self-reported using a modified American Thoracic Society respiratory symptom questionnaire before and after the monitored work shift. |
| Confounding | Probably low | Analyses accounted for some Tier 1 (smoking status) and Tier II confounders (age, sex, height, and mobile home residency, duration of exposure). |
| Incomplete outcome data | Low | 10% of data was excluded for the test group (4%) due to unsatisfactory spirometry results. 10% of data was excluded for the control group (15%) due to unsatisfactory spirometry results (25/300), previous formaldehyde exposure (12/300), absence from post-shift testing (7/300), and to match the test group's race/ethnicity makeup (2/300). Authors attempted to control for the loss of control subjects, and noted that there was no statistical difference between the number of test and control subjects excluded because of deficiencies in spirometric technique. |
| Exposure assessment | Probably high | Passive badge samplers were used to measure personal exposure for 8 hours, with a detection limit of 0.1 ppm and a high degree of specificity. Area levels were measure by active sampler. Analysis was performed by spectrophotometry, and badge data were increased by 20% to adjust for collection efficiency based on previous method studies and independent evaluation of the badges. Control subjects had active area dosimeter measures within work site, however it was not mentioned that non-exposed workers wore personal exposure monitors. |
| Selective outcome reporting | Low | Results are presented for all outcomes specified in the abstract and methods. |
| Conflict of interest | High | The lead author is affiliated with BP America, Inc. The employer of the exposed group also performed the analytical measurements of formaldehyde. |
| Other sources of bias | Probably low | Subjects were workers employed at the Weyerhaeuser Co who were exposed to airborne formaldehyde and control subjects were from 2 nearby food-processing facilities. Authors attempted to address healthy worker bias by looking at the reasons for why employees left their job over the prior 4 years. Out of 54 employees who left, 2 left because of exacerbation of preexisting respiratory conditions (asthma, bronchiectasis). The authors were able to administer a modified symptom questionnaire to 24 of the 54 living former employees, although it is not clear if that includes those that left for exacerbation of respiratory illness. The former employees did not differ from test subjects with regards to symptoms. |
