# Supplementary material for: Exposure to formaldehyde and asthma outcomes: A systematic review, meta-analysis, and economic assessment
Source: PLoS One. 2021 Mar 31;16(3):e0248258. doi: 10.1371/journal.pone.0248258 (PMC8011796; doi:10.1371/journal.pone.0248258)
Supplement: S37 Table — (DOCX) [file pone.0248258.s050.docx]

Supplemental Materials, Table 37. Characteristics of Hsu et al. 2012

| Bias domain | Authors’ judgment | Support for judgment |
| --- | --- | --- |
| Source population representation | Low | Detailed descriptions are included of the population demographic characteristics, as well as inclusion/exclusion criteria, enrollment procedures, participation rates and follow-up rates. They evaluated the cases and controls for any differences. |
| Blinding | Probably low | The final cases and controls were ultimately determined according solely to the clinical diagnosis of practicing pediatricians prior to the sampling of homes. Exposure measurements were conducted through household sampling by trained investigators and is unlikely that those sampling were aware of outcome of children living there or that this would bias their measurements. Blinding was not explicitly discussed. |
| Outcome assessment | Probably low | Subject’s health status was verified by pediatricians, and parents recorded observed daily symptoms of their children for the week that the home investigation visit took place; no objective testing was performed to measure outcome. |
| Confounding | High | The analyses with formaldehyde as the exposure of concern do not account for potential confounders. Differences in factors found between study subjects and the original population, including child’s gender, parental education level, parental allergic history, and parental smoking status, were all considered as potential confounders in models with phthalates as the exposure of concern. |
| Incomplete outcome data | Low | There is no evidence of missing outcome data in the study subjects. |
| Exposure assessment | Probably low | The method for indoor formaldehyde (HCOH) sampling and analyzing adopted the ASTM D5197-03 method. Formaldehyde was collected by DNPH sampling cartridges operating at a flow rate of 100 l/min for 2 h. The samples were analyzed within 1 month after the date of collection by HPLC using UV detection at 360 nm. Limit of detection was not reported, but all values appear to be above detection. However, method but doesn't account for measures outside of the home. |
| Selective outcome reporting | Low | All of the published manuscript's outcomes outlined in the methods, abstract, and/or introduction section that are of interest in the review have been reported in the specified way. |
| Conflict of interest | Low | This study was supported by a government entity (Taiwan National Science Council) and the authors declared no conflicting financial interests. |
| Other sources of bias | Probably low | The main focus of the study is the effect of phthalates on the risk of childhood allergy, asthma, and reported symptoms; not necessarily with a focus on formaldehyde. Other concerns include: ages of children; Asthma and Rhinitis are 'lumped' together. Although the conditions are related, this is not a study specific to "asthma". The study methods state, "Children who had at least two or more parent-reported respiratory and allergic diseases (asthma, allergic rhinitis) or symptoms (wheezing, coughing at night, eczema, sneezing, runny or stuffy nose) during the preceding 12 months were recruited as the potential case subjects." |
