# Supplementary material for: Exposure to formaldehyde and asthma outcomes: A systematic review, meta-analysis, and economic assessment
Source: PLoS One. 2021 Mar 31;16(3):e0248258. doi: 10.1371/journal.pone.0248258 (PMC8011796; doi:10.1371/journal.pone.0248258)
Supplement: S38 Table — (DOCX) [file pone.0248258.s051.docx]

Supplemental Materials, Table 38. Characteristics of Huang et al. 2016

| Bias domain | Authors’ judgment | Support for judgment |
| --- | --- | --- |
| Source population representation | Probably low | Potential selection bias of entering the case-control study would not necessarily be differential between cases (those with doctor-diagnosed asthma) and controls (those without doctor-diagnosed asthma). |
| Blinding | Low | The enrollment of cases and controls was not blinded as the choice of controls was based on their being in the same community as the cases; there was no connection between outcome assessment (done by parents) and exposure assessment (done by blinded inspectors). The inspectors did not know the heath status of the homes they visited. |
| Outcome assessment | Low | Subject’s asthma status was based on doctor’s diagnosis. Parents also reported information for the child’s history of asthma, allergy, and airway disease by an improved questionnaire that was derived from the International Study of Asthma and Allergies in Childhood (ISAAC). Results of questionnaire with respect to asthma were validated in a prior study. |
| Confounding | Low | Adjusted for age, sex, located district of residence, family history of atopy, ownership of the current residence (as a surrogate of SES), household environmental tobacco smoke (ETS), and household dampness-related exposures. |
| Incomplete outcome data | Low | There is no evidence of missing outcome data in the study subjects. |
| Exposure assessment | Probably low | Formaldehyde measurements were obtained via monitoring using validated and reliable measures and were obtained for short (6 hours) and long (24 hour) intervals. The instruments for on-site measurement and sample-collecting were checked every week, and were calibrated every month. No results of QA/QC were reported. |
| Selective outcome reporting | Low | All of the published manuscript's outcomes outlined in the methods, abstract, and/or introduction section that are of interest in the review have been reported in the specified way. |
| Conflict of interest | Low | This work is ﬁnancially supported by the National Natural Science Foundation of China (51278302), Hujiang Foundation of China (D14003), the Innovation Program of Shanghai Municipal Education Commission (14ZZ132), and the Innovation Fund Project for Graduate Student of Shanghai (JWCXSL1401). The funders had no role in study design, data collection and analysis, decision to publish, or preparation of the manuscript. |
| Other sources of bias | High | Of 454 households (186 cases and 268 controls), authors have formaldehyde outcome data for 410 (or 409 reported in Table 6) due to refusal of parents to do monitoring in some houses. The paper concludes that this missing data could affect results in the studied associations, although the proportion of children whose data were missed among cases and controls had no notable differences. There were also significant differences in the season sampled among cases and controls (cases sampled in summer when formaldehyde exposures were lower). |
