# Supplementary material for: Exposure to formaldehyde and asthma outcomes: A systematic review, meta-analysis, and economic assessment
Source: PLoS One. 2021 Mar 31;16(3):e0248258. doi: 10.1371/journal.pone.0248258 (PMC8011796; doi:10.1371/journal.pone.0248258)
Supplement: S39 Table — (DOCX) [file pone.0248258.s052.docx]

Supplemental Materials, Table 39. Characteristics of Hulin et al. 2010

| Bias domain | Authors’ judgment | Support for judgment |
| --- | --- | --- |
| Source population representation | Low | Nested case control study on an urban and a rural cohort. The urban sample was taken from the French Six Cities study cohort and the rural was taken from the FERMA study. The authors compare included cases and controls to their representative cohort and found no significant differences in terms of recorded characteristics. The authors noted a higher parental education among rural cases compared to non-participants. Figure 1 outlines how the final number of participants were selected. |
| Blinding | Probably low | Blinding was not addressed. It is unlikely that parents, who completed the symptom questionnaire, would have been aware of living room formaldehyde levels. Authors did not report whether the analytical chemist who performed the exposure analyses was blinded to participant outcome status. |
| Outcome assessment | Probably low | Asthmatic cases were identified on the basis of an affirmative response to one of the following questions Has your child ever had asthma?, Has your child had wheezing in the chest in the past 12 months? or whether children have already taken medications against asthma crises. Controls were composed of children who answered no to all three questions. Other symptoms were self-reported by parental questionnaire from ICAAS a previously validated form. |
| Confounding | Probably low | Potential confounders included age, sex, allergic rhinitis, parental (mothers and/or fathers) history of allergy, exposure to passive smoking during childhood (defined as any current exposure to cigarettes, pipes, or cigars at home, in utero or during the first year of life) and living area and season combined, school location (rural/urban). SES was not accounted for. |
| Incomplete outcome data | Probably low | 63 urban and 51 rural children were included in the final analyses (56 asthmatics and 58 controls). Sixteen children, nine from the rural area and seven from the urban area, were excluded from the analysis because of missing values. They did not differ significantly from the others in terms of recorded characteristics. Two were excluded because they were living in the same house as previously included cases, and seven because they had missing values on the questionnaire for evaluating confounding factors. Study rated probably low because of discrepancy in Table 3--there are 55 controls listed, but 56 are listed in previous tables, with no explanation for missing control in analysis. |
| Exposure assessment | Probably low | Formaldehyde was assessed continuously during 1 week in the living room of the houses of the 114 children using passive diffusion samplers. Pollutants were assessed during both summer and winter in the urban area and only in summer in the rural area. QC methods were not reported. Additional details have been published elsewhere. |
| Selective outcome reporting | Low | All of the study’s pre-specified (primary and secondary) outcomes outlined in the published manuscript’s methods, abstract, and/or introduction section that are of interest in the review have been reported in the pre-specified way. |
| Conflict of interest | Low | The authors were affiliated with academic institutions and the study was supported by the French government. |
| Other sources of bias | Low | The study appears to be free of other sources of bias. |
