# Supplementary material for: Exposure to formaldehyde and asthma outcomes: A systematic review, meta-analysis, and economic assessment
Source: PLoS One. 2021 Mar 31;16(3):e0248258. doi: 10.1371/journal.pone.0248258 (PMC8011796; doi:10.1371/journal.pone.0248258)
Supplement: S40 Table — (DOCX) [file pone.0248258.s053.docx]

Supplemental Materials, Table 40. Characteristics of Hwang et al. 2011

| Bias domain | Authors’ judgment | Support for judgment |
| --- | --- | --- |
| Source population representation | Probably low | The authors surveyed an elementary school in Seoul and identified children with self-reported or physician diagnosed cases. The control group includes children matched but age and gender, who had no asthma symptoms or an asthma diagnosis. The authors used volunteers to submit to personal, and indoor/outdoor stationary monitoring at their homes. Participation rates were low, with about 7% of those who answered the questionnaire and less than 17% of cases represented in the study. The authors evaluated SES and residential factors and determined no significant differences between the volunteer case and control groups, however there is no comparison of study group to all that responded to the survey. |
| Blinding | Probably low | There was insufficient information about blinding. Researchers visited homes to establish the stationary samples and distribute personal samplers. However, we do not know for sure if the outcome assessors were the same researchers, or blinded to the exposure. There is no information whether measures were taken to ensure blinding to case status. |
| Outcome assessment | Probably low | Asthma cases in children were a combination of defined by parent-reported asthma symptoms or parent-reported physician-diagnosed asthma using an ISAAC questionnaire, known as a previously validated tool. |
| Confounding | Low | Matched case control study, and the authors accounted for all Tier I confounders including passive smoke exposure, SES, and residential factors. The authors performed a statistical analysis and identified no other significant differences between cases and controls, hence there was no need to account for other known measured confounders. |
| Incomplete outcome data | Probably low | This case control study consisted of 33 cases and 40 controls which were matched. All students with measured exposure sampling were included. However, in tables 2 and 3, the numbers of cases and controls vary from the original group presented in table 1 (33 cases and 40 controls), but there was no explanation for what happened to the missing individuals. |
| Exposure assessment | Low | The authors employed two types of passive sampling 3M badges, one to measure VOCs and one to measure formaldehyde. The measurement was conducted at three points by subjects. There was no discussion of detection limits or regarding sampler capacity and the 3 day deployment being adequate. However the authors reference the NIOSH protocol, and if they followed the QA/QC requirements in the protocol, one would anticipate valid measures. All exposure assessment was done the same way for cases and controls. |
| Selective outcome reporting | Low | Results were presented for all the outcomes specified in the abstract and methods. |
| Conflict of interest | Low | The authors were academic and this was a government funded study. |
| Other sources of bias | Low | No other sources of bias identified. |
