# Supplementary material for: Exposure to formaldehyde and asthma outcomes: A systematic review, meta-analysis, and economic assessment
Source: PLoS One. 2021 Mar 31;16(3):e0248258. doi: 10.1371/journal.pone.0248258 (PMC8011796; doi:10.1371/journal.pone.0248258)
Supplement: S41 Table — (DOCX) [file pone.0248258.s054.docx]

Supplemental Materials, Table 41. Characteristics of Jacobsen et al. 2009*

| Bias domain | Authors’ judgment | Support for judgment |
| --- | --- | --- |
| Source population representation | Low | The baseline study population was identified in a cross-sectional study performed during 1997–1998. All factories in Viborg County with 20 employees were asked to participate (45 out of 48 accepted), and a random sample was drawn of factories with 5–20 employees (9 out of 38 accepted). A total of 54 factories participated in the study. The study population was workers employed in the woodworking, assembly and stock departments of these factories. Three factories (two producing refrigerators and one producing hearing aids) in the same area were selected as reference. Participation rate was 68% for wood-workers and 63% for reference workers. No information is provided on whether those lost to follow-up were representative of the study participants overall. |
| Blinding | Probably high | There is no evidence of blinding. Due to their occupation, participants were likely aware of their exposure status. In addition to self-reporting of symptoms, participants were asked about asthma diagnosed by a doctor, asthma symptoms at baseline were validated, and lung function tests were conducted. |
| Outcome assessment | Low | Asthma symptoms as baseline were validated, and lung function testing was performed at baseline and follow-up. Reference is provided to a detailed description of the lung function testing. Respiratory symptoms were self-reported using a modified UK Medical Research Council questionnaire. |
| Confounding | Low | The analyses were adjusted for smoking (Tier I), age, and baseline hay fever, and were stratified by sex (Tier II). The analyses were adjusted for smoking (Tier I), age, and baseline hay fever, and were stratified by sex (Tier II). Did not adjust for SES explicitly, but cases and controls had similar job functions so it would not be unreasonable to assume that SES status was similar. |
| Incomplete outcome data | Low | Authors provide a clear diagram indicating subjects lost to follow-up. |
| Exposure assessment | Probably high | A personal passive SKC UMEx 100 diffusion sampler (SKC, Inc.) was used to collect 24 formaldehyde measurements in 10 factories, using a worst case strategy. It is not clear how authors defined the "worst case" strategy. Low levels of formaldehyde were identified overall. However this could be a function of biased sampling, as 44 of 54 factories were not sampled. |
| Selective outcome reporting | Low | Results were reported for all outcomes specified in the abstract and methods. |
| Conflict of interest | Low | The study was funded by government and non-profit organizations (Viborg County, Denmark, the Danish Work Environment Foundation, the Danish Medical Research Council, the Health Insurance Fund, and the Danish Lung Association). The authors were affiliated with academic institutions and state they have no competing financial interests. |
| Other sources of bias | Probably high | Subjects were employed in a woodworking factory and controls in refrigerator and hearing aid factories. While individuals were included with asthma, some of the most affected could have left the job prior to the study taking place, thus introducing a healthy worker bias, which would likely bias the results towards the null. |

* Additional information was provided from the study authors that was considered in the risk of bias evaluation
