# Supplementary material for: Exposure to formaldehyde and asthma outcomes: A systematic review, meta-analysis, and economic assessment
Source: PLoS One. 2021 Mar 31;16(3):e0248258. doi: 10.1371/journal.pone.0248258 (PMC8011796; doi:10.1371/journal.pone.0248258)
Supplement: S42 Table — (DOCX) [file pone.0248258.s055.docx]

Supplemental Materials, Table 42. Characteristics of Jeong et al. 2011

| Bias domain | Authors’ judgment | Support for judgment |
| --- | --- | --- |
| Source population representation | Probably low | Second grade students from 56 elementary schools (11 schools in an industrial region and 45 schools in a non-industrial region) were enrolled in this study. Written informed consent was provided by parents or guardians for all subjects and a written questionnaire was completed for all students by their parent or guardian (1315 in the industrial region and 1829 in the non-industrial region). 89 students in the industrial region and 81 students in the non-industrial region were excluded because of insufficient completion. The children were comparable in anthropometric measurements. |
| Blinding | Probably low | Blinding is not addressed, but questionnaire was completed by parents who were unlikely to be aware of environmental exposures to the extent that this would bias their responses regarding outcomes. |
| Outcome assessment | Probably low | Outcomes were measured by parental report via questionnaire and skin prick tests. A modified version of the International Study of Asthma Allergies in Children (ISAAC) was used. |
| Confounding | Probably high | Study groups were not statistically different in sex, height, and weight. Study schools where in industrial and non-industrial areas (proxies for SES), and parental history of asthma or AR, and age were also reported. However, analyses were t-tests and hi-squared tests. |
| Incomplete outcome data | Low | There were 89 individuals in the industrial region and 81 individuals in the non-industrial region who were excluded because of insufficient completion. This missing data is unlikely to be related to the true outcome. |
| Exposure assessment | Probably low | A single exposure measurement was made in 11 schools from the industrial region, selected evenly according to pre-examined PM levels. A single measurement was made in 2 schools from the non-industrial region, one from an urban area and one from a rural area. Formaldehyde was measured inside and outside using a Formaldehyde Meter w/Pump Model Z-300XP. No QA/QC methods were described. |
| Selective outcome reporting | Low | Results were reported for all outcomes specified in the abstract and methods. |
| Conflict of interest | Low | This study was supported by the government and an academic institution. All authors were affiliated with academic institutions, and there is no reason to believe that a conflict of interest exists. |
| Other sources of bias | Low | No other threats to internal validity were identified. |
