# Supplementary material for: Exposure to formaldehyde and asthma outcomes: A systematic review, meta-analysis, and economic assessment
Source: PLoS One. 2021 Mar 31;16(3):e0248258. doi: 10.1371/journal.pone.0248258 (PMC8011796; doi:10.1371/journal.pone.0248258)
Supplement: S43 Table — (DOCX) [file pone.0248258.s056.docx]

Supplemental Materials, Table 43. Characteristics of Kilburn et al. 1985

| Bias domain | Authors’ judgment | Support for judgment |
| --- | --- | --- |
| Source population representation | Probably high | Recruitment methods are not reported in detail. Of a crew of 110 male workers, 45 volunteered to participate. 18 male histology technicians were also included in the exposed group. Other hospital employees (26) made up the control group. Inclusion/exclusion criteria are not reported. |
| Blinding | Probably high | Blinding is not addressed. Workers likely unaware of status since both were occupationally exposed to formaldehyde (at different levels). However, no mention of whether investigators were blinded and the measurement of lung function could be influenced by investigator's knowledge of exposure status. |
| Outcome assessment | Low | Lung function measured were recorded using spirometry performed using a waterless volume displacement spirometer with a recorder. Methods are described in detail and are adequate. Histology technicians had peak flow measured during work, while batt makers and controls were measured before and after work. |
| Confounding | Low | Subjects were age, smoking and ethnicity matched. Some analyses are pre- post-shift comparisons for batt workers, stratified by smoking status. SES is not considered; however the authors compared employees before and after shifts, so this essentially 'controls' for the SES effect. |
| Incomplete outcome data | Low | No missing outcome data reported. |
| Exposure assessment | Probably high | Exposure assessed by self-administered, proctored questionnaire; analyses incorporated work assignment as proxy for high and low exposure groups. No formaldehyde measurements were performed. |
| Selective outcome reporting | Low | Results were reported for outcomes as specified. |
| Conflict of interest | Probably low | There is no information on the source of funding. The authors' affiliations are academic. There is no reason to expect potential conflict of interest. |
| Other sources of bias | Probably high | Subjects were volunteer fiberglass batt makers, histology technicians, and controls were hospital employees. While individuals were included with asthma, some of the most affected could have left the job prior to the study taking place, thus introducing a healthy worker bias, which would likely bias the results towards the null. |
