# Supplementary material for: Exposure to formaldehyde and asthma outcomes: A systematic review, meta-analysis, and economic assessment
Source: PLoS One. 2021 Mar 31;16(3):e0248258. doi: 10.1371/journal.pone.0248258 (PMC8011796; doi:10.1371/journal.pone.0248258)
Supplement: S44 Table — (DOCX) [file pone.0248258.s057.docx]

Supplemental Materials, Table 44. Characteristics of Kilburn, Seidman, and Warshaw 1985

| Bias domain | Authors’ judgment | Support for judgment |
| --- | --- | --- |
| Source population representation | Probably low | The study group consisted of 76 female histology technicians in the Los Angeles area, and the reference group consisted of 56 female clerical worker in the same institutions with no workplace exposure to formaldehyde. The reference group was selected to match at least 40 technicians with respect to age, cigarette smoking, and ethnicity. This is likely to limit the potential for self-selection bias among histology technicians concerned that solvent exposure may be associated with health effects. Authors do not indicate why the control group is smaller than the exposed group. Participation rates are not reported. |
| Blinding | Probably high | There is no evidence of blinding, and participants were likely aware of their exposure status. |
| Outcome assessment | Probably low | Symptoms were self-reported using a pre-tested questionnaire. No additional outcome assessment methods were described. Rated as probably low risk of bias because asthma diagnosis confirmed by medical history, not objective testing. |
| Confounding | Low | There were 43 matched pairs with respect to age, cigarette smoking, and ethnicity. Exposed and unexposed participants had different job functions, but worked for the same organization. SES was not considered; however, authors selected exposed and control groups that were from similar SES (technicians compared to secretaries and clerks) and it would not be unreasonable to assume that SES status was similar. |
| Incomplete outcome data | Low | There was no missing data. |
| Exposure assessment | Probably low | Area sampling for formaldehyde was performed by the regional NIOSH laboratory using sorbent tubes, and analyzed by gas chromatography. Sampling for 1-4 hr was done in 10 of the 25 participating laboratories, each of which had three or more technicians in the study. Environmental sampling was not conducted in each laboratory and was conducted during a limited time period compared to actual potential exposure of participants. It is unclear if these 10 laboratories were representative of overall exposure. No QA/QC methods were described. Number of hours of formaldehyde exposure was self-reported. |
| Selective outcome reporting | Low | Results were reported for all outcomes specified in the abstract and methods. |
| Conflict of interest | Probably low | No funding information is provided. All authors are affiliated with academic institutions, and there is no reason to believe that a conflict of interest exists. |
| Other sources of bias | Probably high | Subjects were individuals working as histology technicians and controls were women working as secretaries or clerks at the same institutions with no workplace exposure to formaldehyde. While asthmatics were included, some of the most affected could have left the job prior to the study taking place, thus introducing a healthy worker bias, which would likely bias the results towards the null. |
