# Supplementary material for: Exposure to formaldehyde and asthma outcomes: A systematic review, meta-analysis, and economic assessment
Source: PLoS One. 2021 Mar 31;16(3):e0248258. doi: 10.1371/journal.pone.0248258 (PMC8011796; doi:10.1371/journal.pone.0248258)
Supplement: S45 Table — (DOCX) [file pone.0248258.s058.docx]

Supplemental Materials, Table 45. Characteristics of Kim et al. 2007

| Bias domain | Authors’ judgment | Support for judgment |
| --- | --- | --- |
| Source population representation | Probably low | All primary schools within the Knivsta Municipality, near Uppsala, Sweden, participated in this study. All students were invited to participate, and 68% (1014/1482) responded to the survey. The characteristics of the population are described, but not in detail. |
| Blinding | Probably low | The authors do not mention any blinding. However, it is unlikely that participants or their parents were aware of their exposure status. It is also unlikely that the environmental engineers performing the sampling and the lab analyzing the samples were aware of outcomes. |
| Outcome assessment | Probably low | Asthma and respiratory symptoms were self-reported, and questionnaires were completed cooperatively by participants and a parent. The survey included questions on doctor-diagnosed asthma, asthma medications, and symptoms, but none of this information was confirmed through medical records. Participants were defined as asthmatic if they were either currently using asthma medication or had an asthma attack in the last 12 months. Another set of questions was used to assess validity by asking about asthma symptoms without using the word "asthma". |
| Confounding | High | The study did not account for Tier I confounders, but adjusted for age and gender (Tier II). The survey lacked questions with regard to parental history of asthma and environmental tobacco smoke exposure. |
| Incomplete outcome data | Probably low | The survey response rate was 68% (1014/1482), but there was no other apparent missing data. |
| Exposure assessment | Probably low | Passive samplers were placed in three randomly selected classrooms and one passive sampler was placed outside for each school. The samplers had an air sampling flow of 0.2 l/min during a 6 hour sampling period. Filters were analyzed by liquid chromatography and had a limit of detection of 6 ug/m3. No additional QA/QC methods were described. |
| Selective outcome reporting | Low | Results are reported for all outcomes specified in the abstract and methods. |
| Conflict of interest | Probably low | The authors were affiliated with academic institutions and there is no reason to believe that a conflict of interest exists. Funding sources were not specified. |
| Other sources of bias | Low | No other threats to validity were identified. |
