# Supplementary material for: Exposure to formaldehyde and asthma outcomes: A systematic review, meta-analysis, and economic assessment
Source: PLoS One. 2021 Mar 31;16(3):e0248258. doi: 10.1371/journal.pone.0248258 (PMC8011796; doi:10.1371/journal.pone.0248258)
Supplement: S46 Table — (DOCX) [file pone.0248258.s059.docx]

Supplemental Materials, Table 46. Characteristics of Kim et al. 2011

| Bias domain | Authors’ judgment | Support for judgment |
| --- | --- | --- |
| Source population representation | Probably low | Schools were randomly selected and all 4th graders were invited to participate. Characteristics of the study population were not presented, but several factors were adjusted for in the model. 96% of invited students participated, and subjects for whom key study factors were missing were excluded (450/2365). The response rate among pupils include did not differ from the total population. No information on the potential differences between students in classrooms with measured exposures and those without. |
| Blinding | Probably low | There is no evidence of blinding, but there is no reason to believe that bias was introduced. Outcomes were self-reported and students were unlikely to be aware of their exposure status, and environmental samples were analyzed by an independent, accredited laboratory. |
| Outcome assessment | Probably low | Asthma was assessed through a questionnaire as either having doctor-diagnosed asthma, or current medication and/or asthma attacks during the last 12 months, and wheezing or whistling in the chest during the last 12 months. It is unclear whether parents helped in the completion of the questionnaire, and diagnoses were not confirmed against medical record. |
| Confounding | Probably low | Potential confounders considered included age, sex, self-reported furry pet or pollen allergy, ETS, and selected home environmental. SES was not considered. |
| Incomplete outcome data | Low | 96% (2365/2453) of invited students participated, and about 20% (450/2365) of subjects were excluded because key study factors were missing for them. However, outcome data were reported on all subjects that were actually included. |
| Exposure assessment | Probably low | Indoor and outdoor sampling were performed for a continuous 7-day period using diffusive samplers. Indoor samplers were placed about 1.5 m above the floor in selected classrooms, and outdoor samplers were placed in a plastic box outside classroom windows. It is unclear if the plastic box completely enclosed the sampler and potentially interfered with measurements. Samples were analyzed by an accredited laboratory. No QA/QC details were provided. |
| Selective outcome reporting | Low | Results were presented for all the relevant outcomes specified. |
| Conflict of interest | Probably low | All researchers were affiliated with a university. Though there is no reason to believe that a conflict of interest exists, the authors do not provide any information on the source of funding for the study. |
| Other sources of bias | Low | No other sources of bias identified. |
