# Supplementary material for: Exposure to formaldehyde and asthma outcomes: A systematic review, meta-analysis, and economic assessment
Source: PLoS One. 2021 Mar 31;16(3):e0248258. doi: 10.1371/journal.pone.0248258 (PMC8011796; doi:10.1371/journal.pone.0248258)
Supplement: S47 Table — (DOCX) [file pone.0248258.s060.docx]

Supplemental Materials, Table 47. Characteristics of Kim et al. 2014

| Bias domain | Authors’ judgment | Support for judgment |
| --- | --- | --- |
| Source population representation | Probably low | Participants were identified at a medical college as patients who had been definitely diagnosed as asthmatic. Participation rates were not reported. The participants were primarily housewives who spent most of their daily lives indoors in their own household. The participants were divided into two groups for the two experimental seasons; 9 households had plants indoors for two observation terms, and 8 households had plants indoors for the first observation term and the plants were removed for the second observation term. |
| Blinding | Probably high | Blinding was not discussed, and participants were aware of which study group they were in. Lung function measurements could have been influenced by either participant or investigator's knowledge of their exposure status. |
| Outcome assessment | Probably low | All participants used peak flow meters twice a day for seven days before and after the observation terms. Symptom severity was self-reported using the quality of life questionnaire for adult Korean asthmatics. |
| Confounding | Probably high | Demographic information was presented for participant gender, age, area/size of residents, and year of building completion (SES proxies). In attempt to prevent any confounding effects due to occupation, most participants were housewives. |
| Incomplete outcome data | Low | There is no apparent missing data. |
| Exposure assessment | Low | Air capture was conducted using airtight conditions following US EPA guidance. A personal air sampler with a low-volume vacuum pump was used in the living room for 60 minutes at a flow rate of 0.1 L/min. Formaldehyde was analyzed by HPLC. The procedure was replicated five times. The desorbing efficiency for the target substances was maintained in the range of 85-115% |
| Selective outcome reporting | Low | Results were reported for all outcomes specified in the abstract and methods. |
| Conflict of interest | Low | The authors state that they have no conflict of interest, and the study was funded by the government. |
| Other sources of bias | Low | No other threats to internal validity were identified. |
