# Supplementary material for: Exposure to formaldehyde and asthma outcomes: A systematic review, meta-analysis, and economic assessment
Source: PLoS One. 2021 Mar 31;16(3):e0248258. doi: 10.1371/journal.pone.0248258 (PMC8011796; doi:10.1371/journal.pone.0248258)
Supplement: S48 Table — (DOCX) [file pone.0248258.s061.docx]

Supplemental Materials, Table 48. Characteristics of Kriebel et al. 1993

| Bias domain | Authors’ judgment | Support for judgment |
| --- | --- | --- |
| Source population representation | Low | This study included 24 physical therapy students out of 25 students who were part of a laboratory class dissecting cadavers for 3 hours per week. |
| Blinding | Probably high | Blinding is not reported. Each participant was measured before and after lab exposure. Participants and investigators may have thus known exposure status, in which case measurement of lung function measures could potentially be biased. |
| Outcome assessment | Low | Students self-reported symptoms using a questionnaire. Students also measured PEF at the beginning and end of each laboratory under the supervision on an investigator. Subjects also conducted 1-3 PEV tests during the class. PEV was measured using Mini-Wright peak flowmeters (Armstrong Medical Industries, Lincolnshire, IL). Each student was trained to use the peak flowtometer. The average of three blows was used. |
| Confounding | Low | Age, gender, smoking status, and asthma history were assessed. SES is not specifically considered, however can assume that education is a proxy for SES (which is a reasonable assumption), then given that all the participants were graduate students, then the confounder for SES is accounted for in the participant selection. |
| Incomplete outcome data | Low | Results are complete for 24 participants. A total of 1,496 peak flow measurements were made, with missing data due to absences. In all but 6% of these sessions, subjects produced reproducible PEF measurements; these data were not treated differently |
| Exposure assessment | Probably low | Breathing zone air samples were analyzed for formaldehyde using both the NIOSH 2541 method and the NIOSH 3500 method, sampling for 1 to 1.5 hours. No information provided on QA/QC. |
| Selective outcome reporting | Low | Results were reported for all outcomes specified in the abstract and methods. |
| Conflict of interest | Low | This study was funded by the government and all the authors were affiliated with an academic institution. |
| Other sources of bias | Probably low | The authors note the potential for the healthy worker selection effect and that there may have been an initial period of responsiveness to symptoms and that over time, students may have adapted to the irritant effects. If a healthy worker bias exists, it would likely bias the results towards the null |
