# Supplementary material for: Exposure to formaldehyde and asthma outcomes: A systematic review, meta-analysis, and economic assessment
Source: PLoS One. 2021 Mar 31;16(3):e0248258. doi: 10.1371/journal.pone.0248258 (PMC8011796; doi:10.1371/journal.pone.0248258)
Supplement: S49 Table — (DOCX) [file pone.0248258.s062.docx]

Supplemental Materials, Table 49. Characteristics of Kriebel et al. 2001*

| Bias domain | Authors’ judgment | Support for judgment |
| --- | --- | --- |
| Source population representation | Probably low | A class of 54 physical therapy graduate students exposed to formaldehyde when working on human cadavers were invited to participate. 51 (94%) agreed to participate and data were obtained for 38/51 participants (75%). |
| Blinding | Probably high | There is no evidence of blinding. Pulmonary function tests were performed by technicians. There is no way to tell they were not aware of each participant's exposure level. All participants were exposed to formaldehyde, but it is unlikely that they were aware of their exposure level. |
| Outcome assessment | Low | Pulmonary function tests were performed by NIOSH-certified technicians using spirometers that were calibrated twice daily. Each subject performed a minimum of 5 blows that met the standards of the American Thoracic Society. Each subject was trained individually in the correct use of a peak flowmeter, and was instructed to record five blows at the beginning and end of each laboratory session. Additional symptoms were self-reported using a questionnaire. |
| Confounding | Low | Of the 38 participants, there were 2 current smokers and 5 ex-smokers; there was no evidence that these participants had different responses to formaldehyde than non-smokers, so smoking status was not included in the analysis. Gender, age, and height were also reported, but the multivariate models did not account for them or for SES. However, authors used a longitudinal analysis, so evaluated pre/post exposures, which does not require controlling for individual factors, though authors noted that they evaluated the effect of time on the analysis. |
| Incomplete outcome data | Low | Authors noted substantial absenteeism resulting in loss of outcome data during the latter half of the semester, however sensitivity analyses indicated minimal effects upon coefficients, and thus study was rated low risk of bias. |
| Exposure assessment | Probably low | Formaldehyde was monitored continuously at six locations, representing previously identified homogeneous exposure zones in the laboratory, using a direct-reading instrument equipped with a multiple sampling site switching station and a data logger. The detection limit was 0.05 ppm formaldehyde. The locations of each subject were matched with the exposure zone and the arithmetic mean of exposure for each participant was calculated weekly. Minimal QA/QC information available. |
| Selective outcome reporting | Low | Results are reported for all outcomes specified in the abstract and methods. |
| Conflict of interest | Low | All authors are affiliated with either academic or governmental institutions, and was partially funded by NIEHS. |
| Other sources of bias | Low | No other sources of bias identified. |

* Additional information was provided from the study authors that was considered in the risk of bias evaluation
