# Supplementary material for: Exposure to formaldehyde and asthma outcomes: A systematic review, meta-analysis, and economic assessment
Source: PLoS One. 2021 Mar 31;16(3):e0248258. doi: 10.1371/journal.pone.0248258 (PMC8011796; doi:10.1371/journal.pone.0248258)
Supplement: S50 Table — (DOCX) [file pone.0248258.s063.docx]

Supplemental Materials, Table 50. Characteristics of Krzyzanowski et al. 1990

| Bias domain | Authors’ judgment | Support for judgment |
| --- | --- | --- |
| Source population representation | Probably low | Recruitment based upon stratified, population-based sample; eligible households with children 5-15 years of age, no information on participation/loss to follow-up over two weeks of observation. |
| Blinding | Probably low | No mention of blinding of study personnel or participants; however, asthma assessed via self-administered questionnaire, and exposure assessed in subjects' homes. |
| Outcome assessment | Probably high | The authors used self-administered sprirometry to measure PEFR using a mini-wright portable spirometer and daily diaries to evaluate chronic respiratory symptoms, including asthma; The authors took steps to ensure training of participants and removed the final two days of results due to a measured learning effect. |
| Confounding | Low | All of Tier I (tobacco smoking (active and passive), SES), and most of Tier II confounders (indoor NO2) were accounted for. |
| Incomplete outcome data | Low | The authors indicate that the homes used in the study group had complete questionnaires available (298 children and 613 adults). Outcome data appear to be complete. |
| Exposure assessment | Probably low | The authors used passive samplers to measure formaldehyde levels in multiple areas of the home for two 1 week periods. These devices were prepared in the lab by the researchers then calibrated and compared to a commercial product to confirm consistency of measurement. Humidity was likely not an issue in Tucson, AZ. Minimal QA/QC information provided. |
| Selective outcome reporting | Low | Results were presented for all the relevant outcomes specified. |
| Conflict of interest | Low | The authors were academic and supported by EPA contracts and NIEHS fellowship support. |
| Other sources of bias | Low | No other sources of bias identified. |
