# Supplementary material for: Exposure to formaldehyde and asthma outcomes: A systematic review, meta-analysis, and economic assessment
Source: PLoS One. 2021 Mar 31;16(3):e0248258. doi: 10.1371/journal.pone.0248258 (PMC8011796; doi:10.1371/journal.pone.0248258)
Supplement: S51 Table — (DOCX) [file pone.0248258.s064.docx]

Supplemental Materials, Table 51. Characteristics of Lajoie et al. 2015

| Bias domain | Authors’ judgment | Support for judgment |
| --- | --- | --- |
| Source population representation | Low | Randomized controlled trial with well-documented inclusion/exclusion criteria; authors noted only one participant abandoned study after enrollment; randomization was performed by study statistician independently of the researchers and physicians; participants allocated using a personal identification code for each participant. |
| Blinding | Probably low | Authors note that study was unblinded. However there was random assignment to ventilation intervention and environmental and clinical data were captured blind by two research assistants. |
| Outcome assessment | Probably low | The baseline health evaluation consisted of sing ISAAC questionnaire completed by parents. Asthma was reported by parent report using French Canadian version of core questionnaire specifically for 6-/7-year olds. During the study, parents documented daily symptoms and asthma-related medications in a diary. Nurses trained parents on the use of the peak flow meter, and peak expiratory flow rate was measured twice daily at home. Respiratory function tests were conducted in a clinic. |
| Confounding | Low | Most of Tier I and some of Tier II confounders were accounted for, including age and parents' level of education, gender and eczema history, presence of attached garage or woodstove. Inhaled corticosteroid therapy was noted to be controlled by randomization and no significant difference was noted in medication use between intervention and control groups at baseline. Children in the study were randomized into the intervention and control group and the authors adjusted for confounders which supports the low risk of bias rating. Authors did not adjust for smoking, but children were randomized into intervention groups. |
| Incomplete outcome data | Low | Authors noted only one person was not included in analyses due to abandonment of study during year 2. No other missing outcome data reported. |
| Exposure assessment | Probably low | Duplicate passive samplers were used over a 6-8 day period in the child's bedroom to measure formaldehyde. The cartridges were subsequently analyzed for formaldehyde using high-performance liquid chromatography (GC) according to the ASTM test method D. No detail on QA/QC or LODs are provided. |
| Selective outcome reporting | Low | Results were presented for all the relevant outcomes specified. |
| Conflict of interest | Low | Funding for this study was provided by government organizations and the authors declare that they had no competing financial interests. |
| Other sources of bias | Low | No other sources of bias identified. |
