# Supplementary material for: Exposure to formaldehyde and asthma outcomes: A systematic review, meta-analysis, and economic assessment
Source: PLoS One. 2021 Mar 31;16(3):e0248258. doi: 10.1371/journal.pone.0248258 (PMC8011796; doi:10.1371/journal.pone.0248258)
Supplement: S52 Table — (DOCX) [file pone.0248258.s065.docx]

Supplemental Materials, Table 52. Characteristics of Liu et al. 1991

| Bias domain | Authors’ judgment | Support for judgment |
| --- | --- | --- |
| Source population representation | Low | Sampling was done by age-stratified sampling starting with a list of "randomly" selected mobile homes provided by the CA Department of Housing and Community Development and encompasses a large population. 44% positive response rate and authors note that the age distribution is similar for those who participated and those who did not. Similar demographics comparing those participants who participated in summer and winter seasons. Authors describe process in reasonable detail and provide demographic information. Large sample size included (1000 individuals). |
| Blinding | Low | Randomly selected population, all of whom live in mobile homes. Formaldehyde measurements were handled by survey participants by placing passive monitors in their homes, which were returned with survey responses at the same time. |
| Outcome assessment | Probably high | Outcomes obtained from self-reported symptoms in a questionnaire. No mention of physician confirmation and no in-person interview by study investigators or mention of any follow up through phone, etc. No information provided on the validity of the questionnaires. |
| Confounding | Probably high | Authors controlled for age, sex, and smoking status among the Tier I confounders but not SES. Authors additionally controlled for presence of chronic respiratory/allergy conditions. No Tier II confounders addressed. |
| Incomplete outcome data | Low | No missing outcome data--authors include outcome data for all participants who returned questionnaire. |
| Exposure assessment | Probably low | Passive HCHO monitors were mailed to participants with instructions to uncap monitors and place one in kitchen and one in bedroom. After 7 days of exposure, monitors (along with questionnaires) were mailed back. HCHO concentrations were measured using the chromatrophic acid method. No discussion on QA/QC of measurement methods and there is potential for inaccurate measurements because participants perform for themselves and mail back. |
| Selective outcome reporting | Low | Authors report results for all outcomes outlined in methods section. |
| Conflict of interest | Low | No COI statement included in manuscript, but all authors are affiliated with government (CA Department of Health Services, US EPA). |
| Other sources of bias | Low | No other sources of bias identified. |
