# Supplementary material for: Exposure to formaldehyde and asthma outcomes: A systematic review, meta-analysis, and economic assessment
Source: PLoS One. 2021 Mar 31;16(3):e0248258. doi: 10.1371/journal.pone.0248258 (PMC8011796; doi:10.1371/journal.pone.0248258)
Supplement: S53 Table — (DOCX) [file pone.0248258.s066.docx]

Supplemental Materials, Table 53. Characteristics of Lofstedt et al. 2009*

| Bias domain | Authors’ judgment | Support for judgment |
| --- | --- | --- |
| Source population representation | Probably low | Potentially exposed and unexposed individuals from foundries using the Hot Box method were recruited with assistance from company and occupational health service officials. Ten exposed individuals (14%) declined to participate. The referents were matched by gender and age, but a higher proportion (59 individuals, 31%) declined to participate. A table of characteristics is provided. There was no information provided to determine if participants were similar to those not included. |
| Blinding | Probably high | There is no evidence of blinding. The participants were informed about the aim of the study. Participants were likely aware of their exposure status and outcomes were assessed by questionnaire as well as spirometry measures. |
| Outcome assessment | Low | Lung function tests were performed using a spirometer following the American Thoracic Society's guidelines. Measurements were made before and after a day shift, and were performed in the exposed group after at least 2 days without occupational exposure. Health status and respiratory symptoms were self-reported in a questionnaire. Study rated low risk of bias because objective measures were used (bronchial provocation tests) to determine outcomes. |
| Confounding | Low | Models were adjusted for smoking. Additional characteristics, including BMI and time in present job were explored. Time in present job did not differ between the two groups. There were equal proportion of females in exposed and referent groups. Co-pollutants are also accounted for. Authors did not adjust for SES, but all workers are in the same occupation so it would not be unreasonable to assume that SES status was similar. |
| Incomplete outcome data | Low | There is no missing data in the study participants. |
| Exposure assessment | Probably high | Exposure was measured in the exposed group for an 8 hour period. Reference is provided for another study describing the details of the exposure measurements. Referents' were assumed to be unexposed, and measurements of oil mist were used to support this assumption. Details of the measurement are not provided. |
| Selective outcome reporting | Low | All of the study’s pre-specified (primary and secondary) outcomes outlined in the published manuscript’s methods, abstract, and/or introduction section that are of interest in the review have been reported in the pre-specified way. |
| Conflict of interest | Low | The study was funded by a governmental agency and all authors were affiliated with academic institutions. There is no reason to believe a conflict of interest exists. |
| Other sources of bias | Probably high | Subjects were 74 individuals working in foundry using Hot Box method and controls were employees working outside the halls for core production and die casting. While asthmatics were included, some of the most affected could have left the job prior to the study taking place, thus introducing a healthy worker bias, which would likely bias the results towards the null. |

* Additional information was provided from the study authors that was considered in the risk of bias evaluation
