# Supplementary material for: Exposure to formaldehyde and asthma outcomes: A systematic review, meta-analysis, and economic assessment
Source: PLoS One. 2021 Mar 31;16(3):e0248258. doi: 10.1371/journal.pone.0248258 (PMC8011796; doi:10.1371/journal.pone.0248258)
Supplement: S54 Table — (DOCX) [file pone.0248258.s067.docx]

Supplemental Materials, Table 54. Characteristics of Lofstedt et al. 2011

| Bias domain | Authors’ judgment | Support for judgment |
| --- | --- | --- |
| Source population representation | Probably low | Specific methods were not reported as this was a 4-year follow-up of a previous study on workers. Workers using the Hot Box method were considered exposed. Referents were working with assembling, polishing, surface coating, and product testing in areas well separated from the foundries. One of the 4 original foundries refused to participate. 14 exposed workers and 25 referents were unavailable because of change in work task. One worker from each group chose not to participate. There was no information provided to determine if those participating were similar to those not. There were some differences between the exposed and unexposed groups, but the only significant one was allergic symptoms in childhood. |
| Blinding | Probably high | No information is provided on blinding. Since this is a follow-up study, the subjects were likely aware of the exposure/referent status. The subjects would not have been blind to the potential exposure, but it is possible that the people recording the lung function results may only have known the foundry and not the worker status. However, the lung function measures could have potentially been biased by knowledge regarding exposures. |
| Outcome assessment | Low | Outcomes were assessed and defined consistently across all study participants using valid and reliable methods. Symptoms were self reported. Lung function was investigated using a dry-wedge bellows spirometer according to the American Thoracic Society guidelines. Study rated as low risk of bias because objective measures (pulmonary function tests) were used to determine outcome. |
| Confounding | Low | Smoking was addressed by stratifying the results by smokers and nonsmokers. The characteristics of the exposed workers and the referents were broadly similar (similar proportions of women, smokers, and participants with self-reported asthma and positive Phadiatop (an indicator of atopy) except that there was a significantly lower frequency of childhood allergy in the exposed workers (4% vs 31%,respectively), and this difference was not further addressed. In addition, there may have been additional co-exposures that may have been different between the exposed and referent groups that were not addressed. Authors did not adjust for SES, but all workers are in the same occupation so it would not be unreasonable to assume that SES status was similar. |
| Incomplete outcome data | Low | There is little missing data (4 referents out of 51). The authors describe in detail the reasons for fewer subjects in the follow-up study. |
| Exposure assessment | Probably low | Formaldehyde was determined with diffusive samplers (GMD) as full-shift samples. The analysis of formaldehyde was performed with high performance liquid chromatography techniques, the corresponding analysis of monoisocyanates and diisocyanates with liquid chromatography mass spectrometry techniques (Karlsson et al., 1998a). The detection concentration level for formaldehyde during an 8 h sampling is 20 mg/m3, and for methyl isocyanate and ICA 4 mg/m3 for 15 min short term sampling. Minimal QA/QC information provided. |
| Selective outcome reporting | Low | All outcomes outlined in abstract, introduction and methods sections are reported. |
| Conflict of interest | Low | The study did not receive support from a company, study author, or other entity having a financial interest in the outcome of the study. The study was supported by government agencies and the authors were from the university. |
| Other sources of bias | Probably high | Subjects were individuals working in Swedish brass foundries. The study involved 4 years of follow up, during which time 3 exposed and 1 referent were unavailable for evaluation because they had left due to health problems. Therefore, although asthmatics were included, it is possible that one or more of the workers that left the job could have done so because of severe asthma symptoms, which would introduce a healthy worker bias, which would likely bias the results towards the null. |
