# Supplementary material for: Exposure to formaldehyde and asthma outcomes: A systematic review, meta-analysis, and economic assessment
Source: PLoS One. 2021 Mar 31;16(3):e0248258. doi: 10.1371/journal.pone.0248258 (PMC8011796; doi:10.1371/journal.pone.0248258)
Supplement: S55 Table — (DOCX) [file pone.0248258.s068.docx]

Supplemental Materials, Table 55. Characteristics of Low et al. 1985

| Bias domain | Authors’ judgment | Support for judgment |
| --- | --- | --- |
| Source population representation | Probably low | The authors surveyed a foundry of 114 workers with 49 workers employed in work areas where exposure was a concern. 46 of these workers were present on the day of the study, and authors decided to sample 19 at random. Only 2 individuals refused to participate. Methods used to choose the subsample were not reported. |
| Blinding | Probably high | The authors performed a modified MRC questionnaire on each worker and lung function assessment prior to sampling exposure in the work areas. No other information was provided to assess blinding of research staff. Lung function measures could potentially be biased with knowledge regarding exposures. |
| Outcome assessment | Low | The best of three forced expiratory maneuvers were measured with Vitalograph dry spirometer and calculated FEV1, FVC, and MMEF were capture at the end of shifts on Monday and Friday. No other details were provided to ascertain data quality. Self-reported respiratory symptoms were identified by questionnaire based on MRC questionnaire administered prior to lung function assessment. |
| Confounding | Low | The authors noted some differences in age between exposure groups. However, cigarette smoking was similar in all groups. However, analysis performed to compare workers in a particular setting over time, so each worker group (e.g. General Foundry) serve as their own controls. |
| Incomplete outcome data | Low | No missing outcome data reported. |
| Exposure assessment | Probably high | The authors used chromotropic acid absoprtion spectrometer and Drager tubes to measure formaldehyde levels in the work areas. However they did not discuss limits of detection, the use of quality control samples, or the duration and number of sampling events. Authors used work area as a surrogate for various exposures, but workers often worked multiple areas during a given shift. |
| Selective outcome reporting | Low | Results were presented for all the relevant outcomes specified. |
| Conflict of interest | Probably low | Information on study funding was not provided, but authors were university affiliated. |
| Other sources of bias | Probably high | Subjects were workers employed in the core shop and general foundry. While asthmatics were included, some of the most affected could have left the job prior to the study taking place, thus introducing a healthy worker bias, which would likely bias the results towards the null. |
