# Supplementary material for: Exposure to formaldehyde and asthma outcomes: A systematic review, meta-analysis, and economic assessment
Source: PLoS One. 2021 Mar 31;16(3):e0248258. doi: 10.1371/journal.pone.0248258 (PMC8011796; doi:10.1371/journal.pone.0248258)
Supplement: S56 Table — (DOCX) [file pone.0248258.s069.docx]

Supplemental Materials, Table 56. Characteristics of Madureira et al. 2015

| Bias domain | Authors’ judgment | Support for judgment |
| --- | --- | --- |
| Source population representation | Probably low | This is a cross-sectional study of a large sample of school and schoolchildren in Porto, Portugal, and a case-control study in a subsample of homes that had not been built or refurbished within the previous 6 months. A table of participant characteristics is provided for the cross-sectional portion of the study. The parents’ questionnaire study resulted in collection of data for only 1099 children out of 1639. It is unknown if there are differences between the participating and non-participating populations. The authors note that there was the potential for selection bias if parents of allergic children were more willing to participate, but also note that this potential bias was likely minimal. |
| Blinding | Probably low | There is no evidence of blinding. However, self-report of asthma outcomes is likely to not be influenced as participants likely are unaware of exposure status at low levels. |
| Outcome assessment | Low | Spirometry tests were performed by a well-trained technician according to the American Thoracic Society/European Repiratory Society (ATS/ERS) guidelines for all children with parental consent. A random subsample of five children from each classroom were tested for exhaled nitric oxide measurements according to the ATS/ERS guidelines using a device with a delection limit of 5 ppb. Symptoms and health status were reported by parents based on a standardized and validated questionnaire. |
| Confounding | Probably low | The study included several characteristics, such as age, parents' education level, smoking (not allowed in schools) (Tier I), gender, number of siblings, location of home from traffic, type of home, size of home (Tier II). However, authors do not report adjusting for ETS or smoking at home, so study was rated probably low risk of bias. |
| Incomplete outcome data | Low | Due to revoked or missing consent, data was collected for 1099/1134 participants. Follow up appears to be complete for individuals included in the final sample. |
| Exposure assessment | Low | Formaldehyde was sampled using passive samplers and analyzed by HPLC with an ultraviolet detector according to ISO 16000-4. Duplicate samples were collected in one out of every three schools and in one bedroom out of every three homes. Field blanks were collected and analyzed. The detection limit for formaldehyde was noted as 0.075 ug/m3. |
| Selective outcome reporting | Low | Results are reported for all outcomes specified in the abstract and methods. |
| Conflict of interest | Low | This study was funded by the government, and all authors are affiliated with academic or government organizations. |
| Other sources of bias | Low | No other threats to internal validity were reported. |
