# Supplementary material for: Exposure to formaldehyde and asthma outcomes: A systematic review, meta-analysis, and economic assessment
Source: PLoS One. 2021 Mar 31;16(3):e0248258. doi: 10.1371/journal.pone.0248258 (PMC8011796; doi:10.1371/journal.pone.0248258)
Supplement: S57 Table — (DOCX) [file pone.0248258.s070.docx]

Supplemental Materials, Table 57. Characteristics of Madureira et al. 2016

| Bias domain | Authors’ judgment | Support for judgment |
| --- | --- | --- |
| Source population representation | Probably low | The potential selection biases presented by the authors would not necessarily be differential between cases and controls and the larger group from which they were sampled did not exhibit selection bias. |
| Blinding | Probably low | The outcome and some exposure measures were determined prior to the sampling in the homes through a survey—it is not know from the paper whether cases or controls were known to the home investigators when sampling was done, bt the formaldehyde samples were from monitoring data not rated subjectively. |
| Outcome assessment | Probably low | Asthma cases were based on affirmative responses to one of the questions related to self-reported asthma, but was not confirmed with doctor’s diagnosis. |
| Confounding | Probably low | All of tier 1 and 2 confounders measured, but only adjusted for age and SES (mother’s education as a surrogate). Did not adjust for smoking. |
| Incomplete outcome data | Low | No missing outcome data |
| Exposure assessment | Probably low | Formaldehyde monitoring was performed—field blanks and duplicate samples were collected. No data on QA/QC were presented. |
| Selective outcome reporting | Low | All of the study’s pre-specified (primary and secondary) outcomes outlined in the manuscript’s methods were reported. |
| Conflict of interest | Low | This study was funded by several organizations which appeared to be either government or foundations, and all authors are affiliated with academic or government organizations. |
| Other sources of bias | High | Madureira published a similar paper in a different journal in 2015 on the same cohort of children, reporting similar results |
