# Supplementary material for: Exposure to formaldehyde and asthma outcomes: A systematic review, meta-analysis, and economic assessment
Source: PLoS One. 2021 Mar 31;16(3):e0248258. doi: 10.1371/journal.pone.0248258 (PMC8011796; doi:10.1371/journal.pone.0248258)
Supplement: S58 Table — (DOCX) [file pone.0248258.s071.docx]

Supplemental Materials, Table 58. Characteristics of Malaka et al. 1990

| Bias domain | Authors’ judgment | Support for judgment |
| --- | --- | --- |
| Source population representation | Low | A random sample of 100 workers at a plywood manufacturing company, stratified by smoking habit and length of service ( 5 y and 5 y), was selected to represent the exposed group. Another 100 workers, matched for age, ethnicity, and smoking habit, were selected from a nonexposed population. 93 exposed and 93 unexposed workers completed the study questionnaire; 55 exposed and 50 referents participated in the spirometry measurements. |
| Blinding | Probably high | There is no evidence of blinding in the spirometer measurements or the exposure measurements. Workers were likely aware of their exposure status. |
| Outcome assessment | Low | Across-shift spirometric measurements were conducted following the American Thoracic Society procedures. A randomly selected sub-sample of 10 exposed and 10 control individuals were examined by chest x-ray in a hospital; the results were interpreted by a hospital radiologist and reports were sent to the investigators. Respiratory symptoms were self-reported in a standardized respiratory questionnaire. |
| Confounding | Probably low | Smoking status, length of employment, age, height, weight, and ethnicity, dust exposure were addressed in the study design and/or analyses. Authors did not adjust for SES, but all workers are in the same occupation so it would not be unreasonable to assume that SES status was similar. |
| Incomplete outcome data | Low | Data are complete for 55(out of 93) exposed and 50 (out of 93) referents. 10% of participants did not participate in across-shift spirometric measurements. Authors provided follow up data for all participants, and the number who did not participate in the across-shift spirometric measurements is similar between exposed and unexposed. |
| Exposure assessment | Probably low | Passive samplers were used to collect area measurements of formaldehyde, and the colorimetric method used was recommended by the American Public Health Association. Measurements were also made in the homes of 10 workers. No details were provided on QA/QC methods. |
| Selective outcome reporting | Low | All of the study’s pre-specified (primary and secondary) outcomes outlined in the published manuscript’s methods, abstract, and/or introduction section that are of interest in the review have been reported in the pre-specified way. |
| Conflict of interest | Probably low | Authors were affiliated with an academic institution and there is no reason to believe that a conflict of interest exists. No details were provided on the funding source for the study. |
| Other sources of bias | Probably high | Subjects were identified based on measurements of formaldehyde in the plant. Controls were nonexposed workers. While asthmatics were included, some of the most affected could have left the job prior to the study taking place, thus introducing a healthy worker bias, which would likely bias the results towards the null. |
