# Supplementary material for: Exposure to formaldehyde and asthma outcomes: A systematic review, meta-analysis, and economic assessment
Source: PLoS One. 2021 Mar 31;16(3):e0248258. doi: 10.1371/journal.pone.0248258 (PMC8011796; doi:10.1371/journal.pone.0248258)
Supplement: S59 Table — (DOCX) [file pone.0248258.s072.docx]

Supplemental Materials, Table 59. Characteristics of Mapou et al. 2013

| Bias domain | Authors’ judgment | Support for judgment |
| --- | --- | --- |
| Source population representation | Probably low | Evaluation was based on available information from the RIOPA study. Participant characteristics are provided. |
| Blinding | Probably low | There are no details provided on blinding in the RIOPA study, but it is unlikely that study participants were aware of their exposure level. |
| Outcome assessment | Probably low | Health outcomes of doctor-diagnosed asthma or bronchitis were self-reported in the RIOPA study. The RIOPA questionnaires were based on the National Human Exposure Assessment Survey. |
| Confounding | Probably low | The gender, education level, and household income level of participants are presented. The model adjusted for other factors, including type of vehicle driven and season. Activities that could potentially influence in-vehicle air contamination were self-reported in the RIOPA survey. However, study participants were selected to be nonsmokers (this should be noted in the explanation). However, while there may be similar demographics, the formaldehyde results are simple correlations that do not adjust for SES, a Tier 1 criteria. |
| Incomplete outcome data | Probably low | Outcome data was available for 190 individuals and exposure measurements were available for 222 individuals. |
| Exposure assessment | Low | Exposure assessment procedures were noted in supplemental materials as conducted through detailed sampling protocols; personal air monitoring was conducted near the breathing zone, passive monitoring was conducted and detailed instructions were provided to participants. |
| Selective outcome reporting | Low | Results were presented for all outcomes discussed in the abstract. |
| Conflict of interest | Probably high | The authors are university affiliated. The study was funded by the Mickey Leland National Urban Air Toxics Research Center, and the Health Effects Institute (co-funded by the US EPA and automotive manufacturers). HEI is a nonprofit corporation chartered in 1980 as an independent research organization to provide high-quality, impartial, and relevant science on the health effects of air pollution. However, partial funding for original RIOPA provided by automotive manufacturers. |
| Other sources of bias | High | The authors note that inconsistencies across assigned participant identification numbers resulted in assumptions used to match participant data across data files, which potentially led to information bias/error. It is unclear how much of the data was affected by the inconsistencies. |
