# Supplementary material for: Exposure to formaldehyde and asthma outcomes: A systematic review, meta-analysis, and economic assessment
Source: PLoS One. 2021 Mar 31;16(3):e0248258. doi: 10.1371/journal.pone.0248258 (PMC8011796; doi:10.1371/journal.pone.0248258)
Supplement: S60 Table — (DOCX) [file pone.0248258.s073.docx]

Supplemental Materials, Table 60. Characteristics of Marks et al. 2010

| Bias domain | Authors’ judgment | Support for judgment |
| --- | --- | --- |
| Source population representation | Low | Subjects selection was done in a cluster-randomized fashion, in a two-stage selection process in which schools and classrooms were selected and then participants within the selected classrooms were recruited. Details about the installation and control of heaters, blinding of participants, and the randomization procedure are provided in the Supplemental Material, and do not provide indication of potential risk of selection bias. |
| Blinding | Low | The study is a double-blind, cluster-randomized, crossover study. |
| Outcome assessment | Low | FEV outcomes were measured using valid methods and some QA/QC information was included. Children kept a daily symptom and medication diary for the 6-week study period. There is a likelihood that the self measurements were biased, and there is no validation of the self reported sx/diary data. However, study rated as low risk of bias because objective measures (pulmonary function tests) were used to determine outcome. |
| Confounding | Low | Potential confounders evaluated included heater type allocation, study period (first, second, or third pair of weeks), and day of the week; unflued gas heaters or open fires at home and exposure to ETS at home. Current asthma status by heater type and atopic status by heater type interactions. Study rated low risk of bias because the results are for the use of certain types of heaters as a proxy for formaldehyde, so the authors reported effect estimates that were adjusted for smoking. Also, the exposure was randomly done for each class room 'For each classroom, the order of operation of the unflued and flued gas heaters was randomized separately for each pair of weeks.' Thus each classroom was compared to itself, and confounding by SES not an issue. |
| Incomplete outcome data | Low | Overall participation rate was 77%, and in any given week average participation rates ranged from 64% to 73%. 56b (out of 400) subjects had missing atopy information. However, the authors used mixed models to treat missing values as missing at random. |
| Exposure assessment | Low | Monitoring was performed using a validated method and details were included to address QA/QC. Formaldehyde badges incorporated a "blank correction" section so separate blanks were not required. The formaldehyde badges were analysed using High Performance Liquid Chromatography according to OSHA Method 64 LOD was not reported, however lack of LOD not an issue as long as most/all people are above the LOD. |
| Selective outcome reporting | Low | All of the outcomes outlined in the methods, abstract, and/or introduction section that are of interest in the review have been reported. |
| Conflict of interest | Probably high | This study was funded by the New South Wales Department of Education and Training, who facilitated its implementation. The authors declared they have no actual or potential competing financial interests. However, there is indirect evidence that study was not free of support from a company selling a certain type of heater, hence with a vested interest in the outcome of the study: The study received support from Bowin mfg Pty Ltd (Sydney, Australia) in designing and implementing the installation and operation of the flued and unflued gas heaters. |
| Other sources of bias | Low | There is no evidence of other potential sources of bias. |
