# Supplementary material for: Exposure to formaldehyde and asthma outcomes: A systematic review, meta-analysis, and economic assessment
Source: PLoS One. 2021 Mar 31;16(3):e0248258. doi: 10.1371/journal.pone.0248258 (PMC8011796; doi:10.1371/journal.pone.0248258)
Supplement: S61 Table — (DOCX) [file pone.0248258.s074.docx]

Supplemental Materials, Table 61. Characteristics of Matsunaga et al. 2007

| Bias domain | Authors’ judgment | Support for judgment |
| --- | --- | --- |
| Source population representation | Probably low | The authors described the recruitment of pregnant women within the Osaka Maternal and Child Health Study, a Japanese prospective cohort. The study group was purely volunteer and women had to be pregnant. Of the 1,002 women who consented to participate, 998 had all exposure data and were included in the analysis. Subject characteristics are presented. However, data on personal characteristics, were not available for nonparticipants, and could not be compared with participants. |
| Blinding | Probably low | Blinding of participants is not reported, though it would be unlikely that they would be aware of their formaldehyde exposure levels. However, it is not known whether the analytic chemists were blind to the participant's outcome status. |
| Outcome assessment | Probably low | Asthma was determined by self-report of any medical treatment for asthma in the past 12 months. However, detailed data on the types of medications and the duration of their use were not collected. There is a potential for loss of asthma cases since women who want to become pregnant or who are pregnant might tend to avoid drugs. Study rated as low risk of bias because asthma diagnosis confirmed by medical history, not objective testing. Based on description, can assume both groups were asked the same questions. |
| Confounding | Low | The researchers adjusted for all Tier I and some Tier II confounders. Allergens and familial allergic hystory history were also included in their analysis. |
| Incomplete outcome data | Low | Of the 1,002 women who consented to participate, 998 had all exposure data and were included in the analysis. The authors indicate that when incomplete questionnaires were received at the data center, they would follow up the participants with a telephone interview to complete the questionnaire. |
| Exposure assessment | Probably high | Passive diffusion air sampling tubes worn for 24 hours were were measured by spectrophotometer and had agreement with LC results. Other QC and limits of detection were not addressed. There is no information on how the samplers were worn, or on how researchers assured consistency/compliance for study subjects. |
| Selective outcome reporting | Low | All of the study’s pre-specified (primary and secondary) outcomes outlined in the published manuscript’s methods, abstract, and/or introduction section that are of interest in the review have been reported in the pre-specified way. |
| Conflict of interest | Low | Government funding, all authors are affiliated with academic/government institutions. |
| Other sources of bias | Low | The study appears to be free of other sources of bias. |
