# Supplementary material for: Exposure to formaldehyde and asthma outcomes: A systematic review, meta-analysis, and economic assessment
Source: PLoS One. 2021 Mar 31;16(3):e0248258. doi: 10.1371/journal.pone.0248258 (PMC8011796; doi:10.1371/journal.pone.0248258)
Supplement: S62 Table — (DOCX) [file pone.0248258.s075.docx]

Supplemental Materials, Table 62. Characteristics of Mi et al. 2006

| Bias domain | Authors’ judgment | Support for judgment |
| --- | --- | --- |
| Source population representation | Low | Two school districts in Shanghai were randomly selected (specific randomization methods not reported). None of the schools reported complaints regarding the school environment. Five schools from each district were randomly selected. Three classes from each school were selected, and all students received a questionnaire (n=1435). 1414 students participated (99%). |
| Blinding | Probably low | Blinding was not addressed. However, outcome data were self-reported prior to the exposure assessment, so participants would have been blind to their exposure levels. Air samples were analyzed at an accredited laboratory, thus knowledge of outcomes unlikely to bias exposure assessment. |
| Outcome assessment | Probably low | Current asthma and current respiratory symptoms among pupils assessed through self-report on single questionnaire which required language translation bases on the European Community Respiratory Health Survey and a previous Swedish school study. Current asthma was defined as having either current asthma medication, or having had an asthma attack during the previous 12 months, as in the ECRHS study. However diagnoses were not confirmed by a physician. |
| Confounding | Probably low | Most of Tier I (no SES) and some of Tier II confounders accounted for, including age, smoking, gender, building dampness, other exposures. Smoking information was obtained from the study questionnaire. School environments were "inspected," but the authors did not report the specific method they used to determine the presence of water leaks and/or indoor mold. |
| Incomplete outcome data | Low | Table 1 reports data for 1412 individuals, (for a total number of included participants of 1414). Results (Tables 5 and 6) are complete for 1414 students. |
| Exposure assessment | Probably low | Formaldehyde was measured by pumped air sampling. Measurements were taken for 7 days (4 hours a day) by diffusion sampling. Filters were analyzed by liquid chromatography. Samplers were placed on a table near one wall 0.9 m above the floor. Samplers were analyzed at an accredited laboratory. Authors did not report reliability information or QC methods. |
| Selective outcome reporting | Low | All of the study’s pre-specified (primary and secondary) outcomes outlined in the published manuscript’s methods, abstract, and/or introduction section that are of interest in the review have been reported in the pre-specified way. |
| Conflict of interest | Probably low | Authors were affiliated with the university and university hospitals of Sweden and Shanghai as well as Swedish Environmental Research Institute, however no information on funding was provided. There is no reason to expect potential conflict of interest. |
| Other sources of bias | Low | The study appears to be free of other sources of bias. |
