# Supplementary material for: Exposure to formaldehyde and asthma outcomes: A systematic review, meta-analysis, and economic assessment
Source: PLoS One. 2021 Mar 31;16(3):e0248258. doi: 10.1371/journal.pone.0248258 (PMC8011796; doi:10.1371/journal.pone.0248258)
Supplement: S63 Table — (DOCX) [file pone.0248258.s076.docx]

Supplemental Materials, Table 63. Characteristics of Milton et al. 1996

| Bias domain | Authors’ judgment | Support for judgment |
| --- | --- | --- |
| Source population representation | Low | The authors recruited 32 volunteers from two different work groups at the wool plant. Inclusion and exclusion criteria and demographic information were discussed in detail and seemed adequate. |
| Blinding | Probably high | No blinding reported--measured exposures as well as self-reported outcomes through standard American Thoracic Society questionnaire, other outcomes of spirometry and peak expiratory flow (PEF validated by spirometric PEF) may have been affected by knowledge of exposure and/or outcome. |
| Outcome assessment | Low | Outcomes of respiratory symptoms and asthma by self-report from validated standard American Thoracic Society respiratory questionnaire; change in peak expiratory flow were self-administered however details on training and appropriate methods provided and were validated by correlation with spirometric PEF; spirometry conducted by trained, experienced technicians. Study rated probably low risk of bias because asthma diagnosis confirmed by medical history, not objective testing. Based on description, assume both groups were asked the same questions. |
| Confounding | Low | All of Tier I, and most of Tier II confounders were accounted for, analyses of within-subject changes in outcomes and case-crossover design controlled for time-invariant confounders, as well as additional environmental exposures of endotoxin and phenolic resin and work shift. |
| Incomplete outcome data | Low | This study appeared to have complete outcome data as there was no discussion of missing data. |
| Exposure assessment | Probably low | Exposure sampling was conducted by active area and personal sampling with two samples per day, exposure sampled on the same days as outcome (PEF) logs, for 4 hours each with the inlet of exposure monitor documented as within the breathing zone of subject (lapel placement or within respirator). Exposure groups for analysis based upon exposure measurements as well as current work location and job title. Minimal QA/QC information provided. |
| Selective outcome reporting | Low | Results were presented for all the relevant outcomes specified. |
| Conflict of interest | High | The authors were academic however research was supported by Owens Corning Fiberglass a commercial company with an interest in the exposure studied. This study was also supported by Harvard's NIEHS Environmental Health Sciences Center and NIOSH. |
| Other sources of bias | High | Authors recruited 32 volunteers from two different work groups at the wool plant. Authors admit to potential selection bias in the form of healthy worker effect via transfer bias, based on evidence of the inability to recruit senior maintenance workers because they did not want to have to work in the high exposure areas. Based on seniority, production workers could bid to move among basement, forehearth, and oven jobs. The lowest exposed job open to production workers, the packing crew, was an entry level position only. A healthy worker bias would likely bias the results towards the null |
