# Supplementary material for: Exposure to formaldehyde and asthma outcomes: A systematic review, meta-analysis, and economic assessment
Source: PLoS One. 2021 Mar 31;16(3):e0248258. doi: 10.1371/journal.pone.0248258 (PMC8011796; doi:10.1371/journal.pone.0248258)
Supplement: S64 Table — (DOCX) [file pone.0248258.s077.docx]

Supplemental Materials, Table 64. Characteristics of Norback et al. 1995

| Bias domain | Authors’ judgment | Support for judgment |
| --- | --- | --- |
| Source population representation | Low | The study populations was selected from a subsample of individuals who participated in the European Community respiratory health survey. The survey included a random sample of 20-44 year old adults living in Uppsala, Sweden in 1990. A random subsample of participants were further examined, and from this subsample, all subjects who reported a respiratory symptom (n=74) and 80 non-matched subjects without symptoms were recruited. 47 subjects with asthmatic symptoms (64%) and 41 participants without asthmatic symptoms (57%) participated. The overall response rate for this second phase was 81%. |
| Blinding | Low | The authors note that the study was blinded because exposure measurements were not linked to medical information until the data collection was completed. |
| Outcome assessment | Low | Spirometer measurements were made in triplicate and peak flow variability was calculated. Bronchial hypersensitivity determined by methacholine challenge, performed by a MEFAR inhalaion dosimeter. Symptoms were self-reported using a modified version of the International Union against Tuberculosis and Lung Disease questionnaire. |
| Confounding | Probably low | Potential confounders considered in the analyses included current smoking (Tier I), age, and sex, type o house, presence of pets at home, age of home, living near traffic (Tier II). SES was not specifically addressed. |
| Incomplete outcome data | Low | There is no apparent missing data. |
| Exposure assessment | Probably low | Indoor concentrations of formaldehyde were measured with glass fiber filters impregnated with 2,4-dinitro-phenylhydrazine. The air sampling rate was 0-25 1/min for two hours. The filters were analyzed by liquid chromatography. No further details were provided. |
| Selective outcome reporting | Low | All of the study’s pre-specified (primary and secondary) outcomes outlined in the published manuscript’s methods, abstract, and/or introduction section that are of interest in the review have been reported in the pre-specified way. |
| Conflict of interest | Probably high | All study authors are affiliated with academic institutions. However, funding was received from Pharmacia, a for profit pharmaceutical company - "This study was supported by grants from the Swedish Association against Asthma and Allergy, The Swedish Medical Research Council, The Swedish Society of Medicine, The Swedish Heart and Lung Foundation, The Bror Hjerpstedts Foundation, Pharmacia and the County Council of Uppsala." |
| Other sources of bias | Low | Formaldehyde-specific results are only reported for nocturnal breathlessness. |
