# Supplementary material for: Exposure to formaldehyde and asthma outcomes: A systematic review, meta-analysis, and economic assessment
Source: PLoS One. 2021 Mar 31;16(3):e0248258. doi: 10.1371/journal.pone.0248258 (PMC8011796; doi:10.1371/journal.pone.0248258)
Supplement: S65 Table — (DOCX) [file pone.0248258.s078.docx]

Supplemental Materials, Table 65. Characteristics of Norback et al. 2000

| Bias domain | Authors’ judgment | Support for judgment |
| --- | --- | --- |
| Source population representation | Low | All staff working day shifts (n=95) at four out of eight geriatric hospitals in the Swedish municipality of Ystad were invited to participate in the study. The four sites were selected as representing buildings with different age and design, irrespective of the occurrence of medical symptoms. There was a 93% participation rate (88/95), and one participant was excluded for not answering questions on asthma or asthma symptoms. |
| Blinding | Probably low | There is no information on blinding, and it is possible that subjects were aware of their exposure. However, outcome was evaluated by a doctor and it is unlikely the doctor knew about the participant's exposure status. |
| Outcome assessment | Probably low | The subjects were questioned by a doctor about allergies and other diseases, medications, occupational data, smoking habits and social status. Atopy was defined as a history of allergic manifestations related to exposure to common IgE-mediated allergens in Sweden (tree or grass pollen or furry animals), or a history of childhood eczema using doctor-administered standardized questionnaire. Questions on asthma included one question on doctor’s diagnosed asthma and year of diagnosis. In addition, three key questions on respiratory symptoms, obtained from the European Community Respiratory Health Survey (ECRHS), were used. Questionnaire included standard questions and questions about doctor diagnosed asthma. |
| Confounding | Probably high | Authors measured both Tier I confounders, smoking and SES (factor measured here was "social status"). In addition they measured several Tier II confounders including sex, age, atopy, and other environmental co-exposures. However, it is unclear if the authors adjusted for this information or just collected this information. |
| Incomplete outcome data | Low | There was 10% missing data (8/95). |
| Exposure assessment | Probably low | Indoor concentrations of formaldehyde were sampled on passive samplers for 7 days, and analyzed by high-performance liquid chromatography with glass fiber filters impregnated with 2,4-dinitrophenylhydrazine. No QA/QC methods were described. Information on exposures in the participant’s residence was obtained from a self-administered questionnaire, using the same questions as in earlier studies. |
| Selective outcome reporting | Low | Results are reported for all outcomes specified in the abstract and methods. |
| Conflict of interest | Low | The study was funded by government organization, and all authors were affiliated with an academic institution. |
| Other sources of bias | Probably low | Subjects were staff working day shifts in 4 Swedish hospitals with different years of construction/design. Although a healthy worker bias is possible, the study is not occupational per se--not aimed at workers knowingly exposed to formaldehyde. It is geared more as an analysis of SBS. The concentrations of formaldehyde were low for all buildings. Because of this study design, there is a probably low risk for healthy worker bias, which may bias the results towards the null. |
