# Supplementary material for: Exposure to formaldehyde and asthma outcomes: A systematic review, meta-analysis, and economic assessment
Source: PLoS One. 2021 Mar 31;16(3):e0248258. doi: 10.1371/journal.pone.0248258 (PMC8011796; doi:10.1371/journal.pone.0248258)
Supplement: S66 Table — (DOCX) [file pone.0248258.s079.docx]

Supplemental Materials, Table 66. Characteristics of Nordman et al. 1985

| Bias domain | Authors’ judgment | Support for judgment |
| --- | --- | --- |
| Source population representation | Low | 230 patients with suspected formaldehyde-induced asthma were identified, and 12 cases were considered to be caused by specific sensitization to formaldehyde. Patients were all referred to the Institute of Occupational Health from all over Finland, and all had been exposed to formaldehyde and were suffering from respiratory symptoms compatible with asthma attacks. Patient characteristics for the 12 cases included in the study are described in detail. |
| Blinding | High | There is no discussion of blinding. Outcome measures included spirometry performed by a nurse and bronchial challenge with formaldehyde. Controls were performed, though authors note that it was easy for participants to tell the difference between formaldehyde exposure and some of the controls. |
| Outcome assessment | Low | Clinical examinations included a detailed medical and occupational history, and lung function tests including a bronchial challenge with formaldehyde and a negative control. All PEF measurements were recorded by a nurse and were measured every 3 hours for at least 24 hours before the inhalation testing started, and the best of three measurements were recorded. The degree of bronchial hyperreactivity was assessed with a histamine challenge test. Provocation tests lasted for 30 minutes in the morning and PEF was measured every 15 minutes for the first hour, hourly for 8 hours, and in 3 hour intervals until the following morning. The authors note that there is a possibility of false positives in diagnosing formaldehyde-induced asthma. |
| Confounding | Low | Characteristics are described in detail for 5 case patients. Study rated low because an experimental study where the authors exposed the subjects to formaldehyde and measured their reaction--each person serves as their own control. |
| Incomplete outcome data | Low | There is no apparent missing data. |
| Exposure assessment | Probably low | This was a controlled exposure in a chamber. It is unclear if methods used to expose participants were validated, but they were cited as being previously used in the literature (Pepys et al (36) and Newman Taylor and Davies (37). NIOSH methods use to measure formaldehyde concentrations. However, the authors note that variability in humidity and fluctuations in temperature in the chamber may have caused greater variability in concentrations than the four control measurements revealed. No QA/QC methods were described. |
| Selective outcome reporting | Low | Authors report results relevant to research question of interest. |
| Conflict of interest | Probably low | The study authors were affiliated with a government organization. However, there is no information on the source of funding. |
| Other sources of bias | Probably low | Subjects were patients referred to Institute of Occupational Health because of suspected formaldehyde-induced asthma. Since the only subjects included were those with reasonably severe symptoms linked specifically to their job, this study may have a more limited risk of healthy worker bias, which may bias the results towards the null. |
