# Supplementary material for: Exposure to formaldehyde and asthma outcomes: A systematic review, meta-analysis, and economic assessment
Source: PLoS One. 2021 Mar 31;16(3):e0248258. doi: 10.1371/journal.pone.0248258 (PMC8011796; doi:10.1371/journal.pone.0248258)
Supplement: S67 Table — (DOCX) [file pone.0248258.s080.docx]

Supplemental Materials, Table 67. Characteristics of Popa et al. 1969

| Bias domain | Authors’ judgment | Support for judgment |
| --- | --- | --- |
| Source population representation | Probably high | Selection methods appear to be different for two groups of subjects. Nineteen subjects were recruited from the Allergy Unit of a Department of Occupational Medicine (no details), and 29 subjects were recruited during an epidemiological survey of chronic bronchitis in some industries (details published elsewhere). Inclusion criteria were paroxysmal dyspnea of bronchial asthma or asthmatic bronchitis that was determined only by contact with micromolecular substances with irritating and sensitizing properties (MSIS), and no bronchial asthma or asthmatic bronchitis that was present prior to occupational contact with MSIS. Subjects characteristics are not presented, and there is no information on participation rates. |
| Blinding | Probably high | No information was provided about blinding of participants or study administrators. This study included several different measures of outcomes including biomarkers and inhalation tests that included testing of controls (diluent alone). It is unclear if the patients were aware of control tests vs. MSIS tests. Workers may have been aware of exposures. |
| Outcome assessment | Low | Respiratory tests were also carried out at work in 20 subjects, with 14 examined clinically and spirographically and 6 examined clinically only. |
| Confounding | High | Authors do not mention either possible Tier I confounder (smoking or SES). Of possible Tier II confounders authors mention other occupational exposures. Subject characteristics are not presented. |
| Incomplete outcome data | Low | There is no apparent missing data. |
| Exposure assessment | Probably high | Limited information is provided on exposure methods and measurement. While this was a controlled exposure study, the methods used do not appear to be validated methods. Inhalation tests consisted of a 5-minute nebulization in an open circuit system (flow = 0.2 ml/rnin of the substance tested) of concentrations of various chemicals (formalin: 1:2,500, formaldehyde resin "fume"). Inhalation tests included controls to test for irritating effect of diluent alone. One individual was exposed to urea formaldehyde resin fume, but there was no evidence of measuring the concentration of formaldehyde. |
| Selective outcome reporting | Low | Results are reported for all outcomes specified. |
| Conflict of interest | Probably low | No funding information is provided. Authors are affiliated with hospitals or academic institutions. There is no reason to believe that a conflict of interest exists. |
| Other sources of bias | Probably low | Subjects were patients referred to study because of suspected asthma from occupational contact with various substances. Since the only subjects included were those with reasonably severe symptoms linked specifically to their job, this study may have a more limited risk of healthy worker bias, which may bias the results towards the null. |
