# Supplementary material for: Exposure to formaldehyde and asthma outcomes: A systematic review, meta-analysis, and economic assessment
Source: PLoS One. 2021 Mar 31;16(3):e0248258. doi: 10.1371/journal.pone.0248258 (PMC8011796; doi:10.1371/journal.pone.0248258)
Supplement: S68 Table — (DOCX) [file pone.0248258.s081.docx]

Supplemental Materials, Table 68. Characteristics of Pourmabahabadian et al. 2006

| Bias domain | Authors’ judgment | Support for judgment |
| --- | --- | --- |
| Source population representation | Probably high | Participants were selected from 7 large hospitals in Tehran. Case groups included 124 exposed staff from pathology labs, surgery rooms, endoscopy, and controls included 56 administrative staff. No information provided on how participants were selected, or on the participation rate. Also, it is unclear if the study measured formaldehyde in the control environment or a description of how high levels might be in the other departments. |
| Blinding | Probably high | Blinding of study participants or staff is not mentioned. Outcomes were measured by spirometry and self-report. Given the different job functions of participants, they may have been aware of their exposure status so there is some risk of bias. Investigator knowledge of exposure status could potentially bias lung function measurements as well. |
| Outcome assessment | Low | Symptoms were self-reported in a questionnaire, and spirometric tests were performed for all subjects. Study rated low risk of bias because objective measures (pulmonary function tests) used to determine outcomes. |
| Confounding | High | Authors include the Tier I confounder of smoking in their review, but do not address SES. The study reports information on sex and age and found differences between exposed and non-exposed groups with respect to age, sex, length of service. Results do not appear to be adjusted for covariates. Study rated high risk of bias because authors recorded smoking but it does not appear that they accounted for smoking in their analysis of the comparison of cases and controls. |
| Incomplete outcome data | Low | There is no missing data. |
| Exposure assessment | Probably high | Continuous and spot sampling of formaldehyde in the case departments was conducted at the 7 hospitals. From 6 to 42 8-hour continuous samples were taken, plus spot samples. Methods are not described. It is unclear how non-exposed locations (administrative offices) were determined to be free of formaldehyde. |
| Selective outcome reporting | Low | Results are reported for all outcomes specified in the abstract and methods. |
| Conflict of interest | Probably low | Authors were affiliated with academic institutions. Funding source not specified, and there is no reason to believe that a conflict of interest exists. |
| Other sources of bias | Probably high | Subjects were individuals working in seven large hospitals of Tehran University of Medical Sciences. Case groups from pathology labs, surgery rooms, and endoscopy; controls were administrative staff. While asthmatics were included, some of the most affected could have left the job prior to the study taking place, thus introducing a healthy worker bias, which would likely bias the results towards the null. |
