# Supplementary material for: Exposure to formaldehyde and asthma outcomes: A systematic review, meta-analysis, and economic assessment
Source: PLoS One. 2021 Mar 31;16(3):e0248258. doi: 10.1371/journal.pone.0248258 (PMC8011796; doi:10.1371/journal.pone.0248258)
Supplement: S69 Table — (DOCX) [file pone.0248258.s082.docx]

Supplemental Materials, Table 69. Characteristics of Quackenboss et al. 1989

| Bias domain | Authors’ judgment | Support for judgment |
| --- | --- | --- |
| Source population representation | Probably high | Limited information on selection/recruitment as authors noted; authors only noted responses from a screening health and environmental questionnaire used to classify and select a population based upon risk and exposure factors. Authors cite previous work (Quackenboss et al. 1989). Results from 151 households are discussed in this study. |
| Blinding | Probably low | No mention of blinding of investigators and lung function measures could potentially be influenced with knowledge of participant's exposure status, but unclear if participants were aware of exposure levels. |
| Outcome assessment | Probably low | Peak expiratory flow rates were measured by self-testing (2x per week for at least 5 days during each testing week) following verbal instructions and examples given by trained technicians. Authors mention that there may have been variability in individuals abilities to consistently follow instructions. Allergic symptoms, asthma and nonspecific complaints obtained through symptom diary with no mention of validation. |
| Confounding | Probably low | Authors measure some demographic variables including age, and sex and other possible confounders including smoking and previous lung diseases. SES is not addressed. Possible temporal differences such as day of week are also considered. |
| Incomplete outcome data | Probably low | Unclear if there were missing outcome data-- unable to even find the number of participants in the study. |
| Exposure assessment | Low | Exposure assessment utilized passive sampling tubes. Measurements were made over one week period from several locations within households at breathing level. Assessments were made in several rooms of house--kitchen, main living and each subject's bedroom, and were compared with active area monitoring as well as analyzed with repeat sampling; samplers were developed at Lawrence Berkeley Labs and were evaluated for sensitivity (Hodgson et al. 1984). Samplers were calibrated and a second type of sampler was used as well for comparison. |
| Selective outcome reporting | Low | Authors report results relevant to questions mentioned in abstract and introduction. |
| Conflict of interest | Low | Study funding was provided by the EPA and authors belong to an academic institution. |
| Other sources of bias | Low | No other threats to internal validity were identified. |
