# Supplementary material for: Exposure to formaldehyde and asthma outcomes: A systematic review, meta-analysis, and economic assessment
Source: PLoS One. 2021 Mar 31;16(3):e0248258. doi: 10.1371/journal.pone.0248258 (PMC8011796; doi:10.1371/journal.pone.0248258)
Supplement: S70 Table — (DOCX) [file pone.0248258.s083.docx]

Supplemental Materials, Table 70. Characteristics of Raaschou-Nielsen et al. 2010

| Bias domain | Authors’ judgment | Support for judgment |
| --- | --- | --- |
| Source population representation | Probably low | This was a clinical cohort study from the Copenhagen Prospective Study on Asthma in Childhood (COPSAC) consisting of 411 infants of mothers with asthma. This study included 378 infants (the rest were lost to follow up by 18 months). There were no data presented comparing the lost to follow up population to the full cohort. This cohort was described previously and this article did not contain the inclusion/exclusion criteria. |
| Blinding | Probably high | There is no indication of blinding. Children enrolled in the study attended the clinical research unit for airway symptoms. Parents deployed and retrieved in home environmental sampling media and outcomes were recorded by mother's diaries, likely aware of the study goal. |
| Outcome assessment | Probably low | Parental report of children's outcomes of wheezing symptoms, however training was provided, diary entries reviewed at a visit to dedicated clinical research unit every sixth month and at visits after a 3-day period of respirator symptoms, parents provided book on asthma-like symptoms; sensitivity analyses using data from only those children filling out symptom diaries for at least 90% of time yielded similar conclusions. |
| Confounding | Probably low | Confounders accounted for included by sex, area of residence, mother's education and lung function measured at one month of age; no measure of ETS however authors noted 78% of parents reported ETS exposure less frequent than one day per week during first year of infant's life and all children had mothers with asthma. |
| Incomplete outcome data | Low | There was little (10%) missing formaldehyde data well documented for each modeling approach used |
| Exposure assessment | Probably low | The authors used diffusive passive samplers to measure formaldehyde, at 3 timepoints (6, 12, 18 months for a 10 week duration) in the bedroom of each subject. Samplers were deployed by parents and mailed back to the clinical research unit after sampling. The authors indicate that the formaldehyde sampler had its sampling window modified to make the sampling rate more adequate for the 10 week sampling period. Limits of quantification were not presented. |
| Selective outcome reporting | Low | Results were presented for all the relevant outcomes specified. |
| Conflict of interest | High | The authors were Danish Cancer Society and university affiliated. However, the study was supported by several private foundations, government grants and unrestricted institutional research grants from AstraZenaca, LEOpharma, Pharmacia-Pfizer, and Yamanouchi Pharma. |
| Other sources of bias | Low | No other sources of bias identified. |
