# Supplementary material for: Exposure to formaldehyde and asthma outcomes: A systematic review, meta-analysis, and economic assessment
Source: PLoS One. 2021 Mar 31;16(3):e0248258. doi: 10.1371/journal.pone.0248258 (PMC8011796; doi:10.1371/journal.pone.0248258)
Supplement: S71 Table — (DOCX) [file pone.0248258.s084.docx]

Supplemental Materials, Table 71. Characteristics of Rumchev et al. 2002

| Bias domain | Authors’ judgment | Support for judgment |
| --- | --- | --- |
| Source population representation | Probably low | Cases and controls were children of the same age group. Cases were identified from a single hospital in Perth, Australia; controls were identified through the health department in Perth, Australia and recruited from the community. The authors noted that this sample of controls from the community was more likely to reflect the demographics of the cases identified through the hospital. Housing characteristics were similar in both groups. Participation rates were not provided, but authors note strategies used to minimize potential for selection bias. |
| Blinding | Probably low | There is no evidence of blinding. It is unlikely that doctors diagnosing patients had knowledge of their exposure level. In home sampling protocols had insufficient detail to determine if research personnel had knowledge of case status. However, exposure concentrations were determined by liquid chromatography, which is not likely to be influenced by knowledge of case status. |
| Outcome assessment | Probably low | Asthma was diagnosed by a physician, but authors note that it is difficult to differentiate wheezing illnesses in the study's age group. |
| Confounding | Low | While the researchers adjusted for all of Tier I and some Tier II confounders, including age, socioeconomic status, and exposure to indoor smoking, as well as several other factors including sex, and family history of asthma. |
| Incomplete outcome data | Probably low | The authors indicate that there were low attrition rates between cases (5%) and controls (8%). There is no other apparent missing data, but figures do not present n values. |
| Exposure assessment | Probably low | Passive samplers were placed in the bedroom and living room of participants for an 8 hr sampling period once in summer and once in winter in the center of the living area at 1 meter and at pillow height near the bed in the bedroom of the child. Factors that could affect sampling were measured or accounted for such as humidity and UV light exposure. No QA/QC or detection limits were presented, however a method paper was referenced. |
| Selective outcome reporting | Low | Results were presented for all outcomes outlined in the abstract and methods. |
| Conflict of interest | Probably low | All authors are from academic institutions. Sources of funding were not presented, and no conflict of interest statement was made. |
| Other sources of bias | High | Authors mention the potential for questionnaire responses to be subject to recall or observational biases. Even with the use of a standardized questionnaire of respiratory symptoms, parents may interpret the symptom questions and, therefore, report their child's symptoms in different ways. There was a typographical error in the reporting of results that could not be confirmed by authors upon personal communication. |
