# Supplementary material for: Exposure to formaldehyde and asthma outcomes: A systematic review, meta-analysis, and economic assessment
Source: PLoS One. 2021 Mar 31;16(3):e0248258. doi: 10.1371/journal.pone.0248258 (PMC8011796; doi:10.1371/journal.pone.0248258)
Supplement: S72 Table — (DOCX) [file pone.0248258.s085.docx]

Supplemental Materials, Table 72. Characteristics of Sauder et al. 1987

| Bias domain | Authors’ judgment | Support for judgment |
| --- | --- | --- |
| Source population representation | High | Nine nonsmoking volunteers with a characteristic clinical history of asthma (American Thoracic Society, 1962) and documented hyperreactive airways were recruited from the community. Very little information provided on where volunteers recruited and limited demographic information provided. |
| Blinding | High | Chamber study with no mention of blinding, which means the participants and researchers were likely aware of exposures as they were administered. |
| Outcome assessment | Low | Outcomes were assessed using several methods. Symptoms were self-reported using a questionnaire; the questionnaire included sham questions about heart palpitations and tingling in feet or hands. Spirometric measurements were performed according the American Thoracic Society criteria. Nonspecific airway reactivity was assessed by a methacholine challenge according to a modification of a previously published method (Chai, 1975). Each subject served as his or her own control, receiving clean air during one trial and 3 ppm formaldehyde in another trial. The trials were separated by one week and occurred at the same time of day. Study rated as low risk of bias because objective measures (pulmonary function tests) used to determine outcomes. |
| Confounding | Probably low | All volunteers were nonsmokers. There is no measurement of the other Tier I confounder, SES. A few Tier II confounders including age and sex were reported. Each subject served as his or her own control. |
| Incomplete outcome data | Low | There is no missing data. |
| Exposure assessment | Low | The study was conducted in an environmentally controlled chamber that was continuously monitored by two air monitors fitted with formaldehyde reagent modules. Authors provide detailed exposure methods and monitoring of the system to ensure accurate exposure. Hourly exposure chamber samples were also collected using midget impingers containing 1% sodium bisulfite. The samples were analyzed for HCHO using the NIOSH Chromotrophic Acid Technique (NIOSH, 1977). Air monitors and impinger samples agreed within 5% of each other. |
| Selective outcome reporting | Low | Results are reported for all outcomes specified in the abstract and methods. |
| Conflict of interest | Low | Researchers were academic and funding came from the Department of Energy. |
| Other sources of bias | Probably low | The authors did not specify whether the paired t-test assumed equal or unequal variance. |
