# Supplementary material for: Exposure to formaldehyde and asthma outcomes: A systematic review, meta-analysis, and economic assessment
Source: PLoS One. 2021 Mar 31;16(3):e0248258. doi: 10.1371/journal.pone.0248258 (PMC8011796; doi:10.1371/journal.pone.0248258)
Supplement: S73 Table — (DOCX) [file pone.0248258.s086.docx]

Supplemental Materials, Table 73. Characteristics of Schachter et al. 1987

| Bias domain | Authors’ judgment | Support for judgment |
| --- | --- | --- |
| Source population representation | Probably high | Very limited information is provided on inclusion/exclusion criteria for participants. Fifteen participants who responded to an advertisement to the University community and who had frequent work related exposure to formaldehyde were selected (10 females, 5 males). All were healthy and without signs of asthma. Subject characteristics and work location were presented. |
| Blinding | Low | The study had a random, double-blind design. |
| Outcome assessment | Low | Outcomes were measured by questionnaire, multiple pulmonary function tests, and a methacholine inhalation challenge (MIC) test and methods are described in detail. Pulmonary function was assessed using partial and maximal flow volume curves which were generated using a pneumotachograph integrator system that was calibrated daily. Airway resistance was measured by the interrupter method using an instrument that automatically averaged a series of six tidal breath values. Baseline airway reactivity was assessed by methacholine inhalation challenge. Subjects recorded their peak expiatory flow rate based on three attempts using a portable flow meter. Frequency and severity of irritative symptoms were self-reported using a symptom diary. Study was rated low risk of bias because objective measures (pulmonary function tests) used to determine outcomes. |
| Confounding | Low | The authors evaluated smoking status (Tier I), sex, age, height, weight, and asthma (Tier II), though the analyses do not appear to adjust for any covariates. Study was rated low risk of bias because this was an experimental design, where confounding should not be an issue because of the randomization. |
| Incomplete outcome data | Low | There were no missing data. |
| Exposure assessment | Probably low | Exposure was in a controlled environment and measurements were made by a modified NIOSH impinger method (Balmat and Meadows, 1985) utilizing a DuPont P-4000 constant flow sampler which sampled air at 1 liter/min for 15 min. Development of the samples was carried out using the chromotropic acid technique and spectrophotometry. In addition, ambient FA was measured using a hand-held Formaldemeter (MDA Scientific, Inc, Glenview, IL). Measurement methods were desribed in detail and validated methods were used. No QA/QC methods were described. |
| Selective outcome reporting | Low | Results are presented for all specified outcomes in the abstract and methods. |
| Conflict of interest | High | Researchers were based at Mt.Sinai hospital and Yale University and some funding was provided by the Formaldehyde Institute, Scarsdale, NY suggesting a high risk of bias. |
| Other sources of bias | Low | No other threats to internal validity were identified. Healthy worker bias is not discussed, however all study subjects were hospital workers in pathology, surgery and neuroanatomy departments (no external comparison group used). |
