# Supplementary material for: Exposure to formaldehyde and asthma outcomes: A systematic review, meta-analysis, and economic assessment
Source: PLoS One. 2021 Mar 31;16(3):e0248258. doi: 10.1371/journal.pone.0248258 (PMC8011796; doi:10.1371/journal.pone.0248258)
Supplement: S74 Table — (DOCX) [file pone.0248258.s087.docx]

Supplemental Materials, Table 74. Characteristics of Schenker et al. 1982

| Bias domain | Authors’ judgment | Support for judgment |
| --- | --- | --- |
| Source population representation | Low | No information was provided on recruitment or enrollment procedures. Participants in this study were selected from patients that lived in homes with urea formaldehyde foam insulation that self-referred to an occupational and environmental health clinic. Eighteen adults and six children were studied. No details regarding inclusion/exclusion criteria were reported. However, the recruitment was performed as a "consecutive sample", as it says that "We evaluated these systems in 24 consecutive subjects from six homes with UFFI, self-referred to an occupational and environmental health clinic." Therefore, the study was rated low risk of bias. |
| Blinding | Probably low | There is no mention of blinding of participants or study staff. Since the participants homes were tested for formaldehyde it seems likely that they were aware of possible exposure. However, only respiratory symptoms were self-reported (using a standardized American Thoracic Society questionnaire). Spirometry measures (and other outcomes assessed during the medical examination) are unlikely to be influenced by unblinded staff. |
| Outcome assessment | Low | The main outcomes of interest were spirometry, skin prick testing by standardized methods, and self-reported respiratory symptoms. All participants completed a standard medical history and the American Thoracic Society standard respiratory questionnaire. In addition skin prick testing and lung function tests were conducted (though no information is provided about the training of those conducting these tests). Given that most of the outcome assessment methods were not via self-report, there is a low risk of bias associated with outcome assessment methods. |
| Confounding | Probably high | Using the study questionnaire, authors measured some Tier I and Tier II variables including smoking, age, and sex. They did not measure SES or a proxy such as education. They also did not measure environmental variables that could impact formaldehyde concentration (temperature, humidity, etc.) or provide any information on other possible environmental co-exposures. Reviewers could not find where authors presented this data and it does not appear that any statistical analysis was conducted which accounts for confounders. |
| Incomplete outcome data | Probably high | Based on the results tables, it appears that some tests were not done for some participants and the reasons are not reported. (6 for skin test and 3 for bronchial challenge, (out of the 24 patients with UFFI exposure). Psychological testing was administered on 14 of the 18 adults. There is no information on why the tests were not performed in all study participants. |
| Exposure assessment | Probably high | Details regarding the methods used to conduct formaldehyde analyses in participants' homes are not provided. Authors note that measurements were made between 7-34 months following installation of UFFI; authors noted measured levels of formaldehyde in the study may not be representative of participant exposure since exposures can vary with humidity, temperature, and other sampling variables; authors noted none of the houses had exposures measured immediately following UFFI installation when formaldehyde concentrations can exceed 3 ppm. |
| Selective outcome reporting | Low | Results were presented for all the relevant outcomes specified. |
| Conflict of interest | Low | Funding for this study was from NIEHS and the Division of Lung Diseases, National Heart, Lung, and Blood Institute. Authors were employed by a medical school and hospital. Authors are affiliated with a university and/or hospital. |
| Other sources of bias | Probably low | No other threats to internal validity were identified. Authors noted this as a pilot study investigating health effects of residence in homes with UFFI, and information on health outcomes as well as measured exposures were gathered, however no statistical analysis of respiratory outcomes and formaldehyde exposures was reported. |
