# Supplementary material for: Exposure to formaldehyde and asthma outcomes: A systematic review, meta-analysis, and economic assessment
Source: PLoS One. 2021 Mar 31;16(3):e0248258. doi: 10.1371/journal.pone.0248258 (PMC8011796; doi:10.1371/journal.pone.0248258)
Supplement: S75 Table — (DOCX) [file pone.0248258.s088.docx]

Supplemental Materials, Table 75. Characteristics of Sheppard et al. 1984

| Bias domain | Authors’ judgment | Support for judgment |
| --- | --- | --- |
| Source population representation | Probably high | Seven non-smoking volunteer subjects with asthma and allergic rhinitis participated. Authors provide additional details in inclusion/exclusion criteria. Participants characteristics are provided in Table 1. It is unclear how the subjects were approached about participation in the study, or on the overall participation rates. |
| Blinding | High | Blinding was not discussed. Exposures were produced within a controlled laboratory/experimental chamber setting, and participants/investigators likely knew exposures. |
| Outcome assessment | Probably low | Outcomes were measured by spirometry. No details are provided on methods or the training of the person conducting the spirometry, but this is a more reliable method than self-report and there is no indication that exposure vs. non-exposure measurements would have differed. |
| Confounding | Low | All participants were non-smokers. There is no mention of SES. Authors measured sex, age, height. Study was rated as low because this was an experimental study design and so confounder adjustment is not necessary because of the randomization process. |
| Incomplete outcome data | Low | No missing outcome data reported. |
| Exposure assessment | Low | This was a controlled exposure and authors provide detailed methods on formaldehyde concentration generation and monitoring. Authors used a Miran infrared spectrophotometer to monitor formaldehyde levels. The spectrophotometer was calibrated daily by evaporating l-p1 injections of solutions of known formaldehyde concentrations (l-8 mg/ml) with a recirculating pump, and analyses corrected for the absorbance caused by water vapor at 6.42 m by evaporating a l-k,1 injection of distilled water. |
| Selective outcome reporting | Low | Results were presented for all the relevant outcomes specified. |
| Conflict of interest | Low | Authors were medical researchers supported by academic and government grants ( U.S. Public Health Service, the California Air Resources Board Contract AY-115-30, American Lung Association of California, the Academic Senate of the University of California, San Francisco, and the Pulmonary Faculty Training Grant HL-07159 from the National Heart, Lung, and Blood Institute). |
| Other sources of bias | Low | No other threats to internal validity were identified. |
