# Supplementary material for: Exposure to formaldehyde and asthma outcomes: A systematic review, meta-analysis, and economic assessment
Source: PLoS One. 2021 Mar 31;16(3):e0248258. doi: 10.1371/journal.pone.0248258 (PMC8011796; doi:10.1371/journal.pone.0248258)
Supplement: S76 Table — (DOCX) [file pone.0248258.s089.docx]

Supplemental Materials, Table 76. Characteristics of Smedje and Norback 2000

| Bias domain | Authors’ judgment | Support for judgment |
| --- | --- | --- |
| Source population representation | Probably low | Authors selected 40 public schools "randomly" and contacted principals to see whether they agree to participate. Participation rate was good (39). Self-administered questionnaire was mailed to pupils once in 1993 then as a follow-up in 1997. Questionnaire was mailed to 2,034 pupils and 85% (n=1732) returned. In 1997, questionnaire was sent to the pupils who had returned the survey and 78% responses (n=1347). Overall, total response rate was 66%. Some demographic information provided on demographics (sex, age, history of atopy, percent with furry pets, etc.). |
| Blinding | Probably low | Exposure was performed in schools a few months after the questionnaires had been returned in 1993, then again in 1995. For economic reasons, authors limited the number of measurements to classrooms used for theoretical lessons which they stated were the main sites of exposure. This selection of classrooms after pupils had returned the survey could suggest potential bias from investigators being aware of questionnaire results before obtaining exposure information--process may not have been blinded. |
| Outcome assessment | Probably high | Outcomes obtained from self-reported symptoms in a questionnaire. No mention of physician confirmation and no in-person interview by study investigators or mention of any follow up through phone, etc. No information provided on the validity of the questionnaires. |
| Confounding | Probably high | Authors adjusted for some Tier I confounders (age, and smoking) but not SES. Adjusted for some Tier II confounders (sex) but none of other confounders. |
| Incomplete outcome data | Low | Authors report outcome data for all children included in study (1476), and thoroughly explain participation rates, etc. |
| Exposure assessment | Probably low | Formaldehyde was measured with glass fibre filters impregnated with 2.4-dinitro-phenylhydrazine, with sampling for 4 hours at a rate of 0.2 l/min. The filters were analyzed by liquid chromatography. Method appears to be a standard, validated measure but no information provided on QA/QC of methods. For economic reasons, authors limited the number of measurements to classrooms used for theoretical lessons which they stated were the main sites of exposure. One potential issue is that only 51 of the 199 classrooms were visited during both 1993 and 1995. 1993 data potentially subset of data used in 2001 paper by same authors. |
| Selective outcome reporting | Low | Authors report outcome data for all children included in study (1476), and thoroughly explain participation rates, etc. |
| Conflict of interest | Low | No statement on COI, but all authors affiliated with academic institution (Uppsala university) and funded by government (Swedish council on work life research, county council of Uppsala) and health associations (Swedish association for asthma and allergy and Swedish society of medicine). |
| Other sources of bias | Low | No other risk of bias concerns. |
