# Supplementary material for: Exposure to formaldehyde and asthma outcomes: A systematic review, meta-analysis, and economic assessment
Source: PLoS One. 2021 Mar 31;16(3):e0248258. doi: 10.1371/journal.pone.0248258 (PMC8011796; doi:10.1371/journal.pone.0248258)
Supplement: S77 Table — (DOCX) [file pone.0248258.s090.docx]

Supplemental Materials, Table 77. Characteristics of Smedje and Norback 2001

| Bias domain | Authors’ judgment | Support for judgment |
| --- | --- | --- |
| Source population representation | Probably low | Authors randomly selected 40 public schools in Uppsala. 39 headmasters agree to participate. 615 first grade pupils, 657 4th grade and 762 7th grade students were invited to participate (2034 total). Those who participated in follow-up did not differ between 1997 and 1993 in terms of age, sex, history of atopy, asthma, pollen, pet allergy, or smoking habits. |
| Blinding | Probably low | Exposure was performed in schools a few months after the questionnaires had been returned. In each school, authors chose only a few classrooms to measure exposures, and stated these were the main sites of exposure at school. This selection of classrooms after pupils had returned the survey could suggest potential bias from investigators being aware of questionnaire results before obtaining exposure information--process may not have been blinded. |
| Outcome assessment | Probably high | Outcomes obtained from self-reported symptoms in a questionnaire. No mention of physician confirmation and no in-person interview by study investigators or mention of any follow up through phone, etc. No information provided on the validity of the questionnaires. |
| Confounding | Probably high | Authors adjusted for some Tier I confounders (age, and smoking) but not SES. Adjusted for some Tier II confounders (sex) but none of other confounders. Additionally adjusted for history of atopy. |
| Incomplete outcome data | Low | Authors report outcome data for all children included in study (1347), and thoroughly explain participation rates, etc. |
| Exposure assessment | Probably low | Formaldehyde was measured with glass fibre filters impregnated with 2.4-dinitro-phenylhydrazine, with sampling for 4 hours at a rate of 0.2 l/min. The filters were analyzed by liquid chromatography. Method appears to be a standard, validated measure but no information provided on QA/QC of methods. In each school, authors chose classrooms that represented "main sites of exposure" for students; a total of 98 classrooms were measured in 1993 and 101 in 1995. For this study, authors combined measurements taken in 1993 and 1995, even though previous paper details how new ventilation system was installed after 1993 and before 1995. However, since the study isn't designed to look at this issue, this is likely not problematic. |
| Selective outcome reporting | Low | Authors report results for all outcomes outlined in methods section. |
| Conflict of interest | Low | No statement on COI, but all authors affiliated with academic institution (Uppsala university) and funded by government (Swedish council on work life research, county council of Uppsala) and health associations (Swedish association for asthma and allergy and Swedish society of medicine). |
| Other sources of bias | Low | No other risk of bias concerns. |
