# Supplementary material for: Exposure to formaldehyde and asthma outcomes: A systematic review, meta-analysis, and economic assessment
Source: PLoS One. 2021 Mar 31;16(3):e0248258. doi: 10.1371/journal.pone.0248258 (PMC8011796; doi:10.1371/journal.pone.0248258)
Supplement: S78 Table — (DOCX) [file pone.0248258.s091.docx]

Supplemental Materials, Table 78. Characteristics of Smedje et al. 1997

| Bias domain | Authors’ judgment | Support for judgment |
| --- | --- | --- |
| Source population representation | Probably low | Authors randomly selected 39 schools out of ~130 public schools in Uppsala. All headmasters agree to participate. Each school had between 350-600 pupils. Little information on demographic makeup of participants provided. Year of questionnaire was 1993. Possible that the collected information is a subset of data used in 2000 and 2001 papers by same authors. |
| Blinding | Probably low | Exposure was performed in schools a few months after the questionnaires had been returned. In each school, authors chose 2-3 classrooms "used frequently" by the selected classes and located in the different building; a total of 28 classrooms were investigated. This selection of classrooms after pupils had returned the survey could suggest potential bias from investigators being aware of questionnaire results before obtaining exposure information--process may not have been blinded. |
| Outcome assessment | Probably high | Outcomes obtained from self-reported symptoms in a questionnaire. No mention of physician confirmation and no in-person interview by study investigators or mention of any follow up through phone, etc. No information provided on the validity of the questionnaires. |
| Confounding | High | Authors state in Table 5 that these were regression models "controlling for personal factors", but no explicit mention what these factors were. In the text, authors mention that there was controlling for students with atopic disposition, or food allergy, or who had attended a day care center for several years. It is unclear whether these are the controlling factors. |
| Incomplete outcome data | Low | Authors report outcome data for all children included in study (627), and thoroughly explain participation rates, etc. |
| Exposure assessment | Probably low | Formaldehyde concentrations were measured with glass fibre filters impregnated with 2,4-dinitro-phenylhydrazine using a pump and a sampling rate of 0.2 L/min for 4 h. The filters were analyzed by liquid chromatography. Method appears to be a standard, validated measure but no information provided on QA/QC of methods. In each school, authors chose 2-3 classrooms "used frequently" by the selected classes and located in the different building; a total of 28 classrooms were investigated. |
| Selective outcome reporting | Low | Authors report results for all outcomes outlined in methods section. |
| Conflict of interest | Low | No statement on COI, but all authors affiliated with academic institution (Uppsala university) and funded by government (Swedish council on work life research, county council of Uppsala) and health associations (Swedish association for asthma and allergy and Swedish society of medicine). |
| Other sources of bias | Low | No other risk of bias concerns. |
