# Supplementary material for: Exposure to formaldehyde and asthma outcomes: A systematic review, meta-analysis, and economic assessment
Source: PLoS One. 2021 Mar 31;16(3):e0248258. doi: 10.1371/journal.pone.0248258 (PMC8011796; doi:10.1371/journal.pone.0248258)
Supplement: S79 Table — (DOCX) [file pone.0248258.s092.docx]

Supplemental Materials, Table 79. Characteristics of Tavernier et al. 2006

| Bias domain | Authors’ judgment | Support for judgment |
| --- | --- | --- |
| Source population representation | Probably high | Recruitment took place as part of larger community epidemiologic study. All participants were patients at one of 2 neighboring primary care facilities in the South of Manchester and were exposed to the same geographic conditions in outdoor pollution. Out of 435 eligible children following response to validated screening questionnaire, authors noted almost a quarter (102) of households refused to take part in the study. Due to matching restrictions only 200 children completed the whole sampling process. However, refusal to participate followed identification of matched pairs. A resulting 90 matched homes were finally analyzed. Subjects’ characteristics are not compared between those who chose to participate and those who refused. |
| Blinding | Probably low | Exposures were measured at same time as administration of the questionnaire assessing outcomes. Authors noted that exposure assessment visits were conducted. This was a large study monitoring for many types of exposures, so it is unlikely that participants would have been aware of formaldehyde exposure. |
| Outcome assessment | Probably low | Asthmatic children and matched healthy control subjects were recruited according to their responses to a screening questionnaire validated against a physician diagnosis of asthma. The validation process and positive predictive value (84%) of questionnaire responses were reported previously. Study rated probably low risk of bias because asthma diagnosis confirmed by medical history, not objective testing. Based on description, assume both groups were asked the same questions. |
| Confounding | Probably low | All participants were from a region with similar socioeconomic status and household smoking was measured. Cases were age-, sex-, and sibship size–matched to nonasthmatic children. Multiple environmental co-exposures were evaluated. The study was rated probably low risk of bias because the authors conducted a matched study, and they collected the relevant information. However, they state that they controlled for important confounders, but they don't list what they are. |
| Incomplete outcome data | Low | Results appear complete for the 200 children ultimately included in final analyses. |
| Exposure assessment | Probably low | The formaldehyde–2,4-dinitrophenyl hydrazine derivative was extracted and analyzed by means of HPLC (Hewlett Packard Series 1100) using an ASTM method. Other details, including QA/QC methods, are not reported. |
| Selective outcome reporting | Low | Results were presented for all the relevant outcomes specified. |
| Conflict of interest | Low | Authors were from hospital and research centers. The study was funded by a grant from the North West RD directorate. |
| Other sources of bias | Low | No other threats to internal validity were identified. |
