# Supplementary material for: Exposure to formaldehyde and asthma outcomes: A systematic review, meta-analysis, and economic assessment
Source: PLoS One. 2021 Mar 31;16(3):e0248258. doi: 10.1371/journal.pone.0248258 (PMC8011796; doi:10.1371/journal.pone.0248258)
Supplement: S80 Table — (DOCX) [file pone.0248258.s093.docx]

Supplemental Materials, Table 80. Characteristics of Tuomainen et al. 2013

| Bias domain | Authors’ judgment | Support for judgment |
| --- | --- | --- |
| Source population representation | Probably high | Two blocks of apartment buildings located next to each other in a suburban area were investigated. The case building was constructed according to the instructions of the Finnish Classification, while the control building was conventionally constructed. Little information is provided on inclusion/exclusion criteria for selection of buildings, or on possible differences in the populations inhabiting these buildings. The response rate for the survey varied from 63-89% during the follow-up for the control building. The response rate for the survey varied from 89-100% during the follow-up for the case building, and authors note that mainly the occupants with respiratory diseases answered the questions. |
| Blinding | Probably high | There is no discussion of blinding. It is likely that the residents in the case building knew that there were some differences between their building and others and outcomes were measured by self-report via questionnaire. |
| Outcome assessment | Probably low | Symptoms were self-reported on five occasions using a questionnaire modified from the MM40 and Tuohilampi questionnaire series specially designed to characterize respiratory symptoms and diseases. There is no information on validity/standardization of the questionnaire. Study rated probably low risk of bias because asthma diagnosis confirmed by medical history, not objective testing. Based on description, assume both groups were asked the same questions. |
| Confounding | Probably high | The study examined passive smoking (Tier I), and several other factors including stuffy air, dry air, varying temperature, and other factors (Tier II). Results are presented where people are compared to before and after moving into a particular building (no assessment of confounding required) and the other data are for comparing cases and controls. Study authors collected relevant information (e.g. smoking), but it is not stated that it is used in an analysis to control for the potential for confounding. |
| Incomplete outcome data | Probably high | There is limited discussion of incomplete or missing data and how this may have affected results. |
| Exposure assessment | Probably high | Indoor air quality measurements were made annually for the first three years of occupancy, but were carried out at different times of year because the buildings were not completed at the same time. Samples were collected for 2-4 hours in one apartment per floor (six per building) in the living room and bedroom using dinitrophenylhydrazine cartridges and analyzed by HPLC with diode array detector. No QA/QC methods were provided. |
| Selective outcome reporting | Low | Results were reported for all outcomes specified in the abstract and methods. |
| Conflict of interest | Low | The study was funded by government and academic organizations, and all authors are affiliated with academic or government organizations. |
| Other sources of bias | Low | No additional threats to internal validity were identified. |
