# Supplementary material for: Exposure to formaldehyde and asthma outcomes: A systematic review, meta-analysis, and economic assessment
Source: PLoS One. 2021 Mar 31;16(3):e0248258. doi: 10.1371/journal.pone.0248258 (PMC8011796; doi:10.1371/journal.pone.0248258)
Supplement: S81 Table — (DOCX) [file pone.0248258.s094.docx]

Supplemental Materials, Table 81. Characteristics of Tuthill 1984

| Bias domain | Authors’ judgment | Support for judgment |
| --- | --- | --- |
| Source population representation | Probably low | Kindergarten through 6th grade student households from a public school system in Western Massachusetts were randomly selected. Symptoms were recorded for the youngest child in the household. Out of 436 households, 399 or 91.5 percent had completed interviews. Regarding wood stove exposures, authors note that there may have been some selection bias in that wood stoves were less likely to be installed when child in the household had chronic respiratory problems. |
| Blinding | Probably low | Outcomes were reported via questionnaire given to parents of selected children. There was no discussion of blinding, and parents were likely aware of respiratory health status and exposure factors in their households, but may not be likely to bias results. |
| Outcome assessment | Probably low | Outcomes of acute respiratory infections, chronic bronchitis, asthma, and allergies were self-reported by parents using the questionnaire. The study was rated probably low risk of bias because asthma diagnosis confirmed by medical history, not objective testing. Based on description, assume both groups were asked the same questions. |
| Confounding | Probably high | Authors measured smoking, SES and education. They measured numerous Tier II confounders including age, sex, number of siblings, and possible co-exposures (woodstove) and modifying factors. They state that no control variables were related to formaldehyde exposure and also to acute or chronic respiratory illness. Controlling for woodstove use did not diminish the formaldehyde effect. The study was rated probably high risk of bias because authors collected this information on confounders, but there is no explanation in the text about how the authors accounted for potential confounders. |
| Incomplete outcome data | Low | Results are complete for the 399 study participants. |
| Exposure assessment | Probably high | Formaldehyde exposure was included in the study as a control variable and was indirectly measured by an a priori combination of the following variables: new construction or remodeling since September, new upholstered furniture since September, foam insulation in walls, living in a mobile home, as well as presence of wood stove as exposure proxy. Authors noted exposure was not validated by in-home measurements. |
| Selective outcome reporting | Low | Results were presented for all the relevant outcomes specified. |
| Conflict of interest | Probably low | Author was an academic researcher. Information on study funding was not provided, and there is no reason to suspect potential COI. |
| Other sources of bias | Low | No other threats to internal validity were identified. |
