# Supplementary material for: Exposure to formaldehyde and asthma outcomes: A systematic review, meta-analysis, and economic assessment
Source: PLoS One. 2021 Mar 31;16(3):e0248258. doi: 10.1371/journal.pone.0248258 (PMC8011796; doi:10.1371/journal.pone.0248258)
Supplement: S82 Table — (DOCX) [file pone.0248258.s095.docx]

Supplemental Materials, Table 82. Characteristics of Uba et al. 1989

| Bias domain | Authors’ judgment | Support for judgment |
| --- | --- | --- |
| Source population representation | Probably low | 103 medical students from a single class were recruited. A summary of characteristics for the study group was provided. |
| Blinding | Probably high | There is no evidence of blinding, and participants were likely aware of their exposure. |
| Outcome assessment | Low | Respiratory symptoms were self-reported using respiratory questionnaires for persistent and acute symptoms. The pulmonary function of each subject was measured using the greatest value of acceptable spirometer values. Spirometry was performed by trained technicians according to the American Thoracic Society Snowbird Workshop recommendations. Three spirometers were used, and all tests for each subject were done on the same machine. The spirometer was calibrated at the start and end of each testing session. The study was rated low risk of bias because used objective measures (pulmonary function tests) to determine outcomes. |
| Confounding | Low | The study examined cigarette consumption (Tier I), sex, age, height, ethnicity, and history of asthma (Tier II). SES was not addressed. The study was rated low risk of bias because it was somewhat of an experimental design where outcome was measured before and after exposure (anatomy lab). So each person served as their own control. |
| Incomplete outcome data | Low | 81/103 students completed both surveys, and complete spirometry data was available for 96/103 students. Follow up data was presented for all students who participated. |
| Exposure assessment | Low | 32 samples were collected using personal sampling devices (impingers) located in the breathing zone of the students. Samples were analyzed by spectrophotometry according to NIOSH method 3500. 16 short-term exposures were measured using a portable infrared spectrophotometer. The detection limit was 0.05 ppm. |
| Selective outcome reporting | Low | Results are reported for all specified outcomes in the abstract and methods. |
| Conflict of interest | Probably low | All authors are affiliated with academic institutions. No information provided on financial COI. |
| Other sources of bias | Low | No other threats to internal validity were identified. |
