# Supplementary material for: Exposure to formaldehyde and asthma outcomes: A systematic review, meta-analysis, and economic assessment
Source: PLoS One. 2021 Mar 31;16(3):e0248258. doi: 10.1371/journal.pone.0248258 (PMC8011796; doi:10.1371/journal.pone.0248258)
Supplement: S83 Table — (DOCX) [file pone.0248258.s096.docx]

Supplemental Materials, Table 83. Characteristics of Venn et al. 2003

| Bias domain | Authors’ judgment | Support for judgment |
| --- | --- | --- |
| Source population representation | Probably low | This case control study was performed on children in their first and second years of primary school (6-8 years old) who participated in a study of traffic pollution exposure and childhood wheezing illness in 1995/1996 and self-reported wheezing in that study. Cases reported wheeze in the previous study and in the current study; controls reported wheeze only in the prior study. Authors presented comparisons in tables between sample population and overall cohort and case/controls and sample population, there were no significant differences noted other than there was a difference in highest two classes of SES among participants than non-participants (deferential in level) in both cases and controls. However, SES was adjusted for in multivariate models. |
| Blinding | Low | The authors note that the laboratories analyzing the exposure measures were blinded to the health status of participants. |
| Outcome assessment | Probably low | Case status was reported by parent (wheezing in the past year). Case status was confirmed via peak flow meter to record the best of three blows for morning and evening peak flows, and completed a diary with night-time and daytime symptom scores. Illness status was validated by medical records in a subset of cases when additional consent was given. In addition, some analyses were limited to validated cases and controls. Study rated probably low risk of bias because asthma diagnosis confirmed by medical testing, not objective measures. Based on description, assume both groups were asked the same questions. |
| Confounding | Probably high | The model was adjusted for age, sex, and socioeconomic factors. However, authors did not adjust for parental smoking though they measured cotinine (they did not adjust for the cotinine). They report there was 'no reported different' in rate of smoking in cases/controls homes. However, cotinine levels were different between cases and controls. |
| Incomplete outcome data | Low | There was 10% missing exposure data for both cases and controls. However, follow up results were presented for all participants that consented to participate in the home visits. |
| Exposure assessment | Probably low | The authors used diffusive passive samplers to measure formaldehyde over a three day period in the center the kitchen. Formaldehyde samplers were solvent extracted and analyzed by HPLC. No QA/QC processes are described. |
| Selective outcome reporting | Probably low | The authors note that other outcomes were investigated but that their analyses were not presented in this paper. It is unclear if those outcomes are of interest (i.e. respiratory). Results are presented for all outcomes outlined in the abstract and methods. |
| Conflict of interest | Low | The study was funded by a governmental agency and authors all have academic affiliations. |
| Other sources of bias | Low | No other threats to validity were identified. |
