# Supplementary material for: Exposure to formaldehyde and asthma outcomes: A systematic review, meta-analysis, and economic assessment
Source: PLoS One. 2021 Mar 31;16(3):e0248258. doi: 10.1371/journal.pone.0248258 (PMC8011796; doi:10.1371/journal.pone.0248258)
Supplement: S84 Table — (DOCX) [file pone.0248258.s097.docx]

Supplemental Materials, Table 84. Characteristics of Veremchuk et al. 2016

| Bias domain | Authors’ judgment | Support for judgment |
| --- | --- | --- |
| Source population representation | Probably low | Asthma cases were identified from a federal statistical observation (form No. 12) of municipal clinics in Vladivostok from 2008-2012. All the cases of acute illnesses and the first visit in year on the exacerbation of chronic disease (per 100,000 people) were included in the study. |
| Blinding | Low | The study was effectively blinded because monitoring data was obtained from the Federal Service for Hydrometeorology and Environmental Monitoring, and asthma morbidity was evaluated by data obtained from the federal data of physician-reported morbidities. |
| Outcome assessment | Probably low | Asthma was originally determined by a physician, which may or may not include objective measures. |
| Confounding | High | The study evaluated effects by age groups (children, adolescents, and adults). Climatic factors and indicators of anthropogenic air pollution in the city were also evaluated. No other potential confounding factors were examined. |
| Incomplete outcome data | Probably high | Study rated probably high risk of bias because reviewers could not identify where authors presented the number of patients analyzed, so it is difficult to tell if there is missing data. |
| Exposure assessment | Probably high | Long-term monitoring data on air quality from 2008–2012 was conducted by the Primorsky Department of Hydrometeorology and Environmental Monitoring and Center of Hygiene and Epidemiology of Primorsky Krai. Formaldehyde (mg/m3 ) levels (and other pollutants) were measured in six stationary observation posts in addition to climatic conditions. These were area based measurements but there is insufficient information on spatial resolution or modeling to determine if this was an adequate way to measure exposure of individuals. |
| Selective outcome reporting | Low | Results are presented for all outcomes specified in the abstract and methods. |
| Conflict of interest | Low | Study authors are from medical and government institutions and state they have no competing financial interests. Funding was provided by a grant from the Russian Science Foundation. |
| Other sources of bias | Low | No other threats to validity were identified. |
