# Supplementary material for: Exposure to formaldehyde and asthma outcomes: A systematic review, meta-analysis, and economic assessment
Source: PLoS One. 2021 Mar 31;16(3):e0248258. doi: 10.1371/journal.pone.0248258 (PMC8011796; doi:10.1371/journal.pone.0248258)
Supplement: S85 Table — (DOCX) [file pone.0248258.s098.docx]

Supplemental Materials, Table 85. Characteristics of Wieslander et al. 1997

| Bias domain | Authors’ judgment | Support for judgment |
| --- | --- | --- |
| Source population representation | Low | A screening questionnaire was mailed to a random sample of 3600 men and women aged 20—44 years, selected from the population register of Uppsala (popluation 160,000). All symptomatic responders (n=216) plus another random sample of 800 subjects were selected for further examination at the Department of Lung Medicine during the period April 1991 to February 1992. To enrich the random sample (n=800), all persons from the main sample who had not been selected to the random sample, and who in the postal questionnaire reported use of asthma medication, attacks of asthma or awakening because of shortness of breath, were invited to participate (n=216). The random sample therefore also included some symptomatic subjects. The total response rate in the initial self-administered questionnaire survey was 87%. The response rate in the clinical investigation was 68% among the random sample of 800 subjects, and 83% among the additional 216 symptomatic subjects. There were no significant differences in age, sex, or smoking between those that did and did not participate in the clinical study. |
| Blinding | Low | The authors note that information from the exposure measurements and the questionnaire on exposure were not linked with medical information until all data collection was completed. |
| Outcome assessment | Low | Lung function tests were performed using a spirometer (FEV1) and a peak flow meter (PEF). PEF was recorded twice daily for one week, each time using the best of three measurements. Methacholine challenge was performed using a MEFAR inhalation dosimeter. Symptoms were self-reported using a modified version of the IUATLD questionnaire. |
| Confounding | Probably low | Authors assessed smoking exposure, but did not evaluate SES. Authors measured several Tier II confounders including age, sex, and other environmental exposures. |
| Incomplete outcome data | Low | There was no missing data for outcomes associated with domestic exposures. Outcomes associated with workplace exposures were limited to 464/562 responders, but workplace formaldehyde was not measured. |
| Exposure assessment | Probably low | Formaldehyde was measured in the bedroom of 62/72 randomly selected homes from the symptomatic group and 80 randomly selected homes from the non-symptomatic group. Samples were collected with filters with an air sampling rate of 0.25 l/min for 2 hours, and were analyzed by liquid chromatography. No QA/QC methods were described. |
| Selective outcome reporting | Low | Results are reported for all outcomes specified in the abstract and methods. |
| Conflict of interest | Probably high | All authors were affiliated with academic institutions. The study was funded by the Swedish Association against Asthma and Allergy, The Swedish Medical Research Council, The Swedish Society of Medicine, The Swedish Heart and Lung Foundation, The Bror Hjerpstedts Foundation, Pharmacia Diagnostics, and the County Council of Uppsala. Study rated probably high because Pharmacia is a for profit company that would not be supporting a study unless they had a financial interest in the outcome - based on their history it is possible they make asthma drugs or potentially are involved with formaldehyde - they were bought by Monsanto after this study was done - so there is insufficient info as to what the bias might be specifically. |
| Other sources of bias | Low | No other threats to internal validity were identified. |
