# Supplementary material for: Exposure to formaldehyde and asthma outcomes: A systematic review, meta-analysis, and economic assessment
Source: PLoS One. 2021 Mar 31;16(3):e0248258. doi: 10.1371/journal.pone.0248258 (PMC8011796; doi:10.1371/journal.pone.0248258)
Supplement: S86 Table — (DOCX) [file pone.0248258.s099.docx]

Supplemental Materials, Table 86. Characteristics of Witek, Jr et al. 1986

| Bias domain | Authors’ judgment | Support for judgment |
| --- | --- | --- |
| Source population representation | Probably high | Participants were selected from a self-selected group of people that responded to an advertisement. This included 9 men, 5 women and some asthmatics with an average age of mid-twenties. No additional details are provided. |
| Blinding | Low | This was a randomized, double-blind crossover study with tests performed in random sequence. |
| Outcome assessment | Low | Participants filled out symptom questionnaires upon entering the chamber and after exposure. Pulmonary function tests were also performed at multiple time points post- exposure and detailed methods are provided. Asthmatics were defined through the questionnaire as defined by the American Thoracic Society. |
| Confounding | Low | Study participants were all nonsmokers and asthmatics were included. Age and gender were recorded. SES or similar measures were not recorded. Participants were randomized to interventions. |
| Incomplete outcome data | Probably low | Study rated probably low risk of bias because in table 4, there is a notation for some outcomes that only 14 subjects reporting, but no explanation for why. |
| Exposure assessment | Low | This study used a controlled exposure in a temperature and humidity controlled chamber. Multiple methods were used to test and confirm the concentration of formaldehyde including a modified NIOSH impinger method using a DuPont P-4000 constant flow sampler which sampled air at 1 L/min for 15 min with samples measured by spectrophotometer. In addition samples were measured using a handheld formaldemeter. |
| Selective outcome reporting | Low | Results were presented for all the relevant outcomes specified. |
| Conflict of interest | Probably low | Authors were affiliated with a hospital, a research institution, and a non-profit research institute, though no details were provided on funding for this study. |
| Other sources of bias | Low | No other threats to internal validity were identified. |
