# Supplementary material for: Exposure to formaldehyde and asthma outcomes: A systematic review, meta-analysis, and economic assessment
Source: PLoS One. 2021 Mar 31;16(3):e0248258. doi: 10.1371/journal.pone.0248258 (PMC8011796; doi:10.1371/journal.pone.0248258)
Supplement: S87 Table — (DOCX) [file pone.0248258.s100.docx]

Supplemental Materials, Table 87. Characteristics of Witek, Jr et al. 1987

| Bias domain | Authors’ judgment | Support for judgment |
| --- | --- | --- |
| Source population representation | High | No information is provided about inclusion/exclusion criteria, source population, or demographic or other characteristics. Fifteen asthmatic volunteers were included. |
| Blinding | Low | Randomized double-blind experimental protocol. |
| Outcome assessment | Low | Outcomes were measured by self-report, pulmonary function tests, metacholine challenge. Information on training of staff conducting testing was not provided. All 15 asthma subjects met the American Thoracic Society definition of asthma. Study was rated low risk of bias because outcome assessed using objective measures (pulmonary function tests). |
| Confounding | Probably low | None of the subjects smoked, all were between age 18-35, all subjects refrained from taking asthma medications, caffeinated beverages for at least 24 hours prior to session, and none reported having an upper respiratory infection during the study. Authors also report gender, height and weight. This is a controlled exposure study, hence co-exposure measurement was not necessary. |
| Incomplete outcome data | Low | No missing outcome data were reported. |
| Exposure assessment | Low | The study used a controlled exposure in a temperature and humidity controlled chamber. Multiple methods were used to test and confirm the concentration of formaldehyde including a modified NIOSH impinger method using a DuPont P-4000 constant flow sampler which sampled air at 1 L/min for 15 min with samples measured by spectrophotometer. In addition samples were measured using a handheld formaldemeter. Nonexposure areas (outside chamber) were also tested for formaldehyde. Authors noted formaldehyde was undetectable in chamber by both methods during room air sessions. Furthermore, no formaldehyde was detected in the surrounding halls and labs during exposure days. |
| Selective outcome reporting | Low | Results were presented for all the relevant outcomes specified. |
| Conflict of interest | High | Authors were researchers from academic and medical institutions. Study funding was in part provided by a contract with the Formaldehyde Institute, Scarsdale, NY. |
| Other sources of bias | Low | No other threats to internal validity were identified. |
