# Supplementary material for: Exposure to formaldehyde and asthma outcomes: A systematic review, meta-analysis, and economic assessment
Source: PLoS One. 2021 Mar 31;16(3):e0248258. doi: 10.1371/journal.pone.0248258 (PMC8011796; doi:10.1371/journal.pone.0248258)
Supplement: S88 Table — (DOCX) [file pone.0248258.s101.docx]

Supplemental Materials, Table 88. Characteristics of Yeatts et al. 2012

| Bias domain | Authors’ judgment | Support for judgment |
| --- | --- | --- |
| Source population representation | Low | A nationally representative sample was recruited following a two stage-cluster sample design. The recruitment protocol was well-documented. Response rate was 75% for selected households. |
| Blinding | Probably low | During the second visit family members were interviewed and the two independent researchers read the diffusive tubes onsite. Participants were likely not aware of their exposures since measured in their home. There is no information on whether the interviewer who asked about health status participated in air sampling readings. However placement of sampling equipment would not have been biased on health status of family members. |
| Outcome assessment | Probably low | Symptoms were self-reported using Arab translations of questions that were taken from NHANES, ISAAC (International Study of Asthma and Allergies in Childhood) and BRFSS (Behavioral Risk Factor Surveillance System questions) questionnaires (all validated and acknowledged as reliable). Asthma question was posed as ever having doctor-diagnosed asthma. Authors report that questions were translated from English to Arab and back to English and reviewed by a researcher for accuracy, cultural appropriateness and correct local terminology. For all interviews involving children, responses were obtained from the mother. Study was rated probably low risk of bias because asthma diagnosis confirmed by medical history, not objective testing. Based on description, assume both groups were asked the same questions. |
| Confounding | Low | All Tier I (age, education level, household smoking exposure) and some Tier II confounders were primarily measured by questionnaire. Modeling techniques evaluated influence of outliers reduce leverage of extreme values. |
| Incomplete outcome data | Probably low | There was 10% missing data for some symptoms (ever had wheezing, ever doctor-diagnosed asthma), but about 90% missing data for other symptoms (wheezing in last 12 months or 4 weeks, speech-limiting wheezing). However, the authors state that the high numbers of missing are due to skip patterns for some symptoms. |
| Exposure assessment | Low | Passive samplers were used over a 7-day period and read independently by two field interviewers. Randomly selected duplicate diffusion tubes were used in each house for quality control. A limit of quantification of 0.006 ppm was reported. |
| Selective outcome reporting | Low | Results presented for all outcomes outlined in the abstract and methods. |
| Conflict of interest | Low | The study was funded by government agencies and the authors declare that they have no actual or potential competing financial interests. |
| Other sources of bias | Low | No other threats to internal validity were identified. |
