# Supplementary material for: Exposure to formaldehyde and asthma outcomes: A systematic review, meta-analysis, and economic assessment
Source: PLoS One. 2021 Mar 31;16(3):e0248258. doi: 10.1371/journal.pone.0248258 (PMC8011796; doi:10.1371/journal.pone.0248258)
Supplement: S89 Table — (DOCX) [file pone.0248258.s102.docx]

Supplemental Materials, Table 89. Characteristics of Yoon and Lin 2014

| Bias domain | Authors’ judgment | Support for judgment |
| --- | --- | --- |
| Source population representation | Probably low | 887 participants were selected from all grades of 2 elementary schools, and 814 (91.8%) participated. Little information is provided on inclusion/exclusion criteria for the schools. |
| Blinding | Probably low | Blinding is not addressed. Participants were likely not aware of exposures in the home. It was unclear whether exposure assessor was blinded to the exposure. |
| Outcome assessment | Probably low | Outcome was measured by questionnaires filled out by parents. Asthma status was measured by self report of asthma status as diagnosed by a physician. |
| Confounding | Low | Researchers evaluated both Tier I confounders (smoking and SES) and many Tier II confounders including age, gender, height, weight, and demographic, socioeconomic, and residential environmental conditions. |
| Incomplete outcome data | Low | No missing outcome data reported for the study sample. |
| Exposure assessment | Low | For measurements of formaldehyde, a passive sampler (3M 3721, 3M, Ontario, Canada) was used and instructions for personal sampling for approximately 3 working days (72 hours). The collection media for formaldehyde were 3M Model 3721 formaldehyde diffusion monitors, designed to measure the time-weighted average concentration of formaldehyde gas. Formaldehyde samples were analyzed by UV spectrophotometer (UV mini 1240; Shimadzu, Japan) at 580 nm, in accordance with NIOSH (National Institute for Occupational Safety and Health) method 3500 (NIOSH, 1994). |
| Selective outcome reporting | Low | Results were presented for all the relevant outcomes specified. |
| Conflict of interest | Probably low | Author is an academic researcher. No information on funding for the work is provided, but there is no reason to suspect potential COI. |
| Other sources of bias | Low | No other threats to internal validity were identified. |
