# Supplementary material for: Exposure to formaldehyde and asthma outcomes: A systematic review, meta-analysis, and economic assessment
Source: PLoS One. 2021 Mar 31;16(3):e0248258. doi: 10.1371/journal.pone.0248258 (PMC8011796; doi:10.1371/journal.pone.0248258)
Supplement: S90 Table — (DOCX) [file pone.0248258.s103.docx]

Supplemental Materials, Table 90. Characteristics of Zammit-Tabona et al. 1983

| Bias domain | Authors’ judgment | Support for judgment |
| --- | --- | --- |
| Source population representation | Probably high | 9 of 12 workers from one foundry with bronchial hyperreactivity and respiratory symptoms compatible with asthma and 2 workers from another foundry who were referred by their family doctors were included in the study (11 total). All had occupational exposure to diphenylmethane diisocyanate (MDI). Two eligible workers did not take part. Reasons for inclusion/exclusion are not provided. |
| Blinding | High | This is a chamber study and no discussion of blinding, so may have been aware of exposures. Participants wore noseclips during the formaldehyde challenge though it is unclear if they also did this during the control portion. Lung function was measured by spirometry, but there is no information on who performed the lung function tests. |
| Outcome assessment | Low | Lung function was measured with spirometry, and methods are described in detail. Other outcomes were obtained by standard skin prick tests. |
| Confounding | Probably low | Researchers considered the Tier I confounder of smoking, but did not evaluate SES. They measured some Tier II confounders such as age, other environmental exposures, and additional factors that may be important such as atopic status. |
| Incomplete outcome data | Low | No missing outcome data were reported. |
| Exposure assessment | Probably low | Formaldehyde was measured on one occasion only and no information is provided about measurement methods. Study was rated probably low because there was controlled exposure that was the same for everyone (2.5 ppm) though only measured one time. |
| Selective outcome reporting | Low | Results were presented for all the relevant outcomes specified. |
| Conflict of interest | Low | Researchers were from academica and funding for this study came from the British Columbia Lung Association. |
| Other sources of bias | Probably high | Authors recruited subjects with respiratory symptoms (12 workers with symptoms out of 78). Exposure began approx 4 years earlier. Some of the most affected could have left the job after exposure began and the authors recruited workers. |
