# Supplementary material for: Exposure to formaldehyde and asthma outcomes: A systematic review, meta-analysis, and economic assessment
Source: PLoS One. 2021 Mar 31;16(3):e0248258. doi: 10.1371/journal.pone.0248258 (PMC8011796; doi:10.1371/journal.pone.0248258)
Supplement: S91 Table — (DOCX) [file pone.0248258.s104.docx]

Supplemental Materials, Table 91. Characteristics of Zhai et al. 2013

| Bias domain | Authors’ judgment | Support for judgment |
| --- | --- | --- |
| Source population representation | Low | 186 residential houses in the main urban area in Shenyang were selected as survey objects. Selection of survey houses was carried out strictly according to the “Code for indoor environmental pollution control of civil building engineering” (GB50325-2001). The houses had been decorated in the previous four years, and occupied within the previous three years. Other recruitment methods were not reported. |
| Blinding | Low | Blinding is not addressed. However exposure assessment used active area instrument monitoring and outcomes were determined using American Thoracic Society/National Heart and Lung Institute questionnaires. There is no reason to believe that participants were aware of their level of formaldehyde exposure. |
| Outcome assessment | Low | Symptoms were self-reported using a questionnaire designed and recommended by the American Thoracic Society/Children Questionnaire. One adult was selected randomly from each house to answer the questionnaire, as were 82 children (helped by their parents). However diagnoses were not confirmed by a physician. |
| Confounding | Low | Analysis accounted for smoking in the family, and SES (education status/occupation) (Tier I), and several other factors such as age, gender, height, and weight (Tier II). |
| Incomplete outcome data | Low | There was no apparent missing data. |
| Exposure assessment | Low | Formaldehyde was monitored in the bedrooms, living rooms, and kitchens. Before sampling, the doors and windows of the surveyed rooms were closed for at least 12 h. Formaldehyde was tested for by the acetylacetone-absorption-spectrometry method according to GB50325-2001. 558 samples were collected from 186 houses. The houses were divided into groups of polluted or non-polluted houses; the polluted house group contained houses that tested positive for formaldehyde concentrations in excess of the hygienic standard (0.08 mg/m3). No QA/QC details were provided. |
| Selective outcome reporting | Low | Results are reported for all outcomes specified in the abstract and methods. |
| Conflict of interest | Low | The authors have academic affiliations and declare that there is no conflict of interest. |
| Other sources of bias | Low | No other threats to internal validity were identified. |
