# Supplementary material for: Exposure to formaldehyde and asthma outcomes: A systematic review, meta-analysis, and economic assessment
Source: PLoS One. 2021 Mar 31;16(3):e0248258. doi: 10.1371/journal.pone.0248258 (PMC8011796; doi:10.1371/journal.pone.0248258)
Supplement: S92 Table — (DOCX) [file pone.0248258.s105.docx]

Supplemental Materials, Table 92. Characteristics of Zhao et al. 2008

| Bias domain | Authors’ judgment | Support for judgment |
| --- | --- | --- |
| Source population representation | Low | Ten junior high schools were arbitrarily selected in December 2004 within urban areas of Taiyuan city. In each of the 10 schools, five first-year classes were arbitrary selected, in different parts and floors in the school buildings. If there were fewer than five first-year classes, all were selected. About 90 % of invited students participated in the survey. |
| Blinding | Probably low | Blinding was not addressed. It is unlikely that parents, who completed the symptom questionnaire, would have been aware of classroom or living room formaldehyde levels. Exposure analyses were conducted at an independent laboratory, so exposure assessors would have been blind to the participants' outcome status. |
| Outcome assessment | Probably low | Students completed a questionnaire based on the International Study of Asthma and Allergy in Childhood (ISAAC) and the European Community Respiratory Health Survey (ECRHS) questionnaires. This is considered a valid and reliable questionnaire. The outcomes were not medically confirmed. |
| Confounding | Probably low | Models were adjusted for age, sex, parental asthma or allergy, ETS at home, recent home painting, new floor and new furniture in the preceding 12 months. Data were obtained using the study questionnaire, self-administered. No data on validity of the questionnaire was presented. |
| Incomplete outcome data | Low | There was one school with missing formaldehyde measurements due to technical failures. In one model, data for these individuals were excluded (10 % missing data based on 1836/1993 included data). In another model, the average value of available exposure measurements of other schools was applied. |
| Exposure assessment | Low | Indoor measurements in each of the selected classrooms and one outdoor measurement per school were collected by passive samplers for a continuous 7-day period. Concentrations were measured by accredited laboratories. Detection limit of 1 ug/m3 listed for formaldehyde. |
| Selective outcome reporting | Low | All of the study’s pre-specified (primary and secondary) outcomes outlined in the published manuscript’s methods, abstract, and/or introduction section that are of interest in the review have been reported in the pre-specified way. |
| Conflict of interest | Low | All authors are affiliated with academic/government institutions and declare no competing financial interests. |
| Other sources of bias | Low | The authors note that 4/1993 students were smokers themselves. Interesting that they did not exclude these from the analyses, but unlikely to have an impact on risk estimates. |
