# Supplementary material for: Exposure to formaldehyde and asthma outcomes: A systematic review, meta-analysis, and economic assessment
Source: PLoS One. 2021 Mar 31;16(3):e0248258. doi: 10.1371/journal.pone.0248258 (PMC8011796; doi:10.1371/journal.pone.0248258)
Supplement: S93 Table — (DOCX) [file pone.0248258.s106.docx]

Supplemental Materials, Table 93. Characteristics of Neamtiu et al. 2019

| Bias domain | Authors’ judgment | Support for judgment |
| --- | --- | --- |
| Source population representation | Probably low | Study population was a part of the SINPHONIE project, a multi-institution collaboration across 25 European countries conducted between 2010-2012. Participants for this study were from five public primary schools in Alba County in Romania. 15 total classrooms were included (3 classrooms per school). A total of 280 questionnaires were collected from 139 male and 141 female students, 15 questionnaires were collected from teachers. It was unclear how many questionnaires were sent out and what the participation rate was. Unclear whether there was any missing data from the questionnaires; the sample size for each reported result is not explicitly stated. There is no evidence to suggest either consistency or inconsistency of criteria applied across higher versus lower formaldehyde exposed groups. |
| Blinding | Probably low | No evidence of blinding, but formaldehyde measurements were taken at the classroom level and it is unlikely that the person measuring exposure would know the asthma outcomes for students located within that classroom. |
| Outcome assessment | Probably low | A standardized questionnaire was self-administered to students. Questionnaire has been validated in prior studies. Asthma-like symptoms included: difficulty breathing, dry cough, and wheezing in the past week. |
| Confounding | Probably low | The study adjusted for two Tier I confounders (age, tobacco exposure for the past week) and two Tier II confounders (gender, additional environmental exposure (NO2, CO, CO2, temperature, relative humidity, ventilation rate)). Although SES (Tier I confounder) was not adjusted for, children are attending the same school which may in part control for SES. |
| Incomplete outcome data | Probably low | There was insufficient information provided to assess missing data. Sample sizes were not provided for asthma-related outcomes and authors did not mention the existence of any missing outcome data. |
| Exposure assessment | Low | Formaldehyde exposure in classrooms were analyzed from air samples using high-performance liquid chromatography (HPLC) coupled with a UV-Vis detector. Air samples were collected on Radiello passive samplers exposed for five days inside the three classrooms and in one outside location, in each school. Samplers were installed according to ISO 16000-2 protocol. The method used for formaldehyde analysis was a validated method described and published by the Joint Research Center, the European Commissions' s science and knowledge service. All chemicals used for analysis were of high purity grade. Water needed for analysis was purified using a Milli-Q Ultrapure water purification system. Formaldehyde concentration determination in the air was based on the absolute amount of free formaldehyde detected, the exposure time of five days (108 h and 6480 min, respectively), and the sampling rate for free formaldehyde, of 99 mL/min. |
| Selective outcome reporting | Low | Results are reported for all outcomes specified in the abstract and methods. |
| Conflict of interest | Low | Authors include a conflict of interest statement reporting that there are no conflicts of interest. All authors are either affiliated with a university or a charter organization (Regional Environmental Center for Central and Eastern Europe) |
| Other sources of bias | Low | No additional potential risks of biases noted. |
