# Supplementary material for: Exposure to formaldehyde and asthma outcomes: A systematic review, meta-analysis, and economic assessment
Source: PLoS One. 2021 Mar 31;16(3):e0248258. doi: 10.1371/journal.pone.0248258 (PMC8011796; doi:10.1371/journal.pone.0248258)
Supplement: S94 Table — (DOCX) [file pone.0248258.s107.docx]

Supplemental Materials, Table 94. Characteristics of Yon et al. 2019

| Bias domain | Authors’ judgment | Support for judgment |
| --- | --- | --- |
| Source population representation | Probably low | Study population was a general population-based cohort study performed in 22 randomly selected classrooms of 11 elementary schools in Sonogram City, Korea (inner city Korean children). 620 fifth and sixth grade elementary school students (10-12 years old) were prospectively recruited. Ultimately, 427 students (68.9%) were enrolled who met the study criteria (completed the questionnaire, provided blood and urine samples, and underwent tests for oscillometric lung function, FENO, olfactory threshold, and acoustic rhinometry). All missing data were reported. No comparison was provided of children with missing data who were excluded compared to those who were included in the study. |
| Blinding | Probably low | No evidence of blinding, but formaldehyde measurements were taken at the classroom level and it is unlikely that the person measuring exposure would know the asthma outcomes for students located within that classroom |
| Outcome assessment | Probably low | Asthma outcomes was defined by the presence of characteristic symptoms and/or signs during the previous 12 months, based on the International Study of Asthma and Allergies in Childhood questionnaire, a standard survey. |
| Confounding | Probably low | The study adjusted for two Tier I confounders (age, environmental tobacco smoke exposure) and three Tier II confounders (sex, environmental exposures (keeping a pet at home), physician-diagnosed asthma in parents). Although SES (Tier I confounder) was not adjusted for, children are attending the same school which may in part control for SES. |
| Incomplete outcome data | Probably high | Some of missing data is reported in detail, but in results the reported association estimate for asthma outcomes is only represented for sample size of 10 and it is unclear why data is missing for other participants. |
| Exposure assessment | Probably high | Formaldehyde concentrations were measured twice in each classroom (once in the first half and once in the second half of the academic year). Formaldehyde was measured using formaldehyde monitors with pump (Z-300XP, Environmental Sensors co.) Annual average formaldehyde concentrations per assessment period were determined. Details regarding exposure assessment were brief and no mention of validated measurement techniques or QA/QC. |
| Selective outcome reporting | Low | Results are reported for all outcomes specified in the abstract and methods. |
| Conflict of interest | Low | Each author has completed a standard conflict of interest disclosure form, and only one author disclosed that they were supported by a government grant. |
| Other sources of bias | Low | No additional potential risks of biases noted |
