# Supplementary material for: Exposure to formaldehyde and asthma outcomes: A systematic review, meta-analysis, and economic assessment
Source: PLoS One. 2021 Mar 31;16(3):e0248258. doi: 10.1371/journal.pone.0248258 (PMC8011796; doi:10.1371/journal.pone.0248258)
Supplement: S95 Table — (DOCX) [file pone.0248258.s108.docx]

Supplemental Materials, Table 95. Characteristics of Fsadni et al. 2018

| Bias domain | Authors’ judgment | Support for judgment |
| --- | --- | --- |
| Source population representation | Probably high | Study population were from five primary schools consisting of nine to eleven year old students. Schools were selected randomly from five geographical clusters of schools. Three classrooms were randomly selected by ballot system from each school. Sample size is not reported. |
| Blinding | Probably low | No evidence of blinding, but formaldehyde measurements were taken at the classroom level and it is unlikely that the person measuring exposure would know the asthma outcomes for students located within that classroom |
| Outcome assessment | Probably low | Asthma-related outcomes were assessed using standardized ISAAC health questionnaire answered by student's parents. Lung function tests were performed on students by study investigators. |
| Confounding | High | Authors state that "logistic regression model was designed so as to eliminate potential confounding factors" but there is not mention of what confounding factors, if any, were adjusted for in the regression model for formaldehyde. |
| Incomplete outcome data | High | Quantitative association estimates are not reported; authors only report the direction of the effect (increased or decreased symptoms). It also appears that associations are considered increased/decreased if they were statistically significant. |
| Exposure assessment | Probably low | Radiello passive diffusive tubes (Fondazione Salvatore Maugeri) were used to measure formaldehyde. Sampling took place over a 5-day period (Monday morning until Friday afternoon). Pollutant concentrations were averaged. Details regarding exposure assessment were brief and only mention of QA/QC is in the Discussion section where authors state that "chemical pollutants were measured using standardized methodology and quality control." |
| Selective outcome reporting | Low | Results are reported for all outcomes specified in the abstract and methods. |
| Conflict of interest | Low | Authors include a conflict of interest statement reporting that there are no conflicts of interest. All authors are affiliated with a university |
| Other sources of bias | Low | No additional potential risks of biases noted |
