# Supplementary material for: Exposure to formaldehyde and asthma outcomes: A systematic review, meta-analysis, and economic assessment
Source: PLoS One. 2021 Mar 31;16(3):e0248258. doi: 10.1371/journal.pone.0248258 (PMC8011796; doi:10.1371/journal.pone.0248258)
Supplement: S96 Table — (DOCX) [file pone.0248258.s109.docx]

Supplemental Materials, Table 96. Characteristics of Idavain et al. 2019

| Bias domain | Authors’ judgment | Support for judgment |
| --- | --- | --- |
| Source population representation | Probably high | Study population consisted of participants in two different studies: SINPHONIE (Schools Indoor Pollution and Health: Observatory Network in Europe) and SOHOS (Study of the Health Impact of Oil-Shale Sector). Data collection for SINPHONIE took place in Tartu County during January-February 2012. SOHOS study was collected in Ida-Viru and Laane-Viru Counties between November 2014 and January 2015. In total, 1326 randomly selected schoolchildren from 25 schools, aged 8-12 years, living in North-Eastern Estonia (Ida-Viru and Laane-Viru counties) or South-Eastern Estonia (Tartu County) were included. Schools were selected randomly, according to SES and level of industrial pollution. A total of 1208 subjects completed the questionnaire and 1098 participated in the clinical examination. No discussion of sample size for results or further missing data from study participants, or a comparison of characteristics for students participating in study versus those not participating. |
| Blinding | Probably low | No evidence of blinding, but formaldehyde concentrations were modeled and geocoded to participant's home addresses and it is unlikely that the person measuring exposure would know the asthma outcomes for the student living at that address |
| Outcome assessment | Probably low | General population |
| Confounding | Probably high | The study adjusted for two Tier I confounders (age, parent's education and family income) and two Tier II confounders (sex, BMI). Study did not adjust for smoking status or exposure to secondhand smoke. |
| Incomplete outcome data | Probably low | There was insufficient information provided to assess missing data. Sample sizes were not provided for asthma-related outcomes and authors did not mention the existence of any missing outcome data. |
| Exposure assessment | Probably high | Annual mean concentrations in 2013 of formaldehyde were modelled in a 1x1km grid in Ida-Viru County, using an Eulerian air quality dispersion model that formed part of the Airvivo Air Quality Management System; detailed description is available in cited documentation. Modelled concentrations were linked with the geo-code of each respondent's home address in ArcGIS. No mention of validation or QA/QC of modeling approach. |
| Selective outcome reporting | Low | Results are reported for all outcomes specified in the abstract and methods. |
| Conflict of interest | Probably low | No conflict of interest statement, but all authors have university or government affiliations and the project was funded by grants from the Estonian Environmental Investment Centre (KIK) and the Estonian Ministry of Education and Research. |
| Other sources of bias | Low | No additional potential risks of biases noted |
