# Supplementary material for: Exposure to formaldehyde and asthma outcomes: A systematic review, meta-analysis, and economic assessment
Source: PLoS One. 2021 Mar 31;16(3):e0248258. doi: 10.1371/journal.pone.0248258 (PMC8011796; doi:10.1371/journal.pone.0248258)
Supplement: S97 Table — (DOCX) [file pone.0248258.s110.docx]

Supplemental Materials, Table 97. Characteristics of Willis et al. 2018

| Bias domain | Authors’ judgment | Support for judgment |
| --- | --- | --- |
| Source population representation | Probably high | Study population was obtained from individual patient hospitalizations for the entire state of Pennsylvania (67 total counties) from the Pennsylvania Health Care Cost Containment Council. Counties were excluded that were designated as urban by the PA Department of Health's Asthma Prevalence report due to large differences in urban versus rural air quality and other co-exposures. 29 counties were ultimately included, containing 571 zip codes. A total of 15,837 pediatric asthma-related hospitalizations were included. No discussion of sample size for results or further missing data from study participants. Missing data only addressed in one study characteristic (% with insurance). |
| Blinding | Probably low | No evidence of blinding, but formaldehyde concentrations were modeled to participant's home addresses and it is unlikely that the person measuring exposure would know the asthma outcomes for the child living at that address |
| Outcome assessment | Probably high | Asthma hospitalizations were obtained from the Pennsylvania Healthcare Cost Containment Council hospitalization data by identifying diagnostic codes with a 493 ICD-9 code, which indicates acute asthma exacerbation. While these codes indicate asthma exacerbation for the particular admittance, they are based on clinical observations of respiratory distress. The ICD-9 code data site indicates that "...not all people who have asthma have these symptoms. Having these symptoms doesn't always mean that you have asthma. Your doctor will diagnose asthma based on lung function tests, your medical history, and a physical exam." (http://www.icd9data.com/2015/Volume1/460-519/490-496/493/493.htm) However there is no other information regarding whether children had been diagnosed with asthma or follow-up with clinical examinations to confirm asthma status. Children under the age of 2 years old were excluded to avoid reflection of a viral illness, not a typical asthma case. |
| Confounding | Probably high | The study adjusted for one Tier I confounder (SES (county median household income quartile, county poverty under 18 years old) and three Tier II confounders (sex, race, additional environmental exposures (zip code respiratory hazard index)) as well as county log population density, year, quarter, insurance status, and county unemployment. Study did not adjust for age (although they did stratify results by age) or smoking status or exposure to environmental tobacco smoke. |
| Incomplete outcome data | Probably high | Formaldehyde exposure was evaluated using the Pennsylvania Unconventional Natural Gas Emission Inventory which has annualized emissions data from Unconventional Gas Drilling (UNGD) sites. Pollutants are reported in tons emitted per year. Data were linked from spud data on DrillingInfo to determine how much pollutant was reported per zip code on annual basis, leading to the assessment of annual sum of pollution in tons at the zip code level. Main model modeled exposure in a log-sum framework. No mention of validation or QA/QC of modeling approach. |
| Exposure assessment | Low | Results are reported for all outcomes specified in the abstract and methods. |
| Selective outcome reporting | Low | All of the study’s pre-specified (primary and secondary) outcomes outlined in the published manuscript’s methods, abstract, and/or introduction section that are of interest in the review have been reported in the pre-specified way. |
| Conflict of interest | Probably low | No conflict of interest statement, but all authors have university affiliations and the project was funded by grants from the NIH and authors state that the funders had no role in the design and conduct of study, collection, management, analysis, and interpretation of data, preparation, review, or approval of the manuscript, and decision to submit the manuscript for publication. |
| Other sources of bias | Low | No additional potential risks of biases noted |
