# Supplementary material for: Exposure to formaldehyde and asthma outcomes: A systematic review, meta-analysis, and economic assessment
Source: PLoS One. 2021 Mar 31;16(3):e0248258. doi: 10.1371/journal.pone.0248258 (PMC8011796; doi:10.1371/journal.pone.0248258)
Supplement: S98 Table — (DOCX) [file pone.0248258.s111.docx]

Supplemental Table 98. Egger’s Test for Meta-Analysis (testing for publication bias)

Number of studies = 8 Root MSE = .8959

------------------------------------------------------------------------------

Std_Eff | Coef. Std. Err. t P>|t| [95% Conf. Interval]

-------------+----------------------------------------------------------------

slope | .0561504 .0301545 1.86 0.112 -.0176351 .1299359

bias | .3948334 .3930585 1.00 0.354 -.566946 1.356613

------------------------------------------------------------------------------

Test of H0: no small-study effects P = 0.354
