# Supplementary material for: Exposure to formaldehyde and asthma outcomes: A systematic review, meta-analysis, and economic assessment
Source: PLoS One. 2021 Mar 31;16(3):e0248258. doi: 10.1371/journal.pone.0248258 (PMC8011796; doi:10.1371/journal.pone.0248258)
Supplement: S99 Table — (DOCX) [file pone.0248258.s112.docx]

Supplemental Table 99. Study categorization by population/outcome.

| **Study** | **Children** | **Adults** | **Asthma Diagnosis** | **Asthma Symptoms** | **Pulmonary Measures** | **Meta Analysis** |
| --- | --- | --- | --- | --- | --- | --- |
| Akbar Khanzadeh et al. 1994 (Cross-sectional) |  |  |  |  |  |  |
| Akbar Khanzadeh et al. 1997 (Cross-sectional) |  |  |  |  |  |  |
| Annesi Maesano et al. 2012 (Cross-sectional) |  |  |  |  |  |  |
| Billionnet et al. 2011 (Cross-sectional) |  |  |  |  |  |  |
| Burge et al. 1984 (Case reports) |  |  |  |  |  |  |
| Chatzidiakou et al. 2014 (Cross-sectional) |  |  |  |  |  |  |
| Choi et al. 2009 (Case-control) |  |  |  |  |  |  |
| Dannemiller et al. 2013 (Cross-sectional) |  |  |  |  |  |  |
| De Vos et al. 2009 |  |  |  |  |  |  |
| Delfino et al. 2003  (Cross-sectional) |  |  |  |  |  |  |
| Dumas et al. 2017  (Case control study nested within prospective cohort) |  |  |  |  |  |  |
| Elshaer et al. 2017 (Cross-sectional) |  |  |  |  |  |  |
| Ezratty et al. 2007 (Non-randomized controlled trial) |  |  |  |  |  |  |
| Fornander et al. 2014 (Cross-sectional) |  |  |  |  |  |  |
| Fransman et al. 2003 (Cross-sectional) |  |  |  |  |  |  |
| Frey et al. 2014 (Cross-sectional) |  |  |  |  |  |  |
| Frigas et al. 1984 (Case reports) |  |  |  |  |  |  |
| Frisk et al. 2002 (Cohort (Prospective)) |  |  |  |  |  |  |
| Frisk et al. 2006 (Case-control) |  |  |  |  |  |  |
| Frisk et al. 2009 (Cross-sectional) |  |  |  |  |  |  |
| Fsadni et al. 2018 (Cohort (Prospective)) |  |  |  |  |  |  |
| Gannon et al. 1995 (Case reports) |  |  |  |  |  |  |
| Garrett et al. 1999 (note Garrett et al. 1998 used same cohort so combined to one record) (Cross-sectional) |  |  |  |  |  |  |
| Gorski et al. 1991 (Cohort) |  |  |  |  |  |  |
| Green et al. 1987 (Randomized controlled trial) |  |  |  |  |  |  |
| Hanson et al. 1993 (Cross-sectional) |  |  |  |  |  |  |
| Harving et al. 1990 (Randomized controlled trial) |  |  |  |  |  |  |
| Hendrick et al. 1977 (Cohort (Prospective)) |  |  |  |  |  |  |
| Herbert et al. 1994 (Cross-sectional) |  |  |  |  |  |  |
| Horvath et al. 1988 (Cross-sectional) |  |  |  |  |  |  |
| Hsu et al. 2012 (Case-control) |  |  |  |  |  |  |
| Huang et al. 2016 (Nested case-control) |  |  |  |  |  |  |
| Hulin et al. 2010 (Nested case-control) |  |  |  |  |  |  |
| Hwang et al. 2011 (Case-control) |  |  |  |  |  |  |
| Idavain et al. 2019 (Cross-sectional) |  |  |  |  |  |  |
| Jacobsen et al. 2009 (Cohort (Prospective)) |  |  |  |  |  |  |
| Jeong et al. 2011 (Cross-sectional) |  |  |  |  |  |  |
| Kilburn et al. 1985 (Cohort (Prospective)) |  |  |  |  |  |  |
| Kilburn, Seidman, and Warshaw 1985 (Cross-sectional) |  |  |  |  |  |  |
| Kim et al. 2007 (Cross-sectional) |  |  |  |  |  |  |
| Kim et al. 2011 (Cross-sectional) |  |  |  |  |  |  |
| Kim et al. 2014 (Non-randomized controlled trial) |  |  |  |  |  |  |
| Kriebel et al. 1993 (Cross-sectional) |  |  |  |  |  |  |
| Kriebel et al. 2001 (Cohort (Prospective)) |  |  |  |  |  |  |
| Krzyzanowski et al. 1990 (Cross-sectional) |  |  |  |  |  |  |
| Lajoie et al. 2015 (Randomized controlled trial) |  |  |  |  |  |  |
| Liu et al. 1991 (Cross-sectional) |  |  |  |  |  |  |
| Lofstedt et al. 2009 (Cohort (Prospective)) |  |  |  |  |  |  |
| Lofstedt et al. 2011 (Cohort (Prospective)) |  |  |  |  |  |  |
| Low et al. 1985 (Cross-sectional) |  |  |  |  |  |  |
| Madureira et al. 2015 (note same cohort of children as Madureira et al. 2015b, but more comprehensive, so combined to one record) (Cross-sectional and case-control) |  |  |  |  |  |  |
| Madureira et al. 2016 (Cross-sectional and case-control) |  |  |  |  |  |  |
| Malaka et al. 1990 (Cross-sectional) |  |  |  |  |  |  |
| Mapou et al. 2013 (Cross-sectional) |  |  |  |  |  |  |
| Marks et al. 2010 (Non-randomized controlled trial) |  |  |  |  |  |  |
| Matsunaga et al. 2007 (Cross-sectional) |  |  |  |  |  |  |
| Mi et al. 2006 (Cross-sectional) |  |  |  |  |  |  |
| Milton et al. 1996 (Cross-sectional) |  |  |  |  |  |  |
| Neamtiu et al. 2019 (Cohort (Prospective)) |  |  |  |  |  |  |
| Norback et al. 1995 (Cross-sectional) |  |  |  |  |  |  |
| Norback et al. 2000 (Cross-sectional) |  |  |  |  |  |  |
| Nordman et al. 1985 (Case reports) |  |  |  |  |  |  |
| Popa et al. 1969 (Cohort (Prospective)) |  |  |  |  |  |  |
| Pourmabahabadian et al. 2006 (Cross-sectional) |  |  |  |  |  |  |
| Quackenboss et al. 1989 (Cross-sectional) |  |  |  |  |  |  |
| Raaschou-Nielsen et al. 2010 (Cohort (Prospective)) |  |  |  |  |  |  |
| Rumchev et al. 2002 (Case-control) |  |  |  |  |  |  |
| Sauder et al. 1987 (Non-randomized controlled trial) |  |  |  |  |  |  |
| Schenker et al. 1982 (Cross-sectional) |  |  |  |  |  |  |
| Sheppard et al. 1984 (Non-randomized controlled trial) |  |  |  |  |  |  |
| Smedje and Norback 2000 (Cohort (Prospective)) |  |  |  |  |  |  |
| Smedje and Norback 2001 (Cohort (Prospective)) |  |  |  |  |  |  |
| Smedje et al. 1997 (Cross-sectional) |  |  |  |  |  |  |
| Tavernier et al. 2006 (Cross-sectional) |  |  |  |  |  |  |
| Thetkathuek et al. 2016 (Cross-sectional) |  |  |  |  |  |  |
| Tuomainen et al. 2003 (Cohort) |  |  |  |  |  |  |
| Tuthill 1984 (Cross-sectional) |  |  |  |  |  |  |
| Uba et al. 1989 (Cohort (Prospective)) |  |  |  |  |  |  |
| Venn et al. 2003 (Case-control) |  |  |  |  |  |  |
| Veremchuk et al. 2016 (Cross-sectional) |  |  |  |  |  |  |
| Wieslander et al. 1997 (Cross-sectional) |  |  |  |  |  |  |
| Willis et al. 2018 (Repeated cross-sectional) |  |  |  |  |  |  |
| Witek, Jr et al. 1986 (Randomized controlled trial) |  |  |  |  |  |  |
| Witek, Jr et al. 1987 (Randomized controlled trial) |  |  |  |  |  |  |
| Yeatts et al. 2012 (Cross-sectional) |  |  |  |  |  |  |
| Yon et al. 2019 (Cohort (Prospective)) |  |  |  |  |  |  |
| Yoon and Lin 2014 (Case-control) |  |  |  |  |  |  |
| Zammit-Tabona et al. 1983 (Cross-sectional) |  |  |  |  |  |  |
| Zhai et al. 2013 (Cross-sectional) |  |  |  |  |  |  |
| Zhao et al. 2008 (Cross-sectional) |  |  |  |  |  |  |
