# Supplementary material for: Exposure to formaldehyde and asthma outcomes: A systematic review, meta-analysis, and economic assessment
Source: PLoS One. 2021 Mar 31;16(3):e0248258. doi: 10.1371/journal.pone.0248258 (PMC8011796; doi:10.1371/journal.pone.0248258)
Supplement: S100 Table — (DOCX) [file pone.0248258.s113.docx]

Table 100. Study characteristics by population/outcome

**Children/Asthma Diagnosis**

| **Study (Study Design)** | **Study population & location** | **Sample size** | **Exposure assessment** | **Exposure ranges** | **Outcome assessment (not including pulmonary function tests)** | **Outcomes Reported** | **Confounders** | **Results** |
| --- | --- | --- | --- | --- | --- | --- | --- | --- |
| **Studies considered for meta-analysis** | | | | | | | | |
| Hulin et al. 2010 (Nested case-control) | Children in the general population attending school and living within a city (Clermont-Ferrand, Auvergne, France) who participated in the Six Cities study or in surrounding rural areas (Auvergne, France) | 63 urban children (32 asthmatics and 31 controls) and 51 rural children (24 asthmatics and 27 controls) (mean age 12.6 years) | Formaldehyde measured continuously for one week in the living room; assessed during summer and winter in urban area in 2003-2004 and in summer in rural area 2006-2007 | Median: 19.2 ug/m3; maximum: 75.1 ug/m3 | Questionnaire completed by parents; cases identified on basis of "yes" response to questions about ever having asthma, wheezing in last year, and use of asthma medication | Current asthma, ever asthma, asthma | Age, allergic rhinitis, exposure to passive smoking during early childhood, family history of allergy, location, season, sex | OR=1.07, 95% CI [1.01, 1.13] for asthma cases per 10 ug/m3 increase in formaldehyde exposure in urban environments. OR=1.9, 95% CI [1.08, 3.5] for asthma cases per 10 ug/m3 increase in formaldehyde exposure in rural environments. OR=0.62, 95% CI [0.18, 2.14] for ever asthma comparing high (>19.2 ug/m3) asthma versus low (<19.2 ug/m3) in urban environments. OR=10.72, 95% CI [1.69, 67.61] for ever asthma comparing high (>19.2 ug/m3) asthma versus low (<19.2 ug/m3) in rural environments. OR=0.24, 95% CI [0.03, 2.29] for current asthma comparing high (>19.2 ug/m3) asthma versus low (<19.2 ug/m3) in urban environments. OR=9, 95% CI [1, 82] for current asthma comparing high (>19.2 ug/m3) asthma versus low (<19.2 ug/m3) in rural environments |
| Idavain et al. 2019 (Cross-sectional) | Children in the general population from 25 schools in Ida-Viru, Lääne-Viru and Tartu Counties in Estonia | 1326 children from randomly selected schools. Age of students range 8-12 years, | Annual mean concentrations of formaldehyde in 2013 were modelled | Range: 2.59-4.87 ug/m3 | Asthma-related outcomes were assessed through questionnaires that were distributed to students by teachers and completed by parent and child together. Questions inquired whether child ever had wheezing or whistling in the chest at any time in the past, whether they had asthma diagnosed by physician, whether child ever had attacks of asthma, and whether child had wheezing or whistling in the chest without cold in the past 12 months. Children were then invited for a clinical examination for further evaluation. | Asthma diagnosis, asthma symptoms (wheezing, whistling in chest, asthma attack) | Age, sex, BMI, parent's education and family income. | OR = 1.01, 95% CI: [0.90, 1.13] for every wheezing per 1 ug/m3 formaldehyde exposure. |
| Kim et al. 2007 (Cross-sectional) | Children in the general population attending all eight primary schools in Knivsta Municipality in rural outskirts of Uppsala City, Sweden | 1014 school children (ages 5-15 years, mean age 9 years) | Formaldehyde measured in three classrooms in each school for 6 hours in May-June 2000 | Mean for 23 classrooms: 7.13 ug/m3 (range 3-16) | Questionnaire completed by parents with cooperation of child in April-May 2000 and included questions about asthma (doctor-diagnosed, asthma medications, asthma attacks), airway symptoms during last year without using phrase "asthma" (wheezing, breathlessness) | Current asthma (diagnosed), current asthma medication, wheezing, nocturnal breathlessness, and daytime breathlessness | Age, gender | OR=1.03, 95% CI [0.86, 1.24] for nocturnal breathlessness per 1 ug/m3 increase in formaldehyde exposure in classroom. OR=0.96, 95% CI [0.87, 1.05] for wheeze per 1 ug/m3 increase in formaldehyde exposure in classroom. OR=0.96, 95% CI [0.85, 1.08] for daytime breathlessness per 1 ug/m3 increase in formaldehyde exposure in classroom. OR=1.02, 95% CI [0.93, 1.13] for doctor-diagnosed asthma per 1 ug/m3 increase in formaldehyde exposure in classroom. |
| Kim et al. 2011 (Cross-sectional) | Children in the general population attending 4th grade in twelve randomly-selected schools in three cities (Guri, Namyangju, and Chunchon) in Korea | 1915 total school children (mean age 10 years) | Formaldehyde measured continuously for 7 days in classrooms (n=34) and outside classroom windows (n=12) in November-December 2004 | Mean (classroom): 18.2 ug/m3 (SD 17.3; range 2.7-52.8); mean (outdoor): 16.5 ug/m3 (SD 12.5; range 3.3-45.3) | Questionnaire completed by subjects; current asthma defined as either having current medication or asthma attack in last 12 months | Doctor-diagnosed asthma, current asthma, wheeze | Age, sex, self-reported furry pet or pollen allergy, and home environment (remodeling, changing floor, age of home building, environmental tobacco smoke and indoor dampness) | OR=1.2, 95% CI [0.44, 3.24] self-reported wheezing during last 12 months per 10 ug/m3 increase in outdoor formaldehyde exposure. OR=1.15, 95% CI [0.88, 1.5] self-reported wheezing during last 12 months per 10 ug/m3 increase in indoor classroom formaldehyde exposure. OR=2.1, 95% CI [0.71, 6.23] current asthma (either having current asthma medication or having an asthma attack during the last 12 months) per 10 ug/m3 increase in outdoor formaldehyde exposure. OR=1.04, 95% CI [0.78, 1.4] current asthma per 10 ug/m3 increase in indoor classroom formaldehyde exposure. OR=0.8, 95% CI [0.22, 2.85] asthma diagnosis per 10 ug/m3 increase in outdoor formaldehyde exposure. OR=0.92, 95% CI [0.67, 1.26] asthma diagnosis per 10 ug/m3 increase in indoor classroom formaldehyde exposure. |
| Krzyzanowski et al. 1990 (Cross-sectional) | Municipal employee households in the general population with adults and children 5-15 years of age in Pima County Arizona | 298 children (ages 6-15 years) and 613 adults | Formaldehyde measured in the kitchen, main living area and each subject's bedroom for two 1-week periods | Mean from 202 households: 26 ppb; maximum: 140 ppb | Questionnaire completed by subjects and included questions about asthma (doctor-diagnosed with assessment of current status) and chronic respiratory symptoms (cough/phlegm, wheezing, and shortness of breath with wheezing) | Asthma diagnosis, pulmonary function test | Current smoker, education, environmental tobacco smoke, race/ethnicity | Prevalence rates per 100 subjects of current diagnosed asthma for children: 11.7 (<=40 ppb formaldehyde), 4.2 (41-60ppb formaldehyde), 23.8 (>60ppb formaldehyde). Relation of PEFR (Liters/Minute) to indoor formaldehyde (ppb): -1.28 (mean) +/- 0.46 (SE) for children <15 years; 0.09 (mean) +/-0.27 (SE) for adults >15 years |
| Mi et al. 2006 (Cross-sectional) | Children in the general population attending junior high schools in central Shanghai and western Shanghai near Huang Pu river in China | 1414 total school children from 5 schools in each district participated by questionnaire in November 2000 (aged 12-14 years) | Formaldehyde measured in 30 classrooms for 4 hours using stationary monitor in November-December 2000 | Mean: 9.4 ug/m3 (SD 6.9; range 3-20) | Questionnaire completed by subjects and included questions about asthma (doctor-diagnosed, asthma medications, asthma attacks), airway symptoms during last year without using phrase "asthma" (wheezing, breathlessness) | Current asthma (diagnosed), asthma attack, medication use, current wheeze, nocturnal breathlessness, daytime breathlessness | Age, gender, indoor molds, smoking, water leakage | OR=1.01, 95% CI [0.56, 1.81] for current wheeze per 10 ug/m3 increase in formaldehyde exposure. OR=1.09, 95% CI [0.86, 1.38] for daytime breathlessness per 10 ug/m3 increase in formaldehyde exposure. OR=1.26, 95% CI [0.63, 2.53] for nocturnal breathlessness per 10 ug/m3 increase in formaldehyde exposure. OR=1.24, 95% CI [0.63, 2.45] for asthma attack per 10 ug/m3 increase in formaldehyde exposure. OR=1.26, 95% CI [0.65, 2.46] for asthma medication per 10 ug/m3 increase in formaldehyde exposure. OR=1.3, 95% CI [0.72, 2.32] for current asthma per 10 ug/m3 increase in formaldehyde exposure. |
| Rumchev et al. 2002 (Case-control) | Children in the general population with asthma identified by the Accident and Emergency Dept at the Princess Margaret Hospital for Children and nonasthmatic controls identified through the Health Dept of Western Australia, Perth, Western Australia | 88 asthmatic children (mean age 25 months) and 104 nonasthmatic controls (mean age 20 months) (ages 6 months-3 years) | Formaldehyde measured in the living room and child's bedroom for 8 hours during the day in July-September 1998 and December 1998-March 1999 | Mean bedroom: 30.2 ug/m3; mean living room: 27.5 ug/m3 | Asthma cases were children discharged with medical diagnosis of asthma from emergency department; questionnaire from American Thoracic Society completed by parents for respiratory symptoms (including wheeze) and home characteristics | Asthma diagnosis, wheeze | Age, air conditioning, allergen levels of house dust mite, atopy, child allergies, family history of asthma, humidifier and gas appliances, indoor air pollutants, indoor temperature, presence of pets, relative humidity, sex, smoking inside, socioeconomic status | OR=1.003, 95% CI [1.002-1.004] for asthma diagnosis per 10-unit (ug/m3) increase in formaldehyde exposure. Children who reported wheeze were also exposure to high average indoor levels of formaldehyde (40.5 ug/m3) compared to those without such symptoms (26.7 ug/m3) and the difference was significant (p<0.01) |
| Smedje and Norback 2001 (Prospective Cohort) | Children in the general population attending 39 public schools in Uppsala county in Sweden (follow up study to Smedje and Norback 2000) | 1347 students in 1st, 4th or 7th grade (mean age 10.3 years in 1993 and 14.3 years in 1997) | Formaldehyde was measured for 4 hours in 2-5 secondary school classrooms and for each primary school classroom for each school in 1993 (prior to installation of new ventilation system) and in 1995 (after installation of new ventilation system) | Arithmetic mean: 8 ug/m3 (range <5-72); geometric mean: 4 ug/m3 (SD 2.3) | Questionnaire completed by subjects in 1993 and 1997 included question on whether student had ever had asthma and if diagnosis made by physician; additional questions on lower respiratory symptoms based on questionnaire from European Community Respiratory Health Survey (ECRHS) | Asthma diagnosis (incidence) | Age, atopy, smoking, sex | OR=1.2, 95% CI [0.8-1.7] for asthma diagnosis per 10 ug increase in formaldehyde in classroom air |
| Smedje et al. 1997 (Cross-sectional) | Children in the general population attending 11 public schools in Uppsala county in Sweden | 627 students in 7th grade (ages 13-14 years) | Formaldehyde was measured for 4 hours in 2-3 classrooms for each school (total 28 classrooms) in 1993 | Arithmetic mean: <5 ug/m3 (range <5-10) | Questionnaire on asthmatic symptoms amended from one used in the ECRHS completed by subjects; current asthma defined as physician diagnosis and symptoms within the last year | Current asthma (diagnosed) | Controlled for "personal factors," but no explicit discussion of what these included. May include atopy, food allergy, and whether attended day care center for several years | OR=1.1, 95% CI [1.01-1.2] for current asthma per 1-unit change (mg/m3) in formaldehyde concentration |
| Zhao et al. 2008 (Cross-sectional) | Children in the general population attending first year classes in 10 junior high schools within urban areas of Taiyun, China | 1993 school children (mean age 12.8 years) | Formaldehyde measured continuously for 7 days in one representative location in each school | Mean (classroom): 2.3 ug/m3 (SD 1.1; range 1.0-5.0); mean (outdoor): 5.8 ug/m3 (SD 0.6; range 5.0-7.0) | Questionnaire completed by subjects including questions on asthma (cumulative, doctor-diagnosed, and current) and on respiratory health (wheeze, breathlessness) based on International Study of Asthma and Allergy in Childhood (ISAAC) | Cumulative asthma (diagnosed), wheeze or whistling in the chest, nocturnal and daytime attacks of breathlessness | Personal and home environmental factors, age, environmental tobacco smoke at home, indoor and outdoor pollutants, new floor and new furniture in preceding 12 months, parental asthma or allergy, recent home painting, sex | OR=1.11, 95% CI [0.55, 2.23] for cumulative asthma per 1 ug/m3 increase in formaldehyde exposure indoor. OR=4.61, 95% CI [1.09, 19.5] for cumulative asthma per 1 ug/m3 increase in formaldehyde exposure outdoor. OR=0.93, 95% CI [0.78, 1.1] for daytime breathlessness per 1 ug/m3 increase in formaldehyde exposure indoor. OR=1.29, 95% CI [0.99, 1.68] for cumulative asthma per 1 ug/m3 increase in formaldehyde exposure outdoor. OR=1.11, 95% CI [0.87, 1.41] for wheeze or whistling in the chest per 1 ug/m3 increase in formaldehyde exposure indoor. OR=1.32, 95% CI [0.86, 2.04] for wheeze or whistling in the chest per 1 ug/m3 increase in formaldehyde exposure outdoor. OR=1.92, 95% CI [0.87, 1.41] for nocturnal attacks of breathlessness per 1 ug/m3 increase in formaldehyde exposure indoor. OR=2.03, 95% CI [0.91, 4.54] for nocturnal attacks of breathlessness per 1 ug/m3 increase in formaldehyde exposure outdoor. |
| **Studies not considered for meta-analysis** | | | | | | | | |
| Annesi Maesano et al. 2012 (Cross-sectional) | Children in the general population attending schools recruited into the Six Cities study in France | 6590 children (mean age 10.4 years) | Formaldehyde measured in schools | Median: 26.8 ug/m3; 25th percentile: 16.8 ug/m3; 75th percentile: 33.2 ug/m3 | Medical examination and ISAAC questionnaire completed by parents | Asthma (allergic and non-allergic) over past year, exercise-induced asthma | Paternal or maternal history of asthma and allergic diseases, passive smoking, gender, age | OR=1.1, 95% CI [0.87, 1.38] for asthma in the past year for 2nd tertile of formaldehyde exposure (19.1-28.4 ug/m3) compared to 1st tertile (<=19.1 ug/m3). OR=0.9, 95% CI [0.76, 1.08] for 3rd tertile of formaldehyde exposure (>28.4 ug/m3) compared to 1st, p-value for trend = 0.4428. OR=0.73, 95% CI [0.51, 1.03] for nonallergic asthma in the past year for 2nd tertile of formaldehyde exposure compared to 1st tertile. OR=0.82, 95% CI [0.68, 0.99] for 3rd tertile of formaldehyde exposure compared to 1st, p-value for trend = 0.32498. OR=1.31, 95% CI [1.01, 1.71] for allergic asthma in the past year for 2nd tertile of formaldehyde exposure compared to 1st tertile. OR=0.96, 95% CI [0.69,1.35] for 3rd tertile of formaldehyde exposure compared to 1st, p-value for trend = 0.9542. Correlation between formaldehyde exposure and exercise-induced asthma = -0.018, p-value = 0.2257. |
| Chatzidiakou et al. 2014 (Cross-sectional) | Children in the general population attending two primary state schools in the greater London area | 151 children (mean age 10 years) | Formaldehyde measured in three classrooms and one outdoor site for 5 consecutive days during the heating | Average (suburban school): 32.70 ug/m3 (SD 4.0); average (urban school): 12.81 ug/m3 (SD 3.7) | Standardized questionnaire completed by subjects | Asthma | Personal (gender, age, exposure to tobacco smoke, satisfaction with the school environment, and stress levels) and psychosocial factors. There is no information on which psychosocial factors were considered. | Urban schools had almost eight times higher asthma prevalence and asthmatic symptoms (12.6%) compared with suburban schools (1.6%), p < 0.001 |
| Choi et al. 2009 (Case-control) | Child patients in the general population with atopy recruited from outpatient clinic in Seoul, South Korea | 36 children with allergic asthma (mean age 16.2 years) and 28 non-atopic controls (mean age 15.4) | Formaldehyde measured inside and outside subjects' homes over period from March to June 2006 | Geometric mean (indoor): 42.46 ug/m3 controls, 54.15 ug/m3 allergic asthma cases; geometric mean (outdoor): 5.07 ug/m3 controls, 9.35 ug/m3 allergic asthma cases | Medical records for diagnosis of atopy, with skin prick tests, and IgE assays | Asthma | No confounders or adjustment factors reported | Geometric mean for formaldehyde exposure = 9.35 for allergic asthma cases, 5.07 for non-atopic controls, p-value non-significant |
| Garrett et al. 1999 (note Garrett et al. 1998 used same cohort so combined to one record) (Cross-sectional) | Children in households in the general population recruited for study in Latrobe Valley, Victoria, Australia | 148 children (ages 7-14 years, mean age 10.2 years) from 80 households | Formaldehyde measured for four days in bedrooms of children, living rooms, kitchens, and outside the home between March-April, May, and September 1994 and January-February 1995 | Median: 15.8 ug/m3; maximum: 139 ug/m3 | Questionnaire completed by parents included questions on respiratory symptoms in previous year for cough, shortness of breath, wheeze, asthma attacks and chest tightness | Asthma, respiratory symptom score | Parental allergy and parental asthma | Mean respiratory score=1.09, 95% CI [0.42, 1.76] for formaldehyde exposure <20 ug/m3, 2.21, 95% CI [1.7, 2.75] for formaldehyde exposure 20-50 ug/m3, 2.59, 95% CI [1.67, 3.48] for formaldehyde exposure >50 ug/m3. Proportion asthmatic=16% for formaldehyde exposure <20 ug/m3, 39% for formaldehyde exposure 20-50 ug/m3, 44% for formaldehyde exposure >50 ug/m3. Bedroom formaldehyde-exposure groups showed no significant differences between groups, but there were significant differences between highest recorded formaldehyde level groups (chi-square=6.84, df=2, p=0.03). A higher proportion of asthmatics was seen with higher formaldehyde exposure, with a significant linear trend present (p=0.02). Adjusted odds ratio for asthma was not significantly different from 1.0 (exact OR not provided). |
| Hsu et al. 2012 (Case-control) | Children in the general population attending randomly selected kindergartens and day care centers (n=335 participating) in the greater Tainan Metropolitan area of Taiwan, China | 9 asthmatic children and 42 non-asthmatic controls (ages 3-9 years, mean age 7 years) | Formaldehyde measured in children's bedroom for 2 hours between August 2008 and September 2009 | Median (all children): 6.2 ppb (range 25th-75th percentile 4.3-20.4); median (asthma cases): 4.3 ppb (range 25th-75th percentile 3.2-9.6); median (controls): 13.8 ppb (range 25th-75th percentile 4.3-24.6) | Medical examination by pediatrician to diagnose asthma including physical examination and standardized questionnaire | Asthma diagnosed by medical examination | The analyses with formaldehyde as the exposure of concern do not account for potential confounders. Differences were identified between study subjects and the original population, including child's gender, parental education level, parental allergic history, and parental smoking status. | Median measured 2-hour indoor formaldehyde levels in the bedrooms of physician confirmed asthma case children (4.3 ppb; 25th percentile: 3.2, 75th percentile: 9.6) were statistically significantly lower (p=0.03) than median measured formaldehyde levels in the bedrooms of non-symptomatic control children(13.8 ppb; 25th percentile: 4.3, 75th percentile: 24.6). |
| Huang et al. 2016 (Nested case-control) | Children in the general population attending 88 randomly selected kindergartens from Shanghai's six districts | 1216 children with asthma and 8651 nonsymptomatic children (ages 5-10 years) | Formaldehyde measured as in children's bedroom over seven consecutive days | Mean formaldehyde 24 hour measurements over cases and controls: 21.5 ug/m3 (sd=13.0). 407 (99.3%) samples were <100 ug/m3. Children's ages ranged from 5-10 years. | Questionnaire completed by parents derived from the International Study of Asthma and Allergies in Childhood. Asked questions of whether child has been diagnosed with asthma by a doctor (yes versus no) | Asthma diagnosed by medical examination | Age, sex, located district of residence, family history of atopy, ownership of the current residence, household environmental tobacco smoke, household dampness-related exposures, inspection season | OR=1.21, 95% CI [0.60, 2.45] for childhood asthma comparing 2nd quartile of formaldehyde exposure in child's bedroom to 1st quartile; OR=0.89, 95% CI [0.64, 1.24] for 3rd quartile, OR=1.09, 95% CI [0.86, 1.38] for fourth quartile |
| Hwang et al. 2011 (Case-control) | Children in the general population attending elementary school in Seongbuk, Seoul | 33 asthmatic children and 40 non-asthmatic controls (ages 8-13 years) | Formaldehyde measured using personal, indoor, and outdoor monitors for 3 days in 2008 | Geometric mean (indoor): 33.3 ug/m3; geometric mean (outdoor): 5.0 ug/m3; geometric mean (personal): 27.8 ug/m3 | Parents completed ISAAC questionnaire; children with self-reported asthma symptoms or physician-diagnosed included as cases | Asthma diagnosis | Age, gender, family income, parents' academic background, passive smoking | OR=1.0, 95% CI [1.0, 1.1] for childhood asthma for increasing formaldehyde exposure. Unit of increasing exposure was unclear. |
| Jeong et al. 2011 (Cross-sectional) | Children in the general population attending second grade in 56 elementary schools (11 in Incheon, Korea and 45 in Jeju, Korea) | 1226 children attending Incheon schools (mean age 9.2 years) and 1748 children attending Jeju schools (mean age 9 years) | Formaldehyde measured in 11 Incheon schools and 2 Jeju schools as a single measurement in classrooms, cafeterias, infirmaries, playgrounds, and rooftops in December 2008 | Mean outdoor exposure: 28.64 ug/m3 (SD 65.26) for Incheon and 10.00 ug/m3 (SD 0) for Jeju; mean indoor exposure: 279.44 ug/m3 (SD 23.83) for Incheon and 196.67 ug/m3 (SD 87.31) for Jeju | ISAAC questionnaire completed by parents | Ever asthma, asthma treatment over last 12 months, ever wheezing or whistling, wheezing over last 12 months | Study groups were not statistically different in sex, height, and weight. Study schools where in industrial and non-industrial areas (proxies for SES), parental history of asthma, and age were also reported. Analyses were unadjusted t-tests and chi-squared tests. | N=159 (13.13%) with ever diagnosis of asthma for children living in Incheon (high levels of formaldehyde exposure) compared to N=230 (13.38%) children living in Jeju, p-value=0.47. N=50 (4.19%) with asthma treatment in last 12 months for children living in Incheon compared to N=64 (3.78%) children living in Jeju, p-value=0.57. N=304 (24.96%) with ever wheeze or whistling for children living in Incheon compared to N=321 (18.80%) children living in Jeju, p-value<0.01. N=116 (9.50%) with wheezing in last 12 months for children living in Incheon compared to N=115 (6.83%) children living in Jeju, p-value<0.01. |
| Madureira et al. 2015 (note same cohort of children as Madureira et al. 2015b, but more comprehensive, so combined to one record) (Cross-sectional and case-control) | Children in the general population attending 20 public primary schools and a subset of asthmatic children in Porto, Portugal. Investigation was conducted between 2011-2013. | 1099 school children for classroom exposure measurements; 38 asthmatic children and 30 nonasthmatic children for home exposure measurements (ages 8-9 years) | School measurements: formaldehyde measured over 5 day period at breathing zone in 73 classrooms; Nov. 2011-Dec. 2012 and Nov. 2012-March 2013; Home measurements: formaldehyde measured over period of 7 days in rear of child's bedroom in Nov. 2011-Dec. 2012 and Nov. 2012-March 2013 | Median (school): 17.5 ug/m3; 25th percentile (school): 13.8 ug/m3; 75th percentile (school): 23.1 ug/m3; median (home): 11.4 ug/m3 for cases, 14.8 ug/m3 for controls | Parents completed paper-based questionnaire used in the International Study of Asthma and Allergies in Childhood; asthmatic cases answered yes to at least one question on asthma (doctor-diagnosed; wheezing in last year) | For school measurements: Asthma in school, doctor-diagnosed asthma, wheeze <30 d, wheeze <12 mo, ever wheeze, pulmonary function tests; for home measurements: asthma | No confounders or adjustment factors reported | No statistical comparison between formaldehyde levels between cases (asthmatic children) and controls |
| Smedje and Norback 2000 (Prospective Cohort) | Children in the general population attending 39 public schools in Uppsala county in Sweden | 1476 students in 1st, 4th or 7th grade (mean age 10.4 years in 1993 and 12.3 years in 1995) | Formaldehyde was measured for 4 hours in 2-5 secondary school classrooms and for each primary school classroom for each school in 1993 (prior to installation of new ventilation system) and in 1995 (after installation of new ventilation system) | Geometric mean 1993 (received new ventilation system later): 6 ug/m3; geometric mean 1993 (no new ventilation system): 3 ug/m3; change from 1993-1995 (new ventilation system): -4 ug/m3; change from 1993-1995 (no new ventilation system): 4 ug/m3 | Questionnaire completed by subjects in 1993 & 1995 included questions about asthma and asthma symptoms (amended from those used by ECRHS); current asthma defined as ever had asthma diagnosed by doctor and had at least one asthmatic symptom recently or using medication; asthmatic symptoms include recurrent persistent cough, persistent wheeze or shortness of breath, or during past 12 months had asthma attack, shortness of breath after exercise or nocturnal shortness of breath | Current asthma, ever asthma (doctor-diagnosed), any asthmatic symptoms, more than 1 asthmatic symptom | Age, atopy, smoking, sex | OR=0.3, 95% CI [0.1, 0.8] for any asthmatic symptoms comparing children with new ventilation to those without. OR=0.6, 95% CI [0.2, 2.8] for ever doctor's asthma diagnosis comparing children with new ventilation to those without. OR=1.2, 95% CI [0.4, 4.1] for current asthma comparing children with new ventilation to those without. OR=0.5, 95% CI [0.2, 0.97] for more asthmatic symptoms in 1995 than in 1993 comparing children with new ventilation to those without. |
| Tavernier et al. 2006 (Cross-sectional) | Children in the general population participating in the Indoor Pollutants, Endotoxin, Allergens, Damp and Asthma in Manchester (IPEADAM) study recruited as patients of 2 primary care facilities or healthy controls subjects in South Manchester, United Kingdom | 200 children (ages 4 to 17 years) | Formaldehyde measured during 2 visits per home 1 week apart | Not reported | Questionnaire validated against physician diagnosis of asthma | Asthma diagnosed by medical examination | Considered factors: bedroom sharing, benzene in bedroom, dust mite allergen, endotoxin, furred pet ownership, gas cooking, nitrogen dioxide in bedroom, nitrogen dioxide in living room, number of children in household, presence of smokers, redecoration in living room, respirable suspended particles in living room, self-reported absence of dampness in home, self-reported dampness in kitchen and bathroom, single-parent family, solanesol particulate matter in bedroom, solanesol particulate matter in living room, time in residence. Unclear what author ultimately controlled for. | OR=0.82, 95% CI [0.33, 2.05] for asthma comparing second tertile of exposure to formaldehyde to first tertile. OR=1.22, 95% CI [0.49, 3.07] for asthma comparing third tertile of exposure to formaldehyde to first tertile. |
| Veremchuk et al. 2016 (Cross-sectional) | Adult and children residents in the general population with asthma in Vladivostok, Russia | Asthma morbidity in Vladivostok (sample size not reported) | Formaldehyde measured using air quality monitors from six stationary observation posts during 2008-2012 | Not reported | Medical records | Asthma diagnosis | The study evaluated effects by age groups (children, adolescents, and adults). Climatic factors and indicators of anthropogenic air pollution in the city were also evaluated. | No significant correlation reported between formaldehyde exposure and asthma outcome |
| Yeatts et al. 2012 (Cross-sectional) | Children and adults in the general population recruited based on two-stage cluster sample design in the United Arab Emirates (UAE) | 1590 individuals within four age/sex categories: adult male (ages 19-50 years), adult female (ages 19-50 years), adolescent (ages 11-18 years), and child (ages 6-10 years) | Formaldehyde measured in the common living room for a 7 day period in 2009-2010 | Median: <7.37 ug/m3 (limit of quantification: 7.37; range: 7.37-168.2) | Subjects were interviewed and asked about ever having doctor-diagnosed asthma; respiratory symptoms assessed using ISAAC and Behavioral Risk Factor Surveillance System questions | Ever asthma, wheezing limited speech to 1 or 2 words between breaths, wheezing in last 4 months, wheezing in last 12 months, ever having wheezing and whistling in chest, shortness of breath one or more times a month, shortness of breath in last 12 months, chest tightness/difficulty breathing in last 12 months, chest tightness/difficulty breathing one or more times a month | Urban/rural area, household tobacco smoke exposure, gender, age group | OR=1.43, 95% CI [0.83, 2.46] for chest tightness/difficulty in breathing in last 12 months comparing high formaldehyde exposure group (7.37-168.2ppm) to low formaldehyde exposure group (<7.37ppm). OR=1.55, 95% CI [0.97, 2.48] for shortness of breath in last 12 months comparing high to low formaldehyde exposure group. OR=1.32, 95% CI [0.73, 2.37] for ever asthma comparing high to low formaldehyde exposure group. OR=1.31, 95% CI [0.71, 2.42] for ever having wheezing or whistling in chest comparing high to low formaldehyde exposure group. OR=0.64, 95% CI [0.21, 1.98] for wheezing in past 12 months comparing high to low formaldehyde exposure group. OR=3.48, 95% CI [0.81, 14.89] for wheezing in past 4 weeks comparing high to low formaldehyde exposure group. OR=4.18, 95% CI [1.23, 14.22] for wheezing limiting speech to 1 or 2 words between breaths comparing high to low formaldehyde exposure group. OR=3.68, 95% CI [1.11, 12.27] for shortness of breath one or more times a month comparing high to low formaldehyde exposure group. OR=6.52, 95% CI [1.91, 22.31] for chest tightness/difficulty in breathing one or more times a month comparing high to low formaldehyde exposure group. |
| Yoon and Lin 2014 (Case-control) | Children in the general population attending elementary school in Andong, Korea | 162 students (mean age 11.5 years) | Formaldehyde measured using personal samplers in breathing zone for 3 working days | Geometric mean (asthma cases): 6.96 ug/m3; geometric mean (controls): 8.31 ug/m3 | Questionnaire completed by subjects and included questions from American Thoracic Society criteria; students asked if they had asthma symptoms, and if yes, what symptoms were and if they had been diagnosed with asthma by a physician | Asthma diagnosis | Age, gender, family history of asthma, family income, amount of house sunlight, distance from bus within 100m, household with smokers, outdoor chemical odors | There was no significant difference between the asthmatic group and non-asthmatic group formaldehyde exposure level; the asthmatic group had a formaldehyde exposure of 6.96 ug/m3 GM, 2.26 GSD; the non-asthmatic group had an exposure of 8.31 ug/m3 GM, 1.66 GSD |
| Zhai et al. 2013 (Cross-sectional) | Households of children and adults in the general population decorated within the previous four years in main urban area of Shenyang, China | One adult per household and 82 children in 186 residential houses | Formaldehyde measured in bedrooms, living rooms, and kitchens over 1 month to 3 years | Polluted homes: 0.093 mg/m3 (bedroom); 0.103 mg/m3 (living room); 0.131 mg/m3 (kitchen); non-polluted homes: 0.43 mg/m3 (bedroom); 0.040 mg/m3 (living room); 0.047 mg/m3 (kitchen) | Subjects (including children with parental assistance) completed survey designed by the American Thoracic Society on respiratory health | Asthma, wheeze, respiratory symptoms for adults and children | Age, education, family history of allergy, gender, height, house facing, indoor domestic pets, occupation, smoking in the family, ventilation frequency, weight | Adult asthma prevalence = 0% for non-polluted homes, 1.68% for polluted homes, p-value reported not significant. Child asthma prevalence = 3.22% for non-polluted homes, 40% for polluted homes, p-value<0.05. Adult wheeze prevalence = 2.99% for non-polluted homes, 5.04% for polluted homes, p-value reported not significant. Child wheeze prevalence = 6.56% for non-polluted homes, 10% for polluted homes, p-value reported not significant. Adult OR=2.603, 95% CI [1.77, 3.828] for respiratory symptoms. Child OR=4.250, 95% CI [2.064, 8.753] for respiratory symptoms. |

**Children/Asthma Symptoms**

| **Study \(Study Design)** | **Study population & location** | **Sample size** | **Exposure assessment** | **Exposure ranges** | **Outcome assessment (not including pulmonary function tests)** | **Outcomes Reported** | **Confounders** | **Results** |
| --- | --- | --- | --- | --- | --- | --- | --- | --- |
| **Studies considered for meta-analysis** | | | | | | | | |
| Idavain et al. 2019 (Cross-sectional) | Children in the general population from 25 schools in Ida-Viru, Lääne-Viru and Tartu Counties in Estonia | 1326 children from randomly selected schools. Age of students range 8-12 years, | Annual mean concentrations of formaldehyde in 2013 were modelled | Range: 2.59-4.87 ug/m3 | Asthma-related outcomes were assessed through questionnaires that were distributed to students by teachers and completed by parent and child together. Questions inquired whether child ever had wheezing or whistling in the chest at any time in the past, whether they had asthma diagnosed by physician, whether child ever had attacks of asthma, and whether child had wheezing or whistling in the chest without cold in the past 12 months. Children were then invited for a clinical examination for further evaluation. | Asthma diagnosis, asthma symptoms (wheezing, whistling in chest, asthma attack) | Age, sex, BMI, parent's education and family income. | OR = 1.01, 95% CI: [0.90, 1.13] for every wheezing per 1 ug/m3 formaldehyde exposure. |
| Kim et al. 2007 (Cross-sectional) | Children in the general population attending all eight primary schools in Knivsta Municipality in rural outskirts of Uppsala City, Sweden | 1014 school children (ages 5-15 years, mean age 9 years) | Formaldehyde measured in three classrooms in each school for 6 hours in May-June 2000 | Mean for 23 classrooms: 7.13 ug/m3 (range 3-16) | Questionnaire completed by parents with cooperation of child in April-May 2000 and included questions about asthma (doctor-diagnosed, asthma medications, asthma attacks), airway symptoms during last year without using phrase "asthma" (wheezing, breathlessness) | Current asthma (diagnosed), current asthma medication, wheezing, nocturnal breathlessness, and daytime breathlessness | Age, gender | OR=1.03, 95% CI [0.86, 1.24] for nocturnal breathlessness per 1 ug/m3 increase in formaldehyde exposure in classroom. OR=0.96, 95% CI [0.87, 1.05] for wheeze per 1 ug/m3 increase in formaldehyde exposure in classroom. OR=0.96, 95% CI [0.85, 1.08] for daytime breathlessness per 1 ug/m3 increase in formaldehyde exposure in classroom. OR=1.02, 95% CI [0.93, 1.13] for doctor-diagnosed asthma per 1 ug/m3 increase in formaldehyde exposure in classroom. |
| Kim et al. 2011 (Cross-sectional) | Children in the general population attending 4th grade in twelve randomly-selected schools in three cities (Guri, Namyangju, and Chunchon) in Korea | 1915 total school children (mean age 10 years) | Formaldehyde measured continuously for 7 days in classrooms (n=34) and outside classroom windows (n=12) in November-December 2004 | Mean (classroom): 18.2 ug/m3 (SD 17.3; range 2.7-52.8); mean (outdoor): 16.5 ug/m3 (SD 12.5; range 3.3-45.3) | Questionnaire completed by subjects; current asthma defined as either having current medication or asthma attack in last 12 months | Doctor-diagnosed asthma, current asthma, wheeze | Age, sex, self-reported furry pet or pollen allergy, and home environment (remodeling, changing floor, age of home building, environmental tobacco smoke and indoor dampness) | OR=1.2, 95% CI [0.44, 3.24] self-reported wheezing during last 12 months per 10 ug/m3 increase in outdoor formaldehyde exposure. OR=1.15, 95% CI [0.88, 1.5] self-reported wheezing during last 12 months per 10 ug/m3 increase in indoor classroom formaldehyde exposure. OR=2.1, 95% CI [0.71, 6.23] current asthma (either having current asthma medication or having an asthma attack during the last 12 months) per 10 ug/m3 increase in outdoor formaldehyde exposure. OR=1.04, 95% CI [0.78, 1.4] current asthma per 10 ug/m3 increase in indoor classroom formaldehyde exposure. OR=0.8, 95% CI [0.22, 2.85] asthma diagnosis per 10 ug/m3 increase in outdoor formaldehyde exposure. OR=0.92, 95% CI [0.67, 1.26] asthma diagnosis per 10 ug/m3 increase in indoor classroom formaldehyde exposure. |
| Mi et al. 2006 (Cross-sectional) | Children in the general population attending junior high schools in central Shanghai and western Shanghai near Huang Pu river in China | 1414 total school children from 5 schools in each district participated by questionnaire in November 2000 (aged 12-14 years) | Formaldehyde measured in 30 classrooms for 4 hours using stationary monitor in November-December 2000 | Mean: 9.4 ug/m3 (SD 6.9; range 3-20) | Questionnaire completed by subjects and included questions about asthma (doctor-diagnosed, asthma medications, asthma attacks), airway symptoms during last year without using phrase "asthma" (wheezing, breathlessness) | Current asthma (diagnosed), asthma attack, medication use, current wheeze, nocturnal breathlessness, daytime breathlessness | Age, gender, indoor molds, smoking, water leakage | OR=1.01, 95% CI [0.56, 1.81] for current wheeze per 10 ug/m3 increase in formaldehyde exposure. OR=1.09, 95% CI [0.86, 1.38] for daytime breathlessness per 10 ug/m3 increase in formaldehyde exposure. OR=1.26, 95% CI [0.63, 2.53] for nocturnal breathlessness per 10 ug/m3 increase in formaldehyde exposure. OR=1.24, 95% CI [0.63, 2.45] for asthma attack per 10 ug/m3 increase in formaldehyde exposure. OR=1.26, 95% CI [0.65, 2.46] for asthma medication per 10 ug/m3 increase in formaldehyde exposure. OR=1.3, 95% CI [0.72, 2.32] for current asthma per 10 ug/m3 increase in formaldehyde exposure. |
| Rumchev et al. 2002 (Case-control) | Children in the general population with asthma identified by the Accident and Emergency Dept at the Princess Margaret Hospital for Children and nonasthmatic controls identified through the Health Dept of Western Australia, Perth, Western Australia | 88 asthmatic children (mean age 25 months) and 104 nonasthmatic controls (mean age 20 months) (ages 6 months-3 years) | Formaldehyde measured in the living room and child's bedroom for 8 hours during the day in July-September 1998 and December 1998-March 1999 | Mean bedroom: 30.2 ug/m3; mean living room: 27.5 ug/m3 | Asthma cases were children discharged with medical diagnosis of asthma from emergency department; questionnaire from American Thoracic Society completed by parents for respiratory symptoms (including wheeze) and home characteristics | Asthma diagnosis, wheeze | Age, air conditioning, allergen levels of house dust mite, atopy, child allergies, family history of asthma, humidifier and gas appliances, indoor air pollutants, indoor temperature, presence of pets, relative humidity, sex, smoking inside, socioeconomic status | OR=1.003, 95% CI [1.002-1.004] for asthma diagnosis per 10-unit (ug/m3) increase in formaldehyde exposure. Children who reported wheeze were also exposure to high average indoor levels of formaldehyde (40.5 ug/m3) compared to those without such symptoms (26.7 ug/m3) and the difference was significant (p<0.01) |
| Zhao et al. 2008 (Cross-sectional) | Children in the general population attending first year classes in 10 junior high schools within urban areas of Taiyun, China | 1993 school children (mean age 12.8 years) | Formaldehyde measured continuously for 7 days in one representative location in each school | Mean (classroom): 2.3 ug/m3 (SD 1.1; range 1.0-5.0); mean (outdoor): 5.8 ug/m3 (SD 0.6; range 5.0-7.0) | Questionnaire completed by subjects including questions on asthma (cumulative, doctor-diagnosed, and current) and on respiratory health (wheeze, breathlessness) based on International Study of Asthma and Allergy in Childhood (ISAAC) | Cumulative asthma (diagnosed), wheeze or whistling in the chest, nocturnal and daytime attacks of breathlessness | Personal and home environmental factors, age, environmental tobacco smoke at home, indoor and outdoor pollutants, new floor and new furniture in preceding 12 months, parental asthma or allergy, recent home painting, sex | OR=1.11, 95% CI [0.55, 2.23] for cumulative asthma per 1 ug/m3 increase in formaldehyde exposure indoor. OR=4.61, 95% CI [1.09, 19.5] for cumulative asthma per 1 ug/m3 increase in formaldehyde exposure outdoor. OR=0.93, 95% CI [0.78, 1.1] for daytime breathlessness per 1 ug/m3 increase in formaldehyde exposure indoor. OR=1.29, 95% CI [0.99, 1.68] for cumulative asthma per 1 ug/m3 increase in formaldehyde exposure outdoor. OR=1.11, 95% CI [0.87, 1.41] for wheeze or whistling in the chest per 1 ug/m3 increase in formaldehyde exposure indoor. OR=1.32, 95% CI [0.86, 2.04] for wheeze or whistling in the chest per 1 ug/m3 increase in formaldehyde exposure outdoor. OR=1.92, 95% CI [0.87, 1.41] for nocturnal attacks of breathlessness per 1 ug/m3 increase in formaldehyde exposure indoor. OR=2.03, 95% CI [0.91, 4.54] for nocturnal attacks of breathlessness per 1 ug/m3 increase in formaldehyde exposure outdoor. |
| **Studies not considered for meta-analysis** | | | | | | | | |
| Dannemiller et al. 2013 (Cross-sectional) | Children in the general population participating in formaldehyde monitoring study in primarily low-income homes in Boston, Massachusetts | 37 children (<18 years old) | Formaldehyde measured in homes between July 2008 and February 2010 | Geometric mean: 35.1 ppb (SD 1.98; range 5-132 ppb) | Questionnaire; completed by children >12 and parent for <12 | Asthma Control Test <12 (very poor control) | Age, gender, race, sources of ammonia and Nox in home. All participants were primarily low income. Data on type of housing, age of building home ownership, and resident smoking habits were recorded and considered. | Geometric mean of formaldehyde concentration for those with overall Asthma Control Test (ACT)<12 (very poor control) = 54.0ppb. Geometric mean formaldehyde for all other groups=34.4ppb, p-value=0.078. |
| Delfino et al. 2003  (Cross-sectional) | Children in the general population with physician-diagnosed asthma recruited through referrals from area schools in East Los Angeles County, California | 24 asthmatic children (between 10-15 years of age) | Formaldehyde measured by outdoor stationary monitoring stations between November 1999-January 2000 | Mean: 7.21 ppb (SD 2.41; range 4.27-14.02); interquartile range: 3.16 ppb; 90th percentile: 10.09 ppb | Questionnaire completed by subjects and included daily asthma symptoms (severity scale) and number of inhaler puffs | Asthma symptoms | Study exclusion criteria used to limit confounders such as active and passive smoking, and selected non-working Hispanic children. Confounding by weekend versus weekday, maximum temperature, and respiratory infections was also accounted for. All families in the study had low SES. | OR=1.3, 95% CI [0.33, 5.02] for bothersome or more severe asthma symptoms per 7.21ppb change (mean) in formaldehyde exposure, lag day 0. OR=7.3, 95% CI [1.46, 36.4] for bothersome or more severe asthma symptoms per 7.21ppb change (mean) in formaldehyde exposure, lag day 1. OR=2.27, 95% CI [0.43, 11.9] for asthma symptoms that interfered with daily activities per 7.21ppb change (mean) in formaldehyde exposure, lag day 0. OR=2.64, 95% CI [1.12, 6.21] for asthma symptoms that interfered with daily activities per 7.21ppb change (mean) in formaldehyde exposure, lag day 1. |
| Fsadni et al. 2018 (Prospective Cohort) | Children in the general population at five primary state school randomly selected from five geographical clusters in Malta | Sample size not reported. Age of students range 9-11 years. | Sampling took place over a 5-day period (Monday morning until Friday afternoon). Pollutant concentrations were averaged. | Indoor: mean 11.21 ug/m3 (sd: 2.95, range: 6.67-18.89). Outdoor: mean 1.81 ug/m3 (sd: 0.44, range: 1.37-2.4) | Standardized International Study of Asthma and Allergies in Childhood (ISAAC) questionnaire focusing on wheezing symptoms reported by parents | Asthma symptoms (wheezing), pulmonary lung function tests | No confounders or adjustment factors reported | No quantitative association estimates available. Authors only report that formaldehyde is associated with more likely current wheezing, but not exercise-induced wheezing or nocturnal cough. Quantitative estimates for associations are not reported. |
| Garrett et al. 1999 (note Garrett et al. 1998 used same cohort so combined to one record) (Cross-sectional) | Children in households in the general population recruited for study in Latrobe Valley, Victoria, Australia | 148 children (ages 7-14 years, mean age 10.2 years) from 80 households | Formaldehyde measured for four days in bedrooms of children, living rooms, kitchens, and outside the home between March-April, May, and September 1994 and January-February 1995 | Median: 15.8 ug/m3; maximum: 139 ug/m3 | Questionnaire completed by parents included questions on respiratory symptoms in previous year for cough, shortness of breath, wheeze, asthma attacks and chest tightness | Asthma, respiratory symptom score | Parental allergy and parental asthma | Mean respiratory score=1.09, 95% CI [0.42, 1.76] for formaldehyde exposure <20 ug/m3, 2.21, 95% CI [1.7, 2.75] for formaldehyde exposure 20-50 ug/m3, 2.59, 95% CI [1.67, 3.48] for formaldehyde exposure >50 ug/m3. Proportion asthmatic=16% for formaldehyde exposure <20 ug/m3, 39% for formaldehyde exposure 20-50 ug/m3, 44% for formaldehyde exposure >50 ug/m3. Bedroom formaldehyde-exposure groups showed no significant differences between groups, but there were significant differences between highest recorded formaldehyde level groups (chi-square=6.84, df=2, p=0.03). A higher proportion of asthmatics was seen with higher formaldehyde exposure, with a significant linear trend present (p=0.02). Adjusted odds ratio for asthma was not significantly different from 1.0 (exact OR not provided). |
| Jeong et al. 2011 (Cross-sectional) | Children in the general population attending second grade in 56 elementary schools (11 in Incheon, Korea and 45 in Jeju, Korea) | 1226 children attending Incheon schools (mean age 9.2 years) and 1748 children attending Jeju schools (mean age 9 years) | Formaldehyde measured in 11 Incheon schools and 2 Jeju schools as a single measurement in classrooms, cafeterias, infirmaries, playgrounds, and rooftops in December 2008 | Mean outdoor exposure: 28.64 ug/m3 (SD 65.26) for Incheon and 10.00 ug/m3 (SD 0) for Jeju; mean indoor exposure: 279.44 ug/m3 (SD 23.83) for Incheon and 196.67 ug/m3 (SD 87.31) for Jeju | ISAAC questionnaire completed by parents | Ever asthma, asthma treatment over last 12 months, ever wheezing or whistling, wheezing over last 12 months | Study groups were not statistically different in sex, height, and weight. Study schools where in industrial and non-industrial areas (proxies for SES), parental history of asthma, and age were also reported. Analyses were unadjusted t-tests and chi-squared tests. | N=159 (13.13%) with ever diagnosis of asthma for children living in Incheon (high levels of formaldehyde exposure) compared to N=230 (13.38%) children living in Jeju, p-value=0.47. N=50 (4.19%) with asthma treatment in last 12 months for children living in Incheon compared to N=64 (3.78%) children living in Jeju, p-value=0.57. N=304 (24.96%) with ever wheeze or whistling for children living in Incheon compared to N=321 (18.80%) children living in Jeju, p-value<0.01. N=116 (9.50%) with wheezing in last 12 months for children living in Incheon compared to N=115 (6.83%) children living in Jeju, p-value<0.01. |
| Lajoie et al. 2015 (Randomized controlled trial) | Children in the general population diagnosed with asthma at the Mother Child Centre and living in the greater Quebec area in Canada | 83 asthmatic children (ages 4-13 years) | Children randomly allocated to intervention and non-intervention groups and monitored for one year in pre-intervention stage and for one year in the post-intervention stage and formaldehyde measured in the child's bedroom over 6-8 day period 2009-2011 | Mean (fall/winter): 37.4 ug/m3 (95% CI 32.3-43.3) | Parents were interviewed and completed ISAAC questionnaire on respiratory symptoms including daily symptoms diary | Severe wheezing, effort wheezing, episodes of wheezing, hospitalizations, emergency room visits | Age, eczema | Change in annual prevalence of severe wheezing=1.5%, 95% CI [-20, 23.1] associated with 50% reduction of formaldehyde level from baseline, p-value-0.888. Change in annual prevalence of effort wheezing=-9.1%, 95% CI [-22.4, 4.1] associated with 50% reduction of formaldehyde level from baseline, p-value-0.173. Change in annual prevalence of >=1 episode of wheezing=-14.8%, 95% CI [-28.6, -0.9] associated with 50% reduction of formaldehyde level from baseline, p-value-0.037. Change in annual prevalence of >=4 episode of wheezing=-7.2%, 95% CI [-19.6, 5.3] associated with 50% reduction of formaldehyde level from baseline, p-value-0.255. Change in annual prevalence of >=1 emergency room visit=-16%, 95% CI [-30.5, -1.5] associated with 50% reduction of formaldehyde level from baseline, p-value-0.031. Change in annual prevalence of >=1 hospitalization=-7.9%, 95% CI [-20.6, 4.6] associated with 50% reduction of formaldehyde level from baseline, p-value-0.218. |
| Madureira et al. 2015 (note same cohort of children as Madureira et al. 2015b, but more comprehensive, so combined to one record) (Cross-sectional and case-control) | Children in the general population attending 20 public primary schools and a subset of asthmatic children in Porto, Portugal. Investigation was conducted between 2011-2013. | 1099 school children for classroom exposure measurements; 38 asthmatic children and 30 nonasthmatic children for home exposure measurements (ages 8-9 years) | School measurements: formaldehyde measured over 5 day period at breathing zone in 73 classrooms; Nov. 2011-Dec. 2012 and Nov. 2012-March 2013; Home measurements: formaldehyde measured over period of 7 days in rear of child's bedroom in Nov. 2011-Dec. 2012 and Nov. 2012-March 2013 | Median (school): 17.5 ug/m3; 25th percentile (school): 13.8 ug/m3; 75th percentile (school): 23.1 ug/m3; median (home): 11.4 ug/m3 for cases, 14.8 ug/m3 for controls | Parents completed paper-based questionnaire used in the International Study of Asthma and Allergies in Childhood; asthmatic cases answered yes to at least one question on asthma (doctor-diagnosed; wheezing in last year) | For school measurements: Asthma in school, doctor-diagnosed asthma, wheeze <30 d, wheeze <12 mo, ever wheeze, pulmonary function tests; for home measurements: asthma | No confounders or adjustment factors reported | No statistical comparison between formaldehyde levels between cases (asthmatic children) and controls |
| Madureira et al. 2016 (Cross-sectional and case-control) | A subset of children in the general population who were involved in a cross-sectional investigation recruited from 20 schools in Porto, Portugal | 38 homes of asthmatic children and 30 homes from nonsymptomatic children, average age 8.5 years | Between October 2012-April 2013 visual inspections and air sampling were performed in all homes. Samples were collected in the rear of children's bedroom 1-1.5m above the floor. Outdoor samples were collected when possible at heights of 1-2m above ground. | Mean (cases): 14.6 ug/m3 (sd=10.4); range= 3.68-50.7. Mean (controls): 16.6 ug/m3 (sd=9.49); range = 5.22-43.3 | Questionnaire based on the International Study of Asthma and Allergies in Childhood completed by legal guardians of children | Self-reported asthma symptoms--wheeze (ever wheeze, wheeze in the last 30 days), asthma in school. Reported on cases diagnosed with asthma by physician, but did not show data in publication. | No statistically significant differences in conditions between case and control groups, such as the presence of pets, use of air fresheners, incense stick, humidifiers, stuffed toys and smoking habits at home | p-value=0.199 comparing formaldehyde exposure between cases (asthmatic children) and control |
| Marks et al. 2010 (Non-randomized controlled trial) | Children in the general population in grades 4, 5, or 6 attending 22 schools in Blue Mountains, Southern Highlands, and Goulburn regions of NSW in Australia | 400 school children (mean age 11 years) | Classrooms were alternatively heated with a low-NOx unflued gas heater and with a flued gas heater for 3 weeks each ; formaldehyde measured in classrooms June-September 2009 | Overall: 28.6 ppb; flued heater: 24.7 ppb; unflued heater: 32.6 ppb | Daily symptom and medication diary | Evening wheeze, morning wheeze, evening symptoms: multiple symptoms, morning symptoms, cough or wheeze, bronchodilator use, asthma medication, pulmonary function tests | Exposure to environmental tobacco smoke, home use of gas, open fire for heating (asthma symptoms). Clustering by subject and school, day of the week, study week (pulmonary measures). | OR=1.123, 95% CI [0.856, 1.473] for evening wheeze comparing unflued gas heater to flued gas heater exposure. OR=1.603, 95% CI [1.171, 2.194] for morning wheeze comparing unflued gas heater to flued gas heater exposure. OR=0.963, 95% CI [0.852, 1.089] for evening symptoms comparing unflued gas heater to flued gas heater exposure. OR=0.938, 95% CI [0.801, 1.098] for morning symptoms (cough or wheeze) comparing unflued gas heater to flued gas heater exposure. OR=0.89, 95% CI [0.596, 1.329] for use of bronchodilator comparing unflued gas heater to flued gas heater exposure. Difference between unflued and flued heater exposure FEV1 in the morning mean=0.004, 95% CI [-0.009, 0.017]. Difference between unflued and flued heater exposure FEV1 in the evening mean=0, 95% CI [-0.014, 0.014]. Difference between unflued and flued heater exposure PEF in the morning mean=0.719, 95% CI [-1.239, 2.677]. Difference between unflued and flued heater exposure PEF in the evening mean=0.994, 95% CI [-0.995, 2.983]. |
| Neamtiu et al. 2019  (Prospective Cohort) | Children in the general population attending five public primary schools from Alba County in Romania as part of the SINPHONE cohort study | 280 students from 15 different classrooms in primary school (age not reported) | Formaldehyde exposures were measured for five days inside three classrooms and in one outside location at each school. | Indoor: Mean 34.16 ug/m3 (sd: 15.07, range: 15.50-66.19), Outdoor: Mean 9.50 ug/m3 (sd: 3.23, range: 6.03-12.90) | SINPHONE questionnaire completed by students inquiring about asthma-like symptoms in the past week | Asthma-like symptoms (difficulty breathing, dry cough, and wheezing in the past week) | Age, gender, NO2, CO, CO2, temperature, relative humidity, ventilation rate, and tobacco smoke exposure for the past week | OR = 2.69, 95% CI: [1.04, 6.97] for asthma-like symptoms from exposure formaldehyde concentration (higher formaldehyde (>=35 ug/m3) compared to lower (=35 ug/m3)) |
| Raaschou-Nielsen et al. 2010 (Prospective Cohort) | Infants in the general population born to mothers with asthma participating in Copenhagen Prospective Study on Asthma in Childhood (COPSAC) in Copenhagen, Denmark | 378 infants active in cohort at 18 months of age | Formaldehyde measured in children's bedrooms three times during first 18 months of life, for 10 weeks on each occasion | Mean: 20.3 ug/m3; median: 17.7 ug/m3; 5th-95th percentile: 7.9-36.7 ug/m3 | Questionnaire completed in daily diary by parents when children between 6-18 months of age | Wheezing symptoms | Baseline lung function, education of mother, residential area, sex | OR=1.11, 95% CI [0.47, 2.63] for wheezing symptoms comparing 2nd quintile (12.4-16.3 ug/m3) of formaldehyde exposure to 1st quintile (<12.4 ug/m3); OR=1.21, 95% CI [0.51, 2.92] for 3rd quintile (16.3-20.3 ug/m3) compared to first, OR=1.4, 95% CI [0.57, 3.47] for fourth quintile (20.3-25.6 ug/m3) compared to first; OR=0.67, 95% CI [0.29, 1.54] for 5th quintile (>25.6 ug/m3) compared to first |
| Schenker et al. 1982 (Cross-sectional) | Children and adult residents of six homes in the general population with urea formaldehyde foam insulation (UFFI) in the United States | 24 residents from six homes (ages 7-63 years) | Formaldehyde measured in homes 7 to 34 months following installation of UFFI in 1979-1981 | Range: 0.02-0.23 ppm | Subjects completed American Thoracic Society questionnaire and special supplementary questionnaire for subjects with formaldehyde exposure | Wheeze, pulmonary function tests | Authors report measuring smoking, age, and sex variables, but did not report these data and did not adjust analyses with these variables | Prevalence of participants reporting chronic phlegm=5/24 in UFFI houses. Prevalence of participants reporting chronic cough=11/24 in UFFI houses. Prevalence of participants reporting persistent wheeze=6/24 in UFFI houses. Change in FEV1 for participants in UFFI houses mean=-0.121. Change in FEV1/FVC for participants in UFFI houses mean=-3.74. |
| Smedje and Norback 2000 (Prospective Cohort) | Children in the general population attending 39 public schools in Uppsala county in Sweden | 1476 students in 1st, 4th or 7th grade (mean age 10.4 years in 1993 and 12.3 years in 1995) | Formaldehyde was measured for 4 hours in 2-5 secondary school classrooms and for each primary school classroom for each school in 1993 (prior to installation of new ventilation system) and in 1995 (after installation of new ventilation system) | Geometric mean 1993 (received new ventilation system later): 6 ug/m3; geometric mean 1993 (no new ventilation system): 3 ug/m3; change from 1993-1995 (new ventilation system): -4 ug/m3; change from 1993-1995 (no new ventilation system): 4 ug/m3 | Questionnaire completed by subjects in 1993 & 1995 included questions about asthma and asthma symptoms (amended from those used by ECRHS); current asthma defined as ever had asthma diagnosed by doctor and had at least one asthmatic symptom recently or using medication; asthmatic symptoms include recurrent persistent cough, persistent wheeze or shortness of breath, or during past 12 months had asthma attack, shortness of breath after exercise or nocturnal shortness of breath | Current asthma, ever asthma (doctor-diagnosed), any asthmatic symptoms, more than 1 asthmatic symptom | Age, atopy, smoking, sex | OR=0.3, 95% CI [0.1, 0.8] for any asthmatic symptoms comparing children with new ventilation to those without. OR=0.6, 95% CI [0.2, 2.8] for ever doctor's asthma diagnosis comparing children with new ventilation to those without. OR=1.2, 95% CI [0.4, 4.1] for current asthma comparing children with new ventilation to those without. OR=0.5, 95% CI [0.2, 0.97] for more asthmatic symptoms in 1995 than in 1993 comparing children with new ventilation to those without. |
| Tuthill 1984 (Cross-sectional) | Children in the general population attending a school system in Western Massachusetts | 399 children (youngest in household) | Formaldehyde exposure classified (Y/N) based on interview (new construction or remodeling, new upholstered furniture, foam insulation, living in mobile home) in April 1983 | Unknown | Phone interview completed by parents; questions included number of colds in school year, symptom checklist, length of episode and days of schools missed, physician-diagnosed chronic bronchitis, asthma, or allergies | Respiratory episodes (greater than or equal to 2) | Authors collected information on smoking, SES, education, age, sex, number of siblings, and woodstove exposures. Unclear how these factors were used for as potential adjustment factors. | Relative Risk=2.4, 95% CI [1.7, 3.4] for more than two respiratory episodes comparing those exposed versus those not exposed to formaldehyde. |
| Venn et al. 2003 (Case-control) | Children in the general population in primary schools participating in a study of traffic pollution exposure and childhood in 1995/1996 in Nottingham City, United Kingdom | 193 children with wheeze and 223 children with no reported wheeze (ages 9-11 years) | Formaldehyde measured in child's bedroom for 3 days between 1998-1999 | Range: 0->32 ug/m3 | Daily symptom diary over 4 weeks | Persisting wheezing illness, frequent daytime and nighttime respiratory symptoms | Age, Carstairs deprivation index, sex | OR=0.47, 95% CI [0.17, 1.25] for frequent daytime respiratory symptoms comparing 2nd quartile (16.1-22 ug/m3) of formaldehyde exposure to 1st quartile (<16 ug/m3); OR=2, 95% CI [0.71, 5.65] for 3rd quartile (22.1-32 ug/m3) compared to 1st, OR=2.08, 95% CI [0.71, 6.11] for fourth quartile (>32 ug/m3) compared to 1st, p-value for trend=0.05. OR=1.4, 95% CI [0.54, 3.62] for frequent nighttime respiratory symptoms comparing 2nd quartile of formaldehyde exposure to 1st quartile; OR=1.61, 95% CI [0.62, 4.19] for 3rd quartile compared to 1st, OR=3.33, 95% CI [1.23, 9.01] for fourth quartile compared to 1st, p-value for trend = 0.02. OR=1.14, 95% CI [0.65, 2] for persistent wheezing symptoms comparing 2nd quartile of formaldehyde exposure to 1st quartile; OR=1.08, 95% CI [0.62, 1.86] for 3rd quartile compared to 1st, OR=1.04, 95% CI [0.59, 1.82] for fourth quartile compared to 1st, p-value for trend = 0.93. |
| Willis et al. 2018 (Repeated cross-sectional) | Hospitalized children in the general population living in the entire state of Pennsylvania (67 total counties) | 15,837 children with pediatric asthma-related hospitalizations. Age range from 2-18 years. | Formaldehyde exposure was evaluated using the Pennsylvania Unconventional Natural Gas Emission Inventory which has annualized emissions data from Unconventional Gas Drilling (UNGD) sites. Pollutants reported in tons emitted per year and linked to participants' zip code. | Median: 0.00021 tons/year emissions (range: 0-22.51) | Asthma hospitalizations were obtained from the Pennsylvania Healthcare Cost Containment Council hospitalization data by identifying diagnostic codes with a 493 ICD-9 code, which indicates acute asthma exacerbation. | Asthma hospitalizations and exacerbations | Sex, race, year, quarter, insurance status, zip code respiratory hazard index, county median household income quartile, county unemployment, county poverty under 18 years old, and county log population density | OR = 1.2, 95% CI = [1.06, 1.36] for asthma hospitalizations for increasing formaldehyde exposure (log-sum emissions) |
| Yeatts et al. 2012 (Cross-sectional) | Children and adults in the general population recruited based on two-stage cluster sample design in the United Arab Emirates (UAE) | 1590 individuals within four age/sex categories: adult male (ages 19-50 years), adult female (ages 19-50 years), adolescent (ages 11-18 years), and child (ages 6-10 years) | Formaldehyde measured in the common living room for a 7 day period in 2009-2010 | Median: <7.37 ug/m3 (limit of quantification: 7.37; range: 7.37-168.2) | Subjects were interviewed and asked about ever having doctor-diagnosed asthma; respiratory symptoms assessed using ISAAC and Behavioral Risk Factor Surveillance System questions | Ever asthma, wheezing limited speech to 1 or 2 words between breaths, wheezing in last 4 months, wheezing in last 12 months, ever having wheezing and whistling in chest, shortness of breath one or more times a month, shortness of breath in last 12 months, chest tightness/difficulty breathing in last 12 months, chest tightness/difficulty breathing one or more times a month | Urban/rural area, household tobacco smoke exposure, gender, age group | OR=1.43, 95% CI [0.83, 2.46] for chest tightness/difficulty in breathing in last 12 months comparing high formaldehyde exposure group (7.37-168.2ppm) to low formaldehyde exposure group (<7.37ppm). OR=1.55, 95% CI [0.97, 2.48] for shortness of breath in last 12 months comparing high to low formaldehyde exposure group. OR=1.32, 95% CI [0.73, 2.37] for ever asthma comparing high to low formaldehyde exposure group. OR=1.31, 95% CI [0.71, 2.42] for ever having wheezing or whistling in chest comparing high to low formaldehyde exposure group. OR=0.64, 95% CI [0.21, 1.98] for wheezing in past 12 months comparing high to low formaldehyde exposure group. OR=3.48, 95% CI [0.81, 14.89] for wheezing in past 4 weeks comparing high to low formaldehyde exposure group. OR=4.18, 95% CI [1.23, 14.22] for wheezing limiting speech to 1 or 2 words between breaths comparing high to low formaldehyde exposure group. OR=3.68, 95% CI [1.11, 12.27] for shortness of breath one or more times a month comparing high to low formaldehyde exposure group. OR=6.52, 95% CI [1.91, 22.31] for chest tightness/difficulty in breathing one or more times a month comparing high to low formaldehyde exposure group. |
| Yon et al. 2019 (Prospective Cohort) | Children in the general population attending eleven elementary schools in Seongnam City, Korea | 427 students from 11 randomly selected classrooms, 10 with asthma. Age of students not reported, although all were in elementary school. | Formaldehyde concentrations were measured twice in each classroom (once in the first half and once in the second half of the academic year). | Average classroom concentration: 27.17 ug/m3 (+/-7.72, maximum 60 ug/m3) | Asthma defined by the presence of characteristic symptoms and/or signs during the previous 12 months, based on the International Study of Asthma and Allergies in Childhood questionnaire | Asthma symptoms | Age, sex, environmental tobacco smoke exposure, keeping a pet at home, and physician-diagnosed asthma and AD in parents | OR = 1.023, 95% CI: [0.960, 1.089] for asthma per 1 ug/m3 increase in indoor formaldehyde exposure |
| Zhai et al. 2013 (Cross-sectional) | Households of children and adults in the general population decorated within the previous four years in main urban area of Shenyang, China | One adult per household and 82 children in 186 residential houses | Formaldehyde measured in bedrooms, living rooms, and kitchens over 1 month to 3 years | Polluted homes: 0.093 mg/m3 (bedroom); 0.103 mg/m3 (living room); 0.131 mg/m3 (kitchen); non-polluted homes: 0.43 mg/m3 (bedroom); 0.040 mg/m3 (living room); 0.047 mg/m3 (kitchen) | Subjects (including children with parental assistance) completed survey designed by the American Thoracic Society on respiratory health | Asthma, wheeze, respiratory symptoms for adults and children | Age, education, family history of allergy, gender, height, house facing, indoor domestic pets, occupation, smoking in the family, ventilation frequency, weight | Adult asthma prevalence = 0% for non-polluted homes, 1.68% for polluted homes, p-value reported not significant. Child asthma prevalence = 3.22% for non-polluted homes, 40% for polluted homes, p-value<0.05. Adult wheeze prevalence = 2.99% for non-polluted homes, 5.04% for polluted homes, p-value reported not significant. Child wheeze prevalence = 6.56% for non-polluted homes, 10% for polluted homes, p-value reported not significant. Adult OR=2.603, 95% CI [1.77, 3.828] for respiratory symptoms. Child OR=4.250, 95% CI [2.064, 8.753] for respiratory symptoms. |

**Adult/Asthma Diagnosis**

| **Study**  **(Study Design)** | **Study population & location** | **Sample size** | **Exposure assessment** | **Exposure ranges** | **Outcome assessment (not including pulmonary function tests)** | **Outcomes Reported** | **Confounders** | **Results** |
| --- | --- | --- | --- | --- | --- | --- | --- | --- |
| **Studies considered for meta-analysis** | | | | | | | | |
| Krzyzanowski et al. 1990 (Cross-sectional) | Municipal employee households in the general population with adults and children 5-15 years of age in Pima County Arizona | 298 children (ages 6-15 years) and 613 adults | Formaldehyde measured in the kitchen, main living area and each subject's bedroom for two 1-week periods | Mean from 202 households: 26 ppb; maximum: 140 ppb | Questionnaire completed by subjects and included questions about asthma (doctor-diagnosed with assessment of current status) and chronic respiratory symptoms (cough/phlegm, wheezing, and shortness of breath with wheezing) | Asthma diagnosis, pulmonary function test | Current smoker, education, environmental tobacco smoke, race/ethnicity | Prevalence rates per 100 subjects of current diagnosed asthma for children: 11.7 (<=40 ppb formaldehyde), 4.2 (41-60ppb formaldehyde), 23.8 (>60ppb formaldehyde). Relation of PEFR (Liters/Minute) to indoor formaldehyde (ppb): -1.28 (mean) +/- 0.46 (SE) for children <15 years; 0.09 (mean) +/-0.27 (SE) for adults >15 years |
| **Studies not considered for meta-analysis** | | | | | | | | |
| Akbar Khanzadeh et al. 1994 (Cross-sectional) | Adult medical students exposed to formaldehyde in an occupational setting at an anatomy lab in Toledo, OH | 34 exposed subjects (mean age 26 years) and 12 nonmedical student controls (mean age 31.5 years) | Formaldehyde in breathing zone (personal sample) and general area anatomy lab measured on 9 days of work over period of 6 weeks in fall 1992 | Mean time weighted average (breathing zone): 1.24 ppm (SD 0.61; range 0.07-2.94); time weighted average (cavity): 0.49 ppm (SD 0.18); time weighted average (surface): 0.35 ppm (SD 0.13); time weighted average (area): 1.65 ppm (SD 0.92; range 1.00-2.30) | Questionnaire completed by subjects including questions from Medical Research Council standardized questionnaire; acute symptoms recorded prior to and following laboratory session | Asthma, shortness of breath, wheezing, pulmonary function tests | All subjects were nonsmokers, height and weight were similar between exposed versus non exposed. Slight differences in age and ethnicity. | Prevalence=7% for asthma for those exposed to formaldehyde versus 0% for those not exposed. No statistical association reported. Prevalence=7% for persistent wheezing for those exposed to formaldehyde versus 0% for those not exposed. No statistical association reported. Prevalence=2% for persistent shortness of breath for those exposed to formaldehyde versus 1% for those not exposed. No statistical association reported. Percent acute change in FVC=-1.4, 95% CI [-2.94, 0.14] for those exposed to formaldehyde versus -0.3, 95% CI [-3.22, 2.62] for those not exposed to formaldehyde, p-value<0.1. Percent acute change in FEV1=-0.03, 95% CI [-1.22, 1.16] for those exposed to formaldehyde versus 1, 95% CI [-1.54, 3.54] for those not exposed to formaldehyde. Percent acute change in FEV3=-1.2, 95% CI [-2.67, 0.27] for those exposed to formaldehyde versus 1.3, 95% CI [-0.79, 3.39] for those not exposed to formaldehyde. Percent acute change in FEF25-75%=2.5%, 95% CI [-0.54, 5.54] for those exposed to formaldehyde versus 2.31, 95% CI [0.59, 4.03] for those not exposed to formaldehyde. Percent acute change in FEV1/FVC=1.6%, 95% CI [0.27, 2.93] for those exposed to formaldehyde versus 0.6%, 95% CI [-1.24, 2.44] for those not exposed to formaldehyde, p-value<0.05. |
| Billionnet et al. 2011 (Cross-sectional) | Adults and children in the general population living in homes identified from population-based sample of French households | 1012 individuals over 15 years of age (median age 44 years) | Formaldehyde measured for one week in the bedroom of the reference person of the household; survey took place between October 2003 and December 2005 | Median: 19.4 ug/m3 (range 1.3-86.3) | Questionnaire completed by subjects; asthma in past year determined based on subjects answering "yes" to woken by shortness of breath or asthma attack in last year or current asthma medication (definition suggested by ECRHS) | Asthma | Age, highest educational level, outdoor pollution, presence of mold, presence of pets, relative humidity, sex, smoking, time of survey | OR=1.43 for asthma in the past year comparing high (>=28.03ug/m3) to low (<28.03ug/m3) formaldehyde exposure |
| Elshaer et al. 2017 (Cross-sectional) | Adult medical students exposed to formaldehyde in and occupational setting during dissections and staff members and workers within the Anatomy department at Alexandria University (Egypt) | 454 medical students in their first, second or third year and 16 exposed staff members and workers | Subjects classified as exposed to formalin or not exposed | Not measured--categorized as exposed versus not exposed | Students, staff and workers were subjected to a self-administered predesigned questionnaire to collect information on asthma-related outcomes | Work-related bronchial asthma, exacerbation of pre-existing bronchial asthma | No confounders or adjustment factors reported | Number (percentage) reporting prevalence of work-related bronchial asthma=8 (53.3%) for those exposed to formaldehyde, 0 (0%) for those not exposed to formaldehyde. Number (percentage) reporting exacerbation of pre-existing bronchial asthma=7 (46.7%) for those exposed to formaldehyde, 0 (0%) for those not exposed to formaldehyde. |
| Fornander et al. 2014 (Cross-sectional) | Adult metalworkers exposed to metal working fluids in an occupational setting in Sweden | 271 exposed subjects and 24 non-exposed controls | Formaldehyde measured using both stationary and personal monitors | Mean (stationary): 0.04 mg/m3; mean (personal): 0.1 mg/m3 | Questionnaire with asthma defined as "have or have had" (unclear if asthma was diagnosed or self-reported) | Asthma | No confounders or adjustment factors reported | Number and percent incidence of asthma cases by formaldehyde exposure group: 102 (11%) exposed directly, 169 (15%) exposed indirectly, 24 (17%) not exposed |
| Fransman et al. 2003 (Cross-sectional) | Adult plywood mill workers in an occupational setting in New Zealand | 112 workers and 415 general population controls (mean of 4.7 years employed at mill for group of workers; mean age 34.5 yr) | Formaldehyde measured using personal monitors | Geometric mean: 0.08 ug/m3 (SD 3.0); pressing section (high sample): 0.16 ug/m3 (SD 2.7) | Questionnaire completed by subjects and included questions on respiratory health symptoms; asthma prevalence estimated using ECRHS definition, which is based on proportion of subjects answering "yes" to woken by shortness of breath or asthma attack in last year or current asthma medication | Asthma, wheezing, shortness of breath or wheezing or chest tightness related to work, asthma medication, asthma attack, woken by shortness of breath, shortness of breath with wheezing, wheezing without a cold | Age, gender, race/ethnicity, smoking | OR=4.3, 95% CI [0.7, 27.7] for asthma comparing high formaldehyde exposure group (>=0.08 mg/m3) to low exposure (<0.08 mg/m3). OR=0.4, 95% CI [0, 5.4] for wheezing, shortness of breath or chest tightness related to work comparing high formaldehyde exposure group to low exposure. OR=1, 95% CI [0.1, 15.3] for asthma medication use comparing high formaldehyde exposure group to low exposure. OR=1.6, 95% CI [0.2, 13.2] for wheezing without a cold comparing high formaldehyde exposure group to low exposure. OR=3.5, 95% CI [0.6, 19.1] for shortness of breath with wheezing comparing high formaldehyde exposure group to low exposure. OR=9.5, 95% CI [1.2, 74.7] for woken by shortness of breath comparing high formaldehyde exposure group to low exposure. |
| Frey et al. 2014 (Cross-sectional) | Senior adults in the general population living in a single low-income senior housing building in Phoenix, Arizona | 72 senior residents (56 nonsmokers) | Formaldehyde measured in each apartment unit and outdoors for one hour between June-July 2010 | Range (indoor): 10-80 ppb; median (living room): 36.9 ppb; median (kitchen): 38.8 ppb; median (outdoor): 4.3 ppb | Questionnaire (unclear if asthma was diagnosed or self-reported) | Asthma | No confounders or adjustment factors reported | Authors report that 3 of 16 smokers and 6 or 56 nonsmokers reported asthma |
| Frisk et al. 2006 (Case-control) | Adults in the general population selected from the Orebro, Sweden section of the 1996 FinEsS study | 49 asthmatics and 48 non-asthmatic controls (ages 15-49 years) | Formaldehyde measured as mean 24-hour concentration in the bedroom between January-April 1999, October 1999-January 2000 | Mean: 23 ug/m3 (range <7-98) | Questionnaire completed by subjects; cases were people who replied "yes" to all questions on asthma (doctor diagnosis, asthma medications, asthma attacks/breathlessness in last 10 years and/or 12 months, wheezing within last year) | Asthma | Matched cases and controls based on age group, gender, and type of accommodation. | Mean 24hr formaldehyde levels in bedroom=27ug/m3 for asthma cases in single family housing, 12ug/m3 for asthma cases in multi-family housing, p-value=0.009. Mean 24hr formaldehyde levels in bedroom=26ug/m3 for controls in single family housing, 14ug/m3 for controls in multi-family housing, p-value=0.002. Mean 24hr formaldehyde levels in bedroom=33ug/m3 for asthma cases in homes built before 1975, 19ug/m3 for asthma cases in homes built after 1975, p-value=0.014. |
| Gorski et al. 1991 (Cohort) | Adult workers with respiratory symptoms and exposed to formaldehyde in an occupational setting at textile or shoe manufacturing factories (country not stated, but assume Poland) | 367 workers (ages 23-52 years, mean age 46 years) | Workers occupationally exposed to formaldehyde, exposure levels not reported | Not reported | Medical examination performed according to criteria of American Thoracic Society | Bronchial asthma and chronic bronchitis and dyspnoea, pulmonary function tests | Authors report information on smoking, age, and possible co-exposures, but these were not accounted for in analyses | Results are descriptive only, with no analyses of data. 14 subjects suffered from dyspnoea with clinical signs of chronic bronchitis; acute episodes of dyspnoea, classified as bronchial asthma, were found in 2 subjects, an illness lasted 2 and 7 years, respectively. Resting ventilatory function was measured by spirometry; vital capacity (VC), forced expiratory volume (FEV1) and peak expiratory flow (PEF) were estimated at the beginning of the work-shift and immediately afterwards; in subjects suffering from chronic cough, dyspnoea or sneezing, the test was repeated at the end of the work-shift and PEF was measured during the course of a three-day clinical observation. Mean values of ventilatory parameters in the group of 367 subjects were: VC = 3.47 ± 1.41L, FEV1 = 3.1 ± 0.19 L/s; in the group of bronchitic patients the mean value of VC was 3.03 ± 1.92 L and FEV, 2.13 ± 1.82 L/s; in 14 bronchitic patients the changes of PEF during the three-day observation did not exceed 20% of the initial value; only 2 patients reacted with a decrease of PEF at the end of a non-placebo, non-bronchodilatator day, but no difference between placebo- and-bronchodilatator days were found; the mean value of PC 20 in bronchitic patients was 5.61 mg/mL (± 1.79); in the 2 asthmatics a significant decrease from 3.41 to 1.97 mg/mL and from 2.70 to 2.01 mg/mL was noted after exposure. |
| Jacobsen et al. 2009 (Prospective Cohort) | Occupational cohort of adult workers exposed in woodworking factories and unexposed workers from control factories in Viborg, Denmark | 1377 woodworkers and 297 control workers (male mean age 38.4 years, female mean age 37.8 years) | Formaldehyde measured using personal monitors in 24 samples from 10 factories in 2003-20005 | Median: 0.05 mg/m3 (range 0.03-0.2) | Questionnaire completed by subjects included questions from ECRHS; ever asthma defined as current or ever self-reported asthma; asthma symptoms defined as yes to at least one group A question (doctor-diagnosed asthma, ever had asthma, current asthma, wheeze) and 2 or more group B questions (chest tightness; wake with chest tightness; wake with wheezing; cough in morning; wheeze in cold air; wheeze with exercise; wheeze from pollen; wheeze from animals; asthma medication) | Current asthma, ever asthma, asthma symptoms, ever wheezing | Age, baseline hay fever, smoking | OR=1.5, 95% CI [0.34, 6.51] for current asthma comparing male woodworkers to male reference workers. OR=6.89, 95% CI [0.85, 55.8] for current asthma comparing female woodworkers to female reference workers. OR=1.9, 95% CI [0.44, 9.12] for ever asthma comparing male woodworkers to male reference workers. OR=3.37, 95% CI [0.91, 12.5] for ever asthma comparing female woodworkers to female reference workers. OR=0.73, 95% CI [0.4, 1.33] for ever wheeze comparing male woodworkers to male reference workers. OR=1.58, 95% CI [0.73, 3.42] for ever wheeze comparing female woodworkers to female reference workers. OR=0.75, 95% CI [0.39, 1.45] for respiratory symptoms comparing male woodworkers to male reference workers. OR=1.31, 95% CI [0.6, 2.83] for respiratory symptoms comparing female woodworkers to female reference workers. |
| Kilburn et al. 1985 (Prospective Cohort) | Occupational cohort of adult male fiberglass batt makers, histology technicians and hospital workers in California | 20 exposed batt makers, 20 unexposed batt makers, 18 histology technicians, 26 unexposed hospital workers (ages 20-62 years) | Formaldehyde measured for histology workers, and batt makers were assumed to have higher levels; self-administered questionnaire asked about exposures to formaldehyde and competing or confounding exposures | Range (histology): 0.4-1.9 ppm; assumed higher exposure levels for batt makers | Questionnaire completed by subjects included questions on respiratory disease history and symptoms | Asthma, breathlessness, wheezing, chest tightness and pain/burning, shortness of breath at work, shortness of breath at rest, respiratory mean score, pulmonary function tests | No confounders or adjustment factors reported | Incidence asthma for hot batt makers=5% (n=20), cold batt makers=15% (n=25), histology=6% (n=18), comparison group=9% (n=26). Incidence breathlessness for hot batt makers=35%, cold batt makers=44%, histology=6%, comparison group=12%. Incidence wheezing for hot batt makers=50%, cold batt makers=36%, histology=6%, comparison group=12%. Incidence chest tightness for hot batt makers=50%, cold batt makers=40%, histology=11%, comparison group=0%. Incidence shortness of breath at rest for hot batt makers=30%, cold batt makers=24%, histology=6%, comparison group=0%. Incidence shortness of breath at work for hot batt makers=40%, cold batt makers=40%, histology=11%, comparison group=4%. Mean respiratory mean score for hot batt makers=5.8, cold batt makers=4.9, histology=1.6, comparison group=0.8. Percentage of workers who decreased FVC by 5% or more of their before-shift values for hot batt makers (noncigarette smokers)=22.2%, bat makers (cigarette smokers)=8.6%, p-value<0.01. Percentage of workers who decreased FEV1 by 10% or more of their before-shift values for hot batt makers (noncigarette smokers)=33.3%, bat makers (cigarette smokers)=11.4%, p-value<0.01. Percentage of workers who decreased FEF25-75 by 15% or more of their before-shift values for hot batt makers (noncigarette smokers)=33.3%, bat makers (cigarette smokers)=11.4%, p-value<0.01. Percentage of workers who decreased FEF75-85 by 15% or more of their before-shift values for hot batt makers (noncigarette smokers)=22.2%, bat makers (cigarette smokers)=40%, p-value<0.01. |
| Low et al. 1985 (Cross-sectional) | Adult workers exposed to formaldehyde and unexposed controls in an occupational setting at a foundry in Brisbane, Australia | 46 exposed workers and 17 controls (mean ages range from 25.3-39.1 years based on work area) | Formaldehyde exposure classified based on job | Core shop: not detected; general foundry: 2-4 ppm; shell: <LOD (1 ppm) | Modified standardized questionnaire from Medical Research Council completed by subjects | Asthma or wheeze onset before at foundry and while at foundry, wheeze at work, pulmonary function tests | Authors reported no differences in cigarette smoking between comparison groups, but noted some differences in age | Number (number attributing to specific environmental factor at work) of prevalence of asthma or wheeze onset while at foundry=3 (1), onset before at foundry=1 (0), wheezing while at work 1. FVC measured on Monday morning mean=91.4, 95% CI [85.89, 96.91] for aftercast (not formaldehyde exposed) versus FVC measured on Monday morning mean=84.1, 95% CI [77.88, 90.32] for general foundry workers (formaldehyde exposed), not statistically significant. Change in FEV1 measured on Monday and Friday mean=-8 mL, 95% CI [-92.8, 76.8] for aftercast versus change in FEV1 measured on Monday and Friday mean=4 mL, 95% CI [-62.49, 70.49] for general foundry workers, not statistically significant. Change in FEV1 over the work week mean=-15 mL, 95% CI [-201.55 171.55] for aftercast versus change in FEV1 over the work week mean=-105 mL, 95% CI [-220.82, 10.82] for general foundry workers, not statistically significant. FEV1/FVC% measured on Monday morning mean=80.4, 95% CI [76.58, 84.22] for aftercast versus FEV1/FVC% measured on Monday morning=83.1 mL, 95% CI [78.81, 87.39] for general foundry workers, not statistically significant. FEV1 measured on Monday morning mean=89.5, 95% CI [82.93, 96.07] for aftercast versus FEV1 measured on Monday morning mean=84.6, 95% CI [76.66, 92.54] for general foundry workers, not statistically significant. Change in FEV1 measured at beginning and end of Monday mean=-9 mL, 95% CI [-115, 97] for aftercast versus FEV1 measured at beginning and end of Monday mean=-57, 95% CI [-187.83, 73.83] for general foundry workers, not statistically significant. |
| Malaka et al. 1990 (Cross-sectional) | Male workers exposed to formaldehyde and nonexposed controls at plywood plant in East Java, Indonesia | 55 exposed male workers (mean age 26.6 years) and 50 unexposed male controls (mean age 28.8 years) | Estimate of cumulative formaldehyde exposure calculated from area concentrations and length of service in current job | Mean (exposed group): 6.29 ppm-yr (SD 2.72); range (area concentrations): 0.22-3.48 ppm | Standardized respiratory questionnaire from American Thoracic Society completed by subjects | Asthma, occupational asthma, wheezing, shortness of breath, pulmonary function tests | Age, dust, smoking status, cigarettes per day, weight, height | OR=6.31 for asthma comparing those exposed to formaldehyde versus not, p-value=0. OR=2.84 for occupational asthma comparing those exposed to formaldehyde versus not, p-value=0.02. OR=1.98 for shortness of breath comparing those exposed to formaldehyde versus not, p-value=0.04. OR=1.2 for wheezing comparing those exposed to formaldehyde versus not, p-value=0.36. Mean FEV1=2.78L, 95% CI [2.7, 2.86] comparing those exposed to formaldehyde versus not, p-value=0.001. Mean FEV1/FVC=3% comparing those exposed to formaldehyde versus not. Mean FEF25%-75%=3.44L/s, 95% CI [3.28, 3.6] for those not exposed to formaldehyde, mean FEF25%-75%=3.04L/s, 95% CI [2.88, 3.2] for those exposed to formaldehyde, p-value=0. Multiple regression coefficient=-0.043 for FEF25%-75% for continuous formaldehyde exposure, p-value<0.05. |
| Mapou et al. 2013 (Cross-sectional) | Adults in the general population participating in Relationship of Indoor, Outdoor and Personal Air (RIOPA) Study in communities in Los Angeles County, CA, Elizabeth, NJ, and Houston, TX | 90 adults | Formaldehyde measured in personal passenger vehicles from July 1999-February 2001 | Median: 20.0 mg/m3 (range <4.65-1095.6) | Self-reported doctor-diagnosed | Asthma | In general, authors adjusted for type of vehicle driven, season in which sampling occurred, total minutes driven, and relative humidity but authors did not state which of these factors was included in the analysis of formaldehyde and asthma association. Authors reported on the gender, education level, and household income. | OR=1.03, p-value=0.054 for self-reported doctor-diagnosed asthma per 1 ug/m3 change in formaldehyde exposure in vehicle. Correlation=0.27, p-value=0.004 between formaldehyde exposure (measured inside vehicles) and self-reported doctor-diagnosed asthma |
| Matsunaga et al. 2007 (Cross-sectional) | Pregnant adult women in the general population from Osaka Maternal and Child Health Study in multiple municipalities in Japan | 998 pregnant women | Formaldehyde measured using personal monitors November-March 2003 | Median: 24 ppb; maximum: 131 ppb | Questionnaire completed by subjects and included questions on asthma; asthma considered present if received medical treatment during last year | Current asthma | Age, allergic rhinitis, atopic eczema, current passive smoking at home and work, education, family history of allergy, family income, gestation, indoor domestic pets, mite antigen in house dust, mold in the kitchen, parity, season, smoking | OR=0.8, 95% CI [0.23, 2.84] for current asthma comparing second quartile of formaldehyde exposure (18-27 ug/m3) compared to first quartile (<18 ug/m3), OR=0.72, 95% CI [0.19, 2.77] for current asthma comparing third quartile of formaldehyde exposure (28-46 ug/m3) compared to first quartile, OR=2.15, 95% CI [0.41, 11.28] for current asthma comparing fourth quartile of formaldehyde exposure (>=47 ug/m3) compared to first quartile. p-value for trend=0.47. OR=2.65, 95% CI [0.63, 11.11] for current asthma comparing above 90th percentile formaldehyde exposure (>=47 ug/m3) to those below. Authors report no statistically significant difference. |
| Pourmabahabadian et al. 2006 (Cross-sectional) | Adult workers in an occupational setting at 7 hospitals of Tehran University of Medical Sciences in Tehran, Iran | 180 exposed workers from pathology labs (n=38), surgery rooms (n=65), and endoscopy (n=21) and 56 unexposed controls working in administrative affairs section | Formaldehyde measured as 8 hour continuous and spot samples in different departments of 7 hospitals in 2002-2003 | Mean 8 hour sample: 0.96 ppm (pathology), 0.13 ppm (endoscopy), 0.25 ppm (surgery) | Questionnaire completed by subjects | Asthma, chest tightness, pulmonary function tests | Report on smoking, sex and age but do not adjust results for covariates | Percentage reporting asthma=7.9% (pathology), 19% (endoscopy), 1.5% (surgery) versus 5.4% (nonexposed). No statistical association reported. Percentage reporting chest tightness=31.5% (pathology), 28.6% (endoscopy), 27.7% (surgery) versus 16.1% (nonexposed). No statistical association reported. FEV1 mean=2.3L, 95% CI [2.12, 2.48] for those exposed to formaldehyde versus mean=2.9L, 95% CI [2.58, 3.22] for those not exposed to formaldehyde, p-value<0.001. FVC mean=3.3L, 95% CI [3.12, 3.48] for those exposed to formaldehyde versus mean=4L, 95% CI [3.68, 4.32] for those not exposed to formaldehyde, p-value<0.001. FEV1/FVC mean=69.7%, 95% CI [66.02, 73.38] for those exposed to formaldehyde versus mean=72.5%, 95% CI [66.02, 78.98] for those not exposed to formaldehyde, p-value non-significant. FEF25-75 mean=2.66L/s, 95% CI [2.39, 2.93] for those exposed to formaldehyde versus mean=3.35L/s, 95% CI [2.92, 3.78] for those not exposed to formaldehyde, p-value<0.006. |
| Thetkathuek et al. 2016 (Cross-sectional) | Adult employees exposed to formaldehyde in an occupational setting at a MDF furniture factory in Thailand | 432 volunteers (out of 535 factory workers) | Formaldehyde measured from five work sites in factory for 21 continuous hours on two separate days in March and April 2012 | Formaldehyde exposures were classified as Low (0.66-3.44 ppm), Moderate (3.45-6.89 ppm), or High (>6.89ppm) | Questionnaire based on the American Thoracic Society Respiratory Symptoms Questionnaire, adjusted to fit working conditions in the furniture factory. Questionnaires were completed by the study subjects independently. | Atopic allergic asthma, asthma symptoms (wheeze) | Authors consider variables education, atopic eczema, allergic asthma, allergic rhinitis history, family history, formaldehyde concentrations or MDF dust concentrations, but unclear whether these are the confounder variables in the adjusted analysis reported. | Number (percentage) reporting prevalence of having wheeze during the daytime or nighttime=56 (15%) for low formaldehyde exposure (0.66-3.44 ug/m3), 2 (4.5%) for moderate formaldehyde exposure (3.45-6.89 ug/m3), and 4 (18.2%) for high formaldehyde exposure (>6.89 ug/m3) |
| Veremchuk et al. 2016 (Cross-sectional) | Adult and children residents in the general population with asthma in Vladivostok, Russia | Asthma morbidity in Vladivostok (sample size not reported) | Formaldehyde measured using air quality monitors from six stationary observation posts during 2008-2012 | Not reported | Medical records | Asthma diagnosis | The study evaluated effects by age groups (children, adolescents, and adults). Climatic factors and indicators of anthropogenic air pollution in the city were also evaluated. | No significant correlation reported between formaldehyde exposure and asthma outcome |
| Wieslander et al. 1997 (Cross-sectional) | Adults in the general population randomly selected from population register in Uppsala, Sweden | 562 adults (mean age 32 years) | Formaldehyde measured in the bedroom of a random sample of dwellings of 62 participants | Mean (wall/ceiling painted): 16 ug/m3 (yes) and 21 ug/m3 (no); mean (wood painted): 32 ug/m3 (yes) and 17 ug/m3 (no); mean (kitchen painted): 18 ug/m3 (yes) and 20 ug/m3 (no); mean (bedroom painted): 24 ug/m3 (yes) and 19 ug/m3 (no); mean (bathroom painted): 19 ug/m3 (yes) and 20 ug/m3 (no) | International Union Against Tuberculosis and Lung Disease questionnaire completed by subjects; current asthma defined as combination of bronchial hyperresponsiveness and at least one symptom related to asthma in last year; symptoms included wheezing, shortness of breath, nighttime awakening from breathlessness or tightness of chest | Asthma, wheezing, at least one asthma symptom, daytime and nocturnal breathlessness, pulmonary bronchial hyperresponsiveness | Age, gender, smoking | OR=1.56, 95% CI [0.98, 2.48] for increased prevalence of newly painted dwelling for those reporting asthma v. those not. OR=1.13, 95% CI [0.63, 2.01] for increased prevalence of newly painted workplace for those reporting asthma v. those not. OR=2.33, 95% CI [1.22, 4.46] for increased prevalence of wood painted for those reporting asthma v. those not. OR=2.21, 95% CI [1.09, 4.51] for increased prevalence of kitchen painted for those reporting asthma v. those not. OR=1.21, 95% CI [0.83, 1.76] for increased prevalence of newly painted dwelling for those reporting wheezing v. those not. OR=1.6, 95% CI [1.02, 2.52] for increased prevalence of newly painted workplace for those reporting wheezing v. those not. OR=1.6, 95% CI [0.92, 2.78] for increased prevalence of wood painted for those reporting wheezing v. those not. OR=1.7, 95% CI [0.92, 3.16] for increased prevalence of kitchen painted for those reporting wheezing v. those not. OR=1.16, 95% CI [0.75, 1.79] for increased prevalence of newly painted dwelling for those reporting daytime breathlessness v. those not. OR=1.6, 95% CI [0.96, 2.67] for increased prevalence of newly painted workplace for those reporting daytime breathlessness v. those not. OR=1.94, 95% CI [1.07, 3.5] for increased prevalence of wood painted for those reporting daytime breathlessness v. those not. OR=1.66, 95% CI [0.84, 3.3] for increased prevalence of kitchen painted for those reporting daytime breathlessness v. those not. OR=1.57, 95% CI [1.05, 2.36] for increased prevalence of newly painted dwelling for those reporting nocturnal breathlessness v. those not. OR=1.35, 95% CI [0.82, 2.22] for increased prevalence of newly painted workplace for those reporting nocturnal breathlessness v. those not. OR=1.75, 95% CI [0.98, 3.14] for increased prevalence of wood painted for those reporting nocturnal breathlessness v. those not. OR=2.67, 95% CI [1.42, 5.04] for increased prevalence of kitchen painted for those reporting nocturnal breathlessness v. those not. OR=1.43, 95% CI [1.01, 2.06] for increased prevalence of newly painted dwelling for those reporting at least one asthma symptom v. those not. OR=1.63, 95% CI [1.05, 2.54] for increased prevalence of newly painted workplace for those reporting at least one asthma symptom v. those not. OR=1.8, 95% CI [1.04, 3.12] for increased prevalence of wood painted for those reporting at least one asthma symptom v. those not. OR=2.24, 95% CI [1.2, 4.21] for increased prevalence of kitchen painted for those reporting at least one asthma symptom v. those not. OR=1.37, 95% CI [0.88, 2.13] for increased prevalence of newly painted dwelling for those reporting bronchial hyperresponsiveness v. those not. OR=1.25, 95% CI [0.73, 2.14] for increased prevalence of newly painted workplace for those reporting bronchial hyperresponsiveness v. those not. OR=2, 95% CI [1.06, 3.76] for increased prevalence of wood painted for those reporting bronchial hyperresponsiveness v. those not. OR=2.14, 95% CI [1.08, 4.23] for increased prevalence of kitchen painted for those reporting bronchial hyperresponsiveness v. those not. |
| Yeatts et al. 2012 (Cross-sectional) | Children and adults in the general population recruited based on two-stage cluster sample design in the United Arab Emirates (UAE) | 1590 individuals within four age/sex categories: adult male (ages 19-50 years), adult female (ages 19-50 years), adolescent (ages 11-18 years), and child (ages 6-10 years) | Formaldehyde measured in the common living room for a 7 day period in 2009-2010 | Median: <7.37 ug/m3 (limit of quantification: 7.37; range: 7.37-168.2) | Subjects were interviewed and asked about ever having doctor-diagnosed asthma; respiratory symptoms assessed using ISAAC and Behavioral Risk Factor Surveillance System questions | Ever asthma, wheezing limited speech to 1 or 2 words between breaths, wheezing in last 4 months, wheezing in last 12 months, ever having wheezing and whistling in chest, shortness of breath one or more times a month, shortness of breath in last 12 months, chest tightness/difficulty breathing in last 12 months, chest tightness/difficulty breathing one or more times a month | Urban/rural area, household tobacco smoke exposure, gender, age group | OR=1.43, 95% CI [0.83, 2.46] for chest tightness/difficulty in breathing in last 12 months comparing high formaldehyde exposure group (7.37-168.2ppm) to low formaldehyde exposure group (<7.37ppm). OR=1.55, 95% CI [0.97, 2.48] for shortness of breath in last 12 months comparing high to low formaldehyde exposure group. OR=1.32, 95% CI [0.73, 2.37] for ever asthma comparing high to low formaldehyde exposure group. OR=1.31, 95% CI [0.71, 2.42] for ever having wheezing or whistling in chest comparing high to low formaldehyde exposure group. OR=0.64, 95% CI [0.21, 1.98] for wheezing in past 12 months comparing high to low formaldehyde exposure group. OR=3.48, 95% CI [0.81, 14.89] for wheezing in past 4 weeks comparing high to low formaldehyde exposure group. OR=4.18, 95% CI [1.23, 14.22] for wheezing limiting speech to 1 or 2 words between breaths comparing high to low formaldehyde exposure group. OR=3.68, 95% CI [1.11, 12.27] for shortness of breath one or more times a month comparing high to low formaldehyde exposure group. OR=6.52, 95% CI [1.91, 22.31] for chest tightness/difficulty in breathing one or more times a month comparing high to low formaldehyde exposure group. |
| Zhai et al. 2013 (Cross-sectional) | Households of children and adults in the general population decorated within the previous four years in main urban area of Shenyang, China | One adult per household and 82 children in 186 residential houses | Formaldehyde measured in bedrooms, living rooms, and kitchens over 1 month to 3 years | Polluted homes: 0.093 mg/m3 (bedroom); 0.103 mg/m3 (living room); 0.131 mg/m3 (kitchen); non-polluted homes: 0.43 mg/m3 (bedroom); 0.040 mg/m3 (living room); 0.047 mg/m3 (kitchen) | Subjects (including children with parental assistance) completed survey designed by the American Thoracic Society on respiratory health | Asthma, wheeze, respiratory symptoms for adults and children | Age, education, family history of allergy, gender, height, house facing, indoor domestic pets, occupation, smoking in the family, ventilation frequency, weight | Adult asthma prevalence = 0% for non-polluted homes, 1.68% for polluted homes, p-value reported not significant. Child asthma prevalence = 3.22% for non-polluted homes, 40% for polluted homes, p-value<0.05. Adult wheeze prevalence = 2.99% for non-polluted homes, 5.04% for polluted homes, p-value reported not significant. Child wheeze prevalence = 6.56% for non-polluted homes, 10% for polluted homes, p-value reported not significant. Adult OR=2.603, 95% CI [1.77, 3.828] for respiratory symptoms. Child OR=4.250, 95% CI [2.064, 8.753] for respiratory symptoms. |

**Adult/Asthma Symptoms**

| **Study**  **(Study Design)** | **Study population & location** | **Sample size** | **Exposure assessment** | **Exposure ranges** | **Outcome assessment (not including pulmonary function tests)** | **Outcomes Reported** | **Confounders** | **Results** |
| --- | --- | --- | --- | --- | --- | --- | --- | --- |
| **Studies considered for meta-analysis (None)** | | | | | | | | |
| **Studies not considered for meta-analysis** | | | | | | | | |
| Akbar Khanzadeh et al. 1994 (Cross-sectional) | Adult medical students exposed to formaldehyde in an occupational setting at an anatomy lab in Toledo, OH | 34 exposed subjects (mean age 26 years) and 12 nonmedical student controls (mean age 31.5 years) | Formaldehyde in breathing zone (personal sample) and general area anatomy lab measured on 9 days of work over period of 6 weeks in fall 1992 | Mean time weighted average (breathing zone): 1.24 ppm (SD 0.61; range 0.07-2.94); time weighted average (cavity): 0.49 ppm (SD 0.18); time weighted average (surface): 0.35 ppm (SD 0.13); time weighted average (area): 1.65 ppm (SD 0.92; range 1.00-2.30) | Questionnaire completed by subjects including questions from Medical Research Council standardized questionnaire; acute symptoms recorded prior to and following laboratory session | Asthma, shortness of breath, wheezing, pulmonary function tests | All subjects were nonsmokers, height and weight were similar between exposed versus non exposed. Slight differences in age and ethnicity. | Prevalence=7% for asthma for those exposed to formaldehyde versus 0% for those not exposed. No statistical association reported. Prevalence=7% for persistent wheezing for those exposed to formaldehyde versus 0% for those not exposed. No statistical association reported. Prevalence=2% for persistent shortness of breath for those exposed to formaldehyde versus 1% for those not exposed. No statistical association reported. Percent acute change in FVC=-1.4, 95% CI [-2.94, 0.14] for those exposed to formaldehyde versus -0.3, 95% CI [-3.22, 2.62] for those not exposed to formaldehyde, p-value<0.1. Percent acute change in FEV1=-0.03, 95% CI [-1.22, 1.16] for those exposed to formaldehyde versus 1, 95% CI [-1.54, 3.54] for those not exposed to formaldehyde. Percent acute change in FEV3=-1.2, 95% CI [-2.67, 0.27] for those exposed to formaldehyde versus 1.3, 95% CI [-0.79, 3.39] for those not exposed to formaldehyde. Percent acute change in FEF25-75%=2.5%, 95% CI [-0.54, 5.54] for those exposed to formaldehyde versus 2.31, 95% CI [0.59, 4.03] for those not exposed to formaldehyde. Percent acute change in FEV1/FVC=1.6%, 95% CI [0.27, 2.93] for those exposed to formaldehyde versus 0.6%, 95% CI [-1.24, 2.44] for those not exposed to formaldehyde, p-value<0.05. |
| De Vos et al. 2009  (Non-randomized controlled trial) | Active adult fire fighters exposed in an occupational setting based in Perth Metropolitan fire stations in Australia | 67 fire fighters participated in four field trials where randomly allocated to groups using respirators with different filters (P, POV, POVF filters) | Formaldehyde measured using personal air samples inside respirators; performed 120 min and 60 min burns | Mean (120 min): 0.245 mg/m3 for P filter, 0.021 for POV filter, 0.017 mg/m3 for POVF filter; mean (60 min): 0.44 mg/m3 for P filter, 0.027 mg/m3 for POV filter, 0.015 mg/m3 for POVF filter | Subjects completed questionnaire based on Medical Research Council questionnaire on respiratory symptoms | Respiratory symptoms | FESA years, age group, pack years | OR for increase in respiratory symptoms=0.050, 95% CI [0.004, 0.597] for 0-60 minute P filter versus POV filter, OR=0.234, 95% CI [0.068, 0.797] for 0-60 minute P filter versus POVF filter, OR=0.484, 95% CI [0.034, 6.802] for 0-60 minute POV filter versus POVF filter. OR=0.048, 95% CI [0.006, 0.358] for 0-120 minute P filter versus POV filter, OR=0.237, 95% CI [0.092, 0.613] for 0-120 minute P filter versus POVF filter, OR=1.300, 95% CI [0.149, 11.359] 0-120 minute POV filter versus POVF filter. |
| Dumas et al. 2017  (Case control study nested within prospective cohort) | Adult nurses exposed to formaldehyde in an occupational setting while employed and working within the United States | 4,102 actively employed nurses with physician-diagnosed asthma and use of asthma medication in the past year | Formaldehyde exposure classified by nurse-specific job-task-exposure matrix designed to assign exposure level to formaldehyde as low, medium or high based on a combination of types of nursing jobs and general disinfection tasks | Not measured--categorized as high versus low exposure based on job function | Questionnaire completed by subjects with information regarding diagnosis by physician as having asthma, reported use of asthma medication in the past year, use of prescribed long-term preventative medicine, and Asthma Control Test score (range 5-25) based on five questions on activity limitations, frequency of symptoms and frequency of use of quick-relief medication in past four weeks | Categorical Asthma Control Test (ACT), asthma medication | Age, smoking status, body mass index, race and ethnicity | OR=1.33, p-value=0.02 for decrease in Asthma Control Test comparing high versus low formaldehyde exposure, based on job-task-exposure-matrix (JTEM) defining high as task performed 1-3 or 4-7 days per week versus never or <1 day per week. |
| Fransman et al. 2003 (Cross-sectional) | Adult plywood mill workers in an occupational setting in New Zealand | 112 workers and 415 general population controls (mean of 4.7 years employed at mill for group of workers; mean age 34.5 yr) | Formaldehyde measured using personal monitors | Geometric mean: 0.08 ug/m3 (SD 3.0); pressing section (high sample): 0.16 ug/m3 (SD 2.7) | Questionnaire completed by subjects and included questions on respiratory health symptoms; asthma prevalence estimated using ECRHS definition, which is based on proportion of subjects answering "yes" to woken by shortness of breath or asthma attack in last year or current asthma medication | Asthma, wheezing, shortness of breath or wheezing or chest tightness related to work, asthma medication, asthma attack, woken by shortness of breath, shortness of breath with wheezing, wheezing without a cold | Age, gender, race/ethnicity, smoking | OR=4.3, 95% CI [0.7, 27.7] for asthma comparing high formaldehyde exposure group (>=0.08 mg/m3) to low exposure (<0.08 mg/m3). OR=0.4, 95% CI [0, 5.4] for wheezing, shortness of breath or chest tightness related to work comparing high formaldehyde exposure group to low exposure. OR=1, 95% CI [0.1, 15.3] for asthma medication use comparing high formaldehyde exposure group to low exposure. OR=1.6, 95% CI [0.2, 13.2] for wheezing without a cold comparing high formaldehyde exposure group to low exposure. OR=3.5, 95% CI [0.6, 19.1] for shortness of breath with wheezing comparing high formaldehyde exposure group to low exposure. OR=9.5, 95% CI [1.2, 74.7] for woken by shortness of breath comparing high formaldehyde exposure group to low exposure. |
| Frisk et al. 2002 (Prospective Cohort) | Adult asthmatics in the general population planning to live in their homes for the following 18 months to undergo intervention in Orebro, Sweden | 21 asthmatic adults (ages 28-59) | Formaldehyde measured in homes before and after interventions, during heating season (October-April) | Mean (pre-test): 21 ug/m3 (SD 9); mean (post-test): 19 ug/m3 (SD 5) | Questionnaire completed by subjects | Asthma medication, symptom score, pulmonary function tests | No confounders or adjustment factors reported | Mean of medicine consumption pre-test=9, 95% CI [7.18, 10.82], post-test=8.7, 95% CI [6.74, 10.66], p-value reported not statistically significant. Mean of symptom score pre-test=8.6, 95% CI [6.87, 10.33], post-test=8.8, 95% CI [7.12, 10.48], p-value reported not statistically significant. Mean of VC pre-test=90, 95% CI [81.47, 98.53], post-test=88, 95% CI [78.59, 97.41], p-value reported not statistically significant. Mean of FEV1 pre-test=88, 95% CI [77.7, 98.3], post-test=92, 95% CI [81.4, 102.6], p-value reported not statistically significant. Mean of PD20 (histamine) pre-test=557, 95% CI [265.16, 848.84], post-test=717, 95% CI [417.63, 1016.37], p-value reported not statistically significant. Mean of PEF morning value pre-test=458, 95% CI [407.77, 508.23], post-test=470, 95% CI [417.29, 522.71], p-value reported not statistically significant. Mean of PEF morning evening value pre-test=484, 95% CI [438.75, 529.25], post-test=484, 95% CI [430.79, 537.21], p-value reported not statistically significant. Mean of reversibility % of baseline pre-test=7.6, 95% CI [3.71, 11.49], post-test=2.3, 95% CI [-1.42, 6.02], p-value=0.007. |
| Hanson et al. 1993 (Cross-sectional) | Adult workers surveyed in an occupational setting at hospital in New York | 88 workers exposed to operating room exhaust and 84 non-exposed workers (mean age 35.4 years) | Formaldehyde measured for 8 hours in the operating room | Peak level (operating room): 0.99 ppm; time weighted average: 0.02 ppm | Questionnaire completed by subjects including questions adapted from American Thoracic Society Respiratory Disease Questionnaire | Wheezing (over and under age 35), wheezing (RNs and non-RNs), wheezing (smokers and non-smokers), asthma (temporarily associated and work aggravated), wheezing with dyspnea, wheezing | Analyses were stratified by smoking status, age, gender, job description, and Registered Nurse status | Wheezing prevalence=40% for 4th floor exposed compared to 25% for 4th floor non-exposed, p-value reported not significant. Wheezing prevalence=43% for operating room exposed compared to 24% for operating room non-exposed, p-value<0.05. Wheezing with dyspnea prevalence=26% for 4th floor exposed compared to 25% for 4th floor non-exposed, p-value reported not significant. Wheezing with dyspnea prevalence=31% for operating room exposed compared to 21% for operating room non-exposed, p-value reported not significant. Ever asthma work aggravated prevalence=44% for 4th floor exposed compared to 36% for 4th floor non-exposed, p-value=1.00. Ever asthma work aggravated prevalence=55% for operating room exposed compared to 22% for operating room non-exposed, p-value=0.2. Overall wheezing prevalence risk ratio=1.6 for 4th floor comparing exposed to non-exposed, p-value reported not significant. Overall wheezing prevalence risk ratio=1.8 for operating room comparing exposed to non-exposed, p-value<0.05. Wheezing prevalence risk ratio=1.2 for the 4th floor smokers comparing exposed to non-exposed, p-value reported not significant. Wheezing prevalence risk ratio=1.2 for the operating room non-smokers comparing exposed to non-exposed, p-value reported not significant. Wheezing prevalence risk ratio=1.8 for the 4th floor never smokers comparing exposed to non-exposed, p-value reported not significant. Wheezing prevalence risk ratio=2 for the operating room never smokers comparing exposed to non-exposed, p-value<0.05. Wheezing prevalence risk ratio=2.1 for the 4th floor for RNs comparing exposed to non-exposed, p-value<0.05. Wheezing prevalence risk ratio=1.1 for the operating room for RNs comparing exposed to non-exposed, p-value reported not significant. Wheezing prevalence risk ratio=1 for the 4th floor for non-RNs comparing exposed to non-exposed, p-value reported not significant. Wheezing prevalence risk ratio=3.6 for the operating room for non-RNs comparing exposed to non-exposed, p-value<0.05. Wheezing prevalence risk ratio=1.1 for the 4th floor for those over 35 years old comparing exposed to non-exposed, p-value reported not significant. Wheezing prevalence risk ratio=1.3 for the operating room for those over 35 years old comparing exposed to non-exposed, p-value reported not significant. Wheezing prevalence risk ratio=2.4 for the 4th floor for those under 35 years old comparing exposed to non-exposed, p-value reported not significant. Wheezing prevalence risk ratio=2.2 for the operating room for those under 35 years old comparing exposed to non-exposed, p-value reported not significant. |
| Hendrick et al. 1977 (Prospective Cohort) | Adult workers in an occupational setting in a hemodialysis unit and continuously exposed to formalin at Churchill Hospital in Oxford, United Kingdom | 28 staff members (mean age 45 years) | Formaldehyde exposure classified based on job; workers exposed to varying degrees of formalin in 1976 | Exposed while sterilizing equipment with 34-38% solution of formalin in water w/w | Medical examination and medical history | Wheezing, pulmonary function tests | Age and smoking were described, but not accounted for in analyses | Results are descriptive only, with no analyses of data. Inhalation provocation tests with formalin were used to evaluate 4 staff members and a sister of one patient, all of whom had histories of recurrent attacks of wheezing since becoming exposed regularly to formalin. Author suggested that, while exposure to formalin did not seem to be directly responsible in all cases, it might have increased susceptibility to other provoking agents or induced a hyper-reactive responsiveness of the airways. All the staff were interviewed and underwent simple tests of airways function, namely peak expiratory flow (PEF) using a Wright's meter, and one second forced expiratory volume (FEV1), and forced vital capacity (FVC) using a Vitalograph dry spirometer. The results were compared with predicted values for normal subjects from Bates et al. (1971) and from nomograms supplied by Vitalograph Limited. All staff members were asymptomatic when these tests were carried out, and all readings of FEV1, FVC, and PEF were close to predicted normal values. In all cases the ratio FEV1/FVC exceeded 70%. |
| Herbert et al. 1994 (Cross-sectional) | Adult workers in an occupational setting at an oriented strand board plant exposed to formaldehyde and workers in oilfield and gas plant from same area in United States | 99 exposed workers (mean 5.1 years of employment) and 165 non-exposed controls | Formaldehyde measured from five work sites in factory for 21 continuous hours on two separate days in March and April 2012 | Range: 0.07-0.27 ppm | Subjects completed questionnaire based on International Union Against Tuberculosis and Lung Disease questionnaire | Attacks of wheeze, wheeze with chest tightness, chest tightness, attacks of chest tightness, wheeze occasionally (apart from colds), woken by shortness of breath, shortness of breath with exercise, shortness of breath at rest, shortness of breath, asthma, pulmonary function tests | Smoking, age | OR=5.48, 95% CI [1.85, 16.2] for asthma comparing exposed workers versus non-exposed workers. OR=3.34, 95% CI [1.66, 6.73] for attacks of wheeze comparing exposed workers versus non-exposed workers. OR=2.46, 95% CI [1.22, 4.94] for attacks of chest tightness comparing exposed workers versus non-exposed workers. OR=2.71, 95% CI [1.56, 4.69] for chest rightness comparing exposed workers versus non-exposed workers. OR=5.72, 95% CI [2.78, 11.8] for wheeze with chest tightness comparing exposed workers versus non-exposed workers. OR=2.85, 95% CI [1.63, 4.99] for wheeze occasionally (apart from colds) comparing exposed workers versus non-exposed workers. OR=6.78, 95% CI [1.4, 32.7] for woken by shortness of breath comparing exposed workers versus non-exposed workers. OR=5.44, 95% CI [2.91, 10.2] for shortness of breath comparing exposed workers versus non-exposed workers. OR=4.94, 95% CI [2.52, 9.68] for shortness of breath with exercise comparing exposed workers versus non-exposed workers. OR=3.16, 95% CI [1.37, 7.28] for shortness of breath at rest comparing exposed workers versus non-exposed workers. OR=1.68, 95% CI [0.54, 5.25] for FEV1/FVC<75% comparing exposed workers versus non-exposed workers. OR=1.08, 95% CI [0.32, 3.64] for FEV1/FVC<75% ex-smokers comparing exposed workers versus non-exposed workers. OR=2.98, 95% CI [1.1, 8.07] for FEV1/FVC<75% for current smokers comparing exposed workers versus non-exposed workers. |
| Horvath et al. 1988 (Cross-sectional) | Adult workers exposed to formaldehyde in an occupational setting from particle-board or molded products at Weyerhaeuser Co in Marshfield, Wisconsin and control workers from food-processing facilities in nearby areas | 109 exposed workers and 254 unexposed control workers | Formaldehyde measured using personal monitors (8 hour sample) and area monitors | Range (exposed subjects): 0.04-2.93 ppm; range (control subjects): 0.03-0.12 ppm | Subjects completed modified American Thoracic Society respiratory symptom questionnaire before and after work shift | Wheezing, shortness of breath, difficulty breathing, chest pains/aching/tightness/burning, pulmonary function tests | Evaluated impact of height, age, sex, smoking, mobile home residence, and duration of exposure, but analyses did not adjust for these variables | Wheezing prevalence number (percent)=4 (3.7%) for exposed workers compared to 7 (2.8%) for non-exposed workers, p-value reported not significant. Difficulty in breathing prevalence number (percent)=7 (6.4%) for exposed workers compared to 5 (2.0%) for non-exposed workers, p-value reported not significant. Shortness of breath prevalence number (percent)=9 (8.3%) for exposed workers compared to 13 (5.1%) for non-exposed workers, p-value reported not significant. FEV1=3.62L before shift, 3.58L after shift for exposed workers, p-value<0.05. FEV1=3.59L before shift, 3.55L after shift for non-exposed workers, p-value<0.001. FVC=4.49L before shift, 4.49L after shift for exposed workers, p-value reported not significant. FVC=4.47L before shift, 4.41L after shift for non-exposed workers, p-value<0.001. FEV1/FVC%=80.3 before shift, 79.4 after shift for exposed workers, p-value<0.01. FEV1/FVC%=80.5before shift, 80.8 after shift for non-exposed workers, p-value reported not significant. PEFR=8.02L/s before shift, 8.25L/s after shift for exposed workers, p-value reported not significant. PEFR=8.03L/s before shift, 8.06L/s after shift for non-exposed workers, p-value reported not significant. FEF25%-75%=3.71L/s before shift, 3.53L/s after shift for exposed workers, p-value<0.01. FEF25%-75%=3.68L/s before shift, 3.69L/s after shift for non-exposed workers, p-value reported not significant. FEF25%=6.91L/s before shift, 7.02L/s after shift for exposed workers, p-value reported not significant. FEF25%=6.73L/s before shift, 6.73L/s after shift for non-exposed workers, p-value reported not significant. FEF50%=4.50L/s before shift, 4.34L/s after shift for exposed workers, p-value<0.01. FEF50%=4.38L/s before shift, 4.43L/s after shift for non-exposed workers, p-value reported not significant. FEF75%=1.63L/s before shift, 1.52L/s after shift for exposed workers, p-value<0.01. FEF75%=1.66L/s before shift, 1.66L/s after shift for non-exposed workers, p-value reported not significant. |
| Jacobsen et al. 2009 (Prospective Cohort) | Occupational cohort of adult workers exposed in woodworking factories and unexposed workers from control factories in Viborg, Denmark | 1377 woodworkers and 297 control workers (male mean age 38.4 years, female mean age 37.8 years) | Formaldehyde measured using personal monitors in 24 samples from 10 factories in 2003-20005 | Median: 0.05 mg/m3 (range 0.03-0.2) | Questionnaire completed by subjects included questions from ECRHS; ever asthma defined as current or ever self-reported asthma; asthma symptoms defined as yes to at least one group A question (doctor-diagnosed asthma, ever had asthma, current asthma, wheeze) and 2 or more group B questions (chest tightness; wake with chest tightness; wake with wheezing; cough in morning; wheeze in cold air; wheeze with exercise; wheeze from pollen; wheeze from animals; asthma medication) | Current asthma, ever asthma, asthma symptoms, ever wheezing | Age, baseline hay fever, smoking | OR=1.5, 95% CI [0.34, 6.51] for current asthma comparing male woodworkers to male reference workers. OR=6.89, 95% CI [0.85, 55.8] for current asthma comparing female woodworkers to female reference workers. OR=1.9, 95% CI [0.44, 9.12] for ever asthma comparing male woodworkers to male reference workers. OR=3.37, 95% CI [0.91, 12.5] for ever asthma comparing female woodworkers to female reference workers. OR=0.73, 95% CI [0.4, 1.33] for ever wheeze comparing male woodworkers to male reference workers. OR=1.58, 95% CI [0.73, 3.42] for ever wheeze comparing female woodworkers to female reference workers. OR=0.75, 95% CI [0.39, 1.45] for respiratory symptoms comparing male woodworkers to male reference workers. OR=1.31, 95% CI [0.6, 2.83] for respiratory symptoms comparing female woodworkers to female reference workers. |
| Kilburn et al. 1985 (Prospective Cohort) | Occupational cohort of adult male fiberglass batt makers, histology technicians and hospital workers in California | 20 exposed batt makers, 20 unexposed batt makers, 18 histology technicians, 26 unexposed hospital workers (ages 20-62 years) | Formaldehyde measured for histology workers, and batt makers were assumed to have higher levels; self-administered questionnaire asked about exposures to formaldehyde and competing or confounding exposures | Range (histology): 0.4-1.9 ppm; assumed higher exposure levels for batt makers | Questionnaire completed by subjects included questions on respiratory disease history and symptoms | Asthma, breathlessness, wheezing, chest tightness and pain/burning, shortness of breath at work, shortness of breath at rest, respiratory mean score, pulmonary function tests | No confounders or adjustment factors reported | Incidence asthma for hot batt makers=5% (n=20), cold batt makers=15% (n=25), histology=6% (n=18), comparison group=9% (n=26). Incidence breathlessness for hot batt makers=35%, cold batt makers=44%, histology=6%, comparison group=12%. Incidence wheezing for hot batt makers=50%, cold batt makers=36%, histology=6%, comparison group=12%. Incidence chest tightness for hot batt makers=50%, cold batt makers=40%, histology=11%, comparison group=0%. Incidence shortness of breath at rest for hot batt makers=30%, cold batt makers=24%, histology=6%, comparison group=0%. Incidence shortness of breath at work for hot batt makers=40%, cold batt makers=40%, histology=11%, comparison group=4%. Mean respiratory mean score for hot batt makers=5.8, cold batt makers=4.9, histology=1.6, comparison group=0.8. Percentage of workers who decreased FVC by 5% or more of their before-shift values for hot batt makers (noncigarette smokers)=22.2%, bat makers (cigarette smokers)=8.6%, p-value<0.01. Percentage of workers who decreased FEV1 by 10% or more of their before-shift values for hot batt makers (noncigarette smokers)=33.3%, bat makers (cigarette smokers)=11.4%, p-value<0.01. Percentage of workers who decreased FEF25-75 by 15% or more of their before-shift values for hot batt makers (noncigarette smokers)=33.3%, bat makers (cigarette smokers)=11.4%, p-value<0.01. Percentage of workers who decreased FEF75-85 by 15% or more of their before-shift values for hot batt makers (noncigarette smokers)=22.2%, bat makers (cigarette smokers)=40%, p-value<0.01. |
| Kilburn, Seidman, and Warshaw 1985 (Cross-sectional) | Adult women exposed to formaldehyde working in an occupational setting as histology technicians in 23 hospitals and 2 laboratories and unexposed women working as secretaries and clerks at the same institutions in Los Angeles, California | 76 exposed female histology technicians and 56 unexposed female controls (mean age 40, 39.3, 39.5, and 41.5 for clerical workers 0 hr, 1-3 hour, and >4 hours, respectively) | Formaldehyde measured for 1-4 hours in 10 of 25 laboratories | Range in tissue specimen prep and sampling areas: 0.2-1.9 ppm | Questionnaire completed by subjects | Shortness of breath at work, shortness of breath at rest, chest tightness, chest pain | Matched pairs with respect to age, cigarette smoking, and ethnicity. Exposed (technicians) and unexposed (secretaries and clerks) participants had different job functions, but worked for the same organization were of similar SES status. | Prevalence=5% for chest tightness for clerical workers, 7% after 0hr exposure, 27% after 1-3hr exposure, 40% after >4hr exposure, p-value not reported. Prevalence=5% for chest pain for clerical workers, 14% after 0hr exposure, 23% after 1-3hr exposure, 40% after >4hr exposure, p-value not reported. Prevalence=0% for shortness of breath at rest for clerical workers, 0% after 0hr exposure, 4.5% after 1-3hr exposure, 21% after >4hr exposure, p-value not reported. Prevalence=0% for shortness of breath at work for clerical workers, 14% after 0hr exposure, 27% after 1-3hr exposure, 38% after >4hr exposure, p-value not reported. |
| Kim et al. 2014 (Non-randomized controlled trial) | Adult patients in the general population diagnosed with asthma at the Medical College of Yonsei University in Seoul, Korea | 17 asthmatics (age 30s to 60s) | Subjects assigned to two groups: households where plants were introduced and continued (n=9) and households where plants were introduced and then withdrawn (n=8) in January-September 2006 and 2007 | Continuation study: decrease from 24.2 to 15.5 ug/m3; withdrawal study: decrease from 29.7 to 13.6 ug/m3 | Subjects completed Quality of Life Questionnaire for Adult Korean Asthmatics which includes questions on respiratory symptoms | Respiratory symptoms, pulmonary function test | Demographic information was presented for participant gender, age, area/size of residents, and year of building completion (SES proxies). In attempt to prevent any confounding effects due to occupation, most participants were housewives. These factors were not accounted for in analyses. | No association found between quality of life questionnaire for adult Korean asthmatics (QLQAKA) respiratory score and formaldehyde exposure (data not shown). Mean PEFR measures first morning=405 L/min, 1st evening=416 L/min, 2nd morning=406 L/min, 2nd evening=428 L/min. First experiment PEFR continuation in morning=13.9 L/min, withdrawal in morning=-24.7 L/min, p-value<0.01. PEFR continuation in evening=20.6 L/min, withdrawal in evening=-30.2, p-value<0.01. Second experiment PEFR continuation in morning=-9.69 L/min, withdrawal in morning=-9.23 L/min, p-value reported not significant. PEFR continuation in evening=-15.23 L/min, withdrawal in evening=-15.23, p-value reported not significant. |
| Liu et al. 1991 (Cross-sectional) | Adults in the general population living in randomly selected mobile homes in California | 1394 summer phase residents (663 mobile homes) and 1096 winter phase residents (523 mobile homes) | Formaldehyde measured using monitors mailed to participants in kitchen and master bedroom for 7 days in February/March 1985 and July/August 1984 | Range: 0.01 (limit of detection)-0.46 ppm; mean (summer): 0.089 ppm; mean (winter): 0.088 ppm | Questionnaire completed by subjects; occurrence of asthma attack reported for 2 weeks prior to monitoring period | Asthma attack | Age, sex, and smoking were considered, but these were not accounted for in analyses. | Percentage reporting asthma attack for the two weeks prior to monitoring period=80%. No statistical significance reported. |
| Lofstedt et al. 2009 (Prospective Cohort) | Adult workers exposed to formaldehyde in an occupational setting and unexposed controls in four foundries producing cores with the Hot Box method in Sweden | 64 exposed workers and 134 controls (mean age 44.2 years) | Formaldehyde measured for full shift (8 hours) and considered to reflect individual exposure (mean exposure 8.9 years) in 2001 | Mean: 8.9 ug/m3 (SD 8.8) |  | Pulmonary function tests | Models were adjusted for smoking and co-pollutants (methyl isocyanate and isocyanic acid). Authors explored additional characteristics, including BMI and time in present job. Time in present job did not differ between the two groups. Authors report equal proportion of females in exposed and referent groups. | Percent FEV1 change over work shift adjusting for methyl isocyanate and smoking=-1.5%, 95% CI [-4, 1], p-value reported not significant. Percent FEV1 change over work shift adjusting for isocyanic acid and smoking=-1.6%, 95% CI [-4, 0.9], p-value reported not significant. Percent FEV1 change for unexposed workers mean=0.1%, 95% CI [-0.55, 0.75], for coremakers exposed mean=-2%, 95% CI [-3.56, -0.44], p-value<0.05, for die casters exposed mean=0.3%, 95% CI [-1.36, 1.96], p-value reported not significant, for other exposed mean=-1.1%, 95% CI [-3.25, 1.05], p-value reported not significant, for all exposed mean=-1.4%, 95% CI [-2.47, -0.33], p-value<0.05. |
| Low et al. 1985 (Cross-sectional) | Adult workers exposed to formaldehyde and unexposed controls in an occupational setting at a foundry in Brisbane, Australia | 46 exposed workers and 17 controls (mean ages range from 25.3-39.1 years based on work area) | Formaldehyde exposure classified based on job | Core shop: not detected; general foundry: 2-4 ppm; shell: <LOD (1 ppm) | Modified standardized questionnaire from Medical Research Council completed by subjects | Asthma or wheeze onset before at foundry and while at foundry, wheeze at work, pulmonary function tests | Authors reported no differences in cigarette smoking between comparison groups, but noted some differences in age | Number (number attributing to specific environmental factor at work) of prevalence of asthma or wheeze onset while at foundry=3 (1), onset before at foundry=1 (0), wheezing while at work 1. FVC measured on Monday morning mean=91.4, 95% CI [85.89, 96.91] for aftercast (not formaldehyde exposed) versus FVC measured on Monday morning mean=84.1, 95% CI [77.88, 90.32] for general foundry workers (formaldehyde exposed), not statistically significant. Change in FEV1 measured on Monday and Friday mean=-8 mL, 95% CI [-92.8, 76.8] for aftercast versus change in FEV1 measured on Monday and Friday mean=4 mL, 95% CI [-62.49, 70.49] for general foundry workers, not statistically significant. Change in FEV1 over the work week mean=-15 mL, 95% CI [-201.55 171.55] for aftercast versus change in FEV1 over the work week mean=-105 mL, 95% CI [-220.82, 10.82] for general foundry workers, not statistically significant. FEV1/FVC% measured on Monday morning mean=80.4, 95% CI [76.58, 84.22] for aftercast versus FEV1/FVC% measured on Monday morning=83.1 mL, 95% CI [78.81, 87.39] for general foundry workers, not statistically significant. FEV1 measured on Monday morning mean=89.5, 95% CI [82.93, 96.07] for aftercast versus FEV1 measured on Monday morning mean=84.6, 95% CI [76.66, 92.54] for general foundry workers, not statistically significant. Change in FEV1 measured at beginning and end of Monday mean=-9 mL, 95% CI [-115, 97] for aftercast versus FEV1 measured at beginning and end of Monday mean=-57, 95% CI [-187.83, 73.83] for general foundry workers, not statistically significant. |
| Malaka et al. 1990 (Cross-sectional) | Male workers exposed to formaldehyde and nonexposed controls at plywood plant in East Java, Indonesia | 55 exposed male workers (mean age 26.6 years) and 50 unexposed male controls (mean age 28.8 years) | Estimate of cumulative formaldehyde exposure calculated from area concentrations and length of service in current job | Mean (exposed group): 6.29 ppm-yr (SD 2.72); range (area concentrations): 0.22-3.48 ppm | Standardized respiratory questionnaire from American Thoracic Society completed by subjects | Asthma, occupational asthma, wheezing, shortness of breath, pulmonary function tests | Age, dust, smoking status, cigarettes per day, weight, height | OR=6.31 for asthma comparing those exposed to formaldehyde versus not, p-value=0. OR=2.84 for occupational asthma comparing those exposed to formaldehyde versus not, p-value=0.02. OR=1.98 for shortness of breath comparing those exposed to formaldehyde versus not, p-value=0.04. OR=1.2 for wheezing comparing those exposed to formaldehyde versus not, p-value=0.36. Mean FEV1=2.78L, 95% CI [2.7, 2.86] comparing those exposed to formaldehyde versus not, p-value=0.001. Mean FEV1/FVC=3% comparing those exposed to formaldehyde versus not. Mean FEF25%-75%=3.44L/s, 95% CI [3.28, 3.6] for those not exposed to formaldehyde, mean FEF25%-75%=3.04L/s, 95% CI [2.88, 3.2] for those exposed to formaldehyde, p-value=0. Multiple regression coefficient=-0.043 for FEF25%-75% for continuous formaldehyde exposure, p-value<0.05. |
| Norback et al. 1995 (Cross-sectional) | Adults in the general population randomly selected for screening questionnaire, identified from population register of Uppsala, Sweden | 88 adults (aged 20-44 years, mean age 32 years) | Formaldehyde measured in bedrooms for two hours in October 1991-April 1992 | Mean with nocturnal attacks of breathlessness (bedroom): 29 ug/m3 (range in house <5-100); mean without nocturnal attacks of breathlessness (bedroom): 17 ug/m3 (range in house <5-60) | Questionnaire completed by subjects | Nocturnal breathlessness, respiratory symptoms (including wheezing or whistling in chest and daytime breathlessness), pulmonary function tests | Sex, prevalence of wall to wall carpets, prevalence of house dust mites, current smoker, age | OR=12.5, 95% CI [2, 77.9] for nocturnal breathlessness per 10-fold increase in formaldehyde exposure. Wheezing or whistling in the chest and daytime attacks of shortness of breath reported to show no statistically significant association with formaldehyde exposure. No associations found between bronchial hyper-responsiveness, variability in PEF, FEV, %, and the indoor concentration of asthma. |
| Norback et al. 2000 (Cross-sectional) | Adult workers in an occupational setting at four geriatric hospitals in Ystad Sweden |  | Formaldehyde measured on two different days in each building, in two 6 hour samples per building per day in January-February 1997 (1-2 weeks after medical investigations completed) | Mean (signs of dampness): 5 ug/m3 (range 2-8); mean (no signs of dampness): 5 ug/m3 (range 3-9) | Medical examination where subjects questioned by doctor on doctor-diagnosed asthma and respiratory symptoms over last year using questions from ECRHS (wheeze, daytime and nighttime shortness of breath); current asthma defined as reporting at least one asthma related symptom | Asthma symptoms | Report information on smoking, SES (measured as "social status"), sex, age, atopy, and other environmental factors, but these were not accounted for in analyses | Mean formaldehyde exposure for subjects with asthma symptoms=3 ug/m3, 95% CI [1.89, 4.11]. Mean formaldehyde exposure for subjects without asthma symptoms=5 ug/m3, 95% CI [4.53, 5.47] |
| Popa et al. 1969 (Prospective Cohort) | Adults with asthma related to occupational exposure to simple chemicals were enlisted during epidemiological survey (n=29) attended the inpatient and outpatient allergy unit in hospital (n=19) in Bucharest, Romania | 48 asthmatic adults followed for six months to two years after first diagnosis | Formaldehyde used in allergologic tests | Exposed to 1:2,500 dilution of formalin for inhalation tests | Medical examination/response to tests used for diagnosis; bronchial asthma diagnosis was supported by spontaneous asthma attack and asthmatic bronchitis diagnosis supported by mild asthma attack; authors note no clear cut borderline between these diagnoses | Respiratory symptoms (bronchial asthma and asthmatic bronchitis to formalin) | No confounders or adjustment factors reported | Bronchial asthma to formalin inhalation tests were positive, but were delayed (2 to 4 to 12 hours)-chronologic delayed reaction. For skin tests all subjects had (immunologic) delayed type reactions, delayed intradermal tests (24 to 48 hours), and positive patch tests. Bronchial asthma and asthmatic bronchitis to formalin inhalation tests were characteristically negative. Skin tests were positive in three subjects. PK reaction was negative. Precipitating antibodies were absent. |
| Pourmabahabadian et al. 2006 (Cross-sectional) | Adult workers in an occupational setting at 7 hospitals of Tehran University of Medical Sciences in Tehran, Iran | 180 exposed workers from pathology labs (n=38), surgery rooms (n=65), and endoscopy (n=21) and 56 unexposed controls working in administrative affairs section | Formaldehyde measured as 8 hour continuous and spot samples in different departments of 7 hospitals in 2002-2003 | Mean 8 hour sample: 0.96 ppm (pathology), 0.13 ppm (endoscopy), 0.25 ppm (surgery) | Questionnaire completed by subjects | Asthma, chest tightness, pulmonary function tests | Report on smoking, sex and age but do not adjust results for covariates | Percentage reporting asthma=7.9% (pathology), 19% (endoscopy), 1.5% (surgery) versus 5.4% (nonexposed). No statistical association reported. Percentage reporting chest tightness=31.5% (pathology), 28.6% (endoscopy), 27.7% (surgery) versus 16.1% (nonexposed). No statistical association reported. FEV1 mean=2.3L, 95% CI [2.12, 2.48] for those exposed to formaldehyde versus mean=2.9L, 95% CI [2.58, 3.22] for those not exposed to formaldehyde, p-value<0.001. FVC mean=3.3L, 95% CI [3.12, 3.48] for those exposed to formaldehyde versus mean=4L, 95% CI [3.68, 4.32] for those not exposed to formaldehyde, p-value<0.001. FEV1/FVC mean=69.7%, 95% CI [66.02, 73.38] for those exposed to formaldehyde versus mean=72.5%, 95% CI [66.02, 78.98] for those not exposed to formaldehyde, p-value non-significant. FEF25-75 mean=2.66L/s, 95% CI [2.39, 2.93] for those exposed to formaldehyde versus mean=3.35L/s, 95% CI [2.92, 3.78] for those not exposed to formaldehyde, p-value<0.006. |
| Sauder et al. 1987 (Non-randomized controlled trial) | Adult volunteers in the general population with clinical history of asthma and documented hyperactive airways in United States | 9 asthmatic adults (served as own controls) | Subjects exposed to clean air for 3 hours followed by 3 ppm formaldehyde one week later | Mean exposure: 2.9 ppm (SD 0.14) | Symptom questionnaire completed by subjects | Chest discomfort or tightness, pulmonary function tests | All volunteers were nonsmokers. Reported characteristics of age and sex, but analyses were not adjusted for these variables. | Mean of symptom questionnaire score=0.22 after 0min of clean air exposure, mean of symptom questionnaire score=0.22 after 0min of 3ppm formaldehyde exposure, p-value reported not significant. Mean of symptom questionnaire score=0.11 after 2min of clean air exposure, mean of symptom questionnaire score=0.33 after 2min of 3ppm formaldehyde exposure, p-value reported not significant. Mean of symptom questionnaire score=0.22 after 15min of clean air exposure, mean of symptom questionnaire score=0.22 after 15min of 3ppm formaldehyde exposure, p-value reported not significant. Mean of symptom questionnaire score=0.11 after30min of clean air exposure, mean of symptom questionnaire score=0.22 after 30min of 3ppm formaldehyde exposure, p-value reported not significant. Mean of symptom questionnaire score=0.22 after 60min of clean air exposure, mean of symptom questionnaire score=0.44 after 60min of 3ppm formaldehyde exposure, p-value reported not significant. Mean of symptom questionnaire score=0.44 after 120min of clean air exposure, mean of symptom questionnaire score=0.44 after 120min of 3ppm formaldehyde exposure, p-value reported not significant. Mean of symptom questionnaire score=0.44 after 180min of clean air exposure, mean of symptom questionnaire score=0.44 after 180min of 3ppm formaldehyde exposure, p-value reported not significant. Mean of FEV1=3.02 after 180min of clean air exposure, mean of FEV1=3.07 after 180min of 3ppm formaldehyde exposure, p-value reported not significant. Mean of FVC=4.11 after 180min of clean air exposure, mean of FVC=4.16 after 180min of 3ppm formaldehyde exposure, p-value reported not significant. Mean of FEF25-75%=2.64 after 180min of clean air exposure, mean of FEF25-75%=2.59 after 180min of 3ppm formaldehyde exposure, p-value reported not significant. Mean of SGaw=0.101 after 180min of clean air exposure, mean of SGaw=0.106 after 180min of 3ppm formaldehyde exposure, p-value reported not significant. |
| Schenker et al. 1982 (Cross-sectional) | Children and adult residents of six homes in the general population with urea formaldehyde foam insulation (UFFI) in the United States | 24 residents from six homes (ages 7-63 years) | Formaldehyde measured in homes 7 to 34 months following installation of UFFI in 1979-1981 | Range: 0.02-0.23 ppm | Subjects completed American Thoracic Society questionnaire and special supplementary questionnaire for subjects with formaldehyde exposure | Wheeze, pulmonary function tests | Authors report measuring smoking, age, and sex variables, but did not report these data and did not adjust analyses with these variables | Prevalence of participants reporting chronic phlegm=5/24 in UFFI houses. Prevalence of participants reporting chronic cough=11/24 in UFFI houses. Prevalence of participants reporting persistent wheeze=6/24 in UFFI houses. Change in FEV1 for participants in UFFI houses mean=-0.121. Change in FEV1/FVC for participants in UFFI houses mean=-3.74. |
| Thetkathuek et al. 2016 (Cross-sectional) | Adult employees exposed to formaldehyde in an occupational setting at a MDF furniture factory in Thailand | 432 volunteers (out of 535 factory workers) | Formaldehyde measured from five work sites in factory for 21 continuous hours on two separate days in March and April 2012 | Formaldehyde exposures were classified as Low (0.66-3.44 ppm), Moderate (3.45-6.89 ppm), or High (>6.89ppm) | Questionnaire based on the American Thoracic Society Respiratory Symptoms Questionnaire, adjusted to fit working conditions in the furniture factory. Questionnaires were completed by the study subjects independently. | Atopic allergic asthma, asthma symptoms (wheeze) | Authors consider variables education, atopic eczema, allergic asthma, allergic rhinitis history, family history, formaldehyde concentrations or MDF dust concentrations, but unclear whether these are the confounder variables in the adjusted analysis reported. | Number (percentage) reporting prevalence of having wheeze during the daytime or nighttime=56 (15%) for low formaldehyde exposure (0.66-3.44 ug/m3), 2 (4.5%) for moderate formaldehyde exposure (3.45-6.89 ug/m3), and 4 (18.2%) for high formaldehyde exposure (>6.89 ug/m3) |
| Uba et al. 1989 (Prospective Cohort) | Adult medical students exposed to formaldehyde in an occupational setting in an anatomy lab at the University of Southern California | 103 students in class of 1988 (81 students completed questionnaires after exposure to formaldehyde in anatomy lab and after control laboratory with no formaldehyde exposure) (ages 21-33 years, mean age 24.3 years) | Formaldehyde measured using personal samplers in breathing zones of students during anatomy laboratory in September 1984-April 1985 | Time weighted average: <1 ppm; peak exposures: <5 ppm; mean (while dissecting): 1.9 ppm (range 0.1-5.0); mean (while observing dissection): 1.2 ppm (range 0.2-2.0) | Questionnaire completed by subjects including questions on acute symptoms | Acute wheezing, acute dyspnea, persistent wheezing, persistent wheezing with dyspnea, acute chest tightness, pulmonary function measures | Authors report on cigarette consumption, sex, age, height, ethnicity, and history of asthma, but analyses were not adjusted for covariates | Crude OR=0.03 for subjects reporting symptoms of persistent wheezing at end of school year compared to subjects reporting symptoms only at the beginning of the year, p-value<0.001. Crude OR for subjects reporting acute wheezing only during formaldehyde exposure compared to only during control laboratory=2/0, authors defined this OR as infinite with a 2-sided p-value determined to be not significant. Crude OR for subjects reporting acute chest tightened only during formaldehyde exposure compared to only during control laboratory=4/0, authors defined this OR as infinite with a 2-sided p-value=0.05. FVC mean changes on test day 1=-0.012, mean changes on test day 2=-0.042, p-value<0.001, mean changes on test day 3=-0.042, p-value<0.001. FEF25-75 mean changes on test day 1=-0.079, mean changes on test day 2=-0.089, p-value reported not significant, mean changes on test day 3=0.003, p-value reported not significant. FEV1 mean changes on test day 1=-0.031, mean changes on test day 2=-0.046, p-value=0.03, mean changes on test day 3=-0.021, p-value=0.01. FEV1/FVC mean changes on test day 1=-0.004, mean changes on test day 2=-0.003, p-value reported not significant, mean changes on test day 3=0.002, p-value reported not significant. |
| Wieslander et al. 1997 (Cross-sectional) | Adults in the general population randomly selected from population register in Uppsala, Sweden | 562 adults (mean age 32 years) | Formaldehyde measured in the bedroom of a random sample of dwellings of 62 participants | Mean (wall/ceiling painted): 16 ug/m3 (yes) and 21 ug/m3 (no); mean (wood painted): 32 ug/m3 (yes) and 17 ug/m3 (no); mean (kitchen painted): 18 ug/m3 (yes) and 20 ug/m3 (no); mean (bedroom painted): 24 ug/m3 (yes) and 19 ug/m3 (no); mean (bathroom painted): 19 ug/m3 (yes) and 20 ug/m3 (no) | International Union Against Tuberculosis and Lung Disease questionnaire completed by subjects; current asthma defined as combination of bronchial hyperresponsiveness and at least one symptom related to asthma in last year; symptoms included wheezing, shortness of breath, nighttime awakening from breathlessness or tightness of chest | Asthma, wheezing, at least one asthma symptom, daytime and nocturnal breathlessness, pulmonary bronchial hyperresponsiveness | Age, gender, smoking | OR=1.56, 95% CI [0.98, 2.48] for increased prevalence of newly painted dwelling for those reporting asthma v. those not. OR=1.13, 95% CI [0.63, 2.01] for increased prevalence of newly painted workplace for those reporting asthma v. those not. OR=2.33, 95% CI [1.22, 4.46] for increased prevalence of wood painted for those reporting asthma v. those not. OR=2.21, 95% CI [1.09, 4.51] for increased prevalence of kitchen painted for those reporting asthma v. those not. OR=1.21, 95% CI [0.83, 1.76] for increased prevalence of newly painted dwelling for those reporting wheezing v. those not. OR=1.6, 95% CI [1.02, 2.52] for increased prevalence of newly painted workplace for those reporting wheezing v. those not. OR=1.6, 95% CI [0.92, 2.78] for increased prevalence of wood painted for those reporting wheezing v. those not. OR=1.7, 95% CI [0.92, 3.16] for increased prevalence of kitchen painted for those reporting wheezing v. those not. OR=1.16, 95% CI [0.75, 1.79] for increased prevalence of newly painted dwelling for those reporting daytime breathlessness v. those not. OR=1.6, 95% CI [0.96, 2.67] for increased prevalence of newly painted workplace for those reporting daytime breathlessness v. those not. OR=1.94, 95% CI [1.07, 3.5] for increased prevalence of wood painted for those reporting daytime breathlessness v. those not. OR=1.66, 95% CI [0.84, 3.3] for increased prevalence of kitchen painted for those reporting daytime breathlessness v. those not. OR=1.57, 95% CI [1.05, 2.36] for increased prevalence of newly painted dwelling for those reporting nocturnal breathlessness v. those not. OR=1.35, 95% CI [0.82, 2.22] for increased prevalence of newly painted workplace for those reporting nocturnal breathlessness v. those not. OR=1.75, 95% CI [0.98, 3.14] for increased prevalence of wood painted for those reporting nocturnal breathlessness v. those not. OR=2.67, 95% CI [1.42, 5.04] for increased prevalence of kitchen painted for those reporting nocturnal breathlessness v. those not. OR=1.43, 95% CI [1.01, 2.06] for increased prevalence of newly painted dwelling for those reporting at least one asthma symptom v. those not. OR=1.63, 95% CI [1.05, 2.54] for increased prevalence of newly painted workplace for those reporting at least one asthma symptom v. those not. OR=1.8, 95% CI [1.04, 3.12] for increased prevalence of wood painted for those reporting at least one asthma symptom v. those not. OR=2.24, 95% CI [1.2, 4.21] for increased prevalence of kitchen painted for those reporting at least one asthma symptom v. those not. OR=1.37, 95% CI [0.88, 2.13] for increased prevalence of newly painted dwelling for those reporting bronchial hyperresponsiveness v. those not. OR=1.25, 95% CI [0.73, 2.14] for increased prevalence of newly painted workplace for those reporting bronchial hyperresponsiveness v. those not. OR=2, 95% CI [1.06, 3.76] for increased prevalence of wood painted for those reporting bronchial hyperresponsiveness v. those not. OR=2.14, 95% CI [1.08, 4.23] for increased prevalence of kitchen painted for those reporting bronchial hyperresponsiveness v. those not. |
| Yeatts et al. 2012 (Cross-sectional) | Children and adults in the general population recruited based on two-stage cluster sample design in the United Arab Emirates (UAE) | 1590 individuals within four age/sex categories: adult male (ages 19-50 years), adult female (ages 19-50 years), adolescent (ages 11-18 years), and child (ages 6-10 years) | Formaldehyde measured in the common living room for a 7 day period in 2009-2010 | Median: <7.37 ug/m3 (limit of quantification: 7.37; range: 7.37-168.2) | Subjects were interviewed and asked about ever having doctor-diagnosed asthma; respiratory symptoms assessed using ISAAC and Behavioral Risk Factor Surveillance System questions | Ever asthma, wheezing limited speech to 1 or 2 words between breaths, wheezing in last 4 months, wheezing in last 12 months, ever having wheezing and whistling in chest, shortness of breath one or more times a month, shortness of breath in last 12 months, chest tightness/difficulty breathing in last 12 months, chest tightness/difficulty breathing one or more times a month | Urban/rural area, household tobacco smoke exposure, gender, age group | OR=1.43, 95% CI [0.83, 2.46] for chest tightness/difficulty in breathing in last 12 months comparing high formaldehyde exposure group (7.37-168.2ppm) to low formaldehyde exposure group (<7.37ppm). OR=1.55, 95% CI [0.97, 2.48] for shortness of breath in last 12 months comparing high to low formaldehyde exposure group. OR=1.32, 95% CI [0.73, 2.37] for ever asthma comparing high to low formaldehyde exposure group. OR=1.31, 95% CI [0.71, 2.42] for ever having wheezing or whistling in chest comparing high to low formaldehyde exposure group. OR=0.64, 95% CI [0.21, 1.98] for wheezing in past 12 months comparing high to low formaldehyde exposure group. OR=3.48, 95% CI [0.81, 14.89] for wheezing in past 4 weeks comparing high to low formaldehyde exposure group. OR=4.18, 95% CI [1.23, 14.22] for wheezing limiting speech to 1 or 2 words between breaths comparing high to low formaldehyde exposure group. OR=3.68, 95% CI [1.11, 12.27] for shortness of breath one or more times a month comparing high to low formaldehyde exposure group. OR=6.52, 95% CI [1.91, 22.31] for chest tightness/difficulty in breathing one or more times a month comparing high to low formaldehyde exposure group. |
| Zhai et al. 2013 (Cross-sectional) | Households of children and adults in the general population decorated within the previous four years in main urban area of Shenyang, China | One adult per household and 82 children in 186 residential houses | Formaldehyde measured in bedrooms, living rooms, and kitchens over 1 month to 3 years | Polluted homes: 0.093 mg/m3 (bedroom); 0.103 mg/m3 (living room); 0.131 mg/m3 (kitchen); non-polluted homes: 0.43 mg/m3 (bedroom); 0.040 mg/m3 (living room); 0.047 mg/m3 (kitchen) | Subjects (including children with parental assistance) completed survey designed by the American Thoracic Society on respiratory health | Asthma, wheeze, respiratory symptoms for adults and children | Age, education, family history of allergy, gender, height, house facing, indoor domestic pets, occupation, smoking in the family, ventilation frequency, weight | Adult asthma prevalence = 0% for non-polluted homes, 1.68% for polluted homes, p-value reported not significant. Child asthma prevalence = 3.22% for non-polluted homes, 40% for polluted homes, p-value<0.05. Adult wheeze prevalence = 2.99% for non-polluted homes, 5.04% for polluted homes, p-value reported not significant. Child wheeze prevalence = 6.56% for non-polluted homes, 10% for polluted homes, p-value reported not significant. Adult OR=2.603, 95% CI [1.77, 3.828] for respiratory symptoms. Child OR=4.250, 95% CI [2.064, 8.753] for respiratory symptoms. |
