# Supplementary material for: Exposure to formaldehyde and asthma outcomes: A systematic review, meta-analysis, and economic assessment
Source: PLoS One. 2021 Mar 31;16(3):e0248258. doi: 10.1371/journal.pone.0248258 (PMC8011796; doi:10.1371/journal.pone.0248258)
Supplement: S1 Methods — (DOCX) [file pone.0248258.s115.docx]

Supplemental Methods 1. Exclusion Criteria for screening references

Exclusion criteria (title & abstract screening)

1. Article is a review of formaldehyde exposure and asthma;
2. Article contains no original data (e.g., editorial, review paper not relevant to study question, etc.)
3. Article did not involve human subjects (i.e., animal evidence or cell lines only, case report of single human, etc.);
4. Article did not report formaldehyde exposure, as defined by PECO statement;
5. Article did not report outcomes of asthma, as defined by PECO statement;
6. Other reason (explanation required).

Exclusion criteria (full text screening)

1. Article is a review of formaldehyde exposure and asthma;
2. Article contains no original data (e.g., editorial, review paper not relevant to study question, etc.);
3. Article did not involve human subjects (i.e., animal evidence or cell line only, case report of single human, etc.);
4. Article does not report exposure to formaldehyde, as defined by the PECO statement;
5. Article does not report diagnosis of asthma, asthma signs or symptoms, asthma exacerbation, or indirect measures of asthma, as defined by the PECO statement;
6. There was no comparator group;
7. Duplicate study;
8. Other reasons (explanation required).
9. POSSIBLY Include— other language (specify which language below in comments);
