# Supplementary material for: Exposure to formaldehyde and asthma outcomes: A systematic review, meta-analysis, and economic assessment
Source: PLoS One. 2021 Mar 31;16(3):e0248258. doi: 10.1371/journal.pone.0248258 (PMC8011796; doi:10.1371/journal.pone.0248258)
Supplement: S2 Methods — (DOCX) [file pone.0248258.s116.docx]

Supplemental Methods 2. Data Extraction fields

The source criteria checklists for extraction terms include: gold standard publication checklist (GSPC); ARRIVE guidelines (ARRIVE); Cochrane Handbook for Systematic Reviews of Interventions data collection checklist (Cochrane); GRADE criteria for randomized control trials (GRADE).

Data Collection for Human studies

*Fields are free-form except where choices (in italics) are shown*

SOURCE

Refid:

Reviewer:

Publication year:

Authors’ declared conflicts of interest:

- *None declared*
- *Declared*

If declared, provide details:

Study funding source:

- *Government grant*
- *Industry funded*
- *Nonprofit organization grant*
- *Other*

Study funding source details:

What are the study objectives?:

Site(s) of data collection (city, state, country):

METHODS

Study duration/dates:

Study design:

- *Cross-sectional*
- *Cohort, prospective*
- *Cohort, retrospective*
- *Case-control*
- *Ecological*
- *Other (list details below)*

Study design details:

STUDY POPULATION CHARACTERISTICS

Cohort (give description, e.g. NHANES 2004-2006)

Sample size of total cohort

Total number of study groups

Description of reference group

Sample size (each study group)

Target sample size

Participation/follow-up rates

Inclusion/exclusion criteria/recruitment strategy

Age (each exposure group)

Co-morbidities

Other relevant details (list below)

Exposure measurement timing:

- *Maternal/paternal exposure prior to conception*
- *In utero*
- *Prenatal period*
- *Infancy period (up to 24 months)*
- *Childhood period (24 months and after)*
- *Other (details below)*

Exposure measurement timing details:

Source of exposure data:

- *Biomonitoring (list specific matrix)*
- *Environmental monitoring (list specific matrix)*
- *Questionnaire (list specific determinant of exposure)*
- *Other (specify)*

Range of concentrations of formaldehyde measured, and units:

Frequency of exposure measurements if more than once:

Number of replicate measurements taken:

Other chemical information:

Outcomes measured:

Method of asthma outcome measurement/assessment:

Sex (where outcome measured):

- *Males only*
- *Females only*
- *Males and females*
- *Other (details below)*

Number subjects analyzed (for exposure and outcome):

Number of missing participants:

RESULTS

Statistical methods:

- Statistical tests employed
- Statistic (odds ratio, adjusted odds ratio, beta estimate, etc.)
- p-values given
- Confidence intervals given
- Confounding adjustments in statistical tests

Were known confounders accounted for by study design?

Were known confounders accounted for by analysis?

How were data reported (mean, median, raw data, etc.)?:

Asthma measurement/assessment data for each group (i.e., outcome), if available:

How asthma measurement/assessment data were reported (table, figure, etc.), if available:

Summary data for each group

Estimate of effect with confidence interval and p-value

How was precision reported (standard error, CI, etc.)?:

- *Standard error*
- *Standard deviation*
- *Confidence intervals*
- *Other (details below)*
- *Not stated*

How precision reported details:

Precision estimates:

How precision estimates were reported (table, figure, etc):

Miscellaneous comments by reviewer regarding data analysis:
