# Supplementary material for: Exposure to formaldehyde and asthma outcomes: A systematic review, meta-analysis, and economic assessment
Source: PLoS One. 2021 Mar 31;16(3):e0248258. doi: 10.1371/journal.pone.0248258 (PMC8011796; doi:10.1371/journal.pone.0248258)
Supplement: S3 Methods — (DOCX) [file pone.0248258.s117.docx]

Supplemental Methods 3. Risk of Bias instructions

**Human Studies**

*Note: These criteria for judging risk of bias are for human studies only since we are not evaluating animal studies in this case study.*

***Instructions:***

- *Please evaluate each individual study for the following nine risk of bias domains. Please answer “low risk,” “probably low risk,” “probably high risk,” “high risk,” or “not applicable” and provide details/justification for your rating. If there is empirical evidence or other knowledge that informs the direction of bias, please include this in your answer as well; however, if there is not enough information to do so please do not guess at the direction of bias.*
- *Additionally, please note that some internal validity issues could potentially be appropriately captured in several different risk of bias considerations. In this situation, please select the single most appropriate domain to evaluate this potential bias, to avoid double-counting the same internal validity concern.*

**1. Are the study groups at risk of not representing their source populations in a manner that might introduce selection bias?**

The source population is viewed as the population for which study investigators are targeting their study question of interest. Examples of considerations for this risk of bias domain include: 1) level of detail reported for participant inclusion/exclusion (including details from previously published papers referenced in the article for an existing cohort); 2) participation rates and whether this differed by exposure or outcome group; 3) attrition rates and reasons; and 4) comparisons of study characteristics between the study population and full cohort.

Criteria for a judgment of LOW risk of bias (i.e., answer: “No”):

EITHER:

a) The descriptions of the source population, inclusion/exclusion criteria, recruitment and enrollment procedures, participation and follow-up rates were sufficiently detailed, and adequate data were supplied on the distribution of relevant study sample and population characteristics to support the assertion that risk of selection effects was minimal.

OR

b) Although the descriptions and/or data as indicated in “a” above suggested the potential for selection effects, adequate support was given indicating that potential selection effects were *not* differential across both exposure and outcome.

OR

c) Although the descriptions and/or data as indicated in “a” above suggested the potential for selection effects and there was no support indicating that potential selection effects were *not* differential across both exposure and outcome, selection factors appeared to be well-understood, were measured in the data set, and appropriate adjustment post hoc techniques were used to control for selection bias.

Criteria for the judgment of PROBABLY LOW risk of bias (i.e., answer: “Probably No”):

There is insufficient information about participant selection to permit a judgment of low risk of bias, but there is indirect evidence which suggests that inclusion/exclusion criteria, recruitment and enrollment procedures, and participation and follow-up rates were consistent across groups as described by the criteria for a judgment of low risk of bias.

Criteria for the judgment of PROBABLY HIGH risk of bias (i.e., answer: “Probably Yes”):

There is insufficient information about participant selection to permit a judgment of high risk of bias, but there is indirect evidence which suggests that inclusion/exclusion criteria, recruitment and enrollment procedures, and participation and follow-up rates were inconsistent across groups, as described by the criteria for a judgment of high risk of bias.

Criteria for the judgment of HIGH risk of bias (i.e., answer: “Yes”):

1. There were indications from descriptions of the source population, inclusion/exclusion criteria, recruitment and enrollment procedures, participation and follow-up rates, or data on the distribution of relevant study sample and population characteristics that risk of selection effects were substantial; and
2. There was no support to indicate that potential selection effects were *not* differential across both exposure and outcome; and
3. Adjustment post hoc techniques were not used to control for selection bias.

Criteria for the judgment of NOT APPLICABLE (risk of bias domain is not applicable to study):

There is evidence that participant selection is not an element of study design capable of introducing risk of bias in the study.

**2. Was knowledge of the group assignments inadequately prevented (i.e., blinded or masked) during the study, potentially leading to subjective measurement of either exposure or outcome?**

Criteria for a judgment of LOW risk of bias (i.e., answer: “No”):

Any of the following:

- No blinding, but the review authors judge that the outcome measures as well as the exposure measures are not likely to be influenced by lack of blinding (such as differential outcome assessment where the outcome is assessed using different measurement or estimation metrics across the exposure groups, or differential exposure assessment where exposure is assessed using different measurement or estimation metrics across the diagnostic or outcome groups); or
- Blinding of key study personnel was ensured, and it is unlikely that the blinding could have been broken; or
- Some key study personnel were not blinded, but exposure and outcome assessment was blinded and the non-blinding of others is unlikely to introduce bias.

Criteria for the judgment of PROBABLY LOW risk of bias (i.e., answer: “Probably No”):

There is insufficient information about blinding to permit a judgment of low risk of bias, but there is indirect evidence which suggests the study was adequately blinded, as described by the criteria for a judgment of low risk of bias. For example, investigators were effectively blinded to the exposure and/or outcome groups if the exposure was measured by a separate entity and the outcome was obtained from a hospital record.

Criteria for the judgment of PROBABLY HIGH risk of bias (i.e., answer: “Probably Yes”):

There is insufficient information about blinding to permit a judgment of high risk of bias, but there is indirect evidence which suggests the study was not adequately blinded, as described by the criteria for a judgment of high risk of bias.

Criteria for the judgment of HIGH risk of bias (i.e., answer: “Yes”):

Any of the following:

- No blinding or incomplete blinding, and the outcome measures or exposure measures is likely to be influenced by lack of blinding (i.e., differential outcome or exposure assessment); or
- Blinding of key study personnel attempted, but likely that the blinding could have been broken so as to introduce bias; or
- Some key study personnel were not blinded, and the non-blinding of others was likely to introduce bias.

Criteria for the judgment of NOT APPLICABLE (risk of bias domain is not applicable to study):

There is evidence that blinding is not an element of study design capable of introducing risk of bias in the study.

**3. Were exposure assessment methods lacking accuracy?**

*The following list of considerations represents a collection of factors proposed by experts in various fields that may potentially influence the internal validity of the exposure assessment in a systematic manner (not those that may randomly affect overall study results).* ***These should be interpreted only as suggested considerations, and should not be viewed as scoring or a checklist.***

**List of Considerations**:

*Possible exposure assessment metrics:*

1. *Modeling*
2. *Monitoring*
3. *Biomarkers*
4. *Surrogate measure (i.e., parental survey of household exposures)*

*For each, overall considerations include:*

1. *What is the quality of the metric being used?*
2. *Has the metric been validated for the scenario for which it is being used?*
3. *Is the exposure measured in the study a surrogate for formaldehyde (i.e., live in a new home)?*
4. *What was the temporal coverage (i.e., short or long-term exposure, childhood, occupational)?*
5. *Did the analysis account for prediction uncertainty?*
6. *How was missing data accounted for, and any data imputations incorporated?*
7. *Were sensitivity analyses performed?*

*In particular, for exposure assessment models:*

1. *Were the input data in the study suspected to systematically under- or over-estimate exposure?*
2. *What type of model was used (geostatistical interpolation, land-use regression, dispersion models, personal air sampling models, hybrid models, etc.)?*
3. *Were meteorological variables incorporated in the model and justified by authors in their selection?*
4. *Were data on land use, topography, traffic, monitoring data, emission rates, etc. incorporated and justified by authors in their selection?*
5. *What was the spatial variation (e.g., distance from source) and geographic/spatial accuracy (county, census tract, individual residence)?*
6. *What was the temporal specificity and variation (accuracy to level of the day, pregnancy trimester, year, etc.?)*
7. *What was the address completeness (e.g., only home address at one point in time, or more complete address history throughout pregnancy/postnatal life and other locations such as work)?*
8. *What was the space-time coverage of the model?*
9. *Were time-activity patterns accounted for?*
10. *Were concentrations of formaldehyde resulting from a product of secondary formation accounted for in the model?*

Criteria for a judgment of LOW risk of bias (i.e., answer: “No”):

The reviewers judge that there is low risk of exposure misclassification, i.e.:

- There is high confidence in the accuracy of the exposure assessment methods, such as methods that have been tested for validity and reliability in measuring the targeted exposure; or
- Less-established or less direct exposure measurements are validated against well-established or direct methods; or:

1. Biomarkers: a direct measure of formaldehyde exposure during the time period that exposure is considered relevant (i.e., as defined in the PECO statement) was used, and there is sufficient evidence that relevant factors from the List of Considerations above would imply minimal risk of bias in the exposure assessment; or
2. Monitoring: direct and personal monitoring devices or other indoor air monitoring device were used that have been validated for the formaldehyde exposure and scenario for which it was used and there is sufficient evidence that relevant factors from the List of Considerations above would imply minimal risk of bias in the exposure assessment; or
3. Modeling: the model accounted for the time-activity pattern specific to each research participant, (e.g. includes more than exposure at the residential address) and included modeling methods that have been validated or shown to have a high degree of spatial accuracy (e.g. point location), and/or methods that are themselves validated with good agreement compared to person-based air data collection; and there is sufficient evidence that relevant factors from the List of Considerations above would imply minimal risk of bias in the exposure assessment.

AND if applicable (e.g. for laboratory measurements), appropriate QA/QC for methods are described and are satisfactory, with at least three of the following items reported, or at least two of the following items reported plus evidence of satisfactory performance in a high quality inter-laboratory comparison:

- Limit of detection or quantification;
- standards recovery;
- measure of repeatability;
- investigation and prevention of blanks contamination.

Criteria for the judgment of PROBABLY LOW risk of bias (i.e., answer: “Probably No”):

There is insufficient information about the exposure assessment methods to permit a judgment of low risk of bias, but there is indirect evidence that suggests that methods were robust, as described by the criteria for a judgment of low risk of bias. Studies only reporting that the QA/QC items above were satisfactory but not reporting all of the actual numbers may receive a judgment of “probably low risk of bias.” Additionally:

1. Biomarkers: a measure that included formaldehyde exposure during the time period that exposure is considered relevant and has been validated as a direct measure of exposure (i.e., as defined in the PECO statement) was used, or there is some evidence that relevant factors from the List of Considerations above would imply minimal risk of bias in the exposure assessment.
2. Monitoring: methodologies which directly assess exposure were used, such as personal exposure instruments or indoor air monitors, but had not been validated for that purpose, or if such instruments were worn for less than 4 hours per day, or there is some evidence that relevant factors from the List of Considerations above would imply minimal risk of bias in the exposure assessment.
3. Modeling: the model used methods that do not meet the criteria of including time-activity patterns AND spatial accuracy, and so may not have the level of validation compared to person-based air measurement, but include measurements that have evidence of quality, such as good-quality data inputs, validation against area-based air measurement, or other establishments of the accuracy of the data inputs and models, or there is some evidence that relevant factors from the List of Considerations above would imply minimal risk of bias in the exposure assessment.

Criteria for the judgment of PROBABLY HIGH risk of bias (i.e., answer: “Probably Yes”):

There is insufficient information about the exposure assessment methods to permit a judgment of high risk of bias, but there is indirect evidence that suggests that methods were not robust, as described by the criteria for a judgment of high risk of bias. Additionally:

1. Biomarkers: this includes indirect measures of formaldehyde exposure but not specific to this exposure, such as DNA adducts, inflammation or oxidative stress, during the time period that exposure is considered relevant (i.e., developmental period as defined in the PECO statement), or there is some evidence that relevant factors from the List of Considerations above would imply risk of bias in the exposure assessment.
2. Monitoring: measurement of exposures that may not have been validated for use to study formaldehyde exposure were used, or there is some evidence that relevant factors from the List of Considerations above would imply risk of bias in the exposure assessment.
3. Modeling: models were used that have not been compared to person-based or area-based air measurements and have suspicion of problems estimating true exposure because, for example, they do not have spatial accuracy (e.g. county-level measures), do not pertain to the correct time frame, are based on limited data, or differ in methodology between cases and controls in a study, or there is some evidence that relevant factors from the List of Considerations above would imply risk of bias in the exposure assessment.

Criteria for the judgment of HIGH risk of bias (i.e., answer: “Yes”):

The reviewers judge that there is high risk of exposure misclassification and any one of the following:

- There is low confidence in the accuracy of the exposure assessment methods; or
- Less-established or less direct exposure measurements are not validated and are suspected to introduce bias that impacts the outcome assessment (example: participants are asked to report exposure status retrospectively, subject to recall bias); or
- Uncertain how exposure information was obtained; or:

1. Biomarkers: There is sufficient evidence that relevant factors from the List of Considerations above would imply risk of bias in the exposure assessment.
2. Monitoring: Information from databases or otherwise was gathered that indirectly assessed exposure without considering variables noted in the List of Considerations above, such as spatial variability, land use regression, etc., or there is sufficient evidence that relevant factors from the List of Considerations above would imply risk of bias in the exposure assessment.
3. Modeling: the model used has been demonstrated not to pertain to area-based or person-based measures or has otherwise been previously demonstrated to be unable to describe air levels of exposure for assigning exposure in a research situation, or there is sufficient evidence that relevant factors from the List of Considerations above would imply risk of bias in the exposure assessment.

Criteria for the judgment of NOT APPLICABLE (risk of bias domain is not applicable to study):

There is evidence that exposure assessment methods are not capable of introducing risk of bias in the study.

**4. Were outcome assessment methods lacking accuracy?**

Criteria for a judgment of LOW risk of bias (i.e., answer: “No”):

The reviewers judge that there is low risk of outcome misclassification, i.e.:

- Outcomes were assessed and defined consistently across all study participants, using valid and reliable measures (note that all outcome assessment measures captured in the PECO statement (clinical asthma diagnosis by health practitioner, asthma-related hospital admissions or emergency department visits or outpatient visits or ICU admissions/intubations, asthma-related medication use, ASUI retrospective questionnaire, use of systemic corticosteroids for asthma, wheezing, methacholine challenge test, and/or spirometry measure) are considered beforehand to be valid and reliable, unless other information provided within the study warrants a consideration otherwise); or
- Less-established or less direct outcome measurements are validated against well-established or direct methods; or
- Appropriate sensitivity analyses were conducted that suggest the influence of outcome misclassification would be minimal
- AND, if applicable, appropriate QA/QC for methods is described and is satisfactory.

Criteria for the judgment of PROBABLY LOW risk of bias (i.e., answer: “Probably No”):

There is insufficient information about the outcome assessment methods to permit a judgment of low risk of bias, but there is indirect evidence which suggests that methods were robust, as described by the criteria for a judgment of low risk of bias. Appropriate QA/QC for methods are not described but the review authors judge that the outcome and the outcome assessment are objective and uniform across study groups.

Criteria for the judgment of PROBABLY HIGH risk of bias (i.e., answer: “Probably Yes”):

There is insufficient information about the outcome assessment methods to permit a judgment of high risk of bias, but there is indirect evidence which suggests that methods were not robust, as described by the criteria for a judgment of high risk of bias.

Criteria for the judgment of HIGH risk of bias (i.e., answer: “Yes”):

The reviewers judge that there is high risk of outcome misclassification and any one of the following:

- There is low confidence in the accuracy of the outcome assessment methods; or
- Less-established or less direct outcome measurements are not validated and are suspected to introduce bias that impacts the outcome assessment
- Uncertain how outcome information was obtained

Criteria for the judgment of NOT APPLICABLE (risk of bias domain is not applicable to study):

There is evidence that outcome assessment methods are not capable of introducing risk of bias in the study.

**5. Was potential confounding inadequately incorporated?**

List of important potential confounders, collectively generated by review authors prior to the initiation of screening for studies based on expert opinion and knowledge gathered from the literature:

Tier I: Important confounders

Occupational studies – (1) smoking status or exposure to secondhand or environmental tobacco smoke (unless conducted at a time or place known to have banned smoking in the workplace in which case we would only consider active smoking); and (2) socioeconomic status.

Non-occupational studies – (1) age, in studies where participants are 6 years old or less; (2) smoking status or exposure to secondhand or environmental tobacco smoke; and (3) socioeconomic status/parental education in studies of children.

*Age, smoking status including exposure to secondhand or environmental tobacco smoke, and socioeconomic status/parental education are considered important confounders for as there is evidence they are related to both the exposure (formaldehyde) and health outcome (asthma).*

*Age (in studies of children 6 years old or less) is considered an important confounder because it can impact the diagnosis of asthma, particularly at younger age ranges (<6 years) (1). Age may also be associated with the levels of formaldehyde exposures, inhalation rates, time spent indoors at home versus at school, may vary by age (2).*

*Smoking status and exposure to secondhand/environmental tobacco smoke are considered important confounders because cigarette smoke contains formaldehyde (3, 4) and exposure to cigarette smoke is also generally a recognized risk factor for asthma in childhood (5).*

*Socioeconomic status (and parental education as a surrogate measure) is considered an important confounder because SES is related to indoor exposures to formaldehyde (6) and also related to the outcome of asthma (7).*

Tier II: Other potentially important confounders:

Race/ethnicity, sex, height, weight, BMI, obesity status, parental or family history of asthma, allergy, additional environmental (non-formaldehyde) exposures.

*These confounders were selected from evidence in the literature (8-10) that suggested these might be potential confounders, strongly associated with one of either exposure or outcome but not as strongly associated with the other, or that may be potential surrogates for one of the important confounders listed above. To avoid over-adjustment for confounders, these Tier II confounders are characteristics that could potentially be adjusted for, but would not be a primary consideration in evaluating a study’s risk of bias.*

*Season is considered an potential confounder because it can impact asthma symptoms in children, which are more likely to occur in the fall (11). Likewise, formaldehyde exposures both indoor and outdoor may also vary by season (12, 13). Age in studies (involving individuals > 6 years old) was considered a potential confounder it can be associated with different types of outdoor exposures and the level of exposure (since respiratory rates and levels of contact with floor and wood products differ by age group) (14, 15); however, age>6 years is not known to be associated with asthma diagnosis, signs or symptoms and so as long as age is a consideration in assessing pulmonary function tests, it would not be considered a potentially important confounder.*

Criteria for a judgment of LOW risk of bias (i.e., answer: “No”):

The study appropriately assessed and accounted for (i.e., matched, stratified, excluded certain populations (i.e., smokers) or statistically controlled for) all important confounders (Tier I) using appropriate statistical techniques, or reported that important confounders were evaluated and omitted because inclusion did not substantially affect the results. The determination of specific confounders may also be informed by, but not limited to, the studies included in the overall review,

AND the study appropriately assessed and accounted for (i.e., matched, stratified, or statistically controlled for) other potentially important confounders relevant (Tier II) using appropriate statistical techniques, or reported that these confounders were evaluated and omitted because inclusion did not substantially affect the results,

AND the important potential confounders were measured consistently across study groups using valid and reliable methods, or the influence of covariate measurement error was determined, through sensitivity analysis, to be minimal.

Criteria for the judgment of PROBABLY LOW risk of bias (i.e., answer: “Probably No”):

The study appropriately accounted for most but not all of the important confounders (Tier I) or used appropriate statistical techniques;

AND some of the other potentially important confounders relevant (Tier II) using appropriate statistical techniques,

OR reported that these confounders were evaluated and omitted because inclusion did not substantially affect the results;

AND this is not expected to introduce substantial bias.

Criteria for the judgment of PROBABLY HIGH risk of bias (i.e., answer: “Probably Yes”):

The study evaluated some but not all of the important confounders (Tier I),

AND some but not all of the other potentially important confounders relevant (Tier II),

OR used questionable statistical techniques for confounder adjustment;

AND this is expected to introduce substantial bias.

Criteria for the judgment of HIGH risk of bias (i.e., answer: “Yes”):

The study did not account for or evaluate multiple important confounders (Tier I),

AND did not account for or evaluate multiple other potentially important confounders relevant (Tier II),

OR the important potential confounders were inappropriately measured and/or inappropriately analyzed across study groups.

Criteria for the judgment of NOT APPLICABLE (risk of bias domain is not applicable to study):

There is evidence that outcome assessment methods are not capable of introducing risk of bias in the study.

**6. Were incomplete outcome data inadequately addressed?**

Criteria for a judgment of LOW risk of bias (i.e., answer: “No”):

Participants were followed long enough to obtain outcome measurements

OR any one of the following:

- No missing outcome data; or
- Reasons for missing outcome data unlikely to be related to true outcome (for survival data, censoring unlikely to introduce bias); or
- Attrition or missing outcome data balanced in numbers across exposure groups, with similar reasons for missing data across groups; or
- For dichotomous outcome data, the proportion of missing outcomes compared with observed event risk not enough to have a relevant impact on the exposure effect estimate; or
- For continuous outcome data, plausible effect size (difference in means or standardized difference in means) among missing outcomes not enough to have a relevant impact on the observed effect size; or
- Missing data have been imputed using appropriate methods

Criteria for the judgment of PROBABLY LOW risk of bias (i.e., answer: “Probably No”):

There is insufficient information about incomplete outcome data to permit a judgment of low risk of bias, but there is indirect evidence which suggests incomplete outcome data was adequately addressed, as described by the criteria for a judgment of low risk of bias.

Criteria for the judgment of PROBABLY HIGH risk of bias (i.e., answer: “Probably Yes”):

There is insufficient information about incomplete outcome data to permit a judgment of high risk of bias, but there is indirect evidence which suggests incomplete outcome data was not adequately addressed, as described by the criteria for a judgment of high risk of bias.

Criteria for the judgment of HIGH risk of bias (i.e., answer: “Yes”):

Participants were not followed long enough to obtain outcome measurements

OR any one of the following:

- Reason for missing outcome data likely to be related to true outcome, with either imbalance in numbers or reasons for missing data across exposure groups; or
- For dichotomous outcome data, the proportion of missing outcomes compared with observed event risk enough to induce biologically relevant bias in intervention effect estimate; or
- For continuous outcome data, plausible effect size (difference in means or standardized difference in means) among missing outcomes enough to induce biologically relevant bias in observed effect size; or
- Potentially inappropriate application of imputation.

Criteria for the judgment of NOT APPLICABLE (risk of bias domain is not applicable to study):

There is evidence that incomplete outcome data is not capable of introducing risk of bias in the study.

**7.** **Does the study report appear to have selective outcome reporting?**

Criteria for a judgment of LOW risk of bias (i.e., answer: “No”):

All of the study’s pre-specified (primary and secondary) outcomes outlined in the pre-published protocol or the published manuscript’s methods, abstract, and/or introduction section that are of interest in the review have been reported in the pre-specified way.

Criteria for the judgment of PROBABLY LOW risk of bias (i.e., answer: “Probably No”):

There is insufficient information about selective outcome reporting to permit a judgment of low risk of bias, but there is indirect evidence which suggests the study was free of selective reporting, as described by the criteria for a judgment of low risk of bias. This includes if a pre-published protocol is not available but the study’s pre-specified (primary and secondary) outcomes outlined in the published manuscript’s methods, abstract, and/or introduction section that are of interest in the review have been reported in the pre-specified way.

Criteria for the judgment of PROBABLY HIGH risk of bias (i.e., answer: “Probably Yes”):

There is insufficient information about selective outcome reporting to permit a judgment of high risk of bias, but there is indirect evidence which suggests the study was not free of selective reporting, as described by the criteria for a judgment of high risk of bias. This includes if a pre-published protocol is not available and the study’s pre-specified (primary and secondary) outcomes outlined in the published manuscript’s methods, abstract, and/or introduction section that are of interest in the review have not been reported in the pre-specified way.

Criteria for the judgment of HIGH risk of bias (i.e., answer: “Yes”):

Any one of the following:

- Not all of the study’s pre-specified primary outcomes (as outlined in the pre-published protocol or published manuscript’s methods, abstract, and/or introduction) have been reported; or
- One or more primary outcomes is reported using measurements, analysis methods or subsets of the data (e.g. subscales) that were not pre-specified; or
- One or more reported primary outcomes were not pre-specified (unless clear justification for their reporting is provided, such as an unexpected effect); or
- One or more outcomes of interest are reported incompletely

Criteria for the judgment of NOT APPLICABLE (risk of bias domain is not applicable to study):

There is evidence that selective outcome reporting is not capable of introducing risk of bias in the study.

**8. Did the study receive any support from a company, study author, or other entity having a financial interest in any of the exposures studied?**

Criteria for a judgment of LOW risk of bias (i.e., answer: “No”):

The study did not receive support from a company, study author, or other entity having a financial interest in the outcome of the study. Examples include the following:

- Funding source is limited to government, non-profit organizations, or academic grants funded by government, foundations and/or non-profit organizations;
- Chemicals or other treatment used in study were purchased from a supplier;
- Company affiliated staff are not mentioned in the acknowledgements section;
- Authors were not employees of a company with a financial interest in the outcome of the study;
- Company with a financial interest in the outcome of the study was not involved in the design, conduct, analysis, or reporting of the study and authors had complete access to the data;
- Study authors make a claim denying conflicts of interest;
- Study authors are unaffiliated with companies with financial interest, and there is no reason to believe a conflict of interest exists;
- All study authors are affiliated with a government agency (are prohibited from involvement in projects for which there is a conflict of interest or an appearance of conflict of interest).

Criteria for the judgment of PROBABLY LOW risk of bias (i.e., answer: “Probably No”):

There is insufficient information to permit a judgment of low risk of bias, but there is indirect evidence which suggests the study was free of support from a company, study author, or other entity having a financial interest in the outcome of the study, as described by the criteria for a judgment of low risk of bias.

Criteria for the judgment of PROBABLY HIGH risk of bias (i.e., answer: “Probably Yes”):

There is insufficient information to permit a judgment of high risk of bias, but there is indirect evidence which suggests the study was not free of support from a company, study author, or other entity having a financial interest in the outcome of the study, as described by the criteria for a judgment of high risk of bias.

Criteria for the judgment of HIGH risk of bias (i.e., answer: “Yes”):

The study received support from a company, study author, or other entity having a financial interest in the outcome of the study. Examples of support include:

- Research funds;
- Chemicals, equipment or testing provided at no cost;
- Writing services;
- Author/staff from study was employee or otherwise affiliated with company with financial interest;
- Company limited author access to the data;
- Company was involved in the design, conduct, analysis, or reporting of the study;
- Study authors claim a conflict of interest

Criteria for the judgment of NOT APPLICABLE (risk of bias domain is not applicable to study):

There is evidence that conflicts of interest are not capable of introducing risk of bias in the study.

**9. Did the study appear to have other problems that could put it at a risk of bias?**

Criteria for a judgment of LOW risk of bias (i.e., answer: “No”):

The study appears to be free of other sources of bias.

Criteria for the judgment of PROBABLY LOW risk of bias (i.e., answer: “Probably No”):

There is insufficient information to permit a judgment of low risk of bias, but there is indirect evidence which suggests the study was free of other threats to validity.

Criteria for the judgment of PROBABLY HIGH risk of bias (i.e., answer: “Probably Yes”):

There is insufficient information to permit a judgment of high risk of bias, but there is indirect evidence which suggests the study was not free of other threats to validity, as described by the criteria for a judgment of high risk of bias.

Criteria for the judgment of HIGH risk of bias (i.e., answer: “Yes”):

There is at least one important risk of bias. For example, the study:

- Had a potential source of bias related to the specific study design used; or
- Stopped early due to some data-dependent process (including a formal-stopping rule); or
- The conduct of the study is affected by interim results (e.g. recruiting additional participants from a subgroup showing greater or lesser effect); or
- Has been claimed to have been fraudulent; or
- Had some other problem
