# Supplementary material for: Exposure to formaldehyde and asthma outcomes: A systematic review, meta-analysis, and economic assessment
Source: PLoS One. 2021 Mar 31;16(3):e0248258. doi: 10.1371/journal.pone.0248258 (PMC8011796; doi:10.1371/journal.pone.0248258)
Supplement: S1 Results — (DOCX) [file pone.0248258.s119.docx]

Supplemental Results 1. List of Studies in Bins 2-5

**Bin 2 studies:** Asthma status of study participants unknown or not addressed by authors

1. Franklin, P., Dingle, P., & Stick, S. (2000). Raised exhaled nitric oxide in healthy children is associated with domestic formaldehyde levels. *American Journal of Respiratory and Critical Care Medicine*, *161*(5), 1757-1759.
2. Sauder, L. R., Chatham, M. D., Green, D. J., & Kulle, T. J. (1986). Acute pulmonary response to formaldehyde exposure in healthy nonsmokers. *Journal of occupational medicine.: official publication of the Industrial Medical Association*, *28*(6), 420-424.
3. Slaughter, J. C., Koenig, J. Q., & Reinhardt, T. E. (2004). Association between lung function and exposure to smoke among firefighters at prescribed burns. *Journal of occupational and environmental hygiene*, *1*(1), 45-49.
4. IMBUS, H. R., & TOCHILIN, S. J. (1988). Acute effect upon pulmonary function of low level exposure to phenol-formaldehyde-resin-coated wood. *American Industrial Hygiene Association Journal*, *49*(9), 434-437.
5. Holmström, M., Rosén, G., & Wilhelmsson, B. (1991). Symptoms, airway physiology and histology of workers exposed to medium-density fiber board. *Scandinavian journal of work, environment & health*, 409-413.
6. Symington, P., Coggon, D., & Holgate, S. (1991). Respiratory symptoms in children at schools near a foundry. *Occupational and Environmental Medicine*, *48*(9), 588-591.
7. Ulfvarson, U., Alexandersson, R., Aringer, L., Svensson, E., Hedenstierna, G., Hogstedt, C., ... & Sorsa, M. (1987). Effects of exposure to vehicle exhaust on health. *Scandinavian journal of work, environment & health*, 505-512.
8. Wallner, P., Kundi, M., Moshammer, H., Piegler, K., Hohenblum, P., Scharf, S., ... & Hutter, H. P. (2012). Indoor air in schools and lung function of Austrian school children. *Journal of Environmental Monitoring*, *14*(7), 1976-1982.
9. Wallner, P., Kundi, M., Panny, M., Tappler, P., & Hutter, H. P. (2015). Exposure to air ions in indoor environments: Experimental study with healthy adults. *International journal of environmental research and public health*, *12*(11), 14301-14311.
10. Broder, I., Corey, P., Cole, P., Lipa, M., Mintz, S., & Nethercott, J. R. (1988). Comparison of health of occupants and characteristics of houses among control homes and homes insulated with urea formaldehyde foam: II. Initial health and house variables and exposure-response relationships. *Environmental research*, *45*(2), 156-178.
11. Day, J. H., Lees, R. E., Clark, R. H., & Pattee, P. L. (1984). Respiratory response to formaldehyde and off-gas of urea formaldehyde foam insulation. *Canadian Medical Association Journal*, *131*(9), 1061.
12. Grammer, L. C., Harris, K. E., Shaughnessy, M. A., Sparks, P., Ayars, G. H., Altman, L. C., & Patterson, R. (1990). Clinical and immunologic evaluation of 37 workers exposed to gaseous formaldehyde. *Journal of allergy and clinical immunology*, *86*(2), 177-181.
13. Holmström, M., & Wilhelmsson, B. O. (1988). Respiratory symptoms and pathophysiological effects of occupational exposure to formaldehyde and wood dust. *Scandinavian journal of work, environment & health*, 306-311.
14. Holness, D. L., & Nethercott, J. R. (1989). Health status of funeral service workers exposed to formaldehyde. *Archives of Environmental Health: An International Journal*, *44*(4), 222-228.
15. Main, D. M., & Hogan, T. J. (1983). Health effects of low-level exposure to formaldehyde. *Journal of occupational medicine.: official publication of the Industrial Medical Association*, *25*(12), 896-900.
16. Neghab, M., Soltanzadeh, A., & Choobineh, A. (2011). Respiratory morbidity induced by occupational inhalation exposure to formaldehyde. *Industrial health*, *49,* 89-94.
17. Nunn, A. J., Craigen, A. A., Darbyshire, J. H., Venables, K. M., & Taylor, A. N. (1990). Six year follow up of lung function in men occupationally exposed to formaldehyde. *Occupational and Environmental Medicine*, *47*(11), 747-752.

**Bin 3 studies:** Asthmatics explicitly excluded or there happened to be no asthmatics in the study population

1. Alexandersson, R., & Hedenstierna, G. (1988). Respiratory hazards associated with exposure to formaldehyde and solvents in acid-curing paints. *Archives of Environmental Health: An International Journal*, *43*(3), 222-227.
2. Rudell, B., Sandström, T., Hammarström, U., Ledin, M. L., Hörstedt, P., & Stjernberg, N. (1994). Evaluation of an exposure setup for studying effects of diesel exhaust in humans. *International archives of occupational and environmental health*, *66*(2), 77-83.
3. Saowakon, N., Ngernsoungnern, P., Watcharavitoon, P., Ngernsoungnern, A., & Kosanlavit, R. (2015). Formaldehyde exposure in gross anatomy laboratory of Suranaree University of Technology: a comparison of area and personal sampling. *Environmental Science and Pollution Research*, *22*(23), 19002-19012.
4. Schachter, E. N., Witek Jr, T. J., Tosun, T., Leaderer, B. P., & Beck, G. J. (1986). A study of respiratory effects from exposure to 2 ppm formaldehyde in healthy subjects. *Archives of Environmental Health: An International Journal*, *41*(4), 229-239.
5. Schoenberg, J. B., & Mitchell, C. A. (1975). Airway disease caused by phenolic (phenol-formaldehyde) resin exposure. *Archives of Environmental Health: An International Journal*, *30*(12), 574-577.
6. Shrivastava, A., & Saxena, Y. (2013). Effect of formalin vapours on pulmonary functions of medical students in anatomy dissection hall over a period of one year. *Indian J Physiol Pharmacol*, *57*(3), 255-60.
7. Uthiravelu, P., Saravanan, A., Kumar, C. K., & Vaithiyanandane, V. (2015). Pulmonary function test in formalin exposed and nonexposed subjects: A comparative study. *Journal of pharmacy & bioallied sciences*, *7*(Suppl 1), S35.
8. Wieslander, G., Kumlin, A., & Norbäck, D. (2010). Dampness and 2-ethyl-1-hexanol in floor construction of rehabilitation center: health effects in staff. *Archives of environmental & occupational health*, *65*(1), 3-11.
9. Alexandersson, R., Kolmodin-Hedman, B., & Hedenstierna, G. (1982). Exposure to formaldehyde: effects on pulmonary function. *Archives of Environmental Health: An International Journal*, *37*(5), 279-284.
10. Selmer, A. S. A. (2001). Effects of blasting fumes on exposure and short-term lung function changes in tunnel construction workers. *Scand J Work Environ Health*, *27*(4), 250-257.
11. Green, D. J., Bascom, R., Healey, E. M., Hebel, J. R., Sauder, L. R., & Kulle, T. J. (1989). Acute pulmonary response in healthy, nonsmoking adults to inhalation of formaldehyde and carbon. *Journal of Toxicology and Environmental Health, Part A Current Issues*, *28*(3), 261-275.
12. Khaliq, F., & Tripathi, P. (2009). SHORT COMMUNICATION ACUTE EFFECTS OF FORMALIN ON PULMONARY FUNCTIONS IN GROSS ANATOMY LABORATORY. *Indian J Physiol Pharmacol*, *53*(1), 93-96.
13. Kilburn, K. H., Warshaw, R., & Thornton, J. C. (1989). Pulmonary function in histology technicians compared with women from Michigan: effects of chronic low dose formaldehyde on a national sample of women. *Occupational and Environmental Medicine*, *46*(7), 468-472.
14. Kulle, T. J., Sauder, L. R., Hebel, J. R., Green, D. J., & Chatham, M. D. (1987). Formaldehyde dose-response in healthy nonsmokers. *Japca*, *37*(8), 919-924.
15. Lang, I., Bruckner, T., & Triebig, G. (2008). Formaldehyde and chemosensory irritation in humans: a controlled human exposure study. *Regulatory Toxicology and Pharmacology*, *50*(1), 23-36.

**Bin 4 studies:** No measures of association between quantitative measures of formaldehyde and asthma outcomes

1. Ostojić, L., Bradarić, A., Miše, K., Ostojić, Z., Lovrić, J., Petrović, P., ... & Tocilj, J. (2006). Pulmonary function in persons who are professionally exposed to formaldehyde fumes. *Collegium antropologicum*, *30*(3), 507-511.
2. Pechter, E., Davis, L. K., Tumpowsky, C., Flattery, J., Harrison, R., Reinisch, F., ... & Filios, M. (2005). Work‐related asthma among health care workers: Surveillance data from California, Massachusetts, Michigan, and New Jersey, 1993–1997. *American journal of industrial medicine*, *47*(3), 265-275.
3. Piipari, R., & Keskinen, H. (2005). Agents causing occupational asthma in Finland in 1986–2002: Cow epithelium bypassed by moulds from moisture‐damaged buildings. *Clinical & Experimental Allergy*, *35*(12), 1632-1637.
4. Thun, M. J., Lakat, M. F., & Altman, R. (1982). Symptom survey of residents of homes insulated with urea-formaldehyde foam. *Environmental research*, *29*(2), 320-334.
5. Borm, P. J. A., Jetten, M., Hidayat, S., Van de Burgh, N., Leunissen, P., Kant, I., ... & Soeprapto, H. (2002). Respiratory symptoms, lung function, and nasal cellularity in Indonesian wood workers: a dose-response analysis. *Occupational and environmental medicine*, *59*(5), 338-344.
6. Arbak, P., Bilgin, C., Balbay, O., Yesildal, N., Annakkaya, A. N., & Ulger, F. (2004). Respiratory symptoms and peak expiratory flow rates among furniture-decoration students. *Annals of Agricultural and Environmental Medicine*, *11*(1), 13-17.
7. Gamble, J. F., Nicolich, M. J., Barone, N. J., & Vincent, W. J. (1999). Exposure-response of asphalt fumes with changes in pulmonary function and symptoms. *Scandinavian journal of work, environment & health*, 186-206.
8. de Marco, R., Marcon, A., Rava, M., Cazzoletti, L., Pironi, V., Silocchi, C., & Ricci, P. (2010). Proximity to chipboard industries increases the risk of respiratory and irritation symptoms in children: the Viadana study. *Science of the total environment*, *408*(3), 511-517.
9. Glindmeyer, H. W., Rando, R. J., Lefante, J. J., Freyder, L., Brisolara, J. A., & Jones, R. N. (2008). Longitudinal respiratory health study of the wood processing industry. *American journal of industrial medicine*, *51*(8), 595-609.
10. Goris, J. A., Ang, S., & Navarro, C. (1998). Laboratory safety: Minimizing the toxic effects of formaldehyde. *Laboratory Medicine*, *29*(1), 39-43.
11. Grimsley, L. F., Chulada, P. C., Kennedy, S., White, L., Wildfire, J., Cohn, R. D., ... & Sterling, Y. (2012). Indoor environmental exposures for children with asthma enrolled in the HEAL study, post-Katrina New Orleans. *Environmental health perspectives*, *120*(11), 1600-1606.
12. Hagmar, L., Bellander, T., Englander, V., Ranstam, J., Attewell, R., & Skerfving, S. (1986). Mortality and cancer morbidity among workers in a chemical factory. *Scandinavian journal of work, environment & health*, 545-551.
13. Mohammadpour, A. A., & Maleki, M. O. H. S. E. N. (2011). Effect of formaldehyde exposure on pulmonary function tests of veterinary students in anatomy laboratory. *Journal of Applied Animal Research*, *39*(2), 114-116.
14. Norman, G. R., Pengelly, L. D., Kerigan, A. T., & Goldsmith, C. H. (1986). Respiratory function of children in homes insulated with urea formaldehyde foam insulation. *CMAJ: Canadian Medical Association Journal*, *134*(10), 1135.

**Bin 5 studies:** Abstract only or foreign language studies that did not appear to fit into any other bin

1. Del Carpio, J., Ballachey, M. L., Osterland, C. K., Gilmore, N., & Hoey, J. (1985). 155 Failure of aqueous urea-formaldehyde foam insulation (UFFI) extract to elicit bronchospasm or bronchial hypersensitivity. *Journal of Allergy and Clinical Immunology*, *75*(1), 143.
2. Frigas, E., & Reed, C. E. (1983). 191 Formaldehyde gas bronchial challenge does not provoke asthma. *Journal of Allergy and Clinical Immunology*, *71*(1), 136.
3. Girardi, P., Fracasso, M. E., Marcon, A., Doria, D., Guarda, L., Marchetti, P., ... & de Marco, R. (2012). Outdoor exposure to formaldehyde is associated with increased DNA damage and respiratory symptoms in children.
4. Gorski, P., Palczynski, C., Stankiewicz, J., Kolacinska, B., Ruta, U., Gruchala, J., ... & Hanke, W. (1996, January). Indoor exposure to formaldehyde at concentrations below 50 mu g/m (3) does not contribute to asthma development. In *JOURNAL OF ALLERGY AND CLINICAL IMMUNOLOGY* (Vol. 97, No. 1, pp. 93-93). 11830 WESTLINE INDUSTRIAL DR, ST LOUIS, MO 63146-3318: MOSBY-YEAR BOOK INC.
5. Lorenzini, S., & Knorst, M. M. (2013). Exposure Of Hairdressers To Formaldehyde: Short And Long-Term Effects On Symptoms And Lung Function. *Am J Respir Crit Care Med*, *187*, A3673.
6. Rios, J. L. M., Boechat, J. L., Ramos, M. C. K., Luiz, R. R., Neto, F. A., & e Silva, J. L. (2010). Asthma and rhinitis in office buildings workers and exposure to total volatile organic compounds (TVOC) and formaldehyde. *Journal of Allergy and Clinical Immunology*, *125*(2), AB210.
7. Rios, J. L. M., Boechat, J. L., Ramos, M. C. K., Luiz, R. R., Neto, F. A., & e Silva, J. L. (2011). Asthma Among Office Workers And Exposure To Chemical And Biological Indoor Pollutants. *Journal of Allergy and Clinical Immunology*, *127*(2), AB95.
8. Scheel, E. (1983). Formaldehyde Gas from Chipboard in Houses. *Tidsskrift for Den Norske Laegeforening,* 103;4.
9. Takahashi, K., Maeda, M., Matusmoto, T., Tanabe, K., Fuzimoto, S., Fukasaka, N., ... & Kimura, I. (1986). A community survey and pathogenesis of occupational asthma in sawing-and wood-workers. *The Japanese journal of thoracic diseases*, *24*(4), 447-453.
10. Witek, T., Schachter, E., Brody, D., Tosun, T., Beck, G., & Leaderer, B. (1985, January). A study of lung function and irritation from exposure to formaldehyde in routinely exposed laboratory workers. *Chest,* Vol. 88, pp. S6-S6.
11. Yoshioka, F., Azuma, E., Nakajima, T., Hashimoto, M., Toyoshima, K., & Komachi, Y. (2004). A strategy for assessing environmental influence on airway allergy using a regression binary tree-based method. *[Nihon koshu eisei zasshi] Japanese journal of public health*, *51*(8), 583-591.
12. Zaitseva, N., Shur, P., & Kiryanov, D. (2011). Determination of Formaldehyde Benchmark Level in Blood Using the Model “Blood Concentration—Odds Ratio”. *Epidemiology*, *22*(1), S72-S73.
13. Mezni, A. B., Babay, S., & Jemaâ, A. B. (2012). Asthme professionnel aux produits de pyrolyse des matières plastiques dans une entreprise de fabrication de masques de protection respiratoire. À propos de deux cas. *Revue Francaise d'Allergologie*, *52*(7), 474-479.
14. Fung, C. K. C., Yu, I. T. S., Goggins, W., & Li, A. M. (2011). Wheeze During the First 18 Months of Life: A Prospective Cohort Study to Explore the Associations With Indoor Nitrogen Dioxide and Formaldehyde—Preliminary Results. *Epidemiology*, *22*(1), S38.
15. Harving, H., Korsgaard, J., Dahl, R., Pedersen, O. F., & Mølhave, L. (1986). Low concentrations of formaldehyde in bronchial asthma: a study of exposure under controlled conditions. *British medical journal (Clinical research ed.)*, *293*(6542), 310.
16. Lees, R. E. M., Clark, R. H., & Day, J. H. (1985). 260 Respiratory responses to formaldehyde, formaldehyde free UFFI off-gas and particles in UFFI related asthma. *Journal of Allergy and Clinical Immunology*, *75*(1), 169.
17. Marchand, C., Le Calvé, S., Mirabel, P., Glasser, N., Casset, A., Schneider, N., & De Blay, F. (2007). Exposure to formaldehyde in asthmatic and control dwellings in the area of Strasbourg (France). *Journal of Allergy and Clinical Immunology*, *119*(1), S267.
18. Martins, P., Caires, I., Araújo-Martins, J., Valente, J., Lopes, M., Montuschi, P., ... & Neuparth, N. (2012). 8-isoprostane in exhaled breath condensate (EBC) and air pollution exposure in children with wheezing.
19. Mijakoski, D., Karadzinska-Bislimovska, J., Stoleski, S., Minov, J., & Marsenic, M. (2013). Respiratory symptoms, lung function tests, and sensitization to work-related allergens in female cleaners.
