# Supplementary material for: Exposure to formaldehyde and asthma outcomes: A systematic review, meta-analysis, and economic assessment
Source: PLoS One. 2021 Mar 31;16(3):e0248258. doi: 10.1371/journal.pone.0248258 (PMC8011796; doi:10.1371/journal.pone.0248258)
Supplement: S2 Results — (DOCX) [file pone.0248258.s120.docx]

Supplemental Results 2. List of Excluded Studies

Title and Abstract screening (n=3,658)

| **Author** | **Title** | **Year** |
| --- | --- | --- |
|  | Formalin asthma | 1977 |
|  | Toluene Diisocyanates | 1987 |
|  | Single and double lung transplantation | 1991 |
|  | Reducing poisonings in children | 1997 |
|  | Asthmagen? Critical assessments of the evidence for agents implicated in occupational asthma | 1999 |
|  | IPCS Concise International Chemical Assessment Documents: Asphalt (Bitumen) | 2004 |
|  | Do vaccines contain harmful preservatives, adjuvants, additives or manufacturing residuals? | 2004 |
|  | [Bronchial asthma--mildew as a cause to be identified] | 2007 |
|  | Percutaneous vertebroplasty for treatment of painful osteoporotic vertebral compression fractures: An evidence-based analysis | 2010 |
| Abbadi, A.,Lauer, M.,Swaidani, S.,Hascall, V. | Hyaluronan rafts in airway epithelial cells | 2014 |
| Abd El-Kareem Hussein, D.,Akl, Y. M.,El-Hindawi, A. A. | Idiopathic pleuroparenchymal fibroelastosis, young age presentation, with successful bronchoscopic closure of a complicating bronchopleural fistula: A case report | 2015 |
| Abel, M.,Vliagoftis, H. | Mast cell-fibroblast interactions induce matrix metalloproteinase-9 release from fibroblasts: Role for IgE-mediated mast cell activation | 2008 |
| Abou-Sayed, H. A.,Lesavoy, M. A.,Gruber, R. P. | Enlargement of nasal vault diameter with closed septoturbinotomy | 2007 |
| Agin,,K,,Gachkar,,L, | Assessment of allergic biomarkers; total immunoglobulin E antibodies levels and peripheral blood eosinophil among public transporter drivers with traffic-related respiratory diseases in Tehran | 2014 |
| Ahman,,M,,Alexandersson,,R,,Ekholm,,U,,Bergstrom,,B,,Dahlqvist,,M,,Ulfvarson,,U, | Impeded Lung Function in Molders and Coremakers Handling Furan Resin Sand | 1991 |
| Acosta, P. L.,Caballero, M. T.,Polack, F. P. | Brief History and Characterization of Enhanced Respiratory Syncytial Virus Disease | 2015 |
| Akbar, Khanzadeh,F,,Vaquerano,,M, U.,Akbar, Khanzadeh,M,,Bisesi,,M, S. | Formaldehyde exposure, acute pulmonary response, and exposure control options in a gross anatomy laboratory | 1994 |
| Adami, A. J.,Bracken, S. J.,Guernsey, L. A.,Thrall, R. S.,Adami, A. J.,Andemarium, B. | House Dust Mite-Induced Allergic Airway Disease Is Associated with Reduced Regulatory Immune Cells and Increased Pathology in a Murine Model of Sickle Cell Disease | 2014 |
| Adami, A. J.,Bracken, S. J.,Guernsey, L. A.,Thrall, R. S.,Andemarium, B. | House dust mite-induced allergic airway disease is associated with reduced regulatory immune cells and increased pathology in a murine model of sickle cell disease | 2014 |
| Akpinar, Elci,M,,Siegel,,P, D.,Cox, Ganser,J, M.,Stemple,,K, J.,White,,S, K.,Hilsbos,,K,,Weissman,,D, N. | Respiratory inflammatory responses among occupants of a water-damaged office building | 2008 |
| Al, Khatib,I, A.,Ju'ba,,A,,Kamal,,N,,Hamed,,N,,Hmeidan,,N,,Massad,,S, | Impact of housing conditions on the health of the people at al-Ama'ri refugee camp in the West Bank of Palestine | 2003 |
| Adetona, O.,Reinhardt, T. E.,Domitrovich, J.,Broyles, G.,Adetona, A. M.,Kleinman, M. T.,Ottmar, R. D.,Naeher, L. P. | Review of the health effects of wildland fire smoke on wildland firefighters and the public | 2016 |
| Adewoye, Adeboye H.,Safaya, Surinder,Frampton, Garrett,Lenburg, Marc,Klings, Elizabeth S.,Odhiambo, Adam,Li, Guihua,Gerry, Norman,Farber, Harrison W.,Steinberg, Martin H. | Pulmonary Artery Endothelial Cells Exposed to Acute Chest Syndrome Plasma Express a Novel Repertoire of Genes | 2002 |
| Adhikari, A.,Kettleson, E. M.,Vesper, S.,Kumar, S.,Popham, D. L.,Schaffer, C.,Indugula, R.,Chatterjee, K.,Allam, K. K.,Grinshpun, S. A.,Reponen, T. | Dustborne and airborne Gram-positive and Gram-negative bacteria in high versus low ERMI homes | 2014 |
| Adhikari, A.,Popham, D.,Schaffer, C.,Indugula, R.,Grinshpun, S.,Reponen, T. | Muramic acid in indoor air and dust: Relationship with home characteristics | 2013 |
| Adhikari, A.,Sen, M. M.,Gupta-Bhattacharya, S.,Chanda, S. | Volumetric assessment of airborne fungi in two sections of a rural indoor dairy cattle shed | 2004 |
| Afki, Z.,Herman, D.,Khairsyaf, O.,Medison, I. | The influeunce of cement dust exposure level to respiratory symptomps and pulmonary function in employers cohort of cement factory workers | 2013 |
| Alexandersson,,R,,Hedenstierna,,G, | Respiratory hazards associated with exposure to formaldehyde and solvents in acid-curing paints | 1988 |
| Alexandersson,,R,,Hedenstierna,,G, | Pulmonary-Function in Wood Workers Exposed to Formaldehyde - a Prospective-Study | 1989 |
| Agius, A. M.,Cordina, M.,Calleja, N. | The role of atopy in Maltese patients with chronic rhinitis | 2004 |
| Allen,,R, W.,Mar,,T,,Koenig,,J,,Liu,,L, J.,Gould,,T,,Simpson,,C,,Larson,,T, | Changes in lung function and airway inflammation among asthmatic children residing in a woodsmoke-impacted urban area | 2008 |
| Agritmis, A.,Takka, S. M. D.,Yurdaku, F. M. D. | Bilateral continous infraclavicular block for analgesia after bilateral hand degloving injury | 2010 |
| Agu, E.,Pedersen, P.,Strong, D.,Tulu, B.,He, Q.,Wang, L.,Li, Y. J. | The Smartphone as a Medical Device Assessing enablers, benefits and challenges | 2013 |
| Ahluwalia, S. K.,Matsui, E. C. | The indoor environment and its effects on childhood asthma | 2011 |
| Ahmadi, A.,Hadipour, N. L.,Kamfiroozi, M.,Bagheri, Z. | Theoretical study of aluminum nitride nanotubes for chemical sensing of formaldehyde | 2012 |
| Ahmed, F. E. | Toxicology and human health effects following exposure to oxygenated or reformulated gasoline | 2001 |
| Annesi, Maesano,I,,Norback,,D,,Zielinski,,J,,Bernard,,A,,Gratziou,,C,,Sigsgaard,,T,,Sestini,,P,,Viegi,,G, | Geriatric study in Europe on health effects of air quality in nursing homes (GERIE study) profile: Objectives, study protocol and descriptive data | 2013 |
| Ahnen, T.,Ahnen, M.,Wirth, U.,Schroll, A.,Schardey, H. M.,Schopf, S. | Pathophysiology of airway obstruction caused by wound hematoma after thyroidectomy: an ex vivo study | 2015 |
| Ainsworth, S. K.,Neuman, R. E.,Harley, R. A. | Histamine release from platelets for assay of byssinogenic substances in cotton mill dust and related materials | 1979 |
| Antti, Poika,M,,Nordman,,H,,Koskenvuo,,M,,Kaprio,,J,,Jalava,,M, | Role of Occupational Exposure to Airway Irritants in the Development of Asthma | 1992 |
| Akanbi, M. O.,Ukoli, C. O. | Respiratory symptoms and ventilatory function of cement loaders in Jos, North central Nigeria | 2010 |
| Araki,,A,,Kawai,,T,,Eitaki,,Y,,Kanazawa,,A,,Morimoto,,K,,Nakayama,,K,,Shibata,,E,,Tanaka,,M,,Takigawa,,T,,Yoshimura,,T,,Chikara,,H,,Saijo,,Y,,Kishi,,R, | Prevalence of asthma, atopic dermatitis, and rhinitis and MVOC exposure in single family homes-a survey in 6 cities of Japan | 2011 |
| Arif,,A, A.,Delclos,,G, L.,Serra,,C, | Occupational exposures and asthma among nursing professionals | 2009 |
| Al Ali, W.,Custovic, A.,Simpson, A.,Khoury, A.,Woodcock, A. | Household characteristics and allergen and endotoxin levels in Aleppo, Syrian Arab Republic | 2010 |
| Aris,,R,,Christian,,D,,Sheppard,,D,,Balmes,,J, R. | Acid fog-induced bronchoconstriction. The role of hydroxymethanesulfonic acid | 1990 |
| Alberts, W. M.,Do, P. I. C. O. G. A. | REACTIVE AIRWAYS DYSFUNCTION SYNDROME | 1996 |
| Austin,,J, B.,Russell,,G, | Wheeze, cough, atopy, and indoor environment in the Scottish Highlands | 1997 |
| Alexander, S.,Korman, M. G.,Sievert, W. | Cyanoacrylate in the treatment of gastric varices complicated by multiple pulmonary emboli | 2006 |
| Balmes,,J, R.,Fine,,J, M.,Gordon,,T,,Sheppard,,D, | Potential bronchoconstrictor stimuli in acid fog | 1989 |
| Alexandersson, R.,Hendenstierna, G.,Randma, E.,Rosen, G.,Swenson, A.,Tornling, G. | Symptoms and Lung Function in Low-Exposure to Toluene Diisocyanate by Polyurethane Foam Manufacturing | 1985 |
| Alexeieff, A. | Sur les cellules a poussieres du poumon et les "Herzfehlerzellen." Introduction an probleme de l'immunite | 1927 |
| Barreto,,M, L.,Cunha,,S, S.,Alcantara, Neves,N,,Carvalho,,L, P.,Cruz,,A, A.,Stein,,R, T.,Genser,,B,,Cooper,,P, J.,Rodrigues,,L, C. | Risk factors and immunological pathways for asthma and other allergic diseases in children: background and methodology of a longitudinal study in a large urban center in Northeastern Brazil (Salvador-SCAALA study) | 2006 |
| Bascom,,R, | Differential Responsiveness to Irritant Mixtures - Possible Mechanisms | 1992 |
| Beck,,G, J.,Schachter,,E, N.,Maunder,,L, R.,Schilling,,R, S. | Lung function in cotton-textile workers | 1984 |
| Beckett,,W, S.,Pope,,C, A.,Xu,,X, P.,Christiani,,D, C. | Women's respiratory health in the cotton textile industry: an analysis of respiratory symptoms in 973 non-smoking female workers | 1994 |
| Almeida, B. M.,Breda, G.,Muro, M. D.,Pinheio, R. L.,Queiroz-Telles, F. | Experience of a tertary care hospital in Cryptococosis gattii in Southern Brazil | 2012 |
| Alnassar, S.,Clifton, J.,Finley, R. J.,Sidhu, R. | The use of thoracoscopy to improve medical students' interest and understanding of thoracic anatomy | 2009 |
| Benicio,,M. H, D.,Ferreira,,M, U.,Cardoso,,M. R, A.,Konno,,S, C.,Monteiro,,C, A. | Wheezing conditions in early childhood: prevalence and risk factors in the city of Sao Paulo, Brazil | 2004 |
| Bentayeb,,M,,Norback,,D,,Bednarek,,M,,Bernard,,A,,Cai,,G,,Cerrai,,S,,Eleftheriou,,K, K.,Gratziou,,C,,Holst,,G, J.,Lavaud,,F,,Nasilowski,,J,,Sestini,,P,,Sarno,,G,,Sigsgaard,,T,,Wieslander,,G,,Zielinski,,J,,Viegi,,G,,Annesi, Maesano,I, | Indoor air quality, ventilation and respiratory health in elderly residents living in nursing homes in Europe | 2015 |
| Altissimi, G. | Total correction of the nose | 1991 |
| Berghout,,J,,Miller,,J, D.,Mazerolle,,R,,O'Neill,,L,,Wakelin,,C,,Mackinnon,,B,,Maybee,,K,,Augustine,,D,,Levi,,C, A.,Levi,,C,,Levi,,T,,Milliea,,B, | Indoor environmental quality in homes of asthmatic children on the Elsipogtog Reserve (NB), Canada | 2005 |
| Berke,,J, H. | Cytologic Examination of the Nasal Mucosa in Formaldehyde-Exposed Workers | 1987 |
| Amato, G.,Rottem, M.,Dahl, R.,Blaiss, M. S.,Ridolo, E.,Cecchi, L.,Rosario, N.,Motala, C.,Ansotegui, I.,Annesi-Maesano, I. | Climate change, migration, and allergic respiratory diseases: An update for the allergist | 2011 |
| Ambard-Bretteville, F.,Sorin, C.,Rebeille, F.,Hourton-Cabassa, C.,des Francs-Small, C. C. | Repression of formate dehydrogenase in Solanum tuberosum increases steady-state levels of formate and accelerates the accumulation of proline in response to osmotic stress | 2003 |
| Bertrand,,J, P.,Simon,,V,,Chau,,N, | Associations of symptoms related to isocyanate, ureaformol, and formophenolic exposures with respiratory symptoms and lung function in coal miners | 2007 |
| Amdur, M. O. | The respiratory response of guinea pigs to histamine aerosol | 1966 |
| Bhopal,,R, S.,Moffatt,,S,,Pless, Mulloli,T,,Phillimore,,P, R.,Foy,,C,,Dunn,,C, E.,Tate,,J, A. | Does living near a constellation of petrochemical, steel, and other industries impair health? | 1998 |
| Bielory,,L,,Deener,,A, | Seasonal variation in the effects of major indoor and outdoor environmental variables on asthma | 1998 |
| Amelizad, Z.,Appel, K. E.,Oesch, F.,Hildebrandt, A. G. | Effect of Antibodies against Cytochrome P-450 on Demethylation and Denitrosation of N Nitrosodimethylamine and N Nitrosomethylaniline | 1988 |
| Blanc,,P, D.,Eisner,,M, D.,Katz,,P, P.,Yen,,I, H.,Archea,,C,,Earnest,,G,,Janson,,S,,Masharani,,U, B.,Quinlan,,P, J.,Hammond,,S, K.,Thorne,,P, S.,Balmes,,J, R.,Trupin,,L,,Yelin,,E, H. | Impact of the home indoor environment on adult asthma and rhinitis | 2005 |
| American Society Of Biological, Chemists | Proceedings of twenty-eighth annual meeting, New York City, March 29-31, 1934 | 1934 |
| Boggs,,P, B. | Occupational asthma and rhinitis caused by urea formaldehyde? | 1983 |
| Bohadana,,A, B.,Massin,,N,,Wild,,P,,Toamain,,J, P.,Engel,,S,,Goutet,,P, | Symptoms, airway responsiveness, and exposure to dust in beech and oak wood workers | 2000 |
| Boman,,B, C.,Forsberg,,A, B.,Jarvholm,,B, G. | Adverse health effects from ambient air pollution in relation to residential wood combustion in modern society | 2003 |
| Bonner,,S,,Matte,,T, D.,Fagan,,J,,Andreopoulos,,E,,Evans,,D, | Self-reported moisture or mildew in the homes of Head Start children with asthma is associated with greater asthma morbidity | 2006 |
| Bornehag,,C, G.,Sundell,,J,,Hagerhed, Engman,L,,Sigsggard,,T,,Janson,,S,,Aberg,,N, | 'Dampness' at home and its association with airway, nose, and skin symptoms among 10,851 preschool children in Sweden: a cross-sectional study | 2005 |
| Bornehag,,C, G.,Sundell,,J,,Sigsgaard,,T, | Dampness in buildings and health (DBH): Report from an ongoing epidemiological investigation on the association between indoor environmental factors and health effects among children in Sweden | 2004 |
| Boskabady,,M, H.,Karimiani,,E, G.,Vostacolaei,,H, A. | Respiratory symptoms and pulmonary function changes among carpet weavers in Iran | 2007 |
| Bouhuys,,A, | The forced expiratory volume (FEV-0.75) in healthy males and in textile workers | 1963 |
| Bouhuys,,A,,Wolfson,,R, L.,Horner,,D, W.,Brain,,J, D.,Zuskin,,E, | Byssinosis in cotton textile workers. Respiratory survey of a mill with rapid labor turnover | 1969 |
| Anderson, R. C.,Anderson, J. H. | Respiratory toxicity of mattress emissions in mice | 2000 |
| Bousquet,,J,,Michel,,F, B. | Allergy to Formaldehyde and Ethylene-Oxide | 1991 |
| Bozicevic,,I,,Oreskovic,,S, | Risk factors in asthmatic patients in Croatia | 2000 |
| Brabin,,B,,Smith,,M,,Milligan,,P,,Benjamin,,C,,Dunne,,E,,Pearson,,M, | Respiratory Morbidity in Merseyside Schoolchildren Exposed to Coal-Dust and Air-Pollution | 1994 |
| Andius, P.,Arakawa, H.,Molne, J.,Pullerits, T.,Skoogh, B. E.,Lotvall, J. | Inflammatory responses in skin and airways after allergen challenge in brown Norway rats sensitized to trimellitic anhydride | 1996 |
| Andius, P.,Arakawa, H.,Molne, J.,Pullertis, T.,Skoogh, B. E.,Lotvall, J. | Inflammatory responses in skin and airways after allergen challenge in brown Norway rats sensitized to trimellitic anhydride | 1996 |
| Branco,,Ptbs,,Nunes,,R. A, O.,Alvim, Ferraz,M. C, M.,Martins,,F, G.,Souse,,S. I, V. | Children's exposure to indoor air in urban nurseries - Part II: Gaseous pollutants' assessment | 2015 |
| Braun, Fahrlander,C, | Allergic diseases in farmers' children | 2000 |
| Breysse,,P, N.,Buckley,,T, J.,Williams,,D,,Beck,,C, M.,Jo,,S, J.,Merriman,,B,,Kanchanaraksa,,S,,Swartz,,L, J.,Callahan,,K, A.,Butz,,A, M.,Rand,,C, S.,Diette,,G, B.,Krishnan,,J, A.,Moseley,,A, M.,Curtin, Brosnan,J,,Durkin,,N, B.,Eggleston,,P, A. | Indoor exposures to air pollutants and allergens in the homes of asthmatic children in inner-city Baltimore | 2005 |
| Andrychiewicz, A.,Gorka, K.,Reid, M.,Soja, J.,Sladek, K.,Szczeklik, W. | Modern methods for endoscopic treatment of obstructive pulmonary diseases | 2015 |
| Broadwell,,D, K.,Darcey,,D, J.,Hudnell,,H, K.,Otto,,D, A.,Boyes,,W, K. | Work-Site Clinical and Neurobehavioral Assessment of Solvent-Exposed Microelectronics Workers | 1995 |
| Brochhage,,F, | Do Amines Induce Occupational Asthma in Workers Manufacturing Polyurethane Foams | 1985 |
| Brugge,,D,,Welker, Hood,K,,Kosheleva,,A,,Saddler,,S, | Association and correlation of self-reported home environmental factors and health symptoms | 2006 |
| Brunelli,,A,,Varela,,G, | External Validation of the Recalibrated Thoracic Revised Cardiac Risk Index for Predicting the Risk of Major Cardiac Complications After Lung Resection | 2011 |
| Anon, | Rapid increase in latex allergies | 1994 |
| Buess,,H,,Lerner,,R, | Bronchial Asthma And Asthmatic Bronchitis In The Chemical Industry | 1956 |
| Anon, | Rosin | 2920 |
| Bui,,D, S.,Burgess,,J, A.,Matheson,,M, C.,Erbas,,B,,Perret,,J,,Morrison,,S,,Giles,,G, G.,Hopper,,J, L.,Thomas,,P, S.,Markos,,J,,Abramson,,M, J.,Walters,,E, H.,Dharmage,,S, C. | Ambient wood smoke, traffic pollution and adult asthma prevalence and severity | 2013 |
| Anon, J. B. | Allergy | 1996 |
| Anonymous, | Cetirizine-A new antihistamine | 1996 |
| Burge,,P, S.,Perks,,W,,O'Brien,,I, M.,Hawkins,,R,,Green,,M, | Occupational Asthma In An Electronics Factory | 1979 |
| Burton,,C,,Bradshaw,,L,,Agius,,R,,Burge,,S,,Huggins,,V,,Fishwick,,D, | Medium-density fibreboard and occupational asthma. A case series | 2011 |
| Antens, C. J.,Oldenwening, M.,Wolse, A.,Gehring, U.,Smit, H. A.,Aalberse, R. C.,Kerkhof, M.,Gerritsen, J.,de Jongste, J. C.,Brunekreef, B. | Repeated measurements of mite and pet allergen levels in house dust over a time period of 8 years | 2006 |
| Antova, T.,Pattenden, S.,Brunekreef, B.,Heinrich, J.,Rudnai, P.,Forastiere, F.,Luttmann-Gibson, H.,Grize, L.,Katsnelson, B.,Moshammer, H.,Nikiforov, B.,Slachtova, H.,Slotova, K.,Zlotkowska, R.,Fletcher, T. | Exposure to indoor mould and children's respiratory health in the PATY study | 2008 |
| Caron,,S,,Boileau,,J, C.,Malo,,J, L.,Leblond,,S, | New methodology for specific inhalation challenges with occupational agents | 2010 |
| Carosso,,A,,Ruffino,,C,,Bugiani,,M, | Respiratory diseases in wood workers | 1987 |
| Cartieaux,,E,,Rzepka,,M, A.,Cuny,,D, | Indoor air quality in schools | 2011 |
| Casset,,A,,Marchand,,C,,Purohit,,A,,le, Calve,S,,Uring, Lambert,B,,Donnay,,C,,Meyer,,P,,de, Blay,F, | Inhaled formaldehyde exposure: effect on bronchial response to mite allergen in sensitized asthma patients | 2006 |
| Arafa, Safaa Zaky,Reda, Enayat Salem | Surface features of the monogenean gill parasites Pseudodactylogyrus anguillae and Pseudodactylogyrus bini from the European eel Anguilla anguilla in Egypt | 2012 |
| Araki, A.,Saito, I.,Kanazawa, A.,Morimoto, K.,Nakayama, K.,Shibata, E.,Tanaka, M.,Takigawa, T.,Yoshimura, T.,Chikara, H.,Saijo, Y.,Kishi, R. | Phosphorus flame retardants in indoor dust and their relation to asthma and allergies of inhabitants | 2013 |
| Araki, A.,Tsuboi, T.,Kawai, T.,Bamai, Y. A.,Takeda, T.,Yoshioka, E.,Kishi, R. | Validation of diffusive mini-samplers for aldehyde and VOC and its feasibility for measuring the exposure levels of elementary school children | 2011 |
| Chang,,C, C.,Ruhl,,R, A.,Halpern,,G, M.,Gershwin,,M, E. | Building Components Contributors of the Sick Building Syndrome | 1994 |
| Chapman,,M, D. | Measuring allergen exposure in the home: who benefits? | 2001 |
| Chapman,,M, D. | Asthma in the third world: can environmental intervention improve childhood asthma in U.S. inner cities? | 2006 |
| Cheng,,Y, S.,Zhou,,Y,,Chen,,B, T. | Particle deposition in a cast of human oral airways | 1999 |
| Arima, M.,Tada, M. | Endoscopic ultrasound-guided fine needle aspiration biopsy in esophageal and mediastinal diseases: Clinical indications and results | 2004 |
| Chia,,S, E.,Ong,,C, N.,Foo,,S, C.,Lee,,H, P. | Medical students' exposure to formaldehyde in a gross anatomy dissection laboratory | 1992 |
| Arndt, V.,Rothenbacher, D.,Brenner, H.,Fraisse, E.,Zschenderlein, B.,Daniel, U.,Schuberth, S.,Fliedner, T. M. | Older workers in the construction industry: results of a routine health examination and a five year follow up | 1996 |
| Cho,,Ara,,Jang,,Hong, Seok,Roh,,Yoon, Seok,Park,,Hee, Jin,Talha,,A. F. S, M.,So,,Seung, Young,Lim,,Chae, Woong,Kim,,Bumseok, | Detrimental effects of cement mortar and fly ash mortar on asthma progression | 2013 |
| Cho,,Y, M.,Ryu,,S, H.,Choi,,M, S.,Tinyami,,E, T.,Seo,,S,,Choung,,J, T.,Choi,,J, W. | Asthma and allergic diseases in preschool children in Korea: findings from the pilot study of the Korean Surveillance System for Childhood Asthma | 2014 |
| Arrandale, V. H.,Liss, G. M.,Tarlo, S. M.,Pratt, M. D.,Sasseville, D.,Kudla, I.,Holness, D. L. | Occupational contact allergens: are they also associated with occupational asthma? | 2012 |
| Choi,,J,,Choi,,Y,,Chun,,C,,Sun,,Y,,Sundell,,J, | A study on correlation of home environment and asthma and allergy among preschool children in Seoul, Korea | 2011 |
| Choi,,J,,Hwang,,G, | A case-control study: Exposure assessment of volatile organic compounds and formaldehyde for asthma in children | 2011 |
| Chow,,K, K.,Short,,M,,Zeng,,H, | A comparison of spectroscopic techniques for human breath analysis | 2012 |
| Christiani,,D, C.,Eisen,,E, A.,Wegman,,D, H.,Ye,,T, T.,Gong,,Z, C.,Lu,,P, L.,Dai,,H, L. | Respiratory disease in cotton textile workers in the People's Republic of China. II. Pulmonary function results | 1986 |
| Christiani,,D, C.,Wang,,X, R.,Pan,,L, D.,Zhang,,H, X.,Sun,,B, X.,Dai,,H,,Eisen,,E, A.,Wegman,,D, H.,Olenchock,,S, A. | Longitudinal changes in pulmonary function and respiratory symptoms in cotton textile workers. A 15-yr follow-up study | 2001 |
| Christiani,,D, C.,Ye,,T, T.,Zhang,,S,,Wegman,,D, H.,Eisen,,E, A.,Ryan,,L, A.,Olenchock,,S, A.,Pothier,,L,,Dai,,H, L. | Cotton dust and endotoxin exposure and long-term decline in lung function: results of a longitudinal study | 1999 |
| Chuang,,H, C.,Jones,,T,,Chen,,Y,,Bell,,J,,Wenger,,J,,BeruBe,,K, | Characterisation of airborne particles and associated organic components produced from incense burning | 2011 |
| Audibert, G.,Saunier, C.,Hartemann, D.,Bigard, O.,Haberer, J. P. | Effects of H2-receptor blockers on response of cerebral blood flow to normocapnic hypoxia | 1991 |
| Augustyniak, D.,Piekut, M.,Majkowska-Skrobek, G.,Skala, J. | Bactericidal, opsonophagocytic and anti-adhesive effectiveness of cross-reactive antibodies against Moraxella catarrhalis | 2015 |
| Clarisse,,B,,Laurent,,A, M.,Seta,,N,,Le, Moullec,Y,,El, Hasnaoui,A,,Momas,,I, | Indoor aldehydes: measurement of contamination levels and identification of their determinants in Paris dwellings | 2003 |
| Claudio,,L,,Tulton,,L,,Doucette,,J,,Landrigan,,P, J. | Socioeconomic factors and asthma hospitalization rates in New York City | 1999 |
| Awortwe, C.,Osei-Safo, D.,Asiedu-Gyekye, I. J.,Sackeyfio, A. C. | The anti-inflammatory activity of Taraxacum officinale leaves in ovalbumin-sensitized guinea-pigs | 2013 |
| Cloutier,,Y,,Perrault,,G,,Drolet,,D,,Cartier,,A,,Lemiere,,C,,Malo,,J, L. | Closed-circuit apparatus for specific inhalation challenges with occupational agents in vapour form: Application to formaldehyde | 1996 |
| Cockcroft,,D, W.,Hoeppner,,V, H.,Dolovich,,J, | Occupational asthma caused by cedar urea formaldehyde particle board | 1982 |
| Ayars, G. H.,Altman, L. C.,Frazier, C. E.,Chi, E. Y. | The toxicity of constituents of cedar and pine woods to pulmonary epithelium | 1989 |
| Ayaz, E. A.,Bagis, B.,Turgut, S. | Effect of antiasthmatic medication on the surface roughness and color stability of dental restorative materials | 2013 |
| Cohen,,A, J.,Forse,,M, S.,Tarlo,,S, M. | Occupational Asthma Caused by Pectin Inhalation during the Manufacture of Jam | 1993 |
| Colton,,M,,Hsu,,J,,Chew,,G,,Brugge,,D,,Yip,,F,,Sircar,,K,,Ryan,,P, H.,Sandel,,M,,Adamkiewicz,,G, | Characterizing asthma morbidity and the indoor environment of low-income, chinese-american children in the green housing study | 2014 |
| Bacci, E.,Paggiaro, P. L.,Dente, F. L.,Morelli, M. C.,Talini, D.,Battaglia, A.,Giuntini, C. | Non-specific Bronchial Hyperresponsiveness Induced by Occupational Exposure to Different Irritants | 1989 |
| Corburn,,J,,Osleeb,,J,,Porter,,M, | Urban asthma and the neighbourhood environment in New York City | 2005 |
| Baccioglu, A.,Kalpalklioglu, A. F. | An unusual form of formaldehyde induced lung disease | 2007 |
| Bachert, C. | Sublingual immunotherapy. A survey on the basis of controlled studies on efficacy, tolerability, long-term effects and prevention in children and adults with ALK-Scherax preparations | 2007 |
| Backhouse, Nadine,Delporte, Carla,Apablaza, Cecia,Farias, Mariela,Goity, Leon,Arrau, Sylvia,Negrete, Rosa,Castro, Consuelo,Miranda, Hugo | Antinociceptive activity of Buddleja globosa (matico) in several models of pain | 2008 |
| Cox, Ganser,J, M.,Rao,,C, Y.,Park,,J, H.,Schumpert,,J, C.,Kreiss,,K, | Asthma and respiratory symptoms in hospital workers related to dampness and biological contaminants | 2009 |
| Crain,,E, F.,Walter,,M,,O'Connor,,G, T.,Mitchell,,H,,Gruchalla,,R, S.,Kattan,,M,,Malindzak,,G, S.,Enright,,P,,Evans,,R,,rd,,Morgan,,W,,Stout,,J, W. | Home and allergic characteristics of children with asthma in seven U.S. urban communities and design of an environmental intervention: the Inner-City Asthma Study | 2002 |
| Bahaie, N. S.,Hosseinkhani, M. R.,Ge, X. N.,Kang, B. N.,Ha, S. G.,Blumenthal, M. S.,Jessberger, R.,Rao, S. P.,Sriramarao, P. | Regulation of Eosinophil Trafficking by SWAP-70 and Its Role in Allergic Airway Inflammation | 2012 |
| Curran,,A, D.,Burge,,P, S.,Wiley,,K, | Clinical and immunologic evaluation of workers exposed to glutaraldehyde | 1996 |
| Custovic,,A,,Wijk,,R, G. | The effectiveness of measures to change the indoor environment in the treatment of allergic rhinitis and asthma: ARIA update (in collaboration with GA(2)LEN) | 2005 |
| Daigler,,G, E.,Markello,,S, J.,Cummings,,K, M. | The effect of indoor air pollutants on otitis media and asthma in children | 1991 |
| Dalal,,A, | A sticky hazard for the airway | 1985 |
| Dales,,R, E.,Zwanenburg,,H,,Burnett,,R,,Franklin,,C, A. | Respiratory health effects of home dampness and molds among Canadian children | 1991 |
| Dallongeville,,A,,Costet,,N,,Zmirou, Navier,D,,Le, Bot,B,,Chevrier,,C,,Deguen,,S,,Annesi, Maesano,I,,Blanchard,,O, | Volatile and semi-volatile organic compounds of respiratory health relevance in French dwellings | 2015 |
| Darcey,,D,,Lipscomb,,H, J.,Epling,,C,,Pate,,W,,Cherry,,L, P.,Bernstein,,J, | Clinical findings for residents near a polyurethane foam manufacturing plant | 2003 |
| Balachandran, C.,Duraipandiyan, V.,Balakrishna, K.,Sundaram, R. L.,Vijayakumar, A.,Ignacimuthu, S.,Al-Dhabi, N. A. | Synthesis and medicinal properties of plant-derived vilangin | 2013 |
| Balasubramanian, R.,Nainar, P.,Rajasekar, A. | Airborne bacteria, fungi, and endotoxin levels in residential microenvironments: a case study | 2012 |
| Balharry, D.,Sexton, K.,BeruBe, K. A. | An in vitro approach to assess the toxicity of inhaled tobacco smoke components: Nicotine, cadmium, formaldehyde and urethane | 2008 |
| Baloǧlu, H.,Küçükodaci, Z.,Güngör, A.,Haholu, A.,Cincik, H.,Yaşayan, G.,Gökçe Örsçelik, S. | Human papilloma virus prevalence in hyperplastic tonsils and adenoids in children and young adults | 2010 |
| Baloglu, H.,Kucukodaci, Z.,Gungor, A.,Haholu, A.,Cincik, H.,Yasayan, G.,Orscelik, S. G. | Human Papilloma Virus Prevalence in Hyperplastic Tonsils and Adenoids in Children and Young Adults | 2010 |
| de Marco, R.,Marcon,,A,,Rava,,M,,Cazzoletti,,L,,Pironi,,V,,Silocchi,,C,,Ricci,,P, | Proximity to chipboard industries increases the risk of respiratory and irritation symptoms in children The Viadana study | 2010 |
| Banerjee, E. R.,Jiang, Y.,Henderson, W. R.,Scott, L. M.,Papayannopoulou, T. | alpha 4 and beta 2 integrins have nonredundant roles for asthma development, but for optimal allergen sensitization only alpha 4 is critical | 2007 |
| De Vos, A.J.B.M.,Reisen,,F,,Cook,,A,,Devine,,B,,Weinstein,,P, | Respiratory irritants in Australian bushfire smoke: air toxics sampling in a smoke chamber and during prescribed burns | 2009 |
| Banks, D. E.,Sastre, J.,Butcher, B. T.,Ellis, E.,Rando, R. J.,Barkman, H. W., Jr.,Hammad, Y. Y.,Glindmeyer, H. W.,Weill, H. | Role of inhalation challenge testing in the diagnosis of isocyanate-induced asthma | 1989 |
| Barakzai, S. Z.,Es, C.,Milne, E. M.,Dixon, P. | Ventroaxial luxation of the apex of the corniculate process of the arytenoid cartilage in resting horses during induced swallowing or nasal occlusion | 2007 |
| Barbara, J.,Leynadier, F. | Retention of dust mite allergens by a fabric used for the manufacturing of integral mattress encasements | 2006 |
| Barber, R. D.,Donohue, T. J. | Function of a glutathione-dependent formaldehyde dehydrogenase in Rhodobacter sphaeroides formaldehyde oxidation and assimilation | 1998 |
| Barber, Robert D.,Rott, Marc A.,Donohue, Timothy J. | Characterization of a glutathione-dependent formaldehyde dehydrogenase from Rhodobacter sphaeroides | 1996 |
| Barbera, J. A.,Ramirez, J.,Lopez, F. A.,Roca, J.,Rodriguez-Roisin, R. | New design for fixation of surgically obtained lung specimens | 1989 |
| Barcellos, B. M.,Camelier, P. D. S.,De Jesus, L. E. | Electrocardiographic Monitoring in Patients with Epidermolysis Bullosa: A New Proposal | 2015 |
| Bardana, E. J., Jr. | Building-Related Illness | 1992 |
| Deger,,L,,Plante,,C,,Goudreau,,S,,Smargiassi,,A,,Perron,,S,,Thivierge,,R, L.,Jacques,,L, | Home Environmental Factors Associated With Poor Asthma Control in Montreal Children: A Population-Based Study | 2010 |
| Bardana, E. J. | Indoor pollution and its impact on respiratory health | 2001 |
| Delaney,,P,,Healy,,R, M.,Hanrahan,,J, P.,Gibson,,L, T.,Wenger,,J, C.,Morris,,M, A.,Holmes,,J, D. | Porous silica spheres as indoor air pollutant scavengers | 2010 |
| Bardana Jr, E. J. | Formaldehyde: Hypersensitivity and irritant reactions at work and in the home | 1980 |
| Delclos,,George, L.,Gimeno,,David,,Arif,,Ahmed, A.,Benavides,,Fernando, G.,Zock,,Jan, Paul | Occupational Exposures and Asthma in Health-Care Workers: Comparison of Self-Reports With a Workplace-Specific Job Exposure Matrix | 2009 |
| Delclos,,George, L.,Gimeno,,David,,Arif,,Ahmed, A.,Burau,,Keith, D.,Carson,,Arch,,Lusk,,Christine,,Stock,,Thomas,,Symanski,,Elaine,,Whitehead,,Lawrence, W.,Zock,,Jan, Paul,Benavides,,Fernando, G.,Anto,,Josep, M. | Occupational risk factors and asthma among health care professionals | 2007 |
| Delfino,,R, J. | Evaluation of health effects of toxic air pollutants in a southern California community: A pilot study | 2002 |
| Barker, Jane E.,Hampton, Tom G.,Schuldt, Adam J.,Vogler, Carole A. | Alleviation of heart defects in MPS VII mice by transplantation of hematopoietic cells into non-ablated neonates | 2001 |
| Barnes, C.,Dinakar, C.,Reddy, M.,Portnoy, J. | Frequency of fungi in homes of pediatric allergy patients | 2006 |
| Delfino,,R, J.,Staimer,,N,,Tjoa,,T, | Personal endotoxin exposure in a panel study of school children with asthma | 2011 |
| Barth, R. F.,Robinson, R. G.,Bradshaw, D.,Singla, O. | Labeling and Distribution of Technetium-99m Labeled Corynebacterium-Parvum Propionibacterium-Acnes as Determined by Whole Body Imaging | 1981 |
| Barthel, S. R.,Johansson, M. W.,McNamee, D. M.,Mosher, D. F. | Roles of integrin activation in eosinophil function and the eosinophilic inflammation of asthma | 2008 |
| Basagana, X.,Torrent, M.,Atkinson, W.,Puig, C.,Barnes, M.,Vall, O.,Jones, M.,Sunyer, J.,Cullinan, P. | Domestic aeroallergen levels in Barcelona and Menorca (Spain) | 2002 |
| Bascom, R.,Kesavanathan, J. | Differential susceptibility to inhaled pollutants: Effects of demographics and diseases | 1997 |
| Bashir, S. A. | Home is where the harm is: inadequate housing as a public health crisis | 2002 |
| Basketter, D.,Kimber, I. | Consideration of criteria required for assignment of a (skin) sensitiser a substance of very high concern (SVHC) under the REACH regulation | 2014 |
| Basketter, D. A.,Gilmour, N. J.,Wright, Z. M.,Walters, T.,Boman, A.,Liden, C. | Biocides: Characterization of the allergenic hazard of methylisothiazolinone | 2003 |
| Dietemann, Molard,A,,Kopferschmitt, Kubler,M, C.,Meyer,,P, D.,Tomb,,R,,Pauli,,G, | Allergic asthma due to domestic use of insulating polyurethane foam | 1991 |
| Bates, D. V.,Baker-Anderson, M.,Sizto, R. | Asthma Attack Periodicity a Study of Hospital Emergency Visits in Vancouver British Columbia Canada | 1990 |
| Batterman, S.,Du, L.,Mentz, G.,Mukherjee, B.,Parker, E.,Godwin, C.,Chin, J. Y.,O'Toole, A.,Robins, T.,Rowe, Z.,Lewis, T. | Particulate matter concentrations in residences: an intervention study evaluating stand-alone filters and air conditioners | 2012 |
| Diette,,Gregory, B.,Hansel,,Nadia, N.,Buckley,,Timothy, J.,Curtin, Brosnan,Jean,,Eggleston,,Peyton, A.,Matsui,,Elizabeth, C.,McCormack,,Meredith, C.,Williams,,D'Ann, L.,Breysse,,Patrick, N. | Home indoor pollutant exposures among inner-city children with and without asthma | 2007 |
| Diez,,U,,Kroessner,,T,,Rehwagen,,M,,Richter,,M,,Wetzig,,H,,Schulz,,R,,Borte,,M,,Metzner,,G,,Krumbiegel,,P,,Herbarth,,O, | Effects of indoor painting and smoking on airway symptoms in atopy risk children in the first year of life results of the LARS-study. Leipzig Allergy High-Risk Children Study | 2000 |
| Dimitroulopoulou,,C,,Trantallidi,,M,,Carrer,,P,,Efthimiou,,G, C.,Bartzis,,J, G. | EPHECT II: Exposure assessment to household consumer products | 2015 |
| Baur, X. | [Obstructive airway diseases due to occupation and profession] | 2006 |
| Baur, X.,Bakehe, P.,Vellguth, H. | Bronchial asthma and COPD due to irritants in the workplace - An evidence-based approach | 2012 |
| Baur, X.,Borsch-Galetke, E.,Raulf, M.,Czuppon, A. B.,Scheer, E. | Occupational-type exposure tests and bronchoalveolar lavage analyses in two patients with byssinosis and two asymptomatic cotton workers | 1993 |
| Doi,,S,,Suzuki,,S,,Morishita,,M,,Yamada,,M,,Kanda,,Y,,Torii,,S,,Sakamoto,,T, | The prevalence of IgE sensitization to formaldehyde in asthmatic children | 2003 |
| Dong,,G, H.,Ding,,H, L.,Ma,,Y, N.,Jin,,J,,Cao,,Y,,Zhao,,Y, D.,He,,Q, C. | Housing characteristics, home environmental factors and respiratory health in 14,729 Chinese children | 2008 |
| Dong,,G, H.,Ma,,Y, N.,Ding,,H, L.,Jin,,J,,Cao,,Y,,Zhao,,Y, D.,He,,Q, C. | Effects of housing characteristics and home environmental factors on respiratory symptoms of 10,784 elementary school children from northeast china | 2008 |
| Dong,,G, H.,Ma,,Y, N.,Ding,,H, L.,Jin,,J,,Cao,,Y,,Zhao,,Y, D.,He,,Q, C. | Housing characteristics, home environmental factors and respiratory health in 3945 pre-school children in China | 2008 |
| Dong,,G, H.,Qian,,Z,,Fu,,Q,,Wang,,J,,Trevathan,,E,,Ma,,W,,Liu,,M, M.,Wang,,D,,Ren,,W, H.,Ong,,K, H.,Ferguson,,T, F.,Riley,,E,,Simckes,,M, | A Multiple Indicators Multiple Cause (MIMIC) model of respiratory health and household factors in Chinese children: the seven Northeastern cities (SNEC) study | 2014 |
| Dong,,G, H.,Qian,,Z, M.,Wang,,J,,Trevathan,,E,,Liu,,M, M.,Wang,,D,,Ren,,W, H.,Chen,,W,,Simckes,,M,,Zelicoff,,A, | Home renovation, family history of atopy, and respiratory symptoms and asthma among children living in China | 2014 |
| Baxter, L. K.,Clougherty, J. E.,Laden, F.,Levy, J. I. | Predictors of concentrations of nitrogen dioxide, fine particulate matter, and particle constituents inside of lower socioeconomic status urban homes | 2006 |
| Dotterud,,C, K.,Storro,,O,,Simpson,,M, R.,Johnsen,,R,,Oien,,T, | The impact of pre- and postnatal exposures on allergy related diseases in childhood: a controlled multicentre intervention study in primary health care | 2013 |
| Bazan-Socha, S.,Bukiej, A.,Marcinkiewicz, C.,Musial, J. | Integrins in pulmonary inflammatory diseases | 2005 |
| Bazan-Socha, S.,Bukiej, A.,Pulka, G.,Marcinkiewicz, C.,Musial, J. | Increased expression of collagen receptors: alpha(1)beta(1) and alpha(2)beta(1) integrins on blood eosinophils in bronchial asthma | 2006 |
| Bazan-Socha, S.,Zuk, J.,Jakiela, B.,Pulka, G.,Pelka, K.,Musial, J. | Increased expression of alpha(2) (CD49b), alpha(4) (CD49d) and beta(1) (CD29) integrin subunits on peripheral blood T lymphocytes in clinically stable mild-to-moderate persistent asthma | 2012 |
| Bazan-Socha, S.,Zuk, J.,Macinkiewicz, C.,Zareba, L.,Plutecka, H.,Mlicka-Kowalczyk, E.,Musial, J. | Inhibition of collagen receptors: α1β1 and α2β1 integrins decreases eosinophil transmigration, but has no impact on peripheral blood mononuclear cell movement through human microvascular endothelial cell lung monolayer | 2012 |
| Bazan-Socha, S.,Zuk, J.,Marcinkiewicz, C.,Musial, J. | The impact of viperistatin and VP12, snake venom derived inhibitors of collagen receptors α1β1 and α2β1 integrins, on eosinophils and lymphocytes transmigration through human microvascular endothelial cells M | 2012 |
| Bazan-Socha, S.,Zuk, J.,Musial, J. | Increased expression of selected very late antigen integrin subunits on CD4 and CD8 blood T lymphocytes in patients with clinically stable asymptomatic atopic asthma | 2012 |
| Dykewicz,,M, S.,Patterson,,R,,Cugell,,D, W.,Harris,,K, E.,Wu,,A, F. | Serum IgE and IgG to formaldehyde-human serum albumin: lack of relation to gaseous formaldehyde exposure and symptoms | 1991 |
| Dymek,,A,,Dymek,,L,,Bozek,,A, | The presence of specific IgE to formaldehyde in patients with suspicion of hypersensitivity to this agent | 2004 |
| Ebbehoj,,N, E.,Meyer,,H, W.,Wurtz,,H,,Suadicani,,P,,Valbjorn,,O,,Sigsgaard,,T,,Gyntelberg,,F, | Molds in floor dust, building-related symptoms, and lung function among male and female schoolteachers | 2005 |
| Ebisu,,K,,Holford,,T, R.,Belanger,,K, D.,Leaderer,,B, P.,Bell,,M, L. | Urban land-use and respiratory symptoms in infants | 2011 |
| Edling,,C,,Hellquist,,H,,Odkvist,,L, | Occupational exposure to formaldehyde and histopathological changes in the nasal mucosa | 1988 |
| Eggleston,,P, A.,Butz,,A,,Rand,,C,,Curtin, Brosnan,J,,Kanchanaraksa,,S,,Swartz,,L,,Breysse,,P,,Buckley,,T,,Diette,,G,,Merriman,,B,,Krishnan,,J, A. | Home environmental intervention in inner-city asthma: a randomized controlled clinical trial | 2005 |
| Ehnert,,B,,Lau, Schadendorf,S,,Weber,,A,,Buettner,,P,,Schou,,C,,Wahn,,U, | Reducing domestic exposure to dust mite allergen reduces bronchial hyperreactivity in sensitive children with asthma | 1992 |
| Eisen,,E, A.,Oliver,,L, C.,Christiani,,D, C.,Robins,,J, M.,Wegman,,D, H. | Effects of spirometry standards in two occupational cohorts | 1985 |
| Eisner,,M, D.,Blanc,,P, D. | Gas stove use and respiratory health among adults with asthma in NHANES III | 2003 |
| Elliott,,L,,Arbes,,S, J.,Harvey,,E, S.,Lee,,R, C.,Salo,,P, M.,Cohn,,R, D.,London,,S, J.,Zeldin,,D, C. | Dust weight and asthma prevalence in the National Survey of Lead and Allergens in Housing (NSLAH) | 2007 |
| Elwood,,P, C.,Sweetnam,,P, M.,Bevan,,C,,Saunders,,M, J. | Respiratory disability in ex-cotton workers | 1986 |
| Beers, C. Dale | The chemical nature and function of the endoplasmic granules of the ciliate Conchophthirus curtus | 1962 |
| Epton,,M, J.,Dawson,,R, D.,Brooks,,W, M.,Kingham,,S,,Aberkane,,T,,Cavanagh,,J, A.,Frampton,,C, M.,Hewitt,,T,,Cook,,J, M.,McLeod,,S,,McCartin,,F,,Trought,,K,,Brown,,L, | The effect of ambient air pollution on respiratory health of school children: a panel study | 2008 |
| Behar, S. M. | Treatment with alpha-galactosylceramide prolongs the survival of mice following Mycobacterium tuberculosis infection | 2002 |
| Beilman, G. J.,Shield, C. F., 3rd,Hughes, J. D.,Kelley, H. K.,Ward, L. G.,Beck, D. | The effects of intraoperative administration of OKT3 during renal transplantation | 1993 |
| Beinert, T.,Schaubschlaeger, W.,Vogelmeier, C.,Schluckebier, S.,Behr, J.,Fruhmann, G. | Does formaldehyde potentiate the antigen-antibody reaction? Studies of the mediator release threshold of basophil granulocytes in vitro | 1994 |
| Beinert, T.,Schaubschlager, W.,Vogelmeier, C.,Schluckebier, S.,Behr, J.,Fruhmann, G. | Does formaldehyde potentiate the antigen-antibody reaction? Studies on the mediator release threshold of basophilic granulocytes in vitro | 1994 |
| Bekier, E.,Wyczolkowska, J.,Szyc, H.,Maslinski, C. | The Inhibitory Effect of Nicotinamide on Asthma-Like Symptoms and Eosinophilia in Guinea-Pigs Anaphylactic Mast Cell De Granulation in Mice and Histamine Release from Rat Isolated Peritoneal Mast Cells by Compound 48-80 | 1974 |
| Bekki, K.,Uchiyama, S.,Ohta, K.,Inaba, Y.,Nakagome, H.,Kunugita, N. | Carbonyl compounds generated from electronic cigarettes | 2014 |
| Belanger, K.,Gent, J. F.,Triche, E. W.,Bracken, M. B.,Leaderer, B. P. | Association of indoor nitrogen dioxide exposure with respiratory symptoms in children with asthma | 2005 |
| Belger, A. L.,Blehm, K. D.,Buchan, R. M. | The determination of ambient air quality within an environmental care unit | 1987 |
| Belin, L.,Wass, U.,Audunsson, G.,Mathiasson, L. | Amines: possible causative agents in the development of bronchial hyperreactivity in workers manufacturing polyurethanes from isocyanates | 1983 |
| Feron,,V, J.,Arts,,J. H, E.,Kuper,,C, F.,Slootweg,,P, J.,Woutersen,,R, A. | Health risks associated with inhaled nasal toxicants | 2001 |
| Bell, I. R.,Schwartz, G. E.,Boyer, N. N.,Koithan, M.,Brooks, A. J. | Advances in integrative nanomedicine for improving infectious disease treatment in public health | 2013 |
| Finberg,,L, | The susceptibility of the fetus and child to chemical pollutants. Interaction of the chemical environment with the infant and young child | 1974 |
| Bello, D.,Herrick, C. A.,Smith, T. J.,Woskie, S. R.,Streicher, R. P.,Cullen, M. R.,Liu, Y. C.,Redlich, C. A. | Skin exposure to isocyanates: Reasons for concern | 2007 |
| Fischer,,P, H.,Kriz,,B,,Martuzzi,,M,,Wojtyniak,,B,,Lebret,,E,,van, Reeuwijk,H,,Pikhart,,H,,Briggs,,D,,Gorynski,,P,,Elliott,,P, | Risk factors indoors and prevalences of childhood respiratory health in four countries in Western and Central Europe | 1998 |
| Fishwick,,D,,Barraclough,,R,,Pickering,,T,,Fletcher,,A,,Lewis,,R,,Niven,,R,,Warburton,,C, J. | Comparison of various airflow measurements in symptomatic textile workers | 2010 |
| Fishwick,,D,,Fletcher,,A, M.,Pickering,,C, A.,Mc,,L. Niven R,Faragher,,E, B. | Lung function in Lancashire cotton and man made fibre spinning mill operatives | 1996 |
| Bencivenga, M.,Capasso, M.,Capristo, C.,Del Giudice, M. M., Jr.,Salvestrini, S.,Capasso, S. | Contribution of air-proof doors and windows to asthma in Campania Plain (Italy) | 2004 |
| Franklin,,P, J. | Indoor air quality and respiratory health of children | 2007 |
| Bentley, S.,Tjin, G.,Jaffarj,,Jones, M.,Richeldi, L.,Burgess, J. | Tissue levels of fibulin-1 and periostin do not differ between patients with idiopathic pulmonary fibrosis and chronic hypersensitivity pneumonitis | 2015 |
| Berend, N.,Skoog, C.,Waszkiewicz, L.,Thurlbeck, W. M. | Maximum volumes in excised human lungs: effects of age, emphysema, and formalin inflation | 1980 |
| Frisk,,M,,Magnuson,,A,,Kiviloog,,J,,Ivarsson,,A, B.,Kamwendo,,K, | Increased occurrence of respiratory symptoms is associated with indoor climate risk indicators - a cross-sectional study in a Swedish population | 2007 |
| Berlivet, S.,Moussette, S.,Ouimet, M.,Verlaan, D. J.,Koka, V.,Al Tuwaijri, A.,Kwan, T.,Sinnett, D.,Pastinen, T.,Naumova, A. K. | Interaction between genetic and epigenetic variation defines gene expression patterns at the asthma-associated locus 17q12-q21 in lymphoblastoid cell lines | 2012 |
| Frisk,,M, L.,Stridh,,G,,Ivarsson,,A, B.,Kamwendo,,K, | Can a housing environmental index establish associations between indoor risk indicators and clinical tests in persons with asthma | 2009 |
| Bernedo, N.,Garcia, M.,Gastaminza, G.,Fernandez, E.,Bartolome, B.,Algorta, J.,Munoz, D. | Allergy to laxative compound (Plantago ovata seed) among health care professionals | 2008 |
| Bernstein, D. I. | OCCUPATIONAL ASTHMA | 1992 |
| Bernstein, D. I. | Allergic reactions to workplace allergens | 1997 |
| Bernstein, J. A.,Bernstein, I. L. | CLINICAL ASPECTS OF RESPIRATORY HYPERSENSITIVITY TO CHEMICALS | 7817 |
| Bernstein, J. M.,Gorfien, J.,Noble, B.,Yankaskas,, Jr. | Nasal polyposis: Immunohistochemistry and bioelectrical findings (a hypothesis for the development of nasal polyps) | 1997 |
| Gallicchio,,L,,Miller,,S, R.,Greene,,T,,Zacur,,H,,Flaws,,J, A. | Adverse health outcomes among cosmetologists and noncosmetologists in the Reproductive Outcomes of Salon Employees (ROSE) study | 2010 |
| Gannon,,P. F, G.,Bright,,P,,Campbell,,M,,O'Hickey,,S, P.,Burge,,P, S. | Occupational asthma due to glutaraldehyde and formaldehyde in endoscopy and x-ray departments | 1995 |
| Gannon,,P. F, G.,Bright,,P,,Campbell,,M,,Ohickey,,S, P.,Burge,,P, S. | Occupational Asthma Due to Glutaraldehyde and Formaldehyde in Endoscopy and X-Ray Departments | 1995 |
| Bessot, J. C. | Protection by Drugs and Physical Protection in Work-Related Asthma | 1988 |
| Garrett,,M, H.,Hooper,,M, A.,Hooper,,B, M.,Abramson,,M, J. | Respiratory symptoms in children and indoor exposure to nitrogen dioxide and gas stoves | 1998 |
| Beuerlein, K.,Ruth, P.,Westermann, B.,Lohr, S.,Schipp, R. | Hemocyanin and the branchial heart complex of Sepia officinalis: are the hemocytes involved in hemocyanin metabolism of coleoid cephalopods? | 2002 |
| Bhardwaj, R.,Ducatman, A.,Finkel, M. S.,Petsonk, E.,Hunt, J.,Beto, R. J. | Chronic pulmonary dysfunction following acute inhalation of butyl acrylate | 2013 |
| Gehring,,U,,Bischof,,W,,Fahlbusch,,B,,Wichmann,,H, E.,Heinrich,,J, | House dust endotoxin and allergic sensitization in children | 2002 |
| Ghasemkhani,,M,,Firoozbakhsh,,S,,Azam,,K,,Ghardashi,,F, | Cotton dust exposure, respiratory symptoms and PEFR in textile workers | 2006 |
| Bhullar, J. S.,Gayagoy, J.,Chaudhary, S.,Kolachalam, R. B. | Delayed presentation of a bowel bovie injury after laparoscopic ventral hernia repair | 2013 |
| Biagini, R. E.,Moorman, W. J.,Knecht, E. A.,Clark, J. C.,Bernstein, I. L. | Acute airway narrowing in monkeys from challenge with 2.5 ppm formaldehyde generated from formalin | 1989 |
| Golshan,,M,,Faghihi,,M,,Marandi,,M, M. | Indoor women jobs and pulmonary risks in rural areas of Isfahan, Iran, 2000 | 2002 |
| Gonzalez, Garcia,M,,Caballero,,A,,Jaramillo,,C,,Maldonado,,D,,Torres, Duque,C, A. | Prevalence, risk factors and underdiagnosis of asthma and wheezing in adults 40 years and older: A population-based study | 2015 |
| Birukova, Anna A.,Wu, Tinghuai,Tian, Yufeng,Meliton, Angelo,Sarich, Nicolene,Tian, Xinyong,Leff, Alan,Birukov, Konstantin G. | Iloprost improves endothelial barrier function in lipopolysaccharide-induced lung injury | 2013 |
| Bischof, W.,Koch, A.,Gehring, U.,Fahlbusch, B.,Wichmann, H. E.,Heinrich, J. | Predictors of high endotoxin concentrations in the settled dust of German homes | 2002 |
| Bjorkman, S. T.,Foster, K. A.,O'Driscoll S, M.,Healy, G. N.,Lingwood, B. E.,Burke, C.,Colditz, P. B. | Hypoxic/Ischemic models in newborn piglet: comparison of constant FiO2 versus variable FiO2 delivery | 2006 |
| Bjorksten, B. | RISK FACTORS IN EARLY CHILDHOOD FOR THE DEVELOPMENT OF ATOPIC DISEASES | 1994 |
| Green,,D, A. | Lung function changes in teenage carpet weavers | 2003 |
| Black, Michael B.,Dodd, Darol E.,McMullen, Patrick D.,Pendse, Salil,MacGregor, Judith A.,Gollapudi, B. Bhaskar,Andersen, Melvin E. | Using gene expression profiling to evaluate cellular responses in mouse lungs exposed to V2O5 and a group of other mouse lung tumorigens and non-tumorigens | 2015 |
| Blackwell, M.,Kang, H.,Thomas, A.,Infante, P. | Formaldehyde: evidence of carcinogenicity | 1981 |
| Greenwald,,R,,Fitzpatrick,,A, M.,Gaston,,B,,Marozkina,,N, V.,Erzurum,,S,,Teague,,W, G. | Breath formate is a marker of airway S-nitrosothiol depletion in severe asthma | 2010 |
| Blanc, V. F.,Tremblay, N. A. | The complications of tracheal intubation: a new classification with a review of the literature | 1974 |
| Grimard,,M,,Adams,,F, | The respiratory health of Quebec textile workers | 1981 |
| Gripenback,,S,,Lundgren,,L,,Eklund,,A,,Liden,,C,,Skare,,L,,Tornling,,G,,Grunewald,,J, | Accumulation of eosinophils and T-lymphocytes in the lungs after exposure to pinewood dust | 2005 |
| Bockelbrink, A. G. | Potential and limitations of long term ventilation | 2006 |
| Guo,,H,,Kwok,,N, H.,Cheng,,H, R.,Lee,,S, C.,Hung,,W, T.,Li,,Y, S. | Formaldehyde and volatile organic compounds in Hong Kong homes: Concentrations and impact factors | 2009 |
| Bodner, Kenneth M.,Burns, Carol J.,Randolph, Nancy M.,Salazar, Eugenio J. | A longitudinal study of respiratory health of toluene diisocyanate production workers | 2001 |
| Hagerhed, Engman,L,,Sigsgaard,,T,,Samuelson,,I,,Sundell,,J,,Janson,,S,,Bornehag,,C, G. | Low home ventilation rate in combination with moldy odor from the building structure increase the risk for allergic symptoms in children | 2009 |
| Bogdanffy, M. S.,Morgan, P. H.,Starr, T. B.,Morgan, K. T. | Binding of Formaldehyde to Human and Rat Nasal Mucus and Bovine Serum Albumin | 1987 |
| Bogdanovic, J.,de Pater, A. J.,Doekes, G.,Wouters, I. M.,Heederik, D. J. | Application of porous foams for size-selective measurements of airborne wheat allergen | 2005 |
| Halken,,S, | Environmental causes of asthma in children | 1994 |
| Hanchette,,C,,Lee,,J, H.,Aldrich,,T, E. | Asthma, Air Quality and Environmental Justice in Louisville, Kentucky | 2011 |
| Hansell,,A, L.,Rose,,N,,Cowie,,C, T.,Belousova,,E, G.,Bakolis,,I,,Ng,,K,,Toelle,,B, G.,Marks,,G, B. | Weighted Road Density and Allergic Disease in Children at High Risk of Developing Asthma | 2014 |
| Harvey,,G, R. | Occupational asthma due to methyl methacrylate bone cement | 1986 |
| Bonisch, U.,Bohme, A.,Kohajda, T.,Mogel, I.,Schutze, N.,von Bergen, M.,Simon, J. C.,Lehmann, I.,Polte, T. | Volatile organic compounds enhance allergic airway inflammation in an experimental mouse model | 2012 |
| Bono, R.,Bellisario, V.,Romanazzi, V.,Pirro, V.,Piccioni, P.,Pazzi, M.,Bugiani, M.,Vincenti, M. | Oxidative stress in adolescent passive smokers living in urban and rural environments | 2014 |
| Haverinen, Shaughnessy,U,,Pekkanen,,J,,Hyvarinen,,A,,Nevalainen,,A,,Putus,,T,,Korppi,,M,,Moschandreas,,D, | Children's homes--determinants of moisture damage and asthma in Finnish residences | 2006 |
| Borchers, M. T.,Crosby, J.,Farmer, S.,Sypek, J.,Ansay, T.,Lee, N. A.,Lee, J. J. | Blockade of CD49d inhibits allergic airway pathologies independent of effects on leukocyte recruitment | 2001 |
| Borisenko, N. F.,Raiko, I. E. | HYGIENIC ASPECTS OF OCCUPATIONAL ALLERGIES OF CHEMICAL ORIGIN | 1980 |
| Hayes,,G, B.,Ye,,T, T.,Lu,,P, L.,Dai,,H, L.,Christiani,,D, C. | Respiratory disease in cotton textile workers: epidemiologic assessment of small airway function | 1994 |
| Heard,,B, E. | Low concentrations of formaldehyde in bronchial asthma | 1986 |
| Heikkila,,P,,Martikainen,,R,,Kurppa,,K,,Husgafvel, Pursiainen,K,,Karjalainen,,A, | Asthma incidence in wood-processing industries in Finland in a register-based population study | 2008 |
| Bornholdt, J.,Saber, A. T.,Sharma, A. K.,Savolainen, K.,Vogel, U.,Wallin, H. | Inflammatory response and genotoxicity of seven wood dusts in the human epithelial cell line A549 | 2007 |
| Hendrick,,D, J.,Lane,,D, J. | Formalin asthma in hospital staff | 1975 |
| Herbert,,F, A.,Hessel,,P, A.,Melenka,,L, S.,Yoshida,,K,,Nakaza,,M, | Respiratory consequences of exposure to wood dust and formaldehyde of workers manufacturing oriented strand board | 1994 |
| Herbert,,F, A.,Hessel,,P, A.,Melenka,,L, S.,Yoshida,,K,,Nakaza,,M, | Pulmonary effects of simultaneous exposures to MDI formaldehyde and wood dust on workers in an oriented strand board plant | 1995 |
| Higashi,,T,,Toyama,,T,,Sakurai,,H,,Nakaza,,M,,Omae,,K,,Nakadate,,T,,Yamaguchi,,N, | Cross-sectional study of respiratory symptoms and pulmonary functions in rayon textile workers with special reference to H2S exposure | 1983 |
| Boulet, L. P.,Bowie, D. | Acute occupational respiratory diseases | 1999 |
| Hollund,,B, E.,Moen,,B, E.,Lygre,,S, H.,Florvaag,,E,,Omenaas,,E, | Prevalence of airway symptoms among hairdressers in Bergen, Norway | 2001 |
| Holme,,J,,Hagerhed, Engman,L,,Mattsson,,J,,Sundell,,J,,Bornehag,,C, G. | Culturable mold in indoor air and its association with moisture-related problems and asthma and allergy among Swedish children | 2010 |
| Holmstrom,,M,,Granstrand,,P,,Nylanderfrench,,L, A.,Rosen,,G, | Upper Airway Symptoms and Function in Wood Surface Coating Industry Workers | 1995 |
| Holmstrom,,M,,Rosen,,G,,Wilhelmsson,,B, | Symptoms, Airway Physiology and Histology of Workers Exposed to Medium-Density Fiber Board | 1991 |
| Holness,,D, L.,Sass, Kortsak,A, M.,Pilger,,C, W.,Nethercott,,J, R. | Respiratory function and exposure-effect relationships in wood dust-exposed and control workers | 1985 |
| Holness,,D, L.,Taraschuk,,I, G.,Pelmear,,P, L. | Effect of dust exposure in Ontario cotton textile mills | 1983 |
| Hong,,C, Y.,Ng,,T, P.,Wong,,M, L.,Koh,,K, T.,Goh,,L, G.,Ling,,S, L. | Lifestyle and behavioural risk factors associated with asthma morbidity in adults | 1994 |
| Howden, Chapman,P,,Pierse,,N,,Nicholls,,S,,Gillespie, Bennett,J,,Viggers,,H,,Cunningham,,M,,Phipps,,R,,Boulic,,M,,Fjallstrom,,P,,Free,,S,,Chapman,,R,,Lloyd,,B,,Wickens,,K,,Shields,,D,,Baker,,M,,Cunningham,,C,,Woodward,,A,,Bullen,,C,,Crane,,J, | Effects of improved home heating on asthma in community dwelling children: randomised controlled trial | 2008 |
| Howieson,,S, | Are our homes making us ill? The impact of energy efficiency on indoor air quality | 2014 |
| Hsu,,J,,Chaves,,S,,Iwane,,M,,Qiu,,Y,,Schaffer,,C,,Alvarez, Reeves,M,,Reponen,,T,,Adamkiewicz,,G,,Ryan,,P,,Brugge,,D,,Turcotte,,D,,Yip,,F,,Sircar,,K,,Blount,,B,,Alwis,,J, U.,Sosnoff,,C,,Ashley,,P,,Hedman,,C,,Strebel,,S,,Behbod,,B,,Lozier,,M,,Chew,,G, L. | Associations among volatile organic compounds, allergic sensitization, and respiratory illness in children with asthma enrolled in a study of low-income, green eco-friendly housing (the green housing study) | 2014 |
| Brandt, Eric,Kovacic, Melinda Butsch,Lee, Gerald,Gibson, Aaron,Acciani, Thomas,LeCras, Timothy,Budelsky, Alison,Hershey, Gurjit Khurana | Diesel exhaust particle induction of IL17A contributes to severe asthma (P6373) | 2013 |
| Huang,,Y. C, T.,Tsuang,,W, | Health effects associated with faulty application of spray polyurethane foam in residential homes | 2014 |
| Brandt, E. B.,Myers, J. M. B.,Acciani, T. H.,Ryan, P. H.,Sivaprasad, U.,Ruff, B.,LeMasters, G. K.,Bernstein, D. I.,Lockey, J. E.,LeCras, T. D.,Hershey, G. K. K. | Exposure to allergen and diesel exhaust particles potentiates secondary allergen-specific memory responses, promoting asthma susceptibility | 2015 |
| Branis, M.,Vetvicka, J. | PM10, Ambient Temperature and Relative Humidity during the XXIX Summer Olympic Games in Beijing: Were the Athletes at Risk? | 2010 |
| Bratthauer, G. L.,Fanning, T. G. | Active Line-1 Retrotransposons in Human Testicular Cancer | 1992 |
| Hulin,,M,,Moularat,,S,,Kirchner,,S,,Robine,,E,,Mandin,,C,,Annesi, Maesano,I, | Positive associations between respiratory outcomes and fungal index in rural inhabitants of a representative sample of French dwellings | 2013 |
| Bray, P. G. | Epoxy resins | 1999 |
| Hunter,,P, R.,Davies,,M, A.,Hill,,K,,Whittaker,,M,,Sufi,,F, | The prevalence of self-reported symptoms of respiratory disease and community belief about the severity of pollution from various sources | 2003 |
| Huss,,K,,Naumann,,P, L.,Mason,,P, J.,Nanda,,J, P.,Huss,,R, W.,Smith,,C, M.,Hamilton,,R, G. | Asthma severity, atopic status, allergen exposure and quality of life in elderly persons | 2001 |
| Hwang,,G, S.,Choi,,J, W.,Yoo,,Y,,Choung,,J, T.,Yoon,,C, S. | Residential Environmental Risk Factors for Childhood Asthma Prevalence in Metropolitan and Semirural Cities in Korea | 2012 |
| Hyatt,,R, E. | Formaldehyde exposure--a case in point | 1984 |
| Hyland,,M, E. | A two-phase network theory of atopy and asthma causation: a possible solution to the impact of genes, hygiene and air quality | 2001 |
| Broberg, K.,Tinnerberg, H.,Axmon, A.,Warholm, M.,Rannug, A.,Littorin, M. | Influence of genetic factors on toluene diisocyanate-related symptoms: evidence from a cross-sectional study | 2008 |
| Imai,,M,,Yoshida,,K,,Tomita,,Y,,Kasama,,K,,Kitabatake,,M, | A Clinico Epidemiological Investigation of Bronchial Asthma in Yokkaichi Japan | 1982 |
| Broderick, D.,Stassen, L. F. A. | Presentation of a nasopharyngeal palatal pleomorphic salivary adenoma causing airway obstruction in a patient with a Le Fort 1 fracture: A case report.∗ | 2014 |
| Brodlie, M.,McKean, M. C.,Johnson, G. E.,Fisher, A. J.,Corris, P. A.,Lordan, J. L.,Ward, C. | Reticular basement membrane thickening in endstage cystic fibrosis lung disease | 2009 |
| Brody, A. R.,Vallyathan, N. V.,Craighead, J. E. | Use of Scanning Electron Microscopy and X-Ray Energy Spectrometry to Determine the Elemental Content of Inclusions in Human Tissue Lesions | 1978 |
| Innocenti,,A,,Cirla,,A, M.,Pisati,,G,,Mariano,,A, | Cross-reaction between aromatic isocyanates (TDI and MDI): a specific bronchial provocation test study | 1988 |
| Broms, K.,Svardsudd, K.,Sundelin, C.,Norback, D. | A nationwide study of indoor and outdoor environments in allergen avoidance and conventional daycare centers in Sweden | 2006 |
| Brooks, A. C.,Rickards, K. J.,Cunningham, F. M. | CXCL8 attenuates chemoattractant-induced equine neutrophil migration | 2011 |
| Brooks, S. M. | Occupational and Environmental Asthma | 1992 |
| Jaakkola,,J, J.,Hwang,,B, F.,Jaakkola,,N, | Home dampness and molds, parental atopy, and asthma in childhood: a six-year population-based cohort study | 2005 |
| Broome, M.,Villarreal, B. | Differential staining of mast cells with toluidine blue | 2012 |
| Jaakkola,,J, J.,Piipari,,R,,Jaakkola,,M, S. | Occupation and asthma: a population-based incident case-control study | 2003 |
| Jaakkola,,J. J, K.,Ieromnimon,,A,,Jaakkola,,M, S. | Interior surface materials and asthma in adults: A population-based incident case-control study | 2006 |
| Jaakkola,,J. J, K.,Parise,,H,,Kislitsin,,V,,Lebedeva,,N, I.,Spengler,,J, D. | Asthma, wheezing, and allergies in Russian schoolchildren in relation to new surface materials in the home | 2004 |
| Jaakkola,,M, S.,Nordman,,H,,Piipari,,R,,Uitti,,J,,Laitinen,,J,,Karjalainen,,A,,Hahtola,,P,,Jaakkola,,J, J. | Indoor dampness and molds and development of adult-onset asthma: a population-based incident case-control study | 2002 |
| Jacobs,,R, R.,Boehlecke,,B,,van, Hage-Hamsten,M,,Rylander,,R, | Bronchial reactivity, atopy, and airway response to cotton dust | 1993 |
| Jacobsen,,G,,Schlunssen,,V,,Schaumburg,,I,,Sigsgaard,,T, | Increased incidence of respiratory symptoms among female woodworkers exposed to dry wood | 2009 |
| Jacobsen,,G,,Schlunssen,,V,,Schaumburg,,I,,Taudorf,,E,,Sigsgaard,,T, | Longitudinal lung function decline and wood dust exposure in the furniture industry | 2007 |
| Jacobsen,,G,,Schlünsssen,,V,,Schaumburg,,I,,Sigsgaard,,T, | Cross-shift change and subsequent longitidinal changes in FEV1 in a 6 year follow-up study of wood dust exposed workers | 2012 |
| Jacobsen,,G, H.,Schlunssen,,V,,Schaumburg,,I,,Sigsgaard,,T, | Cross-shift and longitudinal changes in FEV1 among wood dust exposed workers | 2012 |
| Jafta,,N,,Batterman,,S, A.,Gqaleni,,N,,Naidoo,,R, N.,Robins,,T, G. | Characterization of allergens and airborne fungi in low and middle-income homes of primary school children in Durban, South Africa | 2012 |
| Brugge, D.,Reisner, E.,Padro-Martinez, L. T.,Zamore, W.,Owusu, E.,Durant, J. L. | In-Home Air Filtration for Improving Cardiovascular Health: Lessons from a CBPR Study in Public Housing | 2013 |
| Jedrychowski,,W,,Maugeri,,U,,Jedrychowska, Bianchi,I,,Mroz,,E, | The effect of house dust mite sensitization on lung size and airway caliber in symptomatic and nonsymptomatic preadolescent children: A community-based study in Poland | 2002 |
| Brunetti, G.,Moscato, G.,Pelissero, G. | Aspecific bronchial hyperresponsivity in a group of workers in a refrigerator factory | 1981 |
| Bryant-Stephens, T. | Asthma disparities in urban environments | 2009 |
| Johnson,,A,,Moira,,C, Y.,MacLean,,L,,Atkins,,E,,Dybuncio,,A,,Cheng,,F,,Enarson,,D, | Respiratory abnormalities among workers in an iron and steel foundry | 1985 |
| Johnson,,M,,Nriagu,,J,,Hammad,,A,,Savoie,,K,,Jamil,,H, | Asthma, Environmental Risk Factors, and Hypertension Among Arab Americans in Metro Detroit | 2010 |
| Budanova, L. F.,Makarova, V. N. | REACTION OF SPECIFIC AGGLOMERATION OF LEUKOCYTES IN OCCUPATIONAL ALLERGIC DISEASES INDUCED BY HEXAVALENT CHROMIUM | 1980 |
| Just,,A, C.,Miller,,R, L.,Perzanowski,,M, S.,Rundle,,A, G.,Chen,,Q,,Jung,,K, H.,Hoepner,,L,,Camann,,D, E.,Calafat,,A, M.,Perera,,F, P.,Whyatt,,R, M. | Vinyl flooring in the home is associated with children's airborne butylbenzyl phthalate and urinary metabolite concentrations | 2015 |
| Kahraman,,H,,Sucakli,,M, H.,Kilic,,T,,Celik,,M,,Koksal,,N,,Ekerbicer,,H, C. | Longitudinal pulmonary functional loss in cotton textile workers: a 5-year follow-up study | 2013 |
| Kakooei,,H,,Shahtaheri,,S, J.,Karbasi,,H, A. | Evaluation of workers' exposure to methylene diphenyl diisocyanate (MDI) in an automobile manufacturing company, Iran | 2006 |
| Kamat,,S, R.,Kamat,,G, R.,Salpekar,,V, Y.,Lobo,,E, | Distinguishing byssinosis from chronic obstructive pulmonary disease. Results of a prospective five-year study of cotton mill workers in India | 1981 |
| Kamran,,A,,Hanif,,S,,Murtaza,,G, | Risk factors of childhood asthma in children attending Lyari General Hospital | 2015 |
| Bullard, Daniel C.,Qin, Lan,Lorenzo, Isabel,Quinlin, William M.,Doyle, Nicholas A.,Bosse, Roland,Vestweber, Dietmar,Doerschuk, Claire M.,Beaudet, Arthur L. | P-Selectin/ICAM-1 Double Mutant Mice: Acute Emigration of Neutrophils into the Peritoneum Is Completely Absent but Is Normal into Pulmonary Alveoli | 1995 |
| Bundy, K. W.,Gent, J. F.,Beckett, W.,Bracken, M. B.,Belanger, K.,Triche, E.,Leaderer, B. P. | Household airborne Penicillium associated with peak expiratory flow variability in asthmatic children | 2009 |
| Burcham, P. C.,Raso, A.,Thompson, C. A. | Intermediate Filament Carbonylation During Acute Acrolein Toxicity in A549 Lung Cells: Functional Consequences, Chaperone Redistribution, and Protection by Bisulfite | 2010 |
| Burge, P. S. | The prevention of occupational asthma | 1983 |
| Burger, J. A.,Peled, A. | CXCR4 antagonists: targeting the microenvironment in leukemia and other cancers | 2009 |
| Burgos, S.,Sigala, F.,Argueta, L.,Iglesias, V. | Child health environment in the context of relocating of camp site families to social housing | 2015 |
| Burns, Alan R.,Simon, Scott I.,Kukielka, Gilbert L.,Rowen, Judith L.,Lu, Huifang,Mendoza, Leonardo H.,Bronw, Evelyn S.,Entman, Mark L.,Smith, C. Wayne | Chemotactic factors stimulate CD18-dependent canine neutrophil adherence and motility on lung fibroblasts | 1996 |
| Kiec, Swierczynska,M,,Swierczynska, Machura,D,,Chomiczewska, Skora,D,,Nowakowska, Swirta,E,,Krecisz,,B, | Occupational allergic and irritant contact dermatitis in workers exposed to polyurethane foam | 2014 |
| Burr, M. L.,Matthews, I. P.,Arthur, R. A.,Watson, H. L.,Gregory, C. J.,Dunstan, F. D.,Palmer, S. R. | Effects on patients with asthma of eradicating visible indoor mould: a randomised controlled trial | 2007 |
| Kilburn,,K, H. | Neurobehavioral and pulmonary impairment in 105 adults with indoor exposure to molds compared to 100 exposed to chemicals | 2009 |
| Kilpelainen,,M,,Koskenvuo,,M,,Helenius,,H,,Terho,,E, | Wood stove heating, asthma and allergies | 2001 |
| Butz, A. M.,Breysse, P.,Rand, C.,Curtin-Brosnan, J.,Eggleston, P.,Diette, G. B.,Williams, D.,Bernert, J. T.,Matsui, E. C. | Household smoking behavior: effects on indoor air quality and health of urban children with asthma | 2010 |
| Kim,,H,,Kim,,Y, D.,Cho,,S, H. | Formaldehyde exposure levels and serum antibodies to formaldehyde-human serum albumin of Korean medical students | 1999 |
| Cai, G. H.,Malarstig, B.,Kumlin, A.,Johansson, I.,Janson, C.,Norback, D. | Fungal DNA and pet allergen levels in Swedish day care centers and associations with building characteristics | 2011 |
| Kim,,Ho, Hyun,Lee,,Jae, Young,Kim,,Hyo, Jin,Lee,,Yong, Won,Kim,,Kwang, Jin,Park,,Ju, Hee,Shin,,Dong, Chun,Lim,,Young, Wook | Impact of Foliage Plant Interventions in Classrooms on Actual Air Quality and Subjective Health Complaints | 2013 |
| Çaklr, L.,Çolakoʇlu, E.,Coşkan, A. S.,Kutsal, O. | Cell block vs. Cytocentrifuge smear for cytologic evaluation with pleural effusion from a dog | 2012 |
| Calder, P. C. | Marine omega-3 fatty acids and inflammatory processes: Effects, mechanisms and clinical relevance | 2015 |
| Calderon, R.,Alba, P.,Mencia, G. | Acne due isocyanates | 2010 |
| Callesen, M.,Beko, G.,Weschler, C. J.,Langer, S.,Brive, L.,Clausen, G.,Toftum, J.,Sigsgaard, T.,Host, A.,Jensen, T. K. | Phthalate metabolites in urine and asthma, allergic rhinoconjunctivitis and atopic dermatitis in preschool children | 2014 |
| Campidelli, C.,Di Tommaso, L.,Zanetti, G. | [Aneurysmal bone cysts of the nasal cavity. Description of a case and review of the literature] | 2003 |
| Koenig,,J, Q.,Pierson,,W, E. | Nasal responses to air pollutants | 1984 |
| Koistinen,,K,,Kotzias,,D,,Kephalopoulos,,S,,Schlitt,,C,,Carrer,,P,,Jantunen,,M,,Kirchner,,S,,McLaughlin,,J,,Molhave,,L,,Fernandes,,E, O.,Seifert,,B, | The INDEX project: executive summary of a European Union project on indoor air pollutants | 2008 |
| Canalis, E.,Petrov, D.,Torres, A. | Dealing with infection after thoracic surgical procedures | 2013 |
| Koltai,,P, J. | Effects of Air-Pollution on the Upper Respiratory-Tract of Children | 1994 |
| Canesi, Laura,Ciacci, Caterina,Fabbri, Rita,Marcomini, Antonio,Pojana, Giulio,Gallo, Gabriella | Bivalve molluscs as a unique target group for nanoparticle toxicity | 2012 |
| Koskinen,,O, M.,Husman,,T, M.,Meklin,,T, M.,Nevalainen,,A, I. | The relationship between moisture or mould observations in houses and the state of health of their occupants | 1999 |
| Kovesi,,T, | Respiratory disease in Canadian First Nations and Inuit children | 2012 |
| Kovesi,,T,,Zaloum,,C,,Stocco,,C,,Fugler,,D,,Dales,,R, E.,Ni,,A,,Barrowman,,N,,Gilbert,,N, L.,Miller,,J, D. | Heat recovery ventilators prevent respiratory disorders in Inuit children | 2009 |
| Kovesi,,T, A.,Cao,,Z,,Osborne,,G,,Egeland,,G, M. | Severe early lower respiratory tract infection is associated with subsequent respiratory morbidity in preschool Inuit children in Nunavut, Canada | 2011 |
| Kraai,,S,,Verhagen,,L, M.,Valladares,,E,,Goecke,,J,,Rasquin,,L,,Colmenares,,P,,Del, Nogal,B,,Hermans,,P, W.,de, Waard,J, H. | High prevalence of asthma symptoms in Warao Amerindian children in Venezuela is significantly associated with open-fire cooking: a cross-sectional observational study | 2013 |
| Caramori, G.,Casolari, P.,Garofano, E.,Contoli, M.,Gnesini, G.,Padovani, A.,Fan Chung, K.,Barnes, P. J.,Adcock, I. M.,Papi, A. | Expression of the phosphorylated myristoylated alanine-rich C kinase substrate (MARCKS) in COPD bronchial glands | 2011 |
| Caramori, G.,Casolari, P.,Giuffrè, S.,Contoli, M.,Marku, B.,Padovani, A.,Marwick, J.,Chung, K. F.,Barnes, P. J.,Adcock, I. M.,Papi, A. | Increased expression of aquaporin 5 in bronchial glands of smokers with or without COPD | 2010 |
| Caravaca, F.,Pizarro, J. L.,Arrobas, M.,Cubero, J. J.,Antona, J. M.,Sanchez, E. | Hypersensitivity Reactions Related to Acetate Dialysate and Cellulose Acetate Membrane | 1987 |
| Krieger,,J, W.,Song,,L,,Takaro,,T, K.,Stout,,J, | Asthma and the home environment of low-income urban children: Preliminary findings from the Seattle-King County healthy homes project | 2000 |
| Krieger,,J, W.,Takaro,,T, K.,Song,,L,,Weaver,,M, | The Seattle-King County Healthy Homes Project: a randomized, controlled trial of a community health worker intervention to decrease exposure to indoor asthma triggers | 2005 |
| Carlson, David J.,Mason, Elwood W. | Pulmonary alveolar proteinosis. Diagnosis of probable case by examination of sputum | 1960 |
| Carlsten, James R.,Resnick, Murray B.,Meitner, Patricia A.,Sabo, Edmond,Carter, Adam,Kethu, Sripathi,Bhattacharya, Baishali | Expression of Eotaxin-2 and chemokine receptor 3 in eosinophilic esophagitis versus reflux esophagitis | 2006 |
| Carrer, P.,Maroni, M.,Alcini, D.,Cavallo, D. | Allergens in indoor air: environmental assessment and health effects | 2001 |
| Kurniawan,,E,,Winariani,,A, | Association between wood dust exposure and peripheral blood eosinophil count and lung function abnormality of wood factory and furniture workers | 2012 |
| Kwok,,C,,Money,,A,,Carder,,M,,Turner,,S,,Agius,,R,,Orton,,D,,Wilkinson,,S, M. | Occupational disease in beauticians reported to the health and occupation research network from 1996 to 2011 | 2013 |
| Carswell, F.,Oliver, J.,Weeks, J. | Do mite avoidance measures affect mite and cat airborne allergens? | 1999 |
| Kyle,,R, G.,Kukanova,,M,,Campbell,,M,,Wolfe,,I,,Powell,,P,,Callery,,P, | Childhood disadvantage and emergency admission rates for common presentations in London: an exploratory analysis | 2011 |
| La Grutta,S,,Ferrante,,G,,Malizia,,V,,Cibella,,F,,Viegi,,G, | Environmental effects on fractional exhaled nitric oxide in allergic children | 2012 |
| Case, M. W.,Williams, R.,Yeatts, K.,Chen, F. L.,Scott, J.,Svendsen, E.,Devlin, R. B. | Evaluation of a direct personal coarse particulate matter monitor | 2008 |
| Lai,,P,,Hang,,J, Q.,Zhang,,F, Y.,Zheng,,B, Y.,Valeri,,L,,Eisen,,E,,Christiani,,D, | Lung function improvement is sustained after work cessation in shanghai cotton and silk textile workers | 2014 |
| Lai,,P, S.,Hang,,J, Q.,Zhang,,F, Y.,Sun,,J,,Zheng,,B, Y.,Washko,,G, R.,Christiani,,D, C. | Cotton textile work is associated with persistent changes in lung density on quantitative CT scans | 2014 |
| Lai,,P, S.,Hang,,J, Q.,Zhang,,F, Y.,Zheng,,B, Y.,Valeri,,L,,Eisen,,E, A.,Christiani,,D, C. | Lung function improvement is sustained after work cessation in shanghai textile workers | 2014 |
| Lai,,P, S.,Lin,,X,,Zhang,,F, Y.,Hang,,J, Q.,Christiani,,D, C. | The effect of occupational cotton dust exposure on time to development of impaired lung function and death in Shanghai textile workers | 2013 |
| Lai,,P, S.,Mehta,,A, J.,Hofmann,,O,,Hide,,W,,Christiani,,D, C. | Genetic predictors of 3 month decline in baseline FEV1 in newly hired cotton textile workers | 2012 |
| Laraqui,,C, H.,Rahhali,,A,,Laraqui,,O,,Tripodi,,D,,Curtes,,J, P.,Verger,,C,,Caubet,,A, | Byssinosis and occupational asthma among cotton dust-exposed workers | 2002 |
| Larsson,,M,,Hagerhed, Engman,L,,Kolarik,,B,,James,,P,,Lundin,,F,,Janson,,S,,Sundell,,J,,Bornehag,,C, G. | PVC--as flooring material--and its association with incident asthma in a Swedish child cohort study | 2010 |
| Castillejo-Lopez, Casimiro,Arias, Wilma Martinez,Baumgartner, Stefan | The fat-like gene of Drosophila is the true orthologue of vertebrate fat cadherins and is involved in the formation of tubular organs | 2004 |
| Lebowitz,,M, D.,O'Rourke,,M, K. | PULMONARY RESPONSES TO ALLERGENS AND POLLUTANTS | 1995 |
| Lebowitz,,M, D.,O'Rourke,,M, K.,Dodge,,R, | The adverse health effects of biological aerosols, other aerosols, and indoor microclimate on asthmatics and nonasthmatics | 1982 |
| Castro-Rodriguez, J. A. | Predictors of asthma in young children in lowand middle income countries | 2014 |
| Lebowitz,,M, D.,Quackenboss,,J, J.,Krzyzanowski,,M,,Orourke,,M, K.,Hayes,,C, | Multipollutant Exposures and Health Responses to Particulate Matter | 1992 |
| Catalli, A. E.,Thomson, J. V.,Babirad, I. M.,Duong, M.,Doyle, T. M.,Howie, K. J.,Newbold, P.,Craggs, R. I.,Foster, M.,Gauvreau, G. M.,O'Byrne, P. M.,Sehmi, R. | Modulation of beta 1-integrins on hemopoietic progenitor cells after allergen challenge in asthmatic subjects | 2008 |
| Lee,,Y, L.,Lin,,Y, C.,Hsiue,,T, R.,Hwang,,B, F.,Guo,,Y, L. | Indoor and outdoor environmental exposures, parental atopy, and physician-diagnosed asthma in Taiwanese schoolchildren | 2003 |
| Leh,,O. L, H.,Ahmad,,S,,Aiyub,,K,,Jani,,Y, M.,Hwa,,T, K. | Urban Air Environmental Health Indicators for Kuala Lumpur City | 2012 |
| Leino,,T,,Tammilehto,,L,,Hytonen,,M,,Sala,,E,,Paakkulainen,,H,,Kanerva,,L, | Occupational skin and respiratory diseases among hairdressers | 1998 |
| Celebi, S.,Caglar, E.,Develioglu, O. N.,Topak, M.,Yalcin, E.,Kulekci, M. | The effect of the duration of merocel in a glove finger on postoperative morbidity | 2013 |
| Lemiere,,C,,Begin,,D,,Forget,,A,,Boulet,,L, P.,Camus,,M,,Gerin,,M, | Occupational exposures of workers with occupational asthma and work-exacerbated asthma | 2011 |
| Cesaroni, G.,Badaloni, C.,Porta, D.,Forastiere, F.,Perucci, C. A. | Comparison between various indices of exposure to traffic-related air pollution and their impact on respiratory health in adults | 2008 |
| Lemiere,,C,,Desjardins,,A,,Cloutier,,Y,,Drolet,,D,,Perrault,,G,,Cartier,,A,,Malo,,J, L. | Occupational asthma due to formaldehyde resin dust with and without reaction to formaldehyde gas | 1995 |
| Lemiere,,Catherine,,Cloutier,,Yves,,Perrault,,Guy,,Drolet,,Daniel,,Cartier,,Andre,,Malo,,Jean, Luc | Closed-circuit apparatus for specific inhalation challenges with an occupational agent, formaldehyde, in vapor form | 1996 |
| Lesage,,F, X.,Salles,,J,,Deschamps,,F, | Self-employment in joinery: an occupational risk facor? | 2014 |
| Leu,,G,,Klingbeil,,E,,Arasi,,S,,Nadeau,,K, | Prenatal allergen exposure influencing asthma immunopathogenesis | 2015 |
| Cha, D. S.,Jeon, H. | Anti-nociceptive activity of methanol extracts from Ligularia stenocephala | 2013 |
| Chailleux, E.,Dupas, D.,Geraut, C.,Moigneteau, C.,Pariente, R. | [Respiratory pathology of isocyanates] | 1983 |
| Levine,,R, J.,DalCorso,,R, D.,Blunden,,P, B.,Battigelli,,M, C. | The effects of occupational exposure on the respiratory health of West Virginia morticians | 1984 |
| Chambers, C.,Sung, H. Y.,Max, W. | Home exposure to secondhand smoke among people living in multiunit housing and single family housing: a study of California adults, 2003-2012 | 2014 |
| Li,,X,,Sundquist,,J,,Sundquist,,K, | Socioeconomic and occupational groups and risk of asthma in Sweden | 2008 |
| Liebhart,,J,,Malolepszy,,J,,Wojtyniak,,B,,Pisiewicz,,K,,Plusa,,T,,Gladysz,,U, | Prevalence and risk factors for asthma in Poland: Results from the PMSEAD study | 2007 |
| Chan-Yeung, M.,Malo, J. L. | Aetiological Agents in Occupational Asthma | 1994 |
| Chand, N.,Hess, F. G.,Nolan, K.,Diamantis, W.,McGee, J.,Sofia, R. D. | Aeroallergen-induced immediate asthmatic responses and late-phase associated pulmonary eosinophilia in the guinea pig: effect of methylprednisolone and mepyramine | 1990 |
| Lin,,Shao,,Munsie,,Jean, Pierre,Hwang,,Syni, An,Fitzgerald,,Edward,,Cayo,,Michael, R. | Childhood asthma hospitalization and residential exposure to state route traffic | 2002 |
| Lindfors,,A,,Wickman,,M,,Hedlin,,G,,Pershagen,,G,,Rietz,,H,,Nordvall,,S, L. | Indoor environmental risk factors in young asthmatics: a case-control study | 1995 |
| Lipinska, Ojrzanowska,A,,Wiszniewska,,M,,Swierczynska, Machura,D,,Wittczak,,T,,Nowakowska, Swirta,E,,Palczynski,,C,,Walusiak, Skorupa,J, | Work-related respiratory symptoms among health centres cleaners: a cross-sectional study | 2014 |
| Liss,,G, M.,Tarlo,,S, M.,Doherty,,J,,Purdham,,J,,Greene,,J,,McCaskell,,L,,Kerr,,M, | Physician diagnosed asthma, respiratory symptoms, and associations with workplace tasks among radiographers in Ontario, Canada | 2003 |
| Liu,,Fan,,Zhao,,Yang,,Liu,,Yu, Qin,Liu,,Yang,,Sun,,Prig,,Huang,,Mei, Meng,Liu,,Yi,,Dong,,Guang, Hui | Asthma and asthma related symptoms in 23,326 Chinese children in relation to indoor and outdoor environmental factors: The Seven Northeastern Cities (SNEC) Study | 2014 |
| Liu,,M, Z. | The health investigation of cotton textile workers in Beijing | 1987 |
| Liu,,W,,Huang,,C,,Hu,,Y,,Zou,,Z, J.,Zhao,,Z, H.,Sundell,,J, | Association of building characteristics, residential heating and ventilation with asthmatic symptoms of preschool children in Shanghai: A cross-sectional study | 2014 |
| Chapelle, H.,Segalen, C. | [Limits of IgE serodiagnosis of occupational respiratory allergies] | 1988 |
| Lofstedt,,Hakan,,Westberg,,Hakan,,Selden,,Anders, I.,Rudblad,,Stig,,Bryngelsson,,Ing, Liss,Ngo,,Yen,,Svartengren,,Magnus, | Nasal and Ocular Effects in Foundry Workers Using the Hot Box Method | 2011 |
| Love,,R, G.,Smith,,T, A.,Gurr,,D,,Soutar,,C, A.,Scarisbrick,,D, A.,Seaton,,A, | Respiratory and allergic symptoms in wool textile workers | 1988 |
| Lu,,P, L.,Christiani,,D, C.,Ye,,T, T.,Shi,,N, Y.,Gong,,Z, C.,Dai,,H, I.,Zhang,,W, D.,Huang,,J, W.,Liu,,M, Z. | The study of byssinosis in China: a comprehensive report | 1987 |
| Chau, J. P. C.,Lee, D. T. F.,Yu, D. S. F.,Chow, A. Y. M.,Yu, W. C.,Chair, S. Y.,Lai, A. S. F.,Chick, Y. L. | A feasibility study to investigate the acceptability and potential effectiveness of a telecare service for older people with chronic obstructive pulmonary disease | 2012 |
| Maikala,,R, V.,Dempsey,,P, G.,Ciriello,,V, M.,O'Brien,,N, V. | Dynamic pushing on three frictional surfaces: maximum acceptable forces, cardiopulmonary and calf muscle metabolic responses in healthy men | 2009 |
| Chen, Junmei,Whitelock, Jody L.,Morales, Lisa D.,Lopez, Jose A.,Cruz, Miguel A. | The platelet glycoprotein Ib-von Willebrand factor interaction activates the collagen receptor alpha(2)beta(1) and the two adhesive receptors then collaborate to fully activate platelets to form thrombi | 2004 |
| Chen, L.,Chiung, Y.,Cho, H.,Liu, P. | In vitro activation of molecules in signaling pathways about cell proliferation after toluene diisocyanate stimulation | 2012 |
| Chen, Y.,Wong, G. W. K.,Li, J. | Environmental Exposure and Genetic Predisposition as Risk Factors for Asthma in China | 2016 |
| Chen, Y. F.,Li, N.,Jiao, Y. L.,Wei, P.,Zhang, Q. Y.,Rahman, K.,Zheng, H. C.,Qin, L. P. | Antinociceptive activity of petroleum ether fraction from the MeOH extracts of Paederia scandens in mice | 2007 |
| Mandryk,,J,,Alwis,,K, U.,Hocking,,A, D. | Work-related symptoms and dose-response relationships for personal exposures and pulmonary function among woodworkers | 1999 |
| Mansouri,,F,,Pili,,J, P.,Abbasi,,A,,Soltani,,M,,Izadi,,N, | Respiratory problems among cotton textile workers | 2016 |
| Chew, F. T.,Goh, D. Y.,Lee, B. W. | Effects of an acaricide on mite allergen levels in the homes of asthmatic children | 1996 |
| Mariammal,,T,,Amutha, Jaisheeba,A,,Sornaraj,,R, | Work related respiratory symptoms and pulmonary function tests observed among construction and sanitary workers of Thoothukudi | 2012 |
| Chihara, K. | [Surgical and bronchoscopic treatment for COPD] | 2011 |
| Chikhladze, M. | Identifying the specific allergens and monitoring concentration of air-pollution | 2011 |
| Martin,,M, A.,Thomas,,A, M.,Mosnaim,,G,,Greve,,M,,Swider,,S, M.,Rothschild,,S, K. | Home asthma triggers: barriers to asthma control in Chicago Puerto Rican children | 2013 |
| Chisholm, J.,Wise, R. A.,Hansel, N.,Hanes, J.,Neptune, E. R. | Nanoparticle diffusion in sputum as a biomarker of COPD severity | 2013 |
| Chistoserdova, Ludmila,Gomelsky, Larissa,Vorholt, Julia A.,Gomelsky, Mark,Tsygankov, Yuri D.,Lidstrom, Mary E. | Analysis of two formaldehyde oxidation pathways in Methylobacillus flagellatus KT, a ribulose monophosphate cycle methylotroph | 2000 |
| Chitano, Pasquale,Rado, Vanda,Di Stefano, Antonio,Papi, Alberto,Boniotti, Anna,Zancoughi, Gianna,Boschetto, Piera,Romano, Maria,Salmona, Mario,Ciaccia, Adlabeto,Fabbri, Leonardo Michele,Mapp, Cristina Elisabetta | Effect of subchronic in vivo exposure to nitrogen dioxide on lung tissue inflammation, airway microvascular leakage, and in vitro bronchial muscle responsiveness in rats | 1996 |
| Mberikunashe,,J,,Banda,,S,,Chadambuka,,A,,Gombe,,N, T.,Shambira,,G,,Tshimanga,,M,,Matchaba, Hove,R, | Prevalence and risk factors for obstructive respiratory conditions among textile industry workers in Zimbabwe, 2006 | 2010 |
| Chiu, S. M.,Oleinick, N. L. | The Role of DNA Damage and Repair in the Function of Eukaryotic Genes Radiation Induced Single Strand Breaks and Their Rejoining in Chromosomal and Extrachromosomal Ribosomal DNA of Tetrahymena-Pyriformis | 1980 |
| Meadway,,J, | Asthma and atopy in workers with an epoxy adhesive | 1980 |
| Chiung, Y. M.,Kao, Y. Y.,Chen, H. T.,Liu, P. S. | Inhibition by 2,4-toluene diisocyanate of the calcium signaling of neuronal nicotinic acetylcholine receptors in human neuroblastoma SH-SY5Y cells | 2005 |
| Cho, Y.,Kim, H.,Jeong, Y.,Lee, J.,Hwang, Y. | Comparison of serum osteopontin levels in patients with exacerbations and stable chronic obstructive pulmonary disease | 2012 |
| Choi, H.,Schmidbauer, N.,Spengler, J.,Bornehag, C. G. | Sources of propylene glycol and glycol ethers in air at home | 2011 |
| Meo,,S, A. | Lung function in Pakistani wood workers | 2006 |
| Merchant,,J, A.,Halprin,,G, M.,Hudson,,A, R.,Kilburn,,K, H.,McKenzie,,W, N.,Hurst,,D, J.,Bermazohn,,P, | Responses to cotton dust | 1975 |
| Merchant,,J, A.,Lumsden,,J, C.,Kilburn,,K, H.,O'Fallon,,W, M.,Ujda,,J, R.,Germino,,V, H.,Jr,,Hamilton,,J, D. | Dose response studies in cotton textile workers | 1973 |
| Merget,,R,,Bauer,,T,,Kupper,,H, U.,Philippou,,S,,Bauer,,H, D.,Breitstadt,,R,,Bruening,,T, | Health hazards due to the inhalation of amorphous silica | 2002 |
| Christoffers, W. A.,Schuttelaar, M. L. A.,Coenraads, P. J. | Trends and developments in two decades of patch testing | 2013 |
| Miles,,R,,Jacobs,,D, E. | Future Directions in Housing and Public Health: Findings From Europe With Broader Implications for Planners | 2008 |
| Minov,,J,,Karadzinska, Bislimovska,J,,Tutkun,,E,,Vasilevska,,K,,Risteska, Kuc,S,,Stoleski,,S,,Mijakoski,,D, | Chronic obstructive pulmonary disease in never-smoking female workers exposed to cotton dust | 2014 |
| Minov,,J,,Karadzinska, Bislimovska,J,,Vasilevska,,K,,Risteska, Kuc,S,,Stoleski,,S, | Exercise-induced bronchoconstriction in textile and agricultural workers and in bakers | 2007 |
| Mitchell,,J, C.,Chan, Yeung,M, | Contact allergy from Frullania and respiratory allergy from Thuja | 1974 |
| Cil, B. E. | High-flow vascular malformations | 2010 |
| Clark, N. M.,Brown, R. W.,Parker, E.,Robins, T. G.,Remick, D. G.,Philbert, M. A.,Keeler, G. J.,Israel, B. A. | Childhood asthma | 1999 |
| Moitra,,S,,Bandyopadhyay,,A, | Pulmonary candidiasis and respiratory impairments among saw-mill workers | 2013 |
| Molhave,,L,,Bach,,B,,Pedersen,,O, F. | Human reactions to low concentrations of volatile organic compounds | 1986 |
| Mommers,,M,,Jongmans, Liedekerken,A, W.,Derkx,,R,,Dott,,W,,Mertens,,P,,van, Schayck,C, P.,Steup,,A,,Swaen,,G, M.,Ziemer,,B,,Weishoff, Houben,M, | Indoor environment and respiratory symptoms in children living in the Dutch-German borderland | 2005 |
| Monroe,,R, G.,Gamble,,W, J.,La, Farge,C, G.,Gamboa,,R,,Morgan,,C, L.,Rosenthal,,A,,Bullivant,,S, | Myocardial ultrastructure in systole and diastole using ballistic cryofixation | 1968 |
| Montanaro,,A, | Chemically induced nonspecific bronchial hyperresponsiveness | 1997 |
| Morgan,,P, G.,Ong,,S, G. | First report of byssinosis in Hong Kong | 1981 |
| Clausen, G.,Host, A.,Toftum, J.,Beko, G.,Weschler, C.,Callesen, M.,Buhl, S.,Ladegaard, M. B.,Langer, S.,Andersen, B.,Sundell, J.,Bornehag, C. G.,Sigsgaard, T. | Children's health and its association with indoor environments in Danish homes and daycare centres - methods | 2012 |
| Moshammer,,H, | Indoor- and outdoor-generated particles and children with asthma | 2005 |
| Moshammer,,H,,Fletcher,,T,,Heinrich,,J,,Hoek,,G,,Hruba,,F,,Pattenden,,S,,Rudnai,,P,,Slachtova,,H,,Speizer,,F, E.,Zlotkowska,,R,,Neuberger,,M, | Gas cooking is associated with small reductions in lung function in children | 2010 |
| Moshammer,,H,,Neuberger,,M, | Lung function predicts survival in a cohort of asbestos cement workers | 2009 |
| Clement, B.,Zimmermann, M. | Hepatic Microsomal N Demethylation of N Methylbenzamidine N Dealkylation Vs N Oxygenation of Amidines | 1987 |
| Clerico, D. M. | Sources and effects of indoor air pollution, including sick building syndrome | 1996 |
| Cloosterman, S. G.,Hofland, I. D.,Lukassen, H. G.,Wieringa, M. H.,Folgering, HTh,van der Heide, S.,Brunekreef, B.,van Schayck, C. P. | House dust mite avoidance measures improve peak flow and symptoms in patients with allergy but without asthma: a possible delay in the manifestation of clinical asthma? | 1997 |
| Mustapha,,A,,Briggs,,D,,Hansell,,A, | Burden of childhood respiratory illness and indoor air pollution in the niger delta, Southern Nigeria | 2011 |
| Myatt,,T, A.,Minegishi,,T,,Allen,,J, G.,MacIntosh,,D, L. | Control of asthma triggers in indoor air with air cleaners: a modeling analysis | 2008 |
| Nafees,,A, A.,Fatmi,,Z,,Kadir,,M, M.,Sathiakumar,,N, | Pattern and predictors for respiratory illnesses and symptoms and lung function among textile workers in Karachi, Pakistan | 2012 |
| Coats, T. J.,Morgan, B.,Robinson, C.,Biggs, M.,Adnan, A.,Rutty, G. | End-Tidal CO<inf>2</inf> detection during cadaveric ventilation | 2015 |
| Nagoda,,M,,Okpapi,,J, U.,Babashani,,M, | Assessment of respiratory symptoms and lung function among textile workers at Kano Textile Mills, Kano, Nigeria | 2012 |
| Cocalis, J. C.,Rao, C. Y.,Kestenberg, K. A.,Martin, J. E. | NIOSH Health Hazard Evaluation Report: HETA No. 2000-0098-2862. Robinson Run Mine, Shinnston, West Virginia, 2001 | 2005 |
| Cochrane, E. S.,McNamara, S.,Emerson, J. C.,Kuypers, J. M.,Gibson, R. L.,Campbell, A. P. | Self collection of nasal swabs for diagnosis of respiratory viruses in patients with cystic fibrosis: A feasibility study | 2011 |
| Cockcroft, D. W.,Cartier, A.,Jones, G.,Tarlo, S. M.,Dolovich, J.,Hargreave, F. E. | Asthma caused by occupational exposure to a furan-based binder system | 1980 |
| Nemery,,B, | Reactive fallout of World Trade Center dust | 2003 |
| Newhouse,,M, T. | UFFI dust: nonspecific irritant only? | 1982 |
| Nicholls,,A, J.,Platts,,M, M. | Anaphylactoid reactions due to haemodialysis, haemofiltration, or membrane plasma separation | 1982 |
| Cohen, M. R. | FDA committee tackles poor labeling on plastic containers; tracking Kayexalate; orally disintegrating tablets not absorbed in the mouth; supply all units with oral syringes | 2004 |
| Nikic,,Dragana,,Bogdanovic,,Dragan,,Nikolic,,Maja,,Stankovic,,Aleksandra,,Zivkovic,,Nenad,,Djordjevic,,Amelija, | Air quality monitoring in NIS (SERBIA) and health impact assessment | 2009 |
| Nilsson,,L, | The role of air pollutants | 1994 |
| Coiffier, B.,Thieblemont, C.,De Guibert, S.,Dupuis, J.,Ribrag, V.,Bouabdallah, R.,Morschhauser, F.,Cartron, G.,Le Gouill, S.,Casasnovas, O.,Holte, H.,Hatteville, L.,Zilocchi, C.,Oprea, C.,Tilly, H. | Phase II study of anti-CD19 antibody drug conjugate (SAR3419) in combination with rituximab: Clinical activity and safety in patients with relapsed/refractory diffuse large B-cell lymphoma (NCT01470456) | 2013 |
| Coifman, R.,Yang, C. | Novel allergy vaccine delivery system for poison ivy urushiol (PI) and peanut (PN) | 2014 |
| Colaco, B.,Jagana, R.,Colaco, C.,Mittadodla, P.,Rudrappa, M.,Meena, N. K. | Pulmonary artery embolization in life threatening hemoptysis with a Rasmussen aneurysm | 2014 |
| Noonan,,C, W.,Ward,,T, J. | Environmental tobacco smoke, woodstove heating and risk of asthma symptoms | 2007 |
| Collaco, C. R.,Hochman, D. J.,Yngve, M.,Brooks, E. G. | Effects of air pollutants on basophil degranulation | 2004 |
| Norback,,D,,Lampa,,E,,Engvall,,K, | Asthma, allergy and eczema among adults in multifamily houses in Stockholm (3-HE study)--associations with building characteristics, home environment and energy use for heating | 2014 |
| Collins, P. L.,Melero, J. A. | Progress in understanding and controlling respiratory syncytial virus: Still crazy after all these years | 2011 |
| Nordby,,K, C.,Notø,,Eduard,,Skare,,Kjuus, | Prospective monitoring of exposure and lung function among cement production workers - An international study | 2013 |
| Condliffe, A. M.,Chilvers, E. R.,Haslett, C.,Dransfield, I. | Priming differentially regulates neutrophil adhesion molecule expression/function | 1996 |
| North,,C, M.,Valeri,,L,,Zhang,,F, Y.,Lai,,P, S.,Su,,L,,Hang,,J,,Christiani,,D, C. | Cooking fuel type and chronic respiratory symptoms in Shanghai, China: A cross-sectional analysis of an urban, retired occupational cohort | 2015 |
| Conklin, D. A. N. I. E. L. J. O. S. E. P. H. | Novel Treatments of Acrolein-induced Cardiotoxicity | 2013 |
| Conklin, D. A. N. I. E. L. J. O. S. E. P. H. | Novel Treatments of Acrolein-induced Cardiotoxicity | 2014 |
| Nowak,,D,,Heinrich,,J,,Joerres,,R,,Wassmer,,G,,Berger,,J,,Beck,,E,,Boczor,,S,,Claussen,,M,,Wichmann,,H, E.,Magnussen,,H, | Prevalence of respiratory symptoms, bronchial hyperresponsiveness and atopy among adults: West and East Germany | 1996 |
| Constant, S.,Huang, S.,Caulfuty, M.,Bonfante, R.,Monachino, M.,Frauenfelder, R.,Wiszniewski, L. | 28 day repeated dose toxicity test on an in vitro cell model | 2011 |
| Nowicki,,R, W. | Another sticky situation | 2000 |
| Convery, F. R.,Gunn, D. R.,Hughes, J. D.,Martin, W. E. | The relative safety of polymethylmethacrylate. A controlled clinical study of randomly selected patients treated with Charnley and Ring total hip replacements | 1975 |
| Cook, A. J.,Gold, D. R.,Li, Y. | Spatial Cluster Detection for Longitudinal Outcomes Using Administrative Regions | 2013 |
| Cooke, A.,Fergeson, J.,Bulkhi, A.,Casale, T. B. | The Electronic Cigarette: The good, the bad, and the ugly | 2015 |
| Obadia,,M,,Liss,,G, M.,Lou,,W,,Purdham,,J,,Tarlo,,S, M. | Relationships Between Asthma and Work Exposures Among Non-Domestic Cleaners in Ontario | 2009 |
| Cooper, P. J.,Chico, M. E.,Vaca, M.,Amorim, L.,Sandoval, C. A.,Strachan, D. P.,Rodrigues, L. C.,Barreto, M. L. | Effects of maternal geohelminth infections on the development of atopy, eczema and wheeze during the first three years of life: Findings from the ecuavida birth cohort | 2014 |
| Copeland, N. S.,Sharratt, B. S.,Wu, J. Q.,Foltz, R. B.,Dooley, J. H. | A Wood-Strand Material for Wind Erosion Control: Effects on Total Sediment Loss, PM10 Vertical Flux, and PM10 Loss | 2009 |
| Corcoran, T. E.,Chigier, N. | Inertial deposition effects: a study of aerosol mechanics in the trachea using laser Doppler velocimetry and fluorescent dye | 2003 |
| Corey, P. N.,Ashley, M. J.,Chan-Yeung, M. | Racial differences in lung function: search for proportional relationships | 1979 |
| Osman,,E,,Pala,,K, | Occupational exposure to wood dust and health effects on the respiratory system in a minor industrial estate in Bursa, Turkey | 2009 |
| Ostro,,B, D.,Lipsett,,M, J.,Mann,,J, K.,Wiener,,M, B.,Selner,,J, | Indoor air pollution and asthma. Results from a panel study | 1994 |
| Cortjens, B.,De Boer, O. J.,Antonis, A. F. G.,De Jong, R.,Lutter, R.,Van Woensel, J. B. M.,Bem, R. A. | Neutrophil extracellular trap formation in severe respiratory syncytial virus lung infection | 2015 |
| Cosmi, F.,Schiatti, R.,Ignesti, C. | [Diagnostic study of 5 cases of allergic asthma due to sensitization to silk allergens in textile workers from the Prato area] | 1986 |
| Ozkurt,,S,,Kargi,,B, A.,Kavas,,M,,Evyapan,,F,,Kiter,,G,,Baser,,S, | Respiratory symptoms and pulmonary functions of workers employed in Turkish textile dyeing factories | 2012 |
| Costa, D. L.,Amdur, M. O. | Motor Oil Antagonism of the Effects of Sulfur Di Oxide on Pulmonary Function in the Guinea-Pig | 1978 |
| Pala,,M,,Ugolini,,D,,Ceppi,,M,,Rizzo,,F,,Maiorana,,L,,Bolognesi,,C,,Schilio,,T,,Gilli,,G,,Bigatti,,P,,Bono,,R,,Vecchio,,D, | Occupational exposure to formaldehyde and biological monitoring of Research Institute workers | 2008 |
| Costa, Lais Rosa Rodrigues,Goad, Mary Beth P.,Seahorn, Thomas L.,Taylor, Henry W.,Lopez, Mae,Hosgood, Giselle,Moore, Rustin M. | Immunohistochemical localization and distribution of endothelin-1 in lungs of healthy horses and those with summer pasture-associated obstructive pulmonary disease | 2002 |
| Costa, L. R. R.,Seahorn, T. L.,Moore, R. M.,Oliver, J. L.,Hosgood, G. L. | Plasma and bronchoalveolar fluid concentrations of nitric oxide and localization of nitric oxide synthesis in the lungs of horses with summer pasture-associated obstructive pulmonary disease | 2001 |
| Cote, J.,Chan, H.,Brochu, G.,Chan-Yeung, M. | Occupational asthma caused by exposure to neurospora in a plywood factory worker | 1991 |
| Cote, J.,Chan, H.,Brochu, G.,Chanyeung, M. | Occupational Asthma Caused by Exposure to Neurospora in a Plywood Factory-Worker | 1991 |
| Pan,,G, W.,Zhang,,S, J.,Feng,,Y, P.,Takahashi,,K,,Kagawa,,J,,Yu,,L, Z.,Wang,,P,,Liu,,M, J.,Liu,,Q, A.,Hou,,S, W.,Pan,,B, L.,Li,,J, P. | Air pollution and children's respiratory symptoms in six cities of Northern China | 2010 |
| Cousin, G. C. | Accidental application of cyanoacrylate to the mouth | 1990 |
| Parikh,,J, R.,Chatterjee,,B, B.,Rao,,N, M.,Bhagia,,L, J. | The clinical manifestations of byssinosis in Indian textile workers | 1986 |
| Parikh,,J, R.,Majumdar,,P, K.,Shah,,A, R.,Rao,,N, M.,Kashyap,,S, K. | Acute and chronic changes in pulmonary functions among Indian textile workers | 1990 |
| Cox, Barnaby,Hadjantonakis, Anna-Katerina,Collins, Jane E.,Magee, Anthony I. | Cloning and expression throughout mouse development of mFat1, a homologue of the Drosophila tumour suppressor gene fat | 2000 |
| Paudyal,,P,,Semple,,S,,Ayres,,J, | Cross-shift changes in lung function in textile processing workers | 2010 |
| Paudyal,,P,,Semple,,S,,Gairhe,,S,,Steiner,,M. F, C.,Niven,,R,,Ayres,,J, G. | Respiratory symptoms and cross-shift lung function in relation to cotton dust and endotoxin exposure in textile workers in Nepal: A cross-sectional study | 2015 |
| Creely, K. S.,Hughson, G. W.,Cocker, J.,Jones, K. | Assessing isocyanate exposures in polyurethane industry sectors using biological and air monitoring methods | 2006 |
| Criep, Leo H.,Teufel, Romayne A.,Miller, Craig S. | Fungicidal agents in the treatment of allergy to molds | 1958 |
| Peden,,D, B. | Genetic and Environmental Factors in Asthma | 2010 |
| Crippa, M.,Belleri, L.,Mistrello, G.,Tedoldi, C.,Alessio, L. | Prevention of latex allergy among health care workers and in the general population: latex protein content in devices commonly used in hospitals and general practice | 2006 |
| Crocker, D. D.,Kinyota, S.,Dumitru, G. G.,Ligon, C. B.,Herman, E. J.,Ferdinands, J. M.,Hopkins, D. P.,Lawrence, B. M.,Sipe, T. A. | Effectiveness of Home-Based, Multi-Trigger, Multicomponent Interventions with an Environmental Focus for Reducing Asthma Morbidity A Community Guide Systematic Review | 2011 |
| Peters,,J, M.,Murphy,,R, L. | Hazards to health: do it yourself polyurethane foam | 1971 |
| Crosse, K. R.,Bray, J. P.,Orbell, G.,Preston, C. A. | Histological evaluation of the soft palate in dogs affected by brachycephalic obstructive airway syndrome | 2015 |
| Crosse, K. R.,Bray, J. P.,Orbell, G. M. B.,Preston, C. A. | Histological evaluation of the soft palate in dogs affected by brachycephalic obstructive airway syndrome | 2015 |
| Crouch, E.,Parghi, D.,Kuan, S. F.,Persson, A. | Surfactant Protein D Subcellular Localization in Nonciliated Bronchiolar Epithelial Cells | 1992 |
| Pham,,Q, T.,Cavelier,,C,,Mereau,,P,,Mur,,J, M.,Cicolella,,A, | Isocyanates and respiratory function: a study of workers producing polyurethane foam moulding | 1978 |
| Phipatanakul,,W,,Bailey,,A,,Hoffman,,E, B.,Sheehan,,W, J.,Lane,,J, P.,Baxi,,S,,Rao,,D,,Permaul,,P,,Gaffin,,J, M.,Rogers,,C, A.,Muilenberg,,M, L.,Gold,,D, R. | The school inner-city asthma study: design, methods, and lessons learned | 2011 |
| Pierson,,W, E.,Koenig,,J, Q.,Bardana,,E, J.,Jr, | Potential adverse health effects of wood smoke | 1989 |
| Plattsmills,,T. A, E.,Sporik,,R, B.,Chapman,,M, D.,Heymann,,P, W. | The Role of Indoor Allergens in Asthma | 1995 |
| Cummings, K. J.,Fink, J. N.,Vasudev, M.,Piacitelli, C.,Kreiss, K. | Vocal cord dysfunction and other respiratory illnesses in two water-damaged buildings | 2012 |
| Cummins, Philip M. | Occludin: One Protein, Many Forms | 2012 |
| Polyzois,,D,,Polyzoi,,E,,Wells,,J, A.,Koulis,,T, | Poor Indoor Air Quality, Mold Exposure, and Upper Respiratory Tract Infections-Are We Placing Our Children at Risk? | 2016 |
| Ponsonby,,A, L.,Couper,,D,,Dwyer,,T,,Carmichael,,A,,Kemp,,A,,Cochrane,,J, | The relation between infant indoor environment and subsequent asthma | 2000 |
| Porebski,,G,,Wozniak,,M,,Czarnobilska,,E, | Residential proximity to major roadways is associated with increased prevalence of allergic respiratory symptoms in children | 2014 |
| Custovic, A.,Green, R.,Smith, A.,Chapman, M. D.,Woodcock, A. | New mattresses: how fast do they become a significant source of exposure to house dust mite allergens? | 1996 |
| Pourmahabadian,,M,,Azam,,K,,Ghasemkhani,,M, | Pulmonary function study between formaldehyde exposed and non-exposed staffs at some of the Tehran Educational Hospitals | 2006 |
| Pratt,,P, C.,Lynn,,W, S. | Lung disease in cotton textile workers | 1983 |
| Cutz, E.,Yeger, H.,Wong, V.,Bienkowski, E.,Chan, W. | In-Vitro Characteristics of Pulmonary Neuroendocrine Cells Isolated from Rabbit Fetal Lung I. Effects of Culture Media and Nerve Growth Factor | 1985 |
| Czaja, J. M.,McCaffrey, T. V. | Acoustic measurement of subglottic stenosis | 1996 |
| Pross,,H, F.,Day,,J, H.,Clark,,R, H.,Lees,,R, E. | Immunologic studies of subjects with asthma exposed to formaldehyde and urea-formaldehyde foam insulation (UFFI) off products | 1987 |
| D'Arcy, P. F. | Warnings from the Comittee on Safety of Medicines | 1987 |
| Putus,,T,,Tuomainen,,A,,Rautiala,,S, | Chemical and microbial exposures in a school building: adverse health effects in children | 2005 |
| Dabrowski, M. P.,Szmigielski, S.,Szelepin, B. | Immunotropic activity of peat preparation Tolpa (PPT) | 1999 |
| Rabbani,,U,,Nafees,,A, A. | COMPARING LUNG FUNCTION OF TEXTILE WORKERS WITH THE HEALTHY PAKISTANI POPULATION | 2015 |
| Ramani,,S, | Airway obstruction from denture fixative | 2007 |
| Dahlgren, J.,Wardenburg, M.,Peckham, T. | Goodpasture's Syndrome and Silica: A Case Report and Literature Review | 2010 |
| Daisey, J. M.,Angell, W. J. | Survey and critical review of the literature on indoor air quality, ventilation and health symptoms in schools | 1999 |
| Rastogi,,S, K.,Gupta,,B, N.,Mathur,,N,,Husain,,T, | A study of the prevalence of ventilatory obstruction in textile workers exposed to cotton dust | 1989 |
| Rava,,M,,Cazzoletti,,L,,Marcon,,A,,Padovani,,D,,Dall'Acqua,,M,,Bacchi,,S,,Silocchi,,C,,Ricci,,P,,de, Marco,R, | Cause-specific mortality in populations exposed and unexposed to outdoor emissions of formaldehyde and wood dust: the Viadana study | 2009 |
| Dal Negro, R. W.,Iversen, M.,Calverly, P. M. A. | Efficacy and safety of erdosteine in COPD: Results of a 12-month prospective, multinational study | 2015 |
| Dales, R.,Liu, L.,Wheeler, A. J.,Gilbert, N. L. | Quality of indoor residential air and health | 2008 |
| Dales, R.,Raizenne, M. | Residential exposure to volatile organic compounds and asthma | 2004 |
| Dales, R. E.,Kerr, P. E.,Alary, M. | The acute effects of humidifiers on asthma morbidity | 1996 |
| Dales, R. E.,Miller, D.,White, J. | Testing the association between residential fungus and health using ergosterol measures and cough recordings | 1999 |
| Dallas, C. E.,Theiss, J. C.,Harrist, R. B.,Fairchild, E. J. | Respiratory responses in the lower respiratory tract of Sprague-Dawley rats to formaldehyde inhalation | 1986 |
| Richard,,E, E.,Augusta, Chinyere,N, A.,Jeremaiah,,O, S.,Opara,,U. C, A.,Henrieta,,E, M.,Ifunanya,,E, D. | Cement Dust Exposure and Perturbations in Some Elements and Lung and Liver Functions of Cement Factory Workers | 2016 |
| Daneault, S.,Infante-Rivard, C. | HYPOTHESES ON CHANGES IN THE FREQUENCY OF ASTHMA | 1988 |
| Riddervold,,I, S.,Bonlokke,,J, H.,Olin,,A, C.,Gronborg,,T, K.,Schlunssen,,V,,Skogstrand,,K,,Hougaard,,D,,Massling,,A,,Sigsgaard,,T, | Effects of wood smoke particles from wood-burning stoves on the respiratory health of atopic humans | 2012 |
| Dannenberg, A. L.,Jackson, R. J.,Frumkin, H.,Schieber, R. A.,Pratt, M.,Kochtitzky, C.,Tilson, H. H. | The impact of community design and land-use choices on public health: A scientific research agenda | 2003 |
| Dannenberg, C.,Haupt, R.,Mantovani, L.,Skuballa, A.,Korholz, D. | Primary high-grade non-Hodgkin lymphoma of the trachea in an adolescent | 2003 |
| Daugherty, Alan,Kosswig, Ninetta,Cornicelli, Joseph A.,Whitman, Stewart C.,Wolle, Sabine,Rateri, Debra L. | Macrophage-specific expression of class A scavenger receptors enhances granuloma formation in the absence of increased lipid deposition | 2001 |
| Ritchie,,I, M.,Lehnen,,R, G. | Formaldehyde-related health complaints of residents living in mobile and conventional homes | 1987 |
| Davidson, J. M. | Biochemistry and Turnover of Lung Interstitium | 1990 |
| Robertson,,A, S.,Burge,,P, S.,Hedge,,A,,Sims,,J,,Gill,,F, S.,Finnegan,,M,,Pickering,,C. A, C.,Dalton,,G, | Comparison of Health Problems Related to Work and Environmental Measurements in Two Office Buildings with Different Ventilation Systems | 1985 |
| Robins,,T, G.,Haboubi,,G,,Demers,,R, Y.,Schork,,M, A. | Respiratory morbidity of pattern and model makers exposed to wood, plastic, and metal products | 1990 |
| Davis, W. E.,Nishioka, G. J. | Endoscopic partial inferior turbinectomy using a power microcutting instrument | 1996 |
| Daviskas, E.,Anderson, S. D.,Jaques, A.,Charlton, B. | Inhaled Mannitol Improves the Hydration and Surface Properties of Sputum in Patients With Cystic Fibrosis | 2010 |
| Rodriguez,,L, A.,Rey,,J, J.,Herrera,,A, B.,Castro,,H,,Niederbacher,,J,,Vera,,L, M.,Cala,,L, L.,Bolivar,,F, | [Respiratory symptoms associated with asthma prevalence and air pollution in preschool children] | 2010 |
| de Almeida, M. M.,Lopes, I.,Nunes, C. | Characterization of indoor air quality in Portugal - habitar study | 2010 |
| De Biurrun, G.,Muñoz-Barrutia, A.,Heras, G.,Ferrando, A.,Ferreira, M.,Bastarrika, G.,Blanco, D.,Ortiz-De-Solorzano, C.,Montuenga, L. M. | Emphysema promotes lung cancer progression in the A/Jurethane mouse model | 2011 |
| de Blay, F. | Recommendations concerning the study of removal methods for house dust mite allergens | 2000 |
| Ross,,D, J.,Keynes,,H, L.,McDonald,,J, C. | SWORD '97: surveillance of work-related and occupational respiratory disease in the UK | 1999 |
| Rossiter,,E, | Pulmonary function in histology technicians compared with women from Michigan: effects of chronic low dose formaldehyde exposure on a national sample of women | 1990 |
| Rudell,,B,,Wass,,U,,Horstedt,,P,,Levin,,J, O.,Lindahl,,R,,Rannug,,U,,Sunesson,,A, L.,Ostberg,,Y,,Sandstrom,,T, | Efficiency of automotive cabin air filters to reduce acute health effects of diesel exhaust in human subjects | 1999 |
| de Boer, A. H.,Hagedoorn, P.,Gjaltema, D.,Goede, J.,Frijlink, H. W. | Air classifier technology (ACT) in dry powder inhalation - Part 4. Performance of air classifier technology in the Novolizer (R) multi-dose dry powder inhaler | 2006 |
| de Boer, E. M.,Bruynzeel, D. P.,van Ketel, W. G. | Dyshidrotic eczema as an occupational dermatitis in metal workers | 1988 |
| Rumchev,,K,,Spickett,,J,,Bulsara,,M,,Phillips,,M,,Stick,,S, | Association of domestic exposure to volatile organic compounds with asthma in young children | 2004 |
| Saad, Hussein,A,,Thabet,,E, H.,Taha,,M, M.,Shahy,,E, M.,Mahdy, Abdallah,H, | Association of ADAM33 gene polymorphism and arginase activity with susceptibility to ventilatory impairment in wood dust-exposed workers | 2015 |
| De Lorenzi, Davide,Furlanello, Tommaso | What is your diagnosis? Esophageal nodules in a dog | 2010 |
| Sahasrabudhe,,T, R.,Joseph,,T,,Gupta,,A,,Dave,,A, J. | Assessment of respiratory health of building construction workers | 2013 |
| Sahlberg,,B,,Mi,,Y, H.,Norback,,D, | Indoor environment in dwellings, asthma, allergies, and sick building syndrome in the Swedish population: a longitudinal cohort study from 1989 to 1997 | 2009 |
| Salameh,,P,,Karaki,,C,,Awada,,S,,Rachidi,,S,,Al, Hajje,A,,Bawab,,W,,Saleh,,N,,Waked,,M, | Asthma, indoor and outdoor air pollution: A pilot study in Lebanese school teenagers | 2015 |
| Sallis,,R, E. | Pearls | 1999 |
| Deakin, C. D.,McLaren, R. M.,Petley, G. W.,Clewlow, F.,Dalrymple-Hay, M. J. R. | Effects of positive end-expiratory pressure on transthoracic impedance - implications for defibrillation | 1998 |
| Dean, J. H.,House, R. V.,Murray, M. J.,Stillman, W. S.,Irons, R. D.,Steinhagen, W. H.,Phelps, M. C.,Adams, D. O.,Lauer, L. D. | Immune Function and Host Resistance in B-6c-3f-1 Mice Exposed to Formaldehyde | 1984 |
| Dearman, R. J.,Basketter, D. A.,Evans, P.,Kimber, I. | Comparison of cytokine secretion profiles provoked in mice by glutaraldehyde and formaldehyde | 1999 |
| Dearman, R. J.,Humphreys, N.,Skinner, R. A.,Kimber, I. | Allergen-induced cytokine phenotypes in mice: role of CD4 and CD8 T cell populations | 2005 |
| Schei,,M, A.,Hessen,,J, O.,Smith,,K, R.,Bruce,,N,,McCracken,,J,,Lopez,,V, | Childhood asthma and indoor woodsmoke from cooking in Guatemala | 2004 |
| DeCoux, Ashley,Chaplin, Jennifer,Wilson, Glenn,Benjamin, John,Gebb, Sarah | The hyperoxia-mediated decrease in tenascin-C in fetal lung fibroblasts inhibits cell migration | 2012 |
| Delneste, Y.,Jeannin, P.,Gosset, P.,Lassalle, P.,Cardot, E.,Tillie-Leblond, I.,Joseph, M.,Pestel, J.,Tonnel, A. B. | Allergen-stimulated T lymphocytes from allergic patients induce vascular cell adhesion molecule-1 (VCAM-1) expression and IL-6 production by endothelial cells | 1995 |
| Shamssain,,M, H.,Shamsian,,N, | Respiratory symptoms and pulmonary function in a group of women weavers in South Africa | 1997 |
| Demaurex, N.,Downey, G. P.,Waddell, T. K.,Grinstein, S. | Intracellular pH regulation during spreading of human neutrophils | 1996 |
| Shi,,J,,Hang,,J, Q.,Mehta,,A, J.,Zhang,,H, X.,Dai,,H, L.,Su,,L,,Eisen,,E, A.,Christiani,,D, C. | Long-term effects of work cessation on respiratory health of textile workers: a 25-year follow-up study | 2010 |
| Deng, L.,Chen, J.,Long, J.,Duan, Y.,Jiang, X.,Xu, R. | ADAM8 inhibitor peptide reduces inflammation and bronchial hyperresponsivess in Ovalbumin-Sensitized Mice | 2012 |
| Deng, L.,Shi, X.,Zhang, Z. | Numerical modeling of a multilayer-structured bronchial airway vessel and its narrowing behavior | 2015 |
| Deschamps, F.,DeschampsBoulanger, S. | Occupational asthma in 1997 | 1997 |
| Silpasuwan,,P,,Prayomyong,,S,,Sujitrat,,D,,Suwan, Ampai,P, | Cotton Dust Exposure and Resulting Respiratory Disorders Among Home-Based Garment Workers | 2015 |
| Skovsted,,T, A.,Schlunssen,,V,,Schaumburg,,I,,Wang,,P,,Staun, Olsen,P,,Skov,,P, S. | Only few workers exposed to wood dust are detected with specific IgE against pine wood | 2003 |
| Smedje,,G,,Norback,,D, | Incidence of asthma diagnosis and self-reported allergy in relation to the school environment--a four-year follow-up study in schoolchildren | 2001 |
| Smedje,,G,,Norbäck,,D,,Edling,,C, | Asthma among secondary schoolchildren in relation to the school environment | 1997 |
| Smedley,,J, | Is formaldehyde an important cause of allergic respiratory disease? | 1996 |
| Smedley,,J,,Inskip,,H,,Wield,,G,,Coggon,,D, | Work related respiratory symptoms in radiographers | 1996 |
| Sole,,A,,Cordero,,P, J.,Morales,,P,,Martinez,,M, E.,Vera,,F,,Moya,,C, | Epidemic outbreak of interstitial lung disease in aerographics textile workers--the "Ardystil syndrome": a first year follow up | 1996 |
| Dhawan, B. N.,Srimal, R. C. | Anti-inflammatory and some other pharmacological effects of 3,4-trans-2,2-dimethyl-3-phenyl-4-(p-(beta-pyrrolidinoethoxy)-phenyl)-7-methoxy-c hroman (Centchroman) | 1973 |
| Soto-Aguilar, M, C.,Salvaggio,,J, E. | IMMUNOLOGIC ASPECTS OF OCCUPATIONAL ASTHMA | 1991 |
| Spanier,,A, J.,Hornung,,R,,Lierl,,M,,Lanphear,,B, P. | Environmental exposures and exhaled nitric oxide in children with asthma | 2006 |
| Di Michele, R.,Di Renzo, A. M.,Ammazzalorso, S.,Merni, F. | Comparison of physiological responses to an incremental running test on treadmill, natural grass, and synthetic turf in young soccer players | 2009 |
| Spengler,,J, D.,Jaakkola,,J, J.,Parise,,H,,Katsnelson,,B, A.,Privalova,,L, I.,Kosheleva,,A, A. | Housing characteristics and children's respiratory health in the Russian Federation | 2004 |
| DiBardino, D. M.,Ginsburg, M. E.,Whippo, B.,Jellen, P. A.,Thomashow, B.,Maxfield, R.,Bulman, W. A. | Long term outcomes in the post-national emphysema treatment trial era: Surgical Vs. bronchoscopic lung volume reduction | 2015 |
| Dick, S.,Doust, E.,Cowie, H.,Ayres, J. G.,Turner, S. | Associations between environmental exposures and asthma control and exacerbations in young children: A systematic review | 2014 |
| Sripaiboonkij,,P,,Sripaiboonkij,,N,,Phanprasit,,W,,Jaakkola,,M, S. | Respiratory and skin health among glass microfiber production workers: a cross-sectional study | 2009 |
| Dickerman, R. M.,Dunn, E. L.,White, M. G. | The impact of an air evacuation system on cadaver kidney retrieval | 1986 |
| Stewart,,L,,Watson,,A. F, R.,Gee,,I, L.,Fletcher,,G, D.,Niven,,R. M, L.,Frank,,P, | Monitoring internal air quality within a community of known respiratory health status | 2000 |
| Stieb,,D, M.,Burnett,,R, T.,Beveridge,,R, C.,Brook,,J, R. | Association between ozone and asthma emergency department visits in Saint John, New Brunswick, Canada | 1996 |
| Subramanian,,P,,Reynolds,,S,,Breuer,,G,,Whitten,,P, | Analysis of limonene as an indicator for the assessment of environmental air-quality and health symptoms in office buildings | 2000 |
| Suglia,,S, F.,Chambers,,E, C.,Rosario,,A,,Duarte,,C, S. | Asthma and obesity in three-year-old urban children: role of sex and home environment | 2011 |
| Dimas, K. S.,Pantazis, P.,Ramanujam, R. | Chios Mastic Gum: A Plant-produced Resin Exhibiting Numerous Diverse Pharmaceutical and Biomedical Properties | 2012 |
| Ding, H.,Karunanithi, M.,Kanagasingam, Y.,Vignarajan, J.,Moodley, Y. | A pilot study of a mobile-phone-based home monitoring system to assist in remote interventions in cases of acute exacerbation of COPD | 2014 |
| Sun,,Y, X.,Sundell,,J, | On Associations between Housing Characteristics, Dampness and Asthma and Allergies among Children in Northeast Texas | 2013 |
| Ding, Yunfei,Tong, Min,Liu, Shuqian,Moscow, Jeffrey A.,Tai, Hsin-Hsiung | NAD+-linked 15-hydroxyprostaglandin dehydrogenase (15-PGDH) behaves as a tumor suppressor in lung cancer | 2005 |
| Suuronen,,K,,Aalto, Korte,K,,Piipari,,R,,Tuomi,,T,,Jolanki,,R, | Occupational dermatitis and allergic respiratory diseases in Finnish metalworking machinists | 2007 |
| Takaro,,T, K.,Scott,,J, A.,Allen,,R, W.,Anand,,S, S.,Becker,,A, B.,Befus,,A, D.,Brauer,,M,,Duncan,,J,,Lefebyre,,D, L.,Lou,,W,,Mandhane,,P, J.,McLean,,K, E.,Miller,,G,,Sbihi,,H,,Shu,,H,,Subbarao,,P,,Turvey,,S, E.,Wheeler,,A, J.,Zeng,,L, L.,Sears,,M, R.,Brook,,J, R. | The Canadian Healthy Infant Longitudinal Development (CHILD) birth cohort study: assessment of environmental exposures | 2015 |
| Takeda,,M,,Saijo,,Y,,Yuasa,,M,,Kanazawa,,A,,Araki,,A,,Kishi,,R, | Relationship between sick building syndrome and indoor environmental factors in newly built Japanese dwellings | 2009 |
| Talini,,D,,Monteverdi,,A,,Benvenuti,,A,,Petrozzino,,M,,Di, Pede,F,,Lemmi,,M,,Carletti,,A,,Macchioni,,P,,Serretti,,N,,Viegi,,G,,Paggiaro,,P, | Asthma-like symptoms, atopy, and bronchial responsiveness in furniture workers | 1998 |
| Dohle, Carolin Isabel,Richerson, George Bradley | Lack of smooth muscle in arteries next to serotonergic neurons: Reduction of the diffusion barrier for CO2 in areas of central chemoreceptors | 2005 |
| Domenec Llavallol, O. | Exploration Diagnosis and Treatment of the Eustachian Tube Diseases by Means of a Positive Pressure through the External Meatus and Drum Perforations or Paracentesis with Grommet Drain | 1989 |
| Tanzil,,S,,Nafees,,A, A. | Low prevalence of asthma among textile workers in Karachi, Pakistan | 2015 |
| Donaldson, S. H.,Bennett, W. D. | Requirements and strategies for hydration therapies in cf patients | 2010 |
| Tominaga,,S,,Ono,,M, | A plan of the comprehensive study on indoor pollution and its health effects by the Aichi Prefecture, Japan | 1985 |
| Tortolero,,S, R.,Bartholomew,,L, K.,Tyrrell,,S,,Abramson,,S, L.,Sockrider,,M, M.,Markham,,C, M.,Whitehead,,L, W.,Parcel,,G, S. | Environmental allergens and irritants in schools: a focus on asthma | 2002 |
| Dore, A. I.,Santana-Lemos, B. A. A.,Coser, V. M.,Santos, F. L. S.,Dalmazzo, L. F.,Lima, A. S. G.,Jacomo, R. H.,Elias, J.,Falcao, R. P.,Pereira, W. V.,Rego, E. M. | The association of ICAM-1 Exon 6 (E469K) but not of ICAM-1 Exon 4 (G241R) and PECAM-1 Exon 3 (L125V) polymorphisms with the development of differentiation syndrome in acute promyelocytic leukemia | 2007 |
| Tsai,,C, H.,Tung,,K, Y.,Su,,M, W.,Chiang,,B, L.,Chew,,F, T.,Kuo,,N, W.,Lee,,Y, L. | Interleukin-13 genetic variants, household carpet use and childhood asthma | 2013 |
| Tsai,,H, J.,Tsai,,A, C.,Nriagu,,J,,Ghosh,,D,,Gong,,M,,Sandretto,,A, | Risk factors for respiratory symptoms and asthma in the residential environment of 5th grade schoolchildren in Taipei, Taiwan | 2006 |
| Tsuang,,W,,Huang,,Y, C. | Asthma induced by exposure to spray polyurethane foam insulation in a residential home | 2012 |
| dos Santos Franco, Adriana Lino,Dorningos, Helori Vanni,Damazo, Amilcar Sabino,Breithaupt-Faloppa, Ana Cristina,Ligeiro de Oliveira, Ana Paula,Pereira Costa, Soraia Katia,Oliani, Sonia Maria,Oliveira-Filho, Ricardo Martins,Vargaftig, B. Boris,Tavares-de-Lima, Wothan | Reduced allergic lung inflammation in rats following formaldehyde exposure: Long-term effects on multiple effector systems | 2009 |
| Tuomainen,,A,,Seuri,,M,,Sieppi,,A, | Indoor air quality and health problems associated with damp floor coverings | 2003 |
| Douglas, W. W.,Kagayama, M. | Calcium and Stimulus Secretion Coupling in the Mast Cell Stimulant and Inhibitory Effects of Calcium-Rich Media on Exocytosis | 1977 |
| Douwes, J.,Gibson, P.,Pekkanen, J.,Pearce, N. | Non-eosinophilic asthma: importance and possible mechanisms | 2002 |
| Drachev, A. L.,Drachev, L. A.,Kaulen, A. D.,Khitrina, L. V. | The Action of Lanthanum Ions and Formaldehyde on the Proton Pumping Function of Bacterio Rhod Opsin | 1984 |
| Dransfield, I.,Stocks, S. C.,Haslett, C. | Regulation of Cell-Adhesion Molecule Expression and Function-Associated with Neutrophil Apoptosis | 1995 |
| Ulvestad,,B,,Melbostad,,E,,Fuglerud,,P, | Asthma in tunnel workers exposed to synthetic resins | 1999 |
| Du, Lei,Zhou, Jing,Zhang, Jie,Yan, Min,Gong, Lina,Liu, Xinhao,Chen, Mi,Tao, Kaiyu,Luo, Nanfu,Liu, Jin | Actin Filament Reorganization Is a Key Step in Lung Inflammation Induced by Systemic Inflammatory Response Syndrome | 2012 |
| Valic,,F,,Zuskin,,E, | Effects of hemp dust exposure on nonsmoking female textile workers | 1971 |
| Dubovy, P.,Haninec, P. | Non-Specific Cholinesterase Activity of the Developing Peripheral Nerves and Its Possible Function in Cells in Intimate Contact with Growing Axons of Chick Embryo | 1990 |
| Valic,,F,,Zuskin,,E, | Respiratory-function changes in textile workers exposed to synthetic fibers | 1977 |
| Van, Gysel,D,,Govaere,,E,,Verhamme,,K,,Doli,,E,,De, Baets,F, | The influence of bedroom environment on sensitization and allergic symptoms in schoolchildren | 2007 |
| Van, Gysel,D,,Govaere,,E,,Verhamme,,K,,Doli,,E,,De, Baets,F, | Messages from the Aalst allergy study | 2010 |
| Van Miert, E,,Sardella,,A,,Nickmilder,,M,,Bernard,,A, | Respiratory effects associated with wood fuel use: a cross-sectional biomarker study among adolescents | 2012 |
| Veremchuk,,L, V.,Yankova,,V, I.,Vitkina,,T, I.,Nazarenko,,A, V.,Golokhvast,,K, S. | Urban air pollution, climate and its impact on asthma morbidity | 2016 |
| Villberg,,K,,Mussalo, Rauhamaa,H,,Haahtela,,T,,Saarela,,K, | Prevalence of plastic additives in indoor air related to newly diagnosed asthma | 2008 |
| Vyas,,A,,Pickering,,C, A.,Oldham,,L, A.,Francis,,H, C.,Fletcher,,A, M.,Merrett,,T,,Niven,,R, M. | Survey of symptoms, respiratory function, and immunology and their relation to glutaraldehyde and other occupational exposures among endoscopy nursing staff | 2000 |
| Dumas, E. O.,Pollack, G. M. | Opioid Tolerance Development: A Pharmacokinetic/Pharmacodynamic Perspective | 2008 |
| Dummer, W.,Rose, C.,Brocker, E. B. | Expression of CD30 on T helper cells in the inflammatory infiltrate of acute atopic dermatitis but not of allergic contact dermatitis | 1998 |
| Dummer, Wolfgang,Rose, Christian,Broecker, Eva-Bettina | Expression of CD30 on T helper cells in the inflammatory infiltrate of acute atopic dermatitis but not of allergic contact dermatitis | 1998 |
| Dunn, J.,Kartchner, L.,Jania, C.,Tilley, S.,Maile, R.,Cairns, B. A. | Woodsmoke inhalation in a murine model generates acute lung injury that is compounded by cutaneous burn | 2015 |
| Dunston, David,Ashby, Sarah,Krosnowski, Kurt,Ogura, Tatsuya,Lin, Weihong | An Effective Manual Deboning Method To Prepare Intact Mouse Nasal Tissue With Preserved Anatomical Organization | 2013 |
| Wallner,,P,,Kundi,,M,,Panny,,M,,Tappler,,P,,Hutter,,H, P. | Exposure to air ions in indoor environments: Experimental study with healthy adults | 2015 |
| Durstewitz, G.,Terwilliger, N. B. | Developmental changes in hemocyanin expression in the Dungeness crab, Cancer magister | 1997 |
| Dutch, expert committee for occupational standards | Health-based recommended occupational exposure limits for formaldehyde | 1987 |
| Dweik, R. A.,Laskowski, D.,Ozkan, M.,Farver, C.,Erzurum, S. C. | High levels of exhaled nitric oxide (NO) and NO synthase III expression in lesional smooth muscle in lymphangioleiomyomatosis | 2001 |
| Wang,,J,,Li,,B,,Yu,,W,,Yang,,Q,,Wang,,H,,Huang,,D,,Sundell,,J,,Norback,,D, | Rhinitis symptoms and asthma among parents of preschool children in relation to the home environment in Chongqing, China | 2014 |
| Wang,,X, R.,Eisen,,E, A.,Zhang,,H, X.,Sun,,B, X.,Dai,,H, L.,Pan,,L, D.,Wegman,,D, H.,Olenchock,,S, A.,Christiani,,D, C. | Respiratory symptoms and cotton dust exposure; results of a 15 year follow up observation | 2003 |
| Wang,,X, R.,Pan,,L, D.,Zhang,,H, X.,Sun,,B, X.,Dai,,H, L.,Christiani,,D, C. | Follow-up study of respiratory health of newly-hired female cotton textile workers | 2002 |
| Wang,,X, R.,Pan,,L, D.,Zhang,,H, X.,Sun,,B, X.,Dai,,H, L.,Christiani,,D, C. | A longitudinal observation of early pulmonary responses to cotton dust | 2003 |
| Wang,,X, R.,Zhang,,H, X.,Sun,,B, X.,Dai,,H, L.,Hang,,J, Q.,Eisen,,E, A.,Wegman,,D, H.,Olenchock,,S, A.,Christiani,,D, C. | A 20-year follow-up study on chronic respiratory effects of exposure to cotton dust | 2005 |
| Wantke,,F,,Demmer,,C, M.,Tappler,,P,,Gotz,,M,,Jarisch,,R, | Exposure to gaseous formaldehyde induces IgE-mediated sensitization to formaldehyde in school-children | 1996 |
| Wantke,,F,,Focke,,M,,Hemmer,,W,,Bracun,,R,,Wolf, Abdolvahab,S,,Tschabitscher,,M,,Gann,,M,,Tappler,,P,,Goetz,,M,,Jarisch,,R, | Exposure to formaldehyde and phenol during an anatomy dissecting course: sensitizing potency of formaldehyde in medical students | 2000 |
| Warman,,K,,Silver,,E, J.,Wood,,P, R. | Asthma risk factor assessment: what are the needs of inner-city families? | 2006 |
| Warman,,K,,Silver,,E, J.,Wood,,P, R. | Modifiable risk factors for asthma morbidity in Bronx versus other inner-city children | 2009 |
| Eggleston, P. A.,Rosenstreich, D.,Lynn, H.,Gergen, P.,Baker, D.,Kattan, M.,Mortimer, K. M.,Mitchell, H.,Ownby, D.,Slavin, R.,Malveaux, F. | Relationship of indoor allergen exposure to skin test sensitivity in inner-city children with asthma | 1998 |
| Egle, J. L., Jr. | Retention of inhaled formaldehyde, propionaldehyde, and acrolein in the dog | 1972 |
| White,,N, W. | Byssinosis in South Africa. A survey of 2411 textile workers | 1989 |
| White,,W, G.,Morris,,M, J.,Sugden,,E,,Zapata,,E, | Isocyanate-induced asthma in a car factory | 1980 |
| Eigenhuis, C.,Doncker, J. | Electro Receptor Functioning and Morphology Functioning during Histological Fixation | 1983 |
| Wilhelmsson,,B,,Drettner,,B, | Nasal problems in wood furniture workers. A study of symptoms and physiological variables | 1984 |
| Williams,,H,,Moyns,,E,,Bateman,,D, N.,Thomas,,S. H, L.,Thompson,,J, P.,Vale,,J, A. | Hazard of household cleaning products: A study undertaken by the UK National Poisons Information Service | 2012 |
| Willy, Fabro,A, P.,Pavan, Ramos,T,,Rozalem,,A, C.,Mallozi,,M, C.,Solé,,D,,Wandalsen,,G, F. | Adherence to environmental control recommendation among children with asthma and allergic rhinitis | 2015 |
| Winkler,,Kyle, William | Formaldehyde Exposures in a University Anatomy Laboratory | 2011 |
| Wisnewski,,A, V.,Stowe,,M,,Redlich,,C, A. | Occupational exposure to “environmentally-friendly” spray foam insulation associated with isocyanate immune responses and asthmatic symptoms | 2013 |
| Eliacyk, K.,Yenigun, A.,Meteoglu, I.,Balkaya, M. | Immunmodulation of asthma with a diet enriched with omega-3 fatty acids | 2011 |
| Elliot, J.,Wang, K.,Le Cras, T.,Larcombe, A.,Zosky, G.,James, A.,Noble, P. | Transforming growth factor alpha increases extracellular matrix within the airway smooth muscle layer in a transgenic mouse model of airway disease | 2015 |
| Elliot, W. Mark,Hayashi, Shizu,Hogg, James C. | Immunodetection of adenoviral E1A proteins in human lung tissue | 1995 |
| Elliott, W. M.,Hayashi, S.,Hogg, J. C. | Immunodetection of Adenoviral E1a Proteins in Human Lung-Tissue | 1995 |
| Ellis, R.,Leigh, R.,Southam, D.,O'Byrne, P. M.,Inman, M. D. | Morphometric analysis of mouse airways after chronic allergen challenge | 2003 |
| Elms, J.,Fishwick, D.,Robinson, E.,Burge, S.,Huggins, V.,Barber, C.,Williams, N.,Curran, A. | Specific IgE to colophony? | 2005 |
| Wong,,T, W.,Yu,,T, S.,Liu,,H, J.,Wong,,A. H, S. | Household gas cooking: A risk factor for respiratory illnesses in preschool children | 2004 |
| Elsey, C. R. | On the structure and function of the mantle and gill of Ostrea gigas (Thunberg) and Ostrea lurida (Carpenter) | 1935 |
| Wortong,,D,,Chaiear,,N,,Boonsawat,,W, | Risk of asthma in relation to occupation: A hospital-based case-control study | 2015 |
| Wright,,J,,Muhajarine,,N, | Respiratory illness in Saskatoon infants: The impact of housing and neighbourhood characteristics | 2008 |
| Wu,,F,,Takaro,,T, K. | Childhood asthma and environmental interventions | 2007 |
| Endo, L. H.,Sakano, E.,Carnargo, L. A.,Ferreira, D. R.,Pinto, G. A.,Vassallo, J. | Detection of Epstein-Barr virus in children's adenoids by in situ hybridization | 2003 |
| Xiao,,G, B.,Morinaga,,K,,Wang,,R, Y.,Xu,,L, R.,Ma,,Z, H.,Zhang,,X,,Kishimoto,,T,,Kohyama,,N, | Lung disorders of workers exposed to rush smear dust in China | 2006 |
| Yang,,C, Y.,Chiu,,J, F.,Cheng,,M, F.,Lin,,M, C. | Effects of indoor environmental factors on respiratory health of children in a subtropical climate | 1997 |
| Yang,,C, Y.,Lin,,M, C.,Hwang,,K, C. | Childhood asthma and the indoor environment in a subtropical area | 1998 |
| Yang,,C, Y.,Tien,,Y, C.,Hsieh,,H, J.,Kao,,W, Y.,Lin,,M, C. | Indoor environmental risk factors and childhood asthma: A case-control study in a subtropical area | 1998 |
| Erjefalt, J. S.,Persson, C. G. A. | Epithelial barrier formation by airway basal cells | 1997 |
| Yogev, Baggio,T,,Bibi,,H,,Dubnov,,J,,Or, Hen,K,,Carel,,R,,Portnov,,B, A. | Who is affected more by air pollution-Sick or healthy? Some evidence from a health survey of schoolchildren living in the vicinity of a coal-fired power plant in Northern Israel | 2010 |
| Yoon,,H, | Formaldehyde exposure induces airway inflammation Via regulation of the production of eotaxin and reactive oxygen species | 2007 |
| Escolar, J. D.,Escolar, M. A.,Guzman, J.,Roques, M. | Pressure volume curve and alveolar recruitment/de-recruitment. A morphometric model of the respiratory cycle | 2002 |
| Espinosa, Inigo,Briones, Javier,Bordes, Ramon,Brunet, Salut,Martino, Rodrigo,Sureda, Ana,Prat, Jaime,Sierra, Jorge | PKC-beta 2 protein expression predicts for poor response to chemotherapy and survival in patients with low risk diffuse large B-cell lymphoma | 2004 |
| Esplugues, A.,Ballester, F.,Estarlich, M.,Llop, S.,Fuentes-Leonarte, V.,Mantilla, E.,Iniguez, C. | Indoor and outdoor air concentrations of BTEX and determinants in a cohort of one-year old children in Valencia, Spain | 2010 |
| Zhang,,Y, P.,Li,,B, Z.,Huang,,C,,Yang,,X,,Qian,,H,,Deng,,Q, H.,Zhao,,Z, H.,Li,,A, G.,Zhao,,J, N.,Zhang,,X,,Qu,,F,,Hu,,Y,,Yang,,Q,,Wang,,J,,Zhang,,M,,Wang,,F,,Zheng,,X, H.,Lu,,C,,Liu,,Z, J.,Sun,,Y, X.,Mo,,J, H.,Zhao,,Y, L.,Liu,,W,,Wang,,T, T.,Norback,,D,,Bornehag,,C, G.,Sundell,,J, | Ten cities cross-sectional questionnaire survey of children asthma and other allergies in China | 2013 |
| Etzel, R. A. | Indoor Air-Pollution and Childhood Asthma - Effective Environmental Interventions | 1995 |
| Zheng,,T,,Niu,,S,,Lu,,B,,Fan,,X,,Sun,,F,,Wang,,J,,Zhang,,Y,,Zhang,,B,,Owens,,P,,Hao,,L,,Li,,Y,,Leaderer,,B, | Childhood asthma in Beijing, China: a population-based case-control study | 2002 |
| Eyles, J.,Keller-Olaman, S.,Wilson, K. | Environmental disadvantage, and housing, and neighborhood quality in Hamilton, Ontario, Canada | 2005 |
| Zuskin,,E,,Ivankovic,,D,,Schachter,,E, N.,Witek,,T, J.,Jr, | A ten-year follow-up study of cotton textile workers | 1991 |
| Zuskin,,E,,Kanceljak,,B,,Mustajbegovic,,J,,Godnic, Cvar,J,,Schachter,,E, N. | Immunological reactions and respiratory function in wool textile workers | 1995 |
| Fabian, P.,Adamkiewicz, G.,Levy, J. I. | Simulating indoor concentrations of NO2 and PM2.5 in multifamily housing for use in health-based intervention modeling | 2012 |
| Fadili, S.,Clarencon, F.,Cormier, E.,Le Jean, L.,Chiras, J. | Intracardiac cement migration after percutaneous vertebroplasty incidence and risk factors | 2014 |
| Zuskin,,E,,Kanceljak,,B,,Mustajbegovic,,J,,Schachter,,E, N.,Kern,,J, | Respiratory function and immunological reactions in sisal workers | 1994 |
| Zuskin,,E,,Kanceljak,,B,,Schachter,,E, N.,Witek,,T, J.,Maayani,,S,,Goswami,,S,,Marom,,Z,,Rienzi,,N, | Immunological findings in hemp workers | 1992 |
| Zuskin,,E,,Kanceljak,,B,,Schachter,,E, N.,Witek,,T, J.,Mustajbegovic,,J,,Maayani,,S,,Buck,,M, G.,Rienzi,,N, | Immunological findings and respiratory function in cotton textile workers | 1992 |
| Faiz, A.,Tjin, G.,Harkness, L. M.,Oliver, B.,Black, J. L.,Burgess, J. K. | Secreted cathepsin H activity is regulated by corticosteroids and may affect airway remodelling in asthma | 2012 |
| Falkenstern-Ge, R. F.,Ingerl, H.,Kohlhäufl, M. | Lung emphysema treated successfully using volume reduction with lung sealant (AeriSeal®) | 2013 |
| Famulski, Jakub K.,Solecki, D. J. | Brainstem respiratory networks: building blocks and microcircuits | 2013 |
| Fan, Chuifeng,Lin, Xuyong,Wang, Enhua | Clinicopathological significance of cathepsin D expression in non-small cell lung cancer is conditional on apoptosis-associated protein phenotype: an immunohistochemistry study | 2012 |
| Fanat, A. I.,Thomson, J. V.,Radford, K.,Nair, P.,Sehmi, R. | Human airway smooth muscle promotes eosinophil differentiation | 2009 |
| Zuskin,,E,,Mustajbegovic,,J,,Schachter,,E, N. | Follow-up study of respiratory function in hemp workers | 1994 |
| Zuskin,,E,,Mustajbegovic,,J,,Schachter,,E, N.,Doko, Jelinic,J, | Respiratory function of textile workers employed in dyeing cotton and wool fibers | 1997 |
| Zuskin,,E,,Mustajbegovic,,J,,Schachter,,E, N.,Kanceljak,,B,,Godnic, Cvar,J,,Sitar, Srebocan,V, | Respiratory symptoms and lung function in wool textile workers | 1995 |
| Zuskin,,E,,Mustajbegovic,,J,,Schachter,,E, N.,Kern,,J,,Budak,,A,,Godnic, Cvar,J, | Respiratory findings in synthetic textile workers | 1998 |
| Zuskin,,E,,Valic,,F,,Butkovic,,D,,Bouhuys,,A, | Lung function in textile workers | 1975 |
| Zuskin,,Eugenija,,Mustajbegovic,,Jadranka,,Kanceljak,,Bozica,,Schachter,,E. Neil,Macan,,Jelena,,Budak,,Antun, | Respiratory function and immunological status in workers employed in a latex glove manufacturing plant | 1998 |
| Farshidpanah, S.,Cheek, G.,Wallen, J.,McArthur, C. | Blades, scopes, and chambers: A multidisciplinary approach to treating metastatic hemangioendothelioma complicated by persistent bronchopleural fistula | 2012 |
| Faruki, A.,Eapen, G. | Symptomatic polymethyl methacrylate-induced cement pulmonary embolism after kyphoplasty | 2012 |
| Fauziah, O.,Purton, M. D.,Solomon, S. E. | Scanning electron microscopy of the respiratory epithelium of chicks fumigated with formaldehyde vapour | 1996 |
| Fazal, Nadeem,Shelip, Alla,Siddiqui, Erum,Ali, Ashraf,Azim, Anser C.,Al-Ghoul, Walid M. | Differential effector responses by circulating/blood and tissue/peritoneal neutrophils following burn combined with Enterococcus faecalis infection | 2012 |
| Fazekas de St. Groth, S.,Donnelley, Margaret | Studies in experimental immunology of influenza. IV. The protective value of active immunization | 1950 |
| Fedak, P. W. M.,Kieser, T. M.,Maitland, A. M.,Holland, M.,Kasatkin, A.,LeBlanc, P.,Kim, J. K.,King, K. M. | Adhesive-Enhanced Sternal Closure to Improve Postoperative Functional Recovery: A Pilot, Randomized Controlled Trial | 2011 |
| Fedulov, Alexey V.,Kobzik, Lester | Immunotoxicologic analysis of maternal transmission of asthma risk | 2008 |
| Feinman, S. E. | Unusual Skin Manifestations of Exposure to Formaldehyde | 1988 |
| Feinman, S. E. | Respiratory Effects from Formaldehyde | 1988 |
| Felton, S.,Williams, J. D. L. | Contact dermatitis to 'Butterfly Wings' | 2012 |
| Feng, Rui-e,Liu, Hong-rui,Liang, Zhi-yong,Shi, Ju-hong,Zhu, Yuan-jue,Liu, Dong-ge,Ke, Hui-xing,Yu, Ji-yao | Clinicopathologic study of Churg-Strauss syndrome | 2008 |
| Feng, R. E.,Liu, H. R.,Liang, Z. Y.,Shi, J. H.,Zhu, Y. J.,Mu, D. G.,Ke, H. X.,Yu, J. Y. | [Clinicopathologic study of Churg-Strauss syndrome] | 2008 |
| Fent, K. W.,Jayaraj, K.,Gold, A.,Ball, L. M.,Nylander-French, L. A. | Tape-strip sampling for measuring dermal exposure to 1,6-hexamethylene diisocyanate | 2006 |
| Fergusson, R. J.,Milne, L. J.,Crompton, G. K. | Penicillium allergic alveolitis: faulty installation of central heating | 1984 |
| Fernandez, J.,Sisti, F. B.,Rodriguez, M. E.,Yantorno, O. M.,Hozbor, D. F. | Flagellin, a bvg-repressed factor, has a critical role in Bordetella bronchiseptica-host interaction | 2002 |
| Fernandez-Caldas, E.,Gallego, M.,Carnes, J.,Iraola, V. | Enzymatic activity of Dermatophagoides pteronyssinus extracts after acidic treatment | 2008 |
| Fernandez-Fernandez, J.,Kinner, V.,Ferrauti, A. | The physiological demands of hitting and running in tennis on different surfaces | 2010 |
| Ferrante, A.,Harvey, D. P.,Bates, E. J. | Staphylococcus-Aureus-Stimulated Mononuclear Leukocyte-Conditioned Medium Increases the Neutrophil Bactericidal Activity and Augments Oxygen Radical Production and Degranulation in Response to the Bacteria | 1989 |
| Ferrante, A.,Nandoskar, M.,Bates, E. J.,Goh, D. H. B. | Staphylococcus-Aureus-Stimulated Human Mononuclear Leukocyte-Conditioned Medium Augments the Basal and Stimuli-Induced Neutrophil Respiratory Burst and Degranulation | 1987 |
| Ferrari, C.,Cooley, J. A.,Costa, L.,Mays, S.,Swiderski, C. E. | Horses with 'pasture heaves' have airway remodeling that is characteristic of asthmatic airways | 2015 |
| Ferreira, A. M. C.,Cardoso, S. M. | Exploratory study of air quality in elementary schools, Coimbra, Portugal | 2013 |
| Ferreira, H. H. A.,Mendes, J. A.,Ribeiro, M. C.,Moreira, G. C. P.,Silva, M. S.,Dias, N. H.,Albaladejo, B. T.,Pereira, J. A.,Rocha, T. | Does hydrogen sulfide influence apoptosis process in lungs from allergic mice? | 2015 |
| Ferreira, H. H. D.,Costa, R. A. D.,Jacheta, J. M.,Martins, A. R.,Medeiros, M. V.,Macedo-Soares, M. F.,De Luca, I. M. S.,Antunes, E.,De Nucci, G. | Modulation of eosinophil migration from bone marrow to lungs of allergic rats by nitric oxide | 2004 |
| Ferreira-Duarte, A. P.,Squebola-Cola, D.,Mello, G.,Antunes, E.,DeSouza, I. | Inhibitory effects of staphylococcal enterotoxin type A (SEA) and B (SEB) on mice bone marrow eosinophil adhesion in vitro | 2013 |
| Ferres, J.,Justicia, J. L.,Garcia, M. P.,Munoz-Tuduri, M.,Alva, V. | Efficacy of high-dose sublingual immunotherapy in children allergic to house dust mites in real-life clinical practice | 2011 |
| Fetsch, Patricia A.,Abati, Andrea,Litman, Thomas,Morisaki, Kuniaki,Honjo, Yasumasa,Mittal, Khush,Bates, Susan E. | Localization of the ABCG2 mitoxantrone resistance-associated protein in normal tissues | 2006 |
| Fey, K. | Massive administration of isotonic saline solution to RAO horses - a review | 2010 |
| Feyen, H.,Hiemeyer, V. | [Studies on the control of inflammation in acute and chronic inflammatory disease] | 1969 |
| Filley, W. V.,Holley, K. E.,Kephart, G. M.,Gleich, G. J. | Identification by immunofluorescence of eosinophil granule major basic protein in lung tissues of patients with bronchial asthma | 1982 |
| Finkelstein, J.,Hripcsak, G.,Cabrera, M. | Telematic system for monitoring of asthma severity in patients' homes | 1998 |
| Finn, Adam,Rebuck, Naomi,Strobel, Stephan,Moat, Neil,Elliott, Martin | Systemic inflammation during paediatric cardiopulmonary bypass: Changes in neutrophil adhesive properties | 1993 |
| Fischer, A. J.,Adam, R. J.,Michalski, A. S.,Hoegger, M. J.,McMenimen, J. D.,Ostedgaard, L. S.,Stoltz, D. A.,Welsh, M. J. | Measurement of the physical properties of airway and intestinal mucus | 2014 |
| Fischer, E. F.,Vogel, P.,Ziegler, A. S.,Kirkpatrick, C. J. | Effect of Fibrinogen Fragments D and E on the Adhesive Properties of Human Granulocytes to Venous Endothelial Cells | 1991 |
| Fischer, E. G.,Vogel, P.,Ziegler, A. S.,Kirkpatrick, C. J. | Effect of Fibrinogen Fragment-D and Fragment-E on the Adhesive Properties of Human Granulocytes to Venous Endothelial-Cells | 1991 |
| Fischer, Kathleen E.,Nagaraj, Ganesh,Daniels, R. Hugh,Li, Esther,Cowles, Verne E.,Miller, Jennifer L.,Bunger, Mark D.,Desai, Tejal A. | Hierarchical nanoengineered surfaces for enhanced cytoadhesion and drug delivery | 2011 |
| Fish, James E. | Occupational asthma and rhinoconjunctivitis induced by natural rubber latex exposure | 2002 |
| Fitzgerald, K. T.,Bronstein, A. C. | Polyurethane adhesive ingestion | 2013 |
| Flajsig, I.,Cuch, E. C. Y.,Mayosky, A. A.,Rodriguez, R.,Calbet, J. M.,Saura, E.,Fontanillas, C.,Granados, J.,Miralles, A.,Benito, M.,Rabasa, J. M.,Sbraga, F.,Rullan, C.,de Toledo, M. C. O. | Surgical treatment of left ventricular free wall rupture after myocardial infarction: Case series | 2002 |
| Flamant-Hulin, M.,Annesi-Maesano, I.,Caillaud, D. | Relationships between molds and asthma suggesting non-allergic mechanisms. A rural-urban comparison | 2013 |
| Fleischel, Olivier,Gimenez-Amau, Elena,Lepoittevin, Jean-Pierre | Nuclear Magnetic Resonance Studies on Covalent Modification of Amino Acids Thiol and Amino Residues by Monofunctional Aryl C-13-Isocyanates, Models of Skin and Respiratory Sensitizers: Transformation of Thiocarbamates into Urea Adducts | 2009 |
| Fletcher, A.,Choi, J.,Awadalla, M.,Potash, A. E.,Wallen, T. J.,Fletcher, S.,Chang, E. H. | The effect of geniglossal advancement on airway flow using a computational flow dynamics model | 2013 |
| Flisberg, P.,Tornebrandt, K.,Walther, B.,Lundberg, J. | A comparison of the effects on postoperative pain relief of epidural analgesia started before or after surgery | 2000 |
| Flohr, C.,Lee, K. K.,Varma, S. | Merocel nasal packing with airway: a method to help secure full-thickness skin grafts on the nasal tip | 2009 |
| Flood-page, P. T.,Partridge, M. R. | Asthma: A changing perspective on management | 1996 |
| Flores-Calderón, J.,Morán-Villota, S.,Rouassant, S. H.,Nares-Cisneros, J.,Zárate-Mondragón, F.,González-Ortiz, B.,Chávez-Barrera, J. A.,Vázquez-Frías, R.,Martínez-Marín, E. J.,Marín-Rentería, N.,Bojórquez-Ramos, M. C.,Castillo-De León, Y. A.,Ortiz-Galván, R. C.,Varela-Fascinetto, G. | Guidelines for the diagnosis and treatment of extrahepatic portal vein obstruction (EHPVO) in children | 2013 |
| Florian, Stefan,Ghannadan, Minoo,Mayerhofer, Matthias,Aichberger, Karl J.,Hauswirth, Alexander W.,Schernthaner, Gerit-Holger,Printz, Dieter,Fritsch, Gerhard,Boehm, Alexandra,Sonneck, Karoline,Krauth, Maria-Theresa,Mueller, Michael R.,Sillaber, Christian,Sperr, Wolfgang R.,Buehring, Hans-Joerg,Valent, Peter | Evaluation of normal and neoplastic human mast cells for expression of CD172a (SIRP alpha), CD47, and SHP-1 | 2005 |
| Fodeman, J.,Jariwala, S. P.,Jerschow, E.,Hudes, G.,Rosenstreich, D. | Even hydroxyzine can make you itch | 2010 |
| Folch, E.,Hillerdal, G. | When small is better: Volume reduction in severe emphysema | 2015 |
| Fonseca, J. A.,Costa-Pereira, A.,Delgado, L.,Fernandes, L.,Castel-Branco, M. G. | Asthma patients are willing to use mobile and web technologies to support self-management | 2006 |
| Fontan, J. J. P.,Clyman, R. I.,Mauray, F.,Heymann, M. A.,Roman, C. | Respiratory Effects of a Patent Ductus Arteriosus in Premature Newborn Lambs | 1987 |
| Foong, R.,Berry, L.,Hart, P.,Gorman, S.,Zosky, G. | Vitamin D deficiency alters airway structure and function in a chronic house dust mite exposure model | 2013 |
| Foong, R. E.,Gorman, S.,Hart, P. H.,Zosky, G. R. | Vitamin D deficiency alters lung function but does not have an effect on airway smooth muscle mass | 2012 |
| Foong, R. E.,Gorman, S.,Hart, P. H.,Zosky, G. R. | Vitamin d deficiency causes deficits in lung function but does not alter airway smooth muscle mass | 2012 |
| Foong, R. E.,Shaw, N. C.,Berry, L. J.,Hart, P. H.,Gorman, S.,Zosky, G. R. | Vitamin D deficiency causes airway hyperresponsiveness, increases airway smooth muscle mass, and reduces TGF-beta expression in the lungs of female BALB/c mice | 2014 |
| Foong, R. E.,Shaw, N. C.,Hart, P. H.,Gorman, S.,Zosky, G. R. | Maternal vitamin d deficiency does not cause airway hyperresponsiveness and increased airway smooth muscle in young mice | 2014 |
| Foot, N. J.,Orgeig, S.,Daniels, C. B. | The evolution of a physiological system: The pulmonary surfactant system in diving mammals | 2006 |
| Foresi, A.,Pesci, A.,Pelucchi, A.,Gabrielli, M.,Mastropasqua, B.,Bertorelli, G.,Chetta, A.,Olivieri, D. | Bronchial inflammation in mite-sensitive asthmatic subjects after 5 years of specific immunotherapy | 1992 |
| Fornasa, G.,Tsilingiri, K.,Caprioli, F.,Botti, F.,Mapelli, M.,Meller, S.,Kislat, A.,Homey, B.,Di Sabatino, A.,Sonzogni, A.,Viale, G.,Diaferia, G.,Gori, A.,Longhi, R.,Penna, G.,Rescigno, M. | Dichotomy of short and long thymic stromal lymphopoietin isoforms in inflammatory disorders of the bowel and skin | 2015 |
| Foroughi, S.,Thyagarajan, A.,Stone, K. D. | Advances in pediatric asthma and atopic dermatitis | 2005 |
| Forsgren, Arne,Brant, Marta,Riesbeck, Kristian | Immunization with the truncated adhesin Moraxella catarrhalis immunoglobulin D-binding protein (MID764-913) is protective against m. catarrhalis in a mouse model of pulmonary clearance | 2004 |
| Fortoul, T. I.,Rojas-Lemus, M.,Rodriguez-Lara, V.,Gonzalez-Villalva, A.,Ustarroz-Cano, M.,Cano-Gutierrez, G.,Gonzalez-Rendon, S. E.,Montano, L. F.,Altamirano-Lozano, M. | Overview of environmental and occupational vanadium exposure and associated health outcomes: An article based on a presentation at the 8th International Symposium on Vanadium Chemistry, Biological Chemistry, and Toxicology, Washington DC, August 15-18, 2012 | 2014 |
| Foster, Paul S. | Allergic networks regulating eosinophilia | 1999 |
| Fowler, Joseph F.,Skinner, Steven M.,Belsito, Donald V. | Allergic contact dermatitis from formaldehyde resins in permanent press clothing: An underdiagnosed cause of generalized dermatitis | 1992 |
| Fowler, S.,Byron, O.,Jumel, K.,Xing, D.,Corbel, M. J.,Bolgiano, B. | Novel configurations of high molecular weight species of the pertussis toxin vaccine component | 2003 |
| Fox, R. W. | The fungal phenomenon | 2004 |
| Fox, S.,Tobin, M. C.,Buvanendran, A.,Sheinkop, M. | Case series: Identification of patients at risk for arthroplasty failure | 2009 |
| Fraire, A. E.,Shahab, I.,Greenberg, S. D.,Jubran, A.,Noall, M. | Experimental polyacrylamide-induced acute injury in rat lung | 1992 |
| Franchi, M.,Carrer, P.,Kotzias, D.,Rameckers, E. M.,Seppanen, O.,van Bronswijk, J. E.,Viegi, G.,Gilder, J. A.,Valovirta, E. | Working towards healthy air in dwellings in Europe | 2006 |
| Francis, Arul Prakash,Ganapathy, Selvam,Palla, Venkata Rajsekhar,Murthy, Prakhya Balakrishna,Devasena, Thiyagarajan | Future of nano bisdemethoxy curcumin analog: Guaranteeing safer intravenous delivery | 2015 |
| Franco, A. L. D.,Damazo, A. S.,de Souza, H. R. B.,Domingos, H. V.,Oliveira, R. M.,Oliani, S. M.,Costa, S. K. P.,de Lima, W. T. | Pulmonary neutrophil recruitment and bronchial reactivity in formaldehyde-exposed rats are modulated by mast cells and differentially by neuropeptides and nitric oxide | 2006 |
| Franco, A. L. D.,Dorningos, H. V.,Damazo, A. S.,Breithaupt-Faloppa, A. C.,de Oliveira, A. P. L.,Costa, S. K. P.,Oliani, S. M.,Oliveira, R. M.,Vargaftig, B. B.,Tavares-De-Lima, W. | Reduced allergic lung inflammation in rats following formaldehyde exposure: Long-term effects on multiple effector systems | 2009 |
| Franco, G. | New trends in occupational and environmental diseases: the role of the occupational hygienist in recognizing lung diseases | 1994 |
| Frank, Evan A.,Birch, M. Eileen,Yadav, Jagjit S. | MyD88 mediates in vivo effector functions of alveolar macrophages in acute lung inflammatory responses to carbon nanotube exposure | 2015 |
| Frank, Simon J. | Medical device for overcoming airway obstruction | 2005 |
| Franklin, P. J. | Indoor air quality and respiratory health of children | 2007 |
| Freck, E.,Weller, R.,Anz, C.,Shields, J.,Caldwell, J. | True tetracaine allergy following spinal anesthesia, manifesting as asymmetric urticaria | 2010 |
| Freed, D. L. J.,Mansfield, J. | Asthma: What we do and why we do it | 2008 |
| Freedman, B. J. | Sulfur Di Oxide in Foods and Beverages Its Use as a Preservative and Its Effect on Asthma | 1980 |
| Freeman, L.,Wolford, R. W. | Methemoglobinemia secondary to cleaning solution ingestion | 1996 |
| Frey, D. J.,Matas, A. J.,Gillingham, K. J.,Canafax, D.,Payne, W. D.,Dunn, D. L.,Sutherland, D. E.,Najarian, J. S. | Sequential therapy--a prospective randomized trial of MALG versus OKT3 for prophylactic immunosuppression in cadaver renal allograft recipients | 1992 |
| Frey, G.,Bock, K. H.,Meister, H.,Haug, H. U.,Kilian, J.,Ahnefeld, F. W. | [Effects of ventilation with defined formaldehyde concentrations on lung function and lung structures. Animal experiments on the noxiousness of formaldehyde residues after disinfection in the aseptor (author's transl)] | 1979 |
| Freyer, D. R.,Boxer, L. A.,Axtell, R. A.,Todd, R. F., III | Stimulation of Human Neutrophil Adhesive Properties by Adenine Nucleotides | 1988 |
| Fridell, E.,Haeger-Eugensson, M.,Moldanova, J.,Forsberg, B.,Sjoberg, K. | A modelling study of the impact on air quality and health due to the emissions from E85 and petrol fuelled cars in Sweden | 2014 |
| Friedman, Meyer,Byers, Sanford O.,Rosenman, Ray H. | Observation concerning the production and excretion of cholesterol in mammals. XII. Demonstration of the essential role of the hepatic reticulo-endothelial cell (kupffer cell) in the normal disposition of exogenously derived cholesterol | 1954 |
| Friedman, M. A.,Watt, K. M.,Higgins, E. S. | In-Vitro and in-Vivo Effects of Di Methyl Nitrosamine on Mouse Liver Mitochondrial Function | 1977 |
| Frieri, M.,Patel, R. | Unusual presentation of facial angioedema | 2012 |
| Frieri, M.,Valluri, A. | Vitamin D deficiency as a risk factor for allergic disorders and immune mechanisms | 2011 |
| Frigas, E.,Filley, W. V.,Reed, C. E. | Asthma induced by dust from urea-formaldehyde foam insulating material | 1981 |
| Fromme, H.,Bischof, W.,Dietrich, S.,Lahrz, T.,Schierl, R.,Schwegler, U. | Airborne Allergens, Endotoxins, and Particulate Matter in Elementary Schools, Results from Germany (LUPE 2) | 2013 |
| Frondoza, C. G.,Levy, D. A. | Anaphylaxis in Chickens Evidence for in-Vivo and in-Vitro Histamine Release | 1977 |
| Fruchter, O.,Refaely, Y.,Maimon, M. N.,Kramer, M. | Physiological basic for long term improvement following bilateral AeriSeal® system foam sealant lung volume reduction for advanced emphysema | 2012 |
| Fruchter, O.,Rosengarten, D.,Goldberg, E.,Ben-Zvi, H.,Tor, R.,Kramer, M. R. | Airway bacterial colonization and serum C-reactive protein are associated with chronic obstructive pulmonary disease exacerbation following bronchoscopic lung volume reduction | 2016 |
| Fry, R. C.,Rager, J. E.,Bauer, R.,Sebastian, E.,Peden, D. B.,Jaspers, I.,Alexis, N. E. | Air toxics and epigenetic effects: ozone altered microRNAs in the sputum of human subjects | 2014 |
| Fuentes, Victoria,Matheu, Victor,Barranco, Ruth,Tornero, Pilar,Barrio, Manolo,Quirce, Santiago,Baeza, M. L. | Occupational asthma primarily caused by cyanoacrylate in an assembly operator | 2000 |
| Fujibayashi, K.,Kubota-Watanabe, M.,Iizuka, Y. | Effects of R-84760, a selective kappa-opioid receptor agonist, on nociception, locomotion and respiration in rats | 1996 |
| Fujimaki, H.,Kawagoe, A.,Bissonnette, E.,Befus, D. | Mast-Cell Response to Formaldehyde .1. Modulation of Mediator Release | 1992 |
| Fujimaki, H.,Kurokawa, Y.,Kunugita, N.,Kikuchi, M.,Sato, F.,Arashidani, K. | Differential immunogenic and neurogenic inflammatory responses in an allergic mouse model exposed to low levels of formaldehyde | 2004 |
| Fujimaki, H.,Yamamoto, S.,Tin Tin Win, Shwe,Hojo, R.,Sato, F.,Kunugita, N.,Arashidani, K. | Effect of long-term exposure to low-level toluene on airway inflammatory response in mice | 2007 |
| Fujimura, S.,Akaogi, E.,Kondo, T.,Yamauchi, A.,Okabe, T.,Handa, M.,Shionozaki, F.,Saitoh, R.,Nakada, T. | Experience with Surgery for Recurrent Carcinoid Tumor of the Thymus | 1983 |
| Fujisawa, T.,Kephart, G. M.,Gray, B. H.,Gleich, G. J. | The neutrophil and chronic allergic inflammation. Immunochemical localization of neutrophil elastase | 1990 |
| Fujishima, S.,Nakamura, H.,Waki, Y.,Soejima, K.,Takeuchi, Y.,Ogawa, M.,Shiozawa, M.,Hiraoka, Y.,Kawashiro, T.,Aiso, S.,Kanazawa, M. | Cell-associated IL-8 in human blood monocytes: Analysis by flow cytometry | 1996 |
| Fujiwara, M.,Patino, R.,Kukes, G.,Lee, J.,Guerra, C.,Sassoon, C.,Kota, C.,Mazdisnian, F. | Study of the trachea after Teflon injection | 2012 |
| Fukuda, T.,Nishimoto, C.,Miyabe, M.,Toyooka, H. | Unilateral adrenalectomy attenuates hemorrhagic shock-induced analgesia in rats | 2007 |
| Fukui, Mitsuru | Expiratory and urinary elimination of styrene in rats | 1992 |
| Gabehart, K.,Correll, K. A.,Yang, J.,Collins, M. L.,Loader, J. E.,White, C. W.,Dakhama, A. | Effect of postnatal ozone exposure on the developing lung | 2011 |
| Gaffin, J. M.,Kanchongkittiphon, W.,Phipatanakul, W. | Perinatal and early childhood environmental factors influencing allergic asthma immunopathogenesis | 2014 |
| Gaffuri, E.,Brugnone, F. | [Respiratory pathology caused by isocyanates in varnishers] | 1971 |
| Gagarine, A.,Urschel, J. D.,Miller, J. D.,Bennett, W. F.,Young, J. E. M. | Effect of fibrin glue on air leak and length of hospital stay after pulmonary lobectomy | 2003 |
| Gagliardi, L.,Ambroso, M.,Mavro, J.,Furno, F.,Discalzi, G. | Exposure to paraphenylendiamine in hairdressing parlours | 1992 |
| Gagne, S.,Lesage, J.,Ostiguy, C.,Tra, H. V. | Determination of unreacted 2,4-toluene diisocyanate (2,4TDI) and 2,6-toluene diisocyanate (2,6TDI) in foams at ultratrace level by using HPLC-CIS-MS-MS | 2003 |
| Gaillard, Y. P.,Cuquel, A. C.,Boucher, A.,Romeuf, L.,Bevalot, F.,Prevosto, J. M.,Menard, J. M. | A fatality following ingestion of the designer drug meta-chlorophenylpiperazine (mCPP) in an asthmatic--HPLC-MS/MS detection in biofluids and hair | 2012 |
| Gallego, M. T.,Iraola, V.,Himly, M.,Robinson, D. S.,Badiola, C.,Garcia-Robaina, J. C.,Briza, P.,Carnes, J. | Depigmented and Polymerised House Dust Mite Allergoid: Allergen Content, Induction of IgG4 and Clinical Response | 2010 |
| Gallen, C. | Allergies and vaccines | 2008 |
| Galvain, S.,Andre, C.,Vatrinet, C.,Villet, B. | Safety and efficacy studies of liposomes in specific immunotherapy | 1999 |
| Gamberi, G.,Cocchi, S.,Benini, S.,Magagnoli, G.,Kreshak, J.,Gambarotti, M.,Picci, P.,Vanel, D.,Alberghini, M. | Diagnostic accuracyand approach in the ewing family of tumors using molecular and immunohistochemical techniques | 2011 |
| Gamboa, P. M.,de la Cuesta, C. G.,Garcia, B. E.,Castillo, J. G.,Oehling, A. | Late asthmatic reaction in a hairdresser, due to the inhalation of ammonium persulphate salts | 1989 |
| Gamkrelidze, S. | Identifying specific allergens and monitoring concentration of air-pollution in West Georgia | 2011 |
| Gandevia, B. | STUDIES OF VENTILATORY CAPACITY AND HISTAMINE RESPONSE DURING EXPOSURE TO ISOCYANATE VAPOUR IN POLYURETHANE FOAM MANUFACTURE | 1963 |
| Ganguly, R.,Batterman, S.,Isakov, V.,Snydert, M.,Breen, M.,Brakefield-Caldwell, W. | Effect of geocoding errors on traffic-related air pollutant exposure and concentration estimates | 2015 |
| Gannon, P. F.,Berg, A. S.,Gayosso, R.,Henderson, B.,Sax, S. E.,Willems, H. M. | Occupational asthma prevention and management in industry--an example of a global programme | 2005 |
| Gannon, P. F. G.,Berg, A. S.,Gayosso, R.,Henderson, B.,Sax, S. E.,Willems, H. M. J. | Occupational asthma prevention and management in industry - an example of a global programme | 2005 |
| Garcia Algar, O.,Pichini, S.,Basagana, X.,Puig, C.,Vall, O.,Torrent, M.,Harris, J.,Sunyer, J.,Cullinan, P. | Concentrations and determinants of NO2 in homes of Ashford, UK and Barcelona and Menorca, Spain | 2004 |
| Garcia Martinez, J. | [Importance of environmental and preventive measures in the control of asthma in the child] | 2000 |
| García-Abujeta, J. L.,Girones, M. A.,De Larramendi, C. H.,Montoro, J.,De Las Vecillas, L.,Vicario, S.,Rodríguez, F. | Standard patch series around the world. Different place, different patches | 2016 |
| Garcia-Algar, O.,Zapater, M.,Figueroa, C.,Vall, O.,Basagana, X.,Sunyer, J.,Freixa, A.,Guardino, X.,Pichini, S. | Sources and concentrations of indoor nitrogen dioxide in Barcelona, Spain | 2003 |
| Garcia-Delgado, M.,Navarrete-Sanchez, I.,Colmenero, M.,Touma-Fernandez, A.,Lopez-Cuervo, J. E.,Hassan-Montero, L.,Fernandez-Mondejar, E. | Intermittent alveolar overdistension for 30 or 240 minutes does not produce acute lung injury in normal pig lung | 2006 |
| Garcia-Marcos, L.,Batlles-Garrido, J.,Blanco-Quiros, A.,Garcia-Hernandez, G.,Guillen-Grima, F.,Gonzalez-Diaz, C.,Garcia-Merino, A.,Arnedo-Pena, A.,Busquets-Monge, R. M.,Morales-Suarez-Varela, M.,Lopez-Silvarrey-Varela, A.,Garcia-Andoin, N. | Influence of two different geo-climatic zones on the prevalence and time trends of asthma symptoms among Spanish adolescents and schoolchildren | 2009 |
| Garcia-Marcos, L.,Guillen, J. J.,Dinwiddie, R.,Guillen, A.,Barbero, P. | The relative importance of socio-economic status, parental smoking and air pollution (SO2) on asthma symptoms, spirometry and bronchodilator response in 11-year-old children | 1999 |
| Garcia-Ortiz, R.,Munilla, G.,Kulczewski, A.,Silva, L.,Munoz, G.,Miranda, B. | [Byssinosis: an occupational disease of textile workers] | 1987 |
| Gargouri, I.,Fantoni, S.,Masmoudi, M. L.,Gharbi, R.,Frimat, P. | Allergens in the care environment: aetiology, epidemiology and clinical demonstrations | 2002 |
| Garnett, James A.,Muhl, Daniela,Douse, Christopher H.,Hui, Kailyn,Busch, Andreas,Omisore, Ayodele,Yang, Yi,Simpson, Peter,Marchant, Jan,Waksman, Gabriel,Matthews, Steve,Filloux, Alain | Structure-function analysis reveals that the Pseudomonas aeruginosa Tps4 two-partner secretion system is involved in CupB5 translocation | 2015 |
| Garrett, M. H.,Rayment, P. R.,Hooper, M. A.,Abramson, M. J.,Hooper, B. M. | Indoor airborne fungal spores, house dampness and associations with environmental factors and respiratory health in children | 1998 |
| Garvey, J. | Sick building syndrome: Developing an integrated approach to SBS | 1994 |
| Garza, K. M.,Murr, L. E.,Soto, K. F. | ENVR 56-Cytotoxicity and related inflammatory response for some manufactured metal oxide and carbon nanoparticulate material aggregates | 2008 |
| Gasiorowska, J.,Czerwionka-Szaflarska, M.,Swincow, G.,Stefanska, A.,Sypniewska, G. O. | Analysis of concentration of adhesion molecules ICAM-1 and VCAM-1 in infants and young children with wheezy bronchitis | 2010 |
| Gassner, M.,Spuhler, T. | Why Are Deaths from Lung-Diseases More Frequent among the Farming Population | 1995 |
| Gauggel, D. L.,Sarlo, K.,Asquith, T. N. | A Proposed Screen for Evaluating Low-Molecular-Weight Chemicals as Potential Respiratory Allergens | 1993 |
| Gaundar, Shiva,Blyth, Emily,Clancy, Leighton,Simms, Renee,Gottlieb, David | In Vitro Generation of Influenza-Virus Specific T Cells for Adoptive Immunotherapy | 2011 |
| Gavett, S. H.,Haykal-Coates, N.,Copeland, L. B.,Heinrich, J.,Gilmour, M. I. | Metal composition of ambient PM2.5 influences severity of allergic airways disease in mice | 2003 |
| Gavett, S. H.,Koren, H. S. | The role of particulate matter in exacerbation of atopic asthma | 2001 |
| Gavino, A. C.,Tweardy, D. J. | Small-molecule inhibition of stat3 prevents house-dust-mite (HDM)-induced airway inflammation by blocking lung production of Th17 and Th2 cytokines | 2014 |
| Gavriely, N.,Grotberg, J. B. | Flow Limitation and Wheezes in a Constant Flow and Volume Lung Preparation | 1988 |
| Gazca Aguilar, A.,Ortega Cisneros, M.,del Rio Navarro, B.,Sienra Monge, J. J. | [Asthma physiopathology] | 1998 |
| Ge, Xiao Na,Ha, Sung Gil,Liu, Fu-Tong,Rao, Savita P.,Sriramarao, P. | Eosinophil-expressed galectin-3 regulates cell trafficking and migration | 2013 |
| Ge, X. N.,Ha, S. G.,Liu, F. T.,Rao, S. P.,Sriramarao, P. | Eosinophil-expressed galectin-3 regulates cell trafficking and migration | 2013 |
| Gee, I. L.,Watson, A. F. R.,Tavernier, G.,Stewart, L. J.,Fletcher, G.,McL Niven, R. | Indoor air quality, environmental tobacco smoke and asthma: A case control study of asthma in a community population | 2005 |
| Geens, T.,Dugardin, S.,Schockaert, A.,De Cooman, G.,van Sprundel, M. | Air exposure assessment of TDI and biological monitoring of TDA in urine in workers in polyurethane foam industry | 2011 |
| Gehse, M.,Gehring, W.,Gloor, M. | [Occupationally-induced formaldehyde allergy of the immediate type] | 1988 |
| Gelb, A. F.,Yamamoto, A.,Mauad, T.,Kollin, J.,Schein, M. J.,Tran, D.,Beaird, D.,Moridzadeh, R.,Fraser, C.,Nadel, J. A. | Phenotype of chronic asthmatics who never smoked with persistent airway obstruction | 2014 |
| Gent, J. F.,Belanger, K.,Triche, E. W.,Bracken, M. B.,Beckett, W. S.,Leaderer, B. P. | Association of pediatric asthma severity with exposure to common household dust allergens | 2009 |
| GÉorski, P.,Tarkowski, M. | Non specific environmental factors and asthma development | 1992 |
| Geradts, Joseph,Kratzke, Robert A.,Niehans, Gloria A.,Lincoln, Clint E. | Immunohistochemical detection of the cyclin-dependent kinase inhibitor 2/multiple tumor suppressor gene 1 (CDKN2/MTS1) product p16-INK4A in archival human solid tumors: Correlation with retinoblastoma protein expression | 1995 |
| Geradts, Joseph,Wilentz, Robb E.,Roberts, Helen | utImmunohistochemical detection of the alternate INK4a-encoded tumor suppressor protein p14ARF in archival human cancers and cell lines using commercial antibodies: Correlation with p16INK4a expression | 2001 |
| Gerasin, V. A.,Palamarchuk, G. F.,Kizela, A. P. | [The detection of mast cells in the bronchoalveolar contents in lung diseases] | 1989 |
| Geraut, C.,Cleenewerck, M. B.,Tripodi, D. | Inflammatory occupational skin disease among hairdressers: Diagnosis and prevention | 2008 |
| Géraut, C.,Cleenewerck, M. B.,Tripodi, D. | Inflammatory occupational skin disease among hairdressers: Diagnosis and prevention | 2008 |
| Gergen, P. J.,Togias, A. | Inner City Asthma | 2015 |
| German, J. A.,Harper, M. B. | Environmental control of allergic diseases | 2002 |
| Germanaud, J.,Proffit, V.,Janvoie, B.,Lemarie, E.,Lasfargues, G. | Pneumopathy due to isocyanate hypersensitivity: recognition as an occupational disease | 2003 |
| Germolec, D. R.,Luster, M. I. | Hypersensitivity and Occupational Exposure | 1994 |
| Gershwin, L. J.,Schelegle, E. S.,Gunther, R. A.,Anderson, M. L.,Woolums, A. R.,Larochelle, D. R.,Boyle, G. A.,Friebertshauser, K. E.,Singer, R. S. | A bovine model of vaccine enhanced respiratory syncytial virus pathophysiology | 1998 |
| Gerster, F. M.,Hopf, N. B.,Wild, P. P.,Vernez, D. | Airborne Exposures to Monoethanolamine, Glycol Ethers, and Benzyl Alcohol During Professional Cleaning: A Pilot Study | 2014 |
| Gervais, P.,Diamant Berger, O.,Roux, | Toluene diisocyanate poisoning | 1973 |
| Ghooi, R. B.,Bhide, M. B. | Studies in Anaphylaxis 1. A Model of Anaphylactoid Edema for Screening Anti Anaphylactic Agents | 1981 |
| Ghosh, G.,Manglik, A. K. | Respiratory Illness and Environmental Pollution | 2008 |
| Gibson, P. G.,Allen, C. J.,Yang, J. P.,Wong, B. J.,Dolovich, J.,Denburg, J.,Hargreave, F. E. | Intraepithelial mast cells in allergic and nonallergic asthma. Assessment using bronchial brushings | 1993 |
| Gibson, P. G.,Allen, C. J.,Yang, J. P.,Wong, B. J. O.,Dolovich, J.,Denburg, J.,Hargreave, F. E. | Intraepithelial Mast-Cells in Allergic and Nonallergic Asthma - Assessment Using Bronchial Brushings | 1993 |
| Gibson, P. G.,Dolovich, J.,Denburg, J. A.,Girgis-Gabardo, A.,Hargreave, F. E. | Sputum cell counts in airway disease: a useful sampling technique | 1990 |
| Gibson, P. G.,Girgis-Gabardo, A.,Morris, M. M.,Mattoli, S.,Kay, J. M.,Dolovich, J.,Denburg, J.,Hargreave, F. E. | Cellular characteristics of sputum from patients with asthma and chronic bronchitis | 1989 |
| Gilbert, C. R.,Baram, M.,Cavarocchi, N. C. | "Smoking wet": respiratory failure related to smoking tainted marijuana cigarettes | 2013 |
| Gilbert, N. L.,Guay, M.,Miller, J. D.,Judek, S.,Chan, C. C.,Dales, R. E. | Levels and determinants of formaldehyde, acetaldehyde, and acrolein in residential indoor air in Prince Edward Island, Canada | 2005 |
| Gilbert, S.,McGuire, A. L.,Maghera, S.,Sundaresan, S. R.,Seely, A. J.,Maziak, D. E.,Shamji, F. M.,Villeneuve, P. J. | Randomized trial of digital versus analog pleural drainage in patients with or without a pulmonary air leak after lung resection | 2015 |
| Gilchrist, Harry L.,Matz, Philip B. | The residual effects of warfare gases: I. Chlorine | 1933 |
| Giliberti, D. C.,Brown, L. A. S.,Gauthier, T. W. | Impairment of Alveolar Macrophage Migration in Fetal Alcohol Exposure: An in Vitro Model | 2012 |
| Gillespie-Bennett, J.,Pierse, N.,Wickens, K.,Crane, J.,Nicholls, S.,Shields, D.,Boulic, M.,Viggers, H.,Baker, M.,Woodward, A.,Howden-Chapman, P. | Sources of nitrogen dioxide (NO(2)) in New Zealand homes: findings from a community randomized controlled trial of heater substitutions | 2008 |
| Gilmour, M. I. | Interaction of air pollutants and pulmonary allergic responses in experimental animals | 1995 |
| Ginis, Irene,Mentzer, Steven J.,Faller, Douglas V. | Oxygen tension regulates neutrophil adhesion to human endothelial cells via an LFA-1-dependent mechanism | 1993 |
| Giovannangelo, M.,Gehring, U.,Nordling, E.,Oldenwening, M.,de Wind, S.,Bellander, T.,Almqvist, C.,Heinrich, J.,Hoek, G.,Brunekreef, B. | Childhood cat allergen exposure in three European countries: The AIRALLERG study | 2006 |
| Giri, S. N.,Al-Bayati, M. A.,Du, X.,Schelegle, E.,Mohr, F. C.,Margolin, S. B. | Amelioration of doxorubicin-induced cardiac and renal toxicity by pirfenidone in rats | 2003 |
| Giri, S. N.,Al-Bayati, M. A.,Schelegle, E.,Mohr, F. C.,Margolin, S. B. | Amelioration of doxorubicin-induced cardiac and renal toxicity by pirfenidone in rats | 2004 |
| Girod, S.,Galabert, C.,Pierrot, D.,Boissonnade, M. M.,Zahm, J. M.,Baszkin, A.,Puchelle, E. | Role of Phospholipid Lining on Respiratory Mucus Clearance by Cough | 1991 |
| Gismondi, Angela,Bernardini, Giovanni,Santoni, Angela | NK cells and chemokines | 2010 |
| Gismondi, Angela,Jacobelli, Jordan,Mainiero, Fabrizio,Strippoli, Raffaele,Santoni, Angela | Role of Pyk2 in the control of human NK cell migration | 2001 |
| Giusti, F. | Atopy patch test | 2012 |
| Glazova, T. G.,Ryvkin, A. I.,Laryushkina, R. M.,Dindyaev, S. V. | Changes of the Properties of Platelets and Hemostasis Indices during Persistent Course of Bronchial Asthma in Children | 2011 |
| Gleich, Gerald J.,Leiferman, Kristin | Eosinophils and Anti-Pathogen Host Defense | 2013 |
| Glover, D. C.,DeVries, D. R.,Wright, R. A. | Effects of temperature, salinity and body size on routine metabolism of coastal largemouth bass Micropterus salmoides | 2012 |
| Godderis, L.,Deschuyffeleer, T.,Roelandt, H.,Veulemans, H.,Moens, G. | Exposure to metalworking fluids and respiratory and dermatological complaints in a secondary aluminium plant | 2007 |
| Godish, T. | Formaldehyde and building-related illness | 1981 |
| Godish, T. | Indoor air pollution control | 1989 |
| Goebel, M.,Tan, J.,Bernstein, D. | Contact sensitivity to metal and acrylates in patients undergoing joint replacement | 2014 |
| Gokturk, C.,Sugimoto, H.,Blomgren, B.,Roomans, G. M.,Forsberg-Nilsson, K.,Oreland, L.,Sjoquist, M. | Macrovascular changes in mice overexpressing human semicarbazide-sensitive amine oxidase in smooth muscle cells | 2007 |
| Gold, D. R. | Indoor air pollution | 1992 |
| Gold, D. R.,Wright, R. | Population disparities in asthma | 2005 |
| Gold, Diane R.,Wright, Rosalind | Population disparities in asthma | 2005 |
| Goldberg, Michael,Peshkovsky, Courtney,Shifteh, Ashkan,Al-Awqati, Qais | mu-protocadherin, a novel developmentally regulated protocadherin with mucin-like domains | 2000 |
| Golden, R. | Identifying an indoor air exposure limit for formaldehyde considering both irritation and cancer hazards | 2011 |
| Goldstein, I. F.,Hartel, D.,Andrews, L. R.,Weinstein, A. L. | Indoor air pollution exposures of low-income inner-city residents | 1986 |
| Goller, Franz,Larsen, Ole Naesbye | In situ biomechanics of the syrinx and sound generation in pigeons | 1997 |
| Gomes, C.,Freihaut, J.,Bahnfleth, W. | Resuspension of allergen-containing particles under mechanical and aerodynamic disturbances from human walking | 2007 |
| Gómez Nieves, M.,Dominguez Domínguez, E.,Maghfour Martín, Y.,Jiménez Timón, S.,Ahmida, T.,Hernández Arbeiza, F. | Contact allergic dermatitis to methacrylates | 2012 |
| Goncalves, G. M.,Marinho, D. G.,Almanaca, C. C. J.,Marinho, B. G. | Anti-nociceptive and anti-oedematogenic properties of the hydroethanolic extract of Sidastrum micranthum leaves in mice | 2013 |
| Gonçalves, G. M.,Marinho, D. G.,Almança, C. C. J.,Marinho, B. G. | Anti-nociceptive and anti-oedematogenic properties of the hydroethanolic extract of Sidastrum micranthum leaves in mice | 2013 |
| Goncalves, Gabriela Mastrangelo,Marinho, Diogo Guimaraes,Jorden Almanaca, Carlos Cesar,Marinho, Bruno Guimaraes | Anti-nociceptive and anti-oedematogenic properties of the hydroethanolic extract of Sidastrum micranthum leaves in mice | 2013 |
| Gong, M.,Weschler, C. J.,Liu, L.,Shen, H.,Huang, L.,Sundell, J.,Zhang, Y. | Phthalate metabolites in urine samples from Beijing children and correlations with phthalate levels in their handwipes | 2015 |
| Goodman, M.,Paustenbach, D.,Sipe, K.,Malloy, C. D.,Chapman, P.,Burke, M. L.,Figueroa, R.,Zhao, K.,Exuzides, K. A. | Epidemiologic study of pulmonary obstruction in workers occupationally exposed to ethyl and methyl cyanoacrylate | 2000 |
| Goodman, Simon L.,Grote, Hans Juergen,Wilm, Claudia | Matched rabbit monoclonal antibodies against alpha v-series integrins reveal a novel alpha v beta 3-LIBS epitope, and permit routine staining of archival paraffin samples of human tumors | 2012 |
| Gordian, M. E.,Stewart, A. W.,Morris, S. S. | Evaporative Gasoline Emissions and Asthma Symptoms | 2010 |
| Gordon, I. O.,Krishnan, J. A.,Charbeneau, J.,Mazurek, A.,Hogarth, D. K.,Krausz, T.,Husain, A. N. | Airway inflammation in adult patients with symptoms of asthma or COPD does not distinguish those with fixed airway obstruction | 2009 |
| Gordon, R. E.,Lane, B. P. | Immuno Localization of Myosin and Tropo Myosin in Cells Undergoing Ciliogenesis during Regeneration of Rat Tracheal Epithelium | 1984 |
| Gordon, S.,Tee, R. D.,Lowson, D.,Wallace, J.,Newman Taylor, A. J. | Reduction of airborne allergenic urinary proteins from laboratory rats | 1992 |
| Gordon, S.,Tee, R. D.,Lowson, D.,Wallace, J.,Taylor, A. J. N. | Reduction of Airborne Allergenic Urinary Proteins from Laboratory Rats | 1992 |
| Gordon, T.,Harkema, J. R. | Mucous cell metaplasia in the airways of rats exposed to machining fluids | 1995 |
| Gore, R. | Allergen avoidance: the attraction of charge | 2002 |
| Gore, R. B.,Curbishley, L.,Truman, N.,Hadley, E.,Woodcock, A.,Langley, S. J.,Custovic, A. | Intranasal air sampling in homes: relationships among reservoir allergen concentrations and asthma severity | 2006 |
| Gorgojo, I. E.,de las Heras, M.,Fernandez-Nieto, M.,Cuesta, J.,Sastre, J. | Occupational asthma in a hairdresser due to formaldehyde | 2013 |
| Gorizontova, M. N.,Ozhiganova, V. N. | CYTOLOGICAL PICTURE OF THE BRONCHIAL TREE MUCOUS MEMBRANE IN OCCUPATIONAL BRONCHIAL ASTHMA | 1986 |
| Gorizontova, M. N.,Oziganova, V. N. | Cytological characteristics of the bronchial mucosa in occupational bronchial asthma | 1986 |
| Gorman, W. F. | Medical diagnosis versus legal determination of death | 1985 |
| Gorski, P.,Bilewicz, P.,Krakowiak, A. | [Objective evaluation of respiratory ventilation at work by measuring peak expiratory flow rate (PEFR)] | 1992 |
| Gorski, P.,Krakowiak, A.,Ruta, U. | Nasal and bronchial responses to flour-inhalation in subjects with occupationally induced allergy affecting the airway | 2000 |
| Gorski, P.,Tarkowski, M. | Non specific environmental factors and asthma development | 1992 |
| Gowland, M. H. | Food allergen avoidance - the patient's viewpoint | 2001 |
| Gowland, M. H. | Food allergen avoidance: risk assessment for life | 2002 |
| Goyer, N.,BÇgin, D.,Beaudry, C.,Bouchard, M.,Carrier, G.,LavouÇ, J.,Noisel, N.,GÇrin, M. | [Prevention guide - Formaldehyde in the workplace] | 2006 |
| Grabow, T. S.,Dougherty, P. M. | Cervicomedullary intrathecal injection of morphine produces antinociception in the orofacial formalin test in the rat | 2001 |
| Gradinariu, F.,Danulescu, E.,Danulescu, R.,Margineanu, M.,Croitoru, C.,Cazuc, V. | Blood oxidative markers in glass industry workers and related respiratory outcomes | 2012 |
| Grammer, L. C.,Harris, K. E.,Cugell, D. W.,Patterson, R. | Evaluation of a Worker with Possible Formaldehyde Asthma | 1992 |
| Grammer, L. C.,Harris, K. E.,Cugell, D. W.,Patterson, R. | Evaluation of a worker with possible formaldehyde-induced asthma | 1993 |
| Grammer, L. C.,Patterson, R. | Occupational immunologic lung disease | 1987 |
| Graner, Jill,Kim, Jiyoun,Siddiqui, Javed,Natarajan, Sudha,Remick, Daniel | CO treatment does not decrease the inflammatory response in a marine model of asthma induced by house dust extract | 2005 |
| Grashoff, W. F.,Sont, J. K.,Sterk, P. J.,Hiemstra, P. S.,de Boer, W. I.,Stolk, J.,Han, J.,van Krieken, J. M. | Chronic obstructive pulmonary disease: role of bronchiolar mast cells and macrophages | 1997 |
| Grashoff, Wimfred F. H.,Sont, Jacob K.,Sterk, Peter J.,Hiemstra, Pieter S.,De Boer, Willem I.,Stolk, Jan,Van Krieken, J. Han J. M. | Chronic obstructive pulmonary disease: Role of bronchiolar mast cells and macrophages | 1997 |
| Grashoff, W. F. H.,Sont, J. K.,Sterk, P. J.,Hiemstra, P. S.,deBoer, W. I.,Stolk, J.,vanKrieken, Jhjm | Chronic obstructive pulmonary disease - Role of bronchiolar mast cells and macrophages | 1997 |
| Grasmeijer, Floris,Hagedoorn, Paul,Frijlink, Henderik W.,de Boer, Anne H. | Drug Content Effects on the Dispersion Performance of Adhesive Mixtures for Inhalation | 2013 |
| Green, B.,Nayak, A.,Law, B.,Siegel, P.,Beezhold, D. H. | Production of a methylenediphenyl diisocyanate hapten-]specific monoclonal antibody | 2013 |
| Green, B. J.,Mitakakis, T. Z.,Tovey, E. R. | Allergen detection from 11 fungal species before and after germination | 2003 |
| Green, B. J.,Sercombe, J. K.,Tovey, E. R. | Fungal fragments and undocumented conidia function as new aeroallergen sources | 2005 |
| Green, B. J.,Tovey, E. R.,Beezhold, D. H.,Perzanowski, M. S.,Acosta, L. M.,Divjan, A. I.,Chew, G. L. | Surveillance of fungal allergic sensitization using the fluorescent halogen immunoassay | 2009 |
| Green, Gareth M.,Carolin, Diana | The depressant effect of cigarette smoke on the in vitro antibacterial activity of alveolar macrophages | 1967 |
| Green, L. S.,Chun, L. E.,Patton, A. K.,Sun, X.,Rosenthal, G. J.,Richards, J. P. | Mechanism of inhibition for N6022, a first-in-class drug targeting S-nitrosoglutathione reductase | 2012 |
| Green, L. S.,Chun, L. E.,Patton, A. K.,Sun, X. C.,Rosenthal, G. J.,Richards, J. P. | Mechanism of Inhibition for N6022, a First-in-Class Drug Targeting S-Nitrosoglutathione Reductase | 2012 |
| Green, R.,Simpson, A.,Custovic, A.,Faragher, B.,Chapman, M.,Woodcock, A. | The effect of air filtration on airborne dog allergen | 1999 |
| Green, W.,Woolcock, A. J.,Dowse, G. | House dust mites in blankets and houses in the highlands of Papua New Guinea | 1982 |
| Green-McKenzie, J.,Hudes, D. | Grand rounds: Latex-induced occupational asthma in a surgical pathologist | 2005 |
| Greenblatt, M. | Formaldehyde Toxicology: A Review of Recent Developments | 1987 |
| Greenblatt, M. | Formaldehyde Toxicology: A Review of Recent Research and Regulatory Changes | 1988 |
| Gregoire, A.,Michaud, C.,Gagnon, F. | [Oxygen needs!] | 2008 |
| Grellet, S.,Momas, I.,Seta, N.,Bardet, G.,Achard, S. | Reconstituted human airway epithelium 3D-model to assess the impact of indoor air pollutants on the inflammatory response | 2013 |
| Gresele, P. | Platelets and allergic disorders | 2010 |
| Gridley, D. S.,Mao, X. W.,Tian, J.,Cao, J. D.,Perez, C.,Stodieck, L. S.,Ferguson, V. L.,Bateman, T. A.,Pecaut, M. J. | Genetic and Apoptotic Changes in Lungs of Mice Flown on the STS-135 Mission in Space | 2015 |
| Griffiths, G. D.,Lindsay, C. D.,Allenby, A. C.,Bailey, S. C.,Scawin, J. W.,Rice, P.,Upshall, D. G. | Protection against inhalation toxicity of ricin and abrin by immunisation | 1995 |
| Grigg, Jonathan,Kukielka, Gilbert L.,Berens, Kurt L.,Dreyer, William J.,Entman, Mark L.,Smith, C. Wayne | Induction of intercellular adhesion molecule-1 by lipopolysaccharide in canine alveolar macrophages | 1994 |
| Gross, W. E.,Gross, C. W.,Becker, D.,Moore, D.,Phillips, D. | Modified transnasal endoscopic Lothrop procedure as an alternative to frontal sinus obliteration | 1995 |
| Grover, T. R.,Ackerman, K. G.,Le Cras, T. D.,Jobe, A. H.,Abman, S. H. | Repetitive prenatal glucocorticoids increase lung endothelial nitric oxide synthase expression in ovine fetuses delivered at term | 2000 |
| Gruber, A. D.,Gandhi, R.,Pauli, B. U. | The murine calcium-sensitive chloride channel (mCaCC) is widely expressed in secretory epithelia and in other select tissues | 1998 |
| Grutzmacher, Cathy,Park, SunYoung,Elmergreen, Tammy L.,Tang, Yixin,Scheef, Elizabeth A.,Sheibani, Nader,Sorenson, Christine M. | Opposing effects of bim and bcl-2 on lung endothelial cell migration | 2010 |
| Gu, Yapeng,Shea, Jill,Slattum, Gloria,Firpo, Matthew A.,Alexander, Margaret,Mulvihill, Sean J.,Golubovskaya, Vita M.,Rosenblatt, Jody | Defective apical extrusion signaling contributes to aggressive tumor hallmarks | 2015 |
| Gubitosi, A.,Ruggiero, R.,Docimo, G.,Esposito, A.,Esposito, E.,Villaccio, G.,Foroni, F.,Agresti, M. | Treating rhinophyma: A case report illustrating decortication/vaporization with an 808-nm diode laser | 2012 |
| Guel, M.,Scholz, A.,Dill, D.,Wolf, B. | Telemetric Personal Health Monitoring Systems for Asthma and Chronic Obstructive Pulmonary Disease | 2008 |
| Guenther, U.,Manzke, T.,Wrigge, H.,Utschmann, M.,Zinserling, J.,Putensen, C.,Hoeft, A. | The Counteraction of Opioid-Induced Ventilatory Depression by the Serotonin 1A-Agonist 8-OH-DPAT Does Not Antagonize Antinociception in Rats In Situ and In Vivo | 2009 |
| Gui, W.,Wisnewski, A. V.,Neamtiu, I.,Gurzau, E.,Sparer, J. A.,Stowe, M. H.,Liu, J.,Slade, M. D.,Rusu, O. A.,Redlich, C. A. | Inception cohort study of workers exposed to toluene diisocyanate at a polyurethane foam factory: initial one-year follow-up | 2014 |
| Guidoin, R. G.,Gaylor, J. D. S.,Borsanyi, J. P. | Banked Blood Micro Filtration Part 1 Micro Filter Composed of 5 Poly Urethane Foam Layers with Graded Pore Size | 1976 |
| Guieysse-Pellissier, A. | L'etat reaction-nel: Evolution du tissu lymphoide en reaction | 1932 |
| Guillonneau, B.,Wetzel, O.,Lepage, J. Y.,Vallancien, G.,Buzelin, J. M. | Retroperitoneal laparoscopic nephrectomy: animal and human anatomic studies | 1995 |
| Guirgis, H. M.,Townley, R. G. | Relative Significance of Slow Reacting Substance of Anaphylaxis and Histamine in the Mediation of the in-Vitro Anaphylaxis of Human Tracheo Bronchial Muscles | 1974 |
| Guo, H.,Kwok, N. H.,Cheng, H. R.,Lee, S. C.,Hung, W. T.,Li, Y. S. | Formaldehyde and volatile organic compounds in Hong Kong homes: concentrations and impact factors | 2009 |
| Guo, Jing,Han, Bing,Qin, Longjuan,Li, Bing,You, Huihui,Yang, Jiwen,Liu, Dandan,Wei, Chenxi,Nanberg, Eewa,Bornehag, Carl-Gustaf,Yang, Xu | Pulmonary Toxicity and Adjuvant Effect of Di-(2-exylhexyl) Phthalate in Ovalbumin-Immunized BALB/c Mice | 2012 |
| Guo, J.,Han, B.,Qin, L. J.,Li, B.,You, H. H.,Yang, J. W.,Liu, D. D.,Wei, C. X.,Nanberg, E.,Bornehag, C. G.,Yang, X. | Pulmonary Toxicity and Adjuvant Effect of Di-(2-exylhexyl) Phthalate in Ovalbumin-Immunized BALB/c Mice | 2012 |
| Guo, M.,Pei, X. Q.,Mo, F. F.,Liu, J. L.,Shen, X. Y. | Formaldehyde concentration and its influencing factors in residential homes after decoration at Hangzhou, China | 2013 |
| Guo, P.,Yokoyama, K.,Piao, F. Y.,Sakai, K.,Khalequzzaman, M.,Kamijima, M.,Nakajima, T.,Kitamura, F. | Sick Building Syndrome by Indoor Air Pollution in Dalian, China | 2013 |
| Guo, R. B.,Sun, P. L.,Zhao, A. P.,Gu, J.,Ding, X.,Qi, J.,Sun, X. L.,Hu, G. | Chronic asthma results in cognitive dysfunction in immature mice | 2013 |
| Gupta, K. C.,Ulsamer, A. G.,Preuss, P. W. | Formaldehyde in indoor air: Sources and toxicity | 1982 |
| Gupta, P.,Singh, S.,Kumar, S.,Choudhary, M.,Singh, V. | Effect of Dust Aerosol in Patients with Asthma | 2012 |
| Gupta, R. S.,Zhang, X. Y.,Sharp, L. K.,Shannon, J. J.,Weiss, K. B. | Geographic variability in childhood asthma prevalence in Chicago | 2008 |
| Gupta, R. S.,Zhang, X. Y.,Sharp, L. K.,Shannon, J. J.,Weiss, K. B. | The protective effect of community factors on childhood asthma | 2009 |
| Gupta, S. | Asthma-proofing your home | 2004 |
| Gupta, S.,Dogra, T. D. | Air pollution and human health hazards | 2002 |
| Guzman, Josune,Wang, You-Ming,Kalaycioglu, Oya,Schoenfeld, Barbara,Hamm, Hinrich,Bartsch, Wilfried,Costabel, Ulrich | Increased surfactant protein A content in human alveolar macrophages in hypersensitivity pneumonitis | 1992 |
| Haas, Claudia,Ertel, Christian,Gerhards, Roswitha,Schirrmacher, Volker | Introduction of adhesive and costimulatory immune functions into tumor cells by infection with Newcastle Disease Virus | 1998 |
| Hackney, J. A. C. K. D. | POLLUTANT INTERACTIONS IN ACID FOG--RESPIRATORY EFFECTS | 1987 |
| Hackney, J. A. C. K. D. | POLLUTANT INTERACTIONS IN ACID FOG--RESPIRATORY EFFECTS | 1988 |
| Hackney, J. A. C. K. D. | POLLUTANT INTERACTIONS IN ACID FOG--RESPIRATORY EFFECTS | 1989 |
| Haddad, Z. H.,Marsh, D. G.,Campbell, D. H. | Studies on "allergoids" prepared from naturally occurring allergens. II. Evaluation of allergenicity and assay of antigenicity of formalinized mixed grass pollen extracts | 1972 |
| Hagerhed-Engman, L.,Bornehag, C. G.,Sundell, J. | Building characteristics associated with moisture related problems in 8,918 Swedish dwellings | 2009 |
| Haldar, M. K.,Mehta, D. P. | Formalin asthma, a laboratory acquired hazard among hospital staff | 1994 |
| Hales, S.,Lewis, S.,Slater, T.,Crane, J.,Pearce, N. | Prevalence of adult asthma symptoms in relation to climate in New Zealand | 1998 |
| Halfon, N.,Inkelas, M.,Wood, D. | Nonfinancial Barriers to Care for Children and Youth | 1995 |
| Halken, S. | Prevention of allergic disease in childhood: clinical and epidemiological aspects of primary and secondary allergy prevention | 2004 |
| Hall, J. B.,Hino, G. N.,Inouye, L.,Nada, A.,Lau, C. K. H.,Read, G. W. | Anti Microbial Action of Compound 48-80 4 Methoxy-N-Methyl Benzeneethanamine Formaldehyde Product 2. Mechanism of Action | 1983 |
| Halliday, G. M.,McLachlan, E. M. | 4 Groups of Tyrosine Hydroxylase-Immunoreactive Neurons in the Ventrolateral Medulla of Rats, Guinea-Pigs and Cats Identified on the Basis of Chemistry, Topography and Morphology | 1991 |
| Halliday, J. A.,Henry, R. L.,Hankin, R. G.,Hensley, M. J. | Increased Wheeze but Not Bronchial Hyperreactivity near Power-Stations | 1993 |
| Hallstrom, T.,Muller, S. A.,Morgelin, M.,Mollenkvist, A.,Forsgren, A.,Riesbeck, K. | The Moraxella IgD-binding protein MID/Hag is an oligomeric autotransporter | 2008 |
| Halpin, D. M.,Graneek, B. J.,Turner-Warwick, M.,Newman Taylor, A. J. | Extrinsic allergic alveolitis and asthma in a sawmill worker: case report and review of the literature | 1994 |
| Halttunen, P.,Meurala, H.,Standerskjold-Nordenstam, C. G. | New surgical correction of central airway collapse in an asthmatic patient | 1981 |
| Hamilos, D. L.,Thawley, S. E.,Kramper, M. A.,Kamil, A.,Hamid, Q. A. | Effect of intranasal fluticasone on cellular infiltration, endothelial adhesion molecule expression, and proinflammatory cytokine mRNA in nasal polyp disease | 1999 |
| Hamilton, M. | Formaldehyde and the cancer risk | 1984 |
| Hammad, Y. Y.,Rando, R. J.,Abdel-Kader, H. | Considerations in the design and use of human inhalation challenge delivery systems | 1985 |
| Hammarsten, R.,Hammarsten, J.,Jemsby, P. | Preoperative skin testing of materials used in surgical procedures | 2003 |
| Hammarsten, R.,Hildingh, C. | Swedish patients' perceptions of a preoperative skin test | 2005 |
| Han, Fang,Wang, Xiaoqing,Wang, Zhiyong | Characterization of myosin light chain gene up-regulated in the large yellow croaker immunity by interaction with RanGTPase | 2013 |
| Han, Fang,Wang, Xiao-Qing,Yao, Cui-luan,Wang, Zhi-yong | Molecular characterization of Ran gene up-regulated in large yellow croaker (Pseudosciaena crocea) immunity | 2010 |
| Han, Kang,Zhao, Tingbao,Chen, Xiang,Bian, Na,Yang, Tongtao,Ma, Qiong,Cai, Chengkui,Fan, Qingyu,Zhou, Yong,Ma, Baoan | microRNA-194 suppresses osteosarcoma cell proliferation and metastasis in vitro and in vivo by targeting CDH2 and IGF1R | 2014 |
| Han, P.,Zhao, J.,Liu, S. B.,Yang, C. J.,Wang, Y. Q.,Wu, G. C.,Xu, D. M.,Mi, W. L. | Interleukin-33 mediates formalin-induced inflammatory pain in mice | 2013 |
| Han, Z. A.,Chang, V. W. C.,Zhang, L.,Tse, M. S.,Tan, O. K.,Hildemann, L. M. | Preparation of TiO2-Coated Polyester Fiber Filter by Spray-Coating and Its Photocatalytic Degradation of Gaseous Formaldehyde | 2012 |
| Handharyani, Ekowati,Tsukamoto, Masaya,Tsukamoto, Yasuhiro | Expression of SC1, a cell adhesion molecule, promotes the metastatic activities of the Gallus gallus lymphoblastoid cell line MDCC-MSB1 derived from Marek's disease | 2011 |
| Handzel, Z. T. | Effects of environmental pollutants on airways, allergic inflammation, and the immune response | 2000 |
| Hang, J.,Zhou, W.,Wang, X.,Zhang, H.,Sun, B.,Dai, H.,Su, L.,Christiani, D. C. | Microsomal epoxide hydrolase, endotoxin, and lung function decline in cotton textile workers | 2004 |
| Hang, J.,Zhou, W.,Zhang, H.,Sun, B.,Dai, H.,Su, L.,Christiani, D. C. | TLR4 Asp299Gly and Thr399Ile polymorphisms are very rare in the Chinese population | 2004 |
| Hannaway, P. J.,Roundy, C. | Distribution of Dermatophagoides spp., D. farinae and D. pteronyssinus, antigen in homes of patients with asthma in eastern Massachusetts | 1997 |
| Hannink, J. D. C.,van Helvoort, H. A. C.,Dekhuijzen, P. N. R.,Heijdra, Y. E. | Dynamic Hyperinflation During Daily Activities Does COPD Global Initiative for Chronic Obstructive Lung Disease Stage Matter? | 2010 |
| Hannu, T.,Frilander, H.,Kauppi, P.,Kuuliala, O.,Alanko, K. | IgE-mediated occupational asthma from epoxy resin | 2008 |
| Hardie, E. M.,Spodnick, G. J.,Gilson, S. D.,Benson, J. A.,Hawkins, E. C. | Tracheal rupture in cats: 16 cases (1983-1998) | 1999 |
| Hardy, J. A.,Syrett, K. R. | A radiorespirometric method for evaluating inhibitors of sulphate-reducing bacteria | 1983 |
| Harris, B. | The International Journal of Aromatherapy: Editorial | 2005 |
| Harris, R.,Johnson, M. | Paraben induced contact dermatitis masquerading as severe dyshydrotic eczema | 2009 |
| Harris, W. H.,Marshall, J. S.,Yamashiro, S.,Shaikh, N. | Mast cells of the bovine trachea: Staining characteristics, dispersion techniques and response to secretagogues | 1999 |
| Harrison, J. R.,Campbell, J. S. | Urea Formaldehyde Foam Insulation (UFFI) | 1227 |
| Harrison, P. T. | Creature comforts--living with mites and moulds | 1999 |
| Harrison, R. | Building-Related Disorders | 1995 |
| Hart, B. J.,Whitehead, L. | Ecology of house dust mites in Oxfordshire | 1990 |
| Hartmann, A. L.,Zeitler, M. H.,Mauermayer, R. | Air pollution in animal housing. Medical and hygienic aspects | 1986 |
| Harving, H.,Jensen, J. K.,Dahl, R. | [Occurrence of housedust mites in dwellings. Relation to ventilation and humidity] | 1994 |
| Harving, H.,Korsgaard, J.,Dahl, R. | House-dust mite exposure reduction in specially designed, mechanically ventilated "healthy" homes | 1994 |
| Hashimoto, K. | Toxicology of Acetonitrile | 1991 |
| Hashimoto, M.,Ueda, T.,Higashida, T. | Medico Social Services and Emergency Care for Myasthenia Gravis Patients | 1979 |
| Hassman, P. | The health status of workers handling diphenylmethane 4,4': diisocyanate (MDI) | 1973 |
| Hastie, A. T.,Patrick, H.,Fish, J. E. | Inhibition and Recovery of Mammalian Respiratory Ciliary Function after Formaldehyde Exposure | 1990 |
| Hattori, J.,Fujimura, N.,Kanaya, N.,Okazaki, K.,Namiki, A. | Bronchospasm induced by propofol in a patient with sick house syndrome | 2003 |
| Hausen, B. M.,Schmalle, H. | Quinonoid constituents as contact sensitisers in Australian blackwood (Acacia melanoxylon RBR) | 1981 |
| Haustein, K. O.,Hueller, G.,Sensing, H. | The Determination of Aminopyrine Elimination for Control of the Metabolic Capacity of the Liver in Man | 1982 |
| Hayashi, M.,Huber, G. L. | Quantitative Differences in Goblet Cells in the Tracheal Epithelium of Male and Female Rats | 1977 |
| Hayashi, S.,Mori, I.,Nonoyama, T. | Spontaneous proliferative lesions in the nasopharyngeal meatus of F344 rats | 1998 |
| Hayashi, Shim-Mo,Mori, Ikuo,Nonoyama, Takashi | Spontaneous proliferative lesions in the nasopharyngeal meatus of F344 rats | 1998 |
| Hayashi, S. M.,Mori, I.,Nonoyama, T. | Spontaneous proliferative lesions in the nasopharyngeal meatus of F344 rats | 1998 |
| Hayashi, T.,Haba, R.,Kushida, Y.,Kadota, K.,Katsuki, N.,Bando, K.,Miyai, Y.,Shibuya, S.,Matsunaga, T. | Cytopathologic Characteristics and Differential Diagnostic Considerations of Neuroglial Heterotopia of the Retropharyngeal Space | 2011 |
| Hayatdavoudi, G.,Crapo, J. D.,Miller, F. J.,O'Neil, J. J. | Factors determining degree of inflation in intratracheally fixed rat lungs | 1980 |
| Haydon, R. C. | Addressing the prevalence of respiratory allergy in the home environment | 2003 |
| Hayes, D.,Baker, P.,Kopp, B.,Kirkby, S.,Galantowicz, M.,Astor, T. | Surveillance transbronchial biopsy and histological allograft grading in infant lung and heart-lung transplant recipients | 2012 |
| He, Benfu,Liu, Feiye,Ruan, Jian,Li, Aimin,Chen, Jinzhang,Li, Rong,Shen, Jie,Zheng, Dayong,Luo, Rongcheng | Silencing TRPC1 expression inhibits invasion of CNE2 nasopharyngeal tumor cells | 2012 |
| He, M.,Ichinose, T.,Liu, B. Y.,Song, Y.,Yoshida, Y.,Kobayashi, F.,Maki, T.,Yoshida, S.,Nishikawa, M.,Takano, H.,Sun, G. F. | Silica-carrying particulate matter enhances Bjerkandera adusta-induced murine lung eosinophilia | 2016 |
| He, Wenqian,Mullarkey, Caitlin E.,Duty, J. Andrew,Moran, Thomas M.,Palese, Peter,Miller, Matthew S. | Broadly Neutralizing Anti-Influenza Virus Antibodies: Enhancement of Neutralizing Potency in Polyclonal Mixtures and IgA Backbones | 2015 |
| Heard, B. E. | Fixation of the lung with respect to lung volume and air-apace size | 1962 |
| Heard, B. E.,Nunn, A. J.,Kay, A. B. | Mast cells in human lungs | 1989 |
| Heath, D.,Sasindran, R.,Sufi, P. A. | Primary anastomotic stenting for persistent intraoperative leaking anastomosis during laparoscopic gastric bypass: A proactive approach | 2010 |
| Heath, J.,Kurten, R.,House, S. E.,Sikes, J. D.,Kurten, M.,Jones, S. M.,Kennedy, J. L. | IL-25 causes airway hyper-responsiveness of human precision cut lung slices from donors with asthma | 2016 |
| Hebert-Losier, K.,Jensen, K.,Mourot, L.,Holmberg, H. C. | The influence of surface on the running velocities of elite and amateur orienteer athletes | 2014 |
| Heck, H. D.,Casanova, M. | Pharmacodynamics of formaldehyde: Applications of a model for the arrest of DNA replication by DNA-protein cross-links | 1999 |
| Heck, Henry d'A,Casanova, Mercedes | Pharmacodynamics of formaldehyde: Applications of a model for the arrest of DNA replication by DNA-protein cross-links | 1999 |
| Hedayati, M. T.,Mayahi, S.,Denning, D. W. | A study on Aspergillus species in houses of asthmatic patients from Sari City, Iran and a brief review of the health effects of exposure to indoor Aspergillus | 2009 |
| Heederik, D.,Sigsgaard, T.,Thorne, P. S.,Kline, J. N.,Avery, R.,Bonlokke, J. H.,Chrischilles, E. A.,Dosman, J. A.,Duchaine, C.,Kirkhorn, S. R.,Kulhankova, K.,Merchant, J. A. | Health effects of airborne exposures from concentrated animal feeding operations | 2007 |
| Heilig, Charles,Zaloga, Cecylia,Lee, Min,Zhao, Xiyuan,Riser, Bruce,Brosius, Frank,Cortes, Pedro | Immunogold localization of high-affinity glucose transporter isoforms in normal rat kidney | 1995 |
| Heilmann, Christine | Adhesion Mechanisms of Staphylococci | 2011 |
| Heinrich, J. | Influence of indoor factors in dwellings on the development of childhood asthma | 2010 |
| Heinzerling, A.,Hsu, J.,Yip, F. | Respiratory Health Effects of Ultrafine Particles in Children: a Literature Review | 2016 |
| Heise, R. L.,Li, M.,Stober, V.,Hollingsworth, J. W.,Kraft, M.,Garantziotis, S. | Hyaluronan causes sustained human airway myocyte contraction | 2010 |
| Hémery, M. L.,Florent, F.,Tarodo, P.,Demoly, P. | Asthma in a cosmetics industry worker | 2005 |
| Hemmati, A. A.,Ghorbanzadeh, B.,Behmanesh, M. A. | Potentiation of indomethacin-induced anti-inflammatory response by montelukast in formalin-induced inflammation in rats | 2013 |
| Henderson, B.,Nair, S.,Pallas, J.,Williams, M. A. | Fibronectin: a multidomain host adhesin targeted by bacterial fibronectin-binding proteins | 2011 |
| Henderson, R. F.,Hahn, F. F.,Barr, E. B.,Belinsky, S. A.,Menache, M. G.,Benson, J. M. | Carcinogenicity of inhaled butadiene diepoxide in female B6C3F1 mice and Sprague-Dawley rats | 1999 |
| Hendrick, D. J. | The formaldehyde problem: A clinical appraisal | 1983 |
| Hendrick, D. J. | Occupational lung disease: The clinician's view | 2003 |
| Hendrick, D. J.,Rando, R. J.,Lane, D. J.,Morris, M. J. | Formaldehyde asthma: challenge exposure levels and fate after five years | 1982 |
| Henley, Evelyn E.,McPhaul, John J.,Albert, Salomon N. | Anaphylactic reaction to dextran. Report of a case | 1958 |
| Hennington, M. H.,D'Armini, A. M.,Lemasters, J. J.,Egan, T. M. | Cadaver lungs for transplantation. Effect of ventilation with alveolar gas | 1996 |
| Henretig, F. M. | Preparation for Terrorist Threats: Biologic and Chemical Agents | 2009 |
| Henry, C. J.,Fishbein, L.,Meggs, W. J.,Ashford, N. A.,Schulte, P. A.,Anderson, H.,Osborne, J. S.,Sepkovic, D. W. | Approaches for Assessing Health Risks from Complex-Mixtures in Indoor Air - a Panel Overview | 1991 |
| Henry, R. L.,Abramson, R.,Adler, J. A.,Wlodarcyzk, J.,Hensley, M. J. | Asthma in the Vicinity of Power-Stations .1. A Prevalence Study | 1991 |
| Heraud, M. C.,Herbelin-Wagner, M. L. | Risk factors: environment, exposure to tobacco smoke | 2002 |
| Herbarth, O.,Muller, A.,Rehwagen, M.,Richter, M.,Schlink, U. | Description of the spatiotemporal distribution of chemicals and mould spores in (indoor) air | 2004 |
| Herbst, J.,Stanley, W.,Byard, R. W. | Autopsy Reenactment - A Useful Technique in the Evaluation of Adhesive Tape Asphyxia | 2014 |
| Heroult, Melanie,Schaffner, Florence,Pfaff, Dennis,Prahst, Claudia,Kirmse, Robert,Kutschera, Simone,Riedel, Maria,Ludwig, Thomas,Vajkoczy, Peter,Graeser, Ralph,Augustin, Hellmut G. | EphB4 Promotes Site-Specific Metastatic Tumor Cell Dissemination by Interacting with Endothelial Cell-Expressed EphrinB2 | 2010 |
| Herr, M.,Nikasinovic, L.,Foucault, C.,Le Marec, A. M.,Giordanella, J. P.,Just, J.,Momas, I. | Can early household exposure influence the development of rhinitis symptoms in infancy? Findings from the PARIS birth cohort | 2011 |
| Herr, M.,Nikasinovic, L.,Foucault, C.,Le Marrec, A.,Giordanella, J.,Just, J.,Momas, I. | Risk factors for rhinitis symptoms apart from a cold in infancy | 2010 |
| Hershenson, M. B.,Aghili, S.,Punjabi, N.,Hernandez, C.,Ray, D. W.,Garland, A.,Glagov, S.,Solway, J. | Hyperoxia-induced airway hyperresponsiveness and remodeling in immature rats | 1992 |
| Herth, F.,Eberhardt, R.,Valipour, A.,Stanzel, F.,Bonnet, R.,Behr, J.,Marquette, C.,Kramer, M. | 6-month follow-up in patients with advanced upper lobe predominant heterogeneous emphysema treated with endobronchial lung sealant therapy | 2011 |
| Herth, F. J.,Eberhardt, R.,Gompelmann, D.,Kirsten, A.,Schmidt, B.,Behr, J.,Magnussen, H.,Stanzel, F.,Bonnet, R. | Effective endobronchial volume reduction therapy for emphysema using a synthetic adhesive hydrogel-foam | 2010 |
| Herth, F. J. F. | Endoscopy lung volume reduction | 2013 |
| Herzberg, Frank,Schoening, Michael,Schirner, Michael,Topp, Max,Thiel, Eckhard,Kreuser, Ernst-Dietrich | IL-4 and TNF-alpha induce changes in integrin expression and adhesive properties and decrease the lung-colonizing potential of HT-29 colon carcinoma cells | 1996 |
| Hess, A.,Bloch, W.,Rocker, J.,Addicks, K.,Stennert, E.,Michel, O. | In vitro expression of inducible nitric oxide synthase in the nasal mucosa of guinea pigs after incubation with lipopolysaccharides or cytokines | 1998 |
| Hess, M. M.,Kumazawa, H.,Lamprecht, J. | [Aerodynamics of respiratory flow in the nasopharynx] | 1990 |
| Hess-Kosa, Kathleen | Historic Overview | 2011 |
| Hess-Kosa, Kathleen | Animal Allergenic Dust | 2011 |
| Heudorf, U.,Schumann, M.,Angerer, J.,Exner, M. | Dermal and bronchial symptoms in children: are they caused by PAH containing parquet glue or by passive smoking? | 2005 |
| Hewitt, P. J. | Occupational health problems in processing of X-ray photographic films | 1993 |
| Hey, Ariann,Li, Ming-Shi,Hudson, Michael J.,Langford, Paul R.,Kroll, J. Simon | Transcriptional Profiling of Neisseria meningitidis Interacting with Human Epithelial Cells in a Long-Term In Vitro Colonization Model | 2013 |
| Hey, A.,Li, M. S.,Hudson, M. J.,Langford, P. R.,Kroll, J. S. | Transcriptional Profiling of Neisseria meningitidis Interacting with Human Epithelial Cells in a Long-Term In Vitro Colonization Model | 2013 |
| Heyer, G.,Saba, S.,Adamo, R.,Rush, W.,Soong, G.,Cheung, A.,Prince, A. | Staphylococcus aureus agr and sarA functions are required for invasive infection but not inflammatory responses in the lung | 2002 |
| Heyes, J.,Siebers, R.,Parkes, A.,Pierse, N.,Crane, J. | Beta-(1,3)-glucan levels and its determinants in New Zealand bedrooms | 2009 |
| Higgs, Penelope I.,Myers, Paul S.,Postle, Kathleen | Interactions in the TonB-dependent energy transduction complex: ExbB and ExbD form homomultimers | 1998 |
| Higham, A.,Lea, S.,Simpson, K.,Singh, D. | Lipids in the lung: Respiratory inflammation in COPD | 2012 |
| Higo, Naruhito,Naik, Aarti,Bommannan, D. Bommi,Potts, Russell O.,Guy, Richard H. | Validation of reflectance infrared spectroscopy as a quantitative method to measure percutaneous absorption in vivo | 1993 |
| Hikuma, M.,Hirai, T.,Matsuoka, H. | Respiratory response of cell-based sensors to toxicants measured by using pseudo-random signals | 2005 |
| Hilberg, O. | Objective measurement of nasal airway dimensions using acoustic rhinometry: methodological and clinical aspects | 2002 |
| Hilberg, O.,Jackson, A. C.,Swift, D. L.,Pedersen, O. F. | Acoustic rhinometry: evaluation of nasal cavity geometry by acoustic reflection | 1989 |
| Hill, D. J.,Thompson, P. J.,Stewart, G. A.,Carlin, J. B.,Nolan, T. M.,Kemp, A. S.,Hosking, C. S. | The Melbourne house dust mite study: eliminating house dust mites in the domestic environment | 1997 |
| Hill, D. J.,Toleman, M. A.,Evans, D. J.,Villullas, S.,van Alphen, L.,Virji, M. | The variable P5 proteins of typeable and non-typeable Haemophilus influenzae target human CEACAM1 | 2001 |
| Hill, D. J.,Whittles, C.,Virji, M. | A Novel Group of Moraxella catarrhalis UspA Proteins Mediates Cellular Adhesion via CEACAMs and Vitronectin | 2012 |
| Hilton, J.,Dearman, R. J.,Basketter, D. A.,Scholes, E. W.,Kimber, I. | Experimental assessment of the sensitizing properties of formaldehyde | 1996 |
| Hines, C. J.,Waters, M. A.,Larsson, L.,Petersen, M. R.,Saraf, A.,Milton, D. K. | Characterization of endotoxin and 3-hydroxy fatty acid levels in air and settled dust from commercial aircraft cabins | 2003 |
| Hinze, S.,Bergmann, K. C.,Lowenstein, H.,Hansen, G. N. | Cow hair allergen (Bos d 2) content in house dust: correlation with sensitization in farmers with cow hair asthma | 1997 |
| Hiramoto, R. M.,Galisteo, A. J.,do Nascimento, N.,de Andrade, H. F. | 200 Gy sterilised Toxoplasma gondii tachyzoites maintain metabolic functions and mammalian cell invasion, eliciting cellular immunity and cytokine response similar to natural infection in mice | 2002 |
| Hirashima, M.,Ueno, M.,Saita, N.,Takamura, S.,Watanabe, K.,Tsurufuji, S.,Adachi, M. | Induction of an Eosinophil Chemotactic Factor Production from T-Lymphocytes by a B-Cell Lymphoma Line | 1993 |
| Hirota, J. A.,Gold, M. J.,Hiebert, P. R.,Parkinson, L. G.,Wee, T.,Smith, D.,Hansbro, P. M.,Carlsten, C.,VanEeden, S.,Sin, D. D.,McNagny, K. M.,Knight, D. A. | The Nucleotide-Binding Domain, Leucine-Rich Repeat Protein 3 Inflammasome/IL-1 Receptor I Axis Mediates Innate, but Not Adaptive, Immune Responses after Exposure to Particulate Matter under 10 mu m | 2015 |
| Hirsch, T. | Indoor allergen exposure in west and East Germany: a cause for different prevalences of asthma and atopy? | 2000 |
| Hirsch, T.,Range, U.,Walther, K. U.,Hederer, B.,Lassig, S.,Frey, G.,Leupold, W. | Prevalence and determinants of house dust mite allergen in East German homes | 1998 |
| Hirshman, C. A.,Austin, D. R.,Klein, W.,Hanifin, J. M.,Hulbert, W. | Increased metachromatic cells and lymphocytes in bronchoalveolar lavage fluid of dogs with airway hyperreactivity | 1986 |
| Hislop, A.,Muir, D. C.,Jacobsen, M.,Simon, G.,Reid, L. | Postnatal growth and function of the pre-acinar airways | 1972 |
| Hjorth, N.,Roed-Petersen, J. | Occupational protein contact dermatitis in food handlers | 1976 |
| Ho, Jennifer E.,Yeghiazarians, Yerem | A 27 year-old man from 'the Valley' with shortness of breath | 2007 |
| Ho, L. A.,Kuschner, W. G. | Respiratory Health in Home and Leisure Pursuits | 2012 |
| Hoang, Mai P.,Ross, Karen F.,Dawson, D. Brian,Scheuermann, Richard H.,Rogers, Beverly Barton | Human herpesvirus-6 and sudden death in infancy: Report of a case and review of the literature | 1999 |
| Hochman, D. J.,Collaco, C.,Brooks, E. G. | Examination of toxicological effects of aldehyde air pollutants on mast cells (RBL-2H3) | 2006 |
| Hochstedler, C. M.,Leidinger, M. R.,Maher-Sturm, M. T.,Gibson-Corley, K. N.,Meyerholz, D. K. | Immunohistochemical detection of arginase-I expression in formalin-fixed lung and other tissues | 2013 |
| Hodges, Robin R.,Dartt, Darlene A. | Tear film mucins: Front line defenders of the ocular surface; comparison with airway and gastrointestinal tract mucins | 2013 |
| Hodgson, M. J.,Kreiss, K. | Building-Associated Diseases: An Update | 1986 |
| Hoebee, B.,Rietveld, E.,Bont, L.,van Oosten, M.,Hodemaekers, H. M.,Nagelkerke, N. J. D.,Neijens, H. J.,Kimpen, J. L. L.,Kimman, T. G. | Association of severe respiratory syncytial virus bronchiolitis with interleukin-4 and interleukin-4 receptor alpha polymorphisms | 2003 |
| Hoegh, T.,Schwartz, A. R.,Parrish, D. A.,Bijwadia, J. S. | Use of respiratory impedance sensing as respiratory effort sensor for identification of obstructive events | 2009 |
| Hoek, Kristen Lynn,Duffy, Lynn B.,Cassell, Gail H.,Atkinson, T. Prescott | Mycoplasma pneumoniae-induced IL-4 release from rodent mast cells is dependent upon an adhesive phenotype | 2001 |
| Hoekstra, M.,Schuttelaar, M. L.,Coenraads, P. J. | Anaphylaxis, contact urticaria, and allergic asthma caused by persulfates in hair bleaching products | 2010 |
| Hoffmann, H. D.,Schupp, T. | Evaluation of Consumer Risk Resulting from Exposure against Diphenylmethane-4,4 '-Diisocyanate (Mdi) from Polyurethane Foam | 2009 |
| Hofmann, A. | Thoracic Surgery for Pleural Diseases | 1988 |
| Hogaard, N. V. | P79-AsthmaVent-effect of mechanical ventilation on asthmacontrol in house dust mite allergic children with asthma | 2014 |
| Högberg, J.,Larsson, K.,Albin, M.,Järvholm, B.,Montelius, J.,Ott, M. G.,Jolly, A. T. | Letter to the editor: Comments on "respiratory effects of toluene diisocyanate in the workplace: A discussion of exposure-response relationships" (multiple letters) | 2005 |
| Holder, U.,Schmidt, D. E.,Stupperich, E.,Fuchs, G. | Autotrophic Synthesis of Activated Acetic-Acid from 2 Carbon Dioxide in Methanobacterium-Thermoautotrophicum 3. Evidence for Common 1-Carbon Precursor Pool and the Role of Corrinoid | 1985 |
| Holguin, F.,Flores, S.,Ross, Z.,Cortez, M.,Molina, M.,Molina, L.,Rincon, C.,Jerrett, M.,Berhane, K.,Granados, A.,Romieu, I. | Traffic-related exposures, airway function, inflammation, and respiratory symptoms in children | 2007 |
| Holm, A. N.,Guerin, L.,Rao, S.,Attaluri, A.,Ince, M. N.,Schey, R. | Increased nuclear localization of interleukin 33 in eosinophilic esophagitis | 2011 |
| Holtman, J. R.,Crooks, P. A.,Johnson-Hardy, J.,Wala, E. P. | Antinociceptive effects and toxicity of morphine-6-O-sulfate sodium salt in rat models of pain | 2010 |
| Homatas, J.,Bryant, L.,Eiseman, B. | Time limits of cadaver lung viability | 1968 |
| Homma, Masato,Oka, Kitaro,Kobayashi, Hitoshi,Niitsuma, Tomoyuki,Yamamoto, Shinobu,Itoh, Hisao | Liquid chromatographic determination of magnolol in urine collected from volunteers after a single dose of Saiboku-To, an oriental herbal medicine for bronchial asthma | 1993 |
| Hong, Zhen-Yu,Eun, Sung Ho,Park, Kwangwoo,Choi, Won Hoon,Lee, Jung Il,Lee, Eun-Jung,Lee, Ji Min,Story, Michael D.,Cho, Jaeho | Development of a small animal model to simulate clinical stereotactic body radiotherapy-induced central and peripheral lung injuries | 2014 |
| Hong, Z. Y.,Eun, S. H.,Park, K.,Choi, W. H.,Lee, J. I.,Lee, E. J.,Lee, J. M.,Story, M. D.,Cho, J. | Development of a small animal model to simulate clinical stereotactic body radiotherapy-induced central and peripheral lung injuries | 2014 |
| Hoppentocht, M.,Hagedoorn, P.,Frijlink, H. W.,de Boer, A. H. | Technological and practical challenges of dry powder inhalers and formulations | 2014 |
| Hopper, K. E.,Wood, P. R.,Nelson, D. S. | Macrophage Heterogeneity | 1979 |
| Hornung, D. E.,Leopold, D. A.,Youngentob, S. L.,Sheehe, P. R.,Gagne, G. M.,Thomas, F. D.,Mozell, M. M. | Airflow patterns in a human nasal model | 1987 |
| Horohov, D. W.,Dunham, J.,Liu, C.,Betancourt, A.,Stewart, J. C.,Page, A. E.,Chambers, T. M. | Characterization of the in situ immunological responses to vaccine adjuvants | 2015 |
| Horwitz, M. A. | Cell Mediated Immunity in Legionnaires Disease | 1983 |
| Host, A.,Halken, S. | Can we apply clinical studies to real life? Evidence-based recommendations from studies on development of allergic diseases and allergy prevention | 2002 |
| Hou, Q. L.,Jiang, H. Q.,Zhang, X.,Guo, C.,Huang, B.,Wang, P.,Wang, T. P.,Wu, K. Y.,Li, J. A.,Gong, Z. F.,Du, L. B.,Liu, Y.,Liu, L.,Chen, C. | Nitric oxide metabolism controlled by formaldehyde dehydrogenase (fdh, homolog of mammalian GSNOR) plays a crucial role in visual pattern memory in Drosophila | 2011 |
| Hougaard, M.,Menné, T.,Søsted, H. | Occupational asthma in a hairdresser caused by persulfate salts | 2012 |
| Houin, P.,Veress, L. A.,Hendry-Hofer, T. B.,Loader, J. E.,Rioux, J.,Garlick, R.,White, C. W. | Intratracheal heparin improves morbidity and mortality associated with acute sulfur mustard analog inhalation | 2013 |
| Hovda, J. T.,Bellinger, C.,Miles, M. C. | Review of endoscopic lung volume reduction interventions | 2014 |
| Hovener, B. | [Measurements of cuff pressure of different low pressure cuffed endotracheal tubes under mechanical ventilation (author's transl)] | 1977 |
| Hrncir, D. E. | You're the flight surgeon. Isocyanates are the most common cause of occupational asthma | 1990 |
| Hsu, Ming-Che,Shun, Chia-Tung,Liu, Chia-Ming | Increased epithelial cell proliferation in nasal polyps | 2002 |
| Hsu, N. Y.,Lee, C. C.,Wang, J. Y.,Li, Y. C.,Chang, H. W.,Chen, C. Y.,Bornehag, C. G.,Wu, P. C.,Sundell, J.,Su, H. J. | Predicted risk of childhood allergy, asthma, and reported symptoms using measured phthalate exposure in dust and urine | 2011 |
| Htut, Tin,Burgess, Ian F.,Maunder, John W.,Basham, Elizabeth | A pilot study on the effect of one room mechanical ventilation with heat recovery (MVHR) units on house dust mite populations and Der p 1 levels in laboratory simulated bedrooms and on ambient conditions in an occupied bedroom in Cambridge, UK | 1996 |
| Hu, Q. H.,Chen, S. X.,Luo, F. Y.,Zhu, G. Y. | [Protection of cadaver lungs of non-heart-beating donor lung transplantation in rats] | 2008 |
| Hu, Y.,Wu, Y. Y.,Xia, X. J.,Wu, Z.,Liang, W. Q.,Gao, J. Q. | Development of drug-in-adhesive transdermal patch for alpha-asarone and in vivo pharmacokinetics and efficacy evaluation | 2010 |
| Hua, Xiao-Ying,Chen, Ping,Hwang, Jai-Hyun,Yaksh, Tony L. | Antinociception induced by civamide, an orally active capsaicin analogue | 1997 |
| Hua, X. Y.,Chen, P.,Hwang, J.,Yaksh, T. L. | Antinociception induced by civamide, an orally active capsaicin analogue | 1997 |
| Hua, X. Y.,Chen, P.,Hwang, J. H.,Yaksh, T. L. | Antinociception induced by civamide, an orally active capsaicin analogue | 1997 |
| Huang, C. H.,Hoang, Y.,Minn, K.,Godwin, A.,Chien, J. | Genomic medicine using NexGen sequencing to personalized treatment of metastatic adenoid cystic carcinoma (ADCC) | 2013 |
| Huang, J.,Wang, X. P.,Chen, B. M.,Ueda, A.,Aoyama, K.,Matsushita, T. | Immunological effects of toluene diisocyanate exposure on painters | 1991 |
| Huang, J.,Wang, X. P.,Ueda, A.,Aoyama, K.,Chen, B. M.,Matsushita, T. | Allergologic evaluation for workers exposed to toluene diisocyanate | 1991 |
| Huang, Ziwei,Kumar, Santosh,Choi, Won-Tak,Madani, Navid,Dong, Chang-Zhi,Liu, Dongxiang,Wang, Jun,An, Jing,Sodroski, Joseph G. | A new class of chemokine analogs as useful research tools to study chemokine receptor function and promising therapeutic agents | 2004 |
| Huber, K. R.,Sahota, P. S.,Kapeghian, J.,Hsu, H. H.,Amemiya, K.,Arthur, A. T.,Tripp, S. L.,Traina, V. M. | Lack of neuronal vacuolation and necrosis in monkeys treated with Selfotel (CGS 19755), a competitive N-methyl-D-aspartate receptor antagonist | 1997 |
| Huffman, L. J.,Judy, D. J.,Frazer, D.,Shapiro, R. E.,Castranova, V.,Billie, M.,Dedhia, H. V. | Inhalation of toluene diisocyanate is associated with increased production of nitric oxide by rat bronchoalveolar lavage cells | 1997 |
| Hughes, J. F.,Gaunt, L. F.,Gaynor, P. T. | Electrostatic targeting for allergen removal and pest control applications | 2002 |
| Hughes, R. A.,Sufka, K. J. | Morphine Hyperalgesic Effects on the Formalin Test in Domestic-Fowl (Gallus-Gallus) | 1991 |
| Hulbert, A. J.,Hudson, J. W. | Thyroid Function in a Hibernator Spermophilus-Tridecemlineatus | 1976 |
| Hulin, M.,Annesi-Maesano, I. | Allergic diseases in children and farming environment | 2010 |
| Hulin, M.,Annesi-Maesano, I.,Caillaud, D. | Indoor air quality at school and allergy and asthma among schoolchildren. Differences between rural and urban areas | 2011 |
| Hulin, M.,Simoni, M.,Viegi, G.,Annesi-Maesano, I. | Respiratory health and indoor air pollutants based on quantitative exposure assessments | 2012 |
| Hull, L. C.,Burket, C.,Higgins, C.,Ryder, E. F. | Dig - 1, a giant member of the immunoglobulin superfamily, is involved in adhesion of neuronal processes during development in C. elegans | 2003 |
| Hullan, L.,Trezl, L.,Szarvas, T.,Csiba, A. | The hydrazine derivative aminoguanidine inhibits the reaction of tetrahydrofolic acid with hydroxymethylarginine biomolecule | 1998 |
| Humlicek, Alicia L.,Pang, Liyi,Look, Dwight C. | Modulation of airway inflammation and bacterial clearance by epithelial cell ICAM-1 | 2004 |
| Humlicek, A. L.,Pang, L. Y.,Look, D. C. | Modulation of airway inflammation and bacterial clearance by epithelial cell ICAM-1 | 2004 |
| Humphrey, J. H.,Porter, R. R. | An investigation on rabbit antibodies by the use of partition chromatography | 1956 |
| Humphries, M. J.,Olden, K.,Yamada, K. M. | A Synthetic Peptide from Fibronectin Inhibits Experimental Metastasis of Murine Melanoma Cells | 1986 |
| Hung, S. H.,Tseng, H. C.,Tsai, W. H.,Lin, H. H.,Cheng, J. H.,Chang, Y. M. | Care for Asthma via Mobile Phone (CAMP) | 2007 |
| Hunt, L. W.,Colby, T. V.,Weiler, D. A.,Sur, S.,Butterfield, J. H. | Immunofluorescent Staining for Mast-Cells in Idiopathic Pulmonary Fibrosis - Quantification and Evidence for Extracellular Release of Mast-Cell Tryptase | 1992 |
| Hunt, T. C.,Campbell, A. M.,Robinson, C.,Holgate, S. T. | Structural and Secretory Characteristics of Bovine Lung and Skin Mast Cells Evidence for the Existence of Heterogeneity | 1991 |
| Hurley, T. H. O. M. A. S. D. | Inhibition of s-nitrosoglutathione reductase for the treatment of asthma | 2008 |
| Husain, A. N.,Hessel, R. G. | Neonatal pulmonary hypoplasia: an autopsy study of 25 cases | 1993 |
| Huss, K.,Vessey, J. A.,Mason, P.,Aschenbrenner, D. S.,Huss, R. W. | Controlling allergies by assessing risks in the home | 1996 |
| Huss, R. W.,Huss, K.,Squire, E. N., Jr.,Carpenter, G. B.,Smith, L. J.,Salata, K.,Hershey, J. | Mite allergen control with acaricide fails | 1994 |
| Huss-Marp, J.,Eberlein-Konig, B.,Breuer, K.,Mair, S.,Ansel, A.,Darsow, U.,Kramer, U.,Mayer, E.,Ring, J.,Behrendt, H. | Influence of short-term exposure to airborne Der p 1 and volatile organic compounds on skin barrier function and dermal blood flow in patients with atopic eczema and healthy individuals | 2006 |
| Huynh, C. K.,Duc, T. V. | Size selective isocyanate aerosols personal air sampling using porous plastic foams | 2009 |
| Hvid, M.,Vestergaard, C.,Christensen, G.,Kemp, K.,Deleuran, B.,Deleuran, M. | Interleukin-25 in atopic dermatitis | 2009 |
| Hwabejire, J.,Nembhard, C.,Siram, S.,Cornwell, E.,Greene, W. | Homeless and in shock: morbidity and mortality in the homeless after traumatic hemorrhagic shock | 2014 |
| Hwang, David,Sholl, Lynette,Shivdasani, Priyanka,Dong, Fei | NKX2-1 Loss of Function Mutations in Mucinous Lung Adenocarcinoma | 2015 |
| Hynes, H. P.,Brugge, D.,Osgood, N. D.,Snell, J.,Vallarino, J.,Spengler, J. | Investigations into the indoor environment and respiratory health in Boston public housing | 2005 |
| Hyvarinen, A.,Sebastian, A.,Pekkanen, J.,Larsson, L.,Korppi, M.,Putus, T.,Nevalainen, A. | Characterizing microbial exposure with ergosterol, 3-hydroxy fatty acids, and viable microbes in house dust: determinants and association with childhood asthma | 2007 |
| Ibrahim, B. S.,da Silva, C. M.,Barioni, E. D.,Correa-Costa, M.,Drewes, C. C.,Camara, N. O. S.,Tavares-de-Lima, W.,Farsky, S. H. P.,Lino-dos-Santos-Franco, A. | Formaldehyde inhalation during pregnancy abolishes the development of acute innate inflammation in offspring | 2015 |
| Ichikawa, K.,Miura, R. | Influenza Virus Depresses the Pfc Response of Mice by Affecting T-Cell Function | 1987 |
| Ichiura, H.,Kitaoka, T.,Tanaka, H. | Removal of indoor pollutants under UV irradiation by a composite TiO2-zeolite sheet prepared using a papermaking technique | 2003 |
| Iftikhar, I. H.,McGuire, F.,Moizuddin, M.,Lewis, A.,Christ, C.,Musani, A. | Efficacy of bronchoscopic lung volume reduction: A meta-analysis | 2014 |
| Igietseme, Joseph U.,Uriri, Ijindah M.,Hawkins, Ray,Rank, Roger G. | Integrin-mediated epithelial-T cell interaction enhances nitric oxide production and increased intracellular inhibition of Chlamydia | 1996 |
| Iikura, Y.,Uchiyama, H.,Akimoto, K.,Ebisawa, M.,Sakaguchi, N.,Tsubaki, T.,Ishizu, H.,Kabayama, H.,Yagi, K.,Saito, H.,Miura, K.,Onda, T. | Pharmacokinetics and Pharmacodynamics of the Tulobuterol Patch, Hn-078, in Childhood Asthma | 1995 |
| Iizawa, Y.,Kitamoto, N.,Hiroe, K.,Nakao, M. | Streptococcus pneumoniae in the nasal cavity of mice causes lower respiratory tract infection after airway obstruction | 1996 |
| Ijpma, G.,Lauzon, A. M.,Martin, J. G. | The orientation of airway smooth muscle bundles in asthma | 2015 |
| Ikoma, T.,Ito, T.,Okudela, K.,Hayashi, H.,Yazawa, T.,Kitamura, H. | Modulation of the expression of the Cip/Kip family of cyclin-dependent kinase inhibitors in foetal developing lungs of hamsters | 2001 |
| Illing, H. P. A. | Is working in greenhouses healthy? Evidence concerning the toxic risks that might affect greenhouse workers | 1997 |
| Illum, P.,Grymer, L.,Hilberg, O. | Nasal packing after septoplasty | 1992 |
| Im, H.,Oh, S.,Khim, J. Y.,Mun, J.,Kim, Y.,Huh, Y.,Kang, H. S.,Kim, E. M.,Lee, E.,Sul, D. | Toxicoproteomic biomarkers in plasma proteins of rats exposed to formaldehyde | 2005 |
| Imbus, H. R. | Clinical evaluation of patients with complaints related to formaldehyde exposure | 1985 |
| Impellizzeri, Daniela,Cuzzocrea, Salvatore | Targeting selectins for the treatment of inflammatory diseases | 2014 |
| Ingenito, E. P.,Wood, D. E.,Utz, J. P. | Bronchoscopic lung volume reduction in severe emphysema | 2008 |
| Ingram, J. M.,Heymann, P. W. | Environmental Controls in the Management of Asthma | 1993 |
| Innocenti, A.,Romeo, R.,Mariano, A. | Asthma and systemic toxic reaction due to cabreuva (Myrocarpus fastigiatus Fr. All.) wood dust | 1991 |
| Inoue, M.,Ishibashi, Y.,Nogawa, H.,Yasue, T. | L-Carbocisteine promotes the phagocytosis of apoptotic cells by alveolar macrophages | 2010 |
| Ioachim, E.,Assimakopoulos, D.,Goussia, A. C.,Peschos, D.,Skevas, A.,Agnantis, N. J. | Glycoprotein CD44 expression in benign, premalignant and malignant epithelial lesions of the larynx: An immunohistochemical study including correlation with Rb, p53, Ki-67 and PCNA | 1999 |
| Ira, M. | 'Physician heal thyself' occupational health for health care workers | 2001 |
| Irwin, R. S.,Boulet, L. P.,Cloutier, M. M.,Fuller, R.,Gold, P. M.,Hoffstein, V.,Ing, A. J.,McCool, F. D.,O'Byrne, P.,Poe, R. H.,Prakash, U. B. S.,Pratter, M. R.,Rubin, B. K. | Managing cough as a defense mechanism and as a symptom - A consensus panel report of the American College of Chest Physicians | 1998 |
| Ishida, Y.,Sakano, Y.,Ohki, S.,Muraoka, A.,Aizawa, K.,Misawa, Y. | A case of ascending aortic pseudoaneurysm in a patient with aortic replacement | 2012 |
| Ishikawa, Y.,Fujita, A.,Honjo, I. | Nasal Mucociliary Clearance in Patients with Chronic Middle Ear Infection | 1991 |
| Ishizaki, T.,Ikemori, R.,Fukuda, T.,Saito, A. | Fragility of Skin Mast Cells Due to Adhesion of Immuno Globulin E in Allergic Patients Measured by Challenge of Compound 48-80 P Methoxyphenyl Methylamine-Formaldehyde Product Solutions | 1982 |
| Isik, A. H.,Guler, I.,Sener, M. U. | A Low-Cost Mobile Adaptive Tracking System for Chronic Pulmonary Patients in Home Environment | 2013 |
| Itescu, S.,Dalton, J.,Zhang, H. Z.,Winchester, R. | Tissue infiltration in a CD8 lymphocytosis syndrome associated with human immunodeficiency virus-1 infection has the phenotypic appearance of an antigenically driven response | 1993 |
| Ito, K.,Sakamoto, T.,Hayashi, Y.,Morishita, M.,Shibata, E.,Sakai, K.,Takeuchi, Y.,Torii, S. | Role of tachykinin and bradykinin receptors and mast cells in gaseous formaldehyde-induced airway microvascular leakage in rats | 1996 |
| Itoh, M.,Okazaki, Y.,Ikeda, K.,Furukawa, K.,Ohtsubo, S.,Itoh, T. | Partial aortic root remodelling for fistula between the non-coronary sinus and the right atrium | 2008 |
| Iwata, T.,Iida, S.,Hanada, S.,Inoue, H.,Morikawa, Y.,Inoue, K.,Kobayashi, Y.,Miura, T. | Transbronchial occlusion of a malignant bronchopleural fistula with cyanoacrylate glue | 2011 |
| Jaakkola, J. J. K.,Hwang, B. F.,Jaakkola, M. S. | Home Dampness and Molds as Determinants of Allergic Rhinitis in Childhood: A 6-Year, Population-based Cohort Study | 2010 |
| Jaakkola, N.,Ruotsalainen, R.,Jaakkola, J. J. | What are the determinants of children's exposure to environmental tobacco smoke at home? | 1994 |
| Jabbari, F. | Leukocyte adhesion deficiency | 2013 |
| Jablonski, I. | Wearable Interrupter Module for Home-Based Applications in a Telemedical System Dedicated to Respiratory Mechanics Measurements | 2011 |
| Jablonski, I.,Glomb, G.,Guszkowski, T.,Kasprzak, B.,Pekala, J.,Polak, A. G.,Stepien, A. F.,Swierczynski, Z.,Mroczka, J. | Internal Validation of a Telemedical System for Monitoring Patients with Chronic Respiratory Diseases | 2010 |
| Jachak, A.,Lai, S. K.,Hida, K.,Suk, J. S.,Markovic, N.,Biswal, S.,Breysse, P. N.,Hanes, J. | Transport of metal oxide nanoparticles and single-walled carbon nanotubes in human mucus | 2012 |
| Jackman, Janet,Chen, Yongmei,Huang, Arthur,Moffat, Barbara,Scheer, Justin M.,Leong, Steven R.,Lee, Wyne P.,Zhang, Juan,Sharma, Navneet,Lu, Yanmei,Iyer, Suhasini,Shields, Robert L.,Chiang, Nancy,Bauer, Michele C.,Wadley, Diana,Roose-Girma, Merone,Vandlen, Richard,Yansura, Daniel G.,Wu, Yan,Wu, Lawren C. | Development of a Two-part Strategy to Identify a Therapeutic Human Bispecific Antibody That Inhibits IgE Receptor Signaling | 2010 |
| Jackson, M.,Scott, R. | Different patterns of cytokine induction in cultures of respiratory syncytial (RS) virus-specific human T-H cell lines following stimulation with RS virus and RS virus proteins | 1996 |
| Jacob, R. E.,Amidan, B. G.,Soelberg, J.,Minard, K. R. | In vivo MRI of altered proton signal intensity and T2 relaxation in a bleomycin model of pulmonary inflammation and fibrosis | 2010 |
| Jacob, S. E.,Yang, A.,Herro, E.,Zhang, C. | Contact Allergens in a Pediatric Population: Association with Atopic Dermatitis and Comparison with Other North American Referral Centers | 2010 |
| Jacobs, D. E.,Kelly, T.,Sobolewski, J. | Linking public health, housing, and indoor environmental policy: Successes and challenges at local and federal agencies in the United States | 2007 |
| Jacobs, J. H.,Krop, E. J.,de Wind, S.,Spithoven, J.,Heederik, D. J. | Endotoxin levels in homes and classrooms of Dutch school children and respiratory health | 2012 |
| Jacobsen, I.,Christensen, B.,Baelum, J.,Bjerring, N.,Moeller, U.,Sherson, D. | Epoxy and delayed asthma | 2015 |
| Jaffar, J.,Tan, X.,Black, J. L.,Oliver, B. G.,Corte, T.,Argraves, W. S.,Twal, W. O.,Wolters, P.,Burgess, J. K. | Fibulin-1 is increased in the blood of idiopathic pulmonary fibrosis patients | 2012 |
| Jaffar, J.,Tan, X.,Black, J. L.,Oliver, B. G.,Corte, T.,Wolters, P.,Argraves, W. S.,Twal, W. O.,Burgess, J. K. | The serum level of fibulin-1 is elevated in idiopathic pulmonary fibrosis | 2012 |
| Jaffar, J.,Tjin, G.,Unger, S.,Black, J. L.,Oliver, B. G.,Burgess, J. K. | The extracellular matrix protein fibulin-1 is increased in patients with IPF | 2013 |
| Jaffar, J.,Unger, S.,Corte, T.,Wolters, P. J.,Richeldi, L.,Cerri, S.,Argraves, W.,Oliver, B. G.,Black, J. L.,Burgess, J. K. | Levels of fibulin-1 in the lung and serum are increased in fibrotic interstitial lung disease | 2013 |
| Jagels, R. | Health hazards of natural and introduced chemical components of boatbuilding woods | 1985 |
| Jahnsen, F. L.,Brandtzaeg, P.,Halstensen, T. S. | Monoclonal-Antibody Eg2 Does Not Provide Reliable Immunohistochemical Discrimination between Resting and Activated Eosinophils | 1994 |
| Jakobsson, K.,Rannug, A.,Alexandrie, A. K.,Rylander, L.,Hagmar, L. | Airway symptoms and lung function in pipe-layers exposed to MDI-based polyurethane pyrolysis products and welding fumes do not relate to host polymorphism for CYP1A1, GSTM1 and NAT2 | 1997 |
| Jaksztat, E.,Holz, O.,Paasch, K.,Kelly, M. M.,Hargreave, F. E.,Cox, G.,Magnussen, H.,Jorres, R. A. | Effect of freezing of sputum samples on flow cytometric analysis of lymphocyte subsets | 2004 |
| James, A.,Pearce-Pinto, G.,Hillman, D. | Effects of lung volume and surface forces on maximal airway smooth muscle shortening | 1994 |
| James, A.,Pinto, G. P.,Hillman, D. | Effects of Lung-Volume and Surface Forces on Maximal Airway Smooth-Muscle Shortening | 1994 |
| James, A. L.,Pare, P. D.,Hogg, J. C. | Effects of lung volume, bronchoconstriction, and cigarette smoke on morphometric airway dimensions | 1988 |
| Jan, R. L.,Chen, S. H.,Chang, H. Y.,Yeh, H. J.,Shieh, C. C.,Wang, J. Y. | Asthma-like syndrome in school children after accidental exposure to xylene and methylene diphenyl diisocyanate | 2008 |
| Janjic, Bratislav M.,Lu, Ganwei,Pimenov, Alexei,Whiteside, Theresa L.,Storkus, Walter J.,Vujanovic, Nikola L. | Innate direct anticancer effector function of human immature dendritic cells. I. Involvement of an apoptosis-inducing pathway | 2002 |
| Januskevicius, A.,Lavinskiene, S.,Gosens, R.,Hoppenot, D.,Kalinauskaite-Zukauske, V.,Janulaityte, I.,Tamasauskiene, L.,Sakalauskas, R.,Malakauskas, K. | Integrins regulate eosinophil adhesion to the airway smooth muscle cells in asthma patients | 2015 |
| Jared, C.,Toledo, R. C.,Antoniazzi, M. M. | A histological study of the integument of Corythomantis greeningi (Amphibia, Anura, Hylidae) | 1995 |
| Jarvis, D. | The European Community Respiratory Health Survey II | 2002 |
| Javed, A. P.,Whimster, W. F.,Deverell, M. H.,Cookson, M. J. | Distribution of alveolar wall per unit volume in the human lung | 1994 |
| Jaye, David L.,Parkos, Charles A. | Neutrophil migration across intestinal epithelium | 2000 |
| Jemionek, J. F.,Contreras, T. J.,French, J. E.,Shields, L. J. | Technique for Increased Granulocyte Recovery from Human Whole Body by Counterflow Centrifugation Elutriation Part 1 in-Vitro Analyses | 1979 |
| Jentzsch, N. S.,Camargos, P. A.,de Melo, E. M. | Compliance with environmental control measures in the homes of children and adolescents with asthma | 2007 |
| Jeon, S. H.,Jeon, E. H.,Lee, J. Y.,Kim, Y. S.,Yoon, H. J.,Hong, S. P.,Lee, J. H. | The potential of interleukin 12 receptor beta 2 (IL12RB2) and tumor necrosis factor receptor superfamily member 8 (TNFRSF8) gene as diagnostic biomarkers of oral lichen planus (OLP) | 2015 |
| Jephcote, C.,Chen, H. B. | Geospatial analysis of naturally occurring boundaries in road-transport emissions and children's respiratory health across a demographically diverse cityscape | 2013 |
| Jeppsson, M. C.,Mortstedt, H.,Ferrari, G.,Jonsson, B. A. G.,Lindh, C. H. | Identification of covalent binding sites of ethyl 2-cyanoacrylate, methyl methacrylate and 2-hydroxyethyl methacrylate in human hemoglobin using LC/MS/MS techniques | 2010 |
| Jerrett, M.,McConnell, R.,Wolch, J.,Chang, R.,Lam, C.,Dunton, G.,Gilliland, F.,Lurmann, F.,Islam, T.,Berhane, K. | Traffic-related air pollution and obesity formation in children: a longitudinal, multilevel analysis | 2014 |
| Jetmalani, K.,Young, P. M.,Smith, T.,Stewart, P.,Traini, D. | Micronized drug powders in binary mixtures and the effect of physical properties on aerosolisation from combination drug dry powder inhalers | 2013 |
| Jie, Y.,Ismail, N. H.,Jie, X.,Isa, Z. M. | Do indoor environments influence asthma and asthma-related symptoms among adults in homes?: a review of the literature | 2011 |
| Jindal, S. K. | Management and Prevention of Chronic Bronchitis at the Primary Level of Health Care in India | 2009 |
| Jindal, S. K. | Relationship of household air pollution from solid fuel combustion with tuberculosis? | 2014 |
| Joad, J. P.,Ji, C.,Kott, K. S.,Bric, J. M.,Pinkerton, K. E. | In utero and postnatal effects of sidestream cigarette smoke exposure on lung function, hyperresponsiveness, and neuroendocrine cells in rats | 1995 |
| Joad, J. P.,Ji, C. M.,Kott, K. S.,Bric, J. M.,Pinkerton, K. E. | In-Utero and Postnatal Effects of Sidestream Cigarette-Smoke Exposure on Lung-Function, Hyperresponsiveness, and Neuroendocrine Cells in Rats | 1995 |
| Johansson, E.,Reponen, T.,Vesper, S.,Levin, L.,Lockey, J.,Ryan, P.,Bernstein, D. I.,Villareal, M.,Khurana Hershey, G. K.,Schaffer, C.,Lemasters, G. | Microbial content of household dust associated with exhaled NO in asthmatic children | 2013 |
| Johansson, M. W.,Annis, D. S.,Mosher, D. F. | alpha(M)beta(2) Integrin-Mediated Adhesion and Motility of IL-5-Stimulated Eosinophils on Periostin | 2013 |
| Johansson, M. W.,Khanna, M.,Mosher, D. F. | Alterations in the polarized morphology of interleukin-5-stimulated eosinophils upon adhesion to periostin | 2014 |
| Johansson, M. W.,Lye, M. H.,Barthel, S. R.,Duffy, A. K.,Annis, D. S.,Mosher, D. F. | Eosinophils adhere to vascular cell adhesion molecule-1 via podosomes | 2004 |
| John, Rijo M.,Glantz, Stanton A. | It is time to make smokefree environments work in India | 2007 |
| Johnson, D. R.,Dunlap, A.,McFeeley, P.,Gaffney, J.,Busick, B. | Cricothyrotomy performed by prehospital personnel: a comparison of two techniques in a human cadaver model | 1993 |
| Johnson, L.,Montgomery, J. B.,Schneider, J. P.,Townsend, H. G.,Ochs, M.,Singh, B. | Morphometric examination of the equine adult and foal lung | 2014 |
| Johnson, L.,Montgomery, J. B.,Schneider, J. P.,Townsend, H. G. G.,Ochs, M.,Singh, B. | Morphometric Examination of the Equine Adult and Foal Lung | 2014 |
| Johnson, M.,MacNeill, M.,Grgicak-Mannion, A.,Nethery, E.,Xu, X. H.,Dales, R.,Rasmussen, P.,Wheeler, A. | Development of temporally refined land-use regression models predicting daily household-level air pollution in a panel study of lung function among asthmatic children | 2013 |
| Johnson, Mahlon D.,Vito, Fran,O'Connell, Mary J. | Mesothelin expression in the leptomeninges and meningiomas | 2008 |
| Johnson, Teresa R.,Graham, Barney S. | Secreted respiratory syncytial virus G glycoprotein induces interleukin-5 (IL-5), IL-13, and eosinophilia by an IL-4-independent mechanism | 1999 |
| Johnson, T. R.,Graham, B. S. | Contribution of respiratory syncytial virus G antigenicity to vaccine-enhanced illness and the implications for severe disease during primary respiratory syncytial virus infection | 2004 |
| Johnson, Teresa R.,Teng, Michael N.,Collins, Peter L.,Graham, Barney S. | Respiratory syncytial virus (RSV) G glycoprotein is not necessary for vaccine-enhanced disease induced by immunization with formalin-inactivated RSV | 2004 |
| Johnson, Teresa Rene,Varga, Steven M.,Braciale, Thomas J.,Graham, Barney S. | Respiratory syncytial virus (RSV) G-induced vaccine enhanced disease, but not FI-RSV-induced disease, is mediated by Vbeta14+ T cells | 2003 |
| Jolly, H.,Muls, A. C.,Andreyev, H. J. | Efficacy of Endoscopically Applied 5% Intra-Rectal Formalin to Treat Radiation-Induced Rectal Bleeding | 2013 |
| Joly, L. M.,Guyomarc'h, L.,Damm, C.,Dureuil, B.,Veber, B. | [Reliability of pulsatile saturometry in patients with shock. Digital standard sensors are not reliable for facial measurement!] | 2011 |
| Jones, A. P. | Asthma and domestic air quality | 1998 |
| Jones, A. P. | Asthma and the home environment | 2000 |
| Jones, C. T.,Roebuck, M. M.,Walker, D. W.,Johnston, B. M. | The role of the adrenal medulla and peripheral sympathetic nerves in the physiological responses of the fetal sheep to hypoxia | 1988 |
| Jones, M.,Hoover, R.,Meyrick, B. | Endotoxin Enhancement of Lymphocyte Adherence to Cultured Sheep Lung Microvascular Endothelial-Cells | 1992 |
| Jones, P. W. | Prediction of the Acoustic Performance of Small Poroelastic Foam Filled Mufflers: A Case Study | 2010 |
| Jones, R.,Recer, G. M.,Hwang, S. A.,Lin, S. | Association between indoor mold and asthma among children in Buffalo, New York | 2011 |
| Jones, R. C. M.,Hughes, C. R.,Wright, D.,Baumer, J. H. | Early house moves, indoor air, heating methods and asthma | 1999 |
| Jones, R. N.,Rando, R. J.,Glindmeyer, H. W.,Foster, T. A.,Hughes, J. M.,O'Neil, C. E.,Weill, H. | Abnormal lung function in polyurethane foam producers. Weak relationship to toluene diisocyanate exposures | 1992 |
| Jones, R. N.,Rando, R. J.,Glindmeyer, H. W.,Foster, T. A.,Hughes, J. M.,Oneil, C. E.,Weill, H. | Abnormal Lung-Function in Polyurethane Foam Producers - Weak Relationship to Toluene Diisocyanate Exposures | 1992 |
| Joshi, Sachindra,Gupte, Sachin | Lung ACE is Not Involved in The Progression of Pulmonary Arterial Hypertension | 2015 |
| Jovanovic, K.,Siebeck, M.,Gropp, R. | The route to pathologies in chronic inflammatory diseases characterized by T helper type 2 immune cells | 2014 |
| Jovanovic, M.,Vucicevic, B.,Turanjanin, V.,Zivkovic, M.,Spasojevic, V. | Investigation of indoor and outdoor air quality of the classrooms at a school in Serbia | 2014 |
| Jovanovic, S.,Felder-Kennel, A.,Gabrio, T.,Kouros, B.,Link, B.,Maisner, V.,Piechotowski, I.,Schick, K. H.,Schrimpf, M.,Weidner, U.,Zollner, I.,Schwenk, M. | Indoor fungi levels in homes of children with and without allergy history | 2004 |
| Juchet, A.,Chabbert-Broue, A.,Piot, M. | Relations between childhood asthma and environmental factors | 2002 |
| Jude, J. A.,Koziol-White, C.,Scala, J.,Ouyang, Y.,Jester, W.,Maute, C.,Dalton, P.,Panettieri, R. A. | Formaldehyde exposure induces anti-oxidant response in human airway smooth muscle (HASM) cells | 2014 |
| Juen, J.,Cheng, Q.,Prieto-Centurion, V.,Krishnan, J. A.,Schatz, B. | Health Monitors for Chronic Disease by Gait Analysis with Mobile Phones | 2014 |
| Julia, J. C.,Martorell, A.,Ventas, P.,Cerda, J. C.,Torro, I.,Carreira, J.,Guinot, E.,Sanz, J.,Alvarez, V. | Lepidoglyphus destructor acarus in the urban house environment | 1995 |
| Jung, C. H.,Lee, J. Y.,Cho, C. H.,Kim, C. J. | Anti-asthmatic action of quercetin and rutin in conscious guinea-pigs challenged with aerosolized ovalbumin | 2008 |
| Jung, Jihye,Shin, Jung Hee | Effect of Opuntia Ficus-Indica Extract on Anti-Inflammatory in Murine Macrophages | 2010 |
| Jung, K. H.,Perzanowski, M.,Rundle, A.,Moors, K.,Yan, B.,Chillrud, S. N.,Whyatt, R.,Camann, D.,Perera, F. P.,Miller, R. L. | Polycyclic aromatic hydrocarbon exposure, obesity and childhood asthma in an urban cohort | 2014 |
| Jung, Woon-Won,Kim, Eun-Mi,Lee, Eun-Hee,Yun, Hee-Jung,Ju, Hyang-Ran,Jeong, Moon-Jin,Hwang, Kwang-Woo,Sul, Donggeun,Kang, Hyung-Sik | Formaldehyde exposure induces airway inflammation by increasing eosinophil infiltrations through the regulation of reactive oxygen species production | 2007 |
| Jung, W. W.,Kim, E. M.,Lee, E. H.,Yun, H. J.,Ju, H. R.,Jeong, M. J.,Hwang, K. W.,Sul, D.,Kang, H. S. | Formaldehyde exposure induces airway inflammation by increasing eosinophil infiltrations through the regulation of reactive oxygen species production | 2007 |
| Just, J.,Nisakinovic, L.,Laoudi, Y.,Grimfeld, A. | Air pollution and asthma in children | 2006 |
| Jusufovic, E.,Sejdinovic, R. | Relationship of VEGF and p16 expression in tumor and tumor surrounding tissue to spirometry tests and survival outcomes of non-small-cell lung cancer patients | 2013 |
| Kaaria, K.,Hirvonen, A.,Norppa, H.,Piirila, P.,Vainio, H.,Rosenberg, C. | Exposure to 4,4 '-methylenediphenyl diisocyanate (MDI) during moulding of rigid polyurethane foam: determination of airborne MDI and urinary 4,4 '-methylenedianiline (MDA) | 2001 |
| Kaczmarek, U.,Jaworski, A. | Molar-incisor hypomineralisation - Etiology, prevalence, clinical picture and treatment - Review | 2014 |
| Kadivar, S.,Belsito, D. V. | Occupational Dermatitis in Health Care Workers Evaluated for Suspected Allergic Contact Dermatitis | 2015 |
| Kadota, J.,Mizunoe, S.,Mito, K.,Mukae, H.,Yoshioka, S.,Kawakami, K.,Koguchi, Y.,Fukushima, K.,Kon, S.,Kohno, S.,Saito, A.,Uede, T.,Nasu, M. | High plasma concentrations of osteopontin in patients with interstitial pneumonia | 2005 |
| Kagamimori, S.,Naruse, Y.,Kakiuchi, H.,Yamagami, T.,Sokejima, S.,Matsubara, I.,BiLiFu,,Katoh, T. | Does an allergy skin test on school-children predict respiratory symptoms in adulthood? | 1996 |
| Kagawa, Jun,Nohara, Michiko,Pan, Guowei,Qu, Cheng-yi,Yang, Zhi Min | Comparison of respiratory symptoms between schoolchildren in China and Japan | 2001 |
| Kagawa, J.,Nohara, M.,Pan, G.,Qu, C. Y.,Zhi Min, Yang | Comparison of respiratory symptoms between schoolchildren in China and Japan | 2001 |
| Kai, W.,Xu, Y.,Yang, G. T.,Ke, K.,Yan, Z.,Yi, C. | Pilot study on up-regulation of NGF-mRNA of mice induced by Formaldehyde | 2007 |
| Kajiya, T.,Kuroda, A.,Hokonohara, D.,Tei, C. | Heart failure caused by hookworm infection possibly associated with organic food consumption | 2006 |
| Kakar, P. N.,Roy, P. M.,Pant, V.,Das, J. | Anesthesia for joint replacement surgery: Issues with coexisting diseases | 2011 |
| Kakazo, Mexy,Lehmann, Deborah,Coakley, Keith,Gratten, Helen,Saleu, Gerard,Taime, John,Riley, Ian D.,Alpers, Michael P. | Mortality rates and the utilization of health services during terminal illness in the Asaro Valley, Eastern Highlands Province, Papua New Guinea | 1999 |
| Kakubari, I.,Shinkai, N.,Kawakami, R.,Uruno, A.,Takayasu, T.,Yamauchi, H.,Takayama, S.,Takayama, K. | Formulation and evaluation of ethylene-vinyl acetate copolymer matrix patches containing formoterol fumarate | 2006 |
| Kalima, T. V.,Saloniemi, H.,Rahko, T. | Experimental Regional Enteritis in Pigs | 1976 |
| Kalliny, M. I.,Brisolara, J. A.,Glindmeyer, H.,Rando, R. | A survey of size-fractionated dust levels in the U.S. wood processing industry | 2008 |
| Kamata, E.,Nakadate, M.,Ogawa, Y.,Kaneko, T.,Kurokawa, Y.,Yukawa, M. | Acute inhalation toxicity study of formaldehyde in rats: Effect of vapor on the pulmonary surfactant | 1996 |
| Kameda, H.,Nunome, C.,Monma, Y. | Comparison of Effects of Labetalol and Propranolol on the Respiratory Organ from Aspects of Their Effects on Histamine Asthma and Histamine Releasing Activity in Mice and Rats | 1982 |
| Kanerva, L.,Tarvainen, K.,Pinola, A.,Leino, T.,Granlund, H.,Estlander, T.,Jolanki, R.,Forstrom, L. | A Single Accidental Exposure May Result in a Chemical Burn, Primary Sensitization and Allergic Contact Dermatitis | 1994 |
| Kang-Jae, S.,Young-Chun, G.,Wu-Chul, S.,Ki-Seok, K.,Jin, S. H. | Cadaveric study of positional relationship between the ethmoidal foramens using facial bony landmarks | 2016 |
| Kapu, S. D.,Ngwai, Y. B.,Kayode, O.,Akah, P. A.,Wambebe, C.,Gamaniel, K. | Anti-inflammatory, analgesic and anti-lymphocytic activities of the aqueous extract of Crinum giganteum | 2001 |
| Karakitsios, S.,Asikainen, A.,Garden, C.,Semple, S.,De Brouwere, K.,Galea, K. S.,Sánchez-Jiménez, A.,Gotti, A.,Jantunen, M.,Sarigiannis, D. | Integrated exposure for risk assessment in indoor environments based on a review of concentration data on airborne chemical pollutants in domestic environments in Europe | 2015 |
| Karjalainen, A.,Martikainen, R.,Klaukka, T.,Saarinen, K.,Uitti, J. | Risk of asthma among Finnish patients with occupational rhinitis | 2003 |
| Karkhanis, V.,Joshi, J. M. | Cement dust exposure-related emphysema in a construction worker | 2011 |
| Karmali, Priya Prakash,Brunquell, Chris,Tram, Hau,Ireland, Shubha Kale,Ruoslahti, Erkki,Biliran, Hector | Metastasis of Tumor Cells Is Enhanced by Downregulation of Bit1 | 2011 |
| Kase, Y.,Masato, N.,Abe, K.,Tanaka, T. | [Precision and problems in interpretation of acoustic rhinometry in cases of nasal mass lesion] | 1995 |
| Kashiwabara, K.,Nakamura, H. | Analgesic-induced asthma caused by 2.0% ketoprofen adhesive agents, but not by 0.3% agents | 2001 |
| Kasper, Katherine J.,Zeppa, Joseph J.,Wakabayashi, Adrienne T.,Xu, Stacey X.,Mazzuca, Delfina M.,Welch, Ian,Baroja, Miren L.,Kotb, Malak,Cairns, Ewa,Cleary, P. Patrick,Haeryfar, S. M. Mansour,McCormick, John K. | Bacterial Superantigens Promote Acute Nasopharyngeal Infection by Streptococcus pyogenes in a Human MHC Class II-Dependent Manner | 2014 |
| Kastner, P. E.,Casset, A.,Pons, F. | Formaldehyde interferes with airway epithelium integrity and functions in a dose- and time-dependent manner | 2010 |
| Kastner, Pierre Edouard,Le Calve, Stephane,Zheng, Wuyin,Casset, Anne,Pons, Francoise | A dynamic system for single and repeated exposure of airway epithelial cells to gaseous pollutants | 2013 |
| Kastner, P. E.,Le Calve, S.,Zheng, W. Y.,Casset, A.,Pons, F. | A dynamic system for single and repeated exposure of airway epithelial cells to gaseous pollutants | 2013 |
| Kasuya, Yuji,Fujimoto, Keiji,Miyamoto, Masaki,Kawaguchi, Haruma | Activation of human neutrophils by Arg-Gly-Asp-Ser immobilized on microspheres | 1994 |
| Katayama, N.,Fujimura, M.,Yasui, M.,Ogawa, H.,Nakao, S. | Hypersensitivity pneumonitis and bronchial asthma attacks caused by environmental fungi | 2008 |
| Katilmis, H.,Ozturkcan, S.,Basoglu, S.,Aslan, H.,Ilknur, A. E.,Erdogan, N. K.,Uluc, M. E.,Tayfun, M. A.,Dundar, R.,Guvenc, I. A. | New technique for the treatment of bilateral vocal cord paralysis: Vocal and ventricular fold lateralization using crossing sutures with thyroplasty technique | 2010 |
| Katotomichelakis, M.,Tantilipikorn, P.,Holtappels, G.,De Ruyck, N.,Feng, L.,Van Zele, T.,Muangsomboon, S.,Jareonchasri, P.,Bunnag, C.,Danielides, V.,Cuvelier, C. A.,Hellings, P. W.,Bachert, C.,Zhang, N. | Inflammatory patterns in upper airway disease in the same geographical area may change over time | 2013 |
| Katyal, J.,Gupta, Y. K. | Dopamine Release is Involved in Antinociceptive Effect of Theophylline | 2012 |
| Katz, Y.,Verleger, H.,Barr, J.,Rachmiel, M.,Kiviti, S.,Kuttin, E. S. | Indoor survey of moulds and prevalence of mould atopy in Israel | 1999 |
| Kaushik, M.,Gupta, H.,Gupta, P. K.,Jarrett, J. E.,Schuller, D.,Morrow, L. E. | A 28-year-old woman with recurrent pneumomediastinum | 2009 |
| Kawamoto, H.,Takeno, S.,Yajin, K. | [Tumor necrosis factor-alpha and interferon-gamma modulate in vitro expression of nitric oxide synthase in human nasal epithelial cells] | 2002 |
| Kawano, T.,Matsuse, H.,Fukahori, S.,Tsuchida, T.,Nishino, T.,Fukushima, C.,Kohno, S. | Acetaldehyde at a Low Concentration Synergistically Exacerbates Allergic Airway Inflammation as an Endocrine-Disrupting Chemical and as a Volatile Organic Compound | 2012 |
| Kayaba, H.,Meguro, H.,Muto, H.,Kamada, Y.,Adachi, T.,Yamada, Y.,Kanda, A.,Yamaguchi, K.,Hamada, K.,Ueki, S.,Chihara, J. | Activation of eosinophils by rice-husk dust exposure: a possible mechanism for the aggravation of asthma during rice harvest | 2004 |
| Kayano, K.,Date, H.,Uno, K.,Shimizu, N.,Teramoto, S. | Evaluation of the viability of the canine cadaver lung for transplantation | 1993 |
| Kayser, Klaus,Liewald, Florian,Kremer, Klaus,Tacke, Marcel,Storck, Martin,Faber, Penfield,Bonomi, Philip | Alteration of integrated optical density and intercellular structure after induction chemotherapy and survival in lung carcinoma patients treated surgically | 1994 |
| Kayser, Klaus,Stute, Heike,Tacke, Marcel | Minimum spanning tree, integrated optical density and lymph node metastasis in bronchial carcinoma | 1993 |
| Kayser, K.,Zeilinger, C.,Zeng, F. Y.,Gabius, S.,Gabius, H. J.,Weiser, W. Y. | Detection of the lymphokine migration inhibitory factor in normal and disease-affected lung by antibody and by its major binding protein, the interferon antagonist sarcolectin | 1993 |
| Kazeros, A.,Bleck, B.,Garcia-Medina, L.,Adams, A.,Liu, M.,Tse, D.,Reibman, J. | Thymic stromal lymphopoietin mRNA expression in sputum bronchial epithelial cells in WTC related asthma | 2013 |
| Kazeros, A.,Bleck, B.,Lee, R. A.,Tse, D. B.,Chung, S.,Chiu, A.,Reibman, J. | In situ upregulation of thymic stromal lymphopoietin in sputum epithelial cells in asthma | 2012 |
| Ke, K.,Cao, Y.,Zhu, Y.,Yang, G. T.,Yang, X. | Oxidative stress in GSNO reductase regulation in mice lungs | 2007 |
| Kearn, G. C.,Gowing, R. | Glands and Sensilla Associated with the Haptor of the Gill-Parasitic Monogenean Tetraonchus-Monenteron | 1989 |
| Kecelj, P.,Kecelj-Leskovec, N. | An anaphylactic reaction after treatment of minor burn with latex contained adhesive-free plaster | 2010 |
| Keddem, S.,Barg, F. K.,Glanz, K.,Jackson, T.,Green, S.,George, M. | Mapping the urban asthma experience: Using qualitative GIS to understand contextual factors affecting asthma control | 2015 |
| Keeler, G. J.,Morishita, M.,Young, L. H. | Characterization of complex mixtures in urban atmospheres for inhalation exposure studies | 2005 |
| Keim-Malpass, J.,Johnson, C. R. S.,Quandt, S. A.,Arcury, T. A. | Perceptions of housing conditions among migrant farmworkers and their families: implications for health, safety and social policy | 2015 |
| Keith, I. M.,Pelto-Huikko, M.,Schalling, M.,Hokfelt, T. | Calcitonin Gene-Related Peptide and Its Messenger Rna in Pulmonary Neuroendocrine Cells and Ganglia | 1991 |
| Keith, I. M.,Peltohuikko, M.,Schalling, M.,Hokfelt, T. | Calcitonin Gene-Related Peptide and Its Messenger-Rna in Pulmonary Neuroendocrine Cells and Ganglia | 1991 |
| Kelekis, A. D. | Complications of vertebroplasty | 2010 |
| Keles, N.,Ilicali, C.,Deger, K. | The effects of different levels of air pollution on atopy and symptoms of allergic rhinitis | 1999 |
| Keller-Olaman, S. J.,Eyles, J. D.,Elliott, S. J.,Wilson, K.,Dostrovsky, N.,Jerrett, M. | Individual and neighborhood characteristics associated with environmental exposure | 2005 |
| Kellerhoff, N. M.,Lussi, A. | ["Molar-incisor hypomineralization"] | 2004 |
| Kelly, Don P.,Baker, Simon C.,Trickett, Jim,Davey, Margaret,Murrell, J. Colin | Methanesulphonate utilization by a novel methylotrophic bacterium involves an unusual monooxygenase | 1994 |
| Kelly, Kevin J.,Pearson, Michell L.,Kurup, Viswanath P.,Havens, Peter L.,Byrd, Robert S.,Setlock, Mary A.,Butler, Jay C.,Slater, Jay E.,Grammer, Leslie C. | A cluster of anaphylactic reactions in children with spina bifida during general anesthesia: Epidemiologic features, risk factors, and latex hypersensitivity | 1994 |
| Kelly, K. J.,Pearson, M. L.,Kurup, V. P.,Havens, P. L.,Byrd, R. S.,Setlock, M. A.,Butler, J. C.,Slater, J. E.,Grammer, L. C.,Resnick, A.,Roberts, M.,Jarvis, W. R.,Davis, J. P.,Fink, J. N. | A Cluster of Anaphylactic Reactions in Children with Spina-Bifida during General-Anesthesia - Epidemiologic Features, Risk-Factors, and Latex Hypersensitivity | 1994 |
| Kelly, L. A.,Erwin, E. A.,Platts-Mills, T. A. | The indoor air and asthma: the role of cat allergens | 2011 |
| Kelly, M. M.,Hargreave, F. E.,Cox, G. | A method to preserve sputum for delayed examination | 2003 |
| Kelly, M. M.,Hargreave, F. E.,Cox, G. | Letter to the editor | 2010 |
| Kelly, M. T. | Activation of Guinea-Pig Macrophages by Q Fever Rickettsiae | 1977 |
| Kempton, T. J.,Leng, R. A. | Protein Nutrition of Growing Lambs 1. Responses in Growth and Rumen Function to Supplementation of a Low Protein Cellulosic Diet with Either Urea Casein or Formaldehyde Treated Casein | 1979 |
| Kennedy, J.,Jones, S. M.,Kurten, M.,House, S.,Kurten, R. | Effects of rhinovirus (RV) 39 infection on airway hyper-reactivity (AHR) to histamine and carbachol in human airways | 2014 |
| Kennedy, J. L.,Brown, E.,Kurten, M.,Kurten, R.,Jones, S. M. | Rhinovirus infection induces Th2-promoting innate cytokines in an ex vivo precision cut lung slice model | 2015 |
| Kennedy, J. L.,Jones, S. M.,Kurten, M.,House, S.,Kurten, R. | Effects of rhinovirus (RV) 39 infection on airway hyper-reactivity (AHR) to histamine and carbachol in human airways | 2014 |
| Kern, J. A.,Kron, I. L.,Flanagan, T. L.,Binns, O. A.,Scott, W. W.,Chan, B. B.,Zografakis, J. G.,Tribble, C. G. | Denervation of the immature porcine lung impairs normal airway development | 1993 |
| Kern, J. A.,Kron, I. L.,Flanagan, T. L.,Binns, O. A. R.,Scott, W. W.,Chan, B. B. K.,Zografakis, J. G.,Tribble, C. G. | Denervation of the Immature Porcine Lung Impairs Normal Airway Development | 1993 |
| Kesornsukhon, N. | Epicutaneous immunotherapy for treatment of cockroach allergy | 2013 |
| Kespohl, S.,Schlunssen, V.,Jacobsen, G.,Schaumburg, I.,Maryska, S.,Meurer, U.,Bruning, T.,Sigsgaard, T.,Raulf-Heimsoth, M. | Impact of cross-reactive carbohydrate determinants on wood dust sensitization | 2010 |
| Kessy, V. M. J.,Matovelo, J. A. | Immunohistochemical Characterization and Quantification of Lymphocytes Infiltrating Bovine Lungs in East Coast Fever | 2011 |
| Kettleson, E. M.,Kumar, S.,Reponen, T.,Vesper, S.,Adhikari, A. | Bacteria in House Dust: Influence of Mold and Other Environmental Factors | 2012 |
| Khakberdyev, M. M.,Ishchenko, G. N.,El'Tekova, N. I. | Skin reaction to streptococci in workers suffering allergic diseases | 1966 |
| Khalid, I.,Godfrey, A. M.,Ouellette, D. R. | Chemical pneumonitis and subsequent reactive airways dysfunction syndrome after a single exposure to a household product: A case report | 2009 |
| Khan, A. W.,Ahmed, M. G.,Ramesh, B. | Novel sustained release pulsatile capsules of terbutaline sulphate | 2011 |
| Khan, A. W.,Nersesyan, A.,Knasmuller, S.,Moshammer, H.,Kundi, M. | Nuclear anomalies in exfoliated buccal cells in Pakistani cotton weavers | 2015 |
| Khan, F. | Oklahoma healthy homes initiative | 2011 |
| Khan, S. A.,Hashmi, Z. Y. | Comparison of therapeutic values between leukotriene receptor antagonist (Montelukast) and inhaled glucocorticoide (Beclomethasone propionate) in bronchial asthma of adults | 2008 |
| Khaneshi, F.,Farokhi, F.,Shikhi, S. | Histopathological study in testis of male rat asthma has been treated with theophylline | 2012 |
| Khitrina, L. V.,Drachev, L. A.,Kaulen, A. D.,Chekulaeva, L. N. | Inhibition of Bacterio Rhod Opsin by Formalin and Lanthane | 1982 |
| Khokhlov, V. D. | Calculation of tension exerted on a ligature in incomplete hanging | 2001 |
| Kida, K.,Thurlbeck, W. M. | The effects of beta-aminopropionitrile on the growing rat lung | 1980 |
| Kido, Akira,Krueger, Sabine,Haeckel, Carsten,Roessner, Albert | Inhibitory effect of antisense aminopeptidase N (APN/CD13) cDNA transfection on the invasive potential of osteosarcoma cells | 2003 |
| Kilburn, K. H. | Effects of a hydrochloric acid spill on neurobehavioral and pulmonary function | 1996 |
| Kilburn, K. H. | Indoor air effects after building renovation and in manufactured homes | 2000 |
| Kilburn, K. H. | Effects of diesel exhaust on neurobehavioral and pulmonary functions | 2000 |
| Kilburn, K. H. | Indoor mold exposure associated with neurobehavioral and pulmonary impairment: A preliminary report | 2003 |
| Kilburn, K. H.,Thornton, J. C. | Protracted Neurotoxicity from Chlordane Sprayed to Kill Termites | 1995 |
| Kilburn, S.,Lasserson, T. J.,McKean, M. | Pet allergen control measures for allergic asthma in children and adults | 2003 |
| Kilgour, J. D.,Alexander, D. J.,Reed, C. J. | Development of an in vitro rat nasal epithelial model for predicting upper respiratory tract toxicity | 1998 |
| Kilinc, G.,Uysal, P.,Uzuner, N.,Ellidokuz, H. | Does it possible to prevent side effects of inhaled corticosteroids on teeth by preventive dental programs in children with asthma? | 2014 |
| Kim, Cheol-Woo,Song, Jae-Seok,Ahn, Yeon-Soon,Park, Seung-Hyun,Park, Jung-Won,Noh, Jae-Hoon,Hong, Chein-Soo | Occupational asthma due to formaldehyde | 2001 |
| Kim, C. W.,Song, J. S.,Ahn, Y. S.,Park, S. H.,Park, J. W.,Noh, J. H.,Hong, C. S. | Occupational asthma due to formaldehyde | 2001 |
| Kim, Eunjoo,Maeng, Jin-Hee,Lee, Don Haeng,Kim, Joon Mee | Correlation of Biomarkers and Histological Responses in Manufactured Silver Nanoparticle Toxicity | 2009 |
| Kim, H.,Tse, K.,Levin, L.,Bernstein, D.,Reponen, T.,LeMasters, G.,Lummus, Z.,Horner, A. A. | House dust bioactivities predict skin prick test reactivity for children with high risk of allergy | 2012 |
| Kim, Hye Young,Chang, Ya-Jen,Subramanian, Srividya,Lee, Hyun-Hee,Albacker, Lee A.,Matangkasombut, Ponpan,Savage, Paul B.,McKenzie, Andrew N. J.,Smith, Dirk E.,Rottman, James B.,DeKruyff, Rosemarie H.,Umetsu, Dale T. | Innate lymphoid cells responding to IL-33 mediate airway hyperreactivity independently of adaptive immunity | 2012 |
| Kim, H. Y.,Chang, Y. J.,Subramanian, S.,Lee, H. H.,Albacker, L. A.,Matangkasombut, P.,Savage, P. B.,McKenzie, A. N. J.,Smith, D. E.,Rottman, J. B.,DeKruyff, R. H.,Umetsu, D. T. | Innate lymphoid cells responding to IL-33 mediate airway hyperreactivity independently of adaptive immunity | 2012 |
| Kim, J.,Han, Y.,Ahn, J. H.,Lee, S. I.,Lee, K. H.,Ahn, K. | Short-term effect of airborne formaldehyde on skin barrier function in atopic dermatitis | 2015 |
| Kim, J.,Jeong, M.,Lee, M.,Seo, S. | Effects of simultaneous exposure to formaldehyde and house dust mite on atopic dermatitis in vivo | 2012 |
| Kim, J.,McGill, M.,Mitchel, J.,Park, J. A.,Bi, D.,Qazvini, N. T.,Steward, R. L.,Burger, S.,Khabibullin, D.,Medvetz, D.,Notbohm, J.,Randell, S. H.,Butler, J. P.,Manning, M. L.,Henske, E. P.,Drazen, J. M.,Fredberg, J. J. | Intensified intercellular adhesive forces accompany airway epithelial cell unjamming | 2015 |
| Kim, J.,Natarajan, S.,Vaickus, L. J.,Bouchard, J. C.,Beal, D.,Cruikshank, W. W.,Remick, D. G. | Diesel Exhaust Particulates Exacerbate Asthma-Like Inflammation by Increasing CXC Chemokines | 2011 |
| Kim, Jae-Ryong,Kim, Seong-Yong,Kim, Mi-Jin,Kim, Jung-Hye | Differential expression of proteins related to START checkpoint of the cell cycle in human stomach, lung, cervix and liver cancers | 1997 |
| Kim, J. A.,Vogel, D.,Guimond, G.,Hostler, D.,Wang, H. E.,Menegazzi, J. J. | A randomized, controlled comparison of cardiopulmonary resuscitation performed on the floor and on a moving ambulance stretcher | 2006 |
| Kim, J. H.,Park, J. H.,Byun, S. S.,Lee, C. K. | Embolotherapy of a bronchial artery aneurysm presenting as life-threatening mediastinal hematoma | 2014 |
| Kim, J. L.,Elfman, L.,Mi, Y.,Johansson, M.,Smedje, G.,Norback, D. | Current asthma and respiratory symptoms among pupils in relation to dietary factors and allergens in the school environment | 2005 |
| Kim, J. Y.,Chang, J. | Need for a safe vaccine against respiratory syncytial virus infection | 2012 |
| Kim, J. Y.,Jeong, M. S.,Park, K. Y.,Seo, S. J. | Aggravation of atopic dermatitis-like symptoms by consecutive low concentration of formaldehyde exposure in NC/Nga mice | 2013 |
| Kim, Ki-Hyun,Jahan, Shamin Ara,Kabir, Ehsanul | A review of diseases associated with household air pollution due to the use of biomass fuels | 2011 |
| Kim, Ki-Hyun,Jahan, Shamin Ara,Kabir, Ehsanul | A review on human health perspective of air pollution with respect to allergies and asthma | 2013 |
| Kim, K. H.,Jahan, S. A.,Kabir, E. | A review of diseases associated with household air pollution due to the use of biomass fuels | 2011 |
| Kim, K. H.,Jahan, S. A.,Kabir, E. | A review on human health perspective of air pollution with respect to allergies and asthma | 2013 |
| Kim, N.,Seo, J. B.,Song, K. S.,Chae, E. J.,Kang, S. H. | Semi-automatic measurement of the airway dimension by computed tomography using the full-width-half-maximum method: a study on the measurement accuracy according to the CT parameters and size of the airway | 2008 |
| Kim, S. J.,Lee, K. H.,Kim, S. W.,Cho, J. S.,Park, Y. K.,Shin, S. Y. | Changes in Histological Features of Nasal Polyps in a Korean Population over a 17-year Period | 2013 |
| Kim, S. T.,Cho, K. B.,Kang, I. G. | Preventive effects of polyurethane foam and polyvinyl acetate on bleeding and pain in young patients undergoing conchotomies | 2012 |
| Kim, W. J.,Terada, N.,Nomura, T.,Takahashi, R.,Lee, S. D.,Park, J. H.,Konno, A. | Effect of formaldehyde on the expression of adhesion molecules in nasal microvascular endothelial cells: the role of formaldehyde in the pathogenesis of sick building syndrome | 2002 |
| Kim, Y. J.,Kim, M. S.,Ryu, J. C. | Genotoxicity and identification of differentially expressed genes of formaldehyde in human Jurkat cells | 2005 |
| Kim, Y. M.,Kim, Y. S.,Jeon, S. G.,Kim, Y. K. | Immunopathogenesis of allergic asthma: More than the Th2 hypothesis | 2013 |
| Kimbell, J. S.,Gross, E. A.,Joyner, D. R.,Godo, M. N.,Morgan, K. T. | Application of computational fluid dynamics to regional dosimetry of inhaled chemicals in the upper respiratory tract of the rat | 1993 |
| Kimbell, J. S.,Overton, J. H.,Subramaniam, R. P.,Schlosser, P. M.,Morgan, K. T.,Conolly, R. B.,Miller, F. J. | Dosimetry modeling of inhaled formaldehyde: binning nasal flux predictions for quantitative risk assessment | 2001 |
| Kimbell, J. S.,Subramaniam, R. P. | Use of computational fluid dynamics models for dosimetry of inhaled gases in the nasal passages | 2001 |
| Kimbell, J. S.,Subramaniam, R. P.,Gross, E. A.,Schlosser, P. M.,Morgan, K. T. | Dosimetry modeling of inhaled formaldehyde: comparisons of local flux predictions in the rat, monkey, and human nasal passages | 2001 |
| Kimura, M.,Waki, I.,Kokubo, M. | Inhibition of Compound 48-80 Mediated Histamine Release from Isolated Rat Mast Cells by Oosponol Related Compounds 4 Acyl Iso Coumarins | 1978 |
| Kimura, R.,Kimoto, I.,Takeda, M.,Miyake, M.,Sakamoto, T. | Alteration in airway microvascular leakage induced by sensorineural stimulation in rats exposed to inhaled formaldehyde | 2010 |
| Kinger, N.,Yang, H.,McGlynn, P.,Liu, C. | Pulmonary cement embolism in an older woman with shortness of breath | 2014 |
| Kinoshita, T. | Usefulness of intrapleural administration of a large amount of diluted fibrin glue for intractable pneumothoraces | 1998 |
| Kirshenbaum, A. S.,Fischer, E. R.,O'Brien, K.,Gahl, W. A.,Metcalfe, D. D. | Ultrastructural differences in cutaneous mast cells from patients with Hermansky-Pudlak Syndrome | 2011 |
| Kis, A.,Sutto, Z.,Tamasi, L.,Eszes, N.,Losonczy, G.,Mathe, Z.,Langer, R. M.,Nemeth, A.,Muller, V. | Spontaneous pneumomediastinum after kidney transplantation: case report | 2010 |
| Kita, T.,Fujimura, M.,Myou, S.,Ishiura, Y.,Abo, M.,Katayama, N.,Nishitsuji, M.,Yoshimi, Y.,Nomura, S.,Oribe, Y.,Nakao, S. | Potentiation of allergic bronchoconstriction by repeated exposure to formaldehyde in guinea-pigs in vivo | 2003 |
| Kitaoka, H.,Itoh, H. | Computer-assisted three-dimensional volumetry of the human pulmonary acini | 1992 |
| Kitch, B. T.,Chew, G.,Burge, H. A.,Muilenberg, M. L.,Weiss, S. T.,Platts-Mills, T. A.,O'Connor, G.,Gold, D. R. | Socioeconomic predictors of high allergen levels in homes in the greater Boston area | 2000 |
| Kjaergard, H. K.,Axelsen, P. | Effectiveness of autologous fibrin sealant in experimental lung surgery | 1996 |
| Klaric, M. S.,Varnai, V. M.,Calusic, A. L.,Macan, J. | Occupational exposure to airborne fungi in two Croatian sawmills and atopy in exposed workers | 2012 |
| Kleber, M.,Blaszkewicz, M.,Lucas, S.,Bolt, H. M.,Follmann, W. | Mutagenic effects of cutting fluids and components in the Salmonella typhimurium mutagenicity assay | 2002 |
| Kleber, M.,Follmann, W.,Blaszkewicz, M. | Assessing the genotoxicity of industrial cutting fluids under conditions of use | 2004 |
| Kleeberger, S. R.,Peden, D. | Gene-environment interactions in asthma and other respiratory diseases | 2005 |
| Kleeberger, S. T. E. V. E. N. R. | The Role Of Innate Immunity Genes In Viral Infection and Disease Progression | 2009 |
| Klees, J. E.,Ott, M. G. | Diisocyanates in polyurethane plastics applications | 1999 |
| Kleine-Tebbe, J.,Herold, D. A.,Kunkel, G. | Causal allergy treatment: Specific immunotheraphy with inhaled allergens | 2004 |
| Kleinman, M. T.,Mautz, W. J. | The effects of exercise on dose and dose distribution of inhaled automotive pollutants | 1991 |
| Kleinman, Wayne A.,Richie, John P., Jr. | Determination of thiols and disulfides using high-performance liquid chromatography with electrochemical detection | 1995 |
| Klimek, L.,Werfel, T.,Vogelberg, C.,Jung, K. | Authorized diagnostic test allergens for intracutaneous testing are no longer available in Germany. Allergological textbooks need to be rewritten | 2015 |
| Klink, M.,Bednarska, K.,Blus, E.,Kielbik, M.,Sulowska, Z. | Seasonal changes in activities of human neutrophils in vitro | 2012 |
| Klink, M.,Bednarska, K.,Jastrzembska, K.,Banasik, M.,Sulowska, Z. | Signal transduction pathways affected by nitric oxide donors during neutrophil functional response in vitro | 2007 |
| Klink, M.,Tchorzewski, H.,Sulowska, Z. | Oxidative and adhesive responses of human neutrophils to nitrovasodilators in vitro: the role of protein kinases | 2003 |
| Klypina, N. S.,Plakunov, V. K. | The Role of Metabolic Energy in Regulation of Chlortetracycline Transport in Escherichia-Coli | 1979 |
| Knipping, S.,Holzhausen, H. J.,Riederer, A.,Agha-Mir-Salim, P.,Berghaus, A. | [Immunoelectron microscopic findings in patients with allergic rhinitis] | 2002 |
| Knipping, S.,Holzhausen, H. J.,Riederer, A.,Bloching, M. | [Ultrastructural changes in human nasal mucosa in rhinitis medicamentosa] | 2006 |
| Knox, W. Eugene | Glutathione | 1960 |
| Knudson, C.,Hartwig, S.,Meyerholz, D.,Varga, S. | Th2-independent development of vaccine-enhanced disease associated with formalin-inactivated respiratory syncytial virus immunization (P3182) | 2013 |
| Knudson, C. J.,Hartwig, S. M.,Meyerholz, D. K.,Varga, S. M. | RSV Vaccine-Enhanced Disease Is Orchestrated by the Combined Actions of Distinct CD4 T Cell Subsets | 2015 |
| Kobr, J.,Fremuth, J.,Pizingerova, K.,Fikrlova, S.,Jehlicka, P.,Honomichl, P.,Sasek, L.,Racek, J.,Topolcan, O. | Total Body Response to Mechanical Ventilation of Healthy Lungs: an Experimental Study in Piglets | 2010 |
| Koda, S.,Kumagai, S.,Ohara, H. | Environmental monitoring and assessment of short-term exposures to hazardous chemicals of a sterilization process in hospital working environments | 1999 |
| Koegelenberg, C. F. N.,Slebos, D. J.,Shah, P. L.,Theron, J.,Dheda, K.,Allwood, B. W.,Herth, F. J. F. | Time for the Global Rollout of Endoscopic Lung Volume Reduction | 2015 |
| Koenig, J. Q. | Indoor and Outdoor Pollutants and the Upper Respiratory Tract | 1055 |
| Koenig, J. Q. | Indoor and outdoor pollutants and the upper respiratory tract | 1988 |
| Koenig, J. Q.,Jansen, K.,Mar, T. F.,Lumley, T.,Kaufman, J.,Trenga, C. A.,Sullivan, J.,Liu, L. J.,Shapiro, G. G.,Larson, T. V. | Measurement of offline exhaled nitric oxide in a study of community exposure to air pollution | 2003 |
| Koenig, J. Q.,Larson, T. V.,Hanley, Q. S.,Rebolledo, V.,Dumler, K.,Checkoway, H.,Wang, S. Z.,Lin, D.,Pierson, W. E. | Pulmonary function changes in children associated with fine particulate matter | 1993 |
| Koenig, J. Q.,Mar, T. F.,Allen, R. W.,Jansen, K.,Lumley, T.,Sullivan, J. H.,Trenga, C. A.,Larson, T.,Liu, L. J. | Pulmonary effects of indoor- and outdoor-generated particles in children with asthma | 2005 |
| Kolarik, B.,Bornehag, C. G.,Naydenov, K.,Sundell, J.,Stavova, P.,Nielsen, O. F. | The concentrations of phthalates in settled dust in Bulgarian homes in relation to building characteristic and cleaning habits in the family | 2008 |
| Kolodziejczyk, Piotr,Pejsak, Zygmunt | Biological properties of Mycoplasma hyopneumoniae, agent of mycoplasmal pneumonia in swine | 2004 |
| Kon, O. M.,Sihra, B. S.,Till, S. J.,Corrigan, C. J.,Kay, A. B.,Grant, J. A. | Unstimulated basophils in atopic and nonatopic subjects express intracellular interleukin-4: detection by flow cytometry | 1998 |
| Kondo, H.,Wickins, S. C.,Conway, J. A.,Mallicote, M. F.,Sanchez, L. C.,Agnew, D. W.,Farina, L. L.,Abbott, J. R. | Cranial mediastinal liposarcoma in a horse | 2012 |
| Kondo, Y.,Homma, Y.,Takahashi, S.,Kitamura, T.,Kawabe, K. | Transvaginal ultrasound of urethral sphincter at the mid urethra in continent and incontinent women | 2000 |
| Kong, W. J.,Wu, J. H.,Wang, Y. J.,Yue, J. X.,Zhang, S.,Yu, Y. P. | Dysregulation of E-cadherin in chronic rhinosinusitis with nasal polyps | 2010 |
| Kongerud, J.,Naalsund, A. | Occupational asthma: Occurrence, diagnosis, prognosis and prevention | 1992 |
| Konto-Ghiorghi, Y.,Mairey, E.,Mallet, A.,Dumenil, G.,Caliot, E.,Trieu-Cuot, P.,Dramsi, S. | Dual Role for Pilus in Adherence to Epithelial Cells and Biofilm Formation in Streptococcus agalactiae | 2009 |
| Konya, V.,Philipose, S.,Balint, Z.,Olschewski, A.,Marsche, G.,Sturm, E. M.,Schicho, R.,Peskar, B. A.,Schuligoi, R.,Heinemann, A. | Interaction of eosinophils with endothelial cells is modulated by prostaglandin EP4 receptors | 2011 |
| Koo, H. K.,Vasilescu, D. M.,Scott, A. E.,Katsamenis, O.,Warner, J. A.,Sinclair, I.,Hogg, J. C.,Hackett, T. L. | Micro-CT analysis of paraffin embedded lung tissue: Is small airway obstruction an early feature of COPD? | 2015 |
| Koo, H. K.,Vasilescu, D. M.,Scott, A. E.,Warner, J. A.,Sinclair, I.,Hogg, J. C.,Hackett, T. L. | Microct analysis of paraffin embedded lung tissue: Is small airway obstruction an early feature of COPD? | 2014 |
| Koopman, E.,Craenen, A.,Scholten, S.,Huitink, J. | Visual recognition of airway structures in a circulated patient and in a non-circulated cadaver | 2014 |
| Kooragayala, Keshav,Gotoh, Norimoto,Li, Wei,Nellissery, Jacob,Kaden, Talia R.,Covian-Garcia, Raul,Balaban, Robert,Cogliati, Tiziana,Swaroop, Anand | Changes in mitochondria respiration in degenerating mouse retina identified by a novel ex vivo assay | 2015 |
| Kopferschmitt-Kubler, M. C.,Bessot, J. C.,Charles, P.,Baumeiser-Kapps, M.,Pauli, G. | Isocyanates and Occupational Asthma | 1991 |
| Kopp, S. K.,McKay, R. T.,Moller, D. R.,Cassedy, K.,Brooks, S. M. | Asthma and rhinitis due to ethylcyanoacrylate instant glue | 1985 |
| Korhonen, K.,Liukkonen, T.,Ahrens, W.,Astrakianakis, G.,Boffetta, P.,Burdorf, A.,Heederik, D.,Kauppinen, T.,Kogevinas, M.,Osvoll, P.,Rix, B. A.,Saalo, A.,Sunyer, J.,Szadkowska-Stanczyk, I.,Teschke, K.,Westberg, H.,Widerkiewicz, K. | Occupational exposure to chemical agents in the paper industry | 2004 |
| Kornblatt, Jack A. | The water channel of cytochrome c oxidase: Inferences from inhibitor studies | 1998 |
| Korzycka, B.,Kuprys, I.,Elgalal, A.,Gorski, P.,Kuna, P. | Skin prick tests (spts) in epidemiological study in Polish population | 2001 |
| Koseoglu, Secil,Haynes, Christy L. | Understanding the Fundamentals of Platelet Granular Storage and Release at Single Cell Level | 2011 |
| Kostakioti, M.,Stathopoulos, C. | Functional analysis of the Tsh autotransporter from an avian pathogenic Escherichia coli strain | 2004 |
| Kostalova, D. | [Early allergic reactions in the etiology of occupational dermatoses.] | 2000 |
| Kotchmar, D. J.,Stock, T. H.,Holguin, A. H.,Buffler, P. A. | Exposure estimates of the Houston Area Asthma Study | 1982 |
| Kotin, P.,Falk, H. L. | ATMOSPHERE POLLUTANTS | 1964 |
| Koumoundouros, E.,Van Der Velden, J. L.,Bischof, R. J.,Mareels, I. M. Y.,Snibson, K. J. | Increased baseline airway resistance and smooth muscle content in sheep exposed to repeated challenges of house dust mite | 2010 |
| Kovac, Vladimir | Reproductive behavior and early development of the European mudminnow, Umbra Krameri | 1995 |
| Kowalski, M. L.,Szmidt, M.,Grzelewska-Rzymowska, I.,Rozniecki, J. | The Effect of Aspirin during Aspirin Desensitization on Compound 48-80 4 Methoxy-N-Methylbenzeneethanamine-Formaldehyde Product and Histamine-Induced Skin Responses in Aspirin-Sensitive Asthmatics | 1985 |
| Koyama, Y.,Norose-Toyoda, K.,Hirano, S.,Kobayashi, M.,Ebihara, T.,Someki, I.,Fujisaki, H.,Irie, S. | Type I collagen is a non-adhesive extracellular matrix for macrophages | 2000 |
| Koziol-White, C. J.,Scala, J.,Jester, W.,Jude, J. A.,Maute, C.,Dalton, P.,Panettieri, R. A. | Exposure of human small airways and airway smooth muscle to formaldehyde increases agonist-mediated bronchoconstriction and calcium levels, but has little effect on mediator release | 2013 |
| Kradin, R. L.,McCarthy, K. M.,Gifford, J.,Schneeberger, E. E. | Antigen-Independent Binding of T-Cells by Dendritic Cells and Alveolar Macrophages in the Rat | 1989 |
| Kraich, Michael,Klein, Markus,Patino, Edwin,Harrer, Henning,Nickel, Joachim,Sebald, Walter,Mueller, Thomas D. | A modular interface of IL-4 allows for scalable affinity without affecting specificity for the IL-4 receptor | 2006 |
| Kramer, A.,Assadian, O.,Ryll, S.,Selleng, K.,Below, H. | Immediate infection control measures and preventive monitoring after excessive water damage in an aseptic working area of a blood donation service centre | 2015 |
| Kramer, J. M.,Waldrop, T. G.,Frizzell, L. A.,Zachary, J. F.,O'Brien, W. D. | Cardiopulmonary function in rats with lung hemorrhage induced by pulsed ultrasound exposure | 2001 |
| Kramer, M.,Fruchter, O.,Maimon, M. N.,Refaely, Y. | Single session bilateral endoscopic lung volume reduction therapy in advanced upper lobe and homogeneous emphysema using a tissue sealant | 2011 |
| Kramer, M.,Refaely, Y.,Maimon, M. N.,Fruchter, O. | Long term follow-up following bilateral endoscopic lung volume reduction therapy with AeriSeal® system foam sealant for advanced emphysema | 2012 |
| Kramer, M.,Refaely, Y.,Maimon, N.,Fruchter, O. | Safety and efficacy of emphysematous lung sealant (ELS) therapy for advanced emphysema out to 2 years | 2013 |
| Krcmova, I.,Bousova, K. | Occupational asthma caused by diisocyanates: Mechanisms of etiopathogenesis | 2003 |
| Krčmová, I.,Boušová, K. | Occupational asthma caused by diisocyanates: Mechanisms of etiopathogenesis | 2003 |
| Kreger, M.,Sargent, K.,Arons, A.,Standish, M.,Brindis, C. D. | Creating an Environmental Justice Framework for Policy Change in Childhood Asthma: A Grassroots to Treetops Approach | 2011 |
| Kreikemeyer, B.,Klenk, M.,Podbielski, A. | The intracellular status of Streptococcus pyogenes: role of extracellular matrix-binding proteins and their regulation | 2004 |
| Kreutz, W. | The Vascularization of Epiglottic Cartilage a Histological Investigation | 1980 |
| Kreutzberg, G. W. | Microglia, the first line of defence in brain pathologies | 1995 |
| Krieger, J.,Higgins, D. L. | Housing and health: time again for public health action | 2002 |
| Krieger, J.,Jacobs, D. E.,Ashley, P. J.,Baeder, A.,Chew, G. L.,Dearborn, D.,Hynes, H. P.,Miller, J. D.,Morley, R.,Rabito, F.,Zeldin, D. C. | Housing interventions and control of asthma-related indoor biologic agents: a review of the evidence | 2010 |
| Krieger, P.,de Blay, F.,Pauli, G.,Kopferschmitt, M. C. | [Asthma and household chemical pollutants (with the exception of tobacco)] | 1998 |
| Krieger, P.,De, B. L. A. Y. F.,Pauli, G.,Kopferschmitt, M. C. | Asthma and domestic chemical pollutants (excluding tobacco) | 1998 |
| Krishnaswamy, G.,Kelley, J.,Yerra, L.,Smith, J. K.,Chi, D. S. | Human endothelium as a source of multifunctional cytokines: Molecular regulation and possible role in human disease | 1999 |
| Krizman, David,Adey, Nils,Parry, Robert | Application of tissue mesodissection to molecular cancer diagnostics | 2015 |
| Krol, A.,Szarko, M.,Vala, A. | Pressure monitoring-should this become widespread? | 2013 |
| Krone, C. A. | Diisocyanates and nonoccupational disease: a review | 2005 |
| Krone, C. A.,Ely, J. T.,Klingner, T.,Rando, R. J. | Isocyanates in flexible polyurethane foams | 2003 |
| Krone, C. A.,Ely, J. T. A.,Klingner, T.,Rando, R. J. | Isocyanates in flexible polyurethane foams | 2003 |
| Krone, C. A.,Klingner, T. D. | Isocyanates, polyurethane and childhood asthma | 2005 |
| Krone, Cheryl A.,Klingner, Tom D.,Rando, Roy J.,Ely, John T. A. | Letter to the editor - Author's response | 2005 |
| Krop, E. J. M.,Jacobs, J. H.,Sander, I.,Raulf-Heimsoth, M.,Heederik, D. J. J. | Allergens and beta-Glucans in Dutch Homes and Schools: Characterizing Airborne Levels | 2014 |
| Krop, J.,Swierczek, J. | PATIENT WITH SEVERE INTRACTABLE ASTHMA URTICARIA AND THE IRRITABLE BOWEL SYNDROME RESPONSE TO SAUNA THERAPY | 1987 |
| Krug, N.,Thurau, A. M.,Lackie, P.,Baier, J.,Schultze-Werninghau, G.,Reiger, C. H. L.,Schauer, U. | A flow cytometric method for the detection of intracellular basic proteins in unseparated peripheral blood and bone marrow eosinophils | 1996 |
| Kruijsen, D.,Schijf, M. A.,Lukens, M. V.,van Uden, N. O.,Kimpen, J. L.,Coenjaerts, F. E.,van Bleek, G. M. | Local innate and adaptive immune responses regulate inflammatory cell influx into the lungs after vaccination with formalin inactivated RSV | 2011 |
| Kryanovsky, S. G. | Die Atmungsorgane der Fischlarven (Teleostomi) | 1934 |
| Kryzhanovskaya, I. | A fortuitous find in the work-up of a chronic cough | 2015 |
| Ksiazek, A.,Sokolowska, G.,Marczewski, K.,Solski, J. | Leukopenia with Different Regenerated Hemo Dialysis Membranes | 1984 |
| Kudo, H.,Iguchi, S.,Yamada, T.,Kawase, T.,Saito, H.,Otsuka, K.,Mitsubayashi, K. | A flexible transcutaneous oxygen sensor using polymer membranes | 2006 |
| Kuhn, Charles,Mason, Robert J. | Immunolocalization of SPARC, tenascin, and thrombospondin in pulmonary fibrosis | 1995 |
| Kuijpers, T. W.,Hoogerwerf, M.,Kuijpers, K. C.,Schwartz, B. R.,Harlan, J. M. | Cross-Linking of Sialophorin (Cd43) Induces Neutrophil Aggregation in a Cd18-Dependent and a Cd18-Independent Way | 1992 |
| Kukielka, E.,Cederbaum, A. I. | Oxidation of Ethylene Glycol to Formaldehyde by Rat Liver Microsomes Role of Cytochrome P-450 and Reactive Oxygen Species | 1991 |
| Kulkarni, H. S.,Fajt, M. L.,Uvalle, C. E.,Hoffman, E. A.,Cook-Granroth, J.,Wenzel, S. E. | Airway wall thickness on computerized tomography correlates with increased mast cell markers in severe asthma | 2012 |
| Kumar, A.,Sriwastwa, V. M.,Lata, S.,Tiwari, R. K. | A novel study on carpet-dye-induced dermatitis and other changes in Rattus norvegicus | 2012 |
| Kumar, D.,Patel, G.,Vijayakrishnan, L.,Dastidar, S. G.,Ray, A. | Design and Synthesis of 3,5-Disubstituted-1,2,4-Oxadiazoles as Potent Inhibitors of Phosphodiesterase4B2 | 2012 |
| Kumar, K. D. | Human Health Monitoring Mobile Phone Application by Using the Wireless Nanosensor Based Embedded System | 2013 |
| Kumar, S.,Gupta, R. N. | Adverse reactions of cosmetic products: Regulatory challenges in India | 2014 |
| Kumar, S. S.,Marella, S. S.,Vipin, S.,Sharmistha, M. | Evaluation of analgesic and anti-inflammatory activity of symplocos racemosa | 2013 |
| Kumar, V. H.,Kishkurno, S.,Nielson, L.,Wang, H.,Ryan, R. M. | Alveolization and cytokine responses are altered in adult mice exposed to neonatal hyperoxia | 2010 |
| Kundu, S.,De, A.,Mitra, S. | Formaldehyde: Fact sheet reflecting uses, exposure hazards, health effects and toxicological profile- A broad overview for medical professionals and embalmers | 2015 |
| Kunel'skaya, N. L.,Skryabina, L. Yu | Hearing disorders in young subjects | 2014 |
| Kunkel, M.,Wahlmann, U.,Wagner, W. | Acoustic monitoring of the artificial airway--experimental results | 1997 |
| Kuo, C. H.,Miyazaki, D.,Yakura, K.,Araki-Sasaki, K.,Inoue, Y. | Role of Periostin and Interleukin-4 in Recurrence of Pterygia | 2010 |
| Kupper, S.,Mees, S. T.,Gassmann, P.,Brodde, M. F.,Kehrel, B.,Haier, J. | Hydroxyethyl starch normalizes platelet and leukocyte adhesion within pulmonary microcirculation during LPS-induced endotoxemia | 2007 |
| Kurihara, M.,Kataoka, H. | Total pleural covering technique can prevent recurrent pneumothorax withoutadhesion of the lung in lymphangioleiomyomatosis patients requiring lung transplant in the future | 2012 |
| Kurihara, M.,Kataoka, H.,Otuji, M.,Ebana, H. | Preventing lam patients from recurrent pneumothorax - An innovative surgical method without adhesion: Total pleural covering technique | 2011 |
| Kurosawa, M.,Tsukagoshi, H.,Igarashi, Y.,Miyachi, Y. | CV6209 inhibits airway wall thickening and airway hyperresponsiveness to histamine induced by intravenous administration of platelet-activating factor in guinea pigs | 1996 |
| Kurosawa, M.,Yodonawa, S.,Inamura, H.,Tsukagoshi, H. | Inhibition by thromboxane antagonists of airway hyperresponsiveness to histamine induced by 13,14-dihydro-15-keto-PGF2 alpha in guinea-pigs | 1994 |
| Kurosawa, M.,Yodonawa, S.,Tsukagoshi, H. | Inhibition of bronchial hyperresponsiveness to histamine induced by intravenous administration of leukotriene C4 by novel thromboxane A2 receptor antagonists ONO-NT-126 and ONO-8809 in guinea-pigs | 1993 |
| Kurosawa, M.,Yodonawa, S.,Tsukagoshi, H.,Miyachi, Y. | Inhibition by a novel peptide leukotriene receptor antagonist ONO-1078 of airway wall thickening and airway hyperresponsiveness to histamine induced by leukotriene C4 or leukotriene D4 in guinea-pigs | 1994 |
| Kushiya, M.,Saito, K.,Kikuchi, I.,Kobayashi, T.,Hagiwara, K.,Kanazawa, M.,Nagata, M. | Differential effects of salbutamol and montelukast on eosinophil adhesion and superoxide anion generation | 2006 |
| Kuther, K.,Audige, L.,Kube, P.,Welle, M. | Bovine mast cells: distribution, density, heterogeneity, and influence of fixation techniques | 1998 |
| Kuwasaki, T.,Chihara, J.,Kayaba, H.,Kamata, Y.,Oyamada, H.,Saito, N.,Shioya, T.,Sasaki, M.,Kagaya, M.,Tsuda, A. | Whole-blood flow-cytometric analysis of eosinophil EG2 expression as a marker of the pathological conditions of asthma | 1998 |
| Kwan, A. M.,Fung, A. G.,Jansen, P. A.,Schivo, M.,Kenyon, N. J.,Delplanque, J. P.,Davis, C. E. | Personal Lung Function Monitoring Devices for Asthma Patients | 2015 |
| Kwong, F.,Kraske, G.,Nelson, A. M.,Klaustermeyer, W. B. | Acute symptoms secondary to formaldehyde exposure in a pathology resident | 1983 |
| La Grutta, S.,Indinnimeo, L.,di Coste, A.,Ferrante, G.,Landi, M.,Pelosi, U.,Rusconi, F. | Environmental risk factors and lung diseases in children: From guidelines to health effects | 2013 |
| Laberge, S.,Ernst, P.,Ghaffar, O.,Cruikshank, W. W.,Kornfeld, H.,Center, D. M.,Hamid, Q. | Increased expression of interleukin-16 in bronchial mucosa of subjects with atopic asthma | 1997 |
| Laberge, S.,Ghaffar, O.,Boguniewicz, M.,Center, D. M.,Leung, D. Y.,Hamid, Q. | Association of increased CD4(+) T-cell infiltration with increased IL-16 gene expression in atopic dermatitis | 1998 |
| Lachmann, B.,Berggren, P.,Curstedt, T.,Grossmann, G.,Robertson, B. | Combined effects of surfactant substitution and prolongation of inspiration phase in artificially ventilated premature newborn rabbits | 1982 |
| Lachowicz-Scroggins, M. E.,Gordon, E. D.,Urbanek, C.,MacLeod, H. J.,Yuan, S.,Woodruff, P. G.,Ramachandran, S.,Thorner, J. W.,Seibold, M. A.,Fahy, J. V. | Characterization of airway ORMDL3 expression in human asthma and in relation to the asthma gwas variant RS7216389 | 2014 |
| Lacroix, G.,Tissot, S.,Rogerieux, F.,Beaulieu, R.,Cornu, L.,Gillet, C.,Robidel, F.,Lefevre, J. P.,Bois, F. Y. | Decrease in ovalbumin-induced pulmonary allergic response by benzaldehyde but not acetaldehyde exposure in a guinea pig model | 2002 |
| Laffont, H.,Noceto, J. B. | [A case of asthma due to sensitization to formaldehyde] | 1961 |
| Lafon, D.,Garnier, R. | [Toxicity of thermal decomposition products of plastics] | 2008 |
| Lagrelius, Maria,Wahlgren, Carl-Fredrik,Matura, Mihaly,Kull, Inger,Liden, Carola | High prevalence of contact allergy in adolescence: results from the population-based BAMSE birth cohort | 2016 |
| Lagrelius, M.,Wahlgren, C. F.,Matura, M.,Kull, I.,Liden, C. | High prevalence of contact allergy in adolescence: results from the population-based BAMSE birth cohort | 2015 |
| Lagrelius, M.,Wahlgren, C. F.,Matura, M.,Kull, I.,Lidén, C. | High prevalence of contact allergy in adolescence: Results from the population-based BAMSE birth cohort | 2016 |
| Lai, P. S.,Austin, J. M.,Brown, R. D.,Macias, A. A.,Pinilla, M. A.,Englert, J. A.,Hofmann, O.,Hide, W.,Christiani, D. C.,Cernadas, M.,Baron, R. M. | Airway reactivity and persistent inflammation is associated with antigen presenting cell population shifts in a murine model of chronic inhalational endotoxin exposure | 2011 |
| Lai, P. S.,Christiani, D. C. | Long-term respiratory health effects in textile workers | 2013 |
| Lai, P. S.,Hang, J. Q.,Zhang, F. Y.,Lin, X.,Zheng, B. Y.,Dai, H. L.,Su, L.,Cai, T.,Christiani, D. C. | Gender differences in the effect of occupational endotoxin exposure on impaired lung function and death: the Shanghai Textile Worker Study | 2013 |
| Lai, Samuel K.,Wang, Ying-Ying,Hanes, Justin | Mucus-penetrating nanoparticles for drug and gene delivery to mucosal tissues | 2009 |
| Lai, S. K.,Wang, Y. Y.,Hanes, J. | Mucus-penetrating nanoparticles for drug and gene delivery to mucosal tissues | 2009 |
| Lalchev, Z.,Valtcheva, R.,Mitev, V.,Stephanova, E. | Tensiometric study of surface activity and halothane impact on biosurfactant production of lung cells | 2004 |
| Lalrinzuali, K.,Vabeiryureilai, M.,Jagetia, G. C. | Investigation of the Anti-Inflammatory and Analgesic Activities of Ethanol Extract of Stem Bark of Sonapatha Oroxylum indicum in Vivo | 2016 |
| Lam, S.,Chan-Yeung, M. | Ethylenediamine-induced asthma | 1980 |
| Lam, Tram K.,Shao, Stephanie,Zhao, Yingdong,Marincola, Francesco,Pesatori, Angela,Bertazzi, Pier Alberto,Caporaso, Neil E.,Wang, Ena,Landi, Maria Teresa | Influence of Quercetin-Rich Food Intake on microRNA Expression in Lung Cancer Tissues | 2012 |
| Lamas, A. M.,Mulroney, C. M.,Schleimer, R. P. | Studies on the Adhesive Interaction between Purified Human Eosinophils and Cultured Vascular Endothelial Cells | 1988 |
| Lambert, A. L.,Dong, W. M.,Selgrade, M. J. K.,Gilmour, M. I. | Enhanced allergic sensitization by residual oil fly ash particles is mediated by soluble metal constituents | 2000 |
| Lambropoulou, M.,Limberis, V.,Koutlaki, N.,Simopoulou, M.,Ntanovasilis, D.,Vandoros, G. P.,Tatsidou, P.,Kekou, I.,Koutsikogianni, I.,Papadopoulos, N. | Differential expression of tenascin-C in the developing human lung: an immunohistochemical study | 2009 |
| Lamkin, E.,Harford, T. J.,Brown, P. M.,Piedimonte, G.,Lauer, M. E. | Pediatric bronchial epithelial cells synthesize leukocyte-adhesive hyaluronan matrices in response to infection with respiratory syncytial virus | 2015 |
| Lammerding-Koeppel, M.,Greiner-Schroeder, A.,Drews, U. | Muscarinic receptors in the prenatal mouse embryo. Comparison of M35-immunohistochemistry with (3H)quinuclidinyl benzylate autoradiography | 1995 |
| Lammerdingkoppel, M.,Greinerschroder, A.,Drews, U. | Muscarinic Receptors in the Prenatal Mouse Embryo - Comparison of M35-Immunohistochemistry with [H-3] Quinuclidinyl Benzylate Autoradiography | 1995 |
| Lammy, S.,Velu, P. P. | Massive atrial myxoma: surgical treatment for an incidentaloma causing dyspnoea | 2013 |
| Landrigan, P. J. | Formaldehyde | 1992 |
| Lang, Ivan M.,Medda, Bidyut K.,Haworth, Steven T.,Shaker, Reza | Mechanism of the Effects of Esophageal Acidification On the Production and Secretion of Airway Mucus | 2009 |
| Lange, J. H. | Has the indoor and built environment started changing modern health? | 2002 |
| Larese, F.,Fiorito, A.,Casasola, F.,Molinari, S.,Peresson, M.,Barbina, P.,Negro, C. | Sensitization to green coffee beans and work-related allergic symptoms in coffee workers | 1998 |
| Larsen, S. T.,Dallot, C.,Larsen, S. W.,Rose, F.,Poulsen, S. S.,Norgaard, A. W.,Hansen, J. S.,Sorli, J. B.,Nielsen, G. D.,Foged, C. | Mechanism of action of lung damage caused by a nanofilm spray product | 2014 |
| Larsen, S. T.,Sørli, J. B.,Nørgaard, A.,Hansen, J. S. | Toxicity of waterproofing spray products. Elucidation of the mechanism of action | 2014 |
| Larsen, S. T.,Wolkoff, P.,Hammer, M.,Kofoed-Sorensen, V.,Clausen, P. A.,Nielsen, G. D. | Acute airway effects of airborne formaldehyde in sensitized and non-sensitized mice housed in a dry or humid environment | 2013 |
| Larson, S. D.,Plopper, C. G.,Baker, G.,Tarkington, B. K.,Decile, K. C.,Pinkerton, K.,Mansoor, J. K.,Hyde, D. M.,Schelegle, E. S. | Proximal airway mucous cells of ovalbumin-sensitized and -challenged Brown Norway rats accumulate the neuropeptide calcitonin gene-related peptide | 2004 |
| Larsson, M.,Weiss, B.,Janson, S.,Sundell, J.,Bornehag, C. G. | Associations between indoor environmental factors and parental-reported autistic spectrum disorders in children 6-8 years of age | 2009 |
| Lastbom, L.,Colmsjo, A.,Johansson, R.,Karlsson, D.,Melin, J.,Nordqvist, Y.,Skarping, G. | Effects of thermal degradation products from polyurethane foams based on toluene diisocyanate and diphenylmethane diisocyanate on isolated, perfused lung of guinea pig | 2003 |
| Lau, S.,Wahn, J.,Schulz, G.,Sommerfeld, C.,Wahn, U. | Placebo-controlled study of the mite allergen-reducing effect of tannic acid plus benzyl benzoate on carpets in homes of children with house dust mite sensitization and asthma | 2002 |
| Laucho-Contreras, M. E.,Taylor, K. L.,Mahadeva, R.,Boukedes, S. S.,Owen, C. A. | Automated Measurement of Pulmonary Emphysema and Small Airway Remodeling in Cigarette Smoke-exposed Mice | 2015 |
| Lauer, Mark E.,Erzurum, Serpil C.,Mukhopadhyay, Durba,Vasanji, Amit,Drazba, Judith,Wang, Aimin,Fulop, Csaba,Hascall, Vincent C. | Differentiated murine airway epithelial cells synthesize a leukocyte-adhesive hyaluronan matrix in response to endoplasmic reticulum stress | 2008 |
| Lauer, M. E.,Erzurum, S. C.,Mukhopadhyay, D.,Vasanji, A.,Drazba, J.,Wang, A. M.,Fulop, C.,Hascall, V. C. | Differentiated murine airway epithelial cells synthesize a leukocyte-adhesive hyaluronan matrix in response to endoplasmic reticulum stress | 2008 |
| Lauer, M. E.,Mukhopadhyay, D.,Fulop, C.,de la Motte, C. A.,Majors, A. K.,Hascall, V. C. | Primary Murine Airway Smooth Muscle Cells Exposed to Poly(I,C) or Tunicamycin Synthesize a Leukocyte-adhesive Hyaluronan Matrix | 2009 |
| Lauzon, Anne-Marie,Bates, Jason H. T.,Donovan, Graham,Tawhai, Merryn,Sneyd, James,Sanderson, Michael J. | A multi-scale approach to airway hyperresponsiveness: from molecule to organ | 2012 |
| Lavigne, S.,Bosse, M.,Boulet, L. P.,Laviolette, M. | Identification and analysis of eosinophils by flow cytometry using the depolarized side scatter-saponin method | 1997 |
| Lawrence, A.,Fatima, N. | Urban air pollution & its assessment in Lucknow City - The second largest city of North India | 2014 |
| Lawuyi, R.,Fingas, M. | Environmental impact of methyl tert-butyl ether (MTBE) | 1997 |
| Lazar, G.,Biliczki, F.,Kovacs, K. | The Phagocytic Function of the Res in Rats Treated with Polybrene Liquoid and Compound 48-80 P Methoxyphenethylamine Condensation Product with Formaldehyde | 1968 |
| Lazarov, A. | European Standard Series patch test results from a contact dermatitis clinic in Israel during the 7-year period from 1998 to 2004 | 2006 |
| Lazenby, J. J.,Cooley, M. A. | Inhibition of dendritic cell antigen presenting function by the pseudomonas aeruginosa quorum sensing molecule OdDHL | 2005 |
| Lazenby, V.,Hinwood, A.,Callan, A.,Franklin, P. | Formaldehyde personal exposure measurements and time weighted exposure estimates in children | 2012 |
| Le Roux, P.,Quinque, K.,Bonnel, A. S.,Hastier, N.,Le Luyer, B. | Climatotherapy | 2005 |
| Leaderer, B. P.,Belanger, K.,Triche, E.,Holford, T.,Gold, D. R.,Kim, Y.,Jankun, T.,Ren, P.,McSharry Je, J. E.,Platts-Mills, T. A.,Chapman, M. D.,Bracken, M. B. | Dust mite, cockroach, cat, and dog allergen concentrations in homes of asthmatic children in the northeastern United States: impact of socioeconomic factors and population density | 2002 |
| Leal, Lkam,Pierdona, T. M.,Goes, J. G. S.,Fonseca, K. S.,Canuto, K. M.,Silveira, E. R.,Bezerra, A. M. E.,Viana, G. S. B. | A comparative chemical and pharmacological study of standardized extracts and vanillic acid from wild and cultivated Amburana cearensis AC Smith | 2011 |
| Leal, L. K.,Pierdona, T. M.,Goes, J. G.,Fonseca, K. S.,Canuto, K. M.,Silveira, E. R.,Bezerra, A. M.,Viana, G. S. | A comparative chemical and pharmacological study of standardized extracts and vanillic acid from wild and cultivated Amburana cearensis A.C. Smith | 2010 |
| Leal, L. K. A. M.,Pierdona, T. M.,Goes, J. G. S.,Fonseca, K. S.,Canuto, K. M.,Silveira, E. R.,Bezerra, A. M. E.,Viana, G. S. B. | A comparative chemical and pharmacological study of standardized extracts and vanillic acid from wild and cultivated Amburana cearensis AC Smith | 2011 |
| Leal, M. L. D.,Aires, A. R.,Fillapi, A.,Trost, M. E. | Clinical and Pathological Observations Associated with Snake Envenomation in Two Sheep | 2013 |
| Leavey, A.,Perez, H. R.,Burstyn, I. | Endotoxin and particle concentrations in subsidized households with asthmatic children in Philadelphia: A pilot study | 2012 |
| Leb, Victoria M.,Jahn-Schmid, Beatrice,Kueng, Hans J.,Schmetterer, Klaus G.,Haiderer, Daniela,Neunkirchner, Alina,Fischer, Gottfried F.,Hartl, Arnulf,Thalhamer, Josef,Steinberger, Peter,Bohle, Barbara,Seed, Brian,Pickl, Winfried F. | Modulation of allergen-specific T-lymphocyte function by virus-like particles decorated with HLA class II molecules | 2009 |
| Lebenthal, Emanuel,Rolston, David D. K.,Holsclaw, Douglas S., Jr. | Enzyme therapy for pancreatic insufficiency: Present status and future needs | 1994 |
| Leblanc, A.,Coentrao, L.,Martins, P.,Carvalho, B.,Botelho, C.,Castro, E. | Haemodialysis related anaphylaxis - finding the culprit | 2010 |
| Leblanc, A.,Coentrão, L.,Martins, P.,Carvalho, B.,Botelho, C.,Castro, E. | Haemodialysis related anaphylaxis - Finding the culprit | 2010 |
| Lecureur, V.,Arzel, M.,Ameziane, S.,Houlbert, N.,Le Vee, M.,Jouneau, S.,Fardel, O. | MAPK- and PKC/CREB-dependent induction of interleukin-11 by the environmental contaminant formaldehyde in human bronchial epithelial cells | 2012 |
| Lecureur, V.,Arzel, M.,Ameziane, S.,Le Vee, M.,Jouneau, S.,Fardel, O. | Induction of IL-11 by formaldehyde in human bronchial epithelial cells via MAPK and CREB | 2011 |
| Leduc, C.,Qi, S.,Bouzoubaa, S.,Ott, M.,Dazy, A.,de Blay, F. | Long-term effect of acaricides pre-treated home furnishings on mite allergen exposure | 2015 |
| Leduc, Dominique,Beaufort, Nathalie,de Bentzmann, Sophie,Rousselle, Jean-Claude,Namane, Abdelkader,Chignard, Michel,Pidard, Dominique | The Pseudomonas aeruginosa LasB metalloproteinase regulates the human urokinase-type plasminogen activator receptor through domain-specific endoproteolysis | 2007 |
| Lee, A. M.,Kirby, M.,Ohtani, K.,Candido, T.,Shalansky, R.,MacAulay, C.,English, J.,Finley, R.,Lam, S.,Coxson, H. O.,Lane, P. | Validation of airway wall measurements by optical coherence tomography in porcine airways | 2014 |
| Lee, A. M. D.,Kirby, M.,Ohtani, K.,Candido, T.,Shalansky, R.,MacAulay, C.,English, J.,Finley, R.,Lam, S.,Coxson, H. O.,Lane, P. | Validation of Airway Wall Measurements by Optical Coherence Tomography in Porcine Airways | 2014 |
| Lee, A. M. D.,Shalansky, R.,Candido, T.,Ohtani, K.,English, J. C.,Finley, R. J.,Lam, S.,Coxson, H. O. | Using optical coherence tomography (OCT) to measure airway wall structure | 2012 |
| Lee, C. M.,Hong, S. J.,Kim, Y. S.,Park, G. Y.,Goung, S. J. N.,Kim, K. H. | Distribution features of biological hazardous pollutants in residential environments in Korea | 2014 |
| Lee, Eun-Young,Chun, Myung-Kwan,Chang, Jae-Sang,Choi, Hoo-Kyun | Development of matrix based transdermal delivery system for ketotifen | 2014 |
| Lee, E. Y.,Chun, M. K.,Chang, J. S.,Choi, H. K. | Development of matrix based transdermal delivery system for ketotifen | 2014 |
| Lee, G. B.,Brandt, E. B.,Xiao, C.,Gibson, A. M.,Le Cras, T. D.,Brown, L. A. S.,Fitzpatrick, A. M.,Hershey, G. K. K. | Diesel Exhaust Particles Induce Cysteine Oxidation and S-Glutathionylation in House Dust Mite Induced Murine Asthma | 2013 |
| Lee, G. H.,Nam, Y.,Kim, E. M.,Lim, S. J.,Yang, S. J.,Park, K.,Choi, Y.,Gong, Y. D. | Novel N-(2,2 '-disubstituted-2H-cromenyl)-N ',N ''-disubstituted Guanidine Derivatives as an S-Nitrosoglutathion Reductase Inhibitor | 2015 |
| Lee, H.,Kim, G. S. | Geographical and Sociodemographic Risk Factors for Allergic Diseases in Korean Children | 2011 |
| Lee, H. K.,Alarie, Y.,Karol, M. H. | Induction of formaldehyde sensitivity in guinea pigs | 1984 |
| Lee, H. S.,Phoon, W. H. | Occupational Asthma in Singapore | 1989 |
| Lee, H. S.,Phoon, W. H. | Diurnal variation in peak expiratory flow rate among workers exposed to toluene diisocyanate in the polyurethane foam manufacturing industry | 1992 |
| Lee, H. S.,Wang, Y. T.,Cheong, T. H.,Tan, K. T.,Chee, B. E.,Narendran, K. | Occupational Asthma due to Maleic Anhydride | 1991 |
| Lee, J.,Cho, Y.,Hwang, Y.,Kim, H. | Comparison of serum osteopontin levels during acute exacerbation and in stable patients with COPD | 2011 |
| Lee, J.,Singh, G.,Mohindra, V.,Chen, W.,Friedenberg, A. | A case of plastic bronchitis in an adult following cardiopulmonary bypass surgery | 2014 |
| Lee, J. H.,Kim, J.,Lee, S. W.,Suh, J.,Yu, J. S.,Park, E.,Lee, J.,Kim, H.,Lee, K. S.,Chang, E. Y.,Cho, J. B.,Kim, K. B.,Han, Y.,Ahn, K.,Lee, S. I. | The clinical effects of hospitalization in a low pollutant room on atopic dermatitis | 2011 |
| Lee, J. H.,Lee, H. S.,Park, M. R.,Lee, S. W.,Kim, E. H.,Cho, J. B.,Kim, J.,Han, Y.,Jung, K.,Cheong, H. K.,Lee, S. I.,Ahn, K. | Relationship between indoor air pollutant levels and residential environment in children with atopic dermatitis | 2014 |
| Lee, J. S.,Kwak, H. S.,Choi, B. S.,Park, S. Y. | A case of occupational asthma in a plastic injection process worker | 2014 |
| Lee, J. Y.,Ryu, S. H.,Lee, G.,Bae, G. N. | Indoor-to-outdoor particle concentration ratio model for human exposure analysis | 2016 |
| Lee, Kenneth | OSHA Compliance Issues Benzene and Crystalline Silica Exposures in a Grey Iron Foundry | 2009 |
| Lee, K. | Evidence of policy impact on population exposure improvement | 2011 |
| Lee, Kang-Myeong,Park, Jun-Ho,Koh, Sang-Baek,Kim, Jae-Hong,Choi, Eung-Ho,Chang, Sei-Jin | Effect of Lacquering on Indoor Air Carbonyl Compound Quality and Human Malondialdehyde Levels | 2011 |
| Lee, K. M.,Park, J. H.,Koh, S. B.,Kim, J. H.,Choi, E. H.,Chang, S. J. | Effect of lacquering on indoor air carbonyl compound quality and human malondialdehyde levels | 2011 |
| Lee, K. Y.,Kwak, K. H.,Ryu, Y. H.,Lee, S. H.,Baik, J. J. | Impacts of biogenic isoprene emission on ozone air quality in the Seoul metropolitan area | 2014 |
| Lee, M. H.,Kim, Y. A.,Na, T. Y.,Kim, S. H.,Shin, Y. K.,Lee, B. H.,Shin, H. S.,Lee, M. O. | Identification of formaldehyde-responsive genes by suppression subtractive hybridization | 2008 |
| Lee, Myeong Soo,Jeong, Seong Min,Kim, Yong-Kyu,Park, Ki-Won,Lee, Myung Suk,Ryu, Hoon,Moon, Sun-Rock | Qi-training enhances respiratory burst function and adhesive capacity of neutrophils in young adults: A preliminary study | 2003 |
| Lee, M. S.,Jeong, S. M.,Kim, Y. K.,Park, K. W.,Lee, M. S.,Ryu, H.,Moon, S. R. | Qi-training enhances respiratory burst function and adhesive capacity of neutrophils in young adults: A preliminary study | 2003 |
| Lee, S.,Eren, M.,Vaughan, D. E.,Cho, S. H. | A specific PAI-1 inhibitor, PAI-039, reduced airway inflammation and collagen deposition in an ovachallenged murine model of chronic asthma | 2011 |
| Lee, S.,Kim, E.,Kim, J.,Han, Y.,Jeong, H.,Ahn, K. | Relationship between the indoor residential environment and atopic dermatitis symptoms | 2013 |
| Lee, S. Y.,Chang, Y. S.,Cho, S. H. | Allergic diseases and air pollution | 2013 |
| Lee, T.,Harper, M.,Slaven, J. E.,Lee, K.,Rando, R. J.,Maples, E. H. | Wood Dust Sampling: Field Evaluation of Personal Samplers When Large Particles Are Present | 2011 |
| Lee, Wei-Jiunn,Chen, Wen-Kang,Wang, Chau-Jong,Lin, Wea-Lung,Tseng, Tsui-Hwa | Apigenin inhibits HGF-promoted invasive growth and metastasis involving blocking PI3K/Akt pathway and beta 4 integrin function in MDA-MB-231 breast cancer cells | 2008 |
| Leef, M.,Elkins, K. L.,Barbic, J.,Shahin, R. D. | Protective immunity to Bordetella pertussis requires both B cells and CD4(+) T cells for key functions other than specific antibody production | 2000 |
| Leem, J. H. | Cement dust and environmental diseases | 2012 |
| Leggat, P. A.,Smith, D. R.,Kedjarune, U. | Surgical applications of cyanoacrylate adhesives: A review of toxicity | 2007 |
| Lei, Cai-Xia,Zhang, Wei,Sun, Xiao-Wei,Du, Guo-Ping,Li, Wang,Liu, Yin-Kun | Effects of Galectin-3 on proliferation and adhesion of human endometrial cell line | 2007 |
| Lei, M.,Ghezzo, H.,Chen, M. F.,Eidelman, D. H. | Airway smooth muscle orientation in intraparenchymal airways | 1997 |
| Leikauf, G. D. | Mechanisms of aldehyde-induced bronchial reactivity: role of airway epithelium | 1992 |
| Leikauf, G. D. | Hazardous air pollutants and asthma | 2002 |
| Leite, F. C.,Ribeiro, J.,Costa, H. F.,Salgado, P. R. R.,Calheiros, A. S.,Carneiro, A. B.,de Almeida, R. N.,Dias, C. D.,Bozza, P. T.,Piuvezam, M. R. | Curine, an Alkaloid Isolated from Chondrodendron platyphyllum Inhibits Prostaglandin E-2 in Experimental Models of Inflammation and Pain | 2014 |
| Lemi†ere, C.,Cloutier, Y.,Perrault, G.,Drolet, D.,Cartier, A.,Malo, J. L. | Closed-circuit apparatus for specific inhalation challenges with an occupational agent, formaldehyde, in vapor form | 1996 |
| Lemiere, C.,Cloutier, Y.,Perrault, G.,Drolet, D.,Cartier, A.,Malo, J. L. | Closed-Circuit Apparatus for Specific Inhalation Challenges with an Occupational Agent, Formaldehyde, in Vapor Form | 1631 |
| Lemiere, C.,Cloutier, Y.,Perrault, G.,Drolet, D.,Cartier, A.,Malo, J. L. | Closed-circuit apparatus for specific inhalation challenges with an occupational agent, formaldehyde, in vapor form | 1996 |
| Leonenko, Zoya,Finot, Eric,Vassiliev, Vladislav,Amrein, Matthias | Effect of cholesterol on the physical properties of pulmonary surfactant films: Atomic force measurements study | 2006 |
| Lepere, A. J.,Finn, J.,Jacobs, I. | Efficacy of cardiopulmonary resuscitation performed in a dental chair | 2004 |
| Lesage, J.,Goyer, N.,Desjardins, F.,Vincent, J. Y.,Perrault, G. | Workers Exposure to Isocyanates | 1992 |
| Leslie, G. B.,Lunau, F. W. | Indoor air pollution. Problems and priorities | 1992 |
| Lester, M. A.,Levagood, F. B. | PULMONARY EMBOLISM | 1964 |
| Leung, R.,Lam, C. W.,Chan, A.,Lee, M.,Chan, I. H.,Pang, S. W.,Lai, C. K. | Indoor environment of residential homes in Hong Kong--relevance to asthma and allergic disease | 1998 |
| Leung, T. F.,Wong, Y. S.,Chan, I. H.,Yung, E.,Wong, C. K.,Lam, C. W.,Wong, G. W. | Indoor determinants of endotoxin and dust mite exposures in Hong Kong homes with asthmatic children | 2010 |
| Levering, P. R.,Dijkhuizen, L. | Regulation and Function of Transaldolase Isoenzymes Involved in Sugar and One-Carbon Metabolism in the Ribulose Monophosphate Cycle Methylotroph Arthrobacter P-1 | 1986 |
| Levi, A.,Shechter, Y.,Neufeld, E. J.,Schlessinger, J. | Mobility Clustering and Transport of Nerve Growth Factor in Embryonal Sensory Cells and in a Sympathetic Neuronal Cell Line | 1980 |
| Levy, D. A. | Hazards and adverse reactions associated with administration of allergenic extracts | 1980 |
| Levy, J. I.,Nishioka, Y.,Spengler, J. D. | The public health benefits of insulation retrofits in existing housing in the United States | 2003 |
| Lewis, A. J.,Nicholls, P. J. | Histamine Metabolism and Sensitivity in Scorbutic Guinea-Pigs | 1973 |
| Lewis, R. D.,Breyse, P. N.,Lees, P. S. J.,Diener-West, M.,Hamilton, R. G.,Eggleston, P. | Factors affecting the retention of dust mite allergen on carpet | 1998 |
| Lewis, R. D.,Breysse, P. N.,Lees, P. S.,Diener-West, M.,Hamilton, R. G.,Eggleston, P. | Factors affecting the retention of dust mite allergen on carpet | 1998 |
| Lewis, R. D.,Breysse, P. N.,Lees, P. S. J.,Diener-West, M.,Hamilton, R. G.,Eggleston, P. | Factors affecting the retention of dust mite allergen on carpet | 1998 |
| Lewis, T. C.,Parker, E.,Robins, T.,Batterman, S.,Mukherjee, B.,Mentz, G. B.,Ren, X.,Godwin, C.,O'Toole, A. R.,Grant, S. | Suitability of homes of asthmatic children in detroit for installation of window unit air conditioners | 2011 |
| Li, C. S.,Hsu, C. W.,Chua, K. Y.,Hsieh, K. H.,Lin, R. H. | Environmental distribution of house dust mite allergen (Der p 5) | 1996 |
| Li, C. S.,Hsu, C. W.,Tai, M. L. | Indoor pollution and sick building syndrome symptoms among workers in day-care centers | 1997 |
| Li, Dechun,Li, Ruihong,Wu, Ping,Li, Xiaopeng,Zhang, Xinhui | Clinical and Diagnostic Significance of Enteroclysis Through Nasointestinal Decompression Intubation | 2014 |
| Li, Hongmei,Balsells-padros, Jaume,Humphrey, Guy,Chen, Cheng Yi,Cohen, Benjamin,Kumke, Daniel J.,Gosselin, Francis,Nadeau, Christian,Dolman, Sarah,O'Shea, Paul | Development of long term manufacturing process for MK-0633 (Setileuton) | 2010 |
| Li, J.,Lu, Y.,Huang, K.,Wang, C.,Lu, J.,Zhang, C.,Zhong, N. | Chinese response to allergy and asthma in Olympic athletes | 2008 |
| Li, K.,Kim, J.,Jeong, M.,Kim, D.,Seo, S. | Effects of repeated formaldehyde (FA) exposure on atopic dermatitis (AD)-like immune responses in NC/Nga mice | 2011 |
| Li, Li,Das, Anuk M.,Torphy, Theodore J.,Griswold, Don E. | What's in the pipeline? Prospects for monoclonal antibodies (mAbs) as therapies for lung diseases | 2002 |
| Li, Ning,Harkema, Jack R.,Lewandowski, Ryan P.,Wang, Meiying,Bramble, Lori A.,Gookin, Glenn R.,Ning, Zhi,Kleinman, Michael T.,Sioutas, Constantinos,Nel, Andre E. | Ambient ultrafine particles provide a strong adjuvant effect in the secondary immune response: implication for traffic-related asthma flares | 2010 |
| Li, Q.,Jackson, R.,Capellan, D.,Van Snick, J.,Renauld, J. C.,Levitt, R. | The dutch hypothesis revisited: Interleukin 9 over-expression promotes lung emphysema after chronic tobacco smoke exposure | 2012 |
| Li, W.,Saphores, J. D. | Assessing Impacts of Freeway Truck Traffic on Residential Property Values Southern California Case Study | 2012 |
| Li, Yang,Han, Wenyu,Lei, Liancheng,Li, Zhijie,Shi, Lei | MrkD adhesin of Klebsiella pneumoniae expression, purification and analysis of adhesive activity | 2009 |
| Li, Yin,Ye, Yaoqin | Experimental study on adhesion mechanism of airway inflammation in the pathogenesis of bronchial asthma | 1999 |
| Li, Z. B.,Lehar, M.,Nakagawa, H.,Hoh, J. F.,Flint, P. W. | Differential expression of myosin heavy chain isoforms between abductor and adductor muscles in the human larynx | 2004 |
| Liang, K. L.,Su, M. C.,Shiao, J. Y.,Wu, S. H.,Li, Y. H.,Jiang, R. S. | Role of Pollen Allergy in Taiwanese Patients With Allergic Rhinitis | 2010 |
| Liao, V. H.,Chou, W. C.,Chio, C. P.,Ju, Y. R.,Liao, C. M. | A probabilistic approach to quantitatively assess the inhalation risk for airborne endotoxin in cotton textile workers | 2009 |
| Lidstrom, Mary E.,Anthony, Christopher,Biville, Francis,Gasser, Francis,Goodwin, Pat,Hanson, Richard S.,Harms, Nellie | New unified nomenclature for genes involved in the oxidation of methanol in gram-negative bacteria | 1994 |
| Lieberman, J. S.,Kane, G. C. | Asthma mortality: the worldwide response | 1997 |
| Liira, H.,Kovesi, T. | Commentaries on 'Remediating buildings damaged by dampness and mould for preventing or reducing respiratory tract symptoms, infections and asthma' | 2013 |
| Liljelind, I.,Norberg, C.,Egelrud, L.,Westberg, H.,Eriksson, K.,Nylander-French, L. A. | Dermal and inhalation exposure to methylene bisphenyl isocyanate (MDI) in iron foundry workers | 2009 |
| Lillienberg, L.,Burdorf, A.,Mathiasson, L.,Thorneby, L. | Exposure to metalworking fluid aerosols and determinants of exposure | 2008 |
| Lim, S. K.,Kim, J. C.,Moon, C. J.,Kim, G. Y.,Han, H. J.,Park, S. H. | Formaldehyde induces apoptosis through decreased Prx 2 via p38 MAPK in lung epithelial cells | 2010 |
| Limjunyawong, N.,Mock, J.,Mitzner, W. | Instillation and Fixation Methods Useful in Mouse Lung Cancer Research | 2015 |
| Limper, A. H.,Roman, J. | Fibronectin - a Versatile Matrix Protein with Roles in Thoracic Development, Repair and Infection | 1992 |
| Lin, C.,Frieri, M. | Black rubber and p-phenylenediamine contact dermatitis | 2014 |
| Lin, P. C.,Ong, J.,Lee, C. L.,Chen, T. Y.,Lee, Y.,Lai, H. Y. | Comparisons of the GlideScope and Macintosh Laryngoscope in Tracheal Intubation by Medical Students on Fresh Human Cadavers | 2009 |
| Lin, T. C.,Krishnaswamy, G.,Chi, D. S. | Incense smoke: Clinical, structural and molecular effects on airway disease | 2008 |
| Linden, D. R.,Peck, R. D.,Szurszewski, J. H.,Faubion, W. A. | Endogenous colonic hydrogen sulfide prevents inflammatory oxidation | 2012 |
| Lindenberg, L. B.,Wescott, J.,Cole, S.,Davis, J. | Pulmonary thromboembolism in advanced pulmonary fibrosis | 1977 |
| Linder, A.,Lindholm, C. E. | Vocal Fold Lateralization Using Carbon-Dioxide Laser and Fibrin Glue | 1992 |
| Lindstrom, I.,Suojalehto, H.,Henriks-Eckerman, M. L.,Suuronen, K. | Occupational asthma and rhinitis caused by cyanoacrylate-based eyelash extension glues | 2013 |
| Ling, Z. H.,Guo, H.,Zou, S. C. | Preliminary Evaluation of Airborne Pollutants at a Livestock Farm in the Pearl River Delta Region, China | 2010 |
| Linnainmaa, M.,Kiviranta, H.,Laitinen, J.,Laitinen, S. | Control of workers' exposure to airborne endotoxins and formaldehyde during the use of metalworking fluids | 2003 |
| Lino dos Santos Franco, A.,Damazo, A. S.,Beraldo de Souza, H. R.,Domingos, H. V.,Oliveira-Filho, R. M.,Oliani, S. M.,Costa, S. K.,Tavares de Lima, W. | Pulmonary neutrophil recruitment and bronchial reactivity in formaldehyde-exposed rats are modulated by mast cells and differentially by neuropeptides and nitric oxide | 2006 |
| Lino dos Santos Franco, A.,Domingos, H. V.,Damazo, A. S.,Breithaupt-Faloppa, A. C.,de Oliveira, A. P.,Costa, S. K.,Oliani, S. M.,Oliveira-Filho, R. M.,Vargaftig, B. B.,Tavares-de-Lima, W. | Reduced allergic lung inflammation in rats following formaldehyde exposure: long-term effects on multiple effector systems | 2008 |
| Lino-dos-Santos-Franco, A.,Amemiya, R. M.,de Oliveira, A. P. L.,Breithaupt-Faloppa, A. C.,Damazo, A. S.,Oliveira, R. M.,Tavares-de-Lima, W. | Differential effects of female sex hormones on cellular recruitment and tracheal reactivity after formaldehyde exposure | 2011 |
| Lino-dos-Santos-Franco, A.,Amemiya, R. M.,de Oliveira, A. P. L.,Damazo, A. S.,Breithaupt-Faloppa, A. C.,Vitoretti, L. B.,Acceturi, B. G.,Tavares-de-Lima, W. | The putative role of ovary removal and progesterone when considering the effect of formaldehyde exposure on lung inflammation induced by ovalbumin | 2013 |
| Lino-dos-Santos-Franco, A.,Amemiya, R. M.,Ligeiro de Oliveira, A. P.,Breithaupt-Faloppa, A. C.,Damazo, A. S.,Oliveira-Filho, R. M.,Tavares-de-Lima, W. | Differential effects of female sex hormones on cellular recruitment and tracheal reactivity after formaldehyde exposure | 2011 |
| Lino-dos-Santos-Franco, A.,Correa-Costa, M.,Durao, Accd,de Oliveira, A. P. L.,Breithaupt-Faloppa, A. C.,Bertoni, J. D.,Oliveira, R. M.,Camara, N. O. S.,Marcourakis, T.,Tavares-de-Lima, W. | Formaldehyde induces lung inflammation by an oxidant and antioxidant enzymes mediated mechanism in the lung tissue | 2011 |
| Lino-dos-Santos-Franco, A.,Domingos, H. V.,de Oliveira, A. P.,Breithaupt-Faloppa, A. C.,Peron, J. P.,Bolonheis, S.,Muscara, M. N.,Oliveira-Filho, R. M.,Vargaftig, B. B.,Tavares-de-Lima, W. | Differential effects of formaldehyde exposure on the cell influx and vascular permeability in a rat model of allergic lung inflammation | 2010 |
| Lino-dos-Santos-Franco, A.,Domingos, H. V.,de Oliveira, A. P. L.,Breithaupt-Faloppa, A. C.,Peron, J. P. S.,Bolonheis, S.,Muscara, M. N.,Oliveira, R. M.,Vargaftig, B. B.,Tavares-de-Lima, W. | Differential effects of formaldehyde exposure on the cell influx and vascular permeability in a rat model of allergic lung inflammation | 2010 |
| Lino-dos-Santos-Franco, Adriana,Domingos, Helori Vanni,Ligeiro de Oliveira, Ana Paula,Breithaupt-Faloppa, Ana Cristina,Schatzmann Peron, Jean Pierre,Bolonheis, Simone,Muscara, Marcelo Nicolas,Oliveira-Filho, Ricardo Martins,Boris Vargaftig, B.,Tavares-de-Lima, Wothan | Differential effects of formaldehyde exposure on the cell influx and vascular permeability in a rat model of allergic lung inflammation | 2010 |
| Lino-dos-Santos-Franco, A.,Gimenes, J. A.,Ligeiro-de-Oliveira, A. P.,Breithaupt-Faloppa, A. C.,Acceturi, B. G.,Vitoretti, L. B.,Machado, I. D.,Oliveira, R. M.,Farsky, S. H. P.,Moriya, H. T.,Tavares-de-Lima, W. | Formaldehyde inhalation reduces respiratory mechanics in a rat model with allergic lung inflammation by altering the nitric oxide/cyclooxygenase-derived products relationship | 2013 |
| Lino-dos-Santos-Franco, A.,Gimenes-Junior, J. A.,Ligeiro-de-Oliveira, A. P.,Breithaupt-Faloppa, A. C.,Acceturi, B. G.,Vitoretti, L. B.,Machado, I. D.,Oliveira-Filho, R. M.,Farsky, S. H.,Moriya, H. T.,Tavares-de-Lima, W. | Formaldehyde inhalation reduces respiratory mechanics in a rat model with allergic lung inflammation by altering the nitric oxide/cyclooxygenase-derived products relationship | 2013 |
| Lino-dos-Santos-Franco, Adriana,Gimenes-Junior, Joao Antonio,Ligeiro-de-Oliveira, Ana Paula,Breithaupt-Faloppa, Ana Cristina,Acceturi, Beatriz Golega,Vitoretti, Luana Beatriz,Machado, Isabel Daufenback,Oliveira-Filho, Ricardo Martins,Poliselli Farsky, Sandra Helena,Moriya, Henrique Takachi,Tavares-de-Lima, Wothan | Formaldehyde inhalation reduces respiratory mechanics in a rat model with allergic lung inflammation by altering the nitric oxide/cyclooxygenase-derived products relationship | 2013 |
| Lino-dos-Santos-Franco, A.,Shia, M. K.,Domingos, H. V.,Breithaupt-Faloppa, A. C.,de Oliveira, A. P. L.,Oliveira, R. M.,Vargaftig, B. B.,Tavares-de-Lima, W. | Connective tissue mast cells are the target of formaldehyde to induce tracheal hyperresponsiveness in rats: Putative role of leukotriene B-4 and nitric oxide | 2010 |
| Lipinska-Ojrzanowska, A.,Wiszniewska, M.,Walusiak-Skorupa, J. | Corrao's syndrome-a diagnostic dilemma in occupational setting | 2014 |
| Lipkin, G.,March, C.,Gowdey, J. | MAGNESIUM IN EPIDERMIS, DERMIS, AND WHOLE SKIN OF NORMAL AND ATOPIC SUBJECTS | 1964 |
| Lis, S. A. | Fiberoptic diisocyanate personal monitoring device | 2006 |
| Lisboa, B. P. | Separation and characterisation of formaldehydrogenic delta-4-3-oxo-C21-steroids by means of thin-layer chromatography on silica gel | 1966 |
| Littel-van den Hurk, S. V.,Mapletoft, J. W.,Arsic, N.,Kovacs-Nolan, J. | Immunopathology of RSV infection: prospects for developing vaccines without this complication | 2007 |
| Littorin, M.,Amon, A.,Broberg, K.,Sennbro, C. J.,Tinnerberg, H. | Eye and airway symptoms in low occupational exposure to toluene diisocyanate | 2007 |
| Littorin, M.,Rylander, L.,Skarping, G.,Dalene, M.,Welinder, H.,Stromberg, U.,Skerfving, S. | Exposure biomarkers and risk from gluing and heating of polyurethane: a cross sectional study of respiratory symptoms | 2000 |
| Liu, B.,Ichinose, T.,He, M.,Kobayashi, F.,Maki, T.,Yoshida, S.,Yoshida, Y.,Arashidani, K.,Takano, H.,Nishikawa, M.,Sun, G.,Shibamoto, T. | Lung inflammation by fungus, Bjerkandera adusta isolated from Asian sand dust (ASD) aerosol and enhancement of ovalbumin-induced lung eosinophilia by ASD and the fungus in mice | 2014 |
| Liu, B. Y.,Ichinose, T.,He, M.,Kobayashi, F.,Maki, T.,Yoshida, S.,Yoshida, Y.,Arashidani, K.,Takano, H.,Nishikawa, M.,Sun, G. F.,Shibamoto, T. | Lung inflammation by fungus, Bjerkandera adusta isolated from Asian sand dust (ASD) aerosol and enhancement of ovalbumin-induced lung eosinophilia by ASD and the fungus in mice | 2014 |
| Liu, C. C. | Precise and fast video assisted thoracoscopic bronchial sleeve resection | 2014 |
| Liu, D.,Zheng, Y.,Li, B.,Yao, H.,Li, R.,Zhang, Y.,Yang, X. | Adjuvant effects of gaseous formaldehyde on the hyper-responsiveness and inflammation in a mouse asthma model immunized by ovalbumin | 2011 |
| Liu, D. D.,Zheng, Y. D.,Li, B.,Yao, H. C.,Li, R.,Zhang, Y. P.,Yang, X. | Adjuvant effects of gaseous formaldehyde on the hyper-responsiveness and inflammation in a mouse asthma model immunized by ovalbumin | 2011 |
| Liu, F.,Zhao, Y.,Liu, Y. Q.,Liu, Y.,Sun, P.,Huang, M. M.,Liu, Y.,Dong, G. H. | Asthma and asthma related symptoms in 23,326 Chinese children in relation to indoor and outdoor environmental factors: The Seven Northeastern Cities (SNEC) Study | 2014 |
| Liu, F. Y.,Yu, X. L.,Han, Z. Y.,Wang, Y. | Clinical application of ultrasound-guided percutaneous biopsy in mediastinal lesions | 2008 |
| Liu, Guei-Sheung,Liu, Li-Fen,Lin, Che-Jen,Tseng, Jui-Cheng,Chuang, Ming-Ju,Lam, Hing-Chung,Lee, Jenn-Kuen,Yang, Lin-Cheng,Chan, Julie Hwa Yu,Howng, Shen-Long,Tai, Ming-Hong | Gene transfer of pro-opiomelanocortin prohormone suppressed the growth and metastasis of melanoma: Involvement of alpha-melanocyte-stimulating hormone-mediated inhibition of the nuclear factor kappa B/cyclooxygenase-2 pathway | 2006 |
| Liu, Wei,Yi, Dan-Dan,Guo, Jian-Li,Xiang, Zhu-Xing,Deng, Lin-Feng,He, Lei | Nuciferine, extracted from Nelumbo nucifera Gaertn, inhibits tumor-promoting effect of nicotine involving Wnt/beta-catenin signaling in non-small cell lung cancer | 2015 |
| Liu, Xue Jun,Gingrich, Jeffrey R.,Vargas-Caballero, Mariana,Dong, Yi Na,Sengar, Ameet,Beggs, Simon,Wang, Szu-Han,Ding, Hoi Ki,Frankland, Paul W.,Salter, Michael W. | Treatment of inflammatory and neuropathic pain by uncoupling Src from the NMDA receptor complex | 2008 |
| Liu, X. J.,Gingrich, J. R.,Vargas-Caballero, M.,Dong, Y. N.,Sengar, A.,Beggs, S.,Wang, S. H.,Ding, H. K.,Frankland, P. W.,Salter, M. W. | Treatment of inflammatory and neuropathic pain by uncoupling Src from the NMDA receptor complex | 2008 |
| Liu, Y. | The design and implementation of a Virtual Medical Centre for patient home care | 1998 |
| Lizaso, M. T.,Martinez, A.,Asturias, J. A.,Algorta, J.,Madariaga, B.,Labarta, N.,Tabar, A. I. | Biological standardization and maximum tolerated dose estimation of an Alternaria alternata allergenic extract | 2006 |
| Lloyd, R. S. T. E. P. H. E. N.,McCullough, A. M. A. N. D. A. K. | Cellular Responses to DNA-Protein Crosslinks | 2009 |
| Lloyd, R. S. T. E. P. H. E. N.,McCullough, A. M. A. N. D. A. K. | Cellular Responses to DNA-Protein Crosslinks | 2010 |
| Lloyd, R. S. T. E. P. H. E. N.,McCullough, A. M. A. N. D. A. K. | Cellular responses to DNA-protein crosslinks | 2011 |
| Lloyd, R. S. T. E. P. H. E. N.,McCullough, A. M. A. N. D. A. K. | Cellular responses to DNA-protein crosslinks | 2012 |
| Lloyd, R. S. T. E. P. H. E. N.,McCullough, A. M. A. N. D. A. K. | Cellular responses to DNA-protein crosslinks | 2013 |
| Lloyd, R. S. T. E. P. H. E. N.,McCullough, A. M. A. N. D. A. K. | Cellular responses to DNA-protein crosslinks | 2014 |
| Lloyd-Smith, M.,Sheffield-Brotherton, B. | Children's Environmental Health: Intergenerational Equity in Action-A Civil Society Perspective | 2008 |
| Loan, R.,Siebers, R.,Fitzharris, P.,Crane, J. | House dust-mite allergen and cat allergen variability within carpeted living room floors in domestic dwellings | 2003 |
| Lobb, R. R.,Pepinsky, B.,Leone, D. R.,Abraham, W. M. | The role of alpha 4 integrins in lung pathophysiology | 1996 |
| Locke, Darren,Bernstein, Steven,Lynch, Frank,Siami-Namini, Koushan,Walling, Jackie M.,Yonker, Thomas,Yarranton, Geoffrey | An IHC Screen For EphA3 Positive FFPE Tumors | 2013 |
| Loesel, L. S. | Immunopathologic study of chronic sinusitis: a proposal for atopic and non-atopic IgE-activated mast cell allergic inflammation | 2001 |
| Loetsch, J.,Hummel, T.,Warskulat, U.,Coste, O.,Haeussinger, D.,Geisslinger, G.,Tegeder, I. | Congenital Taurine Deficiency in Mice Is Associated with Reduced Sensitivity to Nociceptive Chemical Stimulation | 2014 |
| Lofstedt, H.,Westberg, H.,Selden, A. I.,Rudblad, S.,Bryngelsson, I. L.,Ngo, Y.,Svartengren, M. | Nasal and ocular effects in foundry workers using the hot box method | 2010 |
| Loginova, N. V.,Govorukhina, N. I.,Trotsenko Yu, A. | Metabolism of the Obligate Methylotroph Methylophilus-Methanolovorus | 1981 |
| Loginova, N. V.,Govorukhina, N. I.,Trotsenko Yu, A. | Autotrophic Metabolism of Methanol in Blastobacter-Viscosus | 1981 |
| Logue, J. N.,White, M. V.,Marchetto, D. J. | Pennsylvania's Asthma School Project and descriptive pilot investigation: a focus on environmental health tracking | 2007 |
| Lohmann, Sabine,Herold, Andrea,Bergauer, Tobias,Belousov, Anton,Betzl, Gisela,Demario, Mark,Dietrich, Manuel,Luistro, Leopoldo,Poignee-Heger, Manuela,Schostack, Kathy,Simcox, Mary,Walch, Heiko,Yin, Xuefeng,Zhong, Hua,Weisser, Martin | Gene expression analysis in biomarker research and early drug development using function tested reverse transcription quantitative real-time PCR assays | 2013 |
| Lojda, Z. | Studies on Di Peptidyl Amino Peptidase 4 Glycyl Proline Beta Naphthyl Amidase Part 2 Blood Vessels | 1979 |
| Lonc, E.,Plewa, K. | Microbiological Air Contamination in Poultry Houses | 2010 |
| Long, C. M.,Hosseinkhani, M. R.,Wang, Y.,Sriramarao, P.,Walcheck, B. | ADAM17 activation in circulating neutrophils following bacterial challenge impairs their recruitment | 2012 |
| Long, R. N.,Renne, E. P.,Basu, N. | Understanding the social context of the asgm sector in ghana: A qualitative description of the demographic, health, and nutritional characteristics of a small-scale gold mining community in Ghana | 2015 |
| Longuespee, Remi,Fleron, Maximilien,Pottier, Charles,Quesada-Calvo, Florence,Meuwis, Marie-Alice,Baiwir, Dominique,Smargiasso, Nicolas,Mazzucchelli, Gabriel,De Pauw-Gillet, Marie-Claire,Delvenne, Philippe,De Pauw, Edwin | Tissue Proteomics for the Next Decade? Towards a Molecular Dimension in Histology | 2014 |
| Lonnroth, E. C.,Shahnavaz, H. | Use of polymer materials in dental clinics, case study | 1997 |
| Loo, C. K. J.,Foty, R. G.,Wheeler, A. J.,Miller, J. D.,Evans, G.,Stieb, D. M.,Dell, S. D. | Do Questions Reflecting Indoor Air Pollutant Exposure from a Questionnaire Predict Direct Measure of Exposure in Owner-Occupied Houses? | 2010 |
| Lopes, P.,Duarte, J.,Celmlyn-Jones, J.,Porto, J.,Veríssimo, M. | An unusual cause of chronic cough-foreign body aspiration | 2015 |
| Lopez-Rico, R.,Moneo, I.,Rico, A.,Curiel, G.,Sanchez-Monge, R.,Salcedo, G. | Cereal alpha-amylase inhibitors cause occupational sensitization in the wood industry | 1998 |
| Lougheed, M. D.,Roos, J. O.,Waddell, W. R.,Munt, P. W. | Desquamative interstitial pneumonitis and diffuse alveolar damage in textile workers. Potential role of mycotoxins | 1995 |
| Lovvorn, J. R.,Gillingham, M. P. | A spatial energetics model of cadmium accumulation by diving ducks | 1996 |
| Low, A.,Nguyen, M. T. | Formaldehyde-induced systemic reaction | 2012 |
| Lu, Z.,Li, C. M.,Qiao, Y.,Liu, Y.,Yan, Y.,Yang, X. | Type II vanilloid receptor signaling system: one of the possible mechanisms for the rise in asthma cases | 2005 |
| Lu, Z. S.,Li, C. M.,Qiao, Y.,Liu, Y. S.,Yan, Y.,Yang, X. | Type II vanilloid receptor signaling system: One of the possible mechanisms for the rise in asthma cases | 2005 |
| Lubach, D. | [Diseases caused by diisocyanates. 1. Irritation of the respiratory system and skin] | 1978 |
| Ludwig, M. S.,Dallaire, M. J. | Structural Composition of Lung Parenchymal Strip and Mechanical-Behavior during Sinusoidal Oscillation | 1994 |
| Luebbert, J.,Gorsz, C.,Wolff, F.,Luedtke, H. | A Contribution to the Analysis and Evaluation of Contaminating Factors in Pig Breeding Plants Acting Irritatively on the Respiratory Tract | 1986 |
| Lukacs, N. W.,John, A.,Berlin, A.,Bullard, D. C.,Knibbs, R.,Stoolman, L. M. | E- and P-selectins are essential for the development of cockroach allergen-induced airway responses | 2002 |
| Lum, H.,Mitzner, W. | Effects of 10% formalin fixation on fixed lung volume and lung tissue shrinkage. A comparison of eleven laboratory species | 1985 |
| Luna, L. G.,Green, B. J.,Zhang, F.,Arnold, S. M.,Siegel, P. D.,Bartels, M. J. | Quantitation of 4,4'-methylene diphenyl diisocyanate human serum albumin adducts | 2014 |
| Lund, V. J. | Toxicity of Nasal Respiratory Mucosa in Humans | 1994 |
| Lundback, B. | Epidemiology of rhinitis and asthma | 1998 |
| Lundblad, L.,Lundberg, J. M.,Anggard, A. | Local and Systemic Capsaicin Pretreatment Inhibits Sneezing and the Increase in Nasal Vascular Permeability Induced by Certain Chemical Irritants | 1984 |
| Lundell, S.,Holmner, A.,Rehn, B.,Nyberg, A.,Wadell, K. | Telehealthcare in COPD: A systematic review and meta-analysis on physical outcomes and dyspnea | 2015 |
| Luo, Y. L.,Guo, H. M.,Zhang, Y. L.,Chen, P. X.,Zhu, Y. X.,Huang, J. H.,Zhou, W. L. | Cellular Mechanism Underlying Formaldehyde-Stimulated Cl- Secretion in Rat Airway Epithelium | 2013 |
| Luo, Zhi-Gang,Tang, Hao,Li, Bing,Zhu, Zhi,Ni, Can-Rung,Zhu, Ming-Hua | Genetic alterations of tumor suppressor ING1 in human non-small cell lung cancer | 2011 |
| Luongo, L.,Costa, B.,D'Agostino, B.,Guida, F.,Comelli, F.,Gatta, L.,Matteis, M.,Sullo, N.,De Petrocellis, L.,de Novellis, V.,Maione, S.,Di Marzo, V. | Palvanil, a non-pungent capsaicin analogue, inhibits inflammatory and neuropathic pain with little effects on bronchopulmonary function and body temperature | 2012 |
| Luppi, M. M.,de Melo, A. L.,Motta, R. O.,Malta, M. C.,Gardiner, C. H.,Santos, R. L. | Granulomatous nephritis in psittacines associated with parasitism by the trematode Paratanaisia spp | 2007 |
| Luppi, Marcela M.,de Melo, Alan L.,Motta, Rafael O. C.,Malta, Marcelo C. C.,Gardiner, C. H.,Santos, Renato L. | Granulomatous nephritis in psittacines associated with parasitism by the trematode Paratanaisia spp | 2007 |
| Lutes, M.,Worman, D. J. | An unanticipated complication of a novel approach to airway management | 2009 |
| Lutz, E. A.,Sharma, S.,Casto, B.,Needham, G.,Buckley, T. J. | Effectiveness of UV-C equipped vacuum at reducing culturable surface-bound microorganisms on carpets | 2010 |
| Lv, Yang,Yoshino, Hiroshi | Field Survey on Indoor Air Quality and Health in Japan | 2014 |
| Lv, Y. H.,Zhao, H. J.,Liang, Z. Y.,Dong, H. M.,Liu, L. Y.,Zhang, D. D.,Cai, S. X. | A Mobile Phone Short Message Service Improves Perceived Control of Asthma: A Randomized Controlled Trial | 2012 |
| Lwebuga-Mukasa, J. S.,Oyana, T. J.,Johnson, C. | Local ecological factors, ultrafine particulate concentrations, and asthma prevalence rates in Buffalo, New York, neighborhoods | 2005 |
| Lwebuga-Mukasa, J. S.,Oyana, T. J.,Wydro, P. | Risk factors for asthma prevalence and chronic respiratory illnesses among residents of different neighbourhoods in Buffalo, New York | 2004 |
| Lykens, M. G.,Clanton, T.,Gadek, J. E. | Worsening of Toluene Diisocyanate Tdi Asthma Despite Transfer to an Area of No Measurable Isocyanate Exposure | 1989 |
| Lykogeorgou, M.,Panaghiotopoulou-Gartagani, P.,Choulakis, M.,Kaditis, A.,Georgountzou, A.,Stefanaki, K.,Chrousos, G.,Gemou-Engesaeth, V. | Airway basement membrane thickness in children with asthma | 2013 |
| Lynch, P. P. | Improving the Home-Environment - Developments in Domestic Ventilation | 1995 |
| Lynch, R. M. | Modeling of exposure to carpet-cleaning chemicals preceding irritant-induced asthma in one patient | 2000 |
| Ma, Jie,Mannoor, Kaiissar,Gao, Lu,Tan, Afang,Guarnera, Maria A.,Zhan, Min,Shetty, Amol,Stass, Sanford A.,Xing, Lingxiao,Jiang, Feng | Characterization of microRNA transcriptome in lung cancer by next-generation deep sequencing | 2014 |
| Maberly, D. J.,Anthony, H. M. | Asthma management in a "clean" environment: 1. The effect of challenge with foods and chemicals on the peak flow rate | 1992 |
| Macan, J.,Plavec, D.,Kanceljak, B.,Milkovic-Kraus, S. | Exposure levels and skin reactivity to German cockroach (Blattella germanica) in Croatia | 2003 |
| MacIntosh, D. L.,Minegishi, T.,Kaufman, M.,Baker, B. J.,Allen, J. G.,Levy, J. I.,Myatt, T. A. | The benefits of whole-house in-duct air cleaning in reducing exposures to fine particulate matter of outdoor origin: A modeling analysis | 2010 |
| Mackenstedt, U.,Maerkel, K. | Experimental and Comparative Morphology of Radula Renewal in Pulmonates Mollusca Gastropoda | 1987 |
| Maddocks, M.,Nolan, C. M.,Man, W. D. C.,Polkey, M. I.,Hart, N.,Gao, W.,Rafferty, G. F.,Moxham, J.,Higginson, I. J. | Neuromuscular electrical stimulation to improve exercise capacity in patients with severe COPD: a randomised double-blind, placebo-controlled trial | 2016 |
| Madureira, J.,Paciencia, I.,Ramos, E.,Barros, H.,Fernandes, E. D. | Indoor Air Quality in Primary Schools and in Homes and its Impact on Children's Health - Study Design | 2012 |
| Maeda, Kayo,Takahashi, Kazuhisa,Takahashi, Fumiyuki,Tamura, Naoaki,Maeda, Masahiro,Kon, Shigeyuki,Uede, Toshimitsu,Fukuchi, Yoshinosuke | Distinct roles of osteopontin fragments in the development of the pulmonary involvement in sarcoidosis | 2001 |
| Maeda, M.,Ozaki, T.,Yasuoka, S.,Ogura, T. | Role of Alveolar Macrophages and Neutrophils in the Defense System against Infection of Pseudomonas-Aeruginosa in the Respiratory Tract and the Effect of Derivative of Muramyl Dipeptide | 1990 |
| Magee, Pam | Antiseptic drugs and disinfectants | 2012 |
| Magnussen, H.,Kirsten, A.,Eberhardt, R.,Schmidt, B.,Behr, J.,Stanzel, F.,Bonnet, R.,Herth, F. J. | Ct assessment of regional lung volume changes following endobronchial volume reduction therapy in emphysema patients using a synthetic adhesive hydrogel-foam | 2010 |
| Magnussen, H.,Kirsten, A. M.,Herth, F.,Eberhardt, R.,Stanzel, F.,Bonnet, R.,Behr, J.,Kramer, M. | Effect of fissure integrity on the efficacy of bronchoscopic lung volume reduction therapy using a peripheral acting tissue sealant in patients with advanced upper lobe predominant emphysema | 2011 |
| Maguire, R.,McCann, L.,Miller, M.,Kearney, N. | Nurse's perceptions and experiences of using of a mobile-phone-based Advanced Symptom Management System (ASyMS (c)) to monitor and manage chemotherapy-related toxicity | 2008 |
| Mahadeva, R.,Stewart, S.,Bilton, D.,Lomas, D. A. | Alpha-1 antitrypsin deficiency alleles and severe cystic fibrosis lung disease | 1999 |
| Mahajan, A. N.,Pancholi, S. S. | Pulsatile drug delivery for the treatment of nocturnal asthma: A chronopharmaceutical approach | 2010 |
| Mahakittikun, V.,Boitano, J. J.,Tovey, E.,Bunnag, C.,Ninsanit, P.,Matsumoto, T.,Andre, C. | Mite penetration of different types of material claimed as mite proof by the Siriraj chamber method | 2006 |
| Mahecha-Garcia, A. C.,Garcia-Menaya, J. M.,Bobadilla-Gonzalez, P.,Corrales-Vargas, S. I.,Chiarella-Privette, G. M.,Cordobes-Duran, C. | Anaphylaxis due to oxytocin | 2014 |
| Mahecha-García, A. C.,García-Menaya, J. M.,Bobadilla-González, P.,Corrales-Vargas, S. I.,Chiarella-Privette, G. M.,Cordobés-Duran, C. | Anaphylaxis due to oxytocin | 2014 |
| Mahmic, A.,Tovey, E. R.,Molloy, C. A.,Young, L. | House dust mite allergen exposure in infancy | 1999 |
| Maidment, C. D.,Jones, C. R.,Webb, T. L.,Hathway, E. A.,Gilbertson, J. M. | The impact of household energy efficiency measures on health: A meta-analysis | 2014 |
| Maiellaro, M.,Correa-Costa, M.,Vitoretti, L. B.,Gimenes, J. A.,Camara, N. O. S.,Tavares-de-Lima, W.,Farsky, S. H. P.,Lino-dos-Santos-Franco, A. | Exposure to low doses of formaldehyde during pregnancy suppresses the development of allergic lung inflammation in offspring | 2014 |
| Maiellaro, M.,Correa-Costa, M.,Vitoretti, L. B.,Gimenes Junior, J. A.,Camara, N. O.,Tavares-de-Lima, W.,Farsky, S. H.,Lino-dos-Santos-Franco, A. | Exposure to low doses of formaldehyde during pregnancy suppresses the development of allergic lung inflammation in offspring | 2014 |
| Maiellaro, M.,Macedo, R. S.,Mendes, E.,Tavares-de-Lima, W.,Ferreira, C. M.,Lino-dos-Santos-Franco, A. | High dose of formaldehyde exposure during pregnancy increases neutrophils lung influx evoked by ovalbumin in the offspring | 2016 |
| Mainiero, Fabrizio,Pepe, Angela,Yeon, Mitchell,Ren, Yunling,Giancotti, Filippo G. | The intracellular functions of alpha-6-beta-4 integrin are regulated by EGF | 1996 |
| Maiorano, Eugenio,Piattelli, Adriano,Favia, Gianfranco | Hepatocellular carcinoma metastatic to the oral mucosa: Report of a case with multiple gingival localizations | 2000 |
| Maisey, S. J.,Saunders, S. M.,West, N.,Franklin, P. J. | An extended baseline examination of indoor VOCs in a city of low ambient pollution: Perth, Western Australia | 2013 |
| Maitre, A.,Berode, M.,Pedrix, A.,Romazini, S.,Savolainen, H. | Biological monitoring of occupational exposure to toluene diisocyanate | 1993 |
| Majansky, A. N.,Salina, E. V.,Abajidy, M. A.,Ashkinazy, V. I.,Zaslavskaya, M. I. | Adhesive reactions of buccal epithelial cells on Candida albicans in children with bronchial asthma and gastroduodenitis | 2002 |
| Makatsori, M.,Scadding, G.,McKenzie, R.,Skypala, I.,Durham, S. | Silk contact anaphylaxis | 2011 |
| Małecka, B.,Maciejewski, J.,Maciejewska, J. | Exposure to tobacco smoke - Interdisciplinary problem | 2007 |
| Malek, F. A.,Mîritz, K. U.,Paul, I.,BienengrÑber, V.,FanghÑnel, J. | Influence of formaldehyde on the open field behavior of rats | 1997 |
| Malhotra, N.,Kumar, P.,Malhotra, P. | Bronchospasm due to bone cement vapors: An unusual complication [5] | 2004 |
| Malik, F.,Spencer, C. Y.,Richards, J. B.,Cromar, K. R.,Price, R. E.,Atkins, C. L.,Lazar, M. A.,Haque, I. U.,Johnston, R. A. | Resistin deficiency exacerbates allergen-induced pulmonary inflammation in mice | 2015 |
| Malik, Gunjan,Knowles, Lynn M.,Dhir, Rajiv,Xu, Shuping,Yang, Shuting,Ruoslahti, Erkki,Pilch, Jan | Plasma Fibronectin Promotes Lung Metastasis by Contributions to Fibrin Clots and Tumor Cell Invasion | 2010 |
| Malmberg, Annika B.,Yaksh, Tony L. | Pharmacology of the spinal action of ketorolac, morphine, ST-91, U50488H, and L-PIA on the formalin test and an isobolographic analysis of the NSAID interaction | 1993 |
| Malo, Jean-Luc,Cartier, Andre,Desjardins, Alain,Vande Weyer, Raymond,Vandenplas, Olivier | Occupational asthma caused by oak wood dust | 1995 |
| Malo, J. L.,Bernstein, I. L. | Other Chemical Substances Causing Occupational Asthma | 1993 |
| Malo, J. L.,Cartier, A.,Desjardins, A.,Weyer, R. V.,Vandenplas, O. | Occupational Asthma Caused by Oak Wood Dust | 1995 |
| Malo, J. L.,Cartier, A.,L'Archeveque, J.,Ghezzo, H.,Soucy, F.,Somers, J.,Dolovich, J. | Prevalence of occupational asthma and immunologic sensitization to guar gum among employees at a carpet-manufacturing plant | 1990 |
| Malo, J. L.,Cartier, A.,Pineault, L.,Dugas, M.,Desjardins, A. | Occupational asthma due to heated polypropylene | 1994 |
| Malone, T. A.,Stonestreet, B. S.,Goddard, M.,Oh, W. | Hemodynamic changes in a term newborn piglet model of patent ductus arteriosus | 1990 |
| Mandin, C.,Bonvallot, N.,Kirchner, S.,Keirsbulck, M.,Alary, R.,Cabanes, P. A.,Dor, F.,Le Moullec, Y.,Mullot, J. U.,Peel, A. E.,Rousselle, C. | Development of French Indoor Air Quality Guidelines | 2009 |
| Mandiracioglu, A.,Akgur, S.,Kocabiyik, N.,Sener, U. | Evaluation of neuropsychological symptoms and exposure to benzene, toluene and xylene among two different furniture worker groups in Izmir | 2011 |
| Mangum, James B.,Bermudez, Edilberto,Sar, Madhabananda,Everitt, Jeffrey I. | Osteopontin expression in particle-induced lung disease | 2004 |
| Manjra, A.,Berman, D.,Toerien, A.,Weinberg, E. G.,Potter, P. C. | The effects of a single treatment of an acaricide, Acarosan, and a detergent, Metsan, on Der p 1 allergen levels in the carpets and mattresses of asthmatic children | 1994 |
| Manno, M.,Rugge, M.,Cocheo, V. | Double Fatal Inhalation of Dichloromethane | 1992 |
| Mansfield, J. R.,Burrell, M. H.,Curl, V. J.,Valler, B. J. | Treatment of equine allergic diseases with allergy neutralization. A field study | 1998 |
| Mansour, M.,Lanphear, B. P.,Hornung, R.,Khoury, J.,Bernstein, D. I.,Menrath, W.,Decolongon, J. | A side-by-side comparison of sampling methods for settled, indoor allergens | 2001 |
| Manwani, D.,Biekert, J. J. | The erythroblastic Island | 2008 |
| Manwani, Deepa,Biekert, James J. | The erythroblastic Island | 2008 |
| Manzetti, S.,Andersen, O. | A review of emission products from bioethanol and its blends with gasoline. Background for new guidelines for emission control | 2015 |
| Mapp, C.,Moro, G.,Fabbri, L. | Toluene diisocyanate asthma: inhalation challenge and immunological studies | 1980 |
| Marc, M.,Zabiegala, B.,Namiegnik, J. | Testing and sampling devices for monitoring volatile and semi-volatile organic compounds in indoor air | 2012 |
| Marchand, C.,Le Calvé, S.,Mirabel, Ph,Glasser, N.,Casset, A.,Schneider, N.,de Blay, F. | Concentrations and determinants of gaseous aldehydes in 162 homes in Strasbourg (France) | 2008 |
| Marek, W.,Potthast, J.,Marczynski, B.,Baur, X. | [Toluene diisocyanate-induced bronchial hyperreactivity in an animal model of occupationally-induced lung diseases] | 1993 |
| Margenthaler, J. A.,Longo, W. E.,Virgo, K. S.,Johnson, F. E.,Grossmann, E. M.,Schifftner, T. L.,Henderson, W. G.,Khuri, S. F. | Risk factors for adverse outcomes following surgery for small bowel obstruction | 2006 |
| Marks, G. B.,Tovey, E. R.,Peat, J. K.,Salome, C. M.,Woolcock, A. J. | Variability and repeatability of house dust mite allergen measurement: implications for study design and interpretation | 1995 |
| Marks, Gerald S.,McLaughlin, Brian E.,Vreman, Hendrik J.,Stevenson, David K.,Nakatsu, Kanji,Brien, James F.,Pang, Stephen C. | Heme oxygenase activity and immunohistochemical localization in bovine pulmonary artery and vein | 1997 |
| Maronpot, R. R.,Miller, R. A.,Clarke, W. J.,Westerberg, R. B.,Decker, J. R.,Moss, O. R. | Toxicity of formaldehyde vapor in B6C3F1 mice exposed for 13 weeks | 1986 |
| Marotte, K.,Sabin, C.,Preville, C.,Moume-Pymbock, M.,Wimmerova, M.,Mitchell, E. P.,Imberty, A.,Roy, R. | X-ray structures and thermodynamics of the interaction of PA-IIL from pseudomonas aeruginosa with disaccharide derivatives | 2007 |
| Marozkina, N. V.,Wei, C.,Yemen, S.,Wallrabe, H.,Nagji, A. S.,Liu, L.,Morozkina, T.,Jones, D. R.,Gaston, B. | S-Nitrosoglutathione Reductase in Human Lung Cancer | 2012 |
| Marquardt, D. L.,Parker, C. W.,Sullivan, T. J. | Potentiation of Mast Cell Mediator Release by Adenosine | 1978 |
| Marsboom, Glenn R.,Vermeersch, Pieter,Pokreisz, Peter,Gillijns, Hilde,Pellens, Marijke,Collen, Desire,Janssens, Stefan | Endothelial progenitor cell function in hypoxic pulmonary hypertension | 2005 |
| Marsigliante, S.,Resta, L.,Muscella, A.,Vinson, G. P.,Marzullo, A.,Storelli, C. | AT1 angiotensin II receptor subtype in the human larynx and squamous laryngeal carcinoma | 1996 |
| Martin, I. R.,Wickens, K.,Patchett, K.,Kent, R.,Fitzharris, P.,Siebers, R.,Lewis, S.,Crane, J.,Holbrook, N.,Town, G. I.,Smith, S. | Cat allergen levels in public places in New Zealand | 2000 |
| Martin, J. G.,Opazosaez, A.,Du, T.,Tepper, R.,Eidelman, D. H. | Invivo Airway Reactivity - Predictive Value of Morphological Estimates of Airway Smooth-Muscle | 1992 |
| Martinat, Y.,Wallaert, B.,Just, J.,Seferian, A. | Perinatal exposure and allergic risk | 2011 |
| Martinez-Arguelles, D. B.,Papadopoulos, V. | Identification of Hot Spots of DNA Methylation in the Adult Male Adrenal in Response to In Utero Exposure to the Ubiquitous Endocrine Disruptor Plasticizer Di-(2-ethylhexyl) Phthalate | 2015 |
| Marty, F.,Montandon, D.,Gumener, R.,Zbrodowski, A. | Subcutaneous tissue in the scalp: anatomical, physiological, and clinical study | 1986 |
| Marwitz, S.,Abdullah, M.,Vock, C.,Fine, J. S.,Visvanathan, S.,Gaede, K. I.,Zabel, P.,Goldmann, T. | HOPEBAL: A novel tool to expand the methodological capabilities in pulmonary research | 2012 |
| Marwitz, S.,Pedersen, F.,Watz, H.,Vollmer, E.,Zabel, P.,Goldmann, T. | HOPE-BAL: A novel promising tool for pulmonary research and diagnostics | 2012 |
| Maser, Edmund,Friebertshaeuser, Jutta,Voelker, Bernhard | Purification, characterization and NNK carbonyl reductase activities of 11beta-hydroxysteroid dehydrogenase type 1 from human liver: Enzyme cooperativity and significance in the detoxification of a tobacco-derived carcinogen | 2003 |
| Mashiko, Naohiko | Studies on clinical electromyography. I. Studies on the follower potential | 1960 |
| Massanari, R. M. | Suppression of Tuberculin Hyper Sensitivity during Influenza Infection in Mice | 1979 |
| Massot, O.,Ott, M. | Practical guidelines for asthmatic and allergic patients dealing with hazardous constituents in their indoor environment | 2009 |
| Mastiholimath, V. S.,Dandagi, P. M.,Jain, S. S.,Gadad, A. P.,Kulkarni, A. R. | Time and pH dependent colon specific, pulsatile delivery of theophylline for nocturnal asthma | 2006 |
| Mateer, S.,Maltby, S.,Marks, E.,Goggins, B.,Horvat, J.,Hansbro, P.,Keely, S. | Immune cell mis-homing drives secondary organ inflammation in inflammatory bowel disease; a focus on the respiratory system | 2014 |
| Matheson, Melissa,Rynell, Ann-Christine,McClean, Melissa,Berend, Norbert | Relationship between airway microvascular leakage, edema, and baseline airway functions | 1998 |
| Matheson, M.,Rynell, A. C.,McClean, M.,Berend, N. | Relationship between airway microvascular leakage, edema, and baseline airway functions | 1998 |
| Mathiesen, C.,Hagerhall, C. | The 'antiporter module' of respiratory chain Complex I includes the MrpC/NuoK subunit - a revision of the modular evolution scheme | 2003 |
| Mathur, N.,Rastogi, S. K. | Respiratory effects due to occupational exposure to formaldehyde: Systematic review with meta-analysis | 2007 |
| Matsuda, Miho,Yamashita, Jun K.,Tsukita, Shoichiro,Furuse, Mikio | abLIM3 is a novel component of adherens junctions with actin-binding activity | 2010 |
| Matsui, E. C. | Environmental exposures and asthma morbidity in children living in urban neighborhoods | 2014 |
| Matsui, E. C.,Hansel, N. N.,McCormack, M. C.,Rusher, R.,Breysse, P. N.,Diette, G. B. | Asthma in the inner city and the indoor environment | 2008 |
| Matsui, E. C.,Simons, E.,Rand, C.,Butz, A.,Buckley, T. J.,Breysse, P.,Eggleston, P. A. | Airborne mouse allergen in the homes of inner-city children with asthma | 2005 |
| Matsui, S.,Nakazawa, T. | Occupational asthma in Japan | 1991 |
| Matsuki, H.,Shimizu, Y.,Misawa, K.,Takaoka, M.,Kasuga, H. | The Significance of Guanine in House Dust and Its Applications to Epidemiological Study | 1991 |
| Matsuura, Y. | Determination of 1 3 Bisisocyanatomethylcyclohexane in Working Atmosphere by High-Performance Liquid Chromatography | 1985 |
| Matte, T. D.,Jacobs, D. E. | Housing and health - Current issues and implications for research and programs | 2000 |
| Maurice, F.,Rivory, J. P.,Bousquet, J.,Michel, F. B. | Anaphylactic Shock with Formaldehyde | 1985 |
| Maurice, F.,Rivory, J. P.,Larsson, P. H.,Johansson, S. G.,Bousquet, J. | Anaphylactic shock caused by formaldehyde in a patient undergoing long-term hemodialysis | 1986 |
| Maurice, F.,Rivory, J. P.,Larsson, P. H.,Johansson, S. G. O.,Bousquet, J. | ANAPHYLACTIC SHOCK CAUSED BY FORMALDEHYDE IN A PATIENT UNDERGOING LONG-TERM HEMODIALYSIS | 1986 |
| Maus, Ulrich,Huwe, Julia,Ermert, Leander,Ermert, Monika,Seeger, Werner,Lohmeyer, Juergen | Molecular pathways of monocyte emigration into the alveolar air space of intact mice | 2002 |
| Mautz, W. J.,Kleinman, M. T.,Bhalla, D. K.,Phalen, R. F. | Respiratory tract responses to repeated inhalation of an oxidant and acid gas-particle air pollutant mixture | 2001 |
| Max, W.,Sung, H. Y.,Shi, Y. | The cost of secondhand smoke exposure at home in California | 2014 |
| Maxey, M. L.,Novick, T. L.,Nottingham, J. M. | Repair of diaphragmatic defect complicating peritoneal dialysis with laparoscopic and thoracoscopic approach | 2014 |
| May, L.,Law, A.,Wilson, J.,Bencheqroun, H. | A rare case of a big heart and hematuria | 2014 |
| Mayhew, T. M.,Jenkins, H.,Todd, B.,Clifton, V. L. | Maternal asthma and placental morphometry: effects of severity, treatment and fetal sex | 2008 |
| Mazanceva, G. P. | Estimation of the Body Weight of Some Soil Invertebrates on the Basis of Their Linear Size Measurements | 1976 |
| Mazdisnian, F.,Fujiwara, M.,Kukes, G.,Sassoon, C. | Study of the trachea after teflon injection | 2013 |
| Mazzella, di Bosco M. | Occupational respiratory allergic diseases | 1982 |
| Mbawuike, I. N.,Herscowitz, H. B. | The Role of Membrane Gangliosides in Murine Alveolar Macrophage-Mediated Suppression of the Immune Response | 1988 |
| McAdam, J. G.,Leicester, R. J. | Infection in endoscopy: Protection of staff | 1993 |
| McBride, D.,Keil, T.,Grabenhenrich, L.,Dubakiene, R.,Drasutiene, G.,Fiocchi, A.,Dahdah, L.,Sprikkelman, A. B.,Schoemaker, A. A.,Roberts, G.,Grimshaw, K.,Kowalski, M. L.,Stanczyk-Przyluska, A.,Sigurdardottir, S.,Clausen, M.,Papadopoulos, N. G.,Mitsias, D.,Rosenfeld, L.,Reche, M.,Pascual, C.,Reich, A.,Hourihane, J.,Wahn, U.,Mills, E. N.,Mackie, A.,Beyer, K. | The EuroPrevall birth cohort study on food allergy: baseline characteristics of 12,000 newborns and their families from nine European countries | 2011 |
| McBride, J. T.,Chuang, C. | A technique for quantitating airway size from bronchial casts | 1985 |
| McCabe, B. F. | The problem of the collapsing upper lateral cartilage | 1979 |
| McCann, P.,Namjou, K.,Roller, C.,McMillen, G.,Kamat, P. | IV-VI semiconductor lasers for gas phase biomarker detection - art. no. 675603 | 2007 |
| McCormack, M. C.,Breysse, P. N.,Hansel, N. N.,Matsui, E. C.,Tonorezos, E. S.,Curtin-Brosnan, J.,Williams, D. L.,Buckley, T. J.,Eggleston, P. A.,Diette, G. B. | Common household activities are associated with elevated particulate matter concentrations in bedrooms of inner-city Baltimore pre-school children | 2007 |
| McDermott, R. D.,Wilson, T. L.,Blacker, K. J.,Maute, C.,Ozdener, M. H.,Dalton, P. H. | Functional and inflammatory consequences of veterinary gross anatomy lab enrollment: Effects of formaldehyde on chemosensation | 2009 |
| McDonald, M. D.,Smith, C. P.,Walsh, P. J. | The physiology and evolution of urea transport in fishes | 2006 |
| McElhinney, D. B.,Khan, J. H.,Babcock, W. D.,Hall, T. S. | Thoracic organ donor characteristics associated with successful lung procurement | 2001 |
| McFadyen, James D.,Kaplan, Zane S. | Platelets Are Not Just for Clots | 2015 |
| McGinn, J. D.,Plant, R. L. | Acoustic analysis of upper airway obstruction in the excised human larynx | 2002 |
| McGregor, Douglas,Bolt, Hermann,Cogliano, Vincent,Richter-Reichhelm, Hans-Bernhard | Formaldehyde and glutaraldehyde and nasal cytotoxicity: Case study within the context of the 2006 IPCS human framework for the analysis of a cancer mode of action for humans | 2006 |
| McGregor, D.,Bolt, H.,Cogliano, V.,Richter-Reichhelm, H. B. | Formaldehyde and glutaraldehyde and nasal cytotoxicity: Case study within the context of the 2006 IPCS human framework for the analysis of a cancer mode of action for humans | 2006 |
| McGwin, G.,Lienert, J.,Kennedy, J. I. | Formaldehyde exposure and asthma in children: a systematic review | 2010 |
| McGwin, G., Jr.,Lienert, J.,Kennedy, J. I., Jr. | Formaldehyde exposure and asthma in children: a systematic review | 2011 |
| McGwin, G.,Lienert, J.,Kennedy, J. I. | Formaldehyde Exposure and Asthma in Children: A Systematic Review (vol 118, pg 313, 2010) | 2012 |
| McKee, W. D.,Cochrane, C. G.,Farr, R. S. | A Clinical Study of an Unusual Case of Asthma Associated with Urticaria Pigmentosa Boy Chlorpheniramine Anti Hist Methylsergide Maleate Immunol P Methoxyphenethyl Methylamine Condensation Product with Formaldehyde Metab Guinea-Pig | 1966 |
| McKenna Jr, R. J. | Bronchial blockers for lung volume reduction surgery: Where are we and where are we going? | 2005 |
| McLaughlin, S. A.,McKinney, P. E. | Antacid-induced hypermagnesemia in a patient with normal renal function and bowel obstruction | 1998 |
| McMullin, D. R.,Nsiama, T. K.,Miller, J. D. | Secondary metabolites from Penicillium corylophilum isolated from damp buildings | 2014 |
| McNulty, W.,Zoumot, Z.,Hopkinson, N. S. | Bronchoscopic and percutaneous approaches to lung volume reduction | 2013 |
| McSharry, C.,Vesper, S.,Wymer, L.,Howieson, S.,Chaudhuri, R.,Wright, G. R.,Thomson, N. C. | Decreased FEV1 % in asthmatic adults in Scottish homes with high Environmental Relative Moldiness Index values | 2015 |
| Medvedev, O.,Marshall, A.,Antonov, A. | User-friendly interface for the Smartphone-based self management of pulmonary rehabilitation | 2008 |
| Mehta, H.,Sampson, H. A.,Nowak-Wegrzy, A. | Allergic reactions tovaccines | 2012 |
| Meijer, G. G.,Postma, D. S.,van der Heide, S.,de Reus, D. M.,Roorda, R. J.,Koeter, G. H.,van Aalderen, W. M. | Exogenous stimuli and circadian peak expiratory flow variation in allergic asthmatic children | 1996 |
| Meijer, G. G.,van der Heide, S.,Postma, D. S.,de Reus, D. M.,Koeter, G. H.,van Aalderen, W. M. | House dust mite exposure in asthmatic and healthy children: the difference is carpeting | 1995 |
| Meissner, S.,Knels, L.,Koch, E. | Improved three-dimensional Fourier domain optical coherence tomography by index matching in alveolar structures | 2010 |
| Melchior Gerster, F.,Brenna Hopf, N.,Pierre Wild, P.,Vernez, D. | Airborne exposures to monoethanolamine, glycol ethers, and benzyl alcohol during professional cleaning: a pilot study | 2014 |
| Melendi, G. A.,Hoffman, S. J.,Karron, R. A.,Irusta, P. M.,Laham, F. R.,Humbles, A.,Schofield, B.,Pan, C. H.,Rabold, R.,Thumar, B.,Thumar, A.,Gerard, N. P.,Mitzner, W.,Barnum, S. R.,Gerard, C.,Kleeberger, S. R.,Polack, F. P. | C5 modulates airway hyperreactivity and pulmonary eosinophilia during enhanced respiratory syncytial virus disease by decreasing C3a receptor expression | 2007 |
| Melgoza, E. L.,Sereno, L.,Ciurana, J.,Rosell, A. | Automation design and simulation of a stent | 2012 |
| Melgoza, E. L.,Sereno, L.,Rosell, A.,Ciurana, J. | An integrated parameterized tool for designing a customized tracheal stent | 2012 |
| Melgoza, E. L.,Vallicrosa, G.,Sereno, L.,Ciurana, J.,Rodriguez, C. A. | Rapid tooling using 3D printing system for manufacturing of customized tracheal stent | 2014 |
| Melin, J.,Spanne, M.,Johansson, R.,Bohgard, M.,Skarping, G.,Colmsjo, A. | Characterization of thermally generated aerosols from polyurethane foam | 2001 |
| Mendell, M. J. | Indoor residential chemical emissions as risk factors for respiratory health effects in infants and children: A review | 2006 |
| Mendell, M. J. | Indoor residential chemical emissions as risk factors for respiratory and allergic effects in children: a review | 2007 |
| Mendell, M. J. | Indoor residential chemical exposures with asthma and allergy in infants and children: A review | 2007 |
| Mendell, M. J.,Heath, G. A. | Do indoor pollutants and thermal conditions in schools influence student performance? A critical review of the literature | 2005 |
| Mendes, A.,Pereira, C.,Mendes, D.,Aguiar, L.,Neves, P.,Silva, S.,Batterman, S.,Teixeira, J. P. | Indoor Air Quality and Thermal ComfortResults of a Pilot Study in Elderly Care Centers in Portugal | 2013 |
| Meng, G.,Geme, J. W. S.,Waksman, G. | Repetitive Architecture of the Haemophilus influenzae Hia Trimeric Autotransporter | 2008 |
| Meng, J.,Barnes, C. S.,Rosenwasser, L. J. | Identity of the fungal species present in the homes of asthmatic children | 2012 |
| Meng, Y. Y.,Wilhelm, M.,Rull, R. P.,English, P.,Ritz, B. | Traffic and outdoor air pollution levels near residences and poorly controlled asthma in adults | 2007 |
| Mensing, T.,Marek, W.,Potthast, J.,Baur, X. | The pathomechanism of oxidating substances in the respiratory tract in a hairdressing salon | 1994 |
| Mentese, S. | Are Kindergartens Healthy Enough for the Children? | 2010 |
| Mercer, M. J.,Joubert, G.,Ehrlich, R. I.,Nelson, H.,Poyser, M. A.,Puterman, A.,Weinberg, E. G. | Socioeconomic status and prevalence of allergic rhinitis and atopic eczema symptoms in young adolescents | 2004 |
| Merchant, J. A.,Kilburn, K. H.,O'Fallon, W. M.,Hamilton, J. D.,Lumsden, J. C. | Byssinosis and chronic bronchitis among cotton textile workers | 1972 |
| Merget, Rolf,Stollfuss, Jens,Wiewrodt, Rainer,Fruehauf, Heiko,Koch, Ursula,Bolm-Audorff, Ulrich,Bienfait, Hein-Guenter,Hiltl, Gerd,Schulte-Werninghaus, Gerhard | Diagnostic tests in enzyme allergy | 1993 |
| Merrifield, A. J.,King, S. J. | The oesophageal obturator airway: A study of cadaver lund ventilation through obturator airways and tracheal tubes | 1981 |
| Meyer, H. W.,Wurtz, H.,Suadicani, P.,Valbjorn, O.,Sigsgaard, T.,Gyntelberg, F. | Molds in floor dust and building-related symptoms among adolescent school children: a problem for boys only? | 2005 |
| Meyerholz, D. K. | Immunohistochemical localization of arginase-1 expression in B6 and BALB/c mice | 2013 |
| Meynadier, J. M.,Meynadier, J.,Peyron, J. L.,Peyron, L. | Clinical Aspects of Perfume Allergic Cutaneous Manifestations | 1986 |
| Michael Alberts, W. | What are the odds that exposures will lead to disease? Protecting your health: Avoiding chemicals and other toxic elements | 1997 |
| Michaels, M.,Patel, A.,Cohen, Z. | A hairy situation: An unusual case of lipoid pneumonia | 2011 |
| Michalik, J.,Raczynska-Bojanowska, K. | Oxidation of Methanol by Facultative and Obligate Methylotrophs | 1976 |
| Mihrshahi, S.,Marks, G.,Vanlaar, C.,Tovey, E.,Peat, J. | Predictors of high house dust mite allergen concentrations in residential homes in Sydney | 2002 |
| Mikami, Y.,Numaguchi, Y.,Kobayashi, N.,Fuwa, S.,Hoshikawa, Y.,Saida, Y. | Therapeutic effects of percutaneous vertebroplasty for vertebral metastases | 2012 |
| Mikkelsen, A. B.,Schlunssen, V.,Sigsgaard, T.,Schaumburg, I. | Determinants of wood dust exposure in the Danish furniture industry | 2002 |
| Mikov, M. | Chronic bronchitis and ventilation capacity in three groups of industrial workers (Serbocroatian) | 1974 |
| Milanese, M.,Riccio, A. M.,Gamalero, C.,De Giovanni, B.,Brichetto, L.,Baroffio, M.,Crimi, E.,Brusasco, V.,Canonica, G. W. | A model of allergen-driven human airway contraction: beta(2) pathway dysfunction without cytokine involvement | 2005 |
| Milde, L. N.,Milde, J. H.,Lanier, W. L.,Michenfelder, J. D. | Comparison of the effects of isoflurane and thiopental on neurologic outcome and neuropathology after temporary focal cerebral ischemia in primates | 1988 |
| Miles, R. | Preventing asthma through housing interventions: How supportive is the US policy environment? | 2005 |
| Milewicz, H. J.,Boutz, D. R.,Kelly, A.,Hamilton, E.,Lavender, P.,Marcotte, E.,Thomas, N. S. B. | Effects of reducing the DNA replication protein Mcm7 on the chromatin-bound proteome | 2013 |
| Miller, B. E.,Chapin, R. E.,Pinkerton, K. E.,Gilmore, L. B.,Maronpot, R. R.,Hook, G. E. R. | Quantitation of Silica-Induced Type Ii Cell Hyperplasia by Using Alkaline Phosphatase Histochemistry in Glycol Methacrylate Embedded Lung | 1987 |
| Miller, C. S. | Toxicant-induced loss of tolerance | 2001 |
| Miller, J. D. | House dust mites are unable to penetrate or colonize suede | 2011 |
| Miller, J. D. | Health effects from mold and dampness in housing in western societies: early epidemiology studies and barriers to further progress | 2011 |
| Miller, N. J.,Daniels, C. B.,Schurch, S.,Schoel, W. M.,Orgeig, S. | The surface activity of pulmonary surfactant from diving mammals | 2006 |
| Miller, Natalie J.,Postle, Anthony D.,Orgeig, Sandra,Koster, Grielof,Daniels, Christopher B. | The composition of pulmonary surfactant from diving mammals | 2006 |
| Miller, R. L. | Breathing freely: The need for asthma research on gene-environment interactions | 1999 |
| Miller, Shelly L.,Scaramella, Peter,Campe, Joseph,Goss, Cynthia W.,Diaz-Castillo, Sandra,Hendrikson, Ed,DiGuiseppi, Carolyn,Litt, Jill | An assessment of indoor air quality in recent Mexican immigrant housing in Commerce City, Colorado | 2009 |
| Milner, J.,Chalabi, Z.,Vardoulakis, S.,Wilkinson, P. | Housing interventions and health: Quantifying the impact of indoor particles on mortality and morbidity with disease recovery | 2015 |
| Milosevic, J.,Zhang, T.,Contreras, A.,Trudeau, J. T.,Wenzel, S. E. | Thyroid peroxidase identified as a novel human airway epithelial peroxidase | 2014 |
| Mineshita, M.,Slebos, D. J. | Bronchoscopic interventions for chronic obstructive pulmonary disease | 2014 |
| Mingomataj, E. C.,Gjata, E.,Xhixha, F.,Hyso, E. | A case of isocyanate-induced asthma possibly complicated by food allergy after peanut consumption: a case report | 2008 |
| Mir, L. | Air pollution and calls to SIS Medicines in Paris and Petit Couronne region between 2000 and 2003 | 2007 |
| Miragall, F.,Kadmon, G.,Schachner, M. | Expression of L1 and N-Cam Cell Adhesion Molecules during Development of the Mouse Olfactory System | 1989 |
| Mirmohammadi, M.,Ibrahim, M. H.,Saraji, J. N. | Modeling of hexamethylene diisocyanate and psychrometric parameters and other effective factors in the polyurethane factories | 2010 |
| Mise, N.,Fernandez, I. E.,Eickelberg, O. | SIRT1 activator resveratrol ameliorates pulmonary fibrosis in mice | 2013 |
| Mise, N.,Fernandez, I. E.,Eickelberg, O. | SIRT1 activator resveratrol ameliorates pulmonary fibrosis in mice | 2014 |
| Mishra, A. K.,Rotti, S. B.,Sahai, A.,Madanmohan,,Narayan, K. A. | Byssinosis among male textile workers in Pondicherry: a case-control study | 2003 |
| Mistry, R.,Wickramasingha, N.,Ogston, S.,Singh, M.,Devasiri, V.,Mukhopadhyay, S. | Wheeze and urban variation in South Asia | 2004 |
| Mitchel, J.,McGill, M.,Antoniak, S.,Shore, S. A.,Mackman, N.,Fredberg, J. J.,Park, J. A. | Compressive stress induces reactive oxygen species-dependent release of tissue factor in airway epithelial cells from normal and asthmatic donors | 2014 |
| Mitchell, B. | Building materials can be a major source of indoor air pollution | 2013 |
| Mitchell, J. L.,Liu, Y.,Orr, K.,Chen, Y.,Yim, W.,Wang, R. | A new technique of virtual modeling of individual patient airways in obstructive sleep apnea | 2014 |
| Mitchell, J. L.,Liu, Y.,Wang, R. C.,Orr, K.,Yim, W.,Chen, Y. | A new technique of virtual modeling of individual patient airways in obstructive sleep apnea | 2014 |
| Mitchell, S. J.,Godoy, L.,Shabazz, K.,Horn, I. B. | Internet and Mobile Technology Use Among Urban African American Parents: Survey Study of a Clinical Population | 2014 |
| Mitha, N.,Levy, J.,Annesi-Maesano, I.,Lafortune, J.,Magnier, A. M.,Ibanez, G. | [Indoor air quality and adult asthma] | 2013 |
| Mitha, N.,Lévy, J.,Annesi-Maesano, I.,Lafortune, J.,Magnier, A. M.,Ibanez, G. | Indoor air quality and adult asthma | 2013 |
| Mitman, G. | Cockroaches, Housing, and Race A History of Asthma and Urban Ecology in America | 2008 |
| Mitroi, M.,Capitanescu, A.,Georgescu, C. V.,Mogoanta, C. A.,Popescu, C.,Georgescu, M.,Mitroi, G.,Ionita, E. | Expression pattern of CK7 and CK20 in nasal polyps, at patients with chronic rhinosinusitis with nasal polyposis | 2011 |
| Miyahara, E.,Itagaki, T.,Kuwahara, M.,Kameda, A.,Kodama, M. | A case of elderly lung cancer with malignant pleural effusion and pneumothorax treated by CBDCA, PEM and BEV | 2014 |
| Miyoshi, Tatsu,Shirakusa, Takayuki,Ishikawa, Yuichi,Iwasaki, Akinori,Shiraishi, Takeshi,Makimoto, Yoshifumi,Iwasaki, Hiroshi,Nabeshima, Kazuki | Possible mechanism of metastasis in lung adenocarcinomas with a micropapillary pattern | 2005 |
| Mlacha, S. Z. K.,Peret, T. C. T.,Kumar, N.,Romero-Steiner, S.,Hotopp, J. C. D.,Ishmael, N.,Grinblat-Huse, V.,Riley, D. R.,Erdman, D. D.,Carlone, G. M.,Sampson, J.,Scott, J. A. G.,Tettelin, H. | Transcriptional adaptation of pneumococci and human pharyngeal cells in the presence of a virus infection | 2013 |
| Möbs, C.,Rauber, M.,Hertl, M.,Pfützner, W. | Immune regulatory mechanisms of specific immunotherapy with chemically modified allergen (allergoid) in patients suffering from birch pollen allergy | 2013 |
| Mochizuki, H.,Ohki, Y.,Arakawa, H.,Tokuyama, K.,Morikawa, A. | Effect of ultrasonically nebulized distilled water on airway epithelial cell swelling in guinea pigs | 1999 |
| Moegel, I.,Feltens, R.,Simon, J.,Röder-Stolinski, C.,Herberth, G.,Lehmann, I. | The chlorobenzene induced inflammatory response in lung epithelial cells can be prevented by antioxidants | 2010 |
| Moghissi, A. A. | Strategies for the Development of Indoor Air-Quality Standards | 1991 |
| Moldoveanu, A. M.,Decusara, M. | Home exposure to some indoor air pollutants | 1998 |
| Molinaro, R. J.,Bernstein, J. M.,Koury, S. T. | Localization and quantitation of eotaxin mRNA in human nasal polyps | 2003 |
| Molloy, S. B.,Cheng, M.,Galbally, I. E.,Keywood, M. D.,Lawson, S. J.,Powell, J. C.,Gillett, R.,Dunne, E.,Selleck, P. W. | Indoor air quality in typical temperate zone Australian dwellings | 2012 |
| Momeni, Arash,Mohammadi, Mohammad H. | Respiratory delivery of theophylline by size-targeted starch microspheres for treatment of asthma | 2009 |
| Monier, S.,Hemery, M. L.,Demoly, P.,Dhivert-Donnadieu, H. | Occupational asthma to wood dust | 2008 |
| Moniruzzaman, S.,Hagerhed Engman, L.,James, P.,Sigsgaard, T.,Thorne, P. S.,Sundell, J.,Bornehag, C. G. | Levels of endotoxin in 390 Swedish homes: determinants and the risk for respiratory symptoms in children | 2011 |
| Monse, C.,Bunger, J.,Bruning, T.,Merget, R. | Generation and characterization of airborne ethyl 2-cyanoacrylate atmospheres in a human whole-body exposure unit | 2014 |
| Montanaro, A. | Occupational asthma associated with low molecular weight antigens | 1992 |
| Montanaro, A. | Formaldehyde in the workplace and in the home - Exploring its clinical toxicology | 1996 |
| Montano, D. | Chemical and biological work-related risks across occupations in Europe: A review | 2014 |
| Montefort, S.,Baker, J.,Roche, W. R.,Holgate, S. T. | The Distribution of Adhesive Mechanisms in the Normal Bronchial Epithelium | 1993 |
| Monticello, T. M.,Morgan, K. T. | Chemically-induced nasal carcinogenesis and epithelial cell proliferation: a brief review | 1997 |
| Montuschi, P.,Del Cecato, P.,Ciabattoni, G. | In vitro testing for lung toxicity: A method for distinguishing between immune- and non-immune-mediated reactions to xenobiotics | 1996 |
| Montuschi, P.,DelCecato, P.,Ciabattoni, G. | In vitro testing for lung toxicity: A method for distinguishing between immune- and non-immune-mediated reactions to xenobiotics | 1996 |
| Moody, G. N.,Zeno, B. R. | Localization and treatment of bronchopleural fistula through capnography | 2010 |
| Mooi, R. | Non-Respiratory Podia of Clypeasteroids Echinodermata Echinoidea I. Functional Anatomy | 1986 |
| Moon, H. J.,Yoon, Y. R. | Investigation of Physical Characteristics of Houses and Occupants' Behavioural Factors for Mould Infestation in Residential Buildings | 2010 |
| Moore, B. D.,Hyde, D.,Miller, L.,Wong, E.,Frelinger, J.,Schelegle, E. S. | Allergen and Ozone Exacerbate Serotonin-Induced Increases in Airway Smooth Muscle Contraction in a Model of Childhood Asthma | 2012 |
| Moore, V.,Robertson, A.,Burge, S. | Can solvent inhalation be the cause of occupational asthma? | 2014 |
| Moradi, A. V.,Hashemian, S.,Peyghan, A. A.,Baei, M. T. | Role of Diameter, Model, and Length of Boron Nitride Nanotubes in Adsorption of Formaldehyde | 2015 |
| Morales, C.,Basomba, A.,Carreira, J.,Sastre, A. | Anaphylaxis Produced by Rubber Glove Contact Case Reports and Immunological Identification of the Antigens Involved | 1989 |
| Moran, M. M.,del Camino, D.,Stricker, J.,Witek, J.,Chong, J. A.,Hayward, N. J. | TRPA1 is a potential target for the treatment of asthma | 2008 |
| Morck, D. W.,Costerton, J. W.,Bolingbroke, D. O.,Ceri, H.,Boyd, N. D.,Olson, M. E. | A Guinea-Pig Model of Bovine Pneumonic Pasteurellosis | 1990 |
| Morello-Frosch, R.,Zuk, M.,Jerrett, M.,Shamasunder, B.,Kyle, A. D. | Understanding The Cumulative Impacts Of Inequalities In Environmental Health: Implications For Policy | 2011 |
| Morgan, D. R.,Musa, M. | Self inflicted death following inhalation and ingestion of Builders Polyurethane expandable foam | 2010 |
| Morgan, K. T.,Gross, E. A.,Patterson, D. L. | Distribution Progression and Recovery of Acute Formaldehyde-Induced Inhibition of Nasal Mucociliary Function of F-344 Rats | 1986 |
| Morgan, K. T.,Jiang, X. Z.,Gross, E. A.,Patterson, D. L. | A Procedure for Study of the Effects of Irritant Gases on the Nasal Mucociliary Apparatus of Rats | 1985 |
| Morgan, K. T.,Monticello, T. M. | Airflow, gas deposition, and lesion distribution in the nasal passages | 1990 |
| Morgan, K. T.,Patterson, D. L.,Gross, E. A. | Localization of Areas of Inhibition of Nasal Muco Ciliary Function in Rats Following in-Vivo Exposure to Formaldehyde | 1983 |
| Morgan, K. T.,Patterson, D. L.,Gross, E. A. | Frog Palate Mucociliary Apparatus Structure Function and Response to Formaldehyde Gas | 1984 |
| Morgan, K. T.,Patterson, D. L.,Gross, E. A. | Responses of the Nasal Mucociliary Apparatus of F-344 Rats to Formaldehyde Gas | 1986 |
| Moriguchi, N.,Yamamoto, S.,Isokawa, S.,Andou, A.,Miyata, H. | Granulocyte functions and changes in ability with age in newborns; Report no. 1: Flow cytometric analysis of granulocyte functions in whole blood | 2006 |
| Morinaga, Y.,Yanagihara, K.,Araki, N.,Yamada, K.,Nakamura, S.,Izumikawa, K.,Seki, M.,Kakeya, H.,Yamamoto, Y.,Yamada, Y.,Kohno, S.,Kohno, S. | Legionella pneumophila Induces MUC5AC via ERK-NFkB Pathway in NCI-H292 Cells | 2009 |
| Morino, M.,Suzuki, T.,Ito, M.,Krulwich, T. A. | Purification and Functional Reconstitution of a Seven-Subunit Mrp-Type Na+/H+ Antiporter | 2014 |
| Morisaki, Hiroshi,Katayama, Tomihiro,Kotake, Yoshifumi,Takeda, Junzo,Suematsu, Makoto | In Vivo Analyses of Platelet-Endothelial Interaction during Halothane Anesthesia | 2002 |
| Morishita, M.,Keeler, G. J.,McDonald, J. D.,Wagner, J. G.,Young, L. H.,Utsunomiya, S.,Ewing, R. C.,Harkema, J. R. | Source-to-receptor pathways of anthropogenic PM2.5 in Detroit, Michigan: Comparison of two inhalation exposure studies | 2009 |
| Morisset, M.,Moneret-Vautrin, D. A.,Kanny, G.,Guenard, L.,Beaudouin, E.,Flabbee, J.,Hatahet, R. | Thresholds of clinical reactivity to milk, egg, peanut and sesame in immunoglobulin E-dependent allergies: Evaluation by double-blind or single-blind placebo-controlled oral challenges | 2003 |
| Morita, Y.,Matsumoto, Y.,Miyamoto, T.,Horiuchi, Y. | A Clinical Study of Concanavalin a Induced Histamine Release Utilizing a Single Isotopic Enzymatic Assay of Histamine | 1978 |
| Morris, J. B. | Upper Respiratory-Tract Metabolism of Inspired Alcohol-Dehydrogenase and Mixed-Function Oxidase Substrate Vapors under Defined Air-Flow Conditions | 1993 |
| Morrison, G. C.,Little, J. C.,Xu, Y.,Rao, M.,Enke, D. | Gas-phase exposure history derived from material-phase concentration profiles | 2007 |
| Morrow, C. E.,Schwartz, J. S.,Sutherland, D. E.,Simmons, R. L.,Ferguson, R. M.,Kjellstrand, C. M.,Najarian, J. S. | Predictive value of thallium stress testing for coronary and cardiovascular events in uremic diabetic patients before renal transplantation | 1983 |
| Morrow, W. R.,Taylor, A. F.,Kinsella, J. P.,Lally, K. P.,Gerstmann, D. R.,deLemos, R. A. | Effect of ductal patency on organ blood flow and pulmonary function in the preterm baboon with hyaline membrane disease | 1995 |
| Moscato, G.,Biscaldi, G.,Cottica, D.,Candura, F. | Specific bronchial provocation tests in occupational asthma and methods of stimulation: case report | 1983 |
| Moschioni, Monica,Emolo, Carla,Biagini, Massimiliano,Maccari, Silvia,Pansegrau, Werner,Donati, Claudio,Hilleringmann, Markus,Ferlenghi, Ilaria,Ruggiero, Paolo,Sinisi, Antonia,Pizza, Mariagrazia,Norais, Nathalie,Barocchi, Michele A.,Masignani, Vega | The Two Variants of the Streptococcus pneumoniae Pilus 1 RrgA Adhesin Retain the Same Function and Elicit Cross-Protection In Vivo | 2010 |
| Moshammer, H. | Environment and lungs in childhood and adolescence | 2008 |
| Moshammer, H.,Neuberger, M. | Lung function and life expectancy of asbestos workers | 2008 |
| Moturi, N. W. | Risk factors for indoor air pollution in rural households in Mauche division, Molo district, Kenya | 2010 |
| Mounier-Geyssant, E.,Oury, V.,Mouchot, L.,Paris, C.,Zmirou-Navier, D. | Exposure of hairdressing apprentices to airborne hazardous substances | 2006 |
| Mourao, C. B. F.,Oliveira, F. N.,Carvalho, A. C. E.,Arenas, C. J.,Duque, H. M.,Goncalves, J. C.,Macedo, J. K. A.,Galante, P.,Schwartz, C. A.,Mortari, M. R.,Santos, Mdma,Schwartz, E. F. | Venomic and pharmacological activity of Acanthoscurria paulensis (Theraphosidae) spider venom | 2013 |
| Movahedi, R.,Sharma, S. | Bone cement implantation syndrome during total elbowarthroplasty with a patent foramen ovale | 2012 |
| Msezane, Lambda P.,Katz, Mark H.,Gofrit, Ofer N.,Shalhav, Arieh L.,Zorn, Kevin C. | Hemostatic agents and instruments in laparoscopic renal surgery | 2008 |
| Mueller, Barbara M.,Reisfeld, Ralph A.,Edgington, Thomas S.,Ruf, Wolfram | Expression of tissue factor by melanoma cells promotes efficient hematogenous metastasis | 1992 |
| Mueller, J. U.,Bruckner, T.,Triebig, G. | Exposure study to examine chemosensory effects of formaldehyde on hyposensitive and hypersensitive males | 2013 |
| Mueller-Anneling, L.,Avol, E.,Peters, J. M.,Thorne, P. S. | Ambient endotoxin concentrations in PM10 from Southern California | 2004 |
| Mukai, M.,Woods, L. W.,Stump, S.,Ebel, J. G.,Levitt, A. S.,Frey, M. W.,Smith, J.,Uzal, F. A.,Poppenga, R. H.,Puschner, B. | Detection of diisocyanates in nesting material associated with mortality in pigeon chicks | 2014 |
| Mukund, Swarnalatha,Adams, Michael W. W. | Characterization of a novel tungsten-containing formaldehyde ferredoxin oxidoreductase from the hyperthermophilic archaeon, Thermococcus litoralis: A role for tungsten in peptide catabolism | 1993 |
| Mulero, V.,Pelegrin, P.,Sepulcre, M. P.,Munoz, J.,Meseguer, J. | A fish cell surface receptor defined by a mAb mediates leukocyte aggregation and deactivation | 2001 |
| Muller, K.,Mohring, M.,Furll, M.,Sobiraj, A.,Gmeiner, K.,Schoon, H. A. | Pulmonal thrombosis in female adult cattle considering the clinical history | 2009 |
| Munir, A. K.,Bjorksten, B.,Einarsson, R.,Schou, C.,Ekstrand-Tobin, A.,Warner, A.,Kjellman, N. I. | Cat (Fel d I), dog (Can f I), and cockroach allergens in homes of asthmatic children from three climatic zones in Sweden | 1994 |
| Munoz, Nilda M.,Hamann, Kimm J.,Rabe, Klaus F.,Sano, Hiroyuki,Zhu, Xiangdong,Leff, Alan R. | Augmentation of eosinophil degranulation and LTC4 secretion by integrin-mediated endothelial cell adhesion | 1999 |
| Mur, J. M.,Meyer-Bisch, C.,Cavelier, C. | Study of the pulmonary function of workers exposed to isocyanates at a polyurethane foam injection station | 1982 |
| Murphy, N. G.,Varney, S. M.,Tallon, J. M.,Thompson, J. R.,Blanc, P. D. | Fatal occupational exposure to trimethylsilyl-diazomethane | 2009 |
| Murphy, S. D.,Ulrich, C. E. | MULTI-ANIMAL TEST SYSTEM FOR MEASURING EFFECTS OF IRRITANT GASES AND VAPORS ON RESPIRATORY FUNCTION OF GUINEA PIGS | 1964 |
| Murphy, T. M.,Mitchell, R. W.,Halayko, A.,Roach, J.,Roy, L.,Kelly, E. A.,Munoz, N. M.,Stephens, N. L.,Leff, A. R. | Effect of Maturational Changes in Myosin Content and Morphometry on Airway Smooth-Muscle Contraction | 1991 |
| Murray, A.,David, M. F.,Freigang, B.,Luciuk, G. H.,Zimmerman, B. | SECONDHAND CIGARETTE SMOKE WORSENS SYMPTOMS IN CHILDREN WITH ASTHMA | 1986 |
| Muse, W. T., Jr.,Anthony, J. S.,Bergmann, J. D.,Burnett, D. C.,Crouse, C. L.,Gaviola, B. P.,Thomson, S. A. | Chemical and toxicological evaluation of pyrotechnically disseminated terephthalic acid smoke | 1998 |
| Mustafa, K. Y.,Bos, W.,Lakha, A. S. | Byssinosis in Tanzanian textile workers | 1979 |
| Mutsaers, Steven E. | The mesothelial cell | 2004 |
| Muus, P.,Ridgway, P.,Douglas, J. S.,Bouhuys, A. | Histamine Content of Tracheal and Lung Tissue as a Function of Age in Rats | 1974 |
| Muzaffar, F.,Haroon, T. S. | Airborne contact dermatitis: Changing etiological paradigm | 2014 |
| Myong, J. P.,Koo, J. W.,Kim, K. H.,Kim, H. R.,Jang, T. W. | Pneumoconiosis due to environmental cement dust exposure among the residents near the portland cement factories in Korea | 2013 |
| Nabe, M. | Studies on Clinicopathological Changes in Interstitial Lung Diseases I. Fibronectin in Bronchoalveolar Lavage Fluid | 1987 |
| Nagarajan, Revathi,Ponnuraj, Karthe | Cloning, expression, purification, crystallization and preliminary X-ray diffraction analysis of glyceraldehyde-3-phosphate dehydrogenase from Streptococcus agalactiae NEM316 | 2014 |
| Nagata, Makoto | Differential effects of corticosteroids and theophylline on the adhesive interaction between eosinophils and endothelial cells | 2004 |
| Nagata, M.,Sedgwick, J. B.,Vrtis, R.,Busse, W. W. | Endothelial cells upregulate eosinophil superoxide generation via VCAM-1 expression | 1999 |
| Nagata, M.,Tabe, K.,Choo, J. H.,Sakamoto, Y.,Matsuo, H. | Effect of immunotherapy on the production of eosinophil adhesion-inducing activity from mononuclear cells in house-dust-mite-sensitive bronchial asthma | 1998 |
| Nagata, M.,Tabe, K.,Hong Choo, J.,Sakamoto, Y.,Matsuo, H. | Effect of immunotherapy on the production of eosinophil adhesion-inducing activity from mononuclear cells in house-dust-mite-sensitive bronchial asthma | 1998 |
| Nagata, M.,Yamamoto, H.,Tabe, K.,Sakamoto, Y.,Matsuo, H. | Eosinophil-adhesion-inducing activity produced by antigen-stimulated mononuclear cells involves GM-CSF | 2000 |
| Nagata, N.,Kawajiri, T.,Hayashi, T.,Nakanishi, K.,Nikaido, Y.,Kido, M. | Interstitial pneumonitis and fibrosis associated with the inhalation of hair spray | 1997 |
| Nair, P.,Spande, J. I.,Whalen, W. J. | Marking the Tip Location of Oxygen Partial Pressure Micro Electrodes or Glass Micro Pipettes | 1980 |
| Nakamura, Kazuo,Kariyazono, Hiroko | Influence of Endocrine-disrupting Chemicals on the Immune System | 2010 |
| Nakamura, T. | Experimental Pulmonary Candidiasis in Modified Rabbits Histo Pathological Ultrastructural and Enzyme Cytochemical Studies of Tissue Reactions | 1984 |
| Nakanishi, M.,Furuno, T. | Molecular basis of neuroimmune interaction in an in vitro coculture approach | 2008 |
| Nakanishi, M.,Furuno, T.,Shibata, M. | Molecular basis of neuroimmune interaction in an in vitro coculture approach | 2009 |
| Nakano, Yoshihis,Hori, Mitsuhiko,Yamamoto, Keiji,Otsuka, Saburo | Percutaneous absorption type preparation | 2000 |
| Nakazawa, T. | Occupational asthma due to alkyl cyanoacrylate | 1990 |
| Nankervis, H.,Pynn, E. V.,Boyle, R. J.,Rushton, L.,Williams, H. C.,Hewson, D. M.,Platts-Mills, T. | House dust mite reduction and avoidance measures for treating eczema | 2015 |
| Nashev, Dimitar,Toshkova, Katia,Salasia, S. Isrina O.,Hassan, Abdulwahed A.,Lammler, Christoph,Zschock, Michael | Distribution of virulence genes of Staphylococcus aureus isolated from stable nasal carriers | 2004 |
| Natsis, K.,Didagelos, M.,Manoli, S. M.,Papathanasiou, E.,Sofidis, G.,Anastasopoulos, N. | A bicarotid trunk in association with an aberrant right subclavian artery. Report of two cases, clinical impact, and review of the literature | 2011 |
| Nava, C.,Arbosti, G.,Briatico-Vangosa, G.,Cirla, A. M.,Marchisio, M.,Zedda, S. | Pathology produced by isocyanates: methods of immunological investigation | 1975 |
| Navarro, B. G.,Bourg, V.,Calderon, L.,Facenda, E.,Labrada, A. | Morphological response induced by DPPC liposomes encapsulating allergens from dermatophagoides siboney | 2010 |
| Naz, S.,Nazir, G.,Iram, S.,Mohammad, M.,Umair,,Qari, I. H.,Mohammad, S. | Perceptions of cadaveric dissection in anatomy teaching | 2011 |
| Nazaroff, W. W.,Weschler, C. J. | Cleaning products and air fresheners: exposure to primary and secondary air pollutants | 2004 |
| Nedellec, V.,Mosqueron, L.,Desqueyroux, H. | Decrease in the health impact of road traffic in French urban areas attributable to European emissions standards Euro IV and V. I. Meta-analysis of epidemiological studies to derive the exposure-response function in children | 2009 |
| Neeraj, M.,Rastogi, S. K.,Srivastava, A. K. | Occupational epidemiological studies of respiratory disorders due to exposure to organic solvents - Systematic review with meta-analysis | 2001 |
| Neill, A.,Anderson, P. | Observational cadaveric study of emergency bystander cricothyroidotomy with a ballpoint pen by untrained junior doctors and medical students | 2012 |
| Nelson, H. S. | Advances in upper airway diseases and allergen immunotherapy | 2007 |
| Netterlid, E.,Hindsen, M.,Ekqvist, S.,Henricson, K. A.,Bruze, M. | Young individuals with atopic disease and asthma or rhinoconjunctivitis may have clinically relevant contact allergies | 2014 |
| Nettum, John A. | Bronchoalveolar casting using formalin-fixed canine lungs and a low viscosity silicone rubber | 1993 |
| Neuenschwander, H.,Molto, A.,Bianchi, M. | External nasal dilator strips (ENDS) may improve breathlessness in cancer patients | 2006 |
| Neufeld, Gera,Shraga-Heled, Niva,Lange, Tali,Guttmann-Raviv, Noga,Herzog, Yael,Kessler, Ofra | Semaphorins in cancer | 2005 |
| Neuman, T. R.,Hengesteg, A.,Lepage, R. P.,Kaufman, K. R.,Woodson, G. E. | Three-dimensional motion of the arytenoid adduction procedure in cadaver larynges | 1994 |
| Nevihostenyi, G.,Gyetvai, G.,Kiss, B.,Csizer, Z.,Istok, M.,Kosa, E. | Investigation of House Dust Allergy in Children | 1979 |
| Newman, N. M.,Sherrey, J. H.,Megirian, D. | Infant death and sheepskin rugs | 1983 |
| Newman, S. P.,Steed, K. P.,Reader, S. J.,Pavia, D.,Sohal, A. K. | An in vitro study to assess facial and ocular deposition from Respimat (R) Soft Mist (TM) inhaler | 2007 |
| Neyrinck, A. P.,Van De Wauwer, C.,Geudens, N.,Rega, F. R.,Verleden, G. M.,Wouters, P.,Lerut, T. E.,Van Raemdonck, D. E. | Comparative study of donor lung injury in heart-beating versus non-heart-beating donors | 2006 |
| Ng, N.,Lam, D.,Paulus, P.,Batzer, G.,Horner, A. A. | House dust extracts have both T(H)2 adjuvant and tolerogenic activities | 2006 |
| Ngajilo, D. | Respiratory health effects in poultry workers | 2014 |
| Nguyen, P.,Haley, K.,Hylkema, M. N.,Kobzik, L. | Epigenetic analysis of human fetal airway epithelium: Prenatal programming by maternal smoking? | 2012 |
| Nick, J. A.,Nichols, M.,Chacon, C.,Jones, M.,Nichols, D. P.,Janssen, J. S.,Saavedra, M. T.,Taylor-Cousar, J. L.,Sagel, S. D.,Martiniano, S. L. | Nontuberculous mycobacteria: Who to treat, How to treat, When to stop | 2011 |
| Nields, H. M.,Snider, G. L.,Breuer, R.,Christensen, T. | Reversible Pancreatic Elastase-Induced Bronchial Secretory-Cell Metaplasia in the Rat | 1991 |
| Nielsen, G. D.,Hougaard, K. S.,Larsen, S. T.,Hammer, M.,Wolkoff, P.,Clausen, P. A.,Wilkins, C. K.,Alarie, Y. | Acute airway effects of formaldehyde and ozone in BALB/c mice | 1999 |
| Nielsen, G. D.,Larsen, S. T.,Olsen, O.,Lovik, M.,Poulsen, L. K.,Glue, C.,Wolkoff, P. | Do indoor chemicals promote development of airway allergy? | 2007 |
| Nielsen, G. D.,Larsen, S. T.,Wolkoff, P. | Recent trend in risk assessment of formaldehyde exposures from indoor air | 2012 |
| Nielsen, J.,Sango, C.,Winroth, G.,Hallberg, T.,Skerfving, S. | Systemic reactions associated with polyisocyanate exposure | 1985 |
| Nielsen, J.,Welinder, H.,Skerfving, S. | Allergic Airway Disease Caused by Methyl Tetrahydrophthalic Anhydride in Epoxy Resin | 1989 |
| Nieman, Gary F.,Clark, William R., Jr. | Effects of wood and cotton smoke on the surface properties of pulmonary surfactant | 1994 |
| Nii, Akihiko,Sone, Saburo,Orino, Etsuko,Ogura, Takeshi | Induction of a 26-kDa membrane-form tumor necrosis factor (TNF)-alpha in human alveolar macrophages | 1993 |
| Nijhawan, S.,Majid, S. F.,Sedrak, M.,Sandler, B. J.,Jacobsen, G. R.,Talamini, M. A.,Wittgrove, A.,Horgan, S. | First human NOTES® experience for sleeve gastrectomy at University of California at San Diego (UCSD) | 2011 |
| Nikaido, T.,Tanino, Y.,Wang, X.,Fukuhara, N.,Misa, K.,Sato, Y.,Togawa, R.,Suzuki, Y.,Uematsu, M.,Fukuhara, A.,Sato, S.,Saito, J.,Yokouchi, H.,Munakata, M. | Danaparoid attenuates lipopolysaccharide-induced inflammation in bronchial epithelial cells | 2015 |
| Nikolaou, M.,Valavanis, C.,Aravantinos, G.,Fountzilas, G.,Tamvakis, N.,Lekka, I.,Arapantoni-Dadiot, P.,Zizi, A.,Ghiconti, I.,Economopoulos, Th,Pectasides, D. | Kit expression in male germ cell tumors | 2007 |
| Nisam, M. R.,Zbinden, A.,Chesrown, S.,Barnett, D.,Gold, W. M. | Distribution and Pharmacological Release of Histamine in Canine Lung in-Vivo | 1978 |
| Nishino, M.,Mathai, S. K.,O'Donnell, W. J.,Kradin, R. L. | Intraalveolar fibrin is associated with poor outcomes in cryptogenic organizing pneumonia | 2013 |
| Nishioka, Y.,Levy, J. I.,Norris, G. A.,Bennett, D. H.,Spengler, J. D. | A risk-based approach to health impact assessment for input-output analysis. Part 2: Case study of insulation | 2005 |
| Nissan, R.,Trope, M.,Zhang, C. D.,Chance, B. | Dual wavelength spectrophotometry as a diagnostic test of the pulp chamber contents | 1992 |
| Nittner, D.,Lambertz, I.,Schramm, A.,Mestdagh, P.,Vandesompele, J.,Dyer, M. A.,Schulte, J.,Marine, J. C. | Dicer1 is a synthetic lethal partner of tumour suppressor p53 | 2010 |
| Niven, R. M.,Fletcher, A. M.,Pickering, C. A.,Fishwick, D.,Warburton, C. J.,Simpson, J. C.,Francis, H.,Oldham, L. A. | Chronic bronchitis in textile workers | 1997 |
| Nizami, R. M. | Formaldehyde: Its Hazards At Work And In The Home | 1097 |
| Noble, G. K. | The adaptive modifications of the arboreal tadpoles of Hoplophryne and the torrent tadpoles of Staurois | 1929 |
| Noctor, G.,Mhamdi, A.,Chaouch, S.,Han, Y.,Neukermans, J.,Marquez-Garcia, B.,Queval, G.,Foyer, C. H. | Glutathione in plants: an integrated overview | 2012 |
| Noguchi, H.,Kephart, G. M.,Campbell, R. J.,Li, J. T.,Leiferman, K. M.,Trocme, S. D.,Gleich, G. J. | Tissue Eosinophilia and Eosinophil Degranulation in Orbital Pseudotumor | 1991 |
| Noguchi, T.,Shiga, Y.,Koga, K.,Shigematsu, A. | A method to improve a gas leak on mask ventilation in the patient with a nasogastric tube | 2001 |
| Noisel, N.,Bouchard, M.,Carrier, G. | The Impact Of Reduction Of The Occupational Exposure Limit For Formaldehyde In Quebec: A Re-Evaluation Of Health Risks | 2005 |
| Noisel, N.,Bouchard, M.,Carrier, G. | Evaluation of the health impact of lowering the formaldehyde occupational exposure limit for Quebec workers | 2007 |
| Nomura, K.,Saito, H.,Nishikawa, T.,Aiba, M. | Clinical outcome thirty-five years after unilateral adrenalectomy in a patient with idiopathic hyperaldosteronism and re-evaluation of the resected gland by immunohistochemical staining | 2011 |
| Noonan, C. W.,Ward, T. J. | Asthma randomized trial of indoor wood smoke (ARTIS): rationale and methods | 2012 |
| Norback, D.,Bjornsson, E.,Janson, C.,Palmgren, U.,Boman, G. | Current asthma and biochemical signs of inflammation in relation to building dampness in dwellings | 1999 |
| Norback, D.,Cai, G. H. | Dampness, indoor mould, fungal DNA and respiratory health - molecular methods in indoor epidemiology | 2015 |
| Norback, D.,Nordstrom, K.,Zhao, Z. H. | Carbon dioxide (CO2) demand-controlled ventilation in university computer classrooms and possible effects on headache, fatigue and perceived indoor environment: an intervention study | 2013 |
| Norback, D.,Walinder, R.,Wieslander, G.,Smedje, G.,Erwall, C.,Venge, P. | Indoor air pollutants in schools: nasal patency and biomarkers in nasal lavage | 2000 |
| Norbäck, D.,Wieslander, G.,Nordström, K.,Wålinder, R.,Venge, P. | The effect of air humidification on symptoms and nasal patency, tear film stability, and biomarkers in nasal lavage: A 6 weeks' longitudinal study | 2000 |
| Norback, D.,Zock, J. P.,Plana, E.,Heinrich, J.,Svanes, C.,Sunyer, J.,Kunzli, N.,Villani, S.,Olivieri, M.,Soon, A.,Jarvis, D. | Mould and dampness in dwelling places, and onset of asthma: the population-based cohort ECRHS | 2013 |
| Nordby, K. C.,Notø, H.,Eduard, W.,Skogstad, M.,Kjuus, H. | Prospective monitoring of exposure and lung function among cement production workers-is drop out from the study associated with respiratory health at inclusion? | 2014 |
| Norgaard, A. W.,Hansen, J. S.,Sorli, J. B.,Levin, M.,Wolkoff, P.,Nielsen, G. D.,Larsen, S. T. | Pulmonary Toxicity of Perfluorinated Silane-Based Nanofilm Spray Products: Solvent Dependency | 2014 |
| Normand, J. C.,Grange, F.,Hernandez, C.,Ganay, A.,Davezies, P.,Bergeret, A.,Prost, G. | Occupational Asthma after Exposure to Azodicarbonamide: Report of Four Cases | 1989 |
| Northridge, J.,Ramirez, O. F.,Stingone, J. A.,Claudio, L. | The role of housing type and housing quality in urban children with asthma | 2010 |
| Northup, S.,Presant, L.,Kilburn, K. H. | Lacinilene C-7 methyl ether, an agent from cotton dust causing leukocyte chemotaxis and histamine release (byssinosis) | 1976 |
| Noss, I.,Wouters, I. M.,Bezemer, G.,Metwali, N.,Sander, I.,Raulf-Heimsoth, M.,Heederik, D. J.,Thorne, P. S.,Doekes, G. | beta-(1,3)-Glucan exposure assessment by passive airborne dust sampling and new sensitive immunoassays | 2009 |
| Noss, I.,Wouters, I. M.,Visser, M.,Heederik, D. J.,Thorne, P. S.,Brunekreef, B.,Doekes, G. | Evaluation of a low-cost electrostatic dust fall collector for indoor air endotoxin exposure assessment | 2008 |
| Notø, H. N.,Eduard, | Exposure assessment of thoracic aerosol in an international prospective study of cement production workers | 2013 |
| Nowak, N.,Kakade, P. P.,Annapragada, A. V. | Computational fluid dynamics simulation of airflow and aerosol deposition in human lungs | 2003 |
| Nowicka, D.,Nawrot, U.,Wlodarczyk, K.,Pajaczkowska, M.,Patrzalek, A.,Pecak, A.,Mozdyniewicz, P.,Fleischer, M. | Detection of dermatophytes in human nail and skin dust produced during podiatric treatments in people without typical clinical signs of mycoses | 2016 |
| Nowicka, D.,Nawrot, U.,Włodarczyk, K.,Pajaczkowska, M.,Patrzałek, A.,Pecak, A.,Mozdyniewicz, P.,Fleischer, M. | Detection of dermatophytes in human nail and skin dust produced during podiatric treatments in people without typical clinical signs of mycoses | 2016 |
| Nuristani, A.,Almuti, W.,Mal, H.,Alsyaguh, R.,Horani, M. H.,Salim, O. | Zinc toxicity secondary to denture adhesive misdiagnosed as adrenoleukodystrophy | 2011 |
| Nurmatov, U. B.,Tagieva, N.,Semple, S.,Devereux, G.,Sheikh, A. | Volatile organic compounds and risk of asthma and allergy: a systematic review and meta-analysis of observational and interventional studies | 2013 |
| Nurmatov, U. B.,Tagiyeva, N.,Semple, S.,Devereux, G.,Sheikh, A. | Volatile organic compounds and risk of asthma and allergy: A systematic review of observational and interventional studies | 2013 |
| Nurmatov, U. B.,Tagiyeva, N.,Semple, S.,Devereux, G.,Sheikh, A. | Volatile organic compounds and risk of asthma and allergy: A systematic review | 2015 |
| Nussbaum, C.,Gloning, A.,Pruenster, M.,Frommhold, D.,Bierschenk, S.,Genzel-Boroviczeny, O.,von Andrian, U. H.,Quackenbush, E.,Sperandio, M. | Neutrophil and endothelial adhesive function during human fetal ontogeny | 2013 |
| Nyan, O. A.,Walraven, G. E.,Banya, W. A.,Milligan, P.,Van Der Sande, M.,Ceesay, S. M.,Del Prete, G.,McAdam, K. P. | Atopy, intestinal helminth infection and total serum IgE in rural and urban adult Gambian communities | 2001 |
| Nyan, O. A.,Walraven, G. E. L.,Banya, W. A. S.,Milligan, P.,Van der Sande, M.,Ceesay, S. M.,Del Prete, G.,McAdam, Kpwj | Atopy, intestinal helminth infection and total serum IgE in rural and urban adult Gambian communities | 2001 |
| Nyan, O. A.,Walraven, G. E. L.,Banya, W. A. S.,Milligan, P.,Van Der Sande, M.,Ceesay, S. M.,Del Prete, G.,McAdam, K. P. W. J. | Atopy, intestinal helminth infection and total serum IgE in rural and urban adult Gambian communities | 2001 |
| O'Connor G, T.,Walter, M.,Mitchell, H.,Kattan, M.,Morgan, W. J.,Gruchalla, R. S.,Pongracic, J. A.,Smartt, E.,Stout, J. W.,Evans, R.,Crain, E. F.,Burge, H. A. | Airborne fungi in the homes of children with asthma in low-income urban communities: The Inner-City Asthma Study | 2004 |
| O'Connor, G. T.,Gold, D. R. | Cockroach allergy and asthma in a 30-year-old man | 1999 |
| O'Donnell, J. J.,Birukova, A. A.,Beyer, E. C.,Birukov, K. G. | Gap Junction Protein Connexin43 Exacerbates Lung Vascular Permeability | 2014 |
| O'Keeffe, D. T.,Anthony, C. | The Stoichiometry of Respiration Driven Proton Translocation in Pseudomonas Am-1 and in a Mutant Lacking Cytochrome C | 1978 |
| O'Rourke, M. K.,Lebowitz, M. D. | ENVIRONMENTAL ALLERGENS AND THE DEVELOPMENT OF CHRONIC AND ALLERGIC OBSTRUCTIVE LUNG DISEASES | 1995 |
| O'Sullivan, Nancy L.,Raja, Rajiv,Montgomery, Paul C. | Lymphocyte adhesive interactions with lacrimal gland acinar epithelial cells in primary culture | 1995 |
| Obando, S. U.,Fernández-Nieto, M.,Sastre, J. | Occupational asthma due to polyvinyl chloride and methylmethacrylate, “hidden in an adhesive” | 2013 |
| Ochmann, Marlene,Lemonnier, Francois,Damotte, Diane,Delarue, Richard,Ben-Neriah, Susana,Milpied, Noel,Lamy, Thierry,Tilly, Herve,Gascoyne, Randy D.,Steidl, Christian,Gaulard, Philippe,Leroy, Karen | Mutations of STAT6 DNA Binding Domain Are Characteristic of PMBL and Belong to the Genetic Signature of This Entity | 2012 |
| Ochoa, J.,Roque, A. I.,Daza, J. A. | Hepatic cholangiocarcinoma in a feline and anatomopathologic and clinical findings, compatible with Feline Infectious Peritonitis | 2012 |
| Oczypok, E. A.,Milutinovic, P. S.,Manni, M. L.,Khare, A.,Alcorn, J. F.,Ray, A.,Oury, T. D. | Receptor for advanced glycation end products is an early upstream activator of the Th2 inflammatory immune response in allergic asthma | 2014 |
| Odelowo, E. O.,Komolafe, O. F. | Diagnosis, management and complications of oesophageal and airway foreign bodies | 1990 |
| Ogawa, S.,Fukazawa, R.,Kamisago, M.,Abe, M.,Ohkubo, T.,Watanabe, M.,Akao, M.,Watanabe, M.,Suzuki, N.,Katsube, Y. | Reduced shear stress induces senescence of vascular wall and thrombus formation in patients with giant coronary aneurysm after Kawasaki disease | 2009 |
| Oh, M. N.,Cho, M. J.,Baek, H. K.,Cho, K. S.,Kang, J. H.,Kim, Y.,Kwak, J. Y. | A case of hypersensitivity pneumonitis in an automobile paint sprayer | 2008 |
| Ohatani, T.,Mizuashi, M.,Nakagawa, S.,Aiba, S. | Diesel exhaust particles and formaldehyde deviate human immune system to Th2-dominant pattern by affecting both T cells and dendritic cells | 2004 |
| Ohayon, J. | Identification of increased oral eosinophils in patients with eosinophilic esophagitis (EE) using oral rinse analysis: Proof of concept | 2012 |
| Ohshima, M.,Miyake, M.,Takeda, M.,Kamijima, M.,Sakamoto, T. | Staphylococcal Enterotoxin B Causes Proliferation of Sensory C-Fibers and Subsequent Enhancement of Neurogenic Inflammation in Rat Skin | 2011 |
| Ohtsuka, R.,Shuto, Y.,Fujie, H.,Takeda, M.,Harada, T.,Itagaki, S. | Response of respiratory epithelium of BN and F344 rats to formaldehyde inhalation | 1997 |
| Ohtsuka, Ryoichi,Shuto, Yasufumi,Fujie, Hideaki,Takeda, Makio,Harada, Takanori,Itagaki, Shin-Ichi | Response of respiratory system in brown Norway and Fischer 344 rats to formaldehyde inhalation | 1998 |
| Ohtsuka, R.,Shuto, Y.,Fujie, H.,Takeda, M.,Harada, T.,Itagaki, S. I. | Response of respiratory system in brown Norway and Fischer 344 rats to formaldehyde inhalation | 1997 |
| Ohwada, Akihiko,Takahashi, Hideki,Nagaoka, Isao,Kira, Shiro | Biliary glycoprotein mRNA expression is increased in primary lung cancer, especially in squamous cell carcinoma | 1994 |
| Oie, L.,Hersoug, L. G.,Madsen, J. O. | Residential exposure to plasticizers and its possible role in the pathogenesis of asthma | 1997 |
| Ojo, O. O.,Basu, S.,Jha, A.,Ryu, M.,Schwartz, J.,Doeing, D.,McConville, J.,Vogl, T.,Roth, J.,Halayko, A. J. | S100A8/A9 is a mediator of asthma pathophysiology in an acute allergic model of asthma | 2014 |
| Okazaki, M.,Date, H.,Inokawa, H.,Okutani, D.,Aokage, K.,Nagahiro, I.,Aoe, M.,Sano, Y.,Shimizu, N. | Optimal time for post-mortem heparinization in canine lung transplantation with non-heart-beating donors | 2006 |
| Okazawa, M.,Bai, T. R.,Wiggs, B. R.,Pare, P. D. | Airway smooth muscle shortening in excised canine lung lobes | 1993 |
| Okazawa, M.,Pare, P. D.,Lambert, R. K. | Compliance of peripheral airways deduced from morphometry | 2000 |
| Oktarina, D. A. M.,Sylviningrum, T.,Indrastuti, N. | Colophony as a cause of Riehl melanosis: A case series | 2013 |
| Olaguibel, J. M.,Hernandez, D.,Morales, P.,Peris, A.,Basomba, A. | Occupational asthma caused by inhalation of casein | 1990 |
| Oldenburg, M.,Latza, U.,Baur, X. | Airborne exposure parameters in a cotton spinning mill: Relevance and health effects | 2003 |
| Oldenburg, M.,Latza, U.,Baur, X. | Exposure-response relationship between endotoxin exposure and lung function impairment in cotton textile workers | 2006 |
| Olenchock, S. A.,Christiani, D. C.,Mull, J. C.,Ye, T. T.,Lu, P. L. | Airborne endotoxin concentrations in various work areas within two cotton textile mills in the People's Republic of China | 1990 |
| Oliveira, E. C.,Moura, M. A. G.,Santos, A. M. R.,Oliveira, A. | Treatment of pulmonary giant arteriovenous fistula with cyanocrylate glue a case report | 2012 |
| Olivo, C.,Neves, L.,Lourenc¸o, J.,Almeida, F.,Prado, C.,Tibério, I.,Tanaka, A.,Sasaki, S.,Martins, M.,Lopes, F. | Effects of a protease inhibitor from the tick rhipicephalus boophilus microplus in an experimental model of emphysema | 2011 |
| Olivo, C.,Scarpa, B.,Almeida, F.,Arantes, P.,Lopes, F.,Martins, M. | Time course analysis of lung function and morphometric parameters in a murine model of emphysema | 2011 |
| Olsen, Keith C.,Sapinoro, Ramil E.,Kottmann, R. M.,Kulkarni, Ajit A.,Iismaa, Siiri E.,Johnson, Gail V. W.,Thatcher, Thomas H.,Phipps, Richard P.,Sime, Patricia J. | Transglutaminase 2 and Its Role in Pulmonary Fibrosis | 2011 |
| Olsen, R.,Backman, J.,Molander, P.,Ovrebo, S.,Thorud, S.,Lundanes, E.,Greibrokk, T.,Kronberg, L. | Characterization of adducts formed in the reaction of glutaraldehyde with 2'-deoxyadenosine | 2007 |
| Olson, M. R.,Hartwig, S. M.,Varga, S. M. | The Number of Respiratory Syncytial Virus (RSV)-Specific Memory CD8 T Cells in the Lung to Inhibit RSV Vaccine-Enhanced Is Critical for Their Ability Pulmonary Eosinophilia | 2008 |
| Omae, K.,Higashi, T.,Nakadate, T.,Tsugane, S.,Nakaza, M.,Sakurai, H. | Four-year follow-up of effects of toluene diisocyanate exposure on the respiratory system in polyurethane foam manufacturing workers. II. Four-year changes in the effects on the respiratory system | 1992 |
| Omidbakhsh, N. | A new peroxide-based flexible endoscope-compatible high-level disinfectant | 2006 |
| Ong, K. H.,Lewis, R. D.,Dixit, A.,MacDonald, M.,Yang, M.,Qian, Z. | Inactivation of dust mites, dust mite allergen, and mold from carpet | 2014 |
| Ono, N.,Kusunoki, T.,Ikeda, K. | Correlation between CU/ZN-SOD and macrophages or MUC5AC in eosinophilic chronic rhinosinusitis with nasal polyps | 2012 |
| Ono, N.,Kusunoki, T.,Ikeda, K. | Relationships between IL-17A and macrophages or MUC5AC in eosinophilic chronic rhinosinusitis and proposed pathological significance | 2013 |
| Onodera, Kenji,Sogawa, Norio,Sogawa, Chiharu,Furuta, Hiroaki | The 1999 domestic state of development of anti-asthma | 2000 |
| Openshaw, P. J. | Antiviral immune responses and lung inflammation after respiratory syncytial virus infection | 2005 |
| Openshaw, P. J.,Tregoning, J. S. | Immune responses and disease enhancement during respiratory syncytial virus infection | 2005 |
| Openshaw, P. J. M.,Tregoning, J. S. | Immune responses and disease enhancement during respiratory syncytial virus infection | 2005 |
| Optis, M.,Shaw, K.,Stephenson, P.,Wild, P. | Mold growth in on-reserve homes in Canada: the need for research, education, policy, and funding | 2012 |
| Ordonez, C.,Ferrando, R.,Hyde, D. M.,Wong, H. H.,Fahy, J. V. | Epithelial desquamation in asthma: artifact or pathology? | 2000 |
| Orhan, M.,Ikiz, Z. A.,Saylam, C. Y. | Anatomical features of the opening of the nasolacrimal duct and the lacrimal fold (Hasner's valve) for intranasal surgery: a cadaveric study | 2009 |
| Orlandini, A.,Viotti, G.,Magno, L. | Anaphylactoid reaction induced by patch testing with formaldehyde in an asthmatic | 1988 |
| Orloff, K. G.,Batts-Osborne, D.,Kilgus, T.,Metcalf, S.,Cooper, M. | Antibodies to toluene diisocyanate in an environmentally exposed population | 1998 |
| Orlov, V. A.,Yakunin, G. A.,Grishina, N. B.,Orekhova, N. S. | State of Hemostasis in Various Forms of Pancreatitis | 1981 |
| Ormstad, Heidi | Suspended particulate matter in indoor air: Adjuvants and allergen carriers | 2000 |
| Orr, T. S. C. | Morphology of Human Mast Cell De Granulation and Histamine Release | 1975 |
| Orr, T. S. C.,Hall, D. E.,Gwilliam, J. M.,Cox, J. S. G. | The Effect of Di Sodium Cromoglycate on the Release of Histamine and Degranulation of Rat Mast Cells Induced by Compound 48-80 P Methoxyphenethyl Methylamine Condensation Product with Formaldehyde | 1971 |
| Orrskog, Sofia,Rounioja, Samuli,Spadafina, Tiziana,Gallotta, Marilena,Norman, Martin,Hentrich, Karina,Falker, Stefan,Ygberg-Eriksson, Sofia,Hasenberg, Mike,Johansson, Bjorn,Uotila, Liisa M.,Gahmberg, Carl G.,Barocchi, Michele,Gunzer, Matthias,Normark, Staffan,Henriques-Normark, Birgitta | Pilus Adhesin RrgA Interacts with Complement Receptor 3, Thereby Affecting Macrophage Function and Systemic Pneumococcal Disease | 2013 |
| Orvain, C.,Augusto, J. F.,Marc, G.,Subra, J. F.,Sayegh, J. | Thrombotic microangiopathy with severe renal failure secondary to beta-interferon treatment for multiple sclerosis | 2012 |
| Osborne, M. L.,Sommerhoff, C. P.,Nadel, J. A.,McDonald, D. M. | Histochemical comparison of mast cells obtained from the airways of mongrel dogs and Basenji-Greyhound dogs by bronchoalveolar lavage | 1989 |
| Osiewacz, H. D. | Mitochondrial functions and aging | 2002 |
| Osiewacz, H. D.,Borghouts, C. | Cellular copper homeostasis, mitochondrial DNA instabilities, and lifespan control in the filamentous fungus Podospora anserina | 2000 |
| Osternig, L. R.,Ferber, R.,Mercer, J.,Davis, H. | Human hip and knee torque accommodations to anterior cruciate ligament dysfunction | 2000 |
| Ostojic, V.,Cvoriscec, B.,Ostojic, S. B.,Reznikoff, D.,Stipic-Markovic, A.,Tudjman, Z. | Improving asthma control through telemedicine: A study of short-message service | 2005 |
| Otsuka, Hirokuni,Ohkubo, Kimihiro,Seki, Harumi,Ohnishi, Masaki,Fujikura, Terumichi | Mast cell quantitation in nasal polyps, sinus mucosa and nasal turbinate mucosa | 1993 |
| Ott, M. G.,Diller, W. F.,Jolly, A. T. | Respiratory effects of toluene diisocyanate in the workplace: a discussion of exposure-response relationships | 2003 |
| Ouyang, N.,Ding, J.,Chen, S. | [Effects of dexamethasone on apoptosis of airway inflammatory cells in asthmatic guinea-pigs] | 2001 |
| Overton, J. H.,Kimbell, J. S.,Miller, F. J. | Dosimetry modeling of inhaled formaldehyde: the human respiratory tract | 2001 |
| Owens, G. U. Y.,Adams, Jesse E.,Scott, H. W. | Embolic fat as a measure of adequacy of various oxygenators | 1960 |
| Ozen, Oguz Aslan,Songur, Ahmet,Sarsilmaz, Mustafa,Yaman, Mehmet,Kus, Ilter | Changes of zinc, copper, and iron levels in the lung of male rats after subacute (4-week) and subchronic (13-week) exposure to formaldehyde | 2003 |
| Ozen, Z.,Mumbuc, S.,Sari, I.,Baglam, T.,Karatas, E.,Kanlikama, M. | Cysteinyl leukotriene receptor expression in aspirin-sensitive nasal polyposis patients | 2007 |
| Oziganova, V. N. | Classification of occupational bronchial asthma of chemical aetiology | 1984 |
| Ozkaya, E.,Sogut, A.,Kucukkoc, M.,Eres, M.,Acemoglu, H.,Yuksel, H.,Murat, N. | Sensitization pattern of inhalant allergens in children with asthma who are living different altitudes in Turkey | 2015 |
| Pabst, R. | Exposure to formaldehyde in anatomy: an occupational health hazard? | 1987 |
| Pacheco, K. A. | Allergy to Surgical Implants | 2015 |
| Pacheco, K. A.,Mayer, A.,Erb, S.,Shirname-More, L.,Maier, L. A. | High rates of sensitization to selected metals and bone cement in joint replacement failure patients and preoperative evaluations | 2014 |
| Pacioni, Giovanni | The role of sulphur in truffle metabolism | 1992 |
| Padhi, B. K.,Padhy, P. K. | Domestic fuels, indoor air pollution, and children's health | 2008 |
| Pagaria, S.,Singh, B. D.,Dubey, A.,Avinash, A. | Molar incisor hypomineralisation: A review | 2015 |
| Paggiaro, P. L.,Bancalari, L.,Cianchetti, S.,Bartoli, M. L.,Carletti, A.,Macchioni, P.,Petrozzino, M.,Taccola, M.,Giuntini, C. | Markers of airway inflammation in induced sputum of subjects with RADS | 1995 |
| Paggiaro, P. L.,Cantalupi, R.,Filieri, M.,Loi, A. M.,Parlanti, A.,Toma, G.,Baschieri, L. | Bronchial asthma due to inhaled wood dust: Tanganyika aningre | 1981 |
| Paggiaro, P. L.,Rossi, O.,Lastrucci, L.,Pardi, F.,Pezzini, A.,Baschieri, L. | TDI-induced oculorhinitis and bronchial asthma | 1985 |
| Paine, M. D.,Balon, E. K. | Early Development of the Northern Logperch Percina-Caprodes-Semifasciata According to the Theory of Saltatory Ontogeny | 1984 |
| Pal, T. M.,Groothoff, J. W.,Post, D.,de Monchy, J. G. | Follow-up study of workers in a nylon carpet yarn plant after remedial actions taken against a contaminated humidification system | 2000 |
| Palatianos, G. M.,Paziouros, K.,Vassili, M. I.,Stratigi, P.,Kaklamanisj, L.,Prapas, S.,Panagiotou, M. S.,Iliopoulou, E.,Mardaki, S.,Melissari, E. N. | Effect of exogenous nitric oxide during cardiopulmonary bypass on lung postperfusion histology | 2005 |
| Pallis, F. R.,Conran, N.,Fertrin, K. Y.,Olalla Saad, S. T.,Costa, F. F.,Franco-Penteado, C. F. | Hydroxycarbamide reduces eosinophil adhesion and degranulation in sickle cell anaemia patients | 2014 |
| Pallis, F. R.,Conran, N.,Fertrin, K. Y.,Olalla-Saad, S. T.,Costa, F. F.,Franco-Penteado, C. F. | Altered functional properties of eosinophils in sickle cell anemia and effects of hydroxyurea therapy | 2010 |
| Palombini, L.,Pelayo, R.,Guilleminault, C. | Efficacy of automated continuous positive airway pressure in children with sleep-related breathing disorders in an attended setting | 2004 |
| Pan, Jie,Yeger, Herman,Ratcliffe, Peter,Bishop, Tammie,Cutz, Ernest | Hyperplasia of Pulmonary Neuroepithelial Bodies (NEB) in Lungs of Prolyl Hydroxylase-1(PHD-1) Deficient Mice | 2012 |
| Panda, Sagar Suman,Kumar, Bera VenkataVaraha Ravi,Mohanta, Ganeswar | Stability-indicating RP-HPLC method for simultaneous estimation of levosalbutamol sulfate and theophylline in combined dosage form | 2013 |
| Pandya, C. M.,Ansari, S.,Dalal, B.,Adam, A.,Dogra, S. | A novel use of fibrin sealant glue as a treatment for massive hemoptysis | 2009 |
| Pang, Hong-yu,Cheng, Nan,Chi, Xin-jin,Hei, Zi-qing,Ge, Mian | Effects of in vitro administration of propofol on neutrophils function in healthy volunteers | 2009 |
| Panos, R. J.,Patel, R.,Bak, P. M. | Intratracheal administration of hepatocyte growth factor scatter factor stimulates rat alveolar type II cell proliferation in vivo | 1996 |
| Paranaíba, L. M. R.,Fernandes, C. M.,de Aquino, S. N.,dos Santos, L. A. N.,Martelli-Júnior, H. | Ellis-van Creveld syndrome: Systemic and oral findings | 2012 |
| Paraskevas, G. K.,Raikos, A.,Chouliaras, K.,Papaziogas, B. | Variable anatomical relationship of phrenic nerve and subclavian vein: clinical implication for subclavian vein catheterization | 2011 |
| Pariselli, F.,Sacco, M. G.,Rembges, D. | An optimized method for in vitro exposure of human derived lung cells to volatile chemicals | 2008 |
| Park, Chen Hee,Lee, Hyung Gon,Lee, Seong Heon,Chung, Cheol Won,Yoon, Myung Ha | The role of adrenergic and cholinergic receptors on the antinociception of sildenafil in the spinal cord of rats | 2011 |
| Park, Hye-Kyung,Jeon, Seong-Gyu,Kim, Tae-Bum,Kang, Hye-Ryun,Chang, Yoon-Seok,Kim, Yoon-Keun,Cho, Sang-Heon,Min, Kyung-Up,Kim, You-Young | Occupational asthma and rhinitis induced by a herbal medicine, Wonji (Polygala tenuifolia) | 2005 |
| Park, Jin-Ah,Kim, Jae Hun,Bi, Dapeng,Mitchel, Jennifer A.,Qazvini, Nader Taheri,Tantisira, Kelan,Park, Chan Young,McGill, Maureen,Kim, Sae-Hoon,Gweon, Bomi,Notbohm, Jacob,Steward, Robert, Jr.,Burger, Stephanie,Randell, Scott H.,Kho, Alvin T.,Tambe, Dhananjay T.,Hardin, Corey,Shore, Stephanie A.,Israel, Elliot,Weitz, David A.,Tschumperlin, Daniel J.,Henske, Elizabeth P.,Weiss, Scott T.,Manning, M. Lisa,Butler, James P.,Drazen, Jeffrey M.,Fredberg, Jeffrey J. | Unjamming and cell shape in the asthmatic airway epithelium | 2015 |
| Park, J. A.,Kim, J. H.,Bi, D. P.,Mitchel, J. A.,Qazvini, N. T.,Tantisira, K.,Park, C. Y.,McGill, M.,Kim, S. H.,Gweon, B.,Notbohm, J.,Steward, R.,Burger, S.,Randell, S. H.,Kho, A. T.,Tambe, D. T.,Hardin, C.,Shore, S. A.,Israel, E.,Weitz, D. A.,Tschumperlin, D. J.,Henske, E. P.,Weiss, S. T.,Manning, M. L.,Butler, J. P.,Drazen, J. M.,Fredberg, J. J. | Unjamming and cell shape in the asthmatic airway epithelium | 2015 |
| Park, J. H.,Spengler, J. D.,Yoon, D. W.,Dumyahn, T.,Lee, K.,Ozkaynak, H. | Measurement of air exchange rate of stationary vehicles and estimation of in-vehicle exposure | 1998 |
| Park, J. H.,Spiegelman, D. L.,Burge, H. A.,Gold, D. R.,Chew, G. L.,Milton, D. K. | Longitudinal study of dust and airborne endotoxin in the home | 2000 |
| Park, J. H.,Spiegelman, D. L.,Gold, D. R.,Burge, H. A.,Milton, D. K. | Predictors of airborne endotoxin in the home | 2001 |
| Park, J. H.,Szponar, B.,Larsson, L.,Gold, D. R.,Milton, D. K. | Characterization of lipopolysaccharides present in settled house dust | 2004 |
| Park, J. W.,Kim, C. W.,Kang, D. B.,Lee, I. Y.,Choi, S. Y.,Yong, T. S.,Shin, D. C.,Kim, K. E.,Hong, C. S. | Low flow, long-term air sampling under normal domestic activity to measure house dust mite and cockroach allergens | 2002 |
| Park, Y. S.,Park, C. M.,Lee, H. J.,Goo, J. M.,Chung, D. H.,Lee, S. M.,Yim, J. J.,Kim, Y. W.,Han, S. K.,Yoo, C. G. | Clinical implication of protease-activated receptor-2 in idiopathic pulmonary fibrosis | 2012 |
| Parker, E. A.,Chung, L. K.,Israel, B. A.,Reyes, A.,Wilkins, D. | Community Organizing Network for Environmental Health: Using a Community Health Development Approach to Increase Community Capacity around Reduction of Environmental Triggers | 2010 |
| Parker, M. J.,Gurusamy, K.,Stoker, M. | (i) Surgery in elderly patients | 2004 |
| Parkos, C. A. | Molecular events in neutrophil transepithelial migration | 1997 |
| Parra, F. M.,Igea, J. M.,Quirce, S.,Ferrando, M. C.,Martin, J. A.,Losada, E. | Occupational asthma in a hairdresser caused by persulphate salts | 1992 |
| Parsons, J. F.,Goodwin, B. F. J.,Safford, R. J. | Studies on the action of histamine release by persulphates | 1979 |
| Parsons, J. F.,Goodwin, B. F. J.,Safford, R. J. | Studies on the Action of Histamine Release by Per Sulfates | 1979 |
| Parvaneh, S.,Kronqvist, M.,Johansson, E.,van Hage-Hamsten, M. | Exposure to an abundance of cat (Fel d 1) and dog (Can f 1) allergens in Swedish farming households | 1999 |
| Pasaoglu Karakis, G.,Birben, E.,Bozdogan, G.,Sackesen, C.,Kalayci, O. | Evaluation of the CD14-159 C/T polymorphism and endotoxin exposure in cotton textile workers: Geneenvironment interaction | 2011 |
| Pastuszka, J. S.,Paw, U. K. T.,Lis, D. O.,Wlazlo, A.,Ulfig, K. | Bacterial and fungal aerosol in indoor environment in Upper Silesia, Poland | 2000 |
| Pataki, A.,Madarasz, E.,Kurucz, I. | Fibronectin quantification without antibodies: A bioassay for the detection of the gelatin-captured macromolecule | 2006 |
| Patchett, K.,Lewis, S.,Crane, J.,Fitzharris, P. | Cat allergen (Fel d 1) levels on school children's clothing and in primary school classrooms in Wellington, New Zealand | 1998 |
| Patelarou, E.,Tzanakis, N.,Kelly, F. J. | Exposure to Indoor Pollutants and Wheeze and Asthma Development during Early Childhood | 2015 |
| Patsantara, G.,Piperaki, E. T.,Tzoumaka-Bakoula, C.,Kanariou, M. | P29-Intestinal helminth Enterobius vermicularis as an immunomodulator factor | 2014 |
| Pattanayak, S. P.,Mazumder, P. M.,Sunita, S. | Preliminary studies on antinociceptive activity of Dendrophthoe falcata (L.F) Ettingsh (Loranthaceae) | 2008 |
| Patterson, R.,Grammer, L. C.,Zeiss, C. R.,Harris, K. E.,Shaughnessy, M. A. | USE OF IMMUNOLOGIC TECHNOLOGY IN THE DIAGNOSIS OF ENVIRONMENTAL AND OCCUPATIONAL IMMUNOLOGIC LUNG DISEASE | 1990 |
| Patterson, R.,Pateras, V.,Grammer, L. C.,Harris, K. E. | Human Antibodies against Formaldehyde-Human Serum Albumin Conjugates or Human Serum Albumin in Individuals Exposed to Formaldehyde | 1985 |
| Patterson, R.,Pateras, V.,Grammer, L. C.,Harris, K. E. | Human antibodies against formaldehyde-human serum albumin conjugates or human serum albumin in individuals exposed to formaldehyde | 1986 |
| Paufler, P.,Gebel, T.,Dunkelberg, H. | Quantification of house dust mite allergens in ambient air | 2001 |
| Paulo, M.,De Farias, M. R.,Nogueira, F.,Barbosa, M.,Arruda, L. K.,Filho, N. R. | Influence of gender and sexual reproductive state in concentration of CAN f 1 in the fur of dogs (Canis lupus familiaris) | 2015 |
| Pauls, S. R.,Jottini, S.,Takai, S.,Venner, M.,Wohlsein, P. | Immunohistological demonstration of Rhodococcus equi in a trotter foal | 2009 |
| Pauluhn, J. | Risk assessment of pyrethroids following indoor use | 1996 |
| Paustenbach, D.,Alarie, Y.,Kulle, T.,Schachter, N.,Smith, R.,Swenberg, J.,Witschi, H.,Horowitz, S. B. | A recommended occupational exposure limit for formaldehyde based on irritation | 1997 |
| Paustenbach, D.,Burke, M. L.,Shum, M.,Kalmes, R. | Airborne concentrations of ethyl and methyl cyanoacrylate in the workplace | 2001 |
| Paveglio, S. A.,Allard, J.,Foster Hodgkins, S. R.,Ather, J. L.,Bevelander, M.,Campbell, J. M.,Whittaker LeClair, L. A.,McCarthy, S. M.,van der Vliet, A.,Suratt, B. T.,Boyson, J. E.,Uematsu, S.,Akira, S.,Poynter, M. E. | Airway epithelial indoleamine 2,3-dioxygenase inhibits CD4+ T cells during Aspergillus fumigatus antigen exposure | 2010 |
| Paveglio, S. A.,Allard, J.,Hodgkins, S. R. F.,Ather, J. L.,Bevelander, M.,Campbell, J. M.,LeClair, L. A. W.,McCarthy, S. M.,van der Vliet, A.,Suratt, B. T.,Boyson, J. E.,Uematsu, S.,Akira, S.,Poynter, M. E. | Airway Epithelial Indoleamine 2,3-Dioxygenase Inhibits CD4(+) T Cells during Aspergillus fumigatus Antigen Exposure | 2011 |
| Pavlenko, S. M.,Yudina, T. V.,Guseva, V. A. | Methodological Approaches to an Evaluation of Latent Reactions of Certain Regulatory Systems of the Body in the Case of Different Ways of Intake of Toxic Substances | 1975 |
| Pearson, William G., Jr.,Langmore, Susan E.,Yu, Lou B.,Zumwalt, Ann C. | Muscles Underlying the Elevation of the Hyolaryngeal Complex | 2011 |
| Pechlivanidis, T.,Sichletidis, L. | The clinical status of asthmatic patients allergic to house dust mites-changes after 1 year of specific immunotherapy | 2009 |
| Peden, D.,Reed, C. E. | Environmental and occupational allergies | 2010 |
| Peebles, R. S.,Hashimoto, K.,Collins, R. D.,Jarzecka, K.,Furlong, J.,Mitchell, D. B.,Sheller, J. R.,Graham, B. S. | Immune interaction between respiratory syncytial virus infection and allergen sensitization critically depends on timing of challenges | 2001 |
| Peebles, R. S., Jr.,Sheller, J. R.,Collins, R. D.,Jarzecka, K.,Mitchell, D. B.,Graham, B. S. | Respiratory syncytial virus (RSV)-induced airway hyperresponsiveness in allergically sensitized mice is inhibited by live RSV and exacerbated by formalin-inactivated RSV | 2000 |
| Peerless, S. J.,Nakamura, R.,Rodriguez-Salazar, A.,Hunter, I. G. | Modification of cerebral ischemia with Fluosol | 1985 |
| Peeters, D.,Day, M. J.,Clercx, C. | Distribution of leucocyte subsets in bronchial mucosa from dogs with eosinophilic bronchopneumopathy | 2005 |
| Pekkanen, J.,Hyvarinen, A.,Haverinen-Shaughnessy, U.,Korppi, M.,Putus, T.,Nevalainen, A. | Moisture damage and childhood asthma: a population-based incident case-control study | 2006 |
| Peled, R.,Friger, M.,Bolotin, A.,Bibi, H.,Epstein, L.,Pilpel, D.,Scharf, S. | Fine particles and meteorological conditions are associated with lung function in children with asthma living near two power plants | 2005 |
| Pennanen, S.,Mussalo-Rauhamaa, H.,Harju, A.,Pippuri, M.,Liesivuori, J.,Elg, P.,Hakala, K.,Haahtela, T. | Exposure to mites, sensitisation and allergy to mites in moisture damaged buildings | 2007 |
| Pereira, G.,Cook, A. G.,Haggar, F.,Bower, C.,Nassar, N. | Locally derived traffic-related air pollution and fetal growth restriction: a retrospective cohort study | 2012 |
| Pereira, H. Anne | Cationic antimicrobial protein of Mr 37 kDa: A multifunctional inflammatory protein | 2001 |
[truncated: 401,946 more chars]
