# Supplementary material for: Exposure to formaldehyde and asthma outcomes: A systematic review, meta-analysis, and economic assessment
Source: PLoS One. 2021 Mar 31;16(3):e0248258. doi: 10.1371/journal.pone.0248258 (PMC8011796; doi:10.1371/journal.pone.0248258)
Supplement: S1 References — (DOCX) [file pone.0248258.s121.docx]

**REFERENCES**

1. Morgan WJ, Stern DA, Sherrill DL, Guerra S, Holberg CJ, Guilbert TW, et al. Outcome of Asthma and Wheezing in the First 6 Years of Life. American Journal of Respiratory and Critical Care Medicine. 2005;172(10):1253-8.

2. Moya J, Bearer CF, Etzel RA. Children’s behavior and physiology and how it affects exposure to environmental contaminants. Pediatrics. 2004;113(Supplement 3):996-1006.

3. Bolte G, Heitmann D, Kiranoglu M, Schierl R, Diemer J, W. K, et al. Exposure to environmental tobacco smoke in German restaurants, pubs and discotheques. Journal of Exposure Science and Environmental Epidemiology. 2008;18(3):262-71.

4. Word Health Organization. Tobacco smoke and involuntary smoking. Lyon, France; 2004.

5. Ferrante G, Antona R, Malizia V, Montalbano L, Corsello G, La Grutta S. Smoke exposure as a risk factor for asthma in childhood: A review of current evidence. Allergy and Asthma Proceedings. 2014;35(6):454-61.

6. Hun DE, Siegel JA, Morandi MT, Stock TH, Corsi RL. Cancer risk disparities between Hispanic and non-Hispanic white populations: the role of exposure to indoor air pollution. Environmental health perspectives. 2009;117(12):1925-31.

7. Bhan N, Kawachi I, Glymour MM, Subramanian SV. Time Trends in Racial and Ethnic Disparities in Asthma Prevalence in the United States From the Behavioral Risk Factor Surveillance System (BRFSS) Study (1999–2011) American Journal of Public Health. 2015;105(6):1269-75.

8. Bjornson CL, Mitchell I. Gender differences in asthma in childhood and adolescence. The journal of gender-specific medicine: JGSM: the official journal of the Partnership for Women's Health at Columbia. 1999;3(8):57-61.

9. Brüske I, Flexeder C, Heinrich J. Body mass index and the incidence of asthma in children. Current opinion in allergy and clinical immunology. 2014;14(2):155-60.

10. Langley SJ, Goldthorpe S, Craven M, Morris J, Woodcock A, Custovic A. Exposure and sensitization to indoor allergens: association with lung function, bronchial reactivity, and exhaled nitric oxide measures in asthma. Journal of Allergy and Clinical Immunology. 2003;112(2):362-8.

11. Sears MR, Johnston NW. Understanding the September asthma epidemic. Journal of Allergy and Clinical Immunology. 2007;120(3):526-9.

12. U.S. Consumer Product Safety Commission. An Update on Formaldehyde. Bethesda, MD: US CPSC; 2015. Contract No.: Publication 725.

13. California Environmental Protection Agency. Final Report on the Identification of Formaldehyde as a Toxic Air Contaminant. In: Assessment OoEHH, editor. Oakland, CA1992.

14. Hospital JH, Engorn B, Flerlage J. The Harriet Lane Handbook. 20 ed. Baltimore, MD: Saunders, an imprint of Elsevier Inc.; 2015.

15. Beamer PI, Luik CE, Canales RA, Leckie JO. Quantified outdoor micro-activity data for children aged 7–12-years old. Journal of Exposure Science and Environmental Epidemiology. 2012;22(1):82-92.

16. Guyatt GH, Oxman AD, Montori V, Vist G, Kunz R, Brozek J, et al. GRADE guidelines: 5. Rating the quality of evidence-publication bias. J Clin Epidemiol. 2011;64(12):1277–82.

17. Guyatt GH, Oxman AD, Kunz R, Woodcock J, Brozek J, Helfand M, et al. GRADE guidelines: 8. Rating the quality of evidence-indirectness. J Clin Epidemiol. 2011;64(12):1303-10.

18. Guyatt GH, Oxman AD, Kunz R, Woodcock J, Brozek J, Helfand M, et al. GRADE guidelines: 7. Rating the quality of evidence-inconsistency. J Clin Epidemiol. 2011;64(12):1294-302.

19. Guyatt G, Oxman A, Kunz R, Brozek J, Alonso-Coello P, Rind D, et al. GRADE guidelines: 6. Rating the quality of evidence--imprecision. J Clin Epidemiol. 2011;64(12):1283-93.

20. Higgins JPT, Green S. Cochrane Handbook for Systematic Reviews of Interventions. Version 5.1.0 [Updated March 2011]: The Cochrane Collaboration. Available from [www.cochrane-handbook.org](file:///F:\UCSF\Navigating%20the%20Sciences\JPB%20formaldehyde\Manuscript%20draft\www.cochrane-handbook.org).; 2011. Available from: Available from [www.cochrane-handbook.org](file:///F:\UCSF\Navigating%20the%20Sciences\JPB%20formaldehyde\Manuscript%20draft\www.cochrane-handbook.org).

21. Guyatt GH, Oxman AD, Sultan S, Glasziou P, Akl EA, Alonso-Coello P, et al. GRADE guidelines: 9. Rating up the quality of evidence. J Clin Epidemiol. 2011;64(12):1311–6.

22. National Toxicology Program. Handbook for conducting a literature-based health assessment using OHAT approach for systematic review and evidence integration 2015 [Available from: <http://ntp.niehs.nih.gov/ntp/ohat/pubs/handbookjan2015_508.pdf>.
