# Supplementary material for: Exposure to formaldehyde and asthma outcomes: A systematic review, meta-analysis, and economic assessment
Source: PLoS One. 2021 Mar 31;16(3):e0248258. doi: 10.1371/journal.pone.0248258 (PMC8011796; doi:10.1371/journal.pone.0248258)
Supplement: Attachment — Submitted filename: PLOS review Lan et al. formaldehyde and asthma Reivew .pdf [file pone.0248258.s122.pdf]

Exposure to Formaldehyde and Asthma Outcomes: A Systematic Review, Meta-Analysis, and Economic Assessment

I applaud the authors for identifying a public health problem (e.g., EPA proposed formaldehyde rule does not consider asthma as a health outcome) and conducting a comprehensive approach to improve public health. This approach consisted of a series of systematic reviews and associated meta-analyses, and an economic assessment on exposure to formaldehyde and asthma. I agree that excluding established health effects from the economic analysis can underestimate the benefits of regulations. However, the quality of the health hazard assessment is not adequate or transparent to support the level of evidence conclusions, and thus, opens the door for major criticism and compromises potential public impacts from the economic assessment.

Increase the readability of the four systematic reviews
The manuscript reports on four systematic reviews or groups: two different populations (adults and children) and two asthma outcomes (asthma and asthma exacerbation). The assessment of the evidence is difficult to follow because some results (e.g., description of the studies and risk of bias assessments) are not discussed separately for each systematic review. The authors are encouraged to organize the entire result section into four systematic reviews. Each subsection would discuss the study characteristics, risk of bias assessment, and synthesis of the findings across studies. Table 3 should be divided into four separate tables, one for each group. Citations are often missing in the results and discussion section.

Strengthen the rigor of the systematic reviews

The major limitation of the systematic review is the lack of a rigorous, defendable assessment that supports the strength of evidence conclusions. I feel that the report is inadequate to evaluate whether I agree or disagree with the authors' conclusions. For each assessment, the authors concluded there was sufficient evidence supporting an association between exposure to formaldehyde and asthma diagnosis or symptoms. Sufficient evidence means that chance, bias, and confounding can be ruled out with reasonable confidence. The risk of bias analysis found that many studies with a high or probably high risk of bias (especially for confounding) are included in the assessment. The authors state that they did not believe the bias warrant downgrading; however, they do not provide any rationale to support this assumption. Risk of bias is the potential for bias and does not necessarily mean that the findings biases. To support a conclusion of sufficient evidence, a detailed assessment of the direction, magnitude, and impact of the bias on the findings is needed. The authors should provide a more comprehensive discussion of biases and specific confounders (including co-exposures) across studies.

Another limitation of the assessment is an incomplete discussion of the findings across studies. The conclusions for the children's studies are largely based on the meta-analyses of a small subset of the available studies. The synthesis of the evidence from the studies not included in the meta-analysis and the synthesis of the studies of adults is inadequate and hard to follow as many of the results are providing in supplementary tables and it not clear if all the results are discussed. The authors allude to the fact that some studies did not provide data and only state study author conclusions. These studies should probably not be included in the systematic review. I recognized that many of the studies have different metrics (e.g., OR,

exposure level, asthma prevalence); however, the synthesis and discussion of findings could be improved. For example, perhaps a pooled weighted mean difference in formaldehyde exposure levels could be calculated for studies reporting that type of data.

For the two assessments of childhood exposure, the overall conclusions are largely based on the meta-analyses of a small subset of the available studies. The Hulin study contributes 89% of the weight of the risk estimate for the assessment of childhood exposure to formaldehyde and asthma diagnosis. Moreover, there was considerable internal heterogeneity in this study, i.e., positive associations were found for children in rural but not urban areas. Thus, a careful evaluation of all the evidence is needed to ensure the conclusions are not driven by a subgroup analysis from one study. All of the evidence should be evaluated as a whole before conducting a meta-analysis on a subset of studies. The authors should also assess key issues such as the source of exposure or study design.

Confidence levels
It is not clear how useful the GRADE approach is for reaching the strength of the evidence conclusions. For each of the four assessments, the initial rating of confidence was not upgraded or downgraded based on the eight GRADE factors (e.g., a total of 32 opportunities across the four assessments. As the authors conduct a risk of bias assessment, and the evidence can be downgraded based on that assessment, it is not clear why the initial rating of the initial level of evidence is rated as moderate. It is not clear why dose-response was not upgraded for some of the reviews. The meta-analyses appear to be based on assuming a dose-response relationship to convert to a common unit. the economic impact models a dose-response, Lastly, three of the adult studies on asthma diagnosis had a positive trend.

Literature review

Please update the literature search to identify studies published after March 15, 2018, which should be included in the health assessments and meta-analyses. The meta-risk estimates, which are based on small numbers of studies, are susceptible to the findings from new studies.

Other comments
Introduction: The detailed criticism of the EPA review (lines 79 to 90) may be unnecessary and should be shortened. Was asthma removed from the economic analysis because EPA concluded there was limited evidence for exposure to formaldehyde and asthma or because there were concerns about a higher level of evidence conclusion? A limited conclusion would be adequate justification for this systematic review. If EPA concluded there was sufficient evidence, you could briefly mention (one sentence) that NAS expressed some concerns about their assessment.

Acknowledgments: It is unclear why reviewers who conducted risk of bias analysis and statistical analysis are not authors of the manuscript. These skills and scientific input seem to be critical for the conclusions.

Minor or formatting comments

- Table 3 is unreadable in PDF as it should probably be landscaped (e.g., not all the columns are appearing on the same page and are repeated at the end of the study. Separate tables should be made for each assessment, which should also include the results of each study.

- Supplementary methods 5 appears to be missing.
- Figure 3 is not useful and could be deleted.
- The studies in Figure 7 are not labeled.
- The font in the supplementary figure are small and hard to read
