# Supplementary material for: Exposure to formaldehyde and asthma outcomes: A systematic review, meta-analysis, and economic assessment
Source: PLoS One. 2021 Mar 31;16(3):e0248258. doi: 10.1371/journal.pone.0248258 (PMC8011796; doi:10.1371/journal.pone.0248258)
Supplement: Attachment — Submitted filename: PLOS review Lan et al. formaldehyde and asthma ReivewRevision.pdf [file pone.0248258.s124.pdf]

Exposure to Formaldehyde and Asthma Outcomes: A Systematic Review, Meta-Analysis, and Economic Assessment: Revision

I appreciate the authors' efforts to improve the transparency and strengthen the scientific rigor of the systematic review. However, the revised manuscript is still not adequate to determine whether the body of evidence supports the authors' conclusions using their systematic review method.

Increase the readability of the four systematic reviews

The manuscript reports on four systematic reviews or groups: two different populations (adults and children) and two asthma outcomes (asthma and asthma exacerbation). The assessment of the evidence is difficult to follow because the evidence (e.g., description of the studies and risk of bias assessments) are not discussed separately for each systematic review. The authors' approach of trying to clarify information for the four different assessments within each topic (e.g., study characteristics, outcome) only partly addressed the problem. In order for a reader to determine whether a positive relationship is observed between exposure and outcome where chance, bias, and confounding can be ruled out, for each assessment, the reader needs to be to compare the risk of bias assessment and the findings across studies. However, the study findings are reported in different parts of the manuscript (e.g., forest plots for the meta-analysis, supplemental figures), making it hard to evaluate the collective body of evidence for each assessment. Recommendation for fixing the problem include

- Divide Tables 3 and 4 into separate tables for each assessment. Table 4 is unreadable because the font is too small.
- Include results (including subgroup results) and covariates or each study in Table 3. The covariates will help increase transparency for evaluating potential confounding. Including subgroup analyses will help to ensure findings are robust and evaluate important scientific issues or potential effect modifiers.
- Discuss the risk of bias across studies separately for each assessment as there are different issues for each body of literature.

Recommendations for Table 4

- Improve the organization and formatting, e.g., combine similar information, such as population characteristics, in the same column.
- Ensure that the relevant information is extracted from each study. For example, Smedje and Norback, 2001 entry should mention that the cases are incidence, the follow-up is four years, and clarify that 1995 prevalence cases are not included in the analyses.

The advantages of providing transparency outweigh any disadvantages of repeating information. The supplementary figures are not in a format that is adequate to evaluate (small font)

Strengthen the rigor of the systematic reviews

The authors have made some improvements in providing a little more depth in their assessment; however, the manuscript still lacks the necessary depth to support its conclusions.

- Risk of Bias discussion across studies: Some of the discussion seems to be cherry-picking a few examples rather than a concise systematic discussion of the database. The authors have greater confidence in exposure measurements than other types of exposure assessment methods. However, formaldehyde measurements may result in exposure misclassification or mismeasurements, especially if exposure is measured only once and at one location, e.g., studies measuring formaldehyde at school during would miss formaldehyde exposure at home and from other sources and exposure could change over time. The authors state that many occupational studies using a JEM were considered to have a high risk of bias for exposure assessment. Occupational exposure to formaldehyde is probably higher than exposure from other sources. If so, JEM may be adequate for distinguishing exposure groups.
- Direction/impact of biases: The authors state that the reviewers did not feel there was adequate information to evaluate the direction and the impact of bias. Although the direction of bias cannot always be known, this type of analysis is standard epidemiological practice. For example, are the exposure biases, differential, non-differential: Berkson, Classical? If so, they give clues on the direction of the bias. The authors state there is a healthy worker effect, which is a bias towards the null, in some occupational studies.
- Confounding: Although the authors expanded on the discussion on confounding the assessment is still inadequate; e.g., the authors state that the meta-analysis included some studies with low risk of bias but do not discuss whether the better studies have positive findings or contribute the most weight) As per my earlier comments, the authors should provide a more comprehensive discussion of specific confounders (including co-exposures) across studies.
- Other issues: The authors should identify key scientific issues and systematically evaluate them across studies. The evaluation of these issues (such as indoor/outdoor, cross-sectional design) does not need to be limited to studies included in the meta-analysis.
- GRADE confidence ratings for exposure-response rationales lack transparency or scientific rigor as they refer to the reviewers' opinion without criteria: (!1) Data supported the existence of a dose-response relationship, but review authors did not feel it was strong enough to warrant an upgrade for this domain.” (2) Results from the meta-analysis, which assumes a linear dose-response relationship, appeared to support the existence of an association of increasing response with increased dose. However, there was limited data to statistically evaluate whether there was a dose-response relationship specifically for children. Review authors did not believe that results from the meta-analysis were sufficient to warrant upgrading the body of evidence for evidence of a dose-response relationship.” It is not clear what specifically to children means as the studies are of childhood asthma.
