# Supplementary material for: Exposure to formaldehyde and asthma outcomes: A systematic review, meta-analysis, and economic assessment
Source: PLoS One. 2021 Mar 31;16(3):e0248258. doi: 10.1371/journal.pone.0248258 (PMC8011796; doi:10.1371/journal.pone.0248258)
Supplement: Attachment — Submitted filename: 2021 02 18 PRHE Response to Reviewers.pdf [file pone.0248258.s127.pdf]

UCSF Program on Reproductive Health and the Environment

Department of Obstetrics, Gynecology & Reproductive Sciences
Box 0132
490 Illinois Street, Floor 10
San Francisco, CA 94143

Professor Tracey J. Woodruff, PhD, MPH
Director
prhe@ucsf.edu

February 18, 2021

Dear Dr. Amegah,

Thank you for your recent message and additional edits regarding our manuscript appeal to PLOS ONE. Below we have responded to your comment.

General Comment 1: In order to improve the readability of the article, please move tables 3a and 3b to the supplementary material. In addition, please consider dividing table 3b by study design.

We have moved both tables as requested to the supplement and have updated the table order in the manuscript. The new manuscript is down to ~70 pages (clean) and we thank the editorial team for making this suggestion to improve the readability of our manuscript.

Table 3a: We have moved this to the supplement, and it is now the new Supplemental Table 99. We have made the old Supplemental Table 99 ("Study characteristics by population/outcome") into Supplemental Table 100.

Table 3b: We've moved Table 3b to the supplement and split it up by study design (cohort, case-control, cross-sectional, non-randomized controlled trial, randomized controlled trial, case report) as requested. We have further stratified each table by studies considered for meta-analysis and studies not considered for meta-analysis, similar to (new) Supplemental Table 100. Table 3b is now Supplemental Table 101 ("Study characteristics by study design").

We appreciate your time and patience throughout this process. Let us know if you have any additional questions.

Thank you,

Dr. Tracey Woodruff
