# Supplementary material for: Exposure to formaldehyde and asthma outcomes: A systematic review, meta-analysis, and economic assessment
Source: PLoS One. 2021 Mar 31;16(3):e0248258. doi: 10.1371/journal.pone.0248258 (PMC8011796; doi:10.1371/journal.pone.0248258)
Supplement: Attachment — Submitted filename: PONE-D-19-21348R3 Response to reviewers.docx [file pone.0248258.s126.docx]

**Comments from Reviewer 2 and our Response – Peer review Round 3**

The revised manuscript remains difficult to review primarily due to poor organization and formatting of tables. As stated in my previous comments (on the original and R1 version), ideally, the manuscript should have been divided into four separate systematic reviews, with each review discussing the study characteristics, risk of bias, and findings, and including summary tables and figures.

Following two rounds of peer-review comments, we took the advice of the reviewers and editor and each section in the Results section of the manuscript has been divided into four systematic reviews by population/outcome (i.e., child asthma diagnosis, child asthma symptoms, adult asthma diagnosis, adult asthma symptoms). Although not every section was specifically divided into subsections for each population/outcome, each section does include a separate discussion for each population/outcome category. For instance, the study characteristics, exposure, and outcome results sections include separate discussion for each population/outcome category. Other sections such as the Risk of Bias, Statistical Analysis, and the rating of quality/strength of evidence are separated into four different sections by population/outcome categories. Furthermore, in response to this comment specifically we have created Table 99 in the Supplemental Materials separating the summary table of study characteristics by population/outcome category. Thus, we believe that this comment has been adequately addressed.

The authors did not address the recommendation by myself and the journal editors to create a summary table for each assessment. Because of inadequate formatting, organization, and presentation of the information, I am not able to review table 3 (findings for all studies combined) or critically review this manuscript. Well-designed summary tables are needed for each assessment to understand the discussion of the results and whether the database of studies supports the authors' conclusions. (See recent IARC monograph tables for examples on presenting results and study characteristics).

In response to this comment, we have created the summary table for each population/outcome assessment and included these in the Supplemental Materials (Supplemental Table 99). We chose to include this in the Supplemental Materials due to the 113 pages of required space with the inclusion of this stratified table (primarily because of repetition of studies that appeared in multiple population/outcome categories). We believe that the inclusion of these stratified tables has addressed this comment. We also note that we would be willing to include this table in the main text if deemed necessary by the journal editor; however, due to the required space allocation we believe the location within supplemental materials may be more appropriate.

Reasons include the following:

• The meta-analysis includes only 9 of 24 studies of childhood asthma and formaldehyde; the strength of the evidence of the other 15 studies is not transparent. The supplemental figures mix findings from studies included in the meta-analysis (additional risk estimates) and studies with those not included in the meta-analysis. The scatter plots are not self-explanatory without information from a summary table (e.g., table 3, which is difficult to read). The discussion of the findings of the studies not included in the meta-analysis is inadequate.

It is unclear what Reviewer #2 is referring to by the “strength of the evidence of the other 15 studies.” In systematic review, the only step where individual studies are evaluated is the assessment of risk of bias. Figure 4 displays the risk of bias ratings for all studies, organized by population and outcome. Studies that were included in the meta-analysis are identified with an asterisk, thereby enabling a comparison of individual study quality for studies included in the meta-analysis versus those not included. Furthermore, Table 3b provides the study characteristics of included studies and stratifies by studies considered in the meta-analysis (presented first) versus those not. We understand that Table 3 contains a large amount of information and thus may be difficult for the reader to digest; however, we note that some of this information was added based on Reviewer #2’s comments during the second round of peer review when they suggested the further addition of the Confounders and Results columns. While we agree that the addition of this information is useful to the reader to interpret and evaluate the included studies, this may hinder the efficient synthesis of information provided in this particular table.

The supplemental figures display all other data on the same scale that may be directly comparable in an effort to consider the entire body of evidence when rating the quality and strength of evidence. Reviewer #2 is correct that other risk estimates from the meta-analysis studies are included in these figures; however, this is because these risk estimates may be directly compared with those from other studies. Additional risk estimates in the same study should not be excluded from consideration simply because one estimate from the study has been included in a meta-analysis. The intent of these scatter plots are to directly compare similar results on the same scale across different studies. It is unclear to the authors: (1) why these scatter plots are not interpretable without a summary table, and (2) what the specific recommended edit would be to appropriately address this.

To interpret the quality of individual studies, Figure 4 displays the risk of bias ratings for each study where these estimates were extracted. Again, aligning with best practices in systematic review (such as Cochrane and GRADE), risk of bias assessment is the only step where individual study quality is rated – quality and strength of the evidence is conducted over the entire body of available scientific evidence.

Lastly, the meta-analysis is but a single consideration in the evaluation of data across all included studies. The meta-analysis does not solely drive the rating of evidence; its results must be interpreted in the context of other data (presented in scatterplots when possible) that could not be combined into a meta-analysis. Results from a study could be excluded from the meta-analysis for several reasons unrelated to study quality (for instance if reported on a different scale that cannot be standardized with other estimates included in the meta-analysis). It is important to consider the overall body of evidence when coming to final conclusions regarding the quality and strength of evidence. Thus, our discussion of included studies does not dichotomize studies included versus not included in the meta-analysis, but maintains focus on the overall body of evidence for all included studies.

• The meta-analysis of childhood asthma exacerbation and symptoms included only 5 of 23 studies. The presentation of results suffers similar problems as discussed above.

Figure 4 displays the risk of bias ratings for all studies, organized by population and outcome. Studies that were included in the meta-analysis are identified with an asterisk, thereby enabling a comparison of individual study quality for studies included in the meta-analysis versus those not included. Our response above covers these issues raised by the reviewer.

• The discussion of the findings from adult studies is more comprehensive than that of the childhood studies not included in the meta-analyses. However, there is no comprehensive resource for the findings across studies in adults (except in Table 3, which is hard to read). Results from the studies are reported in multiple figures that are in the supplement files and thus not reader-friendly. The authors note the variety of exposure categories made it challenging to easily compare across different studies, thus, it is unclear why the authors are reluctant to make changes to improve the presentation of the data.

Based on Reviewer #2’s comments over several rounds of peer review, we have addressed this recommendation in the manuscript main text by reducing content in Table 3 by splitting the table of characteristics into two separate tables: one that identifies the categorization of studies by study population and outcome (Table 3a) and one that includes detailed study characteristics of each study (Table 3b). We have also created the summary table for each population/outcome assessment and included these in the Supplemental Materials (Supplemental Table 99). We chose to include this in the Supplemental Materials due to the 113 pages of required space with the inclusion of this stratified table (primarily because of repetition of studies that appeared in multiple population/outcome categories). We believe that the inclusion of these stratified tables has addressed this comment.

Results from the studies are reported in multiple figures in order to graphically display similar data on the same scales for ease of comparability. It would not make sense to plot these data all on the same figure, as they are reported on different scales and would not be interpretable collectively. In displaying study findings, we have followed Cochrane guidance1 as well as the methods as outlined in our study protocol2 regarding data analysis and display methods used to supplement the meta-analysis.

1 McKenzie JE, Brennan SE. Chapter 12: Synthesizing and presenting findings using other methods. In: Higgins JPT, Thomas J, Chandler J, Cumpston M, Li T, Page MJ, Welch VA (editors). Cochrane Handbook for Systematic Reviews of Interventionsversion 6.1 (updated September 2020). Cochrane, 2020. Available from [www.training.cochrane.org/handbook](http://www.training.cochrane.org/handbook).

2 Lam et al. Applying the Navigation Guide Systematic Review Methodology, Case Study #6: Association between Formaldehyde Exposure and Asthma. 2016. Available from: http://www.crd.york.ac.uk/PROSPERO/; Record ID #38766, CRD 42016038766
